# A Catalytic Three‐Component Aminofluorination of Unactivated Alkenes with Electron‐Rich Amino Sources

**DOI:** 10.1002/advs.202305006

**Published:** 2024-01-16

**Authors:** Junchao Dong, Yujie Liang, Yang Li, Wei Guan, Qian Zhang, Junkai Fu

**Affiliations:** ^1^ Jilin Province Key Laboratory of Organic Functional Molecular Design & Synthesis and Institute of Functional Material Chemistry Department of Chemistry Northeast Normal University Changchun 130024 P. R. China; ^2^ Institute of Functional Material Chemistry Department of Chemistry Northeast Normal University Changchun 130024 P. R. China; ^3^ Warshel Institute for Computational Biology and School of Life and Health Sciences School of Medicine The Chinese University of Hong Kong Shenzhen 518172 P. R. China

**Keywords:** aminofluorination, C–F reductive elimination, copper(III), unactivated alkenes, β‐fluoroamine

## Abstract

We present herein a copper‐catalyzed three‐component aminofluorination of unactivated alkenes with *N*‐bromodialkylamines and readily available nucleophilic fluoride under the assistance of a bidentate auxiliary. This protocol exhibits excellent functional group tolerance toward a wide range of unactivated alkenes and *N*‐bromodialkylamines to furnish the corresponding β‐fluoroalkylamines in a highly regio‐ and diastereoselective manner. The appropriate choice of nucleophilic fluoro source is essential to make this reaction a reality. Further DFT calculations show that the exothermic ion exchange between external fluoride ion and Cu(II) intermediate provides additional driving force to the irreversible migratory insertion, which offsets the unfavorable reaction energetics associated with the subsequent C(*sp^3^*)–F reductive elimination. This finding offers a new avenue to catalytic intermolecular aminofluorination of unactivated alkenes with electron‐rich amino sources via a remarkable reductive elimination of Cu(III) species to forge the C(*sp^3^*)–F bonds.

## Graphical abstract

A catalytic three‐component aminofluorination of unactivated alkenes has been achieved in the presence of copper catalyst and a tethered bidentate auxiliary via a remarkable C(sp^3^)−Cu(III)−F reductive elimination. *N*‐Bromodialkylamines serve as electron‐rich amino sources while readily available nucleophilic fluoride is used as fluoro source, providing an efficient access to β‐fluoroalkylamines in an excellent regio‐ and diastereoselective fashion.

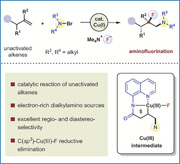

## 1 Introduction

**Figure 1:**
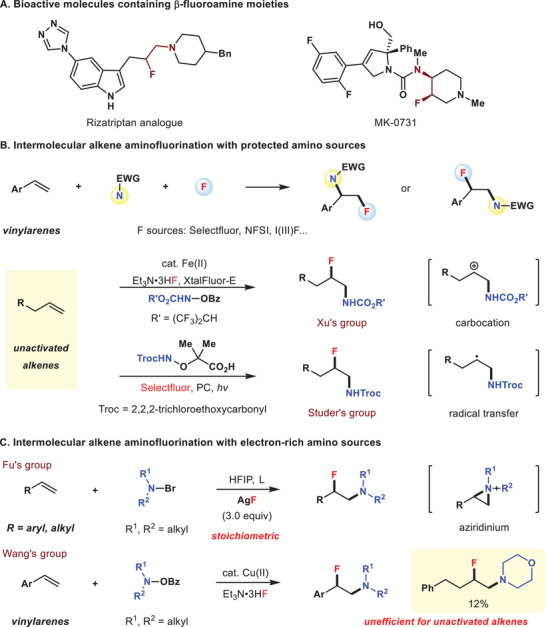
The intermolecular aminofluorination of alkenes.

The intermolecular aminofluorination of alkenes provides an efficient access to β‐fluoroamines,^[^ ^1^ ^]^ that are prevalent in bioactive molecules,^[^ ^2^ ^]^ e.g., Rizatriptan analog for the treatment of migraines,^[^ ^3^ ^]^ MK‐0731 as kinesin spindle protein (KSP) inhibitor^[^ ^4^ ^]^ (**Figure** **1A**). The introduction of fluorine atom can modulate the chemical stability, lipophilicity, and metabolic stability of the targeted molecules,^[^ ^5^ ^]^ and modify the *PK^a^* of the simultaneously incorporated amine nitrogen.^[^ ^6^ ^]^ In this context, the intermolecular alkene aminofluorination with amino sources protected by electron‐withdrawing groups have been well explored. Stavber,^[^ ^7a^ ^]^ Liu,^[^ ^7b^ ^]^ Nevado,^[^ ^7c^ ^]^ and Zhang^[^ ^7d^ ^]^ have done pioneering work on vinylarenes, while Xu^[^ ^8a^ ^]^ and Studer^[^ ^8b^ ^]^ realized aminofluorination reactions on more challenging unactivated alkenes wherein the fluorine atoms were introduced via nucleophilic fluorination of carbocation species and fluorine‐atom radical transfer process, respectively (Figure 1B). In comparison, the installation of fluoride along with an unprotected amine on alkenes has posed a long term challenge,^[^ ^9^ ^]^ but is highly desirable considering the ubiquity of tertiary alkylamines in organic chemistry.^[^ ^10^ ^]^ In traditional ionic way, the electron‐rich dialkyl amines are easily oxidized in the presence of electronic fluorinating reagents.^[^ ^11^ ^]^ What's more, as strong Lewis bases, the dialkyl amines would frequently poison the transition metals.^[^ ^12^ ^]^

Recently, by employing an umpolung strategy via electrophilic aminium radical cations (ARCs),^[^ ^13^ ^]^ we have realized the first intermolecular three‐component alkene aminofluorination with *N*‐bromodialkylamines to obtain fluorinated tertiary alkylamines, and a variety of vinylarenes and unactivated alkenes are compatible. Stoichiometric AgF is necessary to serve as both reductive transition metal and nucleophilic fluoro source to react with an aziridinium intermediate.^[^ ^14^ ^]^ Shortly thereafter, Wang's group reported a copper‐catalyzed intermolecular alkene aminofluorination with *O*‐benzoyl‐*N*,*N*‐dialkylhydroxylamines and Et_3_N·3HF.^[^ ^15^ ^]^ This elegant reaction worked well for vinylarenes, but suffered from extremely low efficiency for unactivated alkene with only one example (Figure 1C). Thus, searching for a catalytic version for simultaneously incorporating fluoride and unprotected amine across unactivated alkenes is highly desirable to offer structurally diverse β‐fluoroalkylamines.

**Figure 2:**
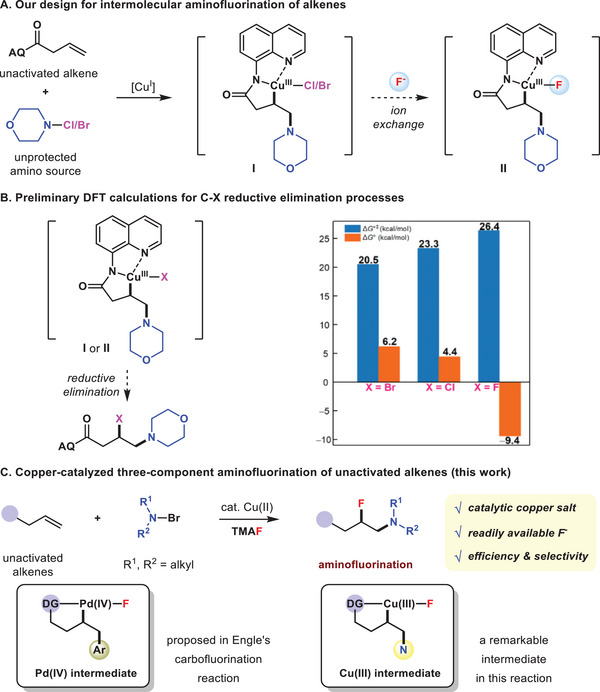
The design and preliminary evaluation of the intermolecular aminofluorination of unactivated alkenes with electron‐rich amino sources.

On the basis of our ongoing efforts in aminyl radical chemistry,^[^ ^14^ ^], [^ ^16^ ^]^ we envision that in the presence of an external fluoride ion, a reaction sequence involving ion exchange of high‐valent copper intermediate **I** followed by reductive elimination of **II** may take place to achieve a catalytic three‐component aminofluorination reaction of unactivated alkenes with electron‐rich amino sources (**Figure** **2A**). However, due to fluorine's high electronegativity and small size, metal–fluorine bonds are significantly polarized,^[^ ^17^ ^]^ resulting in C–F reductive elimination kinetically challenging.^[^ ^18^ ^]^ Thus, for a XFCu(III)–C(*sp^3^*) complex, C(*sp^3^*)–X reductive elimination is usually preferential,^[^ ^19^, ^20^ ^]^ and only limited examples propose C(*sp^3^*)–F reductive elimination with unclear mechanisms.^[^ ^21^ ^]^ Preliminary gas‐phase density functional theory (DFT) calculations showe that the Gibbs activation energy (Δ*G*°^‡^) values of C−X (X = Br, Cl, F) reductive eliminations are 20.5, 23.3 and 26.4 kcal mol^−1^, respectively (Figure 2B, for details, see Figure S1, Supporting Information). Thus, the C–Br/Cl bond formation may occur prior to C─F bond formation. On the other hand, a moderate Δ*G*°^‡^ value of 26.4 kcal mol^−1^, probably benefited from the coordination of electronically rich bidentate auxiliary to the Cu(III) center, also indicates that this aminofluorination reaction is theoretically feasible by tuning the reaction conditions. It should be noted that Engle and co‐workers have recently reported 1,2‐carbofluorination reactions of unactivated alkenes with electrophilic NFSI via challenging C(*sp^3^*)−F reductive elimination from a Pd(IV) intermediate.^[^ ^22^ ^]^

Herein, by using *N*‐bromodialkylamines^[^ ^23^ ^]^ and readily available nucleophilic fluoride as the reaction components, a copper‐catalyzed three‐component aminofluorination of unactivated alkenes with electron‐rich amino sources is reported (Figure 2C). β‐Fluoroalkylamines were efficiently obtained with excellent regio‐ and diastereoselectivity. Theoretical calculation results revealed the key role of nucleophilic fluoride, providing additional driving force via exothermic ion exchange with Cu(II) species to the irreversible migratory insertion of olefin into FCu(II)···N• bond, which finally accelerated the kinetically unfavorable C−F reductive elimination of Cu(III) intermediate.

## 2 Results and Discussion

### 2.1 Reaction Conditions Screening

The study was initiated by treating 4‐bromomorpholine **1a** with 8‐aminoquinoline (AQ) amide‐tethered alkene **2a** ^[^ ^24^ ^]^ in the presence of CuI and external fluoro sources in CH_3_CN at 70 °C. Employment of NaF as the fluoro source produced a small amount (14%) of aminobromination by‐product **4**, while major starting material was recovered in the presence of AgF (**Table** **1**, entries 1–2). Delightedly, it was found that the reaction with Et_3_N⋅3HF furnished the desired aminofluorination product **3a** in 27% yield, along with the generation of **4** (12%) and allylic amination by‐product **5** (10%) (entry 3). Moreover, the utilization of tetramethylammonium fluoride (TMAF) could slightly improve the yield of **3a** to 32% (entry 4). Further screening of reaction solvents showed that the reaction in 1,4‐dioxane gave a similar result as that in CH_3_CN; however, the yield of **5** was dramatically increased to 34% in toluene, likely due to the strong basicity of the fluoride ion in non‐polar solvent (entries 5–6). The alcohol solvent is known to coordinate fluoride as a “flexible” fluoride form with good nucleophilicity and low basicity.^[^ ^25^ ^]^ So we tested a series of alcohol solvents (entries 7–9). Despite the reactions in bulky *^t^* BuOH and *^i^* PrOH led to very low yields, a markedly improved reaction efficiency was observed in EtOH, produing β‐fluoroamine **3a** in 80% yield. Other copper salts, e.g., CuCl and Cu(OAc)_2_, gave inferior yields (entries 10–11), while alternative reductive transition metals including Co(II) and Fe(II) only offered arene bromination by‐product **6** (entries 12–13). The necessity of the copper catalyst for this transformation was further demonstrated via a control experiment completely excluding copper salts, which gave no desired product (entry 14). A preliminary screening of the reaction temperature indicated that 70 °C was the best choice (entries 15–16). Moreover, replacing 4‐bromomorpholine **1a** with 4‐chloromorpholine **1a′** could also deliver the desired product **3a**, albeit in a moderate yield of 47% (entry 17). Of note, the aminofluorination reactions of alkenes bearing other bidentate auxiliaries^[^ ^26^ ^]^ rather than 8‐aminoquinoline amide all failed, while the reactions of simple alkenes without bidentate auxiliaries, such as 4‐phenylbutene, styrene, and 2‐vinyl phenyl acetate in the presence of extra ligands also proved to be ineffective (for more details, see Table S1, Supporting Information). The asymmetric version of this reaction has been evaluated by addition of chair ligands, but the desired chair product was not obtained (for more details, see Table S2, Supporting Information).

**Table 1:** Optimization of the reaction conditions.

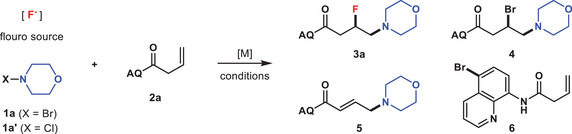				
Entry	Catalyst	Solvent	F^−^ source	Yield%(**3a**/**4**/**5**/**2a**)
1	CuI	CH_3_CN	NaF	0/14/0/51
2	CuI	CH_3_CN	AgF	0/0/0/62
3	CuI	CH_3_CN	Et_3_N⋅3HF	27/12/10/16
4	CuI	CH_3_CN	TMAF	32/16/15/5
5	CuI	1,4‐dioxane	TMAF	25/5/10/28
6	CuI	toluene	TMAF	8/12/34/20
7	CuI	*^t^* BuOH	TMAF	5/0/5/75
8	CuI	*^i^* PrOH	TMAF	19/0/0/55
**9**	**CuI**	**EtOH**	**TMAF**	**80/0/2/0**
10	CuCl	EtOH	TMAF	62/10/3/0
11	Cu(OAc)_2_	EtOH	TMAF	51/0/3/11
12	Co(acac)_2_	EtOH	TMAF	0/0/0/79 (10)^a)^
13	FeCl_2_	EtOH	TMAF	0/0/0/52 (40)^a)^
14	‐	EtOH	TMAF	0/0/0/90
15^b)^	CuI	EtOH	TMAF	59/0/5/0
16^c)^	CuI	EtOH	TMAF	64/0/5/0
17^d)^	CuI	EtOH	TMAF	47/0/3/20

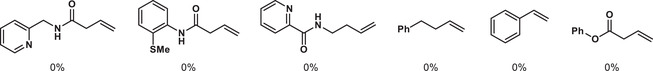
Conditions: **1a** (0.40 mmol) dissolved in solvent (2.0 mL) was added by syringe pump into the mixture of **2a** (0.20 mmol) and nucleophilic fluorides (0.50 mmol) in solvent (3.0 mL) in the presence of catalyst (0.06 mmol) at 70 °C. Isolated yields;

^a)^ Yield of **6** in parentheses;

^b)^ At 50 °C;

^c)^ At 80 °C;

^d)^ With 4‐chloromorpholine **1a′**.

**Scheme 1:**
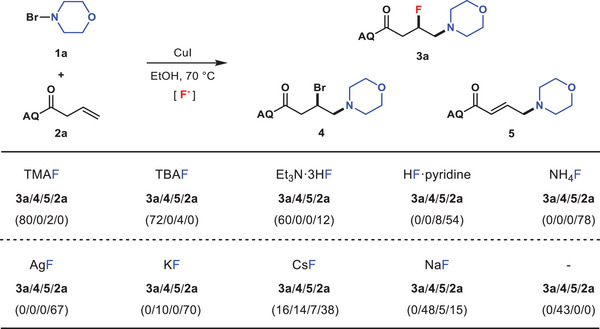
Screening of nucleophilic fluoro sources. Standard conditions: **1a** (0.40 mmol) dissolved in EtOH (2.0 mL) was added by syringe pump into the mixture of **2a** (0.20 mmol) and nucleophilic fluorides (0.50 mmol) in EtOH (3.0 mL) in the presence of CuI (0.06 mmol) at 70 °C.

The above experimental results showed an important role of nucleophilic fluorinating reagents for this reaction. To study the effect in detail, a series of control experiments were conducted under the optimized reaction conditions by varying the nucleophilic fluorides (**Scheme** **1**). Besides TMAF, tetrabutylammonium fluoride (TBAF) and Et_3_N⋅3HF also proved to be good partners to produce **3a** in 72% and 60% yields, respectively. However, employment of either HF⋅pyridine or NH_4_F failed to give any desired product, with the majority of the mass balance attributed to the recovery of **2a**. The reaction with AgF was ineffective, probably because of the precipitation of CuI with AgF. In addition, the frequently used alkali‐metal fluorides have been tested. The alkene substrate **2a** exhibited either low or no reactivity when KF or CsF used as the fluoro source, while β‐bromoamine **4** dominated the product distribution in the presence of NaF. The experiment excluding external fluoride ion produced β‐bromoamine **4** in 43% yield.

### 2.2 Substrate Scope and Derivatizations

**Figure 3:**
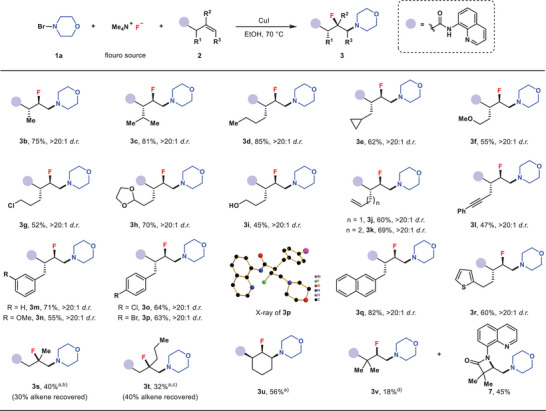
Substrate scope of unactivated alkenes. Standard conditions: **1a** (0.40 mmol) dissolved in EtOH (2.0 mL) was added by syringe pump into the mixture of **2** (0.20 mmol) and TMAF (0.50 mmol) in EtOH (3.0 mL) in the presence of CuI (0.06 mmol) at 70 °C. Isolated yields. a) **1a** (0.60 mmol) dissolved in MeOH (1.0 mL) was added into the reaction mixture in MeOH (1.0 mL). b) IPrCuCl (0.06 mmol) was used. c) CuTC (0.06 mmol) was used. d) **1a′** (0.60 mmol) dissolved in MeOH (1.0 mL) was added by syringe pump into the mixture of **2** (0.20 mmol), ethyl isocyanoacetate (0.06 mmol) and TMAF (0.50 mmol) in MeOH (1.0 mL) with Cu(OTf)_2_ (0.06 mmol) at 70 °C.

With the optimized reaction conditions in hand, the substrate scope of unactivated alkenes was investigated. As shown in **Figure** **3**, the α‐substituted alkenes with methyl, isopropyl and *n*‐butyl groups furnished the corresponding vicinal fluoroamines **3b**–**3d** in good yields and excellent diastereoselectivity. A variety of functional groups including cyclopropyl, methyl ether, chloro atom, acetal and even free hydroxyl group were compatible to deliver products **3e**–**3i**. For diene or enyne compounds, the aminofluorination reactions could chemoselectively occur across the β‐γ olefin moieties (**3j**–**3l**), likely due to the preference of the five‐membered metallacycle.^[^ ^27^ ^]^ Electronically diverse phenyl rings with both electron‐donating (–OMe) and –withdrawing (–Cl, –Br) substituents were well tolerated to produce β‐fluoroamines **3m**–**3p**, and the relative stereochemistry of **3p** was further established by X‐ray crystallographic analysis.^[^ ^28^ ^]^ Alkenyl amides bearing either naphthyl or aromatic heterocycle (e.g., thiophene‐yl) could be smoothly transformed into vicinal fluoroamines **3q** and **3r** in 82% and 60% yields, respectively. It is noteworthy that 1,1‐disubstituted alkenes proved to be suitable substrates to produce **3s** and **3t** bearing tertiary carbon–fluorine stereocenters in moderate yields, and part of the alkene substrate was recovered. Subjecting cyclohexene‐based amide to the optimized reaction conditions successfully furnished the desired aminofluorination product **3u** in 56% yield as a single *cis*‐isomer. The sterically hindered α,α‐dimethyl substituted alkene was also tested. However, the aminofluorination reaction worked in low efficiency to form the desired product **3v** in only 18% yield using 4‐chloromorpholine **1a′** as the amino source; instead, an intermolecular addition/intramolecular cyclization four‐membered lactam **7** ^[^ ^27b,c^ ^]^ was generated in 45% yield. Unfortunately, amides with γ‐ε olefin moiety failed in this reaction, showing that a six‐membered metallacycle was disfavored.^[^ ^29^ ^]^

**Figure 4:**
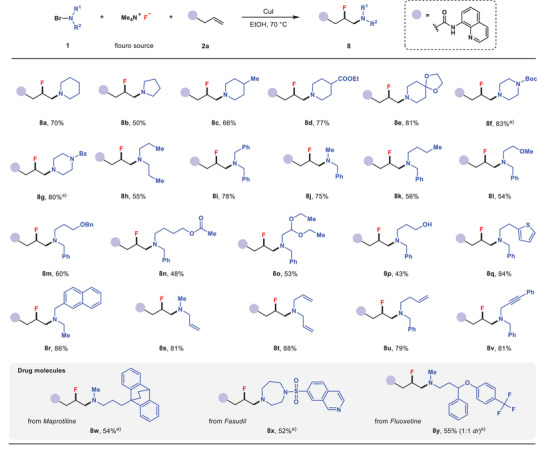
Substrate scope of *N*‐bromodialkylamines. Standard conditions: **1** (0.40 mmol) dissolved in EtOH (2.0 mL) was added by syringe pump into the mixture of **2a** (0.20 mmol) and TMAF (0.50 mmol) in EtOH (3.0 mL) in the presence of CuI (0.06 mmol) at 70 °C. Isolated yields. a) **1** dissolved in DCM (2.0 mL) was added into the reaction mixture.

Next, a range of *N*‐bromodialkylamines were investigated as the reaction partners (**Figure** **4**). *N*‐Bromodialkylamines pre‐prepared from piperidine or pyrrolidine could be directly used in this aminofluorination reaction to give **8a** and **8b** in 70% and 50% yields, respectively. Functionalized cyclic *N*‐bromodialkylamines tethering methyl (**8c**), ester (**8d**), ketal (**8e**), carbamate (**8f**) and amide (**8g**) groups all proved to be suitable partners for this reaction. Acyclic dialkylamines, such as dipropylamine, dibenzylamine, *N*‐methylbenzylamine, and *N*‐n‐butylbenzylamine could all be converted into the corresponding *N*‐bromodialkylamines, and then participated in the aminofluorination reactions to form β‐fluoroamines **8h**–**8k**. Additionally, this reaction was compatible with acyclic *N*‐bromodialkylamines bearing diverse functionalities including ether (**8l**, **8m**), ester (**8n**), acetal (**8o**), free hydroxyl (**8p**), thiophene‐yl (**8q**), and naphthyl (**8r**) groups. It should be noted that *N*‐bromodialkylamines containing allyl, homoallyl, or propargyl groups were all found to be suitable aminating reagents to produce vicinal fluoroamines **8s**‐**8v**, in which the remaining unsaturated carbon–carbon bonds allowed for further functional group inter‐conversion. This methodology could also be applicable to the *N*‐bromodialkylamines derived from bioactive drug molecules, such as Maprotiline (**8w**), Fasudil (**8x**), and Fluoxetine (**8y**), showing the potential of this protocol in late‐stage modification of pharmaceuticals.

**Scheme 2:**
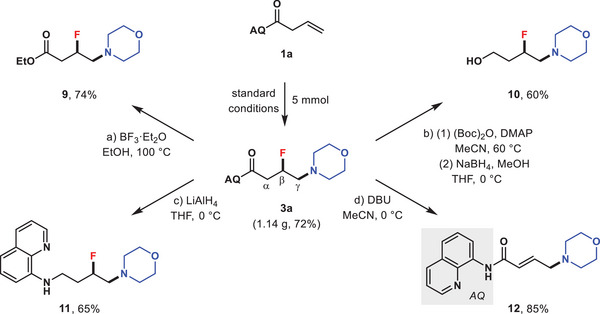
Synthetic utility. Conditions: a) BF_3_⋅Et_2_O (6.0 eq), EtOH, 100 °C, 15 h; b) (1) (Boc)_2_O (2.0 eq), DMAP (0.2 eq), MeCN, 60 °C, 2 h; (2) NaBH_4_ (3.0 eq), MeOH (3.0 eq), THF, 0 °C, 2 h; c) LiAlH_4_ (3.0 eq), THF, 0°C, 2 h; d) DBU (2.0 eq), MeCN, 0°C, 1 h

To further demonstrate the synthetic utility of this protocol, representative derivatizations of the resultant β‐fluoroamines were performed. As delineated in **Scheme** **2**, a gram‐scale (5 mmol) synthesis of **3a** was carried out with a slight decrease in yield (72%). When **3a** was treated with BF_3_⋅Et_2_O in ethanol at 100 °C, the amide moiety was smoothly transformed into ester to give **9** in 74% yield. Protection of the NH in 8‐aminoquinoline (AQ) amide with *t*‐butyloxy carbonyl (Boc) group followed by a reduction with NaBH_4_ in mixed methanol and THF resulted in the formation of primary alcohol **10**. Subjection of **3a** to reductive reaction conditions with LiAlH_4_ could directly convert the amide into secondary amine, and compound **11** was isolated in 65% yield. In addition, basic conditions could promote a H–F elimination to deliver α,β‐unsaturated amide **12** in 85% yield.

### 2.3 Mechanistic Studies

**Scheme 3:**
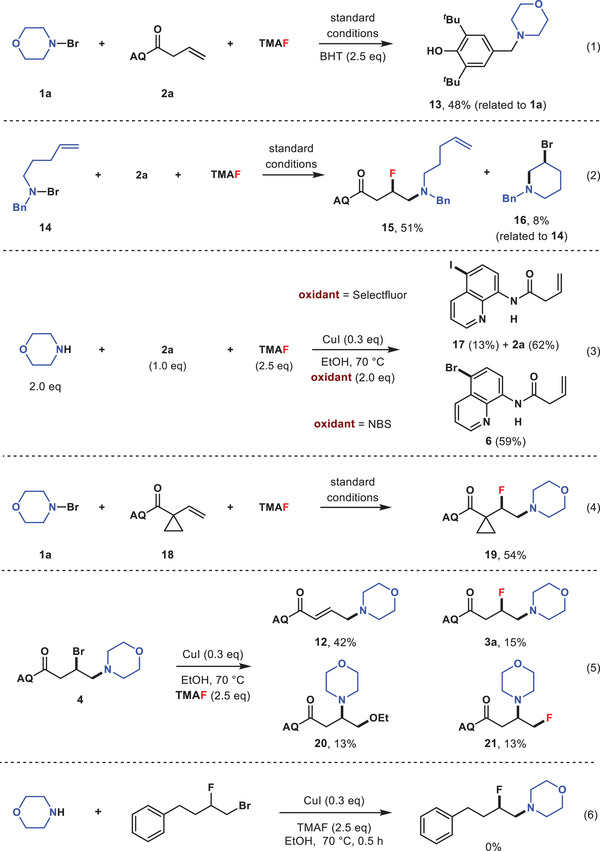
Control experiments.

Several control experiments were performed to gain insight into the reaction mechanism (**Scheme** **3**). Addition of butylated hydroxytoluene (BHT) into the reaction mixture completely suppressed the aminofluorination reaction, and the adduct **13** was isolated (entry 1). Subjection of *N*‐bromodialkylamine **14** bearing a δ−ε double bond to the optimized reaction conditions resulted in the formation of β‐fluoroamine **15** along with a small amount (8%) of intramolecular aminobromination product **16** (entry 2).^[^ ^30^ ^]^ Taken together, these results demonstrated that aminyl radical intermediates might be involved in the aminofluorination reactions. To identify whether the aminyl radicals were generated from the direct oxidation of dialkylamines^[^ ^12^ ^]^ rather than from pre‐prepared *N*‐bromodialkylamines, the reactions of morpholine, alkene **2a** and TMAF were conducted in the presence of external oxidants (entry 3). Unfortunately, with Selectfluor, iodization of AQ moiety occurred to offer **17** in 13% yield along with 62% alkene substrate **2a** recovered; when employing *N*‐bromosuccinimide (NBS) as the oxidant, bromination of AQ moiety dominated to produce **6** as the major by‐product. In addition, a radical clock experiment^[^ ^31^ ^]^ with cyclopropyl alkene **18** as the substrate delivered β‐fluoroamine **19** in 54% yield, and no ring‐opening product was detected (entry 4), indicating that either a secondary carbon radical was not generated or the newly formed carbon radical was quickly trapped prior to ring‐opening of cyclopropane.^[^ ^16^, ^32^ ^]^ To exclude the possibility of a reaction sequence involving alkene aminobromination followed by a regioselective substitution of the bromide with fluoride ion, vicinal bromoamine **4** was treated with TMAF and catalytic CuI in ethanol at 70 °C (entry 5). Besides β‐fluoramine isomers **3a** (15%) and **21** (13%), H–F elimination product **12** (42%) and β‐etheramine **20** were also obtained, suggesting that the aminofluorination reaction did not proceed through a vicinal bromoamine intermediate. Moreover, further treatment of β‐bromofluoride under standard conditions in the presence of morpholine did not lead to the formation of β‐fluoroamine, which could rule out the possible reaction pathway invovling alkene bromofluorination followed by amination of the primary bromide (entry 6).

### 2.4 Theoretical Calculations

**Figure 5:**
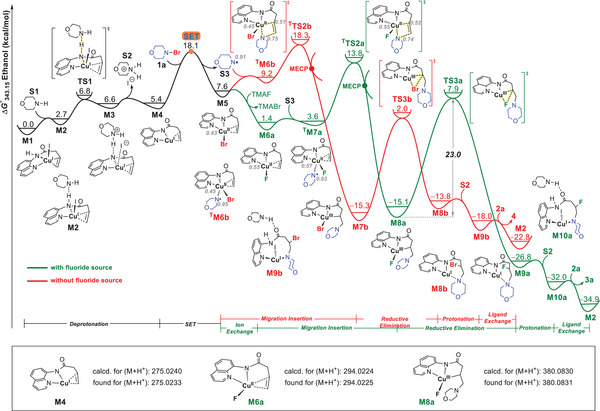
Gibbs energy profiles (Δ*G*°_343.15_) with and without nucleophilic fluoride TMAF calculated at the SMD(ethanol)/(U)M06/[6‐311++G(d,p)/SDD(Cu,I)]//SMD(ethanol)/(U)M06/[6‐31G(d)/LanL2DZ (Cu,I)] level. Spin densities are given in grey italic font.

To gain a deeper understanding of the Cu‐catalyzed three‐component aminofluorination of alkenyl amides with electron‐rich amino source, several competitive reaction mechanisms were evaluated through DFT calculations at the SMD^[^ ^33^ ^]^(ethanol)/(U)M06^[^ ^34^ ^]^/[6‐311++G(d,p)/SDD^[^ ^35^ ^]^(Cu,I)]//SMD(ethanol)/(U)M06/[6‐31G(d)/LanL2DZ^[^ ^36^ ^]^(Cu,I)] level (**Figure** **5**, see the Supporting Information for computational details). By comparing the Gibbs energy profiles with and without nucleophilic fluoride TMAF, the most favorable catalytic cycle consists of seven key elementary steps: deprotonation, single‐electron transfer (SET), ion exchange, migratory insertion, reductive elimination, protonation, and ligand exchange, as depicted with black and green lines in Figure 5.

First, a deprotonation process of **M1** by morpholine **S1** (generated in small amounts during the reactions, detected by both GC−MS and LC−MS) occurs via **TS1** to afford **M3** followed by morpholine hydroiodide **S2** dissociation into an active Cu(I) amino intermediate **M4**, with a Gibbs activation energy (Δ*G*°^‡^) of 6.8 kcal/mol^−1^ and a positive Gibbs free energy change (Δ*G*°) of 5.4 kcal mol^−1^, respectively. Subsequently, the SET between **M4** and 4‐bromomorpholine **1a** requires a much smaller Δ*G*°^‡^ value by 8.9 kcal mol^−1^ than the direct oxidative addition of N−Br bond in **1a** to the Cu(I) center of **M4** to form a Cu(III)−N complex (Figure S2, Supporting Information). Specifically, the favorable single‐electron oxidation of **M4** by **1a** can provide a doublet Cu(II) bromide **M5** and an aminyl radical **S3**, with a moderate Δ*G*°^‡^ value of 12.7 kcal mol^−1[^ ^37^ ^]^ and a positive Δ*G*° value of 2.2 kcal mol^−1^, respectively. The spin densities of **M5** and **S3** are mainly localized on Cu and N with 0.43 and 0.91, respectively. The generation of aminyl radical is consistent with the above control experiments.

From **M5**, the reaction pathways were considered with and without TMAF. In the presence of TMAF, the bromine‐fluoride ion exchange occurs exothermically between **M5** and TMAF to generate a doublet Cu(II) fluoride **M6a** with a negative Δ*G*° value of −6.2 kcal mol^−1^. Next, **M6a** can interact with **S3** to generate three possible intermediates, a closed‐shell singlet Cu(III) fluoride **M7a**, an antiferromagnetic singlet Cu(II) fluoride **^S^M7a** and a triplet Cu(II) fluoride **^T^M7a**. **^T^M7a** is more stable than **M7a** and **^S^M7a** by 18.0 and 4.3 kcal mol^−1^, respectively (Figure S3, Supporting Information). The following migratory insertion of olefin moiety of **^T^M7a** into the Cu(II)···N• bond through **^T^TS2a** and minimum energy crossing point (MECP) can form a relative stable singlet amino Cu(III) fluoride **M8a** (more stable than triplet **^T^M8a** by 8.0 kcal mol^−1^, Figure S3, Supporting Information) with a moderate Δ*G*°^‡^ value of 12.4 kcal mol^−1^ relative to **M6a**. In such migratory insertion, spin inversion between the triplet and singlet energy profiles effectively decreases the activation barrier and alters the ground‐state profile from the triplet state to the singlet state via a MECP.^[^ ^38^ ^]^ Then, C−F reductive elimination of **M8a** occurs through **TS3a** to form a three‐coordinate Cu(I) intermediate **M9a** with a Δ*G*°^‡^ value of 23.0 kcal mol^−1^. Finally, the protonation and ligand exchange processes can release the desired β‐fluoroamine **3a** and regenerate **M2** to restart the catalytic cycle. Overall, **M8a** is the TOF‐determining intermediate (TDI), while **TS3a** is the TOF‐determining transition state (TDTS), thus the C−F reductive elimination is the rate‐determining step of the catalytic cycle. According to the energetic span model,^[^ ^39^ ^]^ the TDTS‐TDI energy difference defines the apparent activation energy of the cycle (Δ*G*°^‡^ = 23.0 kcal mol^−1^). The total Δ*G*° value of this cycle is −37.6 kcal mol^−1^.

In the absence of TMAF, however, **M5** is coordinated preferentially with **S3** followed by olefin insertion into the Cu(II)···N• bond and reductive elimination to afford the β‐bromoamine **4** (see red line in Figure 5), which is consistent with the control experimental result without external fluoride ion shown in Scheme 1. The C−Br reductive elimination is the rate‐determining step of the catalytic cycle with a Δ*G*°^‡^ value of 17.3 kcal mol^−1^.

It should be noted that Cu(I) amino intermediate **M4**, Cu(II) fluoride **M6a**, and amino Cu(III) fluoride **M8a** could all be detected by ESI‐MS (for details, see Part 6 in Supporting Information), which strong supports our proposed mechanism.

**Figure 6:**
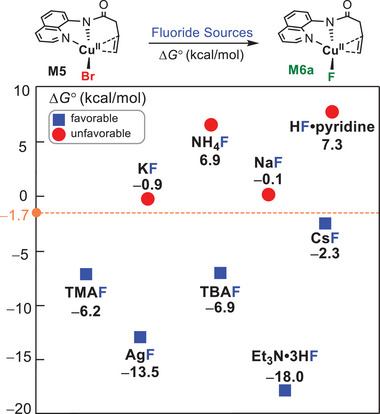
Gibbs energy (Δ*G*°_343.15_) of ion exchange between nucleophilic fluoro sources and Cu(II) bromide **M5** calculated at the SMD(ethanol)/(U)M06/[6‐311++G(d,p)/SDD(Cu,I,Ag,Cs)]//(U)M06/[6‐31G(d)/LanL2DZ (Cu,I,Ag,Cs)] level.

In the comparison between the energy profiles with and without TMAF, it can be found that the relative stability of **^T^TS2a** and **^T^TS2b** determines the feasibility of aminofluorination due to the irreversible migratory insertion. Without considering the driving force of ion exchange, the energy barriers of migratory insertion based on Cu(II) bromide **M5** and Cu(II) fluoride **M6a** are 10.7 and 12.4 kcal mol^−1^, respectively. It means that if such energy barrier difference of 1.7 kcal mol^−1^ can be offset by the enough driving force of ion exchange between **M5** and external fluoro source, the aminofluorination would be feasible. Armed with the above analysis, a series of nucleophilic fluoro sources were used to evaluate the Δ*G*° values of ion exchange with **M5**. As shown in **Figure** **6**, TMAF, AgF, TBAF, Et_3_N•3HF, CsF, KF and NaF rather than NH_4_F and HF•pyridine could spontaneously exchange ions with **M5** to provide **M6a**. However, the initial CuI could precipitate AgF. The free energies released by the ion exchange of KF and NaF with **M5** cannot meet the minimum energy requirement of 1.7 kcal mol^−1^. When CsF is employed as a fluoro source, competing pathways exist between aminofluorination and aminobromination because of the small Δ*G*° values of −2.3 kcal/mol^−1^. Thus, TMAF, TBAF and Et_3_N•3HF can effectively trigger this aminofluorination reaction. These DFT calculations align with the experimental results shown in Scheme 1. The suitable external fluoro source is the precondition of this aminofluorination reaction. Note that for some cases in Scheme 1 (e.g., HF•pyridine and NH_4_F), neither β‐fluoro‐ nor β‐bromoamines are obtained, which may be ascribed to an inhibiting effect of external fluoro source on the aminobromination reaction.

In addition, the catalytic cycle in which the 4‐bromomorpholine **1a** is replaced with 4‐chloromorpholine **1a′** as amino source has also been evaluated to deliver the desired aminofluorination product **3a** (Figures S4 and S5, Supporting Information). An ion exchange of nucleophilic TMAF with Cu(III) intermediate proved to be unfavorable as compared to that with Cu(II) intermediate (Figure S6, Supporting Information).

## 3 Conclusion

In summary, a catalytic three‐component aminofluorination of unactivated alkenes has been realized in the presence of copper catalyst and a tethered bidentate auxiliary. *N*‐Bromodialkylamines were employed as electron‐rich amino sources, while readily available TMAF served as nucleophilic fluoro source, providing a potential for ^18^F positron emission tomography (PET) image. This methodology exhibited a broad substrate scope and excellent functional group tolerance toward terminal, internal and 1,1‐disubstituted alkenes, as well as cyclic and acyclic *N*‐bromodialkylamines, delivering the corresponding alkylamines bearing adjacent secondary or tertiary fluorine stereocenters in a highly regio‐ and diastereoselective manner. Further control experiments and DFT calculations revealed a radical reaction pathway involving a remarkable Cu(III) intermediate. The success of kinetically challenging C(*sp^3^*)–Cu(III)–F reductive elimination process was attributed to the feasibility of irreversible migratory insertion process enabled by exothermic ion exchange between external fluoride ion and the Cu(II) intermediate.

## Conflict of Interest

The authors declare no conflict of interest.

## Supporting information

Supporting Information

## Data Availability

The data that support the findings of this study are available in the supplementary material of this article.
