# Supplementary material for: A Catalytic Three‐Component Aminofluorination of Unactivated Alkenes with Electron‐Rich Amino Sources
Source: Adv Sci (Weinh). 2024 Jan 16;11(12):2305006. doi: 10.1002/advs.202305006 (PMC10966579; doi:10.1002/advs.202305006)
Supplement: Supplementary file 1 — Supporting Information [file ADVS-11-2305006-s001.pdf]

ADVANCED SCIENCE Open Access

Supporting Information

for *Adv. Sci.*, DOI 10.1002/advs.202305006

A Catalytic Three-Component Aminofluorination of Unactivated Alkenes with Electron-Rich Amino Sources

Junchao Dong, Yujie Liang, Yang Li, Wei Guan, Qian Zhang and Junkai Fu**

Supporting Information

A Catalytic Three-Component Aminoflurination of Unactivated Alkenes with Electron-Rich Amino Sources

Junchao Dong,[a],# Yujie Liang,[b],# Yang Li,[a],[c],# Wei Guan,*[b] Qian Zhang,[a] and Junkai Fu*[a]

[a] Jilin Province Key Laboratory of Organic Functional Molecular Design & Synthesis and Institute of Functional Material Chemistry, Department of Chemistry, Northeast Normal University, Changchun 130024, P. R. China

[b] Institute of Functional Material Chemistry, Department of Chemistry, Northeast Normal University, Changchun 130024, P. R. China

[c] Warshel Institute for Computational Biology and School of Life and Health Sciences, School of Medicine, The Chinese University of Hong Kong, Shenzhen, Shenzhen 518172, P. R. China

Tel: +86 13578753505

fujk109@nenu.edu.cn

Table of Contents

Part 1: General information --S2

Part 2: Supplementary data --S3

Part 3: DFT calculations --S10

Part 4: General procedure and characteristic data for products **2a-8y** -----------------S79

Part 5: Procedure and characteristic data for compounds **9-12** ----------------------S106

Part 6: Control experiments and radical clock experiments
---------------------------S110

Part 7: References --S116

Part 8: NMR spectra --S117

Part 1: General information

Unless otherwise noted, all reactions were carried out under an argon atmosphere as well as anhydrous conditions, and all reagents were purchased from commercial suppliers without further purification. Anhydrous tetrahydrofuran (THF) and diethyl ether (Et_2O) were distilled from sodium-benzophenone. Anhydrous 1, 2-dichloroethane (DCE), dichloromethane (DCM), acetonitrile (CH_3CN) and dimethylformamide (DMF) were distilled from calcium hydride. Anhydrous toluene was distilled from sodium. Anhydrous ethanol was distilled from sodium. Anhydrous chloroform ($CHCl_3$) was distilled from phosphorus pentoxide. CuI (99.99%) was purchased from Aldrich.

Reactions were monitored by Thin Layer Chromatography (TLC) on plates (GF254) supplied by Yantai Chemicals (China) visualized by UV or stained with ethanolic solution of phosphomolybdic acid and basic solution of $KMnO_4$. The products were purified by column chromatography over silica gel (300-400 size).

NMR spectra were recorded on a Brüker Advance 600 (^{1}H: 600 MHz, ^{13}C:150 MHz), Brüker Advance 500 (^{1}H: 500 MHz, ^{13}C: 125 MHz), and TMS was used as internal standard. The following abbreviations were used to explain the multiplicities: s = singlet, d = doublet, t = triplet, q = quartet, dd = doublet of doublets, m = multiplet, br = broad.

IR spectra were recorded on an IRPrestige-21 FTIR spectrometer. High resolution mass spectrometric (HRMS) data was recorded on Brüker Apex IV RTMS by using ESI method.

Part 2：Supplementary data

Table S1. The reactions of alkenes without bidentate auxiliary in the presence of an extra ligand.

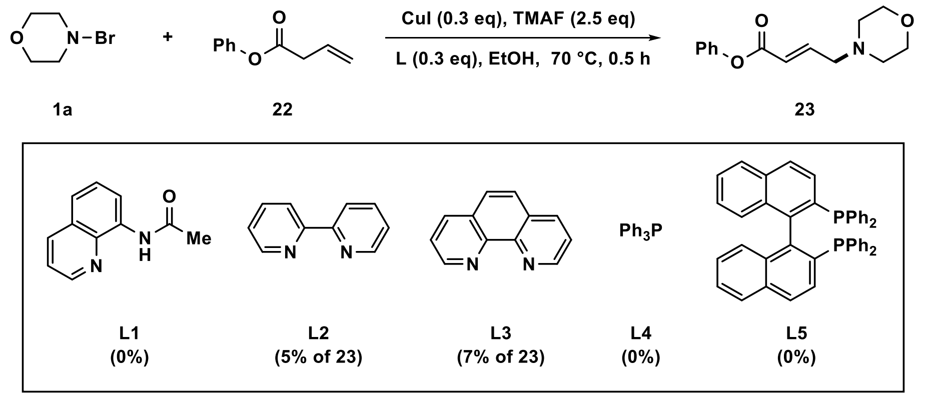

Standard conditions A: **1a** (0.40 mmol) dissolved in EtOH (2.0 mL) was added by syringe pump into the mixture of **22** (0.20 mmol), extra **L** (0.06 mmol), CuI (0.06 mmol), and TMAF (0.50 mmol) in EtOH (3.0 mL) at 70 °C.

Compound **23** was obtained as yellow oil. **^{1}H NMR** (500 MHz, $CDCl_3$) δ 7.38 (t, J = 8.0 Hz, 2H), 7.24 (t, J = 7.5 Hz, 1H), 7.19 – 7.06 (m, 3H), 6.21 (d, J = 15.5 Hz, 1H), 3.74 (t, J = 5.0 Hz, 4H), 3.21 (d, J = 6.0 Hz, 2H), 2.54 – 2.46 (m, 4H). **^{13}C NMR** (150 MHz, $CDCl_3$) δ 164.5, 150.7, 147.0, 129.5, 125.9, 122.8, 121.6, 67.0, 59.7, 53.9. **IR** ν_{max} (film): 2947, 2851, 2360, 2341, 1649, 1537, 1490, 1433, 1378, 841, 746 cm^{-1}. **HRMS** (ESI) m/z calcd for $C_{14}H_{18}NO_3$ $[M+H]^+$: 248.1281; found: 248.1287.

Table S2. Test of the asymmetric version of this reaction.

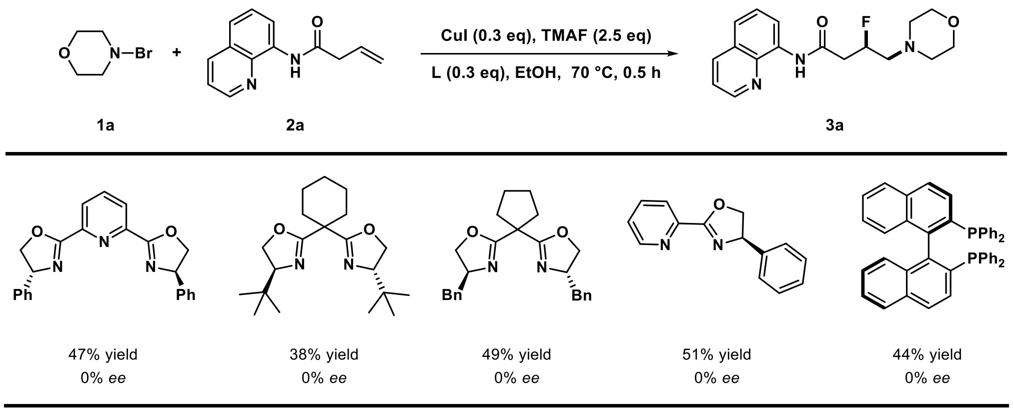

Standard conditions A: **1a** (0.40 mmol) dissolved in EtOH (2.0 mL) was added by syringe pump into the mixture of

2a (0.20 mmol), chair **L** (0.06 mmol), CuI (0.06 mmol), and TMAF (0.50 mmol) in EtOH (3.0 mL) at 70 °C.

HPLC for compound *rac*-**3a** (AD, *n*-hexane/*i*-PrOH = 80/20, flow rate = 0.8 mL/min, l = 254 nm) t_R = 8.2 min, 12.9 min.

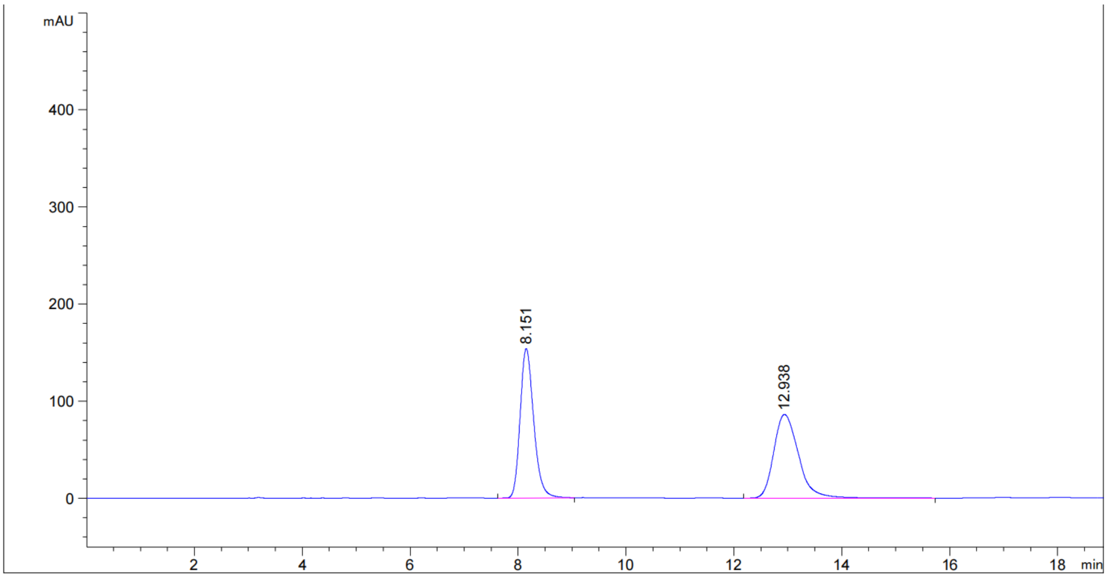

峰<br>#	保留时间<br>[min]	类型	峰宽<br>[min]	峰面积<br>[mAU*s]	峰高<br>[mAU]	峰面积<br>%
1	8.151	BB	0.2688	2689.31665	153.96535	49.5263
2	12.938	BB	0.4970	2740.75586	86.17735	50.4737

Crystallography data

Compound **3p** (CCDC 2054186)

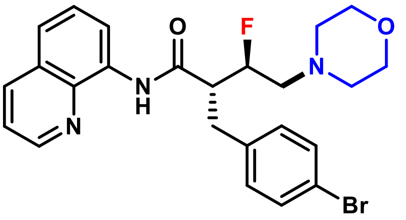

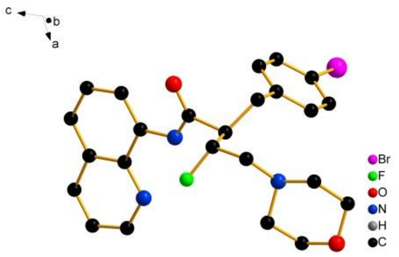

Crystal data

$C_{24}H_{25}BrFN_3O_2$	F(000) = 500
M_r = 486.38	D_x = 1.386 Mg m^{-3}
Monoclinic, $P2_1$	Cu $K\alpha$ radiation, λ = 1.54178 Å
a = 11.6736 (7) Å	Cell parameters from 9846 reflections
b = 9.4847 (6) Å	θ = 2.7–63.6°
c = 12.0145 (7) Å	μ = 2.68 mm^{-1}
β = 118.806 (3)°	T = 293 K
V = 1165.64 (13) Å^3	Block, colorless
Z = 2	0.05 × 0.03 × 0.03 mm

Data collection

CCD area detector diffractometer	R_{int} = 0.030
graphite	θ_{max} = 63.6°, θ_{min} = 4.2°
phi and ω scans	h = -13→13
9130 measured reflections	k = -10→11
3764 independent reflections	l = -13→13
3161 reflections with $I > 2\sigma(I)$	

Refinement

Refinement on F^2	Hydrogen site location: inferred from neighbouring sites
Least-squares matrix: full	H-atom parameters constrained
$R[F^2 > 2\sigma(F^2)]$ = 0.037	$w = 1/[\sigma^2(F_o^2) + (0.038P)^2 + 0.218P]$ where $P = (F_o^2 + 2F_c^2)/3$
$wR(F^2)$ = 0.094	$(\Delta/\sigma)_{max}$ < 0.001
S = 1.05	$\Delta\rangle_{max}$ = 0.26 e Å^{-3}
3764 reflections	$\Delta\rangle_{min}$ = -0.44 e Å^{-3}
280 parameters	Absolute structure: Flack x determined using 1282 quotients [(I+)-(I-)]/[(I+)+(I-)] (Parsons and Flack (2004), Acta Cryst. A60, s61).

1 restraint	Flack parameter: 0.000 (10)

Special details

Geometry. All esds (except the esd in the dihedral angle between two l.s. planes) are estimated using the full covariance matrix. The cell esds are taken into account individually in the estimation of esds in distances, angles and torsion angles; correlations between esds in cell parameters are only used when they are defined by crystal symmetry. An approximate (isotropic) treatment of cell esds is used for estimating esds involving l.s. planes.

Fractional atomic coordinates and isotropic or equivalent isotropic displacement parameters (Å^2)

	x	*y*	*z*	U_{iso}*/U_{eq}
Br1	-0.93555 (7)	-0.99487 (11)	-0.92372 (7)	0.1218 (4)
F1	-0.6069 (3)	-0.4337 (3)	-0.2258 (2)	0.0728 (8)
O1	-0.8734 (3)	-0.6723 (4)	-0.3305 (4)	0.0742 (10)
O2	-0.5014 (4)	-0.1810 (3)	-0.5753 (4)	0.0691 (9)
N1	-0.4459 (4)	-0.8892 (4)	-0.1714 (3)	0.0565 (9)
N2	-0.6812 (3)	-0.7649 (3)	-0.3017 (3)	0.0450 (8)
H2	-0.6199	-0.7537	-0.3211	0.054*
N3	-0.6650 (3)	-0.2813 (3)	-0.4768 (3)	0.0514 (9)
C1	-0.8824 (4)	-0.5262 (5)	-0.5453 (4)	0.0594 (11)
H1A	-0.9584	-0.5267	-0.5326	0.071*
H1B	-0.8798	-0.4361	-0.5821	0.071*
C2	-0.8957 (4)	-0.6422 (5)	-0.6362 (4)	0.0541 (11)
C3	-0.8509 (6)	-0.6220 (7)	-0.7216 (5)	0.0787 (16)
H3	-0.8124	-0.5365	-0.7226	0.094*
C4	-0.8620 (6)	-0.7264 (8)	-0.8059 (5)	0.0860 (18)
H4	-0.8313	-0.7108	-0.8634	0.103*
C5	-0.9181 (5)	-0.8531 (7)	-0.8056 (5)	0.0723 (15)
C6	-0.9599 (5)	-0.8781 (6)	-0.7189 (5)	0.0679 (13)
H6	-0.9951	-0.9652	-0.7163	0.082*

C7	-0.9491 (4)	-0.7729 (5)	-0.6355 (5)	0.0608 (12)
H7	-0.9784	-0.7897	-0.5771	0.073*
C8	-0.7769 (4)	-0.6658 (4)	-0.3451 (4)	0.0497 (10)
C9	-0.6721 (4)	-0.8847 (4)	-0.2281 (4)	0.0440 (9)
C10	-0.7749 (5)	-0.9382 (5)	-0.2161 (4)	0.0560 (11)
H10	-0.8569	-0.8961	-0.2592	0.067*
C11	-0.7566 (6)	-1.0559 (5)	-0.1390 (5)	0.0708 (14)
H11	-0.8276	-1.0925	-0.1334	0.085*
C12	-0.6379 (6)	-1.1178 (6)	-0.0724 (5)	0.0759 (15)
H12	-0.6275	-1.1949	-0.0206	0.091*
C13	-0.5324 (5)	-1.0656 (5)	-0.0820 (4)	0.0604 (12)
C14	-0.4038 (7)	-1.1211 (7)	-0.0135 (5)	0.0841 (18)
H14	-0.3888	-1.1996	0.0381	0.101*
C15	-0.3032 (6)	-1.0621 (7)	-0.0219 (5)	0.0828 (17)
H15	-0.2187	-1.0974	0.0244	0.099*
C16	-0.3299 (5)	-0.9463 (6)	-0.1024 (5)	0.0700 (14)
H16	-0.2600	-0.9062	-0.1078	0.084*
C17	-0.5468 (4)	-0.9479 (4)	-0.1600 (4)	0.0466 (10)
C18	-0.7583 (4)	-0.5426 (4)	-0.4160 (4)	0.0466 (9)
H18	-0.6839	-0.5623	-0.4301	0.056*
C19	-0.7293 (5)	-0.4116 (4)	-0.3343 (5)	0.0579 (12)
H19	-0.7955	-0.4043	-0.3067	0.069*
C20	-0.7276 (5)	-0.2737 (4)	-0.3973 (5)	0.0593 (12)
H20A	-0.8170	-0.2419	-0.4491	0.071*
H20B	-0.6825	-0.2035	-0.3318	0.071*
C21	-0.7008 (5)	-0.1603 (5)	-0.5618 (5)	0.0661 (13)
H21A	-0.6719	-0.0743	-0.5122	0.079*
H21B	-0.7951	-0.1561	-0.6134	0.079*
C22	-0.6396 (5)	-0.1707 (6)	-0.6467 (6)	0.0739 (14)
H22A	-0.6738	-0.2530	-0.7008	0.089*
H22B	-0.6629	-0.0881	-0.7009	0.089*
C23	-0.4671 (5)	-0.2992 (5)	-0.4927 (5)	0.0650 (13)

H23A	-0.3726	-0.3057	-0.4438	0.078*
H23B	-0.4990	-0.3845	-0.5429	0.078*
C24	-0.5228 (5)	-0.2888 (5)	-0.4034 (5)	0.0619 (12)
H24A	-0.4972	-0.3707	-0.3481	0.074*
H24B	-0.4893	-0.2053	-0.3508	0.074*

Atomic displacement parameters (Å^2)

	U^{11}	U^{22}	U^{33}	U^{12}	U^{13}	U^{23}
Br1	0.0938 (5)	0.1773 (8)	0.0856 (5)	0.0014 (5)	0.0362 (4)	-0.0506 (5)
F1	0.088 (2)	0.0691 (17)	0.0578 (16)	-0.0122 (14)	0.0323 (15)	-0.0015 (13)
O1	0.069 (2)	0.065 (2)	0.115 (3)	0.0123 (17)	0.066 (2)	0.025 (2)
O2	0.068 (2)	0.063 (2)	0.088 (3)	-0.0091 (16)	0.046 (2)	0.0095 (18)
N1	0.058 (2)	0.057 (2)	0.057 (2)	0.0143 (18)	0.0293 (18)	0.0037 (18)
N2	0.0473 (19)	0.0399 (18)	0.052 (2)	0.0016 (15)	0.0274 (17)	0.0034 (15)
N3	0.056 (2)	0.0391 (19)	0.067 (2)	-0.0021 (16)	0.0359 (19)	0.0016 (17)
C1	0.048 (2)	0.049 (3)	0.073 (3)	0.008 (2)	0.023 (2)	0.015 (2)
C2	0.040 (2)	0.060 (3)	0.051 (3)	0.0017 (19)	0.013 (2)	0.013 (2)
C3	0.081 (4)	0.091 (4)	0.065 (3)	-0.020 (3)	0.036 (3)	0.014 (3)
C4	0.081 (4)	0.127 (5)	0.055 (3)	-0.015 (4)	0.037 (3)	0.011 (3)
C5	0.051 (3)	0.105 (4)	0.054 (3)	-0.002 (3)	0.020 (2)	-0.006 (3)
C6	0.057 (3)	0.070 (3)	0.078 (3)	-0.008 (2)	0.034 (3)	-0.007 (3)
C7	0.056 (3)	0.061 (3)	0.071 (3)	-0.008 (2)	0.035 (2)	0.003 (3)
C8	0.052 (2)	0.043 (2)	0.063 (3)	0.006 (2)	0.034 (2)	0.008 (2)
C9	0.057 (3)	0.037 (2)	0.037 (2)	-0.0013 (19)	0.0217 (19)	-0.0037 (17)
C10	0.056 (2)	0.055 (3)	0.054 (2)	-0.008 (2)	0.024 (2)	-0.002 (2)
C11	0.087 (4)	0.064 (3)	0.068 (3)	-0.016 (3)	0.043 (3)	0.009 (3)
C12	0.104 (4)	0.068 (3)	0.059 (3)	0.002 (3)	0.042 (3)	0.022 (3)
C13	0.082 (3)	0.057 (3)	0.043 (2)	0.015 (2)	0.031 (2)	0.012 (2)
C14	0.106 (5)	0.092 (4)	0.059 (3)	0.043 (4)	0.043 (3)	0.031 (3)
C15	0.085 (4)	0.103 (4)	0.060 (3)	0.044 (3)	0.035 (3)	0.021 (3)
C16	0.063 (3)	0.081 (3)	0.071 (3)	0.020 (3)	0.036 (3)	0.004 (3)

C17	0.063 (3)	0.041 (2)	0.036 (2)	0.0069 (19)	0.0238 (18)	-0.0036 (18)
C18	0.046 (2)	0.041 (2)	0.058 (2)	0.0054 (17)	0.0293 (19)	0.0064 (19)
C19	0.072 (3)	0.045 (2)	0.079 (3)	-0.001 (2)	0.053 (3)	-0.003 (2)
C20	0.069 (3)	0.040 (2)	0.084 (3)	0.001 (2)	0.050 (3)	-0.002 (2)
C21	0.068 (3)	0.049 (2)	0.087 (4)	0.005 (2)	0.041 (3)	0.018 (3)
C22	0.074 (4)	0.071 (3)	0.081 (4)	-0.001 (3)	0.040 (3)	0.019 (3)
C23	0.060 (3)	0.059 (3)	0.083 (4)	0.000 (2)	0.040 (3)	0.008 (3)
C24	0.059 (3)	0.060 (3)	0.066 (3)	-0.003 (2)	0.029 (3)	0.004 (2)

Geometric parameters (Å, º)

Br1—C5	1.893 (6)	C4—C5	1.370 (9)
F1—C19	1.410 (6)	C5—C6	1.366 (7)
O1—C8	1.222 (5)	C6—C7	1.377 (7)
O2—C22	1.419 (7)	C8—C18	1.522 (6)
O2—C23	1.421 (6)	C9—C10	1.373 (6)
N1—C16	1.316 (6)	C9—C17	1.420 (6)
N1—C17	1.368 (6)	C10—C11	1.399 (6)
N2—C8	1.357 (5)	C11—C12	1.356 (8)
N2—C9	1.412 (5)	C12—C13	1.383 (8)
N3—C20	1.457 (6)	C13—C14	1.420 (8)
N3—C21	1.458 (6)	C13—C17	1.415 (6)
N3—C24	1.458 (6)	C14—C15	1.349 (9)
C1—C2	1.505 (7)	C15—C16	1.396 (7)
C1—C18	1.538 (6)	C18—C19	1.517 (6)
C2—C3	1.372 (7)	C19—C20	1.517 (6)
C2—C7	1.389 (6)	C21—C22	1.504 (8)
C3—C4	1.377 (9)	C23—C24	1.504 (7)
C22—O2—C23	109.4 (4)	C9—C10—C11	120.1 (5)
C16—N1—C17	116.4 (4)	C12—C11—C10	121.5 (5)
C8—N2—C9	126.7 (3)	C11—C12—C13	119.6 (5)

C20—N3—C21	110.8 (3)	C12—C13—C14	123.6 (5)
C20—N3—C24	112.9 (4)	C12—C13—C17	120.7 (5)
C21—N3—C24	108.7 (4)	C17—C13—C14	115.6 (5)
C2—C1—C18	112.1 (3)	C15—C14—C13	121.2 (5)
C3—C2—C1	119.8 (4)	C14—C15—C16	117.8 (5)
C3—C2—C7	117.6 (5)	N1—C16—C15	125.3 (5)
C7—C2—C1	122.5 (4)	N1—C17—C9	117.9 (3)
C2—C3—C4	121.0 (5)	N1—C17—C13	123.6 (4)
C5—C4—C3	120.3 (5)	C13—C17—C9	118.4 (4)
C4—C5—Br1	120.0 (4)	C8—C18—C1	108.9 (3)
C6—C5—Br1	119.9 (5)	C19—C18—C1	112.3 (3)
C6—C5—C4	120.1 (5)	C19—C18—C8	108.3 (3)
C5—C6—C7	119.2 (5)	F1—C19—C18	106.7 (3)
C6—C7—C2	121.7 (5)	F1—C19—C20	109.8 (4)
O1—C8—N2	123.7 (4)	C18—C19—C20	115.9 (4)
O1—C8—C18	120.6 (4)	N3—C20—C19	114.5 (3)
N2—C8—C18	115.8 (3)	N3—C21—C22	110.7 (4)
N2—C9—C17	116.5 (4)	O2—C22—C21	111.6 (5)
C10—C9—N2	123.9 (4)	O2—C23—C24	111.9 (4)
C10—C9—C17	119.6 (4)	N3—C24—C23	109.2 (4)

Part 3：DFT calculations

Computational Details

All DFT calculations were carried out with the Gaussian 09 package. Molecular geometries of all studied systems were optimized at (U)M06[6-31G(d)/LanL2DZ(Cu, I, Ag and Cs)] level.

Frequency calculations were performed to confirm all the stationary points as local minimum (zero imaginary frequencies) or transition states (one imaginary frequency). Intrinsic reaction coordinate (IRC) calculations were conducted to verify that each transition state actually connects the two corresponding minimum. In addition, The single-point energies of all stationary points were performed at the (U)M06[6-311++G(d,p)/SDD(Cu, I, Ag and Cs)] level. Both geometry optimizations and single-point calculations were considered the solvent effects with the SMD model in ethanol solvent. The redox potentials and Marcus electron transfer theory were employed to evaluate the single electron transfer (SET) process.

Redox Potential

We calculated the standard potential ($E_{1/2}^{red}$) versus the standard calomel electrode (E_{SCE}) according to the equation (S1):

$$E_{1/2}^{red} = -\frac{\Delta G_{O|R}}{nF} - E_{SCE} \tag{S1}$$

where F is the Faraday constant, n is the number of electrons transferred, E_{SCE} = 4.51 eV, and $\Delta G_{O|R}$ (eV) is the electronic and thermal free energy difference between oxidized and reduced states.

Table S1. The calculated redox potentials of Cu and **1a** complexes.

Redox Potential	$E_{1/2}^{red}[Cu^{II}/Cu^{I}]$	$E_{1/2}^{red}[S3^{+}/S3]$
**Cal.**	–0.04 V	1.12V

Scheme S1. Single electron transfer processes.

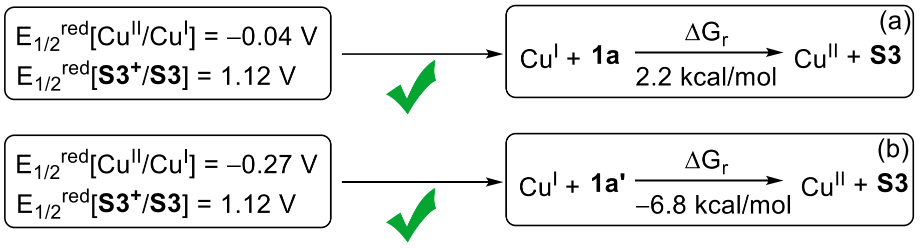

Activation Barrier of Single Electron Transfer Step

According to the Marcus equation, the reorganization energy λ is normally decomposed into internal energy (λ_i) and external energy (λ_o). The internal reorganization energy λ_i can be estimated according to the equation (S2):

$$\lambda_i = [E^D(Q_R) + E^A(Q_R)] - [E^D(Q_P) + E^A(Q_P)] \tag{S2}$$

where Q_R and Q_P are the equilibrium geometries of the reactants and products, respectively. In

addition, the external reorganization energy λ_o may be calculated from equation (S3−S5):

$$\lambda_o = (332\,\text{kcal/mol})\left(\frac{1}{2a_1} + \frac{1}{2a_2} - \frac{1}{R}\right)\left(\frac{1}{\varepsilon_{op}} - \frac{1}{\varepsilon}\right) \quad \text{(S3)}$$

$$\lambda = \lambda_o + \lambda_i \quad \text{(S4)}$$

$$\Delta G^{\circ\neq} = \frac{(\Delta G_r + \lambda)^2}{4\lambda} \quad \text{(S5)}$$

where a_1 is the radii of the oxidant, a_2 is the radii of the reductant, R = $a_1 + a_2$, ε_{op} is the optical dielectric constant (ε_{op} = 2.25), ε is the optical dielectric constant (ε = 3.7), and ΔG_r is the free energy change of the reaction.

Table S2. Estimation of the activation barriers for SET step.

**SET step**	**a$_1$** (Å)	**a$_2$** (Å)	**R** (Å)	**λ** (kcal/mol)	**ΔG$_r$** (kcal/mol)	**ΔG$_{SET}$** (kcal/mol)
(a)	3.22	2.60	5.82	46.3	2.2	12.7
(b)	3.22	2.56	5.78	44.7	−6.8	8.0

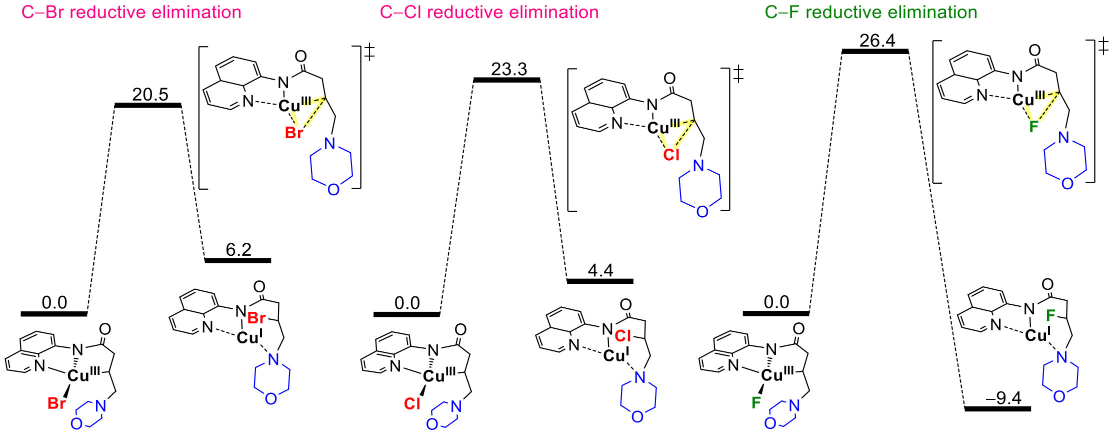

Figure S1. Gibbs energy profiles ($\Delta G^{\circ}_{343.15}$) of three C–X (X = Br, Cl, F) reductive eliminations at the M06/[6-311++G(d,p)/SDD(Cu)]//M06/[6-31G(d)/LanL2DZ (Cu)] level.

To evaluate the feasibility of this intermolecular alkylaminofluorination of alkenes, preliminary density functional theory (DFT) calculations were carried out for the challenging reductive elimination process. As shown in Figure S1, the Gibbs activation energy ($\Delta G^{\circ\ddagger}$) and Gibbs free energy change (ΔG°) of three C–X (X = F, Cl, Br) reductive eliminations were evaluated at the M06/[6-311++G(d,p)/SDD(Cu)]//M06/[6-31G(d)/LanL2DZ(Cu)] level. The preliminary DFT calculations show that the C–Br and C–Cl reductive eliminations seem to be feasible in kinetics,

but they are endothermic in thermodynamics. In contrast, the C–F reductive elimination becomes unfavorable in kinetics, but it possess a potential driving force in thermodynamics due to the strong C–F bond.

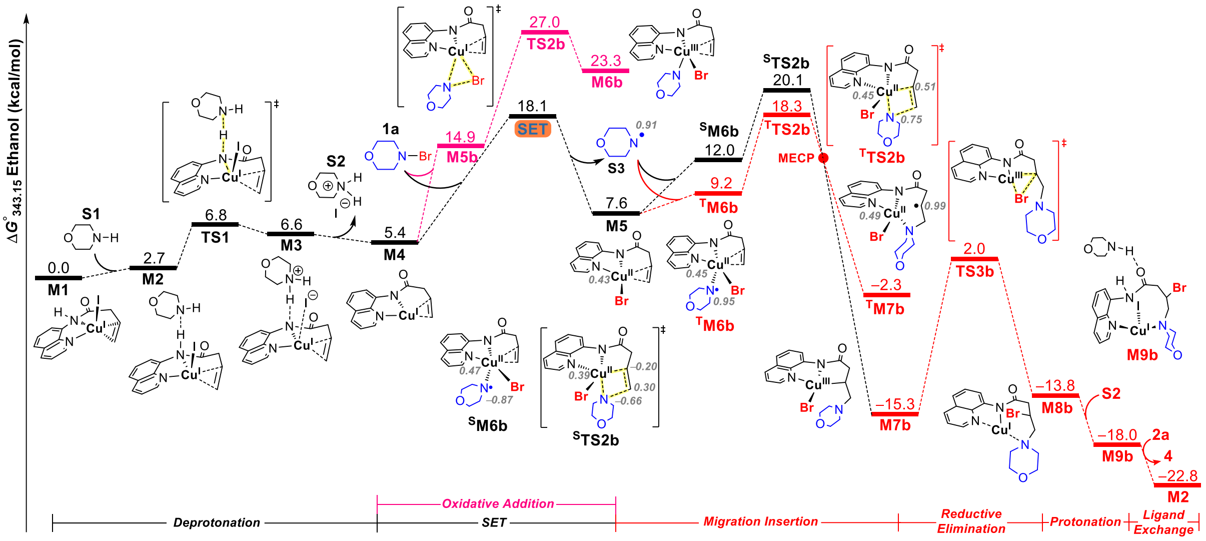

Figure S2. Gibbs energy profiles ($\Delta G°_{343.15}$) without nucleophilic fluoride TMAF calculated at the SMD(ethanol)/(U)M06/[6-311++G(d,p)/SDD(Cu,I)]//SMD(ethanol)/(U)M06/[6-31G(d)/LanL2DZ(Cu,I)] level. Spin densities are given in grey italic font.

As shown in Figure S2, in the absence of TMAF, the most favorable catalytic cycle consists of six key elementary steps: deprotonation, SET, migratory insertion, reductive elimination, protonation, and ligand exchange, as depicted with black and red lines. First, a deprotonation process of **M1** by **S1** occurs via **TS1** to afford **M3** followed by **S2** dissociation into an active Cu(I) amino intermediate **M4** with the $\Delta G^{°‡}$ value of 6.8 kcal/mol. In comparison with the unfavorable oxidative addition of **1a** to **M4** (**M5b** → **TS2b** → **M6b** as depicted with pink line), the Cu(II) bromide complex **M5** and aminyl radical **S3** are obtained through a SET process. Then, **M5** is coordinated preferentially with **S3** followed by olefin insertion into the Cu(II)···N• bond and reductive elimination to afford the β-bromoamine **4**. The C–Br reductive elimination is the rate-determining step of the catalytic cycle with a $\Delta G^{°‡}$ value of 17.3 kcal/mol. The total $\Delta G°$ value of this cycle is –25.5 kcal/mol.

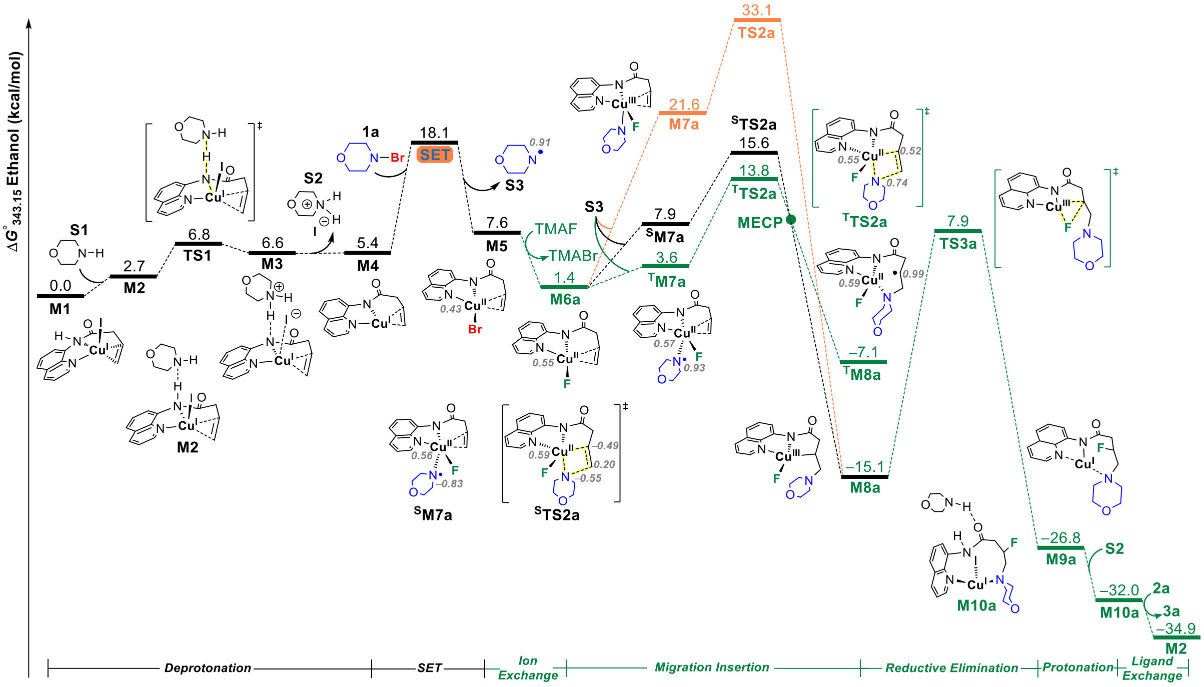

Figure S3. Gibbs energy profiles ($\Delta G^{\circ}_{343.15}$) with nucleophilic fluoride TMAF calculated at the SMD(ethanol)/(U)M06/[6-311++G(d,p)/SDD(Cu,I)]//SMD(ethanol)/(U)M06/[6-31G(d)/LanL2DZ(Cu,I)] level. Spin densities are given in grey italic font.

As shown in Figure S3, with the nucleophilic fluoride of TMAF, the most favorable catalytic cycle consists of seven key elementary steps: deprotonation, SET, ion exchange, migratory insertion, reductive elimination, protonation, and ligand exchange, as depicted with black and green lines. Starting from **M5**, the bromine-fluoride ion exchange occurs exothermically between **M5** and TMAF to generate a doublet Cu(II) fluoride **M6a** with a Δ*G*° value of –6.2 kcal/mol. Next, **M6a** could interact with **S3** to generate three possible intermediates, a closed-shell singlet Cu(III) fluoride **M7a**, an antiferromagnetic singlet Cu(II) fluoride ^{S}**M7a** and a triplet Cu(II) fluoride ^{T}**M7a**. ^{T}**M7a** is more stable than **M7a** and ^{S}**M7a** by 18.0 and 4.3 kcal/mol, respectively. The most favorable pathway is the migratory insertion of olefin moiety of ^{T}**M7a** into the Cu(II)···N• bond through ^{T}**TS2a** and minimum energy crossing point (MECP) can form a relative stable singlet amino Cu(III) fluoride **M8a** with a moderate $\Delta G^{\circ\ddagger}$ value of 12.4 kcal/mol relative to **M6a**. Finally, the reductive elimination, protonation and ligand exchange processes can release the desired β-fluoroamine **3a** and regenerate **M2** to restart the catalytic cycle. Overall, the C–F reductive elimination is the rate-determining step of the catalytic cycle with a $\Delta G^{\circ\ddagger}$ value of 23.0 kcal/mol. The total Δ*G*° value of this cycle is –37.6 kcal/mol.

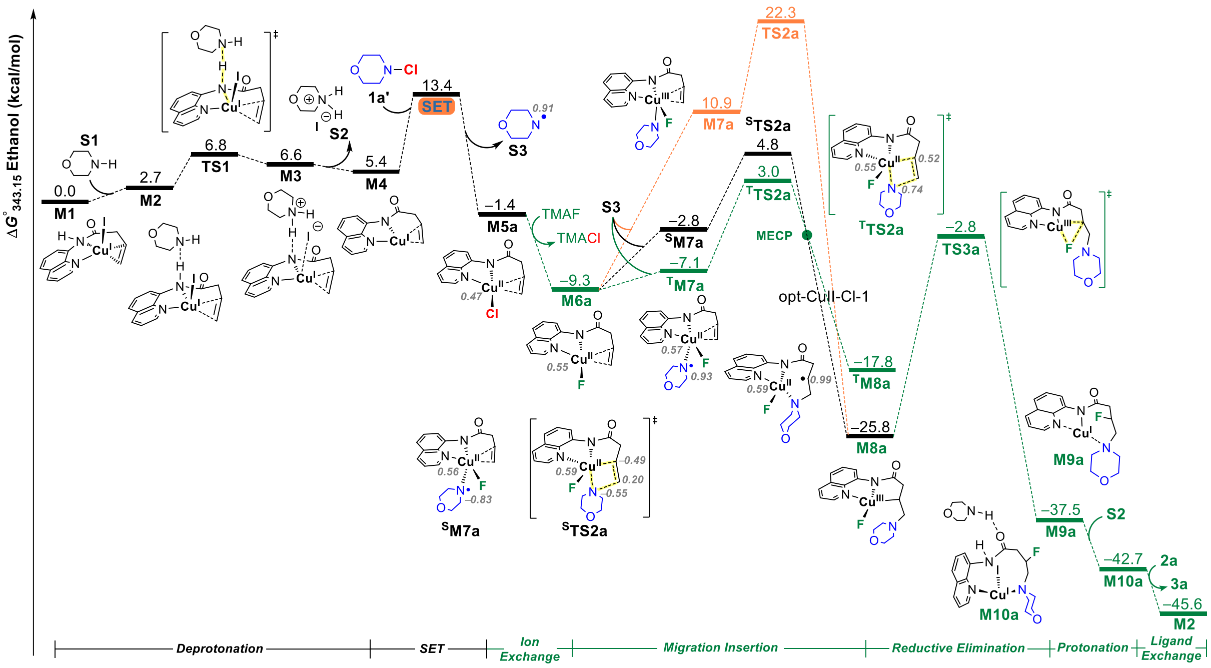

Figure S4. Gibbs energy profiles ($\Delta G°_{343.15}$) with 4-chloromorpholine **1a′** as amino source and TMAF as nucleophilic fluoride source calculated at the SMD(ethanol)/(U)M06/[6-311++G(d,p)/SDD(Cu,I)]//SMD(ethanol)/(U)M06/[6-31G(d)/LanL2DZ (Cu,I)] level. Spin densities are given in grey italic font.

As shown in Figure S4, the catalytic cycle with 4-chloromorpholine **1a′** as amino source in the presence of TMAF has been evaluated to deliver the desired aminofluorination product **3a**. This catalytic cycle is similar to the one with 4-bromomorpholine **1a** as amino source (Figure S3). Overall, the C–F reductive elimination is still the rate-determining step with a $\Delta G°^{‡}$ value of 23.0 kcal/mol. The total $\Delta G°$ value of this cycle is –48.3 kcal/mol.

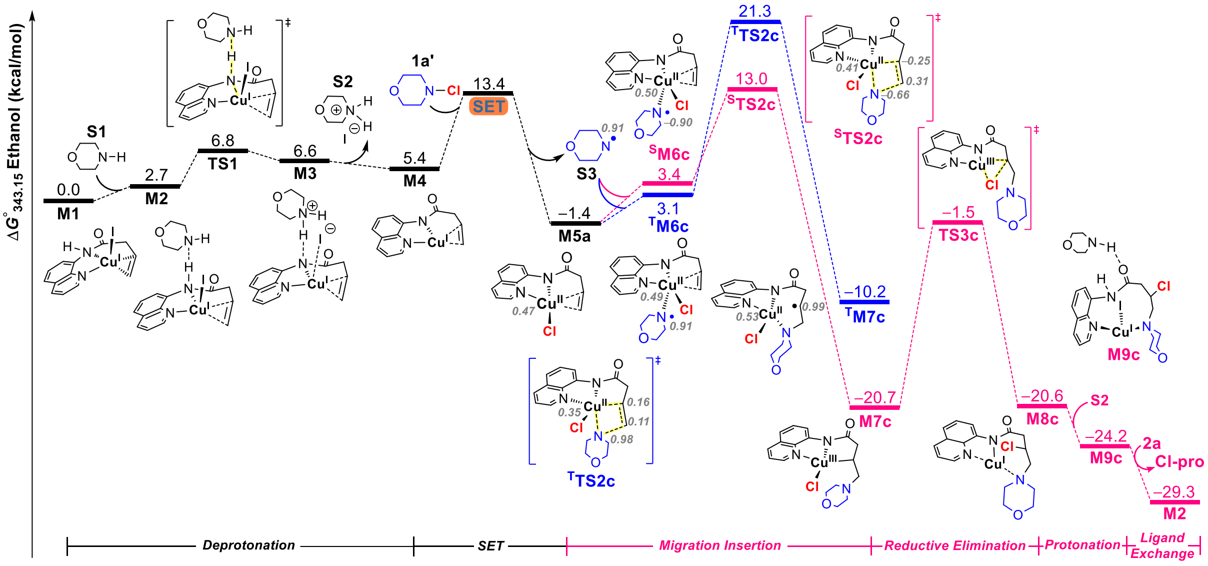

Figure S5. Gibbs energy profiles ($\Delta G°_{343.15}$) with 4-chloromorpholine **1a′** as amino source and without nucleophilic fluoride TMAF calculated at the SMD(ethanol)/(U)M06/[6-311++G(d,p)/SDD(Cu,I)]//SMD(ethanol)/(U)M06/[6-31G(d)/LanL2DZ(Cu,I)] level. Spin densities are given in grey italic font.

As shown in Figure S5, the catalytic cycle with 4-chloromorpholine **1a′** as amino source and without TMAF has also been evaluated to deliver the β-chloramine **Cl-pro**. This catalytic cycle is similar to the one with 4-bromomorpholine **1a** as amino source in the absence of TMAF (Figure S2). Overall, the C–Cl reductive elimination is the rate-determining step with a $\Delta G°^{‡}$ value of 19.2 kcal/mol. The total $\Delta G°$ value of this cycle is –32.0 kcal/mol.

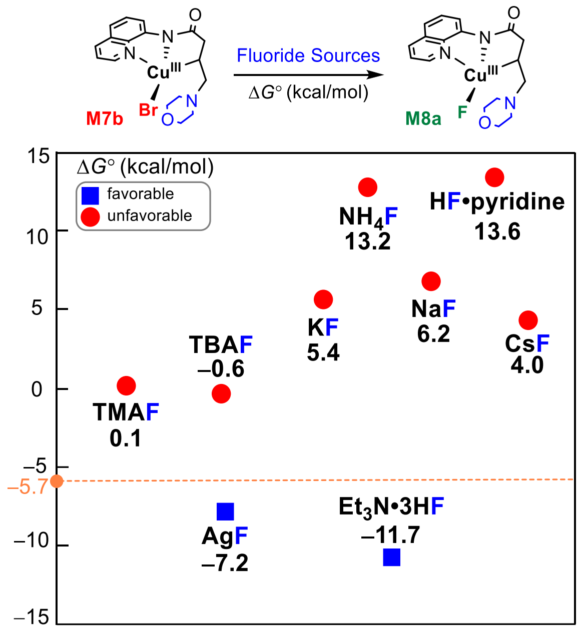

Figure S6. Gibbs energy ($\Delta G°_{343.15}$) of ion exchange between nucleophilic fluoride sources and Cu(III) bromide intermediate **M7b** calculated at the SMD(ethanol)/(U)M06/[6-311++G(d,p)/SDD(Cu,I,Ag,Cs)]//(U)M06/[6-31G(d)/LanL2DZ(Cu,I,Ag,Cs)] level.

In principle, the external nucleophilic fluoride could undergo ion exchange with either Cu(II) intermediate or Cu(III) one. To identify which route is preferable, a series of nucleophilic fluoride sources were tested to evaluate the $\Delta G°$ values of ion exchange with Cu(II) bromide **M5** (Figure 4) and Cu(III) bromide **M7b** (Figure S6). By comparison, the DFT calculation results show that **M5** has more thermodynamic advantages than **M7b** to exchange ion with external fluoride source. Overall, TMAF, TBAF and Et_3N•3HF can effectively trigger this aminofluorination reaction. These DFT calculations align with the experimental results shown in Scheme 1. Therefore, enough exothermic ion exchange between external fluoride source and the Cu(II) bromide provides more driving force to the irreversible migratory insertion, thus promotes the kinetically challenging C(sp^3)–F reductive elimination.

Cartesian Coordinates of Optimized Structures

M1

(U)M06/BSII SCF energy in solution: -895.950379 a.u.

(U)M06/BSI SCF energy in solution: -894.547675 a.u.

(U)M06/BSI SCF Gibbs energy in solution: -894.366953 a.u.

C	1.57336300	-2.34842600	0.10250300
C	1.38570200	-0.03407600	-0.00835500
C	2.79763500	0.11550900	0.00727900
C	3.58258100	-1.05865300	0.06245800
C	2.97746200	-2.28859300	0.10908100
H	1.06431500	-3.31037600	0.14665400
C	0.53105300	1.12776100	-0.07535100
C	3.36564100	1.41117100	-0.03434200
H	4.66856200	-0.96626900	0.06821800
H	3.55553600	-3.20813100	0.15157900
C	2.54101700	2.50777800	-0.08623400
C	1.13852100	2.37637800	-0.10690900
H	4.45001500	1.51372900	-0.02454000
H	2.97186300	3.50781000	-0.11839900
H	0.51090100	3.26127200	-0.15091700
N	0.80426100	-1.27550400	0.04601900

N	-0.81112700	0.83966500	-0.11088000
C	-1.83688800	1.67959800	0.09286900
C	-3.16894600	0.91944800	0.20049900
H	-3.39157800	0.81889500	1.27499200
H	-3.96564300	1.54231900	-0.23013700
O	-1.81179500	2.90862100	0.26080600
C	-3.16107200	-0.43668000	-0.45064600
H	-3.15690800	-0.43103400	-1.54563500
C	-3.29648600	-1.64379300	0.18276100
H	-3.44454700	-1.69810000	1.26362800
H	-3.45400700	-2.56029700	-0.38491500
Cu	-1.23598600	-1.12594600	-0.07091800

S1

(U)M06/BSII SCF energy in solution: -287.50473 a.u.

(U)M06/BSI SCF energy in solution: -287.611873 a.u.

(U)M06/BSI SCF Gibbs energy in solution: -287.50473 a.u.

N	-0.00456400	1.36303600	-0.33451800
O	0.00462500	-1.38863300	0.29404800
C	-1.19401600	0.70682500	0.19829800
C	1.18922600	0.71470300	0.19826000
C	-1.16467300	-0.75235200	-0.19774900
C	1.16973300	-0.74469000	-0.19770100
H	-2.09472000	1.18386600	-0.21003200
H	-1.25107200	0.76820200	1.30382200
H	1.24590300	0.77651200	1.30377600
H	2.08676400	1.19766700	-0.21008200
H	-2.02076900	-1.29341900	0.22285500
H	-1.20066400	-0.84046800	-1.29921500
H	1.20633900	-0.83265000	-1.29916300
H	2.02937400	-1.28004700	0.22299700
H	-0.00783400	2.34123800	-0.05236700

M2

(U)M06/BSII SCF energy in solution: -1183.663705 a.u.

(U)M06/BSI SCF energy in solution: -1182.176814 a.u.

(U)M06/BSI SCF Gibbs energy in solution: -1181.870034 a.u.

O	1.73463100	-0.36791600	3.40023400
N	1.23281500	0.48307800	1.34046400
C	-0.40994700	0.66387200	3.08798800
C	-1.39264100	-0.43969600	2.78950700
C	0.97401600	0.24240800	2.65510400
C	-1.06875400	-1.73578400	2.57093800
H	0.56637900	1.04626800	0.76878100
H	-0.40607500	0.87127600	4.16704700
H	-2.44598000	-0.15607000	2.82587700
H	-1.85027900	-2.48568800	2.44764000
H	-0.04157100	-2.09921200	2.63704800
H	-0.73067600	1.58393500	2.57910000
Cu	-1.07541700	-0.88553300	0.52737800
I	-3.28202900	-0.18058600	-0.72150400
C	0.30152800	-2.33923700	-1.53832000
C	1.90063400	-1.15378000	-0.35223000
C	2.94601000	-1.77828500	-1.09193800
C	2.59109300	-2.71100900	-2.09189900
C	1.26995100	-2.98784000	-2.32369800
H	-0.75681900	-2.55221100	-1.69570900
C	2.25627800	-0.18787600	0.63530700
C	4.29652800	-1.46494300	-0.81269300
H	3.38100500	-3.19547300	-2.66502800
H	0.95601800	-3.69392100	-3.08767400
C	4.60809000	-0.55435700	0.16326200
C	3.58150400	0.09152500	0.87942100
H	5.07658500	-1.96286100	-1.38732100
H	5.64607300	-0.31271700	0.38213700
H	3.82491700	0.83592000	1.63572500
N	0.58742700	-1.46613700	-0.58542400
N	-0.33477300	2.24186900	-0.37599100
O	1.78681500	3.72049200	-1.51126900
C	-0.00144700	3.58190900	0.10922400

C	-0.00518000	2.12150800	-1.79713200
C	1.46965600	3.83870300	-0.13495200
C	1.46354500	2.42766700	-1.99630900
H	-0.21710300	3.64709800	1.18449700
H	-0.59208400	4.36247400	-0.40571200
H	-0.60315700	2.82047300	-2.41203400
H	-0.22537100	1.09994800	-2.13737100
H	1.74420900	4.85597900	0.16838400
H	2.07794000	3.12559000	0.45358800
H	2.08359400	1.66841300	-1.48011000
H	1.72292600	2.41451400	-3.06149900
H	-1.33094300	2.05679700	-0.24619300

TS1

(U)M06/BSII SCF energy in solution: -1183.654243 a.u.

(U)M06/BSI SCF energy in solution: -1182.16676 a.u.

(U)M06/BSI SCF Gibbs energy in solution: -1181.862804 a.u.

O	1.48107500	0.18652200	3.41610300
N	0.84820200	0.45446500	1.20658800
C	-0.73652600	0.95777700	2.92957500
C	-1.68887600	-0.19511700	2.72019400
C	0.67418700	0.53938700	2.54949800
C	-1.33660700	-1.50546200	2.76057400
H	0.30191200	1.36123500	0.33880000
H	-0.75554200	1.27346900	3.98250000
H	-2.74565700	0.06331900	2.62700000
H	-2.09547000	-2.28469100	2.69752500
H	-0.31878200	-1.82697000	2.99182500
H	-1.07580400	1.81251100	2.32678400
Cu	-1.05320100	-0.86629100	0.67397600
I	-3.06442500	-0.08438900	-0.93712900
C	0.26785900	-2.77153000	-1.21048400
C	1.75358500	-1.31828900	-0.17985400
C	2.85839800	-1.94007600	-0.82435000
C	2.59110500	-3.01342200	-1.70520400

C	1.29998300	-3.42675700	-1.90629900
H	-0.76818200	-3.08916900	-1.33616000
C	1.97600800	-0.20821600	0.69406900
C	4.17007000	-1.47917800	-0.56587600
H	3.42378600	-3.49969700	-2.21303200
H	1.05987900	-4.24755300	-2.57714600
C	4.36874600	-0.43838100	0.30627400
C	3.27556100	0.19741100	0.92920200
H	5.00635100	-1.96713500	-1.06535500
H	5.37703000	-0.08429100	0.51404500
H	3.44902300	1.03713900	1.60027800
N	0.47645500	-1.76635100	-0.38061600
N	-0.16642600	2.17143700	-0.46076600
O	1.98594000	3.52553100	-1.70448600
C	0.19756800	3.55496700	-0.08313400
C	0.23683800	1.86492900	-1.85382100
C	1.67117900	3.76833300	-0.34581500
C	1.70265700	2.18104900	-2.04565600
H	-0.03369800	3.70085200	0.97872700
H	-0.40112700	4.25848000	-0.67775000
H	-0.37595100	2.47069900	-2.53649300
H	0.03493600	0.80469000	-2.05081900
H	1.94668800	4.80588000	-0.12790600
H	2.27181100	3.10571000	0.30433600
H	2.32747200	1.50002100	-1.43778900
H	1.97667300	2.04725600	-3.09778200
H	-1.18212100	2.03544300	-0.38181600

M3

(U)M06/BSII SCF energy in solution: -1183.658649 a.u.

(U)M06/BSI SCF energy in solution: -1182.171401 a.u.

(U)M06/BSI SCF Gibbs energy in solution: -1181.863358 a.u.

O	1.50798600	0.65014300	3.44346300
N	0.76263800	0.37544800	1.25728400
C	-0.78316100	1.14046800	2.89147200

C	-1.74067400	-0.01675200	2.74797300
C	0.64609300	0.72751500	2.55552200
C	-1.40848400	-1.32452000	2.88254400
H	0.15593400	1.47956800	0.00442300
H	-0.82355000	1.53906100	3.91563300
H	-2.79460100	0.24245100	2.62353100
H	-2.17543900	-2.09782700	2.86590200
H	-0.39404500	-1.64277600	3.13333800
H	-1.10541900	1.94876400	2.21588500
Cu	-1.05056300	-0.76554100	0.73530600
I	-3.04845000	-0.10995300	-0.95573200
C	0.22385400	-2.85873800	-1.09444100
C	1.70358000	-1.38543000	-0.08402400
C	2.81325000	-2.01209800	-0.71426600
C	2.55027600	-3.10035300	-1.57857800
C	1.26095900	-3.52224200	-1.77565100
H	-0.81097000	-3.18134200	-1.21834600
C	1.90625500	-0.25993400	0.78441800
C	4.12197500	-1.54190200	-0.45702200
H	3.38537900	-3.59200100	-2.07758500
H	1.02650200	-4.35540000	-2.43349000
C	4.30527300	-0.48762400	0.40353200
C	3.20981800	0.15174800	1.01795800
H	4.96451400	-2.03108100	-0.94469800
H	5.31071700	-0.12337000	0.61030000
H	3.38484500	0.99973100	1.67635200
N	0.42859000	-1.83975900	-0.28176900
N	-0.17130000	2.13594000	-0.76454500
O	2.16981800	3.33968400	-1.78596500
C	0.19878300	3.53698500	-0.41529300
C	0.40633900	1.71083200	-2.07379300
C	1.70132500	3.67277600	-0.49335200
C	1.89316700	1.98408900	-2.08234000
H	-0.17519700	3.74673000	0.59211800
H	-0.29453300	4.20057400	-1.13428100

H	-0.10676700	2.28314400	-2.85518000
H	0.19229200	0.64329600	-2.20532300
H	1.99240800	4.70916100	-0.29362600
H	2.17985000	3.02402000	0.26321600
H	2.40793400	1.32772400	-1.35719800
H	2.29642800	1.77729100	-3.07907800
H	-1.19580000	2.02152900	-0.79668900

S2

(U)M06/BSII SCF energy in solution: -299.74337 a.u.

(U)M06/BSI SCF energy in solution: -299.6228 a.u.

(U)M06/BSI SCF Gibbs energy in solution: -299.508611 a.u.

H	-1.53944200	-0.00003500	1.76257200
I	2.23409700	0.00000000	-0.06456400
N	-1.23037200	-0.00002700	0.78140400
O	-3.62523300	0.00002400	-0.71685500
C	-1.71758000	-1.23433000	0.09269300
C	-1.71753800	1.23430300	0.09272200
C	-3.22188900	-1.16565600	-0.02489700
C	-3.22185100	1.16568500	-0.02489500
H	-1.38609800	-2.10157300	0.67090900
H	-1.23954000	-1.24646400	-0.89369600
H	-1.23948100	1.24646400	-0.89365900
H	-1.38605500	2.10152500	0.67097100
H	-3.58653300	-2.02809900	-0.59202200
H	-3.68400200	-1.19110100	0.97836300
H	-3.68397100	1.19115300	0.97836300
H	-3.58645000	2.02814600	-0.59202100
H	-0.18797500	-0.00004200	0.78340500

M4

(U)M06/BSII SCF energy in solution: -883.893549 a.u.

(U)M06/BSI SCF energy in solution: -882.525294 a.u.

(U)M06/BSI SCF Gibbs energy in solution: -882.355078 a.u.

C	1.57336300	-2.34842600	0.10250300

C	1.38570200	-0.03407600	-0.00835500
C	2.79763500	0.11550900	0.00727900
C	3.58258100	-1.05865300	0.06245800
C	2.97746200	-2.28859300	0.10908100
H	1.06431500	-3.31037600	0.14665400
C	0.53105300	1.12776100	-0.07535100
C	3.36564100	1.41117100	-0.03434200
H	4.66856200	-0.96626900	0.06821800
H	3.55553600	-3.20813100	0.15157900
C	2.54101700	2.50777800	-0.08623400
C	1.13852100	2.37637800	-0.10690900
H	4.45001500	1.51372900	-0.02454000
H	2.97186300	3.50781000	-0.11839900
H	0.51090100	3.26127200	-0.15091700
N	0.80426100	-1.27550400	0.04601900
N	-0.81112700	0.83966500	-0.11088000
C	-1.83688800	1.67959800	0.09286900
C	-3.16894600	0.91944800	0.20049900
H	-3.39157800	0.81889500	1.27499200
H	-3.96564300	1.54231900	-0.23013700
O	-1.81179500	2.90862100	0.26080600
C	-3.16107200	-0.43668000	-0.45064600
H	-3.15690800	-0.43103400	-1.54563500
C	-3.29648600	-1.64379300	0.18276100
H	-3.44454700	-1.69810000	1.26362800
H	-3.45400700	-2.56029700	-0.38491500
Cu	-1.23598600	-1.12594600	-0.07091800

M5

(U)M06/BSII SCF energy in solution: -3457.940631 a.u.

(U)M06/BSI SCF energy in solution: -3454.100764 a.u.

(U)M06/BSI SCF Gibbs energy in solution: -3453.931999 a.u.

O	0.99151200	3.63915800	-0.49318000
N	0.41316500	1.45539800	-0.02104200
C	-1.31522800	3.15912300	-0.25712000

C	-2.30591400	2.06871600	-0.01003700
C	0.15577600	2.76096100	-0.26368900
C	-2.50122200	1.51265300	1.20843900
H	-1.41507700	3.93615700	0.51480900
H	-2.94088200	1.75443200	-0.84235500
H	-3.28696600	0.78104200	1.38210400
H	-1.92224700	1.84220300	2.07420600
C	0.56465300	-2.49955600	-0.04470300
C	1.75710300	-0.49989200	0.01747400
C	2.99356800	-1.19144700	0.04787800
C	2.95268800	-2.60280300	0.02485800
C	1.74658100	-3.25689100	-0.02350000
H	-0.41723800	-2.97072100	-0.08359900
C	1.70129900	0.92572700	0.02401600
C	4.19534300	-0.44944900	0.10318700
H	3.89013300	-3.15794400	0.04513600
H	1.68671700	-4.34163600	-0.04355600
C	4.13369300	0.92088100	0.12863700
C	2.90498200	1.61282400	0.08931300
H	5.14621700	-0.97938300	0.13081400
H	5.05217600	1.50391900	0.17639600
H	2.90059100	2.69610100	0.10417500
N	0.57428000	-1.17932700	-0.02072700
H	-1.51265100	3.65264400	-1.21676100
Cu	-0.99919000	0.09884300	0.05919000
Br	-2.71281100	-1.52554600	-0.17110200

M6a

(U)M06/BSII SCF energy in solution: -983.75097 a.u.

(U)M06/BSI SCF energy in solution: -982.332412 a.u.

(U)M06/BSI SCF Gibbs energy in solution: -982.159997 a.u.

O	-1.38269000	3.18185800	0.01141800
N	-0.68687300	0.97458500	-0.00189200
C	-3.04699900	1.48290900	0.11464000
C	-3.27860100	0.11632100	-0.43129700

C	-1.60753600	1.96769400	0.01699900
C	-3.51601300	-0.96768700	0.33298700
H	-3.32400600	1.51662600	1.17981300
H	-3.26860200	0.00817700	-1.51949200
H	-3.71379800	-1.94247700	-0.10398300
H	-3.56967500	-0.88753600	1.42078600
C	1.50673000	-2.29693200	0.01541300
C	1.49411800	0.03484700	0.00565400
C	2.90896500	0.08622100	0.01210100
C	3.60296900	-1.14521300	0.02294200
C	2.91098700	-2.33194800	0.02496400
H	0.89551700	-3.19732900	0.01283500
C	0.68845500	1.21474500	-0.00534500
C	3.54490800	1.34862500	0.00462900
H	4.69260500	-1.13457300	0.02866000
H	3.42559900	-3.28909600	0.03253000
C	2.77020300	2.48124300	-0.00864000
C	1.35911900	2.43067300	-0.01381700
H	4.63258100	1.40036200	0.00791100
H	3.24676700	3.46052900	-0.01590400
H	0.79343500	3.35385600	-0.02267600
N	0.84395400	-1.15813700	0.00821700
H	-3.67607000	2.21703200	-0.40428400
Cu	-1.16277800	-0.94185200	-0.02397500
F	-1.38618400	-2.76564800	-0.06710100

^{T}M7a

(U)M06/BSII SCF energy in solution: -1270.810133 a.u.

(U)M06/BSI SCF energy in solution: -1269.314013 a.u.

(U)M06/BSI SCF Gibbs energy in solution: -1269.027569 a.u.

O	0.35657000	3.50967200	0.93989400
N	0.33345800	1.38336500	0.03442500
C	-1.53109500	2.88820100	-0.37496200
C	-1.67552700	2.36553600	-1.76847500
C	-0.17798600	2.61632900	0.26956900

C	-2.72113700	1.66779200	-2.21449300
H	-2.32666700	2.47732500	0.26737200
H	-0.86525500	2.61526800	-2.45969400
H	-2.78963000	1.33854200	-3.25075900
H	-3.55378900	1.40480900	-1.55869800
C	1.55942900	-2.02844300	-1.53203100
C	2.09034700	-0.20529500	-0.18263200
C	3.40273800	-0.69262000	0.03623800
C	3.75712900	-1.90553100	-0.59747900
C	2.84654200	-2.57161100	-1.38093400
H	0.78755000	-2.52202600	-2.12038900
C	1.62933800	1.01987300	0.39807500
C	4.27654200	0.04218900	0.86995000
H	4.76157900	-2.30213700	-0.45045300
H	3.09803900	-3.50645500	-1.87508700
C	3.82467600	1.20400300	1.44381200
C	2.52095200	1.69745400	1.22031500
H	5.28521200	-0.32889900	1.04599900
H	4.48402700	1.77692900	2.09460600
H	2.21451500	2.62359300	1.69018200
N	1.21228400	-0.89297300	-0.96005500
H	-1.65239400	3.98057500	-0.36398900
Cu	-0.66645000	-0.11256100	-0.80387500
N	-2.21726400	-0.19950800	0.55054500
O	-2.41179300	-2.30964100	2.32962200
C	-3.25019800	-1.17068400	0.35918200
C	-1.96014000	0.02996800	1.94029200
C	-2.73341600	-2.49033900	0.97090500
C	-1.44205400	-1.30681400	2.50846000
H	-3.44196300	-1.32148200	-0.70701400
H	-4.17110700	-0.87734400	0.88768600
H	-2.88210600	0.30013200	2.47964500
H	-1.20307600	0.81093500	2.07884700
H	-3.51256700	-3.25811800	0.91312800
H	-1.85154000	-2.82123700	0.39722200

H	-0.49881500	-1.57627600	1.99897500
H	-1.25385300	-1.20977100	3.58313200
F	-1.40616600	-1.43740000	-1.85151600

^{T}TS2a

(U)M06/BSII SCF energy in solution: -1270.796063 a.u.

(U)M06/BSI SCF energy in solution: -1269.300128 a.u.

(U)M06/BSI SCF Gibbs energy in solution: -1269.011633 a.u.

O	0.64221000	3.64287700	-0.04687500
N	0.33375400	1.35638800	-0.14764800
C	-1.43873300	2.92300300	-0.88817000
C	-2.11662200	1.83278000	-1.62899300
C	-0.04199900	2.64663900	-0.32005200
C	-3.21039100	1.16626400	-1.14895600
H	-2.06278700	3.25034100	-0.04107400
H	-1.64120200	1.47836600	-2.54881300
H	-3.69639100	0.40333000	-1.75690400
H	-3.80809600	1.59470200	-0.34218900
C	1.78834900	-2.14092500	-1.32923600
C	2.15803500	-0.19213600	-0.11150300
C	3.45999600	-0.61395900	0.25720500
C	3.89864500	-1.86314600	-0.23781600
C	3.07540000	-2.62261400	-1.03336600
H	1.07768800	-2.71236700	-1.92428700
C	1.62129700	1.06182300	0.31797500
C	4.23721500	0.21526900	1.09711700
H	4.89643900	-2.21253200	0.02718700
H	3.39329600	-3.58723000	-1.42038400
C	3.70633700	1.40296900	1.53580300
C	2.41641200	1.83029600	1.15577500
H	5.23675900	-0.10489500	1.38805700
H	4.29036200	2.04565900	2.19318400
H	2.04537500	2.78122600	1.51984300
N	1.36273100	-0.97438600	-0.88762500
H	-1.30778600	3.80574300	-1.53109000

Cu	-0.56903700	-0.33691100	-0.79064900
N	-2.30997500	-0.21010900	0.20814800
O	-2.56439500	-1.75881900	2.51816700
C	-3.18811300	-1.36182600	0.20527400
C	-2.29819000	0.42033600	1.51242300
C	-2.65268700	-2.35076100	1.23789300
C	-1.75062200	-0.60514400	2.50057400
H	-3.17738100	-1.82644000	-0.78489300
H	-4.21622200	-1.06764100	0.47651500
H	-3.31742700	0.70790600	1.82168000
H	-1.65802300	1.30977000	1.50228400
H	-3.32738700	-3.21028200	1.32549300
H	-1.66031900	-2.71451000	0.91427800
H	-0.71103900	-0.86800500	2.22331300
H	-1.74651000	-0.19399700	3.51667700
F	-1.15792500	-1.76418300	-1.81579700

M8a

(U)M06/BSII SCF energy in solution: -1270.848615 a.u.

(U)M06/BSI SCF energy in solution: -1269.355189 a.u.

(U)M06/BSI SCF Gibbs energy in solution: -1269.060174 a.u.

O	-0.67409500	3.38647700	0.79280100
N	-0.01617900	1.52520300	-0.40798300
C	-2.24466300	2.28614500	-0.67409700
C	-2.41076500	0.88877300	-1.20869400
C	-0.90475700	2.47833500	-0.00159400
C	-2.97798200	-0.19921100	-0.32112900
H	-3.04109900	2.52695100	0.04680100
H	-2.80008600	0.84367200	-2.22949400
H	-3.32092400	-1.01274400	-0.96919200
H	-3.87195200	0.19895800	0.20407400
C	1.81155100	-1.72335400	-1.67665600
C	1.93151100	0.16804900	-0.32932000
C	3.21237000	-0.19195700	0.15250600
C	3.77321600	-1.39101400	-0.34547300

C	3.08063300	-2.15576800	-1.25221400
H	1.21794000	-2.29765900	-2.38772000
C	1.25949200	1.33975500	0.13237900
C	3.84322900	0.64220700	1.10452800
H	4.75733700	-1.69942400	0.00674800
H	3.49061300	-3.08407200	-1.64130400
C	3.19453400	1.77243000	1.53581900
C	1.91285600	2.13096500	1.06415600
H	4.82768100	0.37090300	1.48281700
H	3.66991500	2.42122400	2.26984200
H	1.43623600	3.03005800	1.43545400
N	1.27098900	-0.60667100	-1.23435700
H	-2.31969900	3.02532700	-1.48418400
Cu	-0.61072800	0.11541100	-1.48852300
N	-2.01006900	-0.75927000	0.62179600
O	-1.04109900	-2.05510800	2.94939400
C	-2.18741700	-2.19291100	0.82290700
C	-1.97587500	-0.07994600	1.91178800
C	-1.07333400	-2.71938800	1.69902400
C	-0.87221500	-0.66191300	2.76763400
H	-2.14666200	-2.69512100	-0.15373700
H	-3.16782800	-2.41989400	1.29070000
H	-2.94863300	-0.18786900	2.43598100
H	-1.79104000	0.99444100	1.77786800
H	-1.21404100	-3.78662900	1.90686400
H	-0.10359300	-2.58857900	1.18000400
H	0.11082700	-0.45919200	2.29823300
H	-0.87681800	-0.20631900	3.76464500
F	-1.26922300	-1.15932500	-2.57226300

TS3a

(U)M06/BSII SCF energy in solution: -1270.808812 a.u.

(U)M06/BSI SCF energy in solution: -1269.325189 a.u.

(U)M06/BSI SCF Gibbs energy in solution: -1269.033337 a.u.

O	1.39308400	3.66078800	0.49380800

N	0.82179100	1.46082600	-0.01194400
C	-0.78114700	3.18754400	-0.37719100
C	-1.87776400	2.19918100	-0.29763800
C	0.60749400	2.78096200	0.11283300
C	-2.14401100	1.43311800	0.97524600
H	-1.14428100	4.05367700	0.21003400
H	-2.76317300	2.46692500	-0.86402400
C	0.98910300	-2.35537000	-1.17620100
C	2.06975400	-0.55929400	-0.18240700
C	3.24213200	-1.33352400	0.02639600
C	3.22428400	-2.68045600	-0.40266100
C	2.10346700	-3.19565100	-1.00165400
H	0.08367900	-2.73297700	-1.65375800
C	2.02075200	0.81900600	0.23519800
C	4.37013100	-0.74480700	0.64384000
H	4.11252600	-3.29300600	-0.24880800
H	2.06108500	-4.22723400	-1.34188600
C	4.31237300	0.57043300	1.03213700
C	3.15589700	1.35082300	0.83447800
H	5.26434900	-1.34704400	0.80011500
H	5.17587800	1.03333000	1.50856100
H	3.14383700	2.38809000	1.15153100
N	0.96797000	-1.09427700	-0.78728000
H	-0.69327900	3.56403700	-1.40676500
Cu	-0.60294900	0.31115100	-0.80718800
N	-2.21689700	-0.00264000	0.71311700
O	-3.17782200	-2.64862700	0.97731200
C	-3.49800300	-0.40770500	0.12913100
C	-1.91504700	-0.80135400	1.90102700
C	-3.45561900	-1.88551500	-0.18195200
C	-1.93257500	-2.26809100	1.53282500
H	-3.65438700	0.15847900	-0.79586500
H	-4.32353000	-0.19130700	0.83474800
H	-2.65160000	-0.60248300	2.70376500
H	-0.91596200	-0.52417100	2.26851000

H	-4.42286400	-2.22689800	-0.56816900
H	-2.68471900	-2.07966200	-0.95320500
H	-1.12156900	-2.47452900	0.80883000
H	-1.77148600	-2.89020900	2.42047000
H	-1.32764400	1.60978200	1.68611700
H	-3.08271800	1.82089000	1.42036900
F	-1.94200800	1.01894200	-2.06208300

M9a

(U)M06/BSII SCF energy in solution: -1270.867265 a.u.

(U)M06/BSI SCF energy in solution: -1269.393685 a.u.

(U)M06/BSI SCF Gibbs energy in solution: -1269.098598 a.u.

O	1.04510100	3.29952300	1.15181900
N	0.81618500	1.39011800	-0.14938000
C	-0.82640900	3.20311100	-0.27963600
C	-2.03471000	2.38844600	-0.69339100
C	0.43837100	2.59295700	0.32447900
C	-2.66015100	1.53202600	0.42429200
H	-1.18754400	3.95474600	0.43614800
H	-2.79331800	3.10526900	-1.03709200
C	1.31073500	-2.60249300	-0.45168700
C	2.28102500	-0.51880300	-0.09953500
C	3.57884100	-1.09816600	-0.03627500
C	3.68147300	-2.49891300	-0.19926300
C	2.55597800	-3.25310300	-0.40981600
H	0.39694300	-3.17435400	-0.61454500
C	2.09637500	0.90341500	0.04768500
C	4.70595100	-0.27576700	0.18542900
H	4.66718800	-2.96208900	-0.15341100
H	2.60640600	-4.33103900	-0.54130000
C	4.52240400	1.07780900	0.33170600
C	3.24448400	1.66156200	0.26723200
H	5.69464300	-0.72972600	0.24004500
H	5.38172000	1.72571100	0.50200800
H	3.14672600	2.73619900	0.38444500

N	1.17625200	-1.29696800	-0.30240400
H	-0.49826200	3.77204400	-1.16465400
Cu	-0.52462600	-0.22117000	-0.20388100
N	-2.45762200	0.07862300	0.29293600
O	-3.45257300	-2.60175100	0.52757200
C	-3.40805000	-0.50727200	-0.67801200
C	-2.71117400	-0.56219800	1.60327600
C	-3.23511200	-2.00614300	-0.73768600
C	-2.57251900	-2.06344600	1.49581200
H	-3.23892600	-0.06274500	-1.66292600
H	-4.43847000	-0.26650700	-0.35743200
H	-3.73048100	-0.30273000	1.94372600
H	-1.99084200	-0.17014300	2.33333400
H	-3.96578400	-2.44294400	-1.42807900
H	-2.22073600	-2.25293700	-1.10536500
H	-1.52638600	-2.32459400	1.24637200
H	-2.82318000	-2.53283300	2.45416200
H	-2.21529200	1.83734500	1.38162700
H	-3.74241900	1.73584100	0.49536900
F	-1.73298200	1.60852100	-1.81452600

M10a

(U)M06/BSII SCF energy in solution: -1570.638388 a.u.

(U)M06/BSI SCF energy in solution: -1569.038626 a.u.

(U)M06/BSI SCF Gibbs energy in solution: -1568.609838 a.u.

O	-2.66706000	-1.27829400	-0.32142000
N	2.30077900	-1.43519000	-1.42671500
N	-0.89569700	0.16260300	-0.19490600
C	-0.50194800	-2.18733000	0.18752100
O	5.10980700	-1.12895300	-1.95028100
C	0.16411600	-2.77565300	-1.04750000
C	-1.47156000	-1.06976100	-0.12936300
C	0.98077300	-1.84822900	-1.93717700
C	2.84018800	-0.40479900	-2.35152400
C	3.24671900	-2.57947500	-1.41506900

C	4.24370700	-0.01104800	-1.95556900
C	4.63908300	-2.11417400	-1.05190700
H	0.11158500	0.21954500	-0.03422900
H	0.26936500	-1.85379900	0.89789500
H	0.76075000	-3.64620100	-0.73934000
H	1.12146800	-2.37507700	-2.89675900
H	0.40210000	-0.93944000	-2.15260100
H	2.17679100	0.47058100	-2.32223200
H	2.84195600	-0.81158900	-3.37851600
H	3.25442700	-3.04455000	-2.41678000
H	2.90741200	-3.32504700	-0.68599700
H	4.64731900	0.71050900	-2.67518500
H	4.23788400	0.46493100	-0.95484000
H	4.64655000	-1.71593900	-0.01824600
H	5.33787700	-2.95690200	-1.10026300
C	1.64918900	3.18021600	-0.75799300
C	-0.53404300	2.46691500	-0.71299100
C	-1.02363300	3.76758700	-1.00514200
C	-0.06603400	4.79578500	-1.16285500
C	1.26863200	4.50871900	-1.03830500
H	2.70859500	2.92800400	-0.66532300
C	-1.46451100	1.39495100	-0.52959200
C	-2.41636100	3.98452300	-1.13031500
H	-0.40696600	5.80732200	-1.38299000
H	2.02945500	5.27690400	-1.15427800
C	-3.28133800	2.93248700	-0.97139600
C	-2.81437900	1.63514200	-0.67245200
H	-2.77806700	4.98747400	-1.35372400
H	-4.35545000	3.08426800	-1.06673700
H	-3.52276700	0.82421300	-0.54763200
N	0.79186400	2.19374300	-0.59961500
H	-1.07206200	-2.98931400	0.67095000
Cu	2.31555800	-0.61059300	0.40482000
H	-4.41359100	-2.22552100	0.74551400
I	2.48475500	0.30636900	2.71012800

N	-5.40784200	-2.38135300	0.93530200
O	-6.46714900	0.17412800	0.14570800
C	-5.84499300	-1.37605200	1.90427000
C	-6.13117400	-2.18416200	-0.32170800
C	-5.74620600	0.03483900	1.36182000
C	-6.03608700	-0.76093500	-0.83473900
H	-5.24881300	-1.46522500	2.82247100
H	-6.89316500	-1.59025900	2.16735300
H	-7.18938100	-2.43745500	-0.14844400
H	-5.74379700	-2.87977500	-1.07872200
H	-6.16929600	0.76777300	2.06136500
H	-4.68269100	0.29293000	1.20061800
H	-4.99239700	-0.54609300	-1.12757200
H	-6.67625100	-0.60529300	-1.71290800
F	-0.85348700	-3.24536300	-1.87645000

1a

(U)M06/BSII SCF energy in solution: -2861.093742 a.u.

(U)M06/BSI SCF energy in solution: -2858.537088 a.u.

(U)M06/BSI SCF Gibbs energy in solution: -2858.443931 a.u.

N	-0.00034600	0.00000000	0.42938600
O	-2.71042100	0.00000000	-0.34686700
C	-0.61719900	-1.19357100	-0.15734500
C	-0.61719900	1.19357100	-0.15734500
C	-2.09616000	-1.16013800	0.17915700
C	-2.09616000	1.16013800	0.17915700
H	-0.15601100	-2.09024700	0.27410800
H	-0.48996200	-1.22469000	-1.25512500
H	-0.48996200	1.22469000	-1.25512500
H	-0.15601100	2.09024700	0.27410800
H	-2.59924700	-2.02608000	-0.26644200
H	-2.23380300	-1.19899400	1.27404800
H	-2.23380300	1.19899400	1.27404800
H	-2.59924700	2.02608000	-0.26644200
Br	1.86297500	0.00000000	-0.01559100

S3

(U)M06/BSII SCF energy in solution: -287.04099 a.u.

(U)M06/BSI SCF energy in solution: -286.958451 a.u.

(U)M06/BSI SCF Gibbs energy in solution: -286.866086 a.u.

N	-0.00000700	1.41766500	0.33671200
O	0.00000500	-1.33919400	-0.30108200
C	1.17246300	0.77083600	-0.18712500
C	-1.17247100	0.77082400	-0.18713400
C	1.16232200	-0.71142200	0.19682100
C	-1.16231800	-0.71142600	0.19683000
H	2.08150900	1.24843000	0.20095200
H	1.19240800	0.82861500	-1.29083700
H	-1.19240000	0.82858600	-1.29084700
H	-2.08152000	1.24842300	0.20092800
H	2.02624600	-1.22716600	-0.23776800
H	1.20821000	-0.81134300	1.29530800
H	-1.20818600	-0.81133700	1.29531900
H	-2.02623900	-1.22718900	-0.23774000

2a

(U)M06/BSII SCF energy in solution: -687.056537 a.u.

(U)M06/BSI SCF energy in solution: -686.874659 a.u.

(U)M06/BSI SCF Gibbs energy in solution: -686.691872 a.u.

O	2.63485600	1.64671500	-0.43350800
N	1.08773400	-0.03994200	-0.48351200
C	3.40652200	-0.58766500	-0.84264800
C	3.94572300	-1.20226800	0.41757100
C	2.35198500	0.46153000	-0.57970200
C	3.61577000	-0.86739000	1.66205600
H	0.96445800	-1.04554600	-0.59684900
H	3.01472400	-1.37905300	-1.49886700
H	4.68251300	-1.99188200	0.25521400
H	4.06899000	-1.36904100	2.51574300
H	2.89424000	-0.08208400	1.89262900

C	-2.16624700	-2.31845300	-0.23951000
C	-1.26621600	-0.21124300	-0.11901100
C	-2.53013100	0.37410400	0.15778300
C	-3.64247800	-0.49624200	0.22655200
C	-3.46554000	-1.84108200	0.02981200
H	-2.00656000	-3.38639400	-0.39964100
C	-0.10657500	0.62561800	-0.19750500
C	-2.63004000	1.77214100	0.35089700
H	-4.62758300	-0.07923100	0.43589000
H	-4.29921800	-2.53763600	0.07699600
C	-1.50141400	2.54689000	0.27282400
C	-0.23612600	1.98477900	0.00049700
H	-3.60530700	2.21090900	0.55912600
H	-1.56812000	3.62326300	0.42188100
H	0.63949500	2.62080900	-0.05346400
N	-1.10376000	-1.54577600	-0.31429400
H	4.22430700	-0.10212800	-1.39365800

3a

(U)M06/BSII SCF energy in solution: -1074.038804 a.u.

(U)M06/BSI SCF energy in solution: -1073.745452 a.u.

(U)M06/BSI SCF Gibbs energy in solution: -1073.437807 a.u.

O	-0.21804800	3.45515800	-0.63030200
N	2.26929000	-0.40645000	0.29888000
N	-0.39787000	1.17198300	-0.56256300
C	1.74803100	2.17338200	-1.08241100
O	3.02302900	-3.14079700	-0.01455700
C	2.62245800	2.04471300	0.15373800
C	0.28449900	2.34143000	-0.74271300
C	2.31013400	0.84661000	1.03263100
C	1.71637500	-1.46804000	1.13771100
C	3.60542500	-0.79714000	-0.15524600
C	1.71242800	-2.77403000	0.37761100
C	3.56348500	-2.13776300	-0.85327000
H	0.12519700	0.30139600	-0.68052300

H	1.91517800	1.29841800	-1.72250100
H	3.67636500	2.04950500	-0.15782700
H	3.04798700	0.81421200	1.86038500
H	1.32431500	1.01502800	1.49163400
H	0.68689600	-1.20169400	1.41977300
H	2.30731800	-1.58616900	2.07008600
H	4.30369100	-0.85166400	0.70624600
H	3.99930100	-0.04914400	-0.85682000
H	1.31844800	-3.58757000	0.99845100
H	1.07676300	-2.67171100	-0.52051500
H	2.96233200	-2.06047200	-1.77800200
H	4.57633400	-2.45534700	-1.12764100
C	-1.80249800	-2.58662600	-0.48393500
C	-2.22119400	-0.34860400	-0.19349300
C	-3.57753100	-0.59617000	0.15333100
C	-4.00969300	-1.94210500	0.16250600
C	-3.12717200	-2.94014400	-0.15580900
H	-1.08827800	-3.37185300	-0.74099700
C	-1.74271900	1.00134600	-0.21622700
C	-4.43634400	0.48132800	0.47031900
H	-5.04459300	-2.16331200	0.42366200
H	-3.42523700	-3.98585600	-0.16007000
C	-3.94900500	1.76202800	0.44089500
C	-2.60718200	2.03065700	0.10082000
H	-5.47316600	0.27260600	0.73199900
H	-4.59889400	2.60047700	0.68552100
H	-2.24839800	3.05264600	0.08697000
N	-1.35485900	-1.34848400	-0.50690800
H	2.05235900	3.06938100	-1.63575800
F	2.43481500	3.17018400	0.95335500

^{T}M6b

(U)M06/BSII SCF energy in solution: -3745.001999 a.u.

(U)M06/BSI SCF energy in solution: -3741.0861 a.u.

(U)M06/BSI SCF Gibbs energy in solution: -3740.801984 a.u.

O	1.31598800	3.66670500	0.03933600
N	0.91604000	1.42986300	-0.38765200
C	-0.68113600	3.09795900	-1.13092100
C	-0.99678700	2.19980000	-2.28203300
C	0.62199200	2.75325100	-0.42414900
C	-2.14253000	1.53210900	-2.43352300
H	-1.50296900	3.08404700	-0.39647900
H	-0.21551900	2.10078200	-3.04085100
H	-2.32899500	0.89966100	-3.29944200
H	-2.95251700	1.62080000	-1.70792700
C	1.28566900	-2.50756000	-0.64814200
C	2.28563000	-0.48840400	-0.07025300
C	3.50619200	-1.13705900	0.24663200
C	3.56222000	-2.53892400	0.07965400
C	2.46159700	-3.22399900	-0.37060400
H	0.37916000	-3.00606500	-0.99085800
C	2.14056300	0.93172400	0.04638200
C	4.59548000	-0.36813900	0.71599900
H	4.48959900	-3.06111000	0.31457000
H	2.47857700	-4.30177500	-0.50939700
C	4.44247300	0.98860400	0.85133800
C	3.23576000	1.64156400	0.52606600
H	5.52993300	-0.86745800	0.96825400
H	5.27131100	1.59284700	1.21770300
H	3.16207300	2.71580400	0.64138100
N	1.20515500	-1.19857900	-0.50174600
H	-0.57763600	4.13926600	-1.46688700
Cu	-0.47082200	0.00925700	-0.58023200
N	-1.61899500	0.72908100	0.96876000
O	-2.65255800	-0.43358400	3.28288000
C	-3.03776700	0.65371800	1.14797100
C	-0.93039900	0.88015000	2.21823400
C	-3.34780600	-0.54693200	2.06361700
C	-1.26272200	-0.34939500	3.08094900
H	-3.54474700	0.51889100	0.18646100

H	-3.40245600	1.57117000	1.63983300
H	-1.28347800	1.78060300	2.74820800
H	0.15270500	0.95039400	2.06077900
H	-4.41863700	-0.56898200	2.29324000
H	-3.06762600	-1.47078200	1.53014700
H	-0.88027100	-1.25890000	2.58282900
H	-0.78679900	-0.25461200	4.06283500
Br	-2.00289400	-1.71861000	-1.16697800

^{T}TS2b

(U)M06/BSII SCF energy in solution: -3744.988353 a.u.

(U)M06/BSI SCF energy in solution: -3741.07125 a.u.

(U)M06/BSI SCF Gibbs energy in solution: -3740.786296 a.u.

O	1.42357000	3.41565700	-1.38795700
N	0.73262300	1.38495700	-0.54203800
C	-0.89060200	2.98996600	-1.51482600
C	-1.90351800	1.91924900	-1.67332800
C	0.53767600	2.58790400	-1.13724900
C	-2.92774500	1.72037100	-0.78972300
H	-1.21889400	3.71025900	-0.74810300
H	-1.75954600	1.20787900	-2.49130600
H	-3.67976300	0.95564700	-0.98161800
H	-3.20307200	2.50382200	-0.08121600
C	1.41174900	-2.53485100	-0.37099000
C	2.26219300	-0.39186200	-0.06323100
C	3.53061300	-0.90474400	0.31251300
C	3.69101300	-2.30830900	0.31330200
C	2.64137000	-3.12264600	-0.03214800
H	0.54434000	-3.13662100	-0.64041400
C	2.01190700	1.01314800	-0.11803300
C	4.55599300	-0.00522700	0.68152400
H	4.65600400	-2.72833600	0.59596300
H	2.73821300	-4.20500400	-0.03916200
C	4.29560800	1.34228100	0.67218500
C	3.04285500	1.85426400	0.27719500

H	5.52680600	-0.39750600	0.98097200
H	5.07095700	2.04596400	0.97129100
H	2.88861600	2.92710100	0.26929300
N	1.23381100	-1.22668000	-0.38029300
H	-0.78362100	3.56141500	-2.44808800
Cu	-0.52617900	-0.19303000	-0.40274800
N	-1.94815500	0.61724600	0.76693800
O	-1.83095600	-0.04492300	3.48599700
C	-2.98379500	-0.20562400	1.36215300
C	-1.47999400	1.59035900	1.73919400
C	-2.35182500	-0.95048700	2.53552800
C	-0.87422600	0.81717500	2.90754800
H	-3.37114300	-0.92014900	0.62911300
H	-3.80862400	0.42661200	1.73226600
H	-2.32192500	2.19769300	2.11112800
H	-0.72684600	2.24991400	1.29519100
H	-3.10369900	-1.56279900	3.04678100
H	-1.55021800	-1.61659700	2.16343000
H	0.00402600	0.24021800	2.55900500
H	-0.54499500	1.51022800	3.69041000
Br	-1.83948400	-1.94949100	-1.34975700

M7b

(U)M06/BSII SCF energy in solution: -3745.047774 a.u.

(U)M06/BSI SCF energy in solution: -3741.13301 a.u.

(U)M06/BSI SCF Gibbs energy in solution: -3740.842177 a.u.

O	0.96676900	3.59680800	-1.08180100
N	0.58878600	1.32167800	-0.94528400
C	-1.26472500	2.72578300	-1.38265000
C	-2.03107700	1.52324000	-0.90722300
C	0.21936100	2.62150900	-1.11418500
C	-2.46175600	1.43493900	0.54708400
H	-1.65439800	3.63374000	-0.89607700
H	-2.79601400	1.18652300	-1.60919200
H	-3.40848300	0.88309000	0.59154000

H	-2.65658700	2.45409000	0.93363500
C	0.90011500	-2.57146300	-0.40197800
C	1.99963700	-0.52493300	-0.46687400
C	3.24321900	-1.12784900	-0.15957800
C	3.25390600	-2.52903700	0.02744300
C	2.09112600	-3.25001700	-0.09029500
H	-0.04855800	-3.09877900	-0.51182200
C	1.88149000	0.88599400	-0.65492500
C	4.39058400	-0.30955200	-0.04288000
H	4.19584100	-3.02196900	0.26711300
H	2.07384400	-4.32725100	0.05198100
C	4.26749200	1.04549500	-0.22367200
C	3.02909400	1.65287200	-0.52362600
H	5.34972900	-0.76830300	0.19259200
H	5.14476800	1.68439000	-0.13527700
H	2.97427700	2.72662100	-0.66004500
N	0.86022200	-1.26585600	-0.57979200
H	-1.39537300	2.86757200	-2.46481500
Cu	-0.73323600	-0.03320600	-0.86610400
N	-1.48431900	0.73299200	1.39050100
O	-0.14483700	-0.11716900	3.73782000
C	-2.10005400	-0.21382000	2.31542800
C	-0.59354000	1.62840300	2.12311200
C	-1.01269800	-0.98369000	3.02932900
C	0.45218100	0.81942600	2.85944800
H	-2.72452000	-0.91226700	1.74255900
H	-2.74238600	0.30678900	3.05423600
H	-1.16812900	2.23923400	2.84910800
H	-0.09608100	2.31860200	1.42816200
H	-1.44079400	-1.67637500	3.76350300
H	-0.43762300	-1.57335600	2.28843900
H	1.10204600	0.29193800	2.13432300
H	1.08392600	1.47393500	3.47175300
Br	-2.40017300	-1.60136800	-1.17956000

TS3b

(U)M06/BSII SCF energy in solution: -3745.019762 a.u.

(U)M06/BSI SCF energy in solution: -3741.112739 a.u.

(U)M06/BSI SCF Gibbs energy in solution: -3740.822408 a.u.

O	0.85012600	-3.67089900	-1.09250400
N	0.68927300	-1.50038900	-0.26626900
C	-1.26294400	-2.87538400	-0.31244700
C	-2.15302000	-1.67195600	-0.40921800
C	0.23316500	-2.72003900	-0.62206000
C	-2.01524900	-0.75950200	-1.62672700
H	-1.68351400	-3.62139600	-1.01161500
H	-3.18737900	-1.93130100	-0.19205600
C	1.47189700	1.91274300	1.63905100
C	2.28523200	0.12862400	0.39672000
C	3.61070100	0.63877100	0.45602300
C	3.80707000	1.85554800	1.14586500
C	2.74821200	2.49435700	1.74153800
H	0.60373700	2.38470500	2.10314300
C	2.00915700	-1.11572200	-0.27550700
C	4.66112700	-0.07972400	-0.15735800
H	4.81362000	2.27097100	1.20229200
H	2.87749000	3.42782600	2.28358500
C	4.38318900	-1.26905000	-0.78681700
C	3.07872900	-1.79360300	-0.84870100
H	5.67427100	0.31813800	-0.11383500
H	5.19075000	-1.83061600	-1.25473000
H	2.88176600	-2.74524500	-1.33361800
N	1.24713500	0.78823000	0.98980000
H	-1.36508200	-3.33007300	0.68416600
Cu	-0.54582100	-0.21114000	0.54855700
N	-1.55519100	0.59601300	-1.32888000
O	-1.21878900	3.37749000	-1.72325700
C	-2.66124100	1.53749500	-1.14204000
C	-0.61944900	1.11111700	-2.33148900
C	-2.10010400	2.88451400	-0.74282600

C	-0.14216700	2.48891900	-1.91761400
H	-3.32577800	1.15457500	-0.35705200
H	-3.24036800	1.63732300	-2.08175900
H	-1.10313800	1.16642200	-3.32695100
H	0.23602900	0.42346400	-2.39071000
H	-2.90133400	3.62381000	-0.63162200
H	-1.58087800	2.78392500	0.23120500
H	0.45336400	2.40423800	-0.98969800
H	0.49349700	2.92267300	-2.69779800
H	-1.27243500	-1.22705700	-2.28795000
H	-2.97866100	-0.73363900	-2.16863600
Br	-2.52806200	-0.46908700	1.71287600

M8b

(U)M06/BSII SCF energy in solution: -3745.046437 a.u.

(U)M06/BSI SCF energy in solution: -3741.141711 a.u.

(U)M06/BSI SCF Gibbs energy in solution: -3740.8499 a.u.

O	0.20027500	-2.71251000	1.72618700
N	-0.34824400	-1.57930000	-0.23111700
C	1.87148300	-2.48747300	0.05098100
C	2.80388700	-1.28396200	0.11489900
C	0.45178400	-2.28158500	0.59567500
C	2.62368100	-0.37054100	1.32777000
H	2.32817200	-3.25390600	0.69408800
H	3.84503800	-1.62334500	0.06756400
C	-2.06342500	1.82649500	-1.57264100
C	-2.31388200	-0.22584400	-0.50320000
C	-3.71940800	-0.02954200	-0.38728100
C	-4.26074500	1.15621100	-0.93108900
C	-3.44437800	2.08253000	-1.52775300
H	-1.37883700	2.55099600	-2.01464200
C	-1.68914800	-1.40919300	0.03092000
C	-4.50890500	-0.99589600	0.27068200
H	-5.33604900	1.32158900	-0.85913700
H	-3.83818100	3.00436000	-1.94845400

C	-3.89758000	-2.10495900	0.80610200
C	-2.51317300	-2.31149800	0.69863300
H	-5.58344200	-0.83911300	0.35537000
H	-4.49562900	-2.85068000	1.32843600
H	-2.06005900	-3.19313500	1.14129700
N	-1.52058100	0.72704800	-1.08362700
H	1.84069600	-2.88154600	-0.97248800
Cu	0.41090300	0.23953500	-0.85360900
N	1.56137000	0.63961200	1.21487200
O	0.13926500	2.94287800	2.10851500
C	2.13046700	1.99226600	1.14098800
C	0.62003900	0.58021900	2.34865900
C	1.02045300	3.01298000	1.01666900
C	-0.43542800	1.65566400	2.20713600
H	2.80072600	2.04744400	0.27250200
H	2.72282600	2.20863800	2.05244700
H	1.16861900	0.73577300	3.29864600
H	0.15582900	-0.41532500	2.37712500
H	1.43289200	4.02880600	1.00131100
H	0.47481000	2.84478000	0.06521300
H	-1.06212200	1.45304900	1.32049100
H	-1.08396800	1.66526600	3.09015200
H	2.39828200	-1.03825700	2.17432200
H	3.58195000	0.12413200	1.55104600
Br	2.67296600	-0.17216400	-1.55263000

M9b

(U)M06/BSII SCF energy in solution: -4044.81934 a.u.

(U)M06/BSI SCF energy in solution: -4040.782718 a.u.

(U)M06/BSI SCF Gibbs energy in solution: -4040.353833 a.u.

O	-2.60377700	-0.61337100	0.58317400
N	2.33739000	-1.70691000	-0.78856500
N	-0.71537600	0.42924500	-0.15913400
C	-0.50275700	-1.75163000	0.86519400
O	5.17765400	-2.02053100	-1.06240800

C	0.02343700	-2.64023900	-0.25753000
C	-1.38692500	-0.59878200	0.42634200
C	0.99249000	-2.07024600	-1.28875900
C	3.10013700	-1.16341500	-1.94219400
C	3.05859600	-2.89800700	-0.27772700
C	4.52754900	-0.86246800	-1.54933100
C	4.49031600	-2.54200200	0.05774100
H	0.30381000	0.39604300	-0.14914400
H	0.35724000	-1.37397600	1.44608000
H	0.43131600	-3.55365400	0.18689300
H	1.12096600	-2.82825200	-2.08017800
H	0.56209200	-1.17811700	-1.76466300
H	2.59644900	-0.25203000	-2.29194200
H	3.09081500	-1.90762500	-2.75827400
H	3.03241700	-3.69038600	-1.04730100
H	2.55974000	-3.26715900	0.62697000
H	5.09067500	-0.51736000	-2.42374800
H	4.55311900	-0.06479800	-0.78074700
H	4.50687100	-1.80563300	0.88475300
H	5.03165900	-3.43763500	0.38199900
C	2.03427900	2.78486000	-1.79727100
C	-0.19053800	2.42658900	-1.34713300
C	-0.57140300	3.64388900	-1.97074400
C	0.46366800	4.43871700	-2.51573000
C	1.76528900	4.01684700	-2.42812000
H	3.06346600	2.42532000	-1.71920100
C	-1.19818500	1.59226500	-0.76404700
C	-1.93830400	4.00658700	-2.02631800
H	0.20797200	5.38068200	-3.00075100
H	2.58272000	4.60667000	-2.83566500
C	-2.88166900	3.17645900	-1.47726700
C	-2.52187600	1.96831700	-0.84249300
H	-2.21925900	4.94153700	-2.50977000
H	-3.93678200	3.44268300	-1.51685100
H	-3.29052900	1.33471500	-0.41439200

N	1.10197200	2.01318400	-1.28002400
H	-1.10289800	-2.37053700	1.54112600
Cu	2.38718200	-0.29604000	0.64053100
H	-4.46665800	-1.35114400	1.62955700
I	2.64316200	1.33496000	2.50082300
N	-5.47794700	-1.36799400	1.78773700
O	-6.22291300	0.97947500	0.30294600
C	-5.84973400	-0.11026900	2.43677700
C	-6.12487600	-1.44049400	0.47698600
C	-5.57840800	1.09838700	1.56398300
C	-5.85312900	-0.21543700	-0.37320400
H	-5.30925000	-0.01409600	3.38812200
H	-6.92554300	-0.15260100	2.67060900
H	-7.21021700	-1.54034000	0.63770600
H	-5.78406200	-2.34177900	-0.05127300
H	-5.95974800	2.02092700	2.02101700
H	-4.48826200	1.21501700	1.42197200
H	-4.77983000	-0.18552300	-0.63474200
H	-6.43160800	-0.23416800	-1.30602900
Br	-1.51943700	-3.29603800	-1.31416800

4

(U)M06/BSII SCF energy in solution: -3548.220668 a.u.

(U)M06/BSI SCF energy in solution: -3545.489803 a.u.

(U)M06/BSI SCF Gibbs energy in solution: -3545.184054 a.u.

O	0.74204000	-2.78324600	1.28461100
N	1.28541100	1.71831900	-0.12912100
N	-0.38611500	-0.87289600	0.72028300
C	1.90593800	-0.70203400	1.49602900
O	0.65470200	4.49944300	-0.04900200
C	2.71991900	-0.24838900	0.29354900
C	0.70712600	-1.56462300	1.16141400
C	1.97343900	0.58831900	-0.73420000
C	0.40280900	2.34631100	-1.11103600
C	2.23330300	2.71461400	0.37428600

C	-0.28733900	3.53979500	-0.49381600
C	1.50058700	3.91803600	0.92490500
H	-0.32867300	0.14842900	0.72313500
H	1.58777500	0.20040800	2.03783500
H	3.61564700	0.26866400	0.65173200
H	2.68905500	0.91878100	-1.51494200
H	1.22210900	-0.03851800	-1.23656400
H	-0.34810400	1.61245700	-1.43821600
H	0.97365800	2.67334300	-2.00526000
H	2.91724600	3.03858000	-0.43807500
H	2.84731800	2.27977600	1.17441300
H	-0.93857400	4.03561500	-1.22343600
H	-0.90057900	3.20506000	0.36230200
H	0.90493300	3.62251800	1.80823200
H	2.21519400	4.68959400	1.23410500
C	-3.37541000	1.75059900	0.18697000
C	-2.67771600	-0.43574700	0.14205600
C	-3.96253500	-0.89055400	-0.26220600
C	-4.97128500	0.08484900	-0.43359300
C	-4.68399700	1.40536200	-0.20848500
H	-3.13215600	2.79949500	0.37174900
C	-1.62754800	-1.39051500	0.33389400
C	-4.19195900	-2.27004000	-0.47081700
H	-5.96707400	-0.23344900	-0.74229700
H	-5.43662600	2.18112700	-0.32836800
C	-3.16657500	-3.15983000	-0.28107300
C	-1.88439900	-2.73045700	0.11878300
H	-5.18281000	-2.60174400	-0.77934600
H	-3.33036500	-4.22423500	-0.44097100
H	-1.09416300	-3.45804900	0.26159700
N	-2.40450900	0.87802400	0.35871300
H	2.54618700	-1.29011400	2.16269000
Br	3.45189800	-1.81016100	-0.66840800

^{S}M7a

(U)M06/BSII SCF energy in solution: -1270.805072 a.u.

(U)M06/BSI SCF energy in solution: -1269.278865 a.u.

(U)M06/BSI SCF Gibbs energy in solution: -1268.990744 a.u.

O	0.10219400	3.52559700	1.20627100
N	0.15229800	1.54970300	-0.00053300
C	-1.84804800	2.90043800	-0.03138100
C	-2.08833000	2.31217900	-1.38215400
C	-0.41363300	2.70133700	0.46227800
C	-2.97059900	1.33907600	-1.65305500
H	-2.53347300	2.45117100	0.70449800
H	-1.47077500	2.70530300	-2.19444400
H	-3.09011200	0.92736100	-2.65192100
H	-3.61407200	0.92854100	-0.87402100
C	1.23646400	-1.85256900	-1.64809100
C	1.89384700	-0.01542900	-0.37056300
C	3.21840700	-0.50550400	-0.24917100
C	3.50372700	-1.73458700	-0.88617900
C	2.52749200	-2.40721200	-1.58195000
H	0.40211400	-2.32716500	-2.16479400
C	1.47461300	1.20899300	0.24005000
C	4.15391600	0.24283000	0.49910000
H	4.51230600	-2.14202200	-0.81133400
H	2.73424600	-3.35469100	-2.07305100
C	3.74538400	1.41468300	1.08843600
C	2.42794700	1.90417200	0.97475200
H	5.17495500	-0.12187400	0.60176000
H	4.45728100	1.99699300	1.67177800
H	2.14298200	2.83147500	1.45678400
N	0.95445400	-0.70728500	-1.06419100
H	-2.03307700	3.98217000	-0.02619500
Cu	-0.89790700	0.12663500	-0.87121500
N	-1.98857900	-0.45064500	0.96003900
O	-1.21271100	-2.87518600	2.08835300
C	-2.83913000	-1.59627700	0.83456600
C	-1.28281300	-0.46378100	2.20512100

C	-1.94273700	-2.85047600	0.88851000
C	-0.40846400	-1.73873500	2.23573800
H	-3.36250000	-1.57132600	-0.12680400
H	-3.55518400	-1.64833800	1.67293400
H	-1.98066200	-0.52114000	3.05842100
H	-0.65543000	0.43282700	2.29941500
H	-2.55674000	-3.75730100	0.86279600
H	-1.28718000	-2.82659200	0.00299300
H	0.34776600	-1.67884500	1.43183100
H	0.10622800	-1.81998600	3.19958900
F	-1.66340900	-1.25105600	-1.79502600

^{S}TS2a

(U)M06/BSII SCF energy in solution: -1270.793985 a.u.

(U)M06/BSI SCF energy in solution: -1269.297986 a.u.

(U)M06/BSI SCF Gibbs energy in solution: -1269.008716 a.u.

O	0.62826300	3.64269700	0.01168900
N	0.32394800	1.35941300	-0.14322900
C	-1.43054000	2.94437400	-0.89586600
C	-2.12476700	1.84957500	-1.61673600
C	-0.05084700	2.65456100	-0.29756300
C	-3.21463400	1.19541000	-1.11455500
H	-2.05715800	3.31088900	-0.06731800
H	-1.67124200	1.49030800	-2.54577300
H	-3.71894000	0.43300600	-1.70786000
H	-3.78845900	1.62820200	-0.29289700
C	1.78309700	-2.12414000	-1.35621400
C	2.14832100	-0.18987000	-0.11284100
C	3.45173300	-0.61290600	0.24881700
C	3.89446900	-1.85286600	-0.26547500
C	3.07305600	-2.60415900	-1.07068900
H	1.07349000	-2.68954800	-1.95828300
C	1.60875300	1.05836000	0.32952400
C	4.22642900	0.20700000	1.10005300
H	4.89445700	-2.20160200	-0.00810200

H	3.39464700	-3.56117300	-1.47314400
C	3.69128900	1.38641900	1.55534700
C	2.40077800	1.81639000	1.17977300
H	5.22727100	-0.11345000	1.38610200
H	4.27289900	2.02171000	2.22197000
H	2.02822100	2.76175400	1.55598100
N	1.35467100	-0.96571000	-0.89686100
H	-1.27028400	3.80615800	-1.56015400
Cu	-0.57033800	-0.31591500	-0.81127900
N	-2.31204200	-0.21475800	0.23050600
O	-2.52032500	-1.82233400	2.50902800
C	-3.18695400	-1.36813800	0.21872000
C	-2.28380300	0.38071200	1.54968600
C	-2.63036600	-2.38298900	1.21678500
C	-1.71109000	-0.66578700	2.50289800
H	-3.19513900	-1.80897300	-0.78287000
H	-4.21082000	-1.08686600	0.51858100
H	-3.29836800	0.65226500	1.88785800
H	-1.64835300	1.27408100	1.55138300
H	-3.30157700	-3.24618000	1.29448600
H	-1.64319700	-2.73642600	0.86715300
H	-0.67674900	-0.91762400	2.19739500
H	-1.68682300	-0.27862900	3.52813300
F	-1.16479600	-1.72706300	-1.84602300

^{T}M8a

(U)M06/BSII SCF energy in solution: -1270.832498 a.u.

(U)M06/BSI SCF energy in solution: -1269.338524 a.u.

(U)M06/BSI SCF Gibbs energy in solution: -1269.04682 a.u.

O	0.43212900	3.67197800	-0.04382700
N	0.28974600	1.37222400	-0.22005800
C	-1.62441900	2.82979700	-0.83283700
C	-2.34281100	1.69295700	-1.45091100
C	-0.18585500	2.63573800	-0.32337300
C	-3.23619400	0.78544900	-0.68576000

H	-2.19149600	3.22362000	0.02755100
H	-2.16235100	1.46681400	-2.50308600
H	-3.80982700	0.15341200	-1.37442600
H	-3.95677200	1.33749000	-0.05553100
C	1.68515700	-2.22725400	-1.12970400
C	2.12523400	-0.16028400	-0.14896400
C	3.45072500	-0.54592100	0.17387600
C	3.85888000	-1.84354200	-0.21132500
C	2.98704500	-2.68286300	-0.86153900
H	0.94073700	-2.84993400	-1.62404300
C	1.60717600	1.13132100	0.18790100
C	4.28022300	0.36339000	0.86820300
H	4.87352800	-2.16565700	0.02223100
H	3.28002100	-3.68586200	-1.16064100
C	3.77271700	1.58976400	1.21687700
C	2.45670500	1.97858800	0.88753500
H	5.29851900	0.07255300	1.12244100
H	4.39499800	2.29598300	1.76484000
H	2.10824300	2.96091400	1.18060900
N	1.28856500	-1.01798200	-0.78783100
H	-1.54522200	3.66513100	-1.54426200
Cu	-0.61683200	-0.32380500	-0.80095200
N	-2.44462900	-0.13076500	0.19043800
O	-2.05697700	-1.78506700	2.49337500
C	-3.10639400	-1.44365200	0.33176600
C	-2.23069700	0.42749700	1.54021000
C	-2.28668800	-2.35441300	1.21798800
C	-1.40842100	-0.53523200	2.36507800
H	-3.19784200	-1.88633200	-0.66401200
H	-4.11156300	-1.29155000	0.76287600
H	-3.21275700	0.59089600	2.01819400
H	-1.71642600	1.39139500	1.47077900
H	-2.81686600	-3.30027900	1.37812300
H	-1.32295500	-2.58073000	0.72495600
H	-0.41272600	-0.67255500	1.89866100

H	-1.26290300	-0.13932600	3.37653700
F	-1.22660900	-1.74481100	-1.81539300

M7a

(U)M06/BSII SCF energy in solution: -1270.78409 a.u.

(U)M06/BSI SCF energy in solution: -1269.29148 a.u.

(U)M06/BSI SCF Gibbs energy in solution: -1269.002418 a.u.

O	0.60915700	3.46043100	0.74198700
N	0.23080600	1.24555500	0.30485700
C	-1.43799100	2.90379400	-0.35952000
C	-1.39533600	2.65126700	-1.83685600
C	-0.11453400	2.56260100	0.30041400
C	-2.24317300	1.85862000	-2.49157500
H	-2.25071500	2.31967700	0.08968800
H	-0.60124600	3.16596000	-2.38606400
H	-2.17621600	1.71535100	-3.56961700
H	-3.04631900	1.33191900	-1.97296400
C	1.61092100	-1.71885000	-1.86217800
C	2.04720400	-0.21635900	-0.14582500
C	3.36504000	-0.70066400	0.02273200
C	3.78091100	-1.73920900	-0.84225200
C	2.91407400	-2.24265700	-1.78275500
H	0.87579900	-2.10667000	-2.56587900
C	1.52184300	0.83349300	0.65842200
C	4.16119800	-0.14123800	1.04989700
H	4.79064000	-2.13746300	-0.74558400
H	3.20974800	-3.04546100	-2.45295700
C	3.63431100	0.84280800	1.85086500
C	2.32248500	1.33483500	1.66830000
H	5.17323500	-0.51381200	1.20122400
H	4.23726100	1.26526700	2.65288400
H	1.94926600	2.11890700	2.31912100
N	1.20802600	-0.74198000	-1.07632800
H	-1.61371200	3.96954700	-0.15878400
Cu	-0.70249600	-0.19290200	-0.58879900

N	-2.36817900	0.01344300	0.43303400
O	-2.48778300	-2.33661800	2.13468300
C	-3.27836000	-1.09660300	0.19166400
C	-2.03596400	0.01260300	1.85262900
C	-2.82002100	-2.43577300	0.76251300
C	-1.52110600	-1.33083900	2.34860000
H	-3.48601200	-1.18579200	-0.87770700
H	-4.21633600	-0.80791000	0.70162800
H	-2.97871800	0.22441700	2.38899700
H	-1.32600100	0.81418500	2.07878600
H	-3.62059500	-3.18011100	0.67952000
H	-1.95090100	-2.78507700	0.18481500
H	-0.57785000	-1.58296000	1.82403100
H	-1.31835400	-1.29603000	3.42523200
F	-1.35946300	-1.50737500	-1.63288300

TS2a

(U)M06/BSII SCF energy in solution: -1270.76391 a.u.

(U)M06/BSI SCF energy in solution: -1269.272652 a.u.

(U)M06/BSI SCF Gibbs energy in solution: -1268.985552 a.u.

O	-0.44930400	3.68435600	0.29506400
N	-0.25895600	1.43668100	-0.16065200
C	-2.41571700	2.55883100	-0.42930100
C	-2.91546800	1.26361700	-0.97787000
C	-0.94220100	2.61221000	-0.06126700
C	-3.58693700	0.35790600	-0.21512100
H	-2.96335500	2.81292400	0.49199000
H	-2.81979000	1.09212700	-2.05369900
H	-4.00827000	-0.54665500	-0.64986600
H	-3.87586100	0.59650800	0.80910400
C	1.92941400	-1.32245900	-2.05905200
C	1.86507000	0.34659100	-0.46001500
C	3.21841400	0.11917500	-0.09848400
C	3.91790700	-0.88774300	-0.80367100
C	3.28109000	-1.60767100	-1.78251700

H	1.38824800	-1.88577600	-2.82090100
C	1.07935800	1.32516600	0.23007900
C	3.78950400	0.86546100	0.95729800
H	4.95880600	-1.08588900	-0.54789700
H	3.79178400	-2.39398400	-2.33292500
C	3.02017400	1.78761700	1.62394500
C	1.67456000	2.01563600	1.27408500
H	4.82661100	0.68286000	1.23594800
H	3.44743400	2.35558100	2.44889800
H	1.09514800	2.74532300	1.83068000
N	1.25344400	-0.38466600	-1.42934600
H	-2.59494100	3.38586800	-1.13026800
Cu	-0.94120800	-0.14219100	-0.98621000
N	-1.86282100	-0.93350900	0.75756300
O	-0.25193800	-2.57647200	2.38691900
C	-2.01314100	-2.36079900	0.74425400
C	-1.43696700	-0.49477300	2.05554400
C	-0.66800100	-3.01451000	1.11703500
C	-0.10517100	-1.17747000	2.42701900
H	-2.31833500	-2.68171100	-0.25732700
H	-2.75088900	-2.68256000	1.49897300
H	-2.17671400	-0.79098200	2.81949400
H	-1.31277200	0.59740600	2.06474300
H	-0.77972600	-4.10335900	1.16095900
H	0.08476700	-2.76155700	0.34808400
H	0.68368600	-0.85119700	1.72810800
H	0.18674800	-0.90145800	3.44641600
F	-1.55895700	-1.49702900	-2.04122400

M5b

(U)M06/BSII SCF energy in solution: -3744.994772 a.u.

(U)M06/BSI SCF energy in solution: -3741.08218 a.u.

(U)M06/BSI SCF Gibbs energy in solution: -3740.796322 a.u.

O	2.58766300	3.28997000	0.26734400
N	1.56998000	1.35322300	-0.50628700

C	0.29280100	3.40160000	-0.34815800
C	-0.61444800	3.32763600	0.84624800
C	1.61171200	2.66502300	-0.18133000
C	-0.46343500	2.55201200	1.91981000
H	0.52396100	4.45804600	-0.55025800
H	-1.48545600	3.98728600	0.79575300
H	-1.18715700	2.57353300	2.73403900
H	0.38130000	1.87125600	2.04180900
C	1.06475200	-2.53024800	0.48766900
C	2.52356400	-0.83143400	-0.10647400
C	3.63300500	-1.72140600	-0.07052100
C	3.37676400	-3.07291200	0.25746500
C	2.09905100	-3.48298900	0.54038500
H	0.03674100	-2.81513000	0.72200200
C	2.70987900	0.55869900	-0.42564700
C	4.93082200	-1.23897100	-0.35284800
H	4.21082500	-3.77448100	0.28430900
H	1.87462600	-4.51456100	0.80087600
C	5.09683700	0.08947300	-0.65778600
C	4.00611800	0.97945400	-0.69403200
H	5.77414000	-1.92792400	-0.32184200
H	6.09093200	0.47546000	-0.88070700
H	4.18169600	2.02163700	-0.94237500
N	1.26285600	-1.26686500	0.17089700
H	-0.25080900	3.02383400	-1.23002000
Cu	-0.11664700	0.35379700	-0.33992600
N	-2.03812300	-0.16379800	-0.37743500
O	-4.53562300	-0.53440800	-1.76598300
C	-2.27399100	-1.26090900	-1.35351200
C	-2.99329400	0.94360500	-0.63804600
C	-3.72970000	-1.65510100	-1.45345700
C	-4.42250600	0.47038900	-0.77460100
H	-1.63005000	-2.11180400	-1.10276700
H	-1.93221000	-0.84998200	-2.31525600
H	-2.65143500	1.37939700	-1.58895200

H	-2.88073500	1.70124300	0.14619200
H	-3.86103600	-2.38571000	-2.25942500
H	-4.07364300	-2.12352500	-0.51415400
H	-4.80564600	0.09594600	0.19125300
H	-5.05901700	1.30659400	-1.08515900
Br	-2.31260500	-0.83763400	1.42687000

TS2b

(U)M06/BSII SCF energy in solution: -3744.973784 a.u.

(U)M06/BSI SCF energy in solution: -3741.060631 a.u.

(U)M06/BSI SCF Gibbs energy in solution: -3740.776475 a.u.

O	2.08065000	3.45989600	-0.58732900
N	1.25754900	1.28911300	-0.56587100
C	-0.18571200	3.12564800	-1.20567600
C	-1.08006900	3.53566200	-0.07211300
C	1.17565400	2.62529600	-0.75298900
C	-0.81858500	3.39250000	1.22539700
H	-0.01802200	3.99360100	-1.86101300
H	-2.02825500	3.98689600	-0.37728400
H	-1.52861000	3.71699500	1.98495800
H	0.11376400	2.95688700	1.58857300
C	1.27365300	-2.71875000	-0.83879700
C	2.41238700	-0.79263900	-0.24936800
C	3.56039200	-1.54483600	0.12472300
C	3.50086100	-2.94967000	-0.01953200
C	2.36433300	-3.54230900	-0.50466700
H	0.35531700	-3.16526500	-1.22504800
C	2.41562100	0.64468900	-0.14601400
C	4.70586900	-0.88407600	0.62401400
H	4.37130300	-3.54408500	0.25893100
H	2.28970000	-4.61953900	-0.63143100
C	4.68787700	0.48231000	0.74018500
C	3.56411100	1.24251900	0.36146900
H	5.57802900	-1.47190200	0.90836200
H	5.55992000	1.00596300	1.13045800

H	3.59388900	2.32127800	0.45534400
N	1.28948500	-1.40698500	-0.71754800
H	-0.70591100	2.37129800	-1.82071400
Cu	-0.33497700	0.16173000	-0.60905500
N	-2.10071200	-0.42133500	-0.78663200
O	-4.76488400	-1.30354100	-0.26065800
C	-2.50434500	-1.80534200	-0.94030000
C	-3.21736000	0.50166300	-0.67548000
C	-3.69710500	-2.21609500	-0.10106600
C	-4.38823700	-0.00199800	0.14267800
H	-1.64129300	-2.46098100	-0.78190300
H	-2.77412600	-1.88367800	-2.01052100
H	-3.54239500	0.62978300	-1.72615600
H	-2.85660400	1.47995000	-0.33454500
H	-4.05794600	-3.19820900	-0.42549600
H	-3.40547000	-2.28559200	0.96023200
H	-4.13143100	0.00924900	1.21519900
H	-5.25493500	0.64973500	-0.01127400
Br	-1.27054400	-0.37789700	1.58186800

M6b

(U)M06/BSII SCF energy in solution: -3744.980473 a.u.

(U)M06/BSI SCF energy in solution: -3741.064213 a.u.

(U)M06/BSI SCF Gibbs energy in solution: -3740.779175 a.u.

O	1.89605600	3.32044600	-1.08127200
N	1.04721700	1.20778800	-0.69743400
C	-0.44435900	3.01044400	-1.35393000
C	-1.15113700	3.60909300	-0.17177800
C	0.95213000	2.52083100	-1.02317300
C	-0.66882100	3.69622100	1.06520200
H	-0.34818700	3.77485100	-2.14012700
H	-2.14934300	4.00021600	-0.38504200
H	-1.24935000	4.14851800	1.86804400
H	0.32217800	3.32678100	1.33595900
C	1.28149400	-2.78257900	-0.96101100

C	2.28054000	-0.80750000	-0.31035700
C	3.43975700	-1.49489100	0.13968600
C	3.46515700	-2.90030800	-0.00390500
C	2.39361000	-3.54753900	-0.56212300
H	0.42207100	-3.28081500	-1.41212800
C	2.20645800	0.62402400	-0.20971800
C	4.50378700	-0.77019200	0.72395700
H	4.34431900	-3.45023800	0.33180300
H	2.38300100	-4.62637300	-0.69553700
C	4.39953800	0.59294200	0.85398800
C	3.26474900	1.28867000	0.39715500
H	5.38353700	-1.30976100	1.07280300
H	5.20626000	1.15760400	1.31904200
H	3.22099000	2.36723100	0.50307100
N	1.21186900	-1.47242500	-0.83200200
H	-1.06599100	2.20980600	-1.79052600
Cu	-0.52590300	0.08164700	-0.57340000
N	-2.12355400	-0.68329900	-1.08434200
O	-4.47888600	-1.60437300	0.25578700
C	-2.40636500	-2.08373600	-0.90153000
C	-3.30956900	0.13166900	-0.95624400
C	-3.29883600	-2.37374600	0.30394900
C	-4.19045800	-0.22429400	0.23777200
H	-1.46880200	-2.64662600	-0.83775500
H	-2.93817900	-2.41775500	-1.81185200
H	-3.89384600	-0.04855900	-1.87893500
H	-3.03278400	1.19379500	-0.94842500
H	-3.59419700	-3.42836500	0.30496500
H	-2.73198400	-2.15758800	1.22461500
H	-3.67004000	0.07657700	1.16255300
H	-5.14505500	0.30878500	0.17415200
Br	-0.89530100	0.01161300	1.72927500

^{S}M6b

(U)M06/BSII SCF energy in solution: -3744.999253 a.u.

(U)M06/BSI SCF energy in solution: -3741.084215 a.u.

(U)M06/BSI SCF Gibbs energy in solution: -3740.798366 a.u.

O	1.59544800	3.44098500	1.01201200
N	0.88625800	1.61374600	-0.21778500
C	-0.62693900	3.45434500	0.15173800
C	-1.36603700	2.90008700	-1.01718600
C	0.74491900	2.83655400	0.34995400
C	-2.53899100	2.24793200	-0.93076300
H	-1.20188000	3.30403800	1.07923200
H	-0.92230500	3.06577300	-2.00239200
H	-3.07117100	1.91801400	-1.81881100
H	-3.03448100	2.09403200	0.02973400
C	0.61443900	-2.21973700	-1.20094600
C	1.99486200	-0.46510900	-0.55488000
C	3.14105700	-1.30109500	-0.58443900
C	2.94910700	-2.65854100	-0.92652800
C	1.69367900	-3.11945900	-1.23200400
H	-0.39757700	-2.53389100	-1.45607500
C	2.08871600	0.91352700	-0.19478800
C	4.40550700	-0.74993500	-0.27680700
H	3.81212800	-3.32374000	-0.94599100
H	1.51705900	-4.15727800	-1.50238800
C	4.49163600	0.58204000	0.04258300
C	3.35364900	1.41215200	0.09131800
H	5.28610300	-1.39027100	-0.29987400
H	5.46069900	1.02287800	0.27243700
H	3.46742400	2.45651600	0.35656200
N	0.75981900	-0.95114800	-0.86933700
H	-0.48190500	4.53761400	0.03942200
Cu	-0.71859900	0.48708300	-0.60442300
N	-1.25272300	0.22607800	1.57049100
O	-0.98988800	-2.08953800	3.12541500
C	-2.41753200	-0.48548000	2.00882800
C	-0.20159000	0.12168900	2.54059700
C	-2.03956200	-1.96386400	2.19340700

C	0.14945700	-1.36590500	2.72284800
H	-3.21870100	-0.40249300	1.26466900
H	-2.76784700	-0.09890300	2.98152200
H	-0.53636500	0.50812700	3.51893400
H	0.68549000	0.68261900	2.21732200
H	-2.89545800	-2.52696600	2.58176100
H	-1.74999100	-2.38250600	1.21356000
H	0.55125500	-1.77295100	1.77723900
H	0.90783200	-1.47916300	3.50541000
Br	-2.47963300	-0.90041000	-1.45327400

^{S}TS2b

(U)M06/BSII SCF energy in solution: -3744.986266 a.u.

(U)M06/BSI SCF energy in solution: -3741.072005 a.u.

(U)M06/BSI SCF Gibbs energy in solution: -3740.786348 a.u.

O	1.50391900	3.77829700	0.08655400
N	0.79007600	1.60750500	-0.26996400
C	-0.83971100	3.38063100	-0.06797100
C	-1.84384600	2.30025400	-0.31177100
C	0.61567800	2.93967200	-0.08305100
C	-2.53713500	1.69468500	0.72311400
H	-1.00893300	3.84016800	0.91804500
H	-2.20967000	2.16044100	-1.33254600
H	-3.39845500	1.06800000	0.49906100
H	-2.46225300	2.09804900	1.73429700
C	0.73426200	-2.24657000	-1.17960800
C	2.01505600	-0.41744900	-0.52931600
C	3.19109200	-1.20677500	-0.46653200
C	3.06825200	-2.58047100	-0.77807000
C	1.84850100	-3.10235500	-1.13054800
H	-0.25352500	-2.60598400	-1.47110600
C	2.03182300	0.97608700	-0.20590700
C	4.40995300	-0.59790900	-0.08927800
H	3.95523800	-3.21203000	-0.73385300
H	1.72906700	-4.15466800	-1.37505300

C	4.42008200	0.74071700	0.21338600
C	3.25170900	1.52850500	0.16171200
H	5.31542300	-1.20134200	-0.04232300
H	5.35199100	1.22173400	0.50739900
H	3.30502200	2.58070600	0.41306700
N	0.81959900	-0.96455000	-0.88669800
H	-0.94712100	4.18490900	-0.80875600
Cu	-0.69236300	0.40688500	-0.66901300
N	-1.40401800	-0.01977000	1.44165900
O	-0.73756300	-1.91748300	3.35276800
C	-2.36492900	-1.02840700	1.79981300
C	-0.63525800	0.37544800	2.59169400
C	-1.55390300	-2.24912800	2.25190100
C	0.14885700	-0.86660400	3.03884600
H	-2.96744100	-1.29645400	0.92146400
H	-3.02034200	-0.70998200	2.62969500
H	-1.27234500	0.70355900	3.43192700
H	0.06366600	1.18093800	2.32617500
H	-2.22583100	-3.05409400	2.57078200
H	-0.93957600	-2.61083500	1.40664200
H	0.84409000	-1.17477000	2.23682100
H	0.72847600	-0.64228200	3.94153100
Br	-2.39549500	-0.77904300	-1.81462600

^{T}M7b

(U)M06/BSII SCF energy in solution: -3745.024972 a.u.

(U)M06/BSI SCF energy in solution: -3741.109903 a.u.

(U)M06/BSI SCF Gibbs energy in solution: -3740.821145 a.u.

O	1.32194300	3.47333700	-1.32323900
N	0.72050100	1.39878800	-0.51084000
C	-0.99419100	3.00877800	-1.28850500
C	-2.01445400	1.94138400	-1.39501600
C	0.46997100	2.62212700	-1.03811000
C	-2.86356500	1.51266900	-0.25377300
H	-1.25372200	3.70918500	-0.47597800

H	-2.15822800	1.44933000	-2.35710500
H	-3.67746300	0.86952100	-0.60762400
H	-3.31747700	2.36820600	0.27816500
C	1.32747400	-2.52373000	-0.27868000
C	2.24472600	-0.39291700	-0.09538500
C	3.51996100	-0.92477700	0.22577000
C	3.64398100	-2.33111800	0.27906700
C	2.55648000	-3.13039400	0.02743500
H	0.43551900	-3.11232100	-0.49201300
C	2.01946800	1.01600100	-0.17275500
C	4.58785200	-0.03965500	0.49581500
H	4.61231000	-2.76625200	0.52524100
H	2.62536100	-4.21428200	0.06435500
C	4.35907600	1.31297800	0.45113600
C	3.09465300	1.84348700	0.12400000
H	5.56632900	-0.44591900	0.74781100
H	5.16899100	2.00704000	0.67053800
H	2.96407800	2.91871500	0.09190800
N	1.18357600	-1.21256300	-0.33177800
H	-0.96816700	3.61138100	-2.20768400
Cu	-0.55560600	-0.15660600	-0.39242900
N	-2.07531500	0.71257400	0.73637500
O	-1.49262400	-0.24696500	3.37865500
C	-2.92651000	-0.27998900	1.42862000
C	-1.45672800	1.56627600	1.77885100
C	-2.10003700	-1.07916300	2.40983900
C	-0.66921500	0.71379200	2.74686700
H	-3.37217700	-0.94396000	0.68128800
H	-3.73313100	0.25649700	1.95814000
H	-2.26180200	2.09906200	2.31386100
H	-0.79816200	2.30631200	1.31532300
H	-2.73579700	-1.79333200	2.94501200
H	-1.32060600	-1.65292100	1.86999600
H	0.15930600	0.20751300	2.21590500
H	-0.23824900	1.34111800	3.53498300

Br	-1.88039700	-1.85115400	-1.43035400

1a’

(U)M06/BSII SCF energy in solution: -747.258608 a.u.

(U)M06/BSI SCF energy in solution: -747.149936 a.u.

(U)M06/BSI SCF Gibbs energy in solution: -747.055756 a.u.

N	-0.53073900	0.44868400	0.00000000
O	0.82692700	-2.01632600	0.00000000
C	0.17863600	-0.01662100	1.19526500
C	0.17863600	-0.01662100	-1.19526500
C	0.17863600	-1.53365200	1.15973400
C	0.17863600	-1.53365200	-1.15973400
H	-0.34788200	0.33747300	2.08953100
H	1.21829900	0.35600800	1.22100500
H	1.21829900	0.35600800	-1.22100500
H	-0.34788200	0.33747300	-2.08953100
H	0.72478900	-1.92326600	2.02620100
H	-0.85817600	-1.91094900	1.20044600
H	-0.85817600	-1.91094900	-1.20044600
H	0.72478900	-1.92326600	-2.02620100
Cl	-0.50950400	2.22791600	0.00000000

M5a

(U)M06/BSII SCF energy in solution: -1344.118207 a.u.

(U)M06/BSI SCF energy in solution: -1342.716053 a.u.

(U)M06/BSI SCF Gibbs energy in solution: -1342.54784 a.u.

O	-0.69030900	3.46647900	-0.43137800
N	-0.39084800	1.21791100	-0.00835300
C	-2.63956000	2.15335800	-0.13541500
C	-3.14147000	0.74919300	-0.04641100
C	-1.12971100	2.33514200	-0.21238000
C	-3.22160100	0.07237400	1.12055800
H	-2.96218800	2.72295400	0.74895000
H	-3.51191800	0.27831200	-0.96107900
H	-3.65560400	-0.92302900	1.18091000

H	-2.90310400	0.53757200	2.05607200
C	1.23616500	-2.38683300	0.01050200
C	1.59488100	-0.08477200	0.02736700
C	3.00033400	-0.26278000	0.05164900
C	3.48983400	-1.58780400	0.05205700
C	2.61611100	-2.64658900	0.03194900
H	0.50520700	-3.19423100	-0.01211200
C	1.00428200	1.21457000	0.01777700
C	3.83497700	0.87757400	0.07787000
H	4.56706500	-1.75106300	0.06793200
H	2.96648600	-3.67509200	0.03107600
C	3.26146500	2.12381600	0.08217300
C	1.86251100	2.30424800	0.05291700
H	4.91563300	0.74575600	0.09806200
H	3.89323900	3.01042700	0.10557500
H	1.45299300	3.30715400	0.05169100
N	0.75363700	-1.15839400	0.01178800
H	-3.06732900	2.66452300	-1.00623500
Cu	-1.17788400	-0.56565900	0.01710400
Cl	-2.09813000	-2.62568200	-0.36550300

^{T}M6c

(U)M06/BSII SCF energy in solution: -1631.177894 a.u.

(U)M06/BSI SCF energy in solution: -1629.696243 a.u.

(U)M06/BSI SCF Gibbs energy in solution: -1629.409799 a.u.

O	0.47632900	3.39825300	1.43648200
N	0.32445600	1.64884600	-0.06766800
C	-1.60658900	3.05131700	0.33359900
C	-2.06581100	2.61384300	-1.01398600
C	-0.15153600	2.72116900	0.61589700
C	-3.05226300	1.73006600	-1.22841300
H	-2.21844800	2.58079000	1.11999800
H	-1.55538500	3.06090100	-1.87098300
H	-3.37055400	1.46429600	-2.23349700
H	-3.60679700	1.28452300	-0.39994000

C	1.04625200	-1.97410200	-1.53046700
C	1.91003100	-0.08447900	-0.48672500
C	3.20908100	-0.65220700	-0.44005300
C	3.36954700	-1.95453000	-0.96408200
C	2.29623000	-2.61695900	-1.50434500
H	0.17083100	-2.45313400	-1.96828600
C	1.64484800	1.21388700	0.04698600
C	4.26909500	0.09342600	0.12292900
H	4.35570500	-2.41717300	-0.93265000
H	2.39259700	-3.61784900	-1.91700200
C	4.01243200	1.34932700	0.61257800
C	2.71945100	1.91065200	0.58557100
H	5.26642600	-0.34237000	0.15899100
H	4.82059600	1.93812100	1.04394000
H	2.56140800	2.90156100	0.99352800
N	0.86506900	-0.76433700	-1.03731400
H	-1.71658800	4.13781700	0.45076700
Cu	-0.90438200	0.29372100	-0.83168600
N	-1.59194400	-0.40014000	1.16456900
O	-1.04885500	-2.72598100	2.62368900
C	-2.61422900	-1.38694100	1.35455300
C	-0.70664600	-0.35679500	2.29291500
C	-1.93773000	-2.75643700	1.53015800
C	-0.05656300	-1.74194400	2.45003800
H	-3.28657500	-1.41174800	0.48875500
H	-3.19098600	-1.17179800	2.27056600
H	-1.26968300	-0.14180500	3.21745300
H	0.06661700	0.41037400	2.15894000
H	-2.69100200	-3.52467300	1.73612200
H	-1.40152400	-3.02025200	0.60161700
H	0.56007200	-1.96684400	1.56042800
H	0.58563300	-1.75547700	3.33752400
Cl	-2.15068900	-1.18809800	-2.07404700

^{T}TS2c

(U)M06/BSII SCF energy in solution: -1631.148317 a.u.

(U)M06/BSI SCF energy in solution: -1629.6696 a.u.

(U)M06/BSI SCF Gibbs energy in solution: -1629.383714 a.u.

O	0.84378200	3.77666000	0.47616600
N	0.55005200	1.57128400	-0.16162000
C	-1.29236600	3.10764900	-0.35539400
C	-2.05450200	1.92138800	-0.88004300
C	0.15383100	2.85135400	0.03175900
C	-3.10060600	1.36468000	-0.14256600
H	-1.78641000	3.52193200	0.53811100
H	-2.11730200	1.79628400	-1.96598800
H	-3.82441100	0.71895800	-0.63639200
H	-3.45528900	1.88014300	0.75159200
C	1.17640500	-2.17700600	-1.26891900
C	2.04811200	-0.26993200	-0.26986600
C	3.29214700	-0.90749900	-0.03287800
C	3.42576300	-2.24827700	-0.45999300
C	2.37692500	-2.88399500	-1.07646500
H	0.31696000	-2.63920500	-1.75653100
C	1.81883200	1.08709800	0.13023500
C	4.32496400	-0.18970000	0.61303400
H	4.37183800	-2.76277800	-0.29139900
H	2.45475100	-3.91414300	-1.41445700
C	4.09521700	1.10719600	0.99937300
C	2.86063300	1.74874200	0.76730000
H	5.28140700	-0.67866400	0.79329800
H	4.88203200	1.66850200	1.50164000
H	2.71927300	2.77470700	1.08716100
N	1.02114900	-0.92697400	-0.88052400
H	-1.28269900	3.92376300	-1.09366300
Cu	-0.65566700	0.30071200	-1.01772600
N	-2.53921100	-0.16438600	1.12957200
O	-1.30716800	-2.23588900	2.56991600
C	-3.08886800	-1.48744200	1.11613300
C	-1.68650500	0.12671900	2.24305800

C	-2.00901900	-2.53692700	1.38406100
C	-0.66241600	-0.98584100	2.46516200
H	-3.58841500	-1.67026800	0.15646300
H	-3.84186900	-1.54876500	1.92357300
H	-2.32061500	0.20131300	3.14574000
H	-1.19248200	1.09597900	2.08717400
H	-2.46808200	-3.52319600	1.51211100
H	-1.31437000	-2.57936500	0.52716400
H	0.05944300	-1.00017400	1.62952700
H	-0.11598200	-0.81383200	3.39874100
Cl	-1.96185800	-1.09569000	-2.29084800

^{T}M7c

(U)M06/BSII SCF energy in solution: -1631.202089 a.u.

(U)M06/BSI SCF energy in solution: -1629.721724 a.u.

(U)M06/BSI SCF Gibbs energy in solution: -1629.432272 a.u.

O	0.73724500	3.64610500	-0.66003700
N	0.40392900	1.38695200	-0.34087800
C	-1.45848900	2.86550500	-1.04440000
C	-2.27814600	1.70601400	-1.46243300
C	0.01095400	2.64404000	-0.66180800
C	-3.15361600	0.95583500	-0.52653500
H	-1.91593400	3.36582900	-0.17350100
H	-2.23186100	1.37640400	-2.50066100
H	-3.79746800	0.26635400	-1.08388900
H	-3.80432300	1.62361300	0.06701600
C	1.62288400	-2.36675300	-0.82163800
C	2.16206200	-0.21624500	-0.11404500
C	3.48282700	-0.61144600	0.21873600
C	3.83728600	-1.95860900	-0.01639500
C	2.91711400	-2.83370900	-0.53804100
H	0.85582100	-3.02723400	-1.22518500
C	1.71514500	1.12873900	0.06750200
C	4.36541700	0.33920100	0.77848600
H	4.84733000	-2.28746900	0.22696000

H	3.16383500	-3.87504700	-0.72734100
C	3.91780100	1.61891300	0.99286800
C	2.61102900	2.01838600	0.64570800
H	5.37786500	0.03679800	1.04222200
H	4.58268800	2.35715800	1.43877600
H	2.30802300	3.04367600	0.82182600
N	1.26682200	-1.11253200	-0.61446800
H	-1.42346900	3.62294000	-1.84062400
Cu	-0.60985500	-0.33439000	-0.61735900
N	-2.35030400	0.12508200	0.42434800
O	-1.89297600	-1.17324100	2.94425300
C	-3.10539900	-1.07541700	0.85022500
C	-1.98744100	0.86920800	1.65328900
C	-2.27368600	-1.90809900	1.79679400
C	-1.17305100	-0.01578300	2.56948700
H	-3.36948700	-1.66180600	-0.03554700
H	-4.03280600	-0.74368000	1.34865400
H	-2.91779400	1.18008500	2.15915100
H	-1.41681500	1.76542200	1.39540900
H	-2.85017800	-2.77390400	2.14143700
H	-1.37218500	-2.28283600	1.27389100
H	-0.22732700	-0.30336100	2.07114800
H	-0.92291000	0.52408800	3.48958800
Cl	-1.55533100	-2.00281200	-1.93349600

^{S}M6c

(U)M06/BSII SCF energy in solution: -1629.409147 a.u.

(U)M06/BSI SCF energy in solution: -1629.696024 a.u.

(U)M06/BSI SCF Gibbs energy in solution: -1629.409147 a.u.

O	-1.02359100	3.76659000	0.24473600
N	-0.59601500	1.53262800	-0.15608600
C	-2.87893400	2.28696800	0.18059400
C	-3.29775500	0.97019800	-0.37882000
C	-1.39523100	2.60050500	0.08002800
C	-3.50667500	-0.11689000	0.39121400

H	-3.47918400	0.90946200	-1.45588100
H	-3.86647300	-1.05670600	-0.02070300
H	-3.38193300	-0.06259800	1.47439600
C	1.23389900	-1.53664400	-1.87279100
C	1.45569900	0.52985400	-0.83108700
C	2.86676100	0.49620300	-0.96892100
C	3.43224700	-0.62917300	-1.61021000
C	2.62322200	-1.63821400	-2.06817900
H	0.55424100	-2.31996100	-2.20776900
C	0.79346900	1.63553800	-0.21650500
C	3.63402700	1.56690100	-0.45689900
H	4.51384000	-0.67820600	-1.73331400
H	3.03084100	-2.51307300	-2.56783500
C	2.99294000	2.61440900	0.15595300
C	1.58894400	2.66117100	0.27685500
H	4.71831200	1.53840000	-0.55368200
H	3.57314400	3.44184900	0.56168700
H	1.12441300	3.51257700	0.75918500
N	0.68029000	-0.49924400	-1.27612600
Cu	-1.25827000	-0.28387900	-0.58942400
N	-0.71816000	-1.23889900	1.38405100
O	1.56290700	-1.71168800	2.95994200
C	-0.15844700	-2.55534200	1.48793400
C	-0.47911800	-0.47353800	2.57405400
C	1.34446400	-2.44870200	1.77801900
C	1.02987100	-0.41124100	2.85477700
H	-0.33350200	-3.11455800	0.55941600
H	-0.62552400	-3.10090000	2.32708600
H	-0.96320700	-0.96021000	3.43954800
H	-0.88411800	0.54167400	2.46370400
H	1.77302500	-3.44627300	1.92515700
H	1.85386300	-1.96750400	0.92286400
H	1.53779100	0.14937700	2.04856700
H	1.21378500	0.10007200	3.80613800
Cl	-2.05144100	-2.14698500	-1.71499300

H	-3.41683900	3.10886100	-0.30825100
H	-3.13174500	2.33632800	1.25147800

^{S}TS2c

(U)M06/BSII SCF energy in solution: -1631.16283 a.u.

(U)M06/BSI SCF energy in solution: -1629.682995 a.u.

(U)M06/BSI SCF Gibbs energy in solution: -1629.395859 a.u.

O	0.74793400	3.77214400	0.71129300
N	0.42568000	1.63396300	-0.10601000
C	-1.44390300	3.16218000	0.00244500
C	-2.22922200	2.02164400	-0.56112700
C	0.03322000	2.88696100	0.23630900
C	-2.99553800	1.19069400	0.24063300
H	-2.38781100	1.98812800	-1.64255400
H	-3.71126800	0.51041800	-0.21706500
H	-3.14985000	1.44338200	1.29115000
C	1.04608900	-2.01377100	-1.51712600
C	1.94218500	-0.17435700	-0.41038500
C	3.18873300	-0.82786400	-0.24009900
C	3.30699300	-2.14173100	-0.74753500
C	2.24394000	-2.73742000	-1.37945200
H	0.17769600	-2.44031300	-2.02055500
C	1.71490000	1.14308100	0.09871300
C	4.23431000	-0.15328200	0.43009800
H	4.25268700	-2.66947100	-0.62548500
H	2.31049800	-3.74737000	-1.77545300
C	4.01238100	1.11399200	0.90774700
C	2.77004300	1.76451700	0.75331800
H	5.19335100	-0.65265800	0.56059600
H	4.80969600	1.64295200	1.42795100
H	2.63262600	2.76352700	1.15070400
N	0.90843900	-0.78870600	-1.05120800
Cu	-0.78249700	0.36200100	-0.94357100
N	-1.78065200	-0.47491100	0.91969100
O	-1.19935900	-2.54137700	2.68126200

C	-2.65896000	-1.61212000	0.98931600
C	-1.25546600	-0.16869800	2.22511400
C	-1.79209500	-2.79934700	1.42796700
C	-0.40518900	-1.37664800	2.64373600
H	-3.08863600	-1.81654300	-0.00018200
H	-3.46705900	-1.47274800	1.72905700
H	-2.05198500	-0.03082100	2.97758500
H	-0.62983500	0.73404100	2.18422300
H	-2.40861500	-3.69916100	1.53381800
H	-1.01617200	-2.98595300	0.66287100
H	0.43496500	-1.50669700	1.93710200
H	0.00162400	-1.22146700	3.64946400
Cl	-2.08099900	-0.76184600	-2.46224300
H	-1.51140600	4.04163300	-0.65258700
H	-1.85852100	3.46691500	0.97528100

M7c

(U)M06/BSII SCF energy in solution: -1631.222094 a.u.

(U)M06/BSI SCF energy in solution: -1629.742442 a.u.

(U)M06/BSI SCF Gibbs energy in solution: -1629.449787 a.u.

O	0.17047400	3.69574700	-0.03244900
N	0.30548100	1.44949800	-0.54831700
C	-1.70641000	2.59319300	-1.07009800
C	-2.21065600	1.18562300	-1.20290400
C	-0.31509300	2.66184600	-0.48176700
C	-2.97459400	0.54511400	-0.06229500
H	-2.38591800	3.18404900	-0.43661700
H	-2.61677200	0.94824800	-2.18862300
H	-3.65950200	-0.19756500	-0.48705600
H	-3.59003900	1.31659700	0.44247800
C	1.48034400	-2.29078500	-1.00701200
C	2.03301600	-0.15898300	-0.26516900
C	3.32497100	-0.57868800	0.13399900
C	3.65562800	-1.93746700	-0.07360400
C	2.74135100	-2.79197900	-0.63821700

H	0.72495700	-2.93387100	-1.45969300
C	1.60124900	1.19067100	-0.09204000
C	4.20246600	0.36406800	0.71698300
H	4.64205300	-2.29147300	0.22494000
H	2.97127700	-3.84089000	-0.80573800
C	3.77747800	1.65816600	0.88470200
C	2.48984900	2.08155200	0.49106300
H	5.19699200	0.04619900	1.02659900
H	4.44478000	2.39225100	1.33363500
H	2.19549700	3.11400300	0.63896100
N	1.14581700	-1.02851600	-0.82831500
H	-1.67177700	3.09112300	-2.04906400
Cu	-0.61660200	-0.06929400	-1.18006900
N	-2.10152700	-0.14310400	0.89447100
O	-1.27576900	-1.27193800	3.36017000
C	-2.68326500	-1.38565800	1.39420900
C	-1.69889400	0.69659000	2.01897300
C	-1.67466600	-2.08839000	2.27454300
C	-0.72007100	-0.05709800	2.89210900
H	-2.93435800	-2.02917900	0.54022400
H	-3.61164000	-1.19106700	1.96890000
H	-2.58392700	0.99297000	2.61851900
H	-1.22082000	1.61643900	1.65608900
H	-2.10202100	-3.00298100	2.70241700
H	-0.79104700	-2.36901700	1.66807200
H	0.20744200	-0.26345100	2.32317300
H	-0.45473900	0.53502100	3.77577500
Cl	-1.73066100	-1.68059800	-2.23520500

TS3c

(U)M06/BSII SCF energy in solution: -1631.18832 a.u.

(U)M06/BSI SCF energy in solution: -1629.716342 a.u.

(U)M06/BSI SCF Gibbs energy in solution: -1629.42687 a.u.

O	-0.22150800	3.32226400	1.18388900
N	0.21971100	1.52614300	-0.23542000

C	-1.91658500	2.55463200	-0.31592800
C	-2.77537300	1.35881900	-0.43431200
C	-0.52791800	2.49513800	0.31087900
C	-3.05300400	0.32762300	0.60993600
H	-2.56127500	3.24757900	0.26316400
H	-3.64444800	1.55639100	-1.06141100
C	1.70535500	-1.95424200	-1.51599700
C	2.08889300	0.06128500	-0.43646000
C	3.46180500	-0.25356400	-0.25345200
C	3.92106800	-1.49386800	-0.75344000
C	3.05079800	-2.34484200	-1.38541000
H	0.98568100	-2.61024900	-2.00811000
C	1.55342000	1.30662100	0.05295300
C	4.30790200	0.66129100	0.41577100
H	4.97122700	-1.75657200	-0.62556000
H	3.37646400	-3.30465300	-1.77877000
C	3.78478700	1.84208900	0.88115700
C	2.42496300	2.16790200	0.70842700
H	5.35867900	0.40911400	0.55410600
H	4.42817900	2.55260700	1.39883000
H	2.04606000	3.11227900	1.08566000
N	1.24086900	-0.80575300	-1.06393800
H	-1.83238300	3.02753700	-1.30614000
Cu	-0.76096300	-0.06926900	-0.95084800
N	-1.94413400	-0.56142400	0.95235800
O	-0.59053800	-2.49318400	2.54456600
C	-2.39817100	-1.95776700	1.03380200
C	-1.27971100	-0.19863500	2.21207200
C	-1.22475800	-2.85971800	1.33380000
C	-0.13825500	-1.15248200	2.48289800
H	-2.84769900	-2.24186500	0.07320900
H	-3.16487500	-2.06834100	1.82429800
H	-2.00697500	-0.24603700	3.04522900
H	-0.89934000	0.82938800	2.15281700
H	-1.56230500	-3.89634300	1.44731600

H	-0.49960900	-2.82070900	0.49902400
H	0.63374500	-1.05198100	1.69825200
H	0.32376800	-0.92604300	3.45055400
Cl	-2.45601500	-0.20839800	-2.46942600
H	-3.40835500	0.90253600	1.49160400
H	-3.90641000	-0.27016800	0.27194800

M8c

(U)M06/BSII SCF energy in solution: -1631.2234 a.u.

(U)M06/BSI SCF energy in solution: -1629.75313 a.u.

(U)M06/BSI SCF Gibbs energy in solution: -1629.45908 a.u.

O	-0.33169800	3.27889000	-1.43616300
N	-0.45954400	1.30404100	-0.21623500
C	1.57833600	2.70718400	-0.23125900
C	2.43755000	1.91131300	0.74276800
C	0.13961300	2.40680100	-0.67782100
C	3.17832800	0.66322600	0.29675100
H	2.17883000	2.80149600	-1.15141700
H	3.23684400	2.59181200	1.06174800
C	-1.79828500	-2.18562800	1.28816100
C	-2.28066800	-0.21279900	0.14629600
C	-3.65561100	-0.56828100	0.06478300
C	-4.05962500	-1.79185000	0.64582000
C	-3.13976300	-2.60104000	1.26082200
H	-1.03719400	-2.80830700	1.75837900
C	-1.80227800	1.03290700	-0.41290700
C	-4.56984000	0.29284000	-0.58350900
H	-5.11107100	-2.07458100	0.59422400
H	-3.42267900	-3.54673700	1.71621600
C	-4.10796000	1.46639600	-1.12327200
C	-2.75170400	1.83578900	-1.04441700
H	-5.61926400	0.00654400	-0.64227100
H	-4.79989100	2.14026300	-1.62787100
H	-2.42769500	2.77363500	-1.47964100
N	-1.38580200	-1.04684600	0.75940300

H	1.51823700	3.73183700	0.16304900
Cu	0.49562900	-0.41195800	0.50823400
N	2.39146500	-0.47673100	-0.20620000
O	2.17606500	-2.81914300	-1.84879400
C	3.07110300	-1.74766900	0.12805300
C	2.26252400	-0.40849400	-1.68167300
C	2.30547000	-2.91824600	-0.44363000
C	1.52422900	-1.61678300	-2.21078800
H	3.13053000	-1.83480000	1.22058500
H	4.09824700	-1.73631000	-0.28053400
H	3.27300600	-0.36698400	-2.12681700
H	1.72516000	0.50654400	-1.95948500
H	2.83601800	-3.85438400	-0.23475300
H	1.30359400	-2.97304100	0.02561800
H	0.48186700	-1.62009800	-1.83707800
H	1.49392600	-1.58524300	-3.30592400
Cl	1.56058400	1.57425100	2.33080400
H	3.90264000	0.98114700	-0.47464100
H	3.76941100	0.31647800	1.15397300

M9c

(U)M06/BSII SCF energy in solution: -1930.993496 a.u.

(U)M06/BSI SCF energy in solution: -1929.401671 a.u.

(U)M06/BSI SCF Gibbs energy in solution: -1928.972306 a.u.

O	-2.63483700	-0.99343500	0.25570700
N	2.33625600	-1.61882900	-1.13067300
N	-0.78086400	0.28316800	-0.12298200
C	-0.48557200	-2.03511300	0.50693200
O	5.17964200	-1.67207100	-1.53335500
C	0.09418500	-2.75251800	-0.70750100
C	-1.41360800	-0.87478300	0.20624600
C	1.00179600	-1.97912200	-1.65935200
C	3.01849400	-0.81235200	-2.17558400
C	3.15530900	-2.82918400	-0.87495100
C	4.43580800	-0.49206600	-1.76376200

C	4.57318400	-2.44407800	-0.51600700
H	0.24009500	0.28783800	-0.09818800
H	0.35805900	-1.69909900	1.13524400
H	0.58782800	-3.66545600	-0.35898300
H	1.14970000	-2.59648300	-2.56136400
H	0.50620900	-1.05103500	-1.97752900
H	2.44459800	0.11082600	-2.33177500
H	3.02822800	-1.38447500	-3.12010800
H	3.15184800	-3.46200800	-1.78053600
H	2.71650100	-3.39929200	-0.04710800
H	4.94146800	0.06131700	-2.56322700
H	4.43685700	0.13696400	-0.85092100
H	4.57803700	-1.87927200	0.43719900
H	5.18214800	-3.34532200	-0.38543000
C	1.83463800	2.97848900	-1.44800000
C	-0.36037500	2.45620100	-1.01747700
C	-0.80751600	3.73197300	-1.45244500
C	0.17766100	4.64191500	-1.90037900
C	1.49800400	4.27198000	-1.89656100
H	2.88113900	2.66377800	-1.43726400
C	-1.31846400	1.50372700	-0.54342000
C	-2.18776000	4.04088500	-1.42488200
H	-0.13146600	5.62983700	-2.24144100
H	2.27946000	4.94969800	-2.23170800
C	-3.08161300	3.10551900	-0.97037300
C	-2.65635700	1.83643300	-0.52392400
H	-2.51761400	5.02184500	-1.76519300
H	-4.14646500	3.33158000	-0.94204100
H	-3.38668400	1.12331700	-0.15845500
N	0.94985800	2.09806200	-1.02943800
H	-1.05912700	-2.76044200	1.09459000
Cu	2.33983000	-0.49324300	0.54954500
H	-4.47185500	-2.03100500	0.98617000
I	2.40021700	0.81632600	2.66606000
N	-5.48427100	-2.15232200	1.07792700

O	-6.35173900	0.43983700	0.18255900
C	-5.97501700	-1.12563600	1.99791900
C	-6.07271400	-1.93277700	-0.24382000
C	-5.76480700	0.27778200	1.46617600
C	-5.86359900	-0.51852400	-0.74807400
H	-5.47624900	-1.23558300	2.97049700
H	-7.05169200	-1.29665900	2.15733500
H	-7.15300400	-2.13764700	-0.17439800
H	-5.64595800	-2.64990300	-0.95848500
H	-6.22766800	1.03094000	2.11729000
H	-4.68058200	0.49035800	1.41617100
H	-4.78710300	-0.35027300	-0.93174900
H	-6.40276100	-0.34012500	-1.68772400
Cl	-1.28893800	-3.36900900	-1.74215100

Cl-pro

(U)M06/BSII SCF energy in solution: -1434.394312 a.u.

(U)M06/BSI SCF energy in solution: -1434.109352 a.u.

(U)M06/BSI SCF Gibbs energy in solution: -1433.804212 a.u.

O	1.73463100	-0.36791600	3.40023400
N	1.23281500	0.48307800	1.34046400
C	-0.40994700	0.66387200	3.08798800
C	-1.39264100	-0.43969600	2.78950700
C	0.97401600	0.24240800	2.65510400
C	-1.06875400	-1.73578400	2.57093800
H	0.56637900	1.04626800	0.76878100
H	-0.40607500	0.87127600	4.16704700
H	-2.44598000	-0.15607000	2.82587700
H	-1.85027900	-2.48568800	2.44764000
H	-0.04157100	-2.09921200	2.63704800
H	-0.73067600	1.58393500	2.57910000
Cu	-1.07541700	-0.88553300	0.52737800
I	-3.28202900	-0.18058600	-0.72150400
C	0.30152800	-2.33923700	-1.53832000
C	1.90063400	-1.15378000	-0.35223000

C	2.94601000	-1.77828500	-1.09193800
C	2.59109300	-2.71100900	-2.09189900
C	1.26995100	-2.98784000	-2.32369800
H	-0.75681900	-2.55221100	-1.69570900
C	2.25627800	-0.18787600	0.63530700
C	4.29652800	-1.46494300	-0.81269300
H	3.38100500	-3.19547300	-2.66502800
H	0.95601800	-3.69392100	-3.08767400
C	4.60809000	-0.55435700	0.16326200
C	3.58150400	0.09152500	0.87942100
H	5.07658500	-1.96286100	-1.38732100
H	5.64607300	-0.31271700	0.38213700
H	3.82491700	0.83592000	1.63572500
N	0.58742700	-1.46613700	-0.58542400
N	-0.33477300	2.24186900	-0.37599100
O	1.78681500	3.72049200	-1.51126900
C	-0.00144700	3.58190900	0.10922400
C	-0.00518000	2.12150800	-1.79713200
C	1.46965600	3.83870300	-0.13495200
C	1.46354500	2.42766700	-1.99630900
H	-0.21710300	3.64709800	1.18449700
H	-0.59208400	4.36247400	-0.40571200
H	-0.60315700	2.82047300	-2.41203400
H	-0.22537100	1.09994800	-2.13737100
H	1.74420900	4.85597900	0.16838400
H	2.07794000	3.12559000	0.45358800
H	2.08359400	1.66841300	-1.48011000
H	1.72292600	2.41451400	-3.06149900
H	-1.33094300	2.05679700	-0.24619300

Part 4: General procedure and characteristic data for products 2a-8y

The alkenes used in this paper:

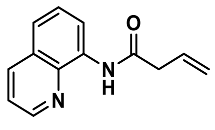

2a

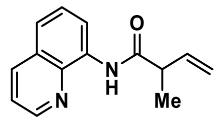

2b

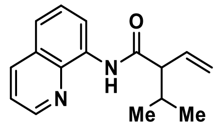

2c

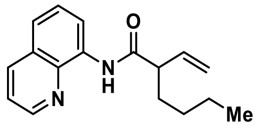

2d

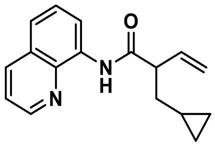

2e

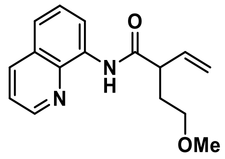

2f

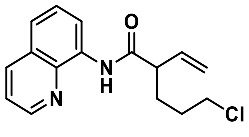

2g

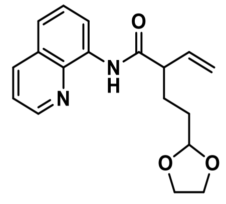

2h

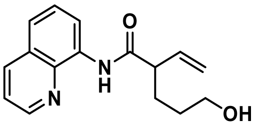

2i

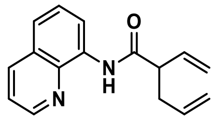

2j

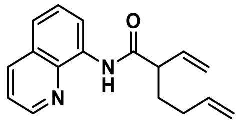

2k

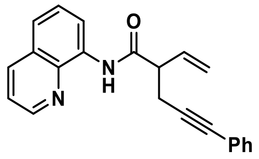

2l

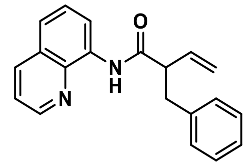

2m

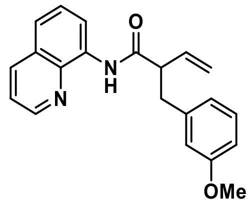

2n

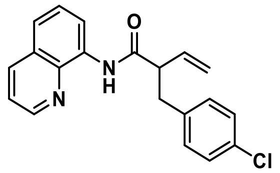

2o

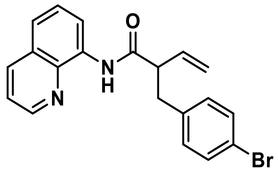

2p

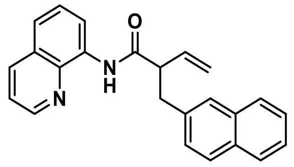

2q

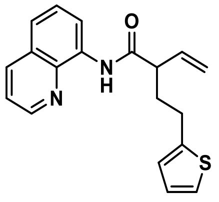

2r

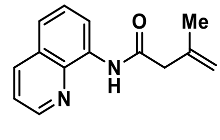

2s

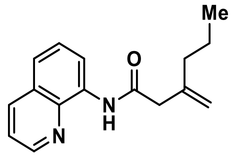

2t

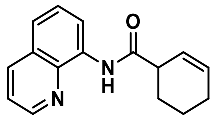

2u

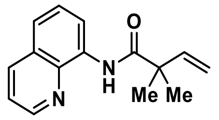

2v

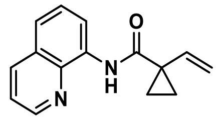

18

2a-2d, **2f**, **2g**, **2j-2p**, **2s**, **2u**, **2v** are known compounds according to ref. 1; **2e**, **2h**, **2i**, **2q**, **2r**, **2t** are known compounds according to ref. 2; **18** is a known compound according to ref. 3.

The dialkylamines used in this paper:

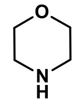

a

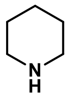

b

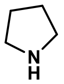

c

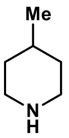

d

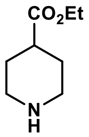

e

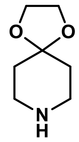

f

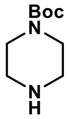

g

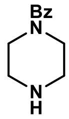

h

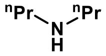

i

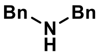

j

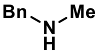

k

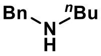

l

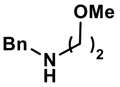

m

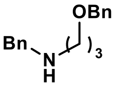

n

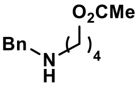

o

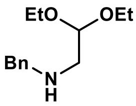

p

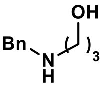

q

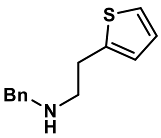

r

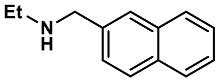

s

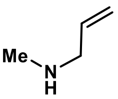

t

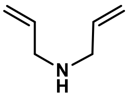

u

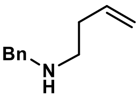

v

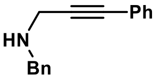

w

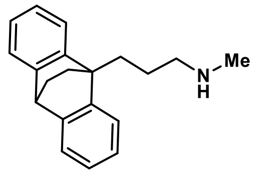

x (Maprotiline)

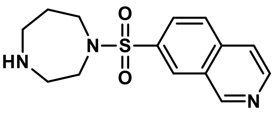

y (Fasudil)

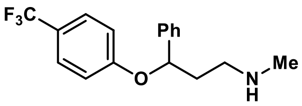

z (Fluoxetine)

The *N*-bromodialkylamines **1a-1z** used in this manuscript were prepared from the corresponding dialkylamines **a-z**. **a-k**, **t**, **u**, **x-z** are commercially available dialkylamines; **l**, **n**, **p** are known dialkylamines according to ref. 2; **m** is a known dialkylamine according to ref. 4; **o** is a known dialkylamine according to ref. 5; **q** is a known dialkylamine according to ref. 6; **r** is a known dialkylamine according to ref. 7; **s** is a known dialkylamine according to ref. 8; **v** is a known dialkylamine according to ref. 9; **w** is a known dialkylamine according to ref. 10.

The general procedure A:

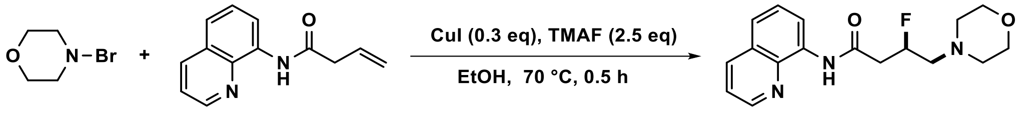

Preparation of *N*-bromodialkylamine compound: To a dry round bottom flask were added morpholine (174 μl, 2.0 mmol), *N*-bromosuccinimide (NBS, 356 mg, 2.0 mmol) and anhydrous EtOH (10 mL). The mixture was stirred at 0 °C avoiding light for 30 min. The solution of **1a** (0.20 M) was carried forward to the next step without further purification.

To a dry Schlenk flask were added CuI (11.4 mg, 0.06 mmol, 0.3 equiv), **2a** (42.4 mg, 0.20 mmol, 1.0 equiv), tetramethylammonium fluoride (TMAF, 46.6 mg, 0.50 mmol, 2.5 equiv) and anhydrous EtOH (3.0 mL). The mixture was degassed three times with argon. Then pre-prepared *N*-bromodialkylamine **1a** (2.0 mL, 0.40 mmol, 0.20 M in EtOH, 2.0 equiv) was added by syringe pump dropwise into the reaction mixture for half an hour at 70 °C (oil bath). This process should avoid exposure to light. When the addition was completed, the mixture was cooled to ambient temperature. The reaction mixture was filtered by Celite, and the filtrate was concentrated *in vacuo*. Further purification by a flash column chromatography using eluents (PE/EA = 1:1) afforded the desired product **3a** as yellow oil (50.7 mg, 0.16 mmol, 80%). **^{1}H NMR** (600 MHz, $CDCl_3$) δ 9.99 (s, 1H), 8.81 (dd, J = 4.2, 1.8 Hz, 1H), 8.77 (dd, J = 6.6, 1.8 Hz, 1H), 8.16 (dd, J = 8.4, 1.8 Hz, 1H), 7.57 – 7.50 (m, 2H), 7.46 (dd, J = 8.4, 4.2 Hz, 1H), 5.35 – 5.22 (m, 1H), 3.81 – 3.67 (m, 4H), 3.06 – 2.92 (m, 2H), 2.83 – 2.67 (m, 2H), 2.65 – 2.58 (m, 2H), 2.58 – 2.52 (m, 2H). **^{13}C NMR** (150 MHz, $CDCl_3$) δ 167.9 (d, J = 6.9 Hz), 148.4, 138.5, 136.5, 134.4, 128.1, 127.5, 122.0, 121.8, 116.8, 89.5 (d, J = 170.4 Hz), 67.0, 61.7 (d, J = 21.3 Hz), 54.4, 42.2 (d, J = 22.5 Hz). **^{19}F NMR (**475 MHz, $CDCl_3$) δ -180.18 – -180.48 (m, 1F). **IR** ν_{max} (film): 2955, 2923, 2852, 2357, 1710, 1560, 1463, 1377, 1246, 969 cm^{-1}. **HRMS** (ESI) m/z calcd for $C_{17}H_{20}FN_3NaO_2$ [M+Na]$^+$: 340.1432; found: 340.1435.

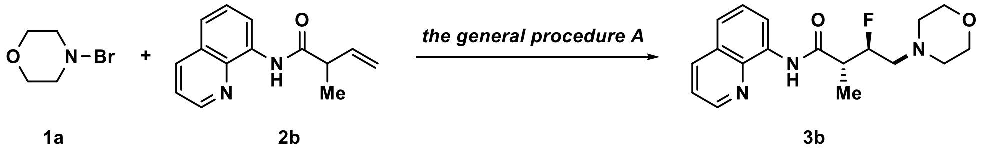

Product **3b** was prepared following the general procedure **A**. Purification using column chromatography (PE/EA = 1:1) afforded **3b** as yellow oil (49.7 mg, 0.15 mmol, 75%). **^{1}H NMR** (600 MHz, $CDCl_3$) δ 10.05 (s, 1H), 8.84 – 8.78 (m, 2H), 8.17 (d, J = 7.8 Hz, 1H), 7.57 – 7.49 (m, 2H), 7.46 (dd, J = 8.4, 4.2 Hz, 1H), 5.05 – 4.91 (m, 1H), 3.75 – 3.67 (m, 4H), 3.11 – 3.01 (m, 1H), 2.80 – 2.70 (m, 2H), 2.62 – 2.57 (m, 2H), 2.56 – 2.50 (m, 2H), 1.40 (d, J = 7.2 Hz, 3H). **^{13}C NMR**

(150 MHz, $CDCl_3$) δ 171.4 (d, J = 3.9 Hz), 148.4, 138.6, 136.5, 134.5, 128.1, 127.5, 121.9, 121.8, 116.8, 93.6 (d, J = 173.3 Hz), 67.1, 59.9 (d, J = 21.3 Hz), 54.5, 45.8 (d, J = 20.7 Hz), 13.8 (d, J = 6.2 Hz). **^{19}F NMR (**475 MHz, $CDCl_3$) δ -182.08 – -182.36 (m, 1F). **IR** ν_{max} (film): 2920, 2850, 2360, 2341, 1771, 1522, 1488, 1424, 1472, 1419, 669, 649, 419 cm^{-1}. **HRMS** (ESI) m/z calcd for $C_{18}H_{23}FN_3O_2$ [M+H]$^+$: 332.1769; found: 332.1769.

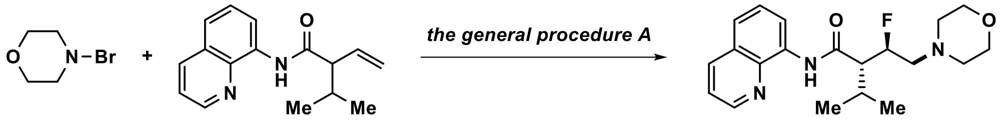

1a **2c** **3c**

Product **3c** was prepared following the general procedure **A**. Purification using column chromatography (PE/EA = 1:1) afforded **3c** as yellow oil (58.2 mg, 0.16 mmol, 81%). **^{1}H NMR** (600 MHz, $CDCl_3$) δ 10.11 (s, 1H), 8.91 – 8.76 (m, 2H), 8.16 (dd, J = 8.4, 1.2 Hz, 1H), 7.58 – 7.49 (m, 2H), 7.45 (dd, J = 8.4, 4.2 Hz, 1H), 5.19 – 5.04 (m, 1H), 3.74 – 3.66 (m, 4H), 2.82 – 2.72 (m, 2H), 2.59 – 2.48 (m, 4H), 2.47 – 2.40 (m, 1H), 2.38 – 2.32 (m, 1H), 1.16 (d, J = 6.6 Hz, 3H), 1.09 (d, J = 6.6 Hz, 3H). **^{13}C NMR** (150 MHz, $CDCl_3$) δ 170.4, 148.5, 138.7, 136.4, 134.6, 128.1, 127.5, 121.7, 116.8, 90.7 (d, J = 174.6 Hz), 67.0, 60.7 (d, J = 21.9 Hz), 59.4 (d, J = 18.9 Hz), 54.4, 27.8 (d, J = 4.2 Hz), 21.0, 20.8. **^{19}F NMR (**475 MHz, $CDCl_3$) δ -191.98 – -192.27 (m, 1F). **IR** ν_{max} (film): 2959, 2925, 2853, 2359, 2341, 1686, 1525, 1486, 1324, 1117, 1010, 826, 792 cm^{-1}. **HRMS** (ESI) m/z calcd for $C_{20}H_{27}FN_3O_2$ [M+H]$^+$: 360.2082; found: 360.2083.

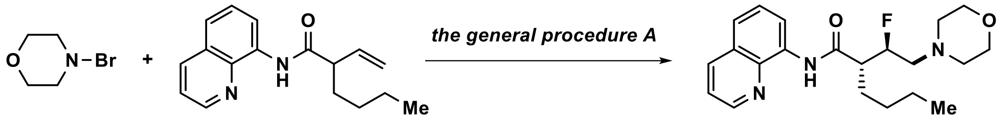

1a **2d** **3d**

Product **3d** was prepared following the general procedure **A**. Purification using column chromatography (PE/EA = 2:1) afforded **3d** as yellow oil (63.4 mg, 0.17 mmol, 85%). **^{1}H NMR** (600 MHz, $CDCl_3$) δ 10.05 (s, 1H), 8.86 – 8.78 (m, 2H), 8.16 (dd, J = 8.4, 1.8 Hz, 1H), 7.56 – 7.50 (m, 2H), 7.46 (dd, J = 8.4, 4.2 Hz, 1H), 5.05 – 4.87 (m, 1H), 3.75 – 3.68 (m, 4H), 2.89 – 2.81 (m, 1H), 2.80 – 2.74 (m, 1H), 2.74 – 2.68 (m, 1H), 2.60 – 2.50 (m, 4H), 2.00 – 1.89 (m, 1H), 1.70 – 1.61 (m, 1H), 1.44 – 1.35 (m, 4H), 0.89 (t, J = 7.2 Hz, 3H). **^{13}C NMR** (150 MHz, $CDCl_3$) δ 170.9 (d, J = 3.2 Hz), 148.4, 138.6, 136.4, 134.5, 128.1, 127.5, 121.8, 121.7, 116.8, 93.0 (d, J = 173.9 Hz), 67.1, 60.3 (d, J = 21.6 Hz), 54.4, 52.3 (d, J = 19.7 Hz), 29.6, 28.6 (d, J = 5.0 Hz), 22.8, 14.0. **^{19}F NMR (**475 MHz, $CDCl_3$) δ -183.40 – -183.80 (m, 1F). **IR** ν_{max} (film): 2956, 2926, 2854, 2362, 2337, 1595, 1528, 1485, 1461, 1378, 1118, 826, 791 cm^{-1}. **HRMS** (ESI) m/z calcd for $C_{21}H_{28}FN_3NaO_2$ [M+Na]$^+$: 396.2058; found: 396.2054.

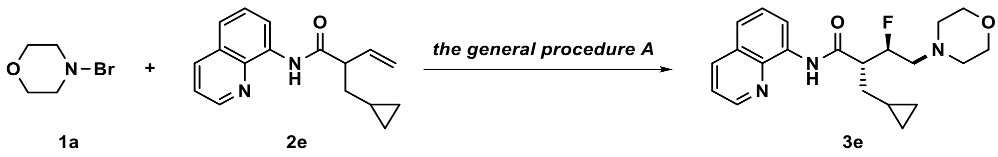

Product **3e** was prepared following the general procedure **A**. Purification using column chromatography (PE/EA = 1:1) afforded **3e** as yellow oil (46.0 mg, 0.12 mmol, 62%). **^{1}H NMR** (500 MHz, $CDCl_3$) δ 10.09 (s, 1H), 8.84 – 8.82 (m, 2H), 8.17 (dd, J = 8.5, 2.0 Hz, 1H), 7.59 – 7.49 (m, 2H), 7.46 (dd, J = 8.5, 4.5 Hz, 1H), 5.11 – 4.93 (m, 1H), 3.78 – 3.66 (m, 4H), 3.03 – 2.95 (m, 1H), 2.81 – 2.68 (m, 2H), 2.60 – 2.52 (m, 4H), 1.94 (ddd, J = 14.0, 10.0, 7.0 Hz, 1H), 1.52 (ddd, J = 13.0, 7.5, 5.0 Hz, 1H), 0.88 – 0.78 (m, 1H), 0.53 – 0.48 (m, 1H), 0.46 – 0.41 (m, 1H), 0.20 – 0.12 (m, 2H). **^{13}C NMR** (125 MHz, $CDCl_3$) δ 170.9, 148.4, 138.6, 136.5, 134.6, 128.1, 127.6, 121.83, 121.77, 116.9, 92.6 (d, J = 172.9 Hz), 67.1, 60.2 (d, J = 21.1 Hz), 54.4, 52.7 (d, J = 19.9 Hz), 33.9 (d, J = 4.9 Hz), 9.2, 5.0, 4.6. **^{19}F NMR (**475 MHz, $CDCl_3$) δ -183.92 – -184.22 (m, 1F). **IR** ν_{max} (film): 2955, 2924, 2853, 2360, 2341, 1685, 1526, 1486, 1377, 1117, 868, 826, 791 cm^{-1}. **HRMS** (ESI) m/z calcd for $C_{21}H_{27}FN_3O_2$ [M+H]$^+$: 372.2082; found: 372.2083.

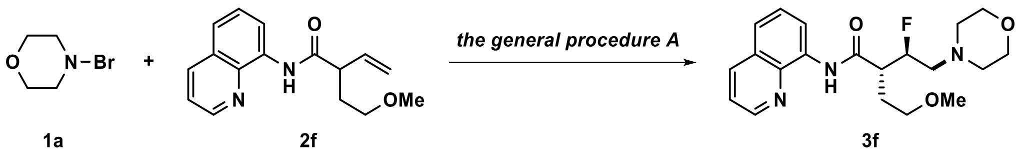

Product **3f** was prepared following the general procedure **A**. Purification using column chromatography (PE/EA = 2:1) afforded **3f** as yellow oil (41.3 mg, 0.11 mmol, 55%). **^{1}H NMR** (600 MHz, $CDCl_3$) δ 10.13 (s, 1H), 8.86 – 8.79 (m, 2H), 8.17 (dd, J = 7.8, 1.8 Hz, 1H), 7.58 – 7.50 (m, 2H), 7.46 (dd, J = 8.4, 4.2 Hz, 1H), 5.08 – 4.94 (m, 1H), 3.78 – 3.68 (m, 4H), 3.58 – 3.51 (m, 1H), 3.46 (td, J = 9.0, 4.2 Hz, 1H), 3.31 (s, 3H), 3.20 – 3.11 (m, 1H), 2.82 – 2.71 (m, 2H), 2.60 – 2.51 (m, 4H), 2.15 (ddd, J = 15.0, 10.2, 4.8 Hz, 1H), 1.98 (ddd, J = 14.4, 9.6, 5.4 Hz, 1H). **^{13}C NMR** (150 MHz, $CDCl_3$) δ 170.5 (d, J = 3.0 Hz), 148.4, 138.6, 136.4, 134.5, 128.1, 127.5, 121.9, 121.8, 116.9, 92.7 (d, J = 173.9 Hz), 70.0, 67.1, 60.2 (d, J = 21.8 Hz), 58.9, 54.4, 48.4 (d, J = 20.3 Hz), 28.8 (d, J = 5.1 Hz). **^{19}F NMR** (565 MHz, $CDCl_3$) δ -183.76 – -184.02 (m, 1F). **IR** ν_{max} (film): 2923, 2852, 2360, 2341, 1684, 1524, 1488, 1375, 1322, 1116, 826, 792, 669 cm^{-1}. **HRMS** (ESI) m/z calcd for $C_{20}H_{27}FN_3O_3$ [M+H]$^+$: 376.2031; found: 376.2034.

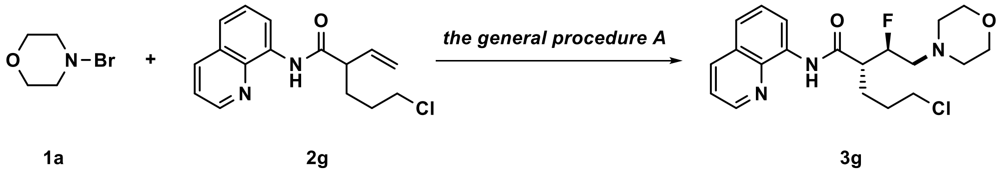

Product **3g** was prepared following the general procedure **A**. Purification using column chromatography (PE/EA = 2:1) afforded **3g** as yellow oil (40.9 mg, 0.10 mmol, 52%). **^{1}H NMR** (600 MHz, $CDCl_3$) δ 10.08 (s, 1H), 8.91 – 8.70 (m, 2H), 8.17 (d, J = 8.4 Hz, 1H), 7.61 – 7.51 (m, 2H), 7.47 (dd, J = 8.4, 4.2 Hz, 1H), 5.01 – 4.88 (m, 1H), 3.79 – 3.67 (m, 4H), 3.65 – 3.60 (m, 1H), 3.60 – 3.53 (m, 1H), 2.98 – 2.89 (m, 1H), 2.83 – 2.70 (m, 2H), 2.64 – 2.47 (m, 4H), 2.12 – 2.03 (m, 1H), 1.99 – 1.85 (m, 3H). **^{13}C NMR** (150 MHz, $CDCl_3$) δ 170.2 (d, J = 3.2 Hz), 148.5, 138.6, 136.5, 134.4, 128.1, 127.5, 122.1, 121.8, 117.0, 93.0 (d, J = 174.5 Hz), 67.1, 60.1 (d, J = 21.8 Hz), 54.5, 51.3 (d, J = 20.0 Hz), 44.8, 30.3, 26.2 (d, J = 5.4 Hz). **^{19}F NMR (**475 MHz, $CDCl_3$) δ -183.82 – -184.18 (m, 1F). **IR** ν_{max} (film): 2954, 2924, 2853, 2360, 2341, 1683, 1524, 1457, 1376, 1116, 826, 791, 669 cm^{-1}. **HRMS** (ESI) m/z calcd for $C_{20}H_{26}ClFN_3O_2$ $[M+H]^+$: 394.1692; found: 394.1707.

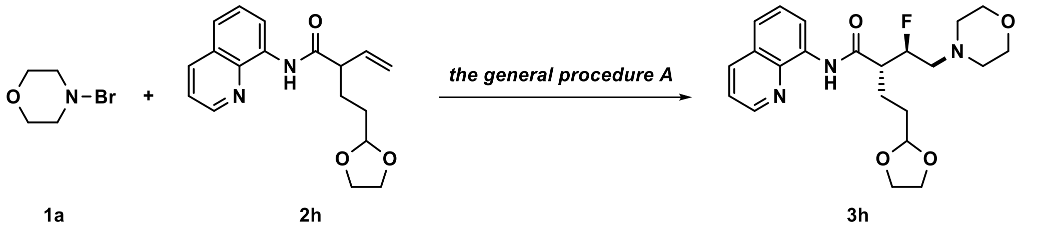

Product **3h** was prepared following the general procedure **A**. Purification using column chromatography (PE/EA = 2:1) afforded **3h** as yellow oil (58.4 mg, 0.14 mmol, 70%). **^{1}H NMR** (500 MHz, $CDCl_3$) δ 10.12 (s, 1H), 8.83 (dd, J = 6.5, 2.0 Hz, 1H), 8.81 (dd, J = 4.5, 2.0 Hz, 1H), 8.16 (dd, J = 8.5, 2.0 Hz, 1H), 7.56 – 7.50 (m, 2H), 7.46 (dd, J = 8.5, 4.5 Hz, 1H), 5.06 – 4.93 (m, 1H), 4.92 (t, J = 4.5 Hz, 1H), 4.02 – 3.98 (m, 1H), 3.96 – 3.92 (m, 1H), 3.89 – 3.83 (m, 2H), 3.77 – 3.68 (m, 4H), 3.08 – 2.97 (m, 1H), 2.80 – 2.71 (m, 2H), 2.61 – 2.50 (m, 4H), 2.08 – 2.00 (m, 1H), 1.88 – 1.84 (m, 1H), 1.83 – 1.75 (m, 2H). **^{13}C NMR** (125 MHz, $CDCl_3$) δ 170.6 (d, J = 3.6 Hz), 148.3, 138.6, 136.5, 134.5, 128.1, 127.5, 121.9, 121.7, 116.9, 104.4, 92.9 (d, J = 174.1 Hz), 67.1, 65.1, 65.0, 60.1 (d, J = 21.5 Hz), 54.4, 51.4 (d, J = 19.9 Hz), 31.3, 23.2 (d, J = 5.6 Hz). **^{19}F NMR (**475 MHz, $CDCl_3$) δ -183.02 – -183.42 (m, 1F). **IR** ν_{max} (film): 2924, 2856, 2360, 2341, 1684, 1522, 1488, 1457, 1374, 931, 866, 826, 669 cm^{-1}. **HRMS** (ESI) m/z calcd for $C_{22}H_{29}FN_3O_4$ $[M+H]^+$: 418.2137; found: 418.2145.

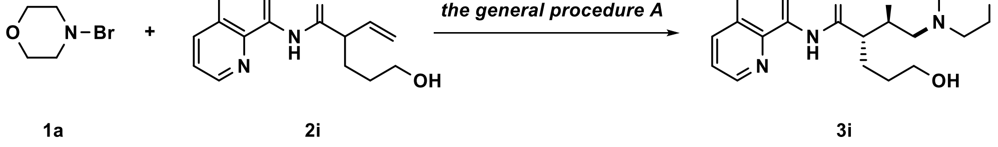

Product **3i** was prepared following the general procedure **A**. Purification using column

chromatography (PE/EA = 1:2) afforded **3i** as yellow oil (33.8 mg, 0.09 mmol, 45%). **^{1}H NMR** (600 MHz, $CDCl_3$) δ 10.07 (s, 1H), 8.84 – 8.76 (m, 2H), 8.17 (d, J = 8.4 Hz, 1H), 7.58 – 7.51 (m, 2H), 7.46 (dd, J = 8.4, 4.2 Hz, 1H), 5.03 – 4.91 (m, 1H), 3.78 – 3.75 (m, 1H), 3.74 – 3.70 (m, 4H), 3.70 – 3.66 (m, 1H), 3.04 – 2.96 (m, 1H), 2.82 – 2.72 (m, 2H), 2.60 – 2.56 (m, 2H), 2.56 – 2.50 (m, 2H), 2.10 – 1.98 (m, 2H), 1.80 – 1.75 (m, 1H), 1.73 – 1.70 (m, 1H). **^{13}C NMR** (150 MHz, $CDCl_3$) δ 170.8 (d, J = 3.0 Hz), 148.5, 138.7, 136.6, 134.3, 128.2, 127.5, 122.1, 121.8, 117.2, 93.1 (d, J = 173.9 Hz), 67.0, 62.5, 60.2 (d, J = 21.5 Hz), 54.5, 51.5 (d, J = 20.0 Hz), 30.3, 25.3 (d, J = 5.3 Hz). **^{19}F NMR (**475 MHz, $CDCl_3$) δ -182.35 – -182.65 (m, 1F). **IR** ν_{max} (film): 2925, 2855, 2360, 2341, 1685, 1527, 1486, 1323, 1116, 827, 792 cm^{-1}. **HRMS** (ESI) m/z calcd for $C_{20}H_{27}FN_3O_3$ [M+H]$^+$: 376.2031; found: 376.2033.

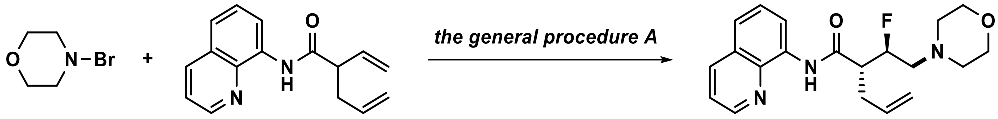

1a **2j** **3j**

Product **3j** was prepared following the general procedure **A**. Purification using column chromatography (PE/EA = 2:1) afforded **3j** as yellow oil (42.9 mg, 0.12 mmol, 60%). **^{1}H NMR** (600 MHz, $CDCl_3$) δ 10.07 (s, 1H), 8.85 – 8.78 (m, 2H), 8.16 (dd, J = 8.4, 1.8 Hz, 1H), 7.56 – 7.50 (m, 2H), 7.46 (dd, J = 8.4, 4.2 Hz, 1H), 5.86 (ddt, J = 16.8, 10.2, 7.2 Hz, 1H), 5.21 (dd, J = 16.8, 1.8 Hz, 1H), 5.09 (d, J = 10.2 Hz, 1H), 5.05 – 4.91 (m, 1H), 3.77 – 3.67 (m, 4H), 3.02 – 2.93 (m, 1H), 2.84 – 2.77 (m, 1H), 2.76 – 2.67 (m, 2H), 2.58 – 2.54 (m, 4H), 2.52 – 2.47 (m, 1H). **^{13}C NMR** (150 MHz, $CDCl_3$) δ 170.0 (d, J = 2.7 Hz), 148.4, 138.6, 136.5, 134.6, 134.4, 128.1, 127.5, 121.9, 121.8, 118.2, 116.9, 92.1 (d, J = 174.5 Hz), 67.0, 60.0 (d, J = 21.9 Hz), 54.4, 51.5 (d, J = 19.8 Hz), 33.0 (d, J = 5.3 Hz). **^{19}F NMR (**475 MHz, $CDCl_3$) δ -186.32 – -186.68 (m, 1F). **IR** ν_{max} (film): 2955, 2924, 2855, 2359, 2361, 2342, 1685, 1525, 1485, 1457, 1377, 1322, 1264, 1116, 916, 866, 826, 791, 741 cm^{-1}. **HRMS** (ESI) m/z calcd for $C_{20}H_{25}FN_3O_2$ [M+H]$^+$:358.1925; found: 358.1933.

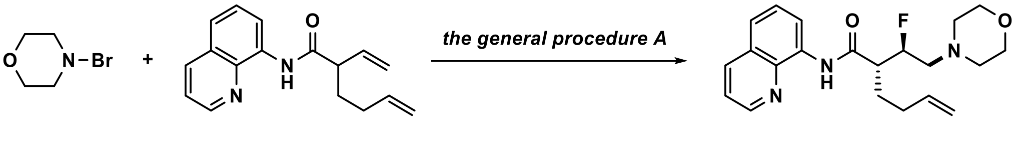

1a **2k** **3k**

Product **3k** was prepared following the general procedure **A**. Purification using column chromatography (PE/EA = 2:1) afforded **3k** as yellow oil (51.2 mg, 0.14 mmol, 69%). **^{1}H NMR** (600 MHz, $CDCl_3$) δ 10.05 (s, 1H), 8.85 – 8.80 (m, 2H), 8.17 (d, J = 8.4 Hz, 1H), 7.57 – 7.51 (m, 2H), 7.46 (dd, J = 8.4, 4.2 Hz, 1H), 5.82 (ddt, J = 16.8, 10.2, 6.6 Hz, 1H), 5.08 (d, J = 16.8 Hz, 1H),

5.03 (d, J = 10.8 Hz, 1H), 5.01 – 4.91 (m, 1H), 3.78 – 3.69 (m, 4H), 2.97 – 2.88 (m, 1H), 2.82 – 2.70 (m, 2H), 2.60 – 2.51 (m, 4H), 2.28 – 2.22 (m, 1H), 2.21 – 2.14 (m, 1H), 2.11 – 2.03 (m, 1H), 1.77 – 1.73 (m, 1H). **^{13}C NMR** (150 MHz, $CDCl_3$) δ 170.5 (d, J = 3.3 Hz), 148.4, 138.6, 137.5, 136.4, 134.4, 128.1, 127.5, 121.9, 121.8, 116.9, 116.0, 92.9 (d, J = 174.0 Hz), 67.0, 60.2 (d, J = 21.5 Hz), 54.4, 51.2 (d, J = 20.0 Hz), 31.4, 27.8 (d, J = 5.1 Hz). **^{19}F NMR (**475 MHz, $CDCl_3$) δ -183.16 – -183.56 (m, 1F). **IR** ν_{max} (film): 2954, 2924, 2853, 2360, 2341, 1685, 1525, 1485, 1457, 1377, 1323, 1116, 914, 868, 825, 791, 755 cm^{-1}. **HRMS** (ESI) m/z calcd for $C_{21}H_{27}FN_3O_2$ [M+H]$^+$: 372.2082; found: 372.2074.

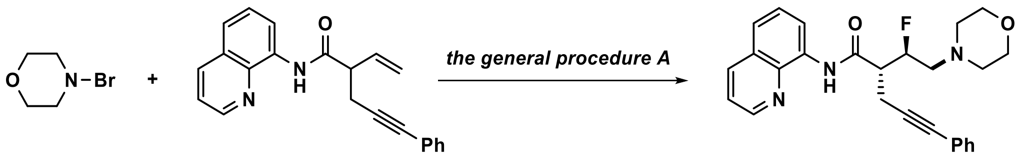

1a **2l** **3l**

Product **3l** was prepared following the general procedure **A**. Purification using column chromatography (PE/EA = 2:1) afforded **3l** as yellow solid (40.5 mg, 0.09 mmol, 47%), m.p. 70 °C. **^{1}H NMR** (600 MHz, $CDCl_3$) δ 10.26 (s, 1H), 8.85 (d, J = 6.6 Hz, 1H), 8.71 (d, J = 3.6 Hz, 1H), 8.16 (d, J = 8.4 Hz, 1H), 7.58 – 7.52 (m, 2H), 7.44 (dd, J = 8.4, 4.2 Hz, 1H), 7.32 (d, J = 7.2 Hz, 2H), 7.27 – 7.19 (m, 3H), 5.31 – 5.17 (m, 1H), 3.83 – 3.77 (m, 2H), 3.77 – 3.71 (m, 2H), 3.34 – 3.25 (m, 1H), 3.04 (dd, J = 16.8, 7.8 Hz, 1H), 2.98 (dd, J = 17.4, 7.2 Hz, 1H), 2.88 (ddd, J = 19.8, 13.2, 5.4 Hz, 1H), 2.80 (ddd, J = 18.0, 13.2, 6.0 Hz, 1H), 2.58 (br, 4H). **^{13}C NMR** (150 MHz, $CDCl_3$) δ 169.0 (d, J = 2.6 Hz), 148.3, 138.7, 136.5, 134.5, 131.7, 128.3, 128.12, 128.10, 127.6, 123.3, 122.0, 121.8, 117.2, 91.2 (d, J = 174.8 Hz), 86.4, 83.1, 67.0, 59.6 (d, J = 23.3 Hz), 54.4, 50.0 (d, J = 20.1 Hz), 19.3 (d, J = 5.7 Hz). **^{19}F NMR (**475 MHz, $CDCl_3$) δ -190.18 – -190.43 (m, 1F). **IR** ν_{max} (film): 2924, 2853, 2359, 2341, 1684, 1558, 1488, 1424, 1375, 1323, 1116, 1010, 826, 791, 756, 690 cm^{-1}. **HRMS** (ESI) m/z calcd for $C_{26}H_{27}FN_3O_2$ [M+H]$^+$: 432.2082; found: 432.2083.

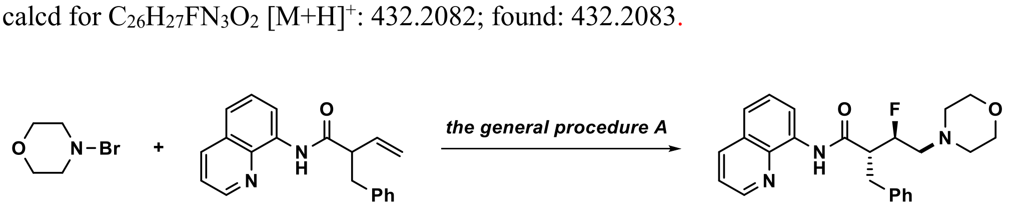

1a **2m** **3m**

Product **3m** was prepared following the general procedure **A**. Purification using column chromatography (PE/EA = 2:1) afforded **3m** as yellow oil (57.8 mg, 0.14 mmol, 71%). **^{1}H NMR** (600 MHz, $CDCl_3$) δ 9.98 (s, 1H), 8.79 (d, J = 7.8 Hz, 1H), 8.75 (d, J = 4.2 Hz, 1H), 8.11 (d, J = 8.4 Hz, 1H), 7.54 – 7.45 (m, 2H), 7.41 (dd, J = 8.4, 4.2 Hz, 1H), 7.30 (d, J = 7.8 Hz, 2H), 7.25 (t, J = 7.2 Hz, 2H), 7.16 (t, J = 7.2 Hz, 1H), 4.98 – 4.84 (m, 1H), 3.73 – 3.70 (m, 2H), 3.69 – 3.65 (m, 2H), 3.25

(dd, J = 13.2, 7.8 Hz, 1H), 3.22 – 3.14 (m, 1H), 3.09 (dd, J = 13.2, 6.6 Hz, 1H), 2.83 – 2.70 (m, 2H), 2.55 – 2.49 (m, 2H), 2.48 – 2.42 (m, 2H). **^{13}C NMR** (150 MHz, $CDCl_3$) δ 169.9 (d, J = 2.3 Hz), 148.2, 138.5, 136.3, 134.4, 129.1, 128.7, 128.0, 127.4, 126.73, 126.72, 121.8, 121.7, 116.9, 91.6 (d, J = 174.8 Hz), 67.0, 59.9 (d, J = 22.2 Hz), 54.4, 53.5 (d, J = 19.5 Hz), 34.7(d, J = 4.7 Hz). **^{19}F NMR (**475 MHz, $CDCl_3$) δ -187.95 – -188.33 (m, 1F). **IR** ν_{max} (film): 2954, 2924, 2853, 2360, 2341, 1770, 1684, 1522, 1485, 1455, 1376, 1323, 1116, 825, 790, 748, 698 cm^{-1}. **HRMS** (ESI) m/z calcd for $C_{24}H_{26}FN_3NaO_2$ [M+Na]$^+$: 430.1901; found: 430.1910.

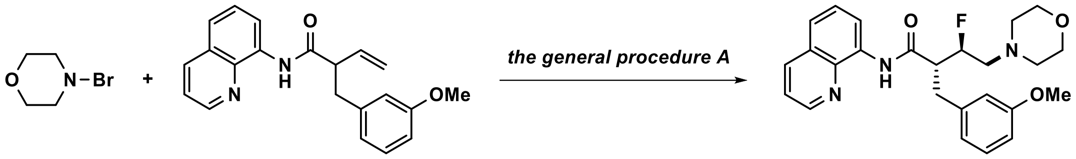

1a **2n** **3n**

Product **3n** was prepared following general procedure **A**. Purification using column chromatography (PE/EA = 2:1) afforded **3n** as yellow oil (48.1 mg, 0.11 mmol, 55%). **^{1}H NMR** (600 MHz, $CDCl_3$) δ 9.99 (s, 1H), 8.80 (d, J = 7.2 Hz, 1H), 8.76 (d, J = 3.6 Hz, 1H), 8.13 (d, J = 7.8 Hz, 1H), 7.54 – 7.48 (m, 2H), 7.43 (dd, J = 8.4, 4.2 Hz, 1H), 7.17 (t, J = 7.8 Hz, 1H), 6.89 (d, J = 7.8 Hz, 1H), 6.84 (s, 1H), 6.70 (dd, J = 8.4, 2.4 Hz, 1H), 4.99 – 4.85 (m, 1H), 3.75 – 3.72 (m, 2H), 3.71 (s, 3H), 3.70 – 3.66 (m, 2H), 3.24 – 3.15 (m, 2H), 3.07 (dd, J = 12.6, 6.6 Hz, 1H), 2.84 – 2.78 (m, 1H), 2.77 – 2.70 (m, 1H), 2.55 – 2.49 (m, 2H), 2.48 – 2.43 (m, 2H). **^{13}C NMR** (150 MHz, $CDCl_3$) δ 169.9 (d, J = 2.4 Hz), 159.9, 148.2, 140.1, 138.5, 136.4, 134.4, 129.7, 128.0, 127.4, 121.9, 121.7, 121.5, 116.9, 114.7, 112.4, 91.6 (d, J = 174.8 Hz), 67.0, 59.9 (d, J = 22.2 Hz), 55.2, 54.4, 53.4 (d, J = 19.8 Hz), 34.8 (d, J = 4.7 Hz). **^{19}F NMR** (565 MHz, $CDCl_3$) δ -187.98 – -188.26 (m, 1F). **IR** ν_{max} (film): 2924, 2853, 2360, 2341, 1684, 1524, 1486, 1456, 1424, 1375, 1323, 1259, 1116, 1044, 868, 825, 790, 669 cm^{-1}. **HRMS** (ESI) m/z calcd for $C_{25}H_{29}FN_3O_3$ [M+H]$^+$: 438.2187; found: 438.2184.

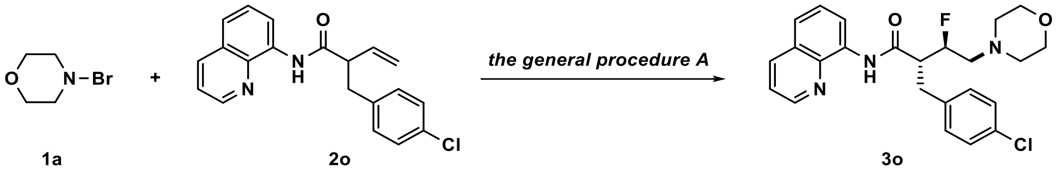

1a **2o** **3o**

Product **3o** was prepared following the general procedure **A**. Purification using column chromatography (PE/EA = 2:1) afforded **3o** as yellow oil (56.5 mg, 0.13 mmol, 64%). **^{1}H NMR** (600 MHz, $CDCl_3$) δ 9.93 (s, 1H), 8.79 – 8.74 (m, 2H), 8.15 (dd, J = 8.4, 1.8 Hz, 1H), 7.54 – 7.49 (m, 2H), 7.44 (dd, J = 8.4, 4.2 Hz, 1H), 7.25 – 7.19 (m, 4H), 4.95 – 4.83 (m, 1H), 3.75 – 3.71 (m, 2H), 3.70 – 3.66 (m, 2H), 3.21 (dd, J = 13.2, 8.4 Hz, 1H), 3.17 – 3.10 (m, 1H), 3.05 (dd, J = 13.2, 6.6 Hz,

1H), 2.84 – 2.72 (m, 2H), 2.55 – 2.51 (m, 2H), 2.50 – 2.45 (m, 2H). **^{13}C NMR** (150 MHz, $CDCl_3$) δ 169.5 (d, J = 2.6 Hz), 148.3, 138.6, 137.1, 136.4, 134.3, 132.7, 130.5, 128.9, 128.1, 127.5, 122.0, 121.8, 117.0, 91.7 (d, J = 174.9 Hz), 67.0, 59.9 (d, J = 22.1 Hz), 54.5, 53.6 (d, J = 19.8 Hz), 34.1 (d, J = 5.0 Hz). **^{19}F NMR (**475 MHz, $CDCl_3$) δ -187.52 – -187.80 (m, 1F). **IR** ν_{max} (film): 2921, 2850, 2359, 2341, 1684, 1525, 1488, 1424, 1375, 1323, 1116, 1013, 826, 791, 669 cm^{-1}. **HRMS** (ESI) m/z calcd for $C_{24}H_{26}ClFN_3O_2$ [M+H]$^+$: 442.1692; found: 442.1695.

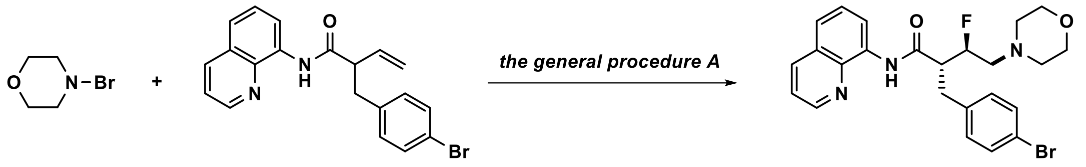

Product **3p** was prepared following the general procedure **A**. Purification using column chromatography (PE/EA = 2:1) afforded **3p** as yellow soild (61.1 mg, 0.13 mmol, 63%), m.p. 85 °C. **^{1}H NMR** (600 MHz, $CDCl_3$) δ 9.93 (s, 1H), 8.80 – 8.73 (m, 2H), 8.14 (dd, J = 8.4, 1.8 Hz, 1H), 7.54 – 7.50 (m, 2H), 7.44 (dd, J = 8.4, 4.2 Hz, 1H), 7.36 (d, J = 8.4 Hz, 2H), 7.17 (d, J = 8.4 Hz, 2H), 4.98 – 4.83 (m, 1H), 3.75 – 3.71 (m, 2H), 3.70 – 3.66 (m, 2H), 3.20 (dd, J = 13.2, 8.4 Hz, 1H), 3.16 – 3.09 (m, 1H), 3.03 (dd, J = 13.2, 6.0 Hz, 1H), 2.84 – 2.72 (m, 2H), 2.56 – 2.51 (m, 2H), 2.50 – 2.45 (m, 2H). **^{13}C NMR** (150 MHz, $CDCl_3$) δ 169.5 (d, J = 2.4 Hz), 148.3, 138.5, 137.6, 136.4, 134.3, 131.9, 130.9, 128.1, 127.4, 122.0, 121.8, 120.7, 117.0, 91.7 (d, J = 175.1 Hz), 67.0, 59.9 (d, J = 22.2 Hz), 54.5, 53.5 (d, J = 19.8 Hz), 34.1 (d, J = 5.0 Hz). **^{19}F NMR (**475 MHz, $CDCl_3$) δ -187.45 – -188.85 (m, 1F). **IR** ν_{max} (film): 2955, 2925, 2853, 2360, 2341, 1685, 1525, 1486, 1424, 1377, 1323, 1241, 1116, 1070, 1011, 826, 791, 749 cm^{-1}. **HRMS** (ESI) m/z calcd for $C_{24}H_{26}BrFN_3O_2$ [M+H]$^+$: 486.1187; found: 486.1181.

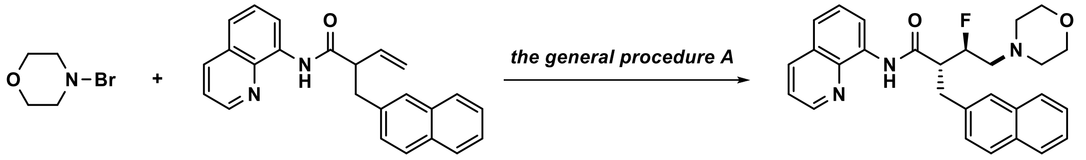

Product **3q** was prepared following the general procedure **A**. Purification using column chromatography (PE/EA = 2:1) afforded **3q** as yellow solid (75.0 mg, 0.16 mmol, 82%), m.p. 60 °C. **^{1}H NMR** (600 MHz, $CDCl_3$) δ 9.72 (s, 1H), 8.76 (d, J = 7.8 Hz, 1H), 8.57 (dd, J = 4.2, 1.8 Hz, 1H), 8.32 (d, J = 8.4 Hz, 1H), 8.06 (dd, J = 8.4, 1.8 Hz, 1H), 7.81 (d, J = 8.4 Hz, 1H), 7.66 – 7.57 (m, 2H), 7.49 (t, J = 7.8 Hz, 2H), 7.43 (dd, J = 14.4, 7.8 Hz, 2H), 7.34 (dd, J = 8.4, 4.2 Hz, 1H), 7.25 – 7.22 (m, 1H), 5.12 – 4.93 (m, 1H), 3.75 – 3.67 (m, 4H), 3.62 (dd, J = 13.8, 6.6 Hz, 1H), 3.56 (dd, J

= 13.8, 8.4 Hz, 1H), 3.49 – 3.41 (m, 1H), 2.90 – 2.76 (m, 2H), 2.62 – 2.54 (m, 2H), 2.46 – 2.40 (m, 2H). **^{13}C NMR** (150 MHz, $CDCl_3$) δ 170.1 (d, J = 2.6 Hz), 148.1, 138.4, 136.2, 134.3, 134.2, 131.8, 129.1, 127.9, 127.6, 127.3, 126.3, 125.7, 125.6, 123.8, 121.8, 121.5, 116.9, 92.4 (d, J = 174.0 Hz), 67.0, 59.7 (d, J = 21.6 Hz), 54.5, 52.1 (d, J = 19.8 Hz), 32.0 (d, J = 5.0 Hz). **^{19}F NMR (**475 MHz, $CDCl_3$) δ -184.25 – -184.75 (m, 1F). **IR** ν_{max} (film): 2955, 2921, 2851, 2360, 2341, 1686, 1525, 1486, 1456, 1424, 1378, 1324, 1164, 1117, 924, 877, 826, 790, 779, 669 cm^{-1}. **HRMS** (ESI) m/z calcd for $C_{28}H_{29}FN_3O_2$ $[M+H]^+$: 458.2238; found: 458.2240.

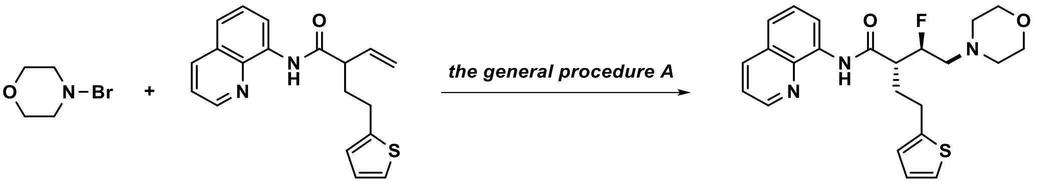

1a **2r** **3r**

Product **3r** was prepared following the general procedure **A**. Purification using column chromatography (PE/EA = 2:1) afforded **3r** as yellow oil (51.3 mg, 0.12 mmol, 60%). **^{1}H NMR** (600 MHz, $CDCl_3$) δ 10.06 (s, 1H), 8.85 (d, J = 7.2 Hz, 1H), 8.81 (d, J = 4.2 Hz, 1H), 8.16 (d, J = 8.4 Hz, 1H), 7.58 – 7.51 (m, 2H), 7.46 (dd, J = 8.4, 4.2 Hz, 1H), 7.14 (d, J = 4.8 Hz, 1H), 6.92 (t, J = 4.2 Hz, 1H), 6.87 – 6.82 (m, 1H), 5.02 – 4.90 (m, 1H), 3.73 – 3.64 (m, 4H), 3.06 – 2.90 (m, 3H), 2.80 – 2.67 (m, 2H), 2.58 – 2.52 (m, 2H), 2.51 – 2.45 (m, 2H), 2.42 – 2.33 (m, 1H), 2.04 – 1.95 (m, 1H). **^{13}C NMR** (150 MHz, $CDCl_3$) δ 170.2 (d, J = 3.2 Hz), 148.4, 143.8, 138.6, 136.4, 134.4, 128.1, 127.5, 126.9, 125.1, 123.6, 122.0, 121.8, 116.9, 93.0 (d, J = 174.6 Hz), 67.0, 59.9 (d, J = 21.8 Hz), 54.4, 50.7 (d, J = 19.8 Hz), 30.7 (d, J = 5.1 Hz), 27.6. **^{19}F NMR (**475 MHz, $CDCl_3$) δ -183.12 – -183.43 (m, 1F). **IR** ν_{max} (film): 2925, 2854, 2360, 2341, 1686, 1526, 1486, 1456, 1425, 1378, 1324, 1245, 1117, 1011, 869, 826, 792, 696 cm^{-1}. **HRMS** (ESI) m/z calcd for $C_{23}H_{27}FN_3O_2S$ $[M+H]^+$: 428.1803; found: 428.1806.

The general procedure B:

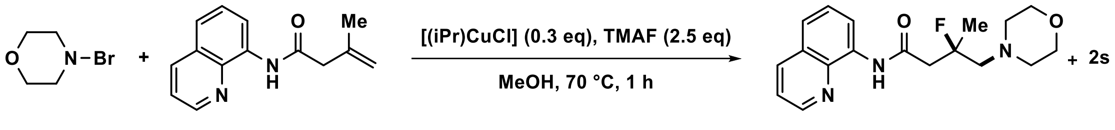

1a **2s** **3s**

Preparation of *N*-bromodialkylamine compound: To a dry round bottom flask were added morpholine (260 μl, 3.0 mmol), *N*-bromosuccinimide (NBS, 534 mg, 3.0 mmol) and anhydrous

MeOH (5.0 mL). The mixture was stirred at 0 °C avoiding light for 30 min. The solution of **1a** (0.60 M) was carried forward to the next step without further purification.

To a dry Schlenk flask were added [(iPr)CuCl] (29.3 mg, 0.06 mmol, 0.3 equiv), **2s** (45.2 mg, 0.20 mmol, 1.0 equiv), tetramethylammonium fluoride (TMAF, 46.6 mg, 0.50 mmol, 2.5 equiv) and anhydrous MeOH (1.0 mL). The mixture was degassed three times with argon. Then pre-prepared *N*-bromodialkylamine **1a** (1.0 mL, 0.60 mmol, 0.60 M in MeOH, 3.0 equiv) was added by syringe pump dropwise into the reaction mixture for 1 hr at 70 °C (oil bath). This process should avoid exposure to light. When the addition was completed, the mixture was cooled to ambient temperature. The reaction mixture was filtered by Celite, and the filtrate was concentrated *in vacuo*. Further purification by a flash column chromatography using eluents (PE/EA = 3:1) afforded the desired product **3s** as yellow oil (26.5 mg, 0.08 mmol, 40%) and recovered the alkene substrate **2s** (13.6 mg, 0.06 mmol, 30%). **^{1}H NMR** (500 MHz, $CDCl_3$) δ 10.19 (s, 1H), 8.81 (dd, J = 4.0, 2.0 Hz, 1H), 8.79 (dd, J = 6.5, 2.0 Hz, 1H), 8.16 (dd, J = 8.0, 1.5 Hz, 1H), 7.56 – 7.50 (m, 2H), 7.46 (dd, J = 8.0, 4.0 Hz, 1H), 3.82 – 3.74 (m, 2H), 3.74 – 3.66 (m, 2H), 3.23 (dd, J = 15.0, 13.0 Hz, 1H), 2.89 (t, J = 14.0 Hz, 1H), 2.77 – 2.66 (m, 3H), 2.63 (d, J = 4.0 Hz, 1H), 2.57 – 2.48 (m, 2H), 1.55 (d, J = 22.5 Hz, 3H). **^{13}C NMR** (125 MHz, $CDCl_3$) δ 168.2 (d, J = 11.6 Hz), 148.3, 138.6, 136.5, 134.8, 128.1, 127.5, 121.9, 121.8, 116.9, 97.5 (d, J = 170.6 Hz), 67.2, 65.2 (d, J = 19.9 Hz), 55.0 (d, J = 2.8 Hz), 46.6 (d, J = 24.6 Hz), 24.0 (d, J = 23.6 Hz). **^{19}F NMR (**475 MHz, $CDCl_3$) δ -137.49 – -137.85 (m, 1F). **IR** ν_{max} (film): 2955, 2924, 2853, 2360, 2341, 1684, 1525, 1486, 1424, 1380, 1324, 1260, 1117, 1071, 1016, 922, 866, 791, 756, 669 cm^{-1}. **HRMS** (ESI) m/z calcd for $C_{18}H_{23}FN_3O_2$ [M+H]$^+$: 332.1769; found: 332.1770.

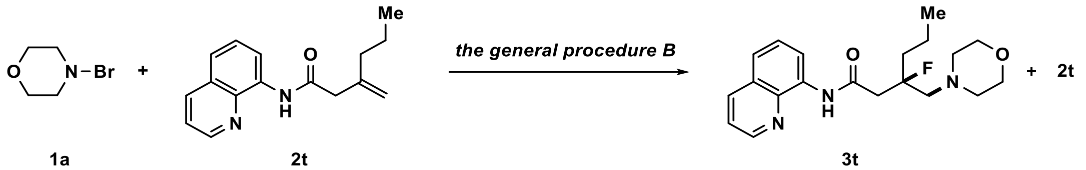

Product **3t** was prepared following the general procedure **B**. except that CuTC (11.4 mg, 0.06 mmol, 0.3 equiv) was used to replace [(iPr)CuCl]. Purification using column chromatography (PE/EA = 3:1) afforded **3t** as yellow oil (23.0 mg, 0.06 mmol, 32%) and recovered the alkene substrate **2t** (20.3 mg, 0.08 mmol, 40%). **^{1}H NMR** (600 MHz, $CDCl_3$) δ 10.22 (s, 1H), 8.83 – 8.77 (m, 2H), 8.16 (d, J = 8.4 Hz, 1H), 7.56 – 7.50 (m, 2H), 7.46 (dd, J = 8.4, 4.2 Hz, 1H), 3.82 – 3.75 (m, 2H), 3.72 – 3.65 (m, 2H), 3.26 (t, J = 13.2 Hz, 1H), 2.88 (t, J = 13.8 Hz, 1H), 2.75 – 2.67 (m, 3H), 2.64 (d, J = 8.4 Hz, 1H), 2.53 – 2.46 (m, 2H), 1.86 – 1.74 (m, 2H), 1.63 – 1.51 (m, 2H), 0.96 (t, J =

7.8 Hz, 3H). **^{13}C NMR** (150 MHz, $CDCl_3$) δ 168.4 (d, J = 13.1 Hz), 148.3, 138.7, 136.5, 134.9, 128.2, 127.5, 121.84, 121.76, 117.0, 99.3 (d, J = 173.3 Hz), 67.2, 64.0 (d, J = 19.8 Hz), 55.1 (d, J = 3.2 Hz), 44.5 (d, J = 24.6 Hz), 39.4 (d, J = 21.6 Hz), 16.5 (d, J = 4.7 Hz), 14.6. **^{19}F NMR (**475 MHz, $CDCl_3$) δ -145.30 – -145.75 (m, 1F). **IR** ν_{max} (film): 2955, 2925, 2852, 2360, 2342, 1684, 1525, 1485, 1424, 1379, 1324, 1245, 1116, 912, 826, 791, 748 cm^{-1}. **HRMS** (ESI) m/z calcd for $C_{20}H_{27}FN_3O_2$ [M+H]$^+$: 360.2082; found: 360.2085.

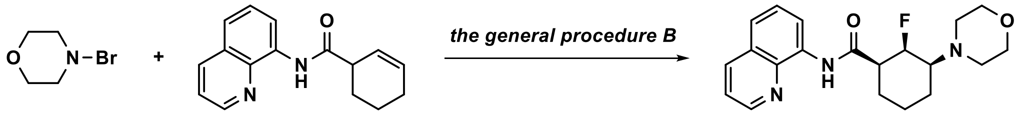

1a **2u** **3u**

Product **3u** was prepared following the general procedure **B**. except that CuI (11.4 mg, 0.06 mmol, 0.3 equiv) was used to replace [(iPr)CuCl]. Purification using column chromatography (PE/EA = 3:1) afforded **3t** as yellow oil (40.0 mg, 0.11 mmol, 56%). **^{1}H NMR** (600 MHz, $CDCl_3$) δ 10.28 (s, 1H), 8.83 – 8.79 (m, 2H), 8.15 (dd, J = 8.4, 1.8 Hz, 1H), 7.56 – 7.49 (m, 2H), 7.45 (dd, J = 8.4, 4.2 Hz, 1H), 5.29 – 5.16 (m, 1H), 3.76 – 3.68 (m, 4H), 3.28 – 3.17 (m, 1H), 2.91 – 2.83 (m, 1H), 2.67 – 2.60 (m, 4H), 2.21 – 2.12 (m, 1H), 1.97 – 1.89 (m, 1H), 1.88 – 1.80 (m, 1H), 1.73 – 1.66 (m, 3H). **^{13}C NMR** (150 MHz, $CDCl_3$) δ 171.3, 148.4, 138.8, 136.4, 134.7, 128.1, 127.5, 121.8, 121.7, 116.8, 90.5 (d, J = 175.2 Hz), 67.5, 61.7 (d, J = 20.3 Hz), 50.7, 45.8 (d, J = 18.6 Hz), 24.6 (d, J = 4.2 Hz), 23.4, 20.0. **^{19}F NMR (**475 MHz, $CDCl_3$) δ -191.95 – -193.05 (m, 1F). **IR** ν_{max} (film): 2953, 2925, 2853, 2360, 2341, 1681, 1527, 1486, 1459, 1379, 1325, 1260, 1161, 1118, 1011, 865, 826, 791, 756 cm^{-1}. **HRMS** (ESI) m/z calcd for $C_{20}H_{25}FN_3O_2$ [M+H]$^+$: 358.1925; found: 358.1930.

The procedure for the synthesis of product 3v:

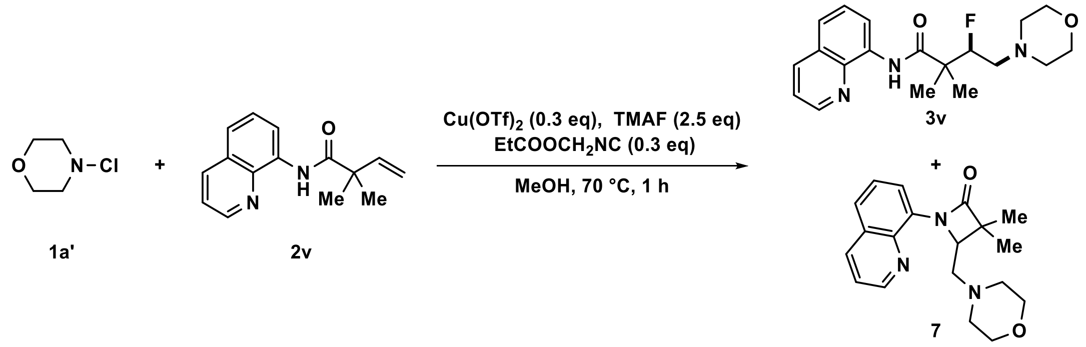

To a dry Schlenk flask were added $Cu(OTf)_2$ (21.7 mg, 0.06 mmol, 0.3 equiv), **2v** (48.0 mg, 0.20 mmol, 1.0 equiv), tetramethylammonium fluoride (TMAF, 46.6 mg, 0.50 mmol, 2.5 equiv),

ethyl isocyanoacetate (6.5 μL, 0.06 mmol, 0.3 equiv) and anhydrous MeOH (1.0 mL). The mixture was degassed three times with argon. Then purified *N*-chlorodialkylamine **1a'**[11] (72.6 mg, 0.60 mmol, 3.0 equiv) dissolved in anhydrous MeOH (1.0 mL) was added by syringe pump dropwise into the reaction mixture for 1 hr at 70 °C (oil bath). This process should avoid exposure to light. When the addition was completed, the mixture was cooled to ambient temperature. The reaction mixture was filtered by Celite, and the filtrate was concentrated *in vacuo.* Further purification by a flash column chromatography using eluents (PE/EA = 3:1) afforded the desired product **3v** as yellow oil (12.4 mg, 0.04 mmol, 18%) and compound **7** as yellow oil (29.3 mg, 0.09 mmol, 45%).

Compound **3v** : **^{1}H NMR** (600 MHz, $CDCl_3$) δ 10.37 (s, 1H), 8.82 (dd, *J* = 4.2, 1.8 Hz, 1H), 8.76 (dd, *J* = 7.2, 1.8 Hz, 1H), 8.17 (dd, *J* = 7.8, 1.2 Hz, 1H), 7.56 – 7.50 (m, 2H), 7.46 (dd, *J* = 8.4, 4.2 Hz, 1H), 5.00 – 4.87 (m, 1H), 3.64 – 3.57 (m, 4H), 2.75 – 2.63 (m, 2H), 2.55 – 2.48 (m, 4H), 1.50 (s, 3H), 1.45 (s, 3H). **^{13}C NMR** (150 MHz, $CDCl_3$) δ 173.4 (d, *J* = 4.1 Hz), 148.5, 138.9, 136.5, 134.6, 128.1, 127.5, 121.8, 116.6, 96.1 (d, *J* = 176.6 Hz), 67.0, 59.4 (d, *J* = 21.9 Hz), 54.3, 47.2 (d, *J* = 19.5 Hz), 22.9 (d, *J* = 5.3 Hz), 20.8 (d, *J* = 4.7 Hz). **^{19}F NMR (**475 MHz, $CDCl_3$) δ -188.15 – -188.43 (m, 1F). **IR** ν_{max} (film): 2955, 2924, 2853, 1741, 1681, 1530, 1487, 1462, 1378, 1325, 1154, 1118, 825, 791, 592 cm^{-1}. **HRMS** (ESI) m/z calcd for $C_{19}H_{25}FN_3O_2$ [M+H]$^+$: 346.1925; found: 346.1928.

Compound **7** : **^{1}H NMR** (600 MHz, $CDCl_3$) δ 8.82 (dd, *J* = 4.2, 1.8 Hz, 1H), 8.16 (d, *J* = 7.8 Hz, 1H), 8.12 (dd, *J* = 7.8, 1.2 Hz, 1H), 7.58 (d, *J* = 8.4 Hz, 1H), 7.49 (t, *J* = 7.8 Hz, 1H), 7.40 (dd, *J* = 8.4, 4.2 Hz, 1H), 5.04 (dd, *J* = 9.0, 4.2 Hz, 1H), 3.57 – 3.47 (m, 4H), 2.75 (dd, *J* = 12.6, 4.8 Hz, 1H), 2.61 – 2.50 (m, 3H), 2.43 – 2.33 (m, 2H), 1.55 (s, 3H), 1.39 (s, 3H). **^{13}C NMR** (150 MHz, $CDCl_3$) δ 173.7, 148.9, 141.4, 136.1, 133.7, 129.1, 126.8, 124.2, 122.4, 121.4, 67.2, 64.3, 58.3, 54.2, 53.4, 23.7, 17.0. **IR** ν_{max} (film): 3350, 2954, 2928, 2821, 2360, 2341, 1684, 1525, 1486, 1425, 1385, 1326, 1144, 1096, 1039, 827, 792, 758 cm^{-1}. **HRMS** (ESI) m/z calcd for $C_{19}H_{24}N_3O_2$ [M+H]$^+$: 326.1863; found: 326.1870.

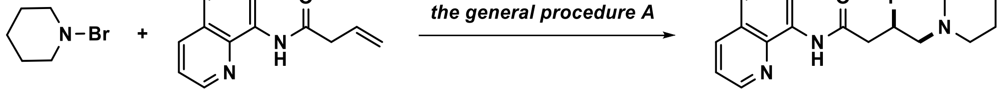

1b **2a** **8a**

Product **8a** was prepared following the general procedure **A**. Purification using column chromatography (PE/EA = 5:1) afforded **8a** as yellow oil (44.1 mg, 0.14 mmol, 70%). **^{1}H NMR** (600 MHz, $CDCl_3$) δ 10.00 (s, 1H), 8.81 (dd, *J* = 4.2, 1.8 Hz, 1H), 8.78 (dd, *J* = 7.2, 1.8 Hz, 1H), 8.16 (dd, *J* = 8.4, 1.8 Hz, 1H), 7.57 – 7.49 (m, 2H), 7.45 (dd, *J* = 8.4, 4.2 Hz, 1H), 5.36 – 5.21 (m,

1H), 3.00 – 2.91 (m, 2H), 2.75 (ddd, J = 20.4, 13.8, 6.6 Hz, 1H), 2.62 (ddd, J = 24.0, 13.8, 4.2 Hz, 1H), 2.56 – 2.45 (m, 4H), 1.63 – 1.55 (m, 4H), 1.46 – 1.39 (m, 2H). **^{13}C NMR** (150 MHz, $CDCl_3$) δ 168.1 (d, J = 5.6 Hz), 148.3, 138.5, 136.4, 134.5, 128.1, 127.5, 121.9, 121.8, 116.8, 89.5 (d, J = 170.1 Hz), 62.3 (d, J = 21.3 Hz), 55.4, 42.5 (d, J = 22.4 Hz), 26.1, 24.3. **^{19}F NMR** (565 MHz, $CDCl_3$) δ -179.81 – -180.08 (m, 1F). **IR** ν_{max} (film): 2994, 2927, 2360, 2341, 1770, 1758, 1685, 1526, 1486, 1424, 1375, 1325, 1245, 1051, 913, 826, 747 cm^{-1}. **HRMS** (ESI) m/z calcd for $C_{18}H_{23}FN_3O$ [M+H]$^+$: 316.1820; found: 316.1820.

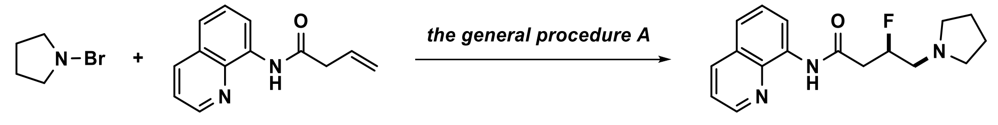

1c 2a 8b

Product **8b** was prepared following the general procedure **A**. Purification using column chromatography (PE/EA = 5:1) afforded **8b** as yellow oil (30.1 mg, 0.10 mmol, 50%). **^{1}H NMR** (600 MHz, $CDCl_3$) δ 10.07 (s, 1H), 8.80 (dd, J = 4.2, 1.8 Hz, 1H), 8.78 (dd, J = 7.2, 1.8 Hz, 1H), 8.16 (dd, J = 7.8, 1.8 Hz, 1H), 7.56 – 7.50 (m, 2H), 7.45 (dd, J = 8.4, 4.2 Hz, 1H), 5.32 – 5.16 (m, 1H), 3.04 – 2.97 (m, 1H), 2.96 (dd, J = 6.0, 3.0 Hz, 1H), 2.94 – 2.87 (m, 1H), 2.78 (ddd, J = 17.4, 13.2, 3.6 Hz, 1H), 2.68 – 2.62 (m, 2H), 2.62 – 2.55 (m, 2H), 1.83 – 1.76 (m, 4H). **^{13}C NMR** (150 MHz, $CDCl_3$) δ 168.1 (d, J = 6.0 Hz), 148.3, 138.5, 136.4, 134.6, 128.1, 127.5, 121.9, 121.8, 116.8, 90.2 (d, J = 170.6 Hz), 59.2 (d, J = 21.2 Hz), 55.0, 42.3 (d, J = 22.4 Hz), 23.7. **^{19}F NMR** (565 MHz, $CDCl_3$) δ -180.60 – -180.87 (m, 1F). **IR** ν_{max} (film): 2924, 2853, 2360, 2341, 1684, 1527, 1465, 1377, 1264, 895, 743, 706, 669 cm^{-1}. **HRMS** (ESI) m/z calcd for $C_{17}H_{21}FN_3O$ [M+H]$^+$: 302.1663; found: 302.1673.

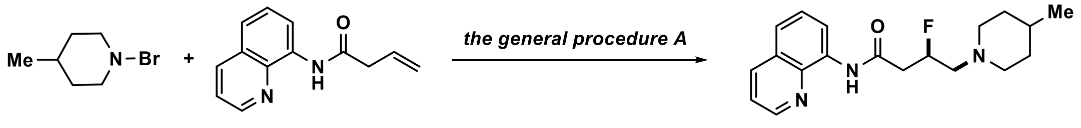

1d 2a 8c

Product **8c** was prepared following the general procedure **A**. Purification using column chromatography (PE/EA = 5:1) afforded **8c** as yellow oil (43.5 mg, 0.13 mmol, 66%). **^{1}H NMR** (600 MHz, $CDCl_3$) δ 10.00 (s, 1H), 8.81 (dd, J = 4.2, 1.8 Hz, 1H), 8.78 (dd, J = 7.2, 1.8 Hz, 1H), 8.16 (dd, J = 8.4, 1.8 Hz, 1H), 7.57 – 7.51 (m, 2H), 7.46 (dd, J = 8.4, 4.2 Hz, 1H), 5.33 – 5.22 (m, 1H), 3.00 – 2.95 (m, 2H), 2.94 – 2.91 (m, 1H), 2.90 – 2.86 (m, 1H), 2.76 (ddd, J = 19.8, 13.8, 6.0 Hz, 1H), 2.64 (ddd, J = 24.0, 13.8, 4.2 Hz, 1H), 2.14 – 2.05 (m, 2H), 1.62 – 1.55 (m, 2H), 1.30 – 1.21 (m, 3H), 0.89 (d, J = 6.6 Hz, 3H). **^{13}C NMR** (150 MHz, $CDCl_3$) δ 168.1 (d, J = 5.9 Hz), 148.4, 138.5, 136.5, 134.5, 128.1, 127.5, 121.9, 121.8, 116.8, 89.6 (d, J = 170.3 Hz), 61.9 (d, J = 21.5 Hz), 54.8 (d,

J = 5.4 Hz), 42.5 (d, J = 22.5 Hz), 34.4 (d, J = 4.4 Hz), 30.6, 22.0. **^{19}F NMR** (565 MHz, $CDCl_3$) δ -179.84 – -180.12 (m, 1F). **IR** ν_{max} (film): 3480, 2921, 2850, 2360, 2341, 1652, 1634, 1521, 1110, 749, 669 cm^{-1}. **HRMS** (ESI) m/z calcd for $C_{19}H_{25}FN_3O$ $[M+H]^+$: 330.1976; found: 330.1983.

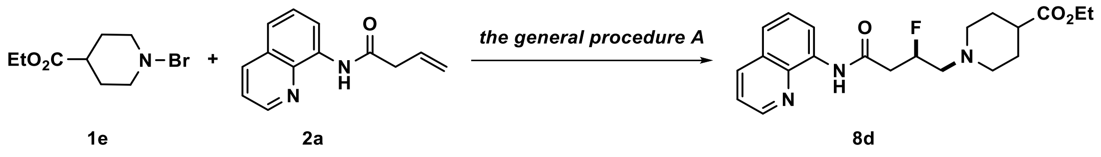

Product **8d** was prepared following the general procedure **A**. Purification using column chromatography (PE/EA = 2:1) afforded **8d** as yellow oil (59.6 mg, 0.15 mmol, 77%). **^{1}H NMR** (600 MHz, $CDCl_3$) δ 10.00 (s, 1H), 8.82 (dd, J = 4.2, 1.8 Hz, 1H), 8.77 (dd, J = 7.2, 2.4 Hz, 1H), 8.16 (dd, J = 7.8, 1.2 Hz, 1H), 7.56 – 7.51 (m, 2H), 7.46 (dd, J = 8.4, 4.2 Hz, 1H), 5.31 – 5.19 (m, 1H), 4.13 (q, J = 7.2 Hz, 2H), 3.03 – 2.93 (m, 3H), 2.92 – 2.87 (m, 1H), 2.77 (ddd, J = 20.4, 13.8, 6.6 Hz, 1H), 2.68 (ddd, J = 18.0, 14.4, 4.2 Hz, 1H), 2.31 – 2.24 (m, 1H), 2.23 – 2.17 (m, 2H), 1.93 – 1.86 (m, 2H), 1.83 – 1.78 (m, 2H), 1.25 (t, J = 6.6 Hz, 3H). **^{13}C NMR** (150 MHz, $CDCl_3$) δ 175.2, 168.0 (d, J = 6.6 Hz), 148.4, 138.5, 136.5, 134.5, 128.1, 127.5, 121.9, 121.8, 116.8, 89.7 (d, J = 170.3 Hz), 61.5 (d, J = 21.5 Hz), 60.4, 53.8, 42.3 (d, J = 22.4 Hz), 41.0, 28.4 (d, J = 5.1 Hz), 14.4. **^{19}F NMR (**475 MHz, $CDCl_3$) δ -179.66 – -179.98 (m, 1F). **IR** ν_{max} (film): 2360, 2341, 1733, 1683, 1652, 1521, 1489, 1457, 1275, 1042, 749, 669, 650, 418 cm^{-1}. **HRMS** (ESI) m/z calcd for $C_{21}H_{27}FN_3O_3$ $[M+H]^+$: 388.2031; found: 388.2037.

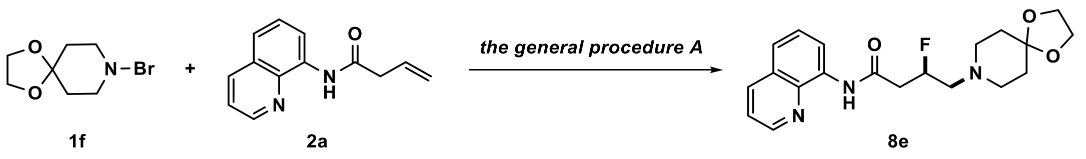

Product **8e** was prepared following the general procedure **A**. Purification using column chromatography (PE/EA = 5:1) afforded **8e** as yellow oil (60.5 mg, 0.16 mmol, 81%). **^{1}H NMR** (600 MHz, $CDCl_3$) δ 10.01 (s, 1H), 8.81 (dd, J = 4.2, 1.8 Hz, 1H), 8.77 (dd, J = 7.2, 1.8 Hz, 1H), 8.16 (dd, J = 7.8, 1.2 Hz, 1H), 7.56 – 7.50 (m, 2H), 7.46 (dd, J = 8.4, 4.2 Hz, 1H), 5.31 – 5.21 (m, 1H), 3.96 – 3.93 (m, 4H), 3.04 – 2.93 (m, 2H), 2.81 (ddd, J = 20.4, 13.8, 6.0 Hz, 1H), 2.76 – 2.71 (m, 1H), 2.70 – 2.66 (m, 2H), 2.66 – 2.59 (m, 2H), 1.80– 1.74 (m, 4H). **^{13}C NMR** (150 MHz, $CDCl_3$) δ 168.0 (d, J = 6.2 Hz), 148.4, 138.5, 136.5, 134.5, 128.1, 127.5, 121.9, 121.8, 116.8, 107.1, 89.8 (d, J = 170.4 Hz), 64.3, 60.9 (d, J = 21.5 Hz), 52.3, 42.3 (d, J = 22.4 Hz), 34.9. **^{19}F NMR** (565 MHz, $CDCl_3$) δ -180.04 – -180.40 (m, 1F). **IR** ν_{max} (film): 3350, 2954, 2928, 2821, 2360, 2341, 1684,

1525, 1486, 1425, 1385, 1364, 1144, 1096, 1039, 827, 792, 758 cm^{-1}. **HRMS** (ESI) m/z calcd for $C_{20}H_{25}FN_3O_3$ $[M+H]^+$: 374.1874; found: 374.1880.

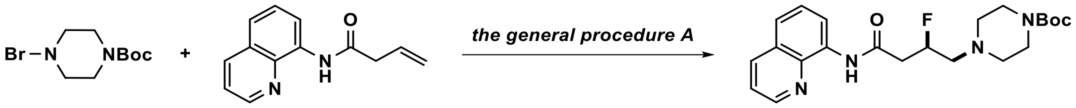

Product **8f** was prepared following the general procedure **A**. Purification using column chromatography (PE/EA = 5:1) afforded **8f** as yellow oil (69.1 mg, 0.17 mmol, 83%). **^{1}H NMR** (600 MHz, $CDCl_3$) δ 9.98 (s, 1H), 8.80 (d, J = 4.2 Hz, 1H), 8.76 (d, J = 7.2 Hz, 1H), 8.15 (d, J = 8.4 Hz, 1H), 7.55 – 7.49 (m, 2H), 7.45 (dd, J = 8.4, 4.2 Hz, 1H), 5.33 – 5.20 (m, 1H), 3.48 – 3.40 (m, 4H), 3.00 (dd, J = 5.4, 3.0 Hz, 1H), 2.96 (d, J = 6.0 Hz, 1H), 2.78 (ddd, J = 20.4, 13.8, 6.0 Hz, 1H), 2.70 (ddd, J = 18.0, 13.8, 4.2 Hz, 1H), 2.58 – 2.52 (m, 2H), 2.51 – 2.45 (m, 2H), 1.46 (s, 9H). **^{13}C NMR** (150 MHz, $CDCl_3$) δ 167.8 (d, J = 6.9 Hz), 154.8, 148.3, 138.4, 136.4, 134.3, 128.0, 127.4, 121.9, 121.7, 116.8, 89.5 (d, J = 170.6 Hz), 79.7, 61.3 (d, J = 21.3 Hz), 53.7, 43.6, 42.1 (d, J = 22.7 Hz), 28.5. **^{19}F NMR (**475 MHz, $CDCl_3$) δ -180.05 – -180.40 (m, 1F). **IR** ν_{max} (film): 2926, 2854, 2359, 2341, 1686, 1527, 1486, 1326, 1168, 826, 757 cm^{-1}. **HRMS** (ESI) m/z calcd for $C_{22}H_{30}FN_4O_3$ $[M+H]^+$: 417.2296; found: 417.2296.

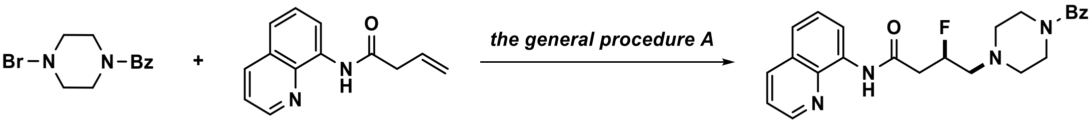

Product **8g** was prepared following the general procedure **A**. Purification using column chromatography (PE/EA = 5:1) afforded **8g** as yellow oil (67.2 mg, 0.16 mmol, 80%). **^{1}H NMR** (600 MHz, $CDCl_3$) δ 9.98 (s, 1H), 8.81 (dd, J = 4.2, 1.8 Hz, 1H), 8.76 (dd, J = 6.6, 3.0 Hz, 1H), 8.17 (d, J = 8.4 Hz, 1H), 7.51 – 7.55 (m, 2H), 7.46 (dd, J = 8.4, 4.2 Hz, 1H), 7.43 – 7.35 (m, 5H), 5.35 – 5.21 (m, 1H), 3.80 (br, 2H), 3.43 (br, 2H), 3.00 (d, J = 6.0 Hz, 1H), 2.97 (d, J = 6.0 Hz, 1H), 2.86 – 2.79 (m, 1H), 2.78 – 2.71 (m, 1H), 2.68 – 2.48 (m, 4H). **^{13}C NMR** (150 MHz, $CDCl_3$) δ 170.4, 167.7 (d, J = 7.5 Hz), 148.4, 138.4, 136.6, 135.9, 134.4, 129.8, 128.6, 128.1, 127.5, 127.2, 122.0, 121.8, 116.8, 89.6 (d, J = 170.6 Hz), 61.2 (d, J = 21.3 Hz), 53.9, 47.8, 42.2 (d, J = 22.7 Hz). **^{19}F NMR (**475 MHz, $CDCl_3$) δ -180.02 – -180.41 (m, 1F). **IR** ν_{max} (film): 2955, 2924, 2853, 2360, 2341, 1684, 1525, 1486, 1325, 1259, 1159, 826, 697 cm^{-1}. **HRMS** (ESI) m/z calcd for $C_{24}H_{26}FN_4O_2$ $[M+H]^+$: 421.2034; found: 421.2033.

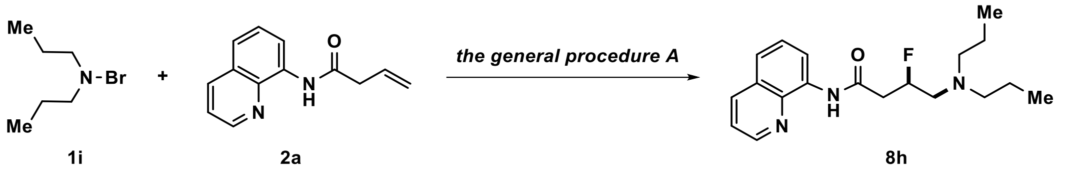

Product **8h** was prepared following the general procedure **A**. Purification using column chromatography (PE/EA = 5:1) afforded **8h** as yellow oil (36.4 mg, 0.11 mmol, 55%). **^{1}H NMR** (600 MHz, $CDCl_3$) δ 9.99 (s, 1H), 8.80 (d, J = 4.2 Hz, 1H), 8.78 (d, J = 6.6 Hz, 1H), 8.15 (d, J = 8.4 Hz, 1H), 7.55 – 7.49 (m, 2H), 7.44 (dd, J = 8.4, 4.2 Hz, 1H), 5.32 – 5.13 (m, 1H), 3.02 – 2.88 (m, 2H), 2.86 – 2.70 (m, 2H), 2.52 – 2.45 (m, 4H), 1.51 – 1.44 (m, 4H), 0.88 (t, J = 7.2 Hz, 6H). **^{13}C NMR** (150 MHz, $CDCl_3$) δ 168.4 (d, J = 4.2 Hz), 148.3, 138.4, 136.4, 134.5, 128.0, 127.4, 121.8, 121.7, 116.7, 90.2 (d, J = 169.5 Hz), 57.7 (d, J = 22.1 Hz), 57.1, 42.4 (d, J = 22.2 Hz), 20.4, 11.9. **^{19}F NMR (**475 MHz, $CDCl_3$) δ -181.42 – -181.95 (m, 1F). **IR** ν_{max} (film): 2958, 2926, 2360, 2341, 1684, 1526, 1486, 1457, 1378, 1325, 826, 791, 755 cm^{-1}. **HRMS** (ESI) m/z calcd for $C_{19}H_{27}FN_3O$ $[M+H]^+$: 332.2133; found: 332.2137.

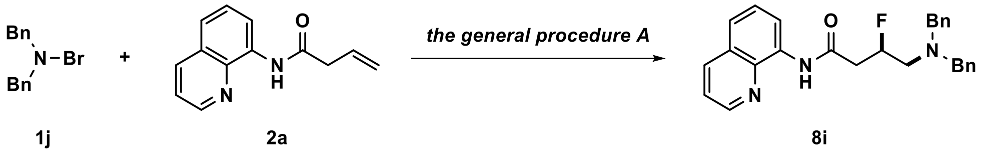

Product **8i** was prepared following the general procedure **A**. Purification using column chromatography (PE/EA = 5:1) afforded **8i** as yellow oil (66.6 mg, 0.16 mmol, 78%). **^{1}H NMR** (600 MHz, $CDCl_3$) δ 9.90 (s, 1H), 8.81 (dd, J = 4.2, 1.8 Hz, 1H), 8.75 (dd, J = 7.2, 2.4 Hz, 1H), 8.16 (dd, J = 8.4, 1.8 Hz, 1H), 7.55 – 7.50 (m, 2H), 7.45 (dd, J = 8.4, 4.2 Hz, 1H), 7.37 (d, J = 7.2 Hz, 4H), 7.28 (t, J = 7.8 Hz, 4H), 7.21 (t, J = 7.2 Hz, 2H), 5.38 – 5.20 (m, 1H), 3.73 – 3.70 (m, 2H), 3.69 – 3.66 (m, 2H) , 2.90 – 2.84 (m, 1H), 2.84 – 2.74 (m, 3H). **^{13}C NMR** (150 MHz, $CDCl_3$) δ 168.2 (d, J = 4.1 Hz), 148.4, 139.1, 138.5, 136.5, 134.5, 129.1, 128.4, 128.1, 127.5, 127.2, 121.85, 121.76, 116.8, 90.2 (d, J = 169.8 Hz), 59.2, 56.4 (d, J = 21.8 Hz), 42.3 (d, J = 22.2 Hz). **^{19}F NMR (**475 MHz, $CDCl_3$) δ -181.08 – -181.45 (m, 1F). **IR** ν_{max} (film): 2924, 2851, 2360, 2341, 1770, 1685, 1527, 1486, 1456, 1375, 1326, 1245, 1050, 913, 825, 791, 746, 698 cm^{-1}. **HRMS** (ESI) m/z calcd for $C_{27}H_{26}FN_3NaO$ $[M+Na]^+$: 450.1952; found: 450.1957.

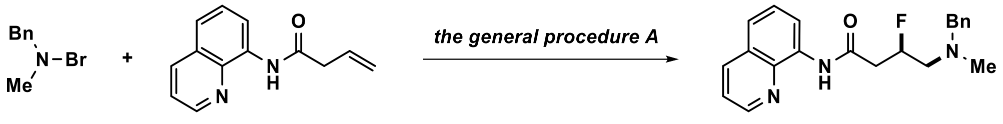

Product **8j** was prepared following the general procedure **A**. Purification using column chromatography (PE/EA = 5:1) afforded **8j** as yellow oil (52.7 mg, 0.15 mmol, 75%). **^{1}H NMR** (600 MHz, $CDCl_3$) δ 10.02 (s, 1H), 8.80 – 8.75 (m, 2H), 8.15 (dd, J = 7.8, 1.2 Hz, 1H), 7.56 – 7.48 (m, 2H), 7.44 (dd, J = 8.4, 4.2 Hz, 1H), 7.34 (d, J = 6.6 Hz, 2H), 7.28 (t, J = 7.2 Hz, 2H), 7.22 (t, J = 7.8 Hz, 1H), 5.35 – 5.20 (m, 1H), 3.64 (d, J = 13.2 Hz, 1H), 3.58 (d, J = 13.2 Hz, 1H), 2.97 – 2.93 (m 1H), 2.91 (d, J = 6.0 Hz, 1H), 2.81 (ddd, J = 20.4, 13.8, 6.0 Hz, 1H), 2.72 (ddd, J = 18.0, 13.2, 4.2 Hz, 1H), 2.33 (s, 3H). **^{13}C NMR** (150 MHz, $CDCl_3$) δ 168.2 (d, J = 5.3 Hz), 148.3, 138.8, 138.5, 136.4, 134.5, 129.1, 128.4, 128.1, 127.5, 127.2, 121.9, 121.7, 116.8, 90.0 (d, J = 170.0 Hz), 63.0, 60.1 (d, J = 21.8 Hz), 43.4, 42.3 (d, J = 22.4 Hz). **^{19}F NMR (**475 MHz, $CDCl_3$) δ -180.95 – -181.30 (m, 1F). **IR** ν_{max} (film): 3347, 2954, 2925, 2850, 2360, 2341, 1686, 1527, 1486, 1455, 1425, 1385, 1326, 1022, 826, 792, 741, 669 cm^{-1}. **HRMS** (ESI) m/z calcd for $C_{21}H_{23}FN_3O$ [M+H]$^+$: 352.1820; found: 352.1827.

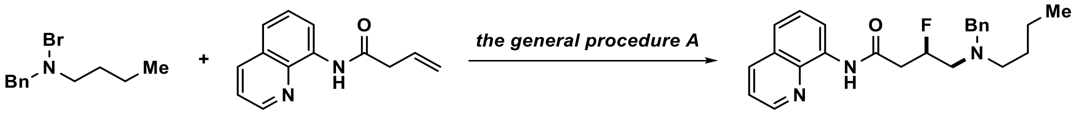

Product **8k** was prepared following the general procedure **A**. Purification using column chromatography (PE/EA = 5:1) afforded **8k** as yellow oil (44.0 mg, 0.11 mmol, 56%). **^{1}H NMR** (600 MHz, $CDCl_3$) δ 9.94 (s, 1H), 8.80 (dd, J = 4.2, 1.8 Hz, 1H), 8.77 (dd, J = 7.2, 1.8 Hz, 1H), 8.15 (dd, J = 7.8, 1.8 Hz, 1H), 7.55 – 7.49 (m, 2H), 7.45 (dd, J = 8.4, 4.2 Hz, 1H), 7.34 (d, J = 7.2 Hz, 2H), 7.28 (t, J = 7.8 Hz, 2H), 7.21 (t, J = 7.2 Hz, 1H), 5.31 – 5.15 (m, 1H), 3.67 (s, 2H), 2.94 – 2.86 (m, 1H), 2.86 – 2.80 (m, 2H), 2.76 (ddd, J = 18.6, 13.8, 4.8 Hz, 1H), 2.54 (td, J = 7.2, 4.2 Hz, 2H), 1.51 – 1.43 (m, 2H), 1.33 – 1.26 (m, 2H), 0.85 (t, J = 7.2 Hz, 3H). **^{13}C NMR** (150 MHz, $CDCl_3$) δ 168.3 (d, J = 4.4 Hz), 148.3, 139.6, 138.5, 136.4, 134.5, 129.0, 128.3, 128.0, 127.5, 127.0, 121.8, 121.7, 116.8, 90.3 (d, J = 169.5 Hz), 59.5, 57.0 (d, J = 22.2 Hz), 54.6, 42.4 (d, J = 22.2 Hz), 29.4, 20.6, 14.2. **^{19}F NMR (**475 MHz, $CDCl_3$) δ -181.20 – -181.52 (m, 1F). **IR** ν_{max} (film): 2955, 2923, 2851, 2360, 2341, 1684, 1526, 1486, 1457, 1424, 1326, 825, 791, 669 cm^{-1}. **HRMS** (ESI) m/z calcd for $C_{24}H_{29}FN_3O$ [M+H]$^+$: 394.2289; found: 394.2284.

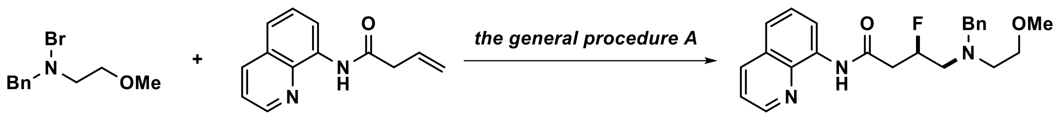

Product **8l** was prepared following the general procedure **A**. Purification using column chromatography (PE/EA = 5:1) afforded **8l** as yellow oil (42.7 mg, 0.11 mmol, 54%). **^{1}H NMR** (600 MHz, $CDCl_3$) δ 9.96 (s, 1H), 8.81 (dd, J = 4.2, 1.2 Hz, 1H), 8.77 (dd, J = 7.2, 1.8 Hz, 1H), 8.16 (dd, J = 8.4, 1.8 Hz, 1H), 7.56 – 7.49 (m, 2H), 7.45 (dd, J = 8.4, 4.2 Hz, 1H), 7.35 (d, J = 7.2 Hz, 2H), 7.28 (t, J = 7.2 Hz, 2H), 7.21 (t, J = 7.2 Hz, 1H), 5.32 – 5.17 (m, 1H), 3.79 (d, J = 13.8 Hz, 1H), 3.75 (d, J = 13.8 Hz, 1H), 3.49 (t, J = 6.0 Hz, 2H), 3.30 (s, 3H), 2.99 – 2.92 (m, 2H), 2.92 – 2.84 (m, 2H), 2.83 – 2.75 (m, 2H). **^{13}C NMR** (150 MHz, $CDCl_3$) δ 168.3 (d, J = 4.7 Hz), 148.4, 139.3, 138.5, 136.5, 134.5, 129.1, 128.4, 128.1, 127.5, 127.2, 121.82, 121.76, 116.8, 90.5 (d, J = 169.5 Hz), 71.6, 60.2, 58.9, 57.6 (d, J = 21.8 Hz), 54.0, 42.2 (d, J = 22.2 Hz). **^{19}F NMR** (565 MHz, $CDCl_3$) δ -181.25 – -181.55 (m, 1F). **IR** ν_{max} (film): 2954, 2916, 2848, 2360, 2341, 1683, 1526, 1486, 1462, 1376, 1263, 1102, 825, 743, 669 cm^{-1}. **HRMS** (ESI) m/z calcd for $C_{23}H_{27}FN_3O_2$ [M+H]$^+$: 396.2082; found: 396.2086.

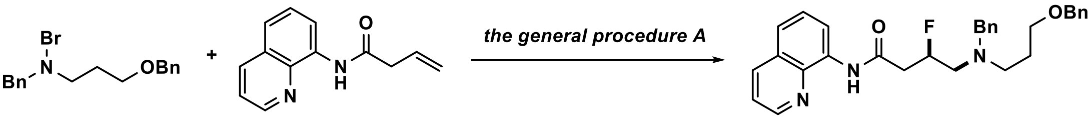

Product **8m** was prepared following the general procedure **A**. Purification using column chromatography (PE/EA = 7:1) afforded **8m** as yellow oil (58.2 mg, 0.12 mmol, 60%). **^{1}H NMR** (600 MHz, $CDCl_3$) δ 9.94 (s, 1H), 8.80 (dd, J = 4.2, 1.2 Hz, 1H), 8.77 (dd, J = 7.2, 2.4 Hz, 1H), 8.15 (dd, J = 8.4, 1.8 Hz, 1H), 7.55 – 7.50 (m, 2H), 7.45 (dd, J = 8.4, 4.2 Hz, 1H), 7.34 – 7.26 (m, 8H), 7.25 – 7.18 (m, 2H), 5.28 – 5.16 (m, 1H), 4.43 (s, 2H), 3.72 – 3.64 (m, 2H), 3.54 – 3.47 (m, 2H), 2.90 – 2.82 (m, 3H), 2.77 (ddd, J = 19.2, 14. 4, 4.8 Hz, 1H), 2.71 – 2.63 (m, 2H), 1.86 – 1.78 (m, 2H). **^{13}C NMR** (150 MHz, $CDCl_3$) δ 168.2 (d, J = 4.5 Hz), 148.4, 139.3, 138.7, 138.5, 136.4, 134.5, 129.1, 128.44, 128.38, 128.1, 127.8, 127.6, 127.5, 127.1, 121.82, 121.76, 116.8, 90.2 (d, J = 169.8 Hz), 73.0, 68.5, 59.5, 57.1 (d, J = 21.9 Hz), 51.7, 42.3 (d, J = 22.2 Hz), 27.7. **^{19}F NMR (**475 MHz, $CDCl_3$) δ -181.20 – -181.48 (m, 1F). **IR** ν_{max} (film): 2921, 2850, 2360, 2341, 1683, 1525, 1487, 1456, 1261, 1100, 748, 669 cm^{-1}. **HRMS** (ESI) m/z calcd for $C_{30}H_{33}FN_3O_2$ [M+H]$^+$: 486.2551; found: 486.2542.

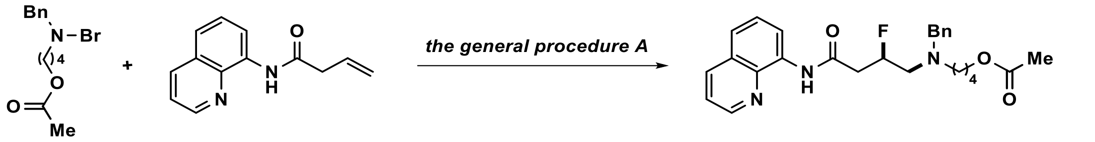

1o 2a 8n

Product **8n** was prepared following the general procedure **A**. Purification using column chromatography (PE/EA = 7:1) afforded **8n** as yellow oil (43.3 mg, 0.10 mmol, 48%). **^{1}H NMR** (600 MHz, $CDCl_3$) δ 9.94 (s, 1H), 8.81 (dd, J = 4.2, 1.2 Hz, 1H), 8.76 (dd, J = 7.2, 2.4 Hz, 1H), 8.16 (d, J = 8.4 Hz, 1H), 7.56 – 7.51 (m, 2H), 7.46 (dd, J = 8.4, 4.2 Hz, 1H), 7.34 (d, J = 7.8 Hz, 2H), 7.28 (t, J = 7.8 Hz, 2H), 7.21 (t, J = 7.2 Hz, 1H), 5.31 – 5.19 (m, 1H), 4.00 (t, J = 6.6 Hz, 2H), 3.72 – 3.66 (m, 2H), 2.89 (d, J = 6.0 Hz, 1H), 2.87 – 2.75 (m, 3H), 2.63 – 2.55 (m, 2H), 2.01 (s, 3H), 1.65 – 1.61 (m, 2H), 1.58 – 1.53 (m, 2H). **^{13}C NMR** (150 MHz, $CDCl_3$) δ 171.3, 168.1, 148.4, 139.3, 138.5, 136.5, 134.5, 129.1, 128.4, 128.1, 127.5, 127.2, 121.9, 121.8, 116.8, 90.2 (d, J = 168.5 Hz), 64.5, 59.5, 57.1 (d, J = 21.8 Hz), 54.4, 42.3 (d, J = 22.4 Hz), 26.5, 23.8, 21.1. **^{19}F NMR (**475 MHz, $CDCl_3$) δ -181.25 – -181.70 (m, 1F). **IR** ν_{max} (film): 2954, 2925, 2853, 2360, 2341, 1733, 1683, 1525, 1486, 1456, 1385, 1325, 1240, 1026, 826, 792, 698, 669 cm^{-1}. **HRMS** (ESI) m/z calcd for $C_{26}H_{31}FN_3O_3$ $[M+H]^+$: 452.2344; found: 452.2349.

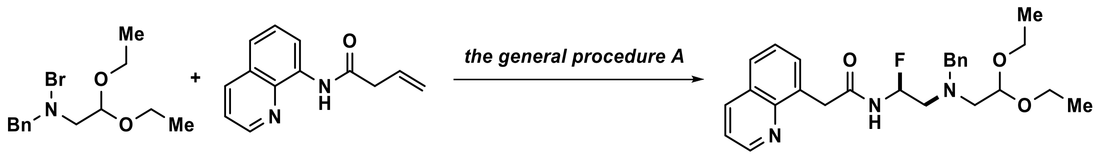

1p 2a 8o

Product **8o** was prepared following the general procedure **A**. Purification using column chromatography (PE/EA = 6:1) afforded **8o** as yellow oil (48.0 mg, 0.11 mmol, 53%). **^{1}H NMR** (600 MHz, $CDCl_3$) δ 9.95 (s, 1H), 8.81 (dd, J = 4.2, 1.2 Hz, 1H), 8.77 (dd, J = 7.2, 1.8 Hz, 1H), 8.16 (dd, J = 7.8, 1.2 Hz, 1H), 7.55 – 7.50 (m, 2H), 7.45 (dd, J = 8.4, 4.2 Hz, 1H), 7.35 (d, J = 7.2 Hz, 2H), 7.28 (d, J = 7.8 Hz, 2H), 7.20 (t, J = 7.8 Hz, 1H), 5.30 – 5.20 (m, 1H), 4.57 (t, J = 5.4 Hz, 1H), 3.86 – 3.79 (m, 2H), 3.65 – 3.59 (m, 2H), 3.52 – 3.45 (m, 2H), 3.02 – 2.92 (m, 2H), 2.92 – 2.90 (m, 1H), 2.87 (d, J = 6.0 Hz, 1H), 2.80 (dd, J = 13.8, 5.4 Hz, 1H), 2.75 (dd, J = 14.4, 5.4 Hz, 1H), 1.21 – 1.15 (m, 6H). **^{13}C NMR** (150 MHz, $CDCl_3$) δ 168.3 (d, J = 3.9 Hz), 148.3, 139.4, 138.5, 136.5, 134.5, 129.1, 128.4, 128.1, 127.5, 127.2, 121.81, 121.75, 116.8, 102.5, 90.6 (d, J = 169.8 Hz), 62.3 (d, J = 18.9 Hz), 60.6, 58.0 (d, J = 21.9 Hz), 57.6, 42.2 (d, J = 22.1 Hz), 15.5. **^{19}F NMR (**475 MHz, $CDCl_3$) δ -181.65 – -182.00 (m, 1F). **IR** ν_{max} (film): 3463, 2954, 2922, 2850, 2360, 2341, 1635, 1526, 1487, 1457, 1376, 1326, 1099, 750, 669 cm^{-1}. **HRMS** (ESI) m/z calcd for $C_{26}H_{33}FN_3O_3$ $[M+H]^+$: 454.2500; found: 454.2513.

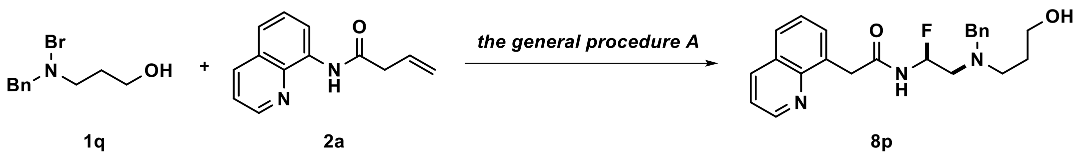

Product **8p** was prepared following the general procedure **A**. Purification using column chromatography (PE/EA = 3:1) afforded **8p** as yellow oil (34.0 mg, 0.09 mmol, 43%). **^{1}H NMR** (600 MHz, $CDCl_3$) δ 9.92 (s, 1H), 8.81 (dd, J = 4.2, 1.8 Hz, 1H), 8.75 (dd, J = 6.6, 2.4 Hz, 1H), 8.17 (dd, J = 8.4, 1.8 Hz, 1H), 7.56 – 7.51 (m, 2H), 7.46 (dd, J = 8.4, 4.2 Hz, 1H), 7.32 (d, J = 6.6 Hz, 2H), 7.29 (t, J = 7.2 Hz, 2H), 7.22 (t, J = 7.2 Hz, 1H), 5.39 – 5.25 (m, 1H), 3.95 (br, 1H), 3.80 – 3.75 (m, 2H), 3.76 (d, J = 4.2 Hz, 1H), 3.65 (d, J = 13.2 Hz, 1H), 2.96 – 2.86 (m, 2H), 2.84 – 2.79 (m, 2H), 2.78 – 2.71 (m, 2H), 1.84 – 1.69 (m, 2H). **^{13}C NMR** (150 MHz, $CDCl_3$) δ 167.9 (d, J = 5.0 Hz), 148.4, 138.4, 138.3, 136.5, 134.3, 129.3, 128.6, 128.1, 127.49, 127.46, 122.0, 121.8, 116.9, 89.5 (d, J = 170.6 Hz), 63.0, 59.4, 57.1 (d, J = 21.0 Hz), 53.7, 42.3 (d, J = 22.7 Hz), 28.8. **^{19}F NMR (**475 MHz, $CDCl_3$) δ -181.59 – -181.91 (m, 1F). **IR** ν_{max} (film): 3467, 2954, 2924, 2852, 2359, 2341, 1739, 1683, 1652, 1526, 1486, 1374, 1241, 1047, 749, 669 cm^{-1}. **HRMS** (ESI) m/z calcd for $C_{23}H_{27}FN_3O_2$ $[M+H]^+$: 396.2082; found: 396.2088.

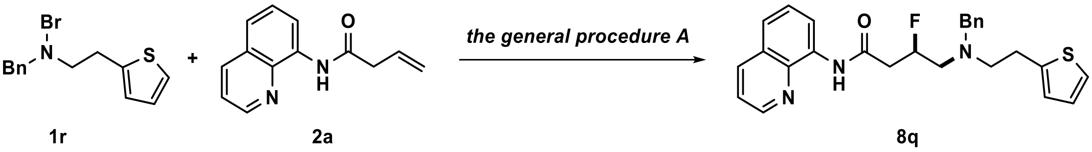

Product **8q** was prepared following the general procedure **A**. Purification using column chromatography (PE/EA = 7:1) afforded **8q** as yellow oil (75.1 mg, 0.17 mmol, 84%). **^{1}H NMR** (600 MHz, $CDCl_3$) δ 9.89 (s, 1H), 8.82 (dd, J = 4.2, 1.8 Hz, 1H), 8.75 (dd, J = 6.6, 1.8 Hz, 1H), 8.16 (dd, J = 8.4, 1.8 Hz, 1H), 7.56 – 7.51 (m, 2H), 7.46 (dd, J = 8.4, 4.2 Hz, 1H), 7.33 (d, J = 7.2 Hz, 2H), 7.27 (d, J = 7.2 Hz, 2H), 7.21 (t, J = 7.2 Hz, 1H), 7.11 (d, J = 5.4 Hz, 1H), 6.91 (dd, J = 4.8, 3.0 Hz, 1H), 6.79 (d, J = 3.0 Hz, 1H), 5.28 – 5.17 (m, 1H), 3.80 (d, J = 13.8 Hz, 1H), 3.73 (d, J = 13.8 Hz, 1H), 3.03 (t, J = 7.2 Hz, 2H), 2.99 – 2.90 (m, 2H), 2.90 – 2.82 (m, 2H), 2.81 – 2.73 (m, 2H). **^{13}C NMR** (150 MHz, $CDCl_3$) δ 168.2 (d, J = 4.8 Hz), 148.4, 142.9, 139.0, 138.5, 136.5, 134.5, 129.1, 128.5, 128.1, 127.5, 127.3, 126.9, 125.0, 123.5, 121.85, 121.77, 116.9, 90.4 (d, J = 169.8 Hz), 59.4, 57.0 (d, J = 21.9 Hz), 56.8, 42.1 (d, J = 22.4 Hz), 28.1. **^{19}F NMR (**475 MHz, $CDCl_3$) δ -180.84 – -181.16 (m, 1F). **IR** ν_{max} (film): 2924, 2853, 2360, 2122, 1770, 1684, 1525, 1484, 1458, 1381, 1323, 1244, 1164, 1057, 912, 825, 790, 736, 699 cm^{-1}. **HRMS** (ESI) m/z calcd for $C_{26}H_{27}FN_3OS$ $[M+H]^+$: 448.1853; found: 448.1851.

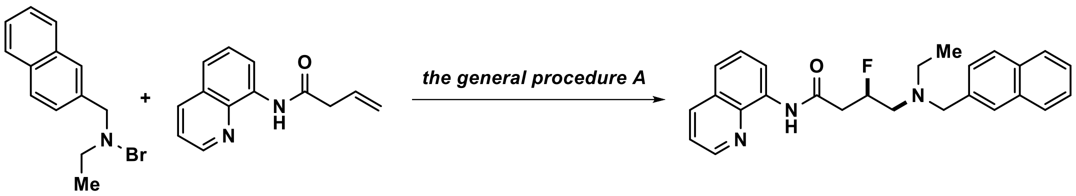

Product **8r** was prepared following the general procedure **A**. Purification using column chromatography (PE/EA = 7:1) afforded **8r** as yellow oil (71.4 mg, 0.17 mmol, 86%). **^{1}H NMR** (600 MHz, $CDCl_3$) δ 9.95 (s, 1H), 8.78 (d, J = 4.2 Hz, 1H), 8.75 (d, J = 6.6 Hz, 1H), 8.15 (d, J = 8.4 Hz, 1H), 7.80 – 7.76 (m, 2H), 7.75 (d, J = 4.8 Hz, 2H), 7.54 (d, J = 8.4 Hz, 1H), 7.54 – 7.48 (m, 2H), 7.45 (d, J = 3.6 Hz, 1H), 7.44 – 7.40 (m, 2H), 5.34 – 5.23 (m, 1H), 3.85 (s, 2H), 2.95 – 2.87 (m, 3H), 2.86 – 2.80 (m, 1H), 2.68 (q, J = 7.2 Hz, 2H), 1.09 (t, J = 7.2 Hz, 3H). **^{13}C NMR** (150 MHz, $CDCl_3$) δ 168.3 (d, J = 5.0 Hz), 148.3, 138.5, 137.1, 136.4, 134.5, 133.5, 132.9, 128.1, 127.82, 127.75, 127.5, 127.4, 126.0, 125.6, 121.8, 121.7, 116.8, 90.3 (d, J = 169.8 Hz), 59.2, 56.4 (d, J = 22.1 Hz), 48.5, 42.3 (d, J = 22.5 Hz), 11.9. **^{19}F NMR (**475 MHz, $CDCl_3$) δ -181.40 – -181.75 (m, 1F). **IR** ν_{max} (film): 3347, 2958, 2925, 2853, 2359, 2341, 1685, 1526, 1486, 1458, 1424, 1325, 1260, 1166, 1117, 825, 791, 669 cm^{-1}. **HRMS** (ESI) m/z calcd for $C_{26}H_{27}FN_3O$ $[M+H]^+$: 416.2133; found: 416.2142.

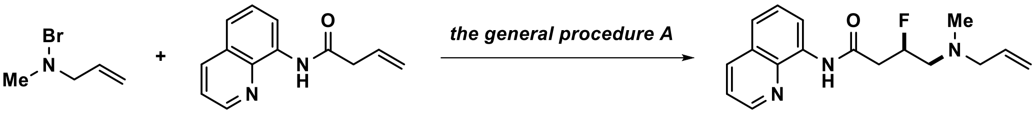

Product **8s** was prepared following the general procedure **A**. Purification using column chromatography (PE/EA = 5:1) afforded **8s** as yellow oil (48.8 mg, 0.16 mmol, 81%). **^{1}H NMR** (600 MHz, $CDCl_3$) δ 10.02 (s, 1H), 8.81 (dd, J = 4.2, 1.2 Hz, 1H), 8.78 (dd, J = 7.2, 1.8 Hz, 1H), 8.16 (dd, J = 8.4, 1.8 Hz, 1H), 7.57 – 7.49 (m, 2H), 7.46 (dd, J = 8.4, 4.2 Hz, 1H), 5.88 (ddt, J = 16.8, 9.6, 6.0 Hz, 1H), 5.34 – 5.22 (m, 1H), 5.22 – 5.18 (m, 1H), 5.16 – 5.13 (m, 1H), 3.15 (dd, J = 13.8, 6.6 Hz, 1H), 3.09 (dd, J = 13.8, 6.6 Hz, 1H), 2.99 – 2.95 (m, 1H), 2.94 – 2.92 (m, 1H), 2.81 (ddd, J = 19.8, 13.8, 6.6 Hz, 1H), 2.69 (ddd, J = 17.4, 13.8, 3.6 Hz, 1H), 2.36 (s, 3H). **^{13}C NMR** (150 MHz, $CDCl_3$) δ 168.1 (d, J = 5.0 Hz), 148.4, 138.5, 136.5, 135.4, 134.5, 128.1, 127.5, 121.9, 121.8, 118.1, 116.8, 89.7 (d, J = 170.4 Hz), 61.6, 59.8 (d, J = 21.3 Hz), 43.2, 42.3 (d, J = 22.4 Hz). **^{19}F NMR (**475 MHz, $CDCl_3$) δ -180.96 – -181.24 (m, 1F). **IR** ν_{max} (film): 2920, 2850, 2359, 2341, 1683, 1521, 1488, 1456, 1417, 1375, 750, 669 cm^{-1}. **HRMS** (ESI) m/z calcd for $C_{17}H_{21}FN_3O$ $[M+H]^+$: 302.1663; found: 302.1655.

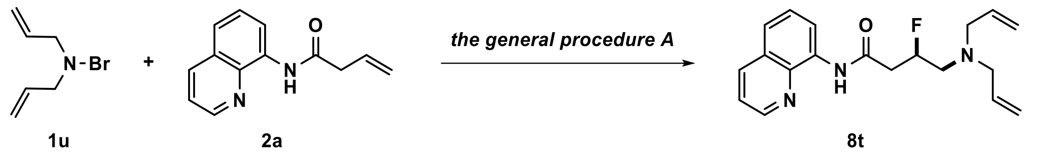

Product **8t** was prepared following the general procedure **A**. Purification using column chromatography (PE/EA = 6:1) afforded **8t** as yellow oil (57.6 mg, 0.18 mmol, 88%). **^{1}H NMR** (600 MHz, $CDCl_3$) δ 9.99 (s, 1H), 8.80 (dd, J = 4.2, 1.8 Hz, 1H), 8.77 (dd, J = 7.2, 1.2 Hz, 1H), 8.14 (dd, J = 7.8, 1.8 Hz, 1H), 7.54 – 7.49 (m, 2H), 7.44 (dd, J = 8.4, 4.2 Hz, 1H), 5.86 (ddt, J = 16.8, 10.2, 6.6 Hz, 2H), 5.32 – 5.21 (m, 1H), 5.21 – 5.17 (m, 2H), 5.13 (dd, J = 10.2, 1.2 Hz, 2H), 3.22 (dd, J = 13.8, 6.0 Hz, 2H), 3.18 (dd, J = 13.8, 6.6 Hz, 2H), 2.94 (dd, J = 5.4, 3.6 Hz, 1H), 2.90 (d, J = 6.0 Hz, 1H), 2.85 (ddd, J = 19.8, 13.8, 6.0 Hz, 1H), 2.77 (ddd, J = 18.6, 14.4, 4.8 Hz, 1H). **^{13}C NMR** (150 MHz, $CDCl_3$) δ 168.2 (d, J = 4.7 Hz), 148.3, 138.4, 136.4, 135.5, 134.5, 128.0, 127.4, 121.8, 121.7, 118.0, 116.7, 90.1 (d, J = 169.7 Hz), 57.9, 56.1 (d, J = 21.9 Hz), 42.3 (d, J = 22.4 Hz). **^{19}F NMR (**475 MHz, $CDCl_3$) δ -181.28 – -181.56 (m, 1F). **IR** ν_{max} (film): 3459, 2956, 2928, 2859, 2360, 2341, 1688, 1638, 1528, 1486, 1463, 1385, 1326, 826, 791, 756 cm^{-1}. **HRMS** (ESI) m/z calcd for $C_{19}H_{23}FN_3O$ [M+H]$^+$: 328.1820; found: 328.1829.

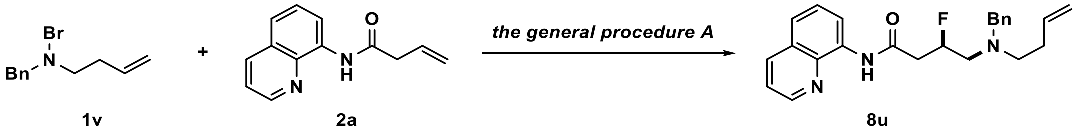

Product **8u** was prepared following the general procedure **A**. Purification using column chromatography (PE/EA = 8:1) afforded **8u** as yellow oil (61.8 mg, 0.16 mmol, 79%). **^{1}H NMR** (600 MHz, $CDCl_3$) δ 9.94 (s, 1H), 8.81 (dd, J = 4.2, 1.8 Hz, 1H), 8.76 (dd, J = 7.2, 1.8 Hz, 1H), 8.16 (dd, J = 7.8, 1.2 Hz, 1H), 7.56 – 7.48 (m, 2H), 7.45 (dd, J = 8.4, 4.2 Hz, 1H), 7.34 (d, J = 7.2 Hz, 2H), 7.28 (t, J = 7.2 Hz, 2H), 7.21 (t, J = 7.2 Hz, 1H), 5.79 (ddt, J = 16.8, 10.2, 6.6 Hz, 1H), 5.31 – 5.16 (m, 1H), 5.03 (dd, J = 17.4, 1.8 Hz, 1H), 4.99 (d, J = 10.2 Hz, 1H), 3.71 (s, 2H), 2.91 – 2.84 (m, 3H), 2.83 – 2.76 (m, 1H), 2.66 (td, J = 7.2, 4.8 Hz, 2H), 2.31 – 2.24 (m, 2H). **^{13}C NMR** (150 MHz, $CDCl_3$) δ 168.3 (d, J = 4.7 Hz), 148.4, 139.3, 138.5, 136.8, 136.5, 134.5, 129.1, 128.4, 128.1, 127.5, 127.2, 121.83, 121.76, 116.8, 115.9, 90.3 (d, J = 170.0 Hz), 59.4, 56.9 (d, J = 22.1 Hz), 54.3, 42.3 (d, J = 22.2 Hz), 31.8. **^{19}F NMR** (565 MHz, $CDCl_3$) δ -181.21 – -181.47 (m, 1F). **IR** ν_{max} (film):2917, 2849, 2360, 2341, 1683, 1653, 1525, 1488, 1472, 1457, 1386, 1326, 1260, 826, 791, 749, 699 cm^{-1}. **HRMS** (ESI) m/z calcd for $C_{24}H_{27}FN_3O$ [M+H]$^+$: 392.2133; found: 392.2131.

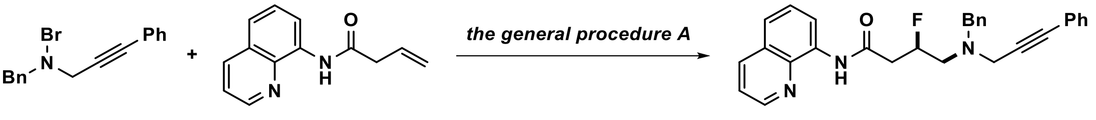

1w **2a** **8v**

Product **8v** was prepared following the general procedure **A**. Purification using column chromatography (PE/EA = 8:1) afforded **8v** as yellow oil (73.1 mg, 0.16 mmol, 81%). **^{1}H NMR** (600 MHz, $CDCl_3$) δ 10.02 (s, 1H), 8.79 – 8.76 (m, 2H), 8.15 (dd, J = 8.4, 1.8 Hz, 1H), 7.54 – 7.50 (m, 2H), 7.46 – 7.43 (m, 3H), 7.42 (d, J = 7.2 Hz, 2H), 7.31 – 7.29 (m, 4H), 7.29 – 7.27 (m, 1H), 7.25 – 7.22 (m, 1H), 5.38 – 5.26 (m, 1H), 3.86 (d, J = 13.2 Hz, 1H), 3.80 (d, J = 13.2 Hz, 1H), 3.68 – 3.60 (m, 2H), 3.09 – 3.00 (m, 3H), 2.98 – 2.95 (m, 1H). **^{13}C NMR** (150 MHz, $CDCl_3$) δ 168.1 (d, J = 5.4 Hz), 148.4, 138.50, 138.49, 136.4, 134.5, 131.9, 129.4, 128.5, 128.4, 128.2, 128.1, 127.5, 127.4, 123.3, 121.9, 121.8, 116.8, 90.3 (d, J = 170.1 Hz), 85.9, 84.3, 59.0, 56.4 (d, J = 21.9 Hz), 43.8, 42.3 (d, J = 22.5 Hz). **^{19}F NMR** (565 MHz, $CDCl_3$) δ -181.28 – -181.56 (m, 1F). **IR** ν_{max} (film): 2954, 2923, 2851, 2360, 2341, 1652, 1634, 1525, 1457, 1376, 1259, 750, 669, 649, 418 cm^{-1}. **HRMS** (ESI) m/z calcd for $C_{29}H_{27}FN_3O$ $[M+H]^+$: 452.2133; found: 452.2109.

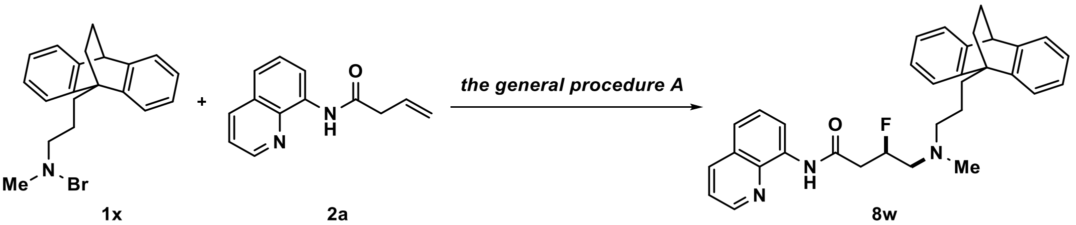

1x **2a** **8w**

Product **8w** was prepared following the general procedure **A**, except that **1x** pre-prepared in DCM (0.20 M, 2.0 mL) was added into the reaction mixture. Purification using column chromatography (PE/EA = 1:1) afforded **8w** as yellow oil (54.8 mg, 0.11 mmol, 54%). **^{1}H NMR** (600 MHz, $CDCl_3$) δ 10.03 (s, 1H), 8.77 (dd, J = 7.2, 1.8 Hz, 1H), 8.73 (dd, J = 4.2, 1.8 Hz, 1H), 8.11 (dd, J = 8.4, 1.8 Hz, 1H), 7.52 – 7.47 (m, 2H), 7.39 (dd, J = 8.4, 4.2 Hz, 1H), 7.25 – 7.24 (m, 2H), 7.23 – 7.21 (m, 2H), 7.10 – 7.07 (m, 2H), 7.06 – 7.02 (m, 2H), 5.44 – 5.22 (m, 1H), 4.24 (t, J = 3.0 Hz, 1H), 3.04 – 2.99 (m, 1H), 2.98 (d, J = 6.0 Hz, 1H), 2.91 (ddd, J = 20.4, 13.8, 6.6 Hz, 1H), 2.83 – 2.79 (m, 1H), 2.78 – 2.71 (m, 2H), 2.47 – 2.45 (m, 1H), 2.44 (s, 3H), 2.44 – 2.42 (m, 1H), 1.99 – 1.90 (m, 2H), 1.79 – 1.77 (m, 2H), 1.54 – 1.50 (m, 2H). **^{13}C NMR** (150 MHz, $CDCl_3$) δ 168.1 (d, J = 5.7 Hz), 148.3, 145.57, 145.55, 145.10, 145.08, 138.4, 136.4, 134.5, 128.0, 127.4, 125.4, 125.28, 125.26, 123.40, 123.38, 121.9, 121.7, 121.40, 121.39, 116.8, 89.9 (d, J = 170.1 Hz), 60.9 (d, J = 21.3 Hz), 59.6, 44.9, 44.7, 43.5, 42.3 (d, J = 22.5 Hz), 29.8, 28.9, 27.8, 22.9. **^{19}F NMR (**475 MHz, $CDCl_3$) δ -180.70 – -181.06 (m, 1F). **IR** ν_{max} (film): 2950, 2866, 1736, 1687, 1527, 1486, 1456, 1424, 1385, 1326, 1240, 1046, 826, 792, 757, 561 cm^{-1}. **HRMS** (ESI) m/z calcd for $C_{33}H_{35}FN_3O$ $[M+H]^+$:

508.2759 ; found: 508.2764.

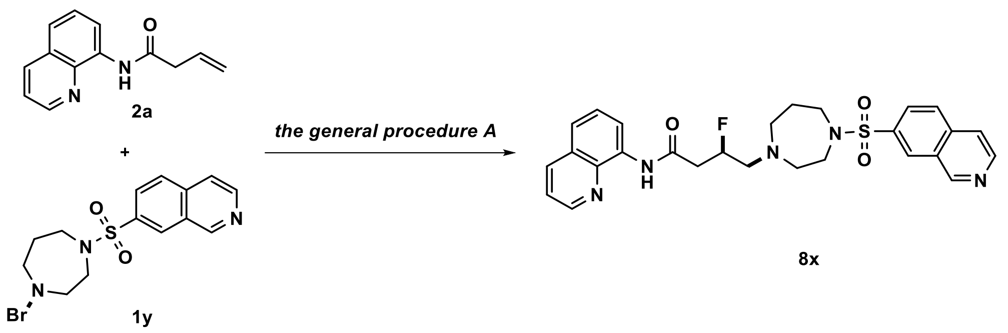

Product **8x** was prepared following the general procedure **A**, except that **1y** pre-prepared in DCM (0.20 M, 2.0 mL) was added into the reaction mixture. Purification using column chromatography (PE/EA = 1:1) afforded **8x** as yellow oil (54.2 mg, 0.10 mmol, 52%). **^{1}H NMR** (600 MHz, $CDCl_3$) δ 9.93 (s, 1H), 9.33 (s, 1H), 8.77 (dd, J = 4.2, 1.8 Hz, 1H), 8.73 (dd, J = 5.4, 3.0 Hz, 1H), 8.67 (d, J = 6.0 Hz, 1H), 8.44 (d, J = 6.0 Hz, 1H), 8.32 (dd, J = 7.2, 1.2 Hz, 1H), 8.17 – 8.13 (m, 2H), 7.68 – 7.64 (m, 1H), 7.53 – 7.50 (m, 2H), 7.44 (dd, J = 8.4, 4.2 Hz, 1H), 5.23 – 5.10 (m, 1H), 3.50 – 3.45 (m, 4H), 2.93 (dd, J = 7.2, 4.2 Hz, 1H), 2.93 – 2.87 (m, 3H), 2.87 – 2.81 (m, 4H), 1.89 – 1.81 (m, 2H). **^{13}C NMR** (150 MHz, $CDCl_3$) δ 167.8 (d, J = 6.9 Hz), 153.3, 148.3, 145.1, 138.4, 136.5, 134.7, 134.3, 133.4, 133.0, 131.7, 129.3, 128.0, 127.4, 126.0, 121.9, 121.8, 117.7, 116.7, 90.1 (d, J = 170.6 Hz), 60.3 (d, J = 21.3 Hz), 57.1, 55.3, 48.5, 46.9, 41.9 (d, J = 22.7 Hz), 28.5. **^{19}F NMR (**475 MHz, $CDCl_3$) δ -181.53 – -181.84 (m, 1F). **IR** ν_{max} (film): 2926, 2851, 2360, 2341, 1687, 1614, 1528, 1486, 1455, 1425, 1386, 1327, 1251, 1161, 1111, 1067, 1009, 826, 791, 757, 701 cm^{-1}. **HRMS** (ESI) m/z calcd for $C_{27}H_{28}FN_5NaO_3S$ [M+Na]$^+$: 544.1789 ; found: 544.1799.

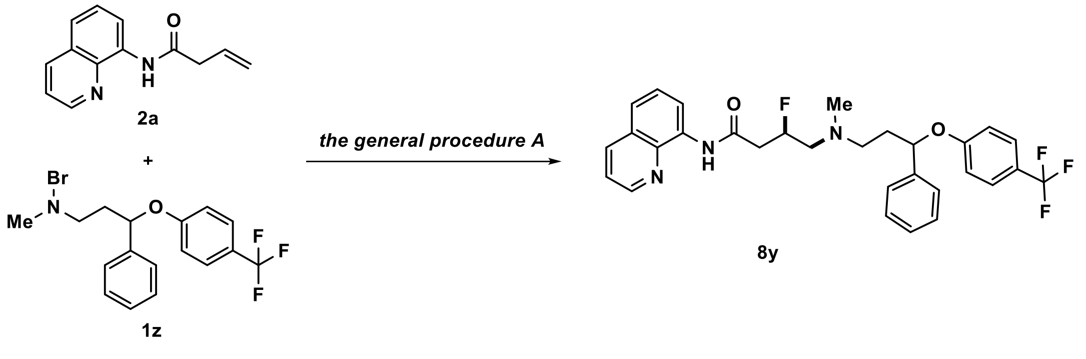

Product **8y** was prepared following the general procedure **A**, except that **1z** pre-prepared in DCM (0.20 M, 2.0 mL) was added into the reaction mixture. Purification using column chromatography (PE/EA = 1:1) afforded **8y** as yellow oil (59.3 mg, 0.11 mmol, 55%, *dr* = 1:1). **^{1}H NMR** (600 MHz, $CDCl_3$) δ 9.96 (s, 1H), 8.80 – 8.77 (m, 1H), 8.77 – 8.74 (m, 1H), 8.16 (dd, J = 3.0, 1.8 Hz, 0.5H), 8.15 (dd, J = 3.0, 1.8 Hz, 0.5H), 7.54 – 7.50 (m, 2H), 7.45 (dd, J = 4.2, 3.0 Hz, 0.5H),

7.43 (dd, J = 4.2, 3.0 Hz, 0.5H), 7.38 – 7.35 (m, 2H), 7.33 – 7.28 (m, 4H), 7.25 – 7.21 (m, 1H), 6.88 (d, J = 7.8 Hz, 2H), 5.34 – 5.30 (m, 1H), 5.28 – 5.17 (m, 1H), 2.92 – 2.78 (m, 3H), 2.73 – 2.55 (m, 3H), 2.35 (s, 1.5H), 2.34 (s, 1.5H), 2.21 – 2.14 (m, 1H), 2.03 – 1.96 (m, 1H). **^{13}C NMR** (150 MHz, $CDCl_3$) δ 168.0 (dd, J = 6.5, 6.5 Hz), 160.8, 160.7, 148.4, 141.3, 141.2, 138.5, 136.5, 134.4, 128.9, 128.1, 127.94, 127.88, 127.5, 126.88, 126.85, 126.83, 126.80, 126.05, 125.97, 125.4, 123.6, 122.9, 122.7, 121.9, 121.79, 121.78, 116.8, 115.9, 89.7 (d, J = 170.1 Hz), 78.4, 78.2, 60.9 (d, J = 4.1 Hz), 60.7 (d, J = 4.5 Hz), 54.49, 54.47, 43.33, 43.26, 42.2 (d, J = 14.1 Hz), 42.0 (d, J = 14.4 Hz), 36.7, 36.6. **^{19}F NMR (**475 MHz, $CDCl_3$) δ -61.43 – -61.49 (m, 3F), -180.55 – -181.20 (m, 1F). **IR** ν_{max} (film): 2928, 1769, 1758, 1686, 1615, 1528, 1486, 1462, 1425, 1373, 1325, 1245, 1157, 1135, 1102, 1049, 913, 827, 793, 742, 679, 591 cm^{-1}. **HRMS** (ESI) m/z calcd for $C_{30}H_{30}F_4N_3O_2$ $[M+H]^+$: 540.2269 ; found: 540.2272.

Part 5: Procedure and characteristic data for compounds 9-12

Procedure for continuous reaction

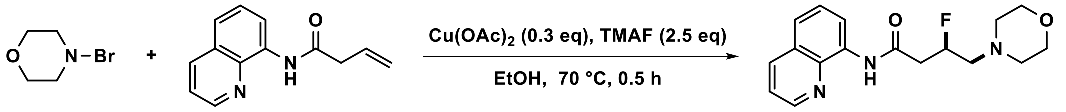

1a 2a 3a

A round-bottomed flask equiped with a nitrogen balloon. A solution of **2a** (424 mg, 2.0 mmol, 1.0 equiv) in EtOH (20 mL) containing $Cu(OAc)_2$ (109 mg, 0.60 mmol, 0.3 equiv) and tetramethylammonium fluoride (TMAF, 466 mg, 5.0 mmol, 2.5 equiv) was prepared and fed into the reactor by pump 1. A second solution of *N*-bromodialkylamine **1a** (20 mL, 4.0 mmol, 0.20 M in EtOH, 2.0 equiv) was fed by pump 2. Pump 3 was used to pump the reaction solution out of the reactor. The flow rate of pump 3 (outflow from reactor) should equal the total flow rate of pumps 1 and 2 (reactor input). The reactor was heated to 70 °C and stirred for half an hour under nitrogen. The reaction solution was collected in reactor volume (residence time) fractions. The reaction

mixture was filtered by Celite, and the filtrate was concentrated *in vacuo*. Further purification by a flash column chromatography using eluents (PE/EA = 1:1) afforded the desired product **3a** as yellow oil (355 mg, 1.12 mmol, 56%).

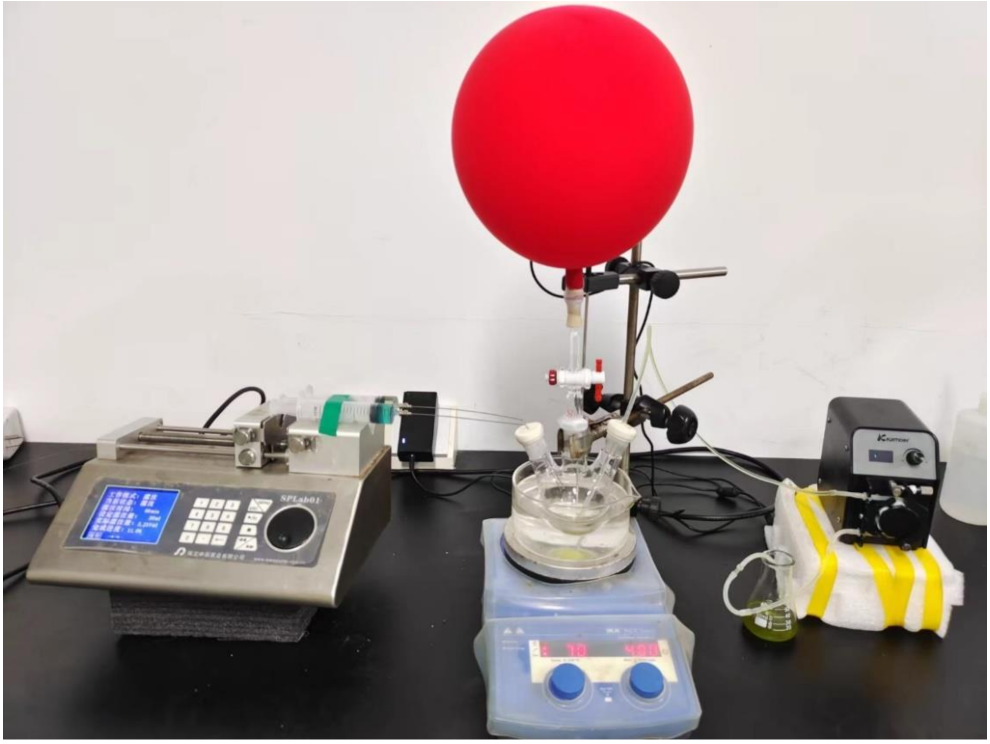

Figure S7. The continuous reaction.

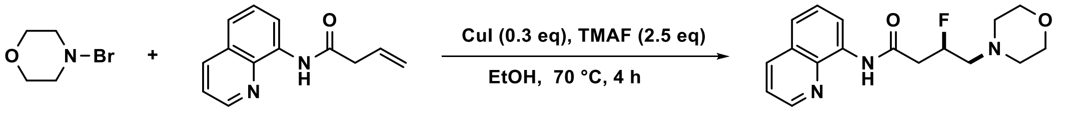

1a **2a** **3a**

To a dry Schlenk flask were added CuI (286 mg, 1.5 mmol, 0.3 equiv), **2a** (1.06 g, 5.0 mmol, 1.0 equiv), tetramethylammonium fluoride (TMAF, 1.16 g, 13 mmol, 2.5 equiv) and anhydrous EtOH (75 mL). The mixture was degassed three times with argon. Then pre-prepared *N*-bromodialkylamine **1a** (50 mL, 10 mmol, 0.20 M in EtOH, 2.0 equiv) was added by syringe pump dropwise into the reaction mixture for 4 hrs at 70 °C (oil bath). This process should avoid exposure to light. When the addition was completed, the mixture was cooled to ambient temperature. The reaction mixture was filtered by Celite, and the filtrate was concentrated *in vacuo*. Further purification by a flash column chromatography using eluents (PE/EA = 1:1) afforded the desired product **3a** as yellow oil (1.14 g, 3.6 mmol, 72%).

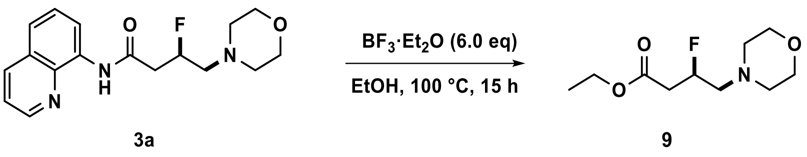

To a dry Schlenk flask were added **3a** (63.4 mg, 0.20 mmol, 1.0 equiv), anhydrous EtOH (2.0 mL) and $BF_3 \cdot Et_2O$ (0.32 mL, 1.2 mmol, 6.0 equiv, 48% in Et_2O) at ambient temperature. The mixture was degassed three times with argon and refluxed at 100 °C (oil bath) for 15 hrs. Once completion, the reaction was cooled to ambient temperature, diluted with DCM (10 mL) and quenched by Et_3N (0.28 mL, 2.0 mmol, 10 equiv). Rotary evaporation of the organic solvent and further purification using column chromatography (PE/EA = 5:1) afforded **9** as colourless oil (32.4 mg, 0.15 mmol, 74% yield). **^{1}H NMR** (600 MHz, $CDCl_3$) δ 5.21 – 5.04 (m, 1H), 4.18 (q, J = 7.2 Hz, 2H), 3.74 (t, J = 4.8 Hz, 4H), 2.78 – 2.73 (m, 2H), 2.72 – 2.65 (m, 2H), 2.63 – 2.57 (m, 4H), 1.28 (t, J = 7.2 Hz, 3H). **^{13}C NMR** (150 MHz, $CDCl_3$) δ 170.0 (d, J = 7.1 Hz), 88.4 (d, J = 170.6 Hz), 66.8, 61.8 (d, J = 21.3 Hz), 61.0, 54.2, 38.7 (d, J = 23.7 Hz), 14.3. **^{19}F NMR (**475 MHz, $CDCl_3$) δ -180.95 – -181.32 (m, 1F). **IR** ν_{max} (film): 2955 , 2922, 2851, 2359, 2341, 1737, 1462, 1377, 1263, 1189, 1146, 1119, 1034, 868, 738 cm^{-1}. **HRMS** (ESI) m/z calcd for $C_{10}H_{19}FNO_3$ $[M+H]^+$: 220.1343; found: 220.1353.

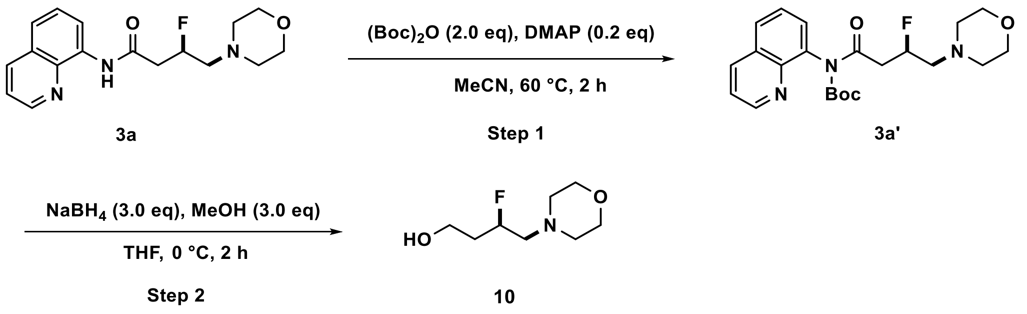

Step 1: To a dry round-bottom flask were added **3a** (63.4 mg, 0.20 mmol, 1.0 equiv), 4-dimethylaminopyridine (4.9 mg, 0.04 mmol, 0.20 equiv) and $(Boc)_2O$ (92 μL, 0.40 mmol, 2.0 equiv). The reaction flask was evacuated and backfilled with N_2, followed by the addition of anhydrous MeCN (2.0 mL). The reaction mixture was heated at 60 °C (oil bath) for 2 hrs. After cooling to room temperature, the mixture was concentrated *in vacuo* and the residue was directly purified by column chromatography (PE/EA = 2:1) to afford the Boc-protected amide **3a'** as light yellow oil (70.9 mg, 0.17 mmol, 85% yield). **^{1}H NMR** (600 MHz, $CDCl_3$) δ 8.88 (dd, J = 4.2, 1.8 Hz, 1H), 8.17 (dd, J = 8.4, 1.8 Hz, 1H), 7.83 (dd, J = 8.4, 1.8 Hz, 1H), 7.58 – 7.52 (m, 2H), 7.42 (dd, J = 8.4, 4.2 Hz, 1H), 5.39 – 5.22 (m, 1H), 3.74 – 3.68 (m, 4H), 3.65 – 3.58 (m, 1H), 3.58 – 3.50 (m, 1H), 2.76 (ddd, J = 21.0, 13.8, 6.6 Hz, 1H), 2.67 (ddd, J = 17.4, 13.8, 3.6 Hz, 1H), 2.62 – 2.56 (m, 2H), 2.56 – 2.50 (m, 2H), 1.23 (s, 9H). **^{13}C NMR** (150 MHz, $CDCl_3$) δ 172.9 (d, J = 8.3

Hz), 152.9, 150.5, 144.2, 136.6, 136.2, 129.2, 129.0, 128.4, 126.2, 121.7, 88.8 (d, J = 168.6 Hz), 83.1, 67.1, 62.3 (d, J = 20.6 Hz), 54.3, 41.8 (d, J = 24.5 Hz), 27.8. **IR** ν_{max} (film): 2925, 2854, 2360, 2341, 1769, 1742, 1694, 1527, 1486, 1457, 1374, 1244, 1169, 1119, 1047, 1006, 827, 735 cm^{-1}. **^{19}F NMR** (565 MHz, $CDCl_3$) δ -181.10 – -181.40 (m, 1F). **HRMS** (ESI) m/z calcd for $C_{22}H_{28}FN_3NaO_4$ [M+Na]$^+$: 440.1956; found: 440.1955.

Step 2: To a solution of the Boc-protected amide **3a'** (70.9 mg, 0.17 mmol, 1.0 equiv) in THF (2.0 mL) was added MeOH (20.6 μL, 0.51 mmol, 3.0 equiv) and $NaBH_4$ (19.3 mg, 0.51 mmol, 3.0 equiv) at 0 °C, and the reaction mixture was stirred at this temperature for 2 hrs. After completed, the solvent volume was doubled with Et_2O (2.0 mL) and the solution was cooled to 0 °C. Saturated aqueous NH_4Cl (1.0 mL) was added dropwise followed by addition of water (1.0 mL).The resulting mixture was extracted with Et_2O, and the organic layer was washed with brine, dried over anhydrous $MgSO_4$, and evaporated. The residue was purified by column chromatography using eluents (PE/EA = 1:1) to afford the desired product **10** as yellow oil (21.1 mg, 0.12 mmol, 70%). **^{1}H NMR** (600 MHz, $CDCl_3$) δ 4.90 – 4.78 (m, 1H), 3.80 – 3.76 (m, 1H), 3.75 – 3.72 (m, 4H), 3.71 – 3.67 (m, 1H), 2.71 – 2.61 (m, 2H), 2.60 – 2.54 (m, 4H), 2.04 – 1.92 (m, 2H). **^{13}C NMR** (150 MHz, $CDCl_3$) δ 90.6 (d, J = 167.7 Hz), 66.7, 62.4 (d, J = 23.1 Hz), 58.0 (d, J = 7.7 Hz), 54.4, 37.3 (d, J = 20.3 Hz). **^{19}F NMR (**475 MHz, $CDCl_3$) δ -180.76 – -181.05 (m, 1F). **IR** ν_{max} (film): 2955, 2923, 2853, 2362, 2342, 1655, 1526, 1457, 1376, 1297, 1117, 1067, 1009, 868, 795 cm^{-1}. **HRMS** (ESI) m/z calcd for $C_8H_{17}FNO_2$ [M+H]$^+$: 178.1238; found: 178.1230.

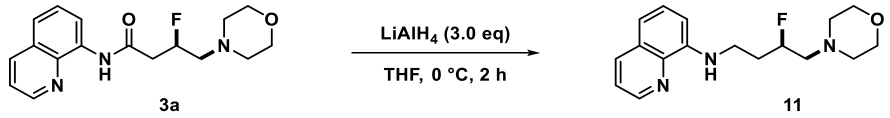

To a solution of $LiAlH_4$ (22.8 mg, 0.60 mmol, 3.0 equiv) in anhydrous THF (1.0 mL) at 0 °C was added dropwise a solution of compound **3a** (63.4 mg, 0.20 mmol, 1.0 equiv) in THF (1.0 mL). Then the mixture was stirred for 2 hrs at the same temperature. Once completion, an aqueous solution of NaOH (10 mL, 10% in water) was added dropwise into the reaction mixture. The mixture was extracted with Et_2O (15 mL × 3). The combined organic phase was dried over $MgSO_4$ and concentrated *in vacuo*. And the residue was purified by column chromatography using eluents (PE/EA = 1:1) to afford the desired product **11** as yellow oil (39.4 mg, 0.13 mmol, 65%). **^{1}H NMR** (600 MHz, $CDCl_3$) δ 8.70 (dd, J = 4.2, 1.8 Hz, 1H), 8.06 (dd, J = 8.4, 1.8 Hz, 1H), 7.40 – 7.36 (m, 2H), 7.05 (d, J = 7.8 Hz, 1H), 6.70 (d, J = 7.8 Hz, 1H), 6.26 (br, 1H), 4.99 – 4.87 (m, 1H), 3.72 (t, J = 4.8 Hz, 4H), 3.54 – 3.49 (m, 2H), 2.69 (ddd, J = 20.4, 13.8, 6.6 Hz, 1H), 2.59 – 2.52 (m, 5H), 2.15 – 2.06 (m, 2H). **^{13}C NMR** (150 MHz, $CDCl_3$) δ 147.0, 144.7, 138.4, 136.2, 128.8, 127.9, 121.6, 114.1, 104.7, 90.9 (d, J = 168.3 Hz), 67.1, 62.7 (d, J = 21.2 Hz), 54.4, 39.5 (d, J = 5.1 Hz), 33.2 (d,

J = 20.7 Hz). **^{19}F NMR (**475 MHz, $CDCl_3$) δ -181.73 – -182.06 (m, 1F). **IR** ν_{max} (film): 2955, 2852, 2357, 2340, 1655, 1527, 1458, 1377, 1324, 1113, 862, 792 cm^{-1}. **HRMS** (ESI) m/z calcd for $C_{17}H_{23}FN_3O$ [M+H]$^+$: 304.1820; found: 304.1823.

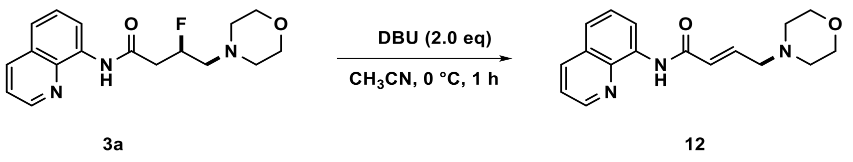

To a solution of the compound **3a** (63.4 mg, 0.20 mmol, 1.0 equiv) in CH_3CN (2.0 mL) was added 1,8-diazabicyclo[5.4.0]undec-7-ene (DBU, 60 µL, 0.40 mmol, 2.0 equiv) at 0 °C, and the reaction mixture was stirred at this temperature for 1 hr. After completed, the solvent was removed under reduced pressure and the residue was purified by column chromatography using eluents (PE/EA = 1:2) to afford the desired product **12** as yellow oil (50.5 mg, 0.17 mmol, 85%). Compound **12** is a known compound.[2]

Part 6: Control experiments and radical clock experiments

Detection of copper intermediates by ESI-MS

The Cu(I) amino intermediate **M4**, Cu(II) fluoride **M6a** and amino Cu(III) fluoride **M8a** could be detected by ESI-MS analysis when the aminofluorination reaction was preformed under optimized conditions after 10 min. These results provide a direct evidence for our proposed reaction mechanism.

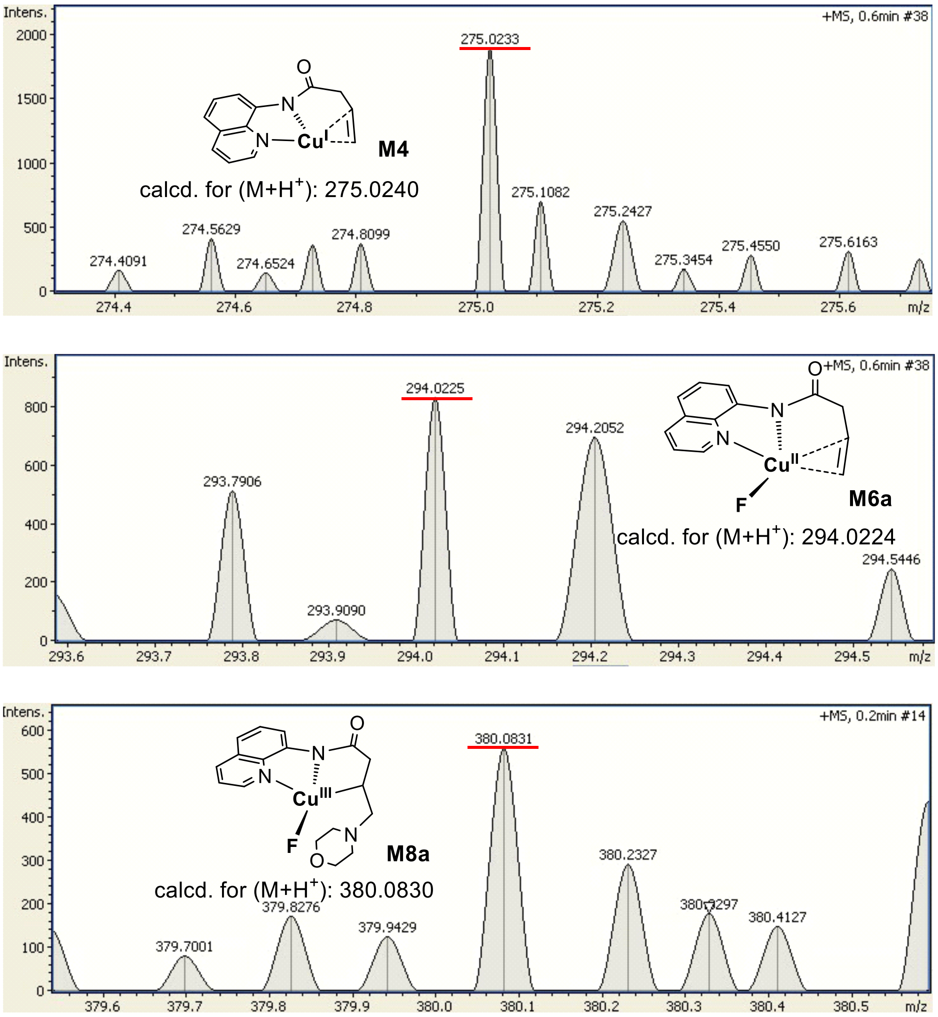

Figure S8. Detection of copper intermediates by ESI-MS.

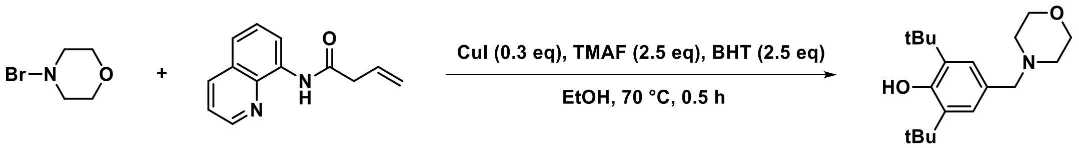

1a 2a 13

To a dry Schlenk flask were added CuI (11.4 mg, 0.06 mmol, 0.3 equiv), **2a** (42.4 mg, 0.20 mmol, 1.0 equiv), tetramethylammonium fluoride (TMAF, 46.6 mg, 0.50 mmol, 2.5 equiv), butylated hydroxytoluene (BHT, 110 mg, 0.50 mmol, 2.5 equiv) and anhydrous EtOH (3.0 mL). The mixture was degassed three times with argon. Then pre-prepared *N*-bromodialkylamine **1a** (2.0 mL, 0.40 mmol, 0.20 M in EtOH, 2.0 equiv) was added by syringe pump dropwise into the reaction mixture for half an hour at 70 °C (oil bath). This process should avoid exposure to light. When the addition was completed, the mixture was cooled to ambient temperature. The reaction mixture was filtered by Celite, and the filtrate was concentrated *in vacuo*. Purification using column

chromatography (PE/EA = 6:1) afforded compound **13** as yellow oil (58.6 mg, 0.19 mmol, 48% related to **1a**). Compound **13** is a known compound.[12]

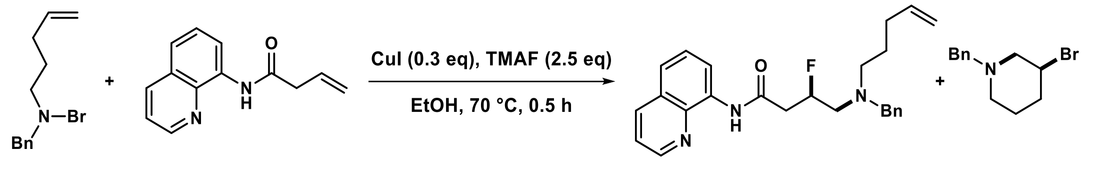

To a dry Schlenk flask were added CuI (11.4 mg, 0.06 mmol, 0.3 equiv), **2a** (42.4 mg, 0.20 mmol, 1.0 equiv), tetramethylammonium fluoride (TMAF, 46.6 mg, 0.50 mmol, 2.5 equiv) and anhydrous EtOH (3.0 mL). The mixture was degassed three times with argon. Then pre-prepared *N*-bromodialkylamine **14** (2.0 mL, 0.40 mmol, 0.20 M in EtOH, 2.0 equiv) was added by syringe pump dropwise into the reaction mixture for half an hour at 70 °C (oil bath). This process should avoid exposure to light. When the addition was completed, the mixture was cooled to ambient temperature. The reaction mixture was filtered by Celite, and the filtrate was concentrated *in vacuo*. Purification using column chromatography (PE/EA = 5:1) afforded **15** as yellow oil (41.3 mg, 0.10 mmol, 51%) and known compound **16**[13] as yellow oil (8.1 mg, 0.03 mmol, 8% related to **14**). Compound **15** : **^{1}H NMR** (600 MHz, $CDCl_3$) δ 9.94 (s, 1H), 8.81 (dd, J = 4.2, 1.8 Hz, 1H), 8.76 (dd, J = 7.2, 1.8 Hz, 1H), 8.16 (dd, J = 8.4, 1.8 Hz, 1H), 7.56 – 7.50 (m, 2H), 7.45 (dd, J = 8.4, 4.2 Hz, 1H), 7.34 (d, J = 6.6 Hz, 2H), 7.28 (t, J = 7.2 Hz, 2H), 7.21 (t, J = 7.2 Hz, 1H), 5.76 (ddt, J = 16.8, 10.2, 6.6 Hz, 1H), 5.29 – 5.18 (m, 1H), 5.00 – 4.93 (m, 1H), 4.93 – 4.88 (m, 1H), 3.71 – 3.66 (m, 2H), 2.92 – 2.87 (m, 1H), 2.86 – 2.82 (m, 2H), 2.82 – 2.73 (m, 1H), 2.59 – 2.53 (m, 2H), 2.07 – 2.03 (m, 2H), 1.62 – 1.58 (m, 2H). **^{13}C NMR** (150 MHz, $CDCl_3$) δ 168.3 (d, J = 4.4 Hz), 148.4, 139.5, 138.7, 138.5, 136.5, 134.5, 129.1, 128.4, 128.1, 127.5, 127.1, 121.83, 121.76, 116.8, 114.7, 90.3 (d, J = 169.8 Hz), 59.5, 57.1 (d, J = 21.9 Hz), 54.4, 42.4 (d, J = 22.1 Hz), 31.5, 26.6. **^{19}F NMR (**475 MHz, $CDCl_3$) δ -181.20 – -181.51 (m, 1F). **IR** ν_{max} (film):3350, 2955, 2924, 2853, 2361, 2342, 1685, 1527, 1486, 1458, 1424, 1378, 912, 825, 791, 741, 695 cm^{-1}. **HRMS** (ESI) m/z calcd for $C_{25}H_{29}FN_3O$ $[M+H]^+$: 406.2289; found: 406.2283.

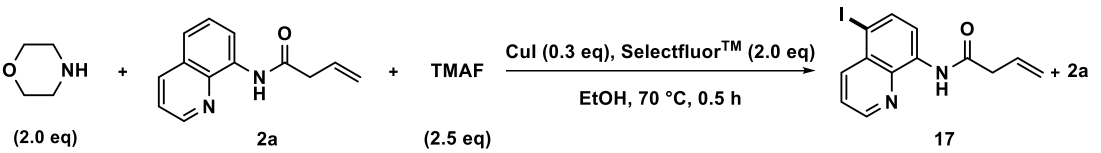

To a solution of CuI (11.4 mg, 0.06 mmol, 0.3 equiv), **2a** (42.4 mg, 0.20 mmol, 1.0 equiv), tetramethylammonium fluoride (TMAF, 46.6 mg, 0.50 mmol, 2.5 equiv) and morpholine (35 µL,

0.40 mmol, 2.0 equiv) in anhydrous EtOH (5.0 mL) was added Selectfluor^{TM} (142 mg, 0.40 mmol, 2.0 equiv). The mixture was degassed three times with argon and then heated at 70 °C (oil bath) for half an hour. This process should avoid exposure to light. After cooled to ambient temperature, the reaction mixture was filtered by Celite, and the filtrate was concentrated *in vacuo*. Purification using column chromatography (PE/EA = 5:1) afforded **17** as yellow oil (8.8 mg, 0.03 mmol, 13%) and recovered the alkene substrate **2a** (26.3 mg, 0.12 mmol, 62%). **^{1}H NMR** (600 MHz, $CDCl_3$) δ 9.97 (s, 1H), 8.77 (dd, J = 4.2, 1.2 Hz, 1H), 8.55 (d, J = 7.8 Hz, 1H), 8.37 (dd, J = 8.4, 1.8 Hz, 1H), 8.07 (d, J = 7.8 Hz, 1H), 7.53 (dd, J = 8.4, 4.2 Hz, 1H), 6.13 (ddt, J = 17.4, 10.2, 7.2 Hz, 1H), 5.42 – 5.39 (m, 1H), 5.39 – 5.36 (m, 1H), 3.35 (d, J = 7.2 Hz, 2H). **^{13}C NMR** (150 MHz, $CDCl_3$) δ 169.4, 149.0, 140.9, 139.2, 138.4, 135.4, 130.9, 129.7, 123.3, 120.4, 118.0, 89.5, 43.3. **HRMS** (ESI) m/z calcd for $C_{13}H_{11}IN_2NaO$ $[M+Na]^+$: 360.9808; found: 360.9812.

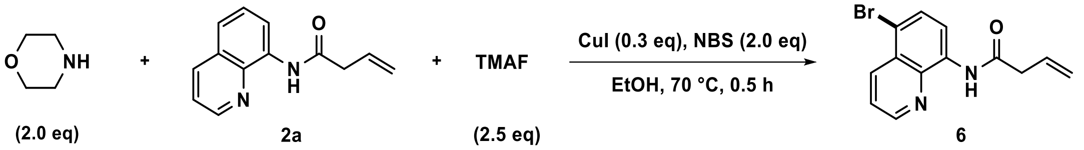

To a dry Schlenk flask were added CuI (11.4 mg, 0.06 mmol, 0.3 equiv), **2a** (42.4 mg, 0.20 mmol, 1.0 equiv), tetramethylammonium fluoride (TMAF, 46.6 mg, 0.50 mmol, 2.5 equiv), morpholine (35 μL, 0.40 mmol, 2.0 equiv) and anhydrous EtOH (3.0 mL). The mixture was degassed three times with argon. Then a solution of *N*-bromosuccinimide (NBS, 71.2 mg, 0.40 mmol, 2.0 equiv) in EtOH (2.0 ml) was added by syringe pump dropwise into the reaction mixture for half an hour at 70 °C (oil bath). This process should avoid exposure to light. When the addition was completed, the mixture was cooled to ambient temperature. The reaction mixture was filtered by Celite, and the filtrate was concentrated *in vacuo*. Purification using column chromatography (PE/EA = 5:1) afforded **6** as yellow oil (34.2 mg, 0.12 mmol, 59%). **^{1}H NMR** (600 MHz, $CDCl_3$) δ 9.93 (s, 1H), 8.81 (dd, J = 4.2, 1.2 Hz, 1H), 8.66 (d, J = 8.4 Hz, 1H), 8.51 (dd, J = 8.4, 1.2 Hz, 1H), 7.78 (d, J = 8.4 Hz, 1H), 7.55 (dd, J = 8.4, 4.2 Hz, 1H), 6.13 (ddt, J = 17.4, 10.2, 7.2 Hz, 1H), 5.43 – 5.39 (m, 1H), 5.39 – 5.36 (m, 1H), 3.35 (d, J = 7.2 Hz, 2H). **^{13}C NMR** (150 MHz, $CDCl_3$) δ 169.4, 148.9, 139.3, 136.1, 134.4, 131.0, 130.9, 127.3, 122.8, 120.4, 117.1, 114.4, 43.3. **HRMS** (ESI) m/z calcd for $C_{13}H_{11}BrN_2NaO$ $[M+Na]^+$: 312.9947; found: 312.9943.

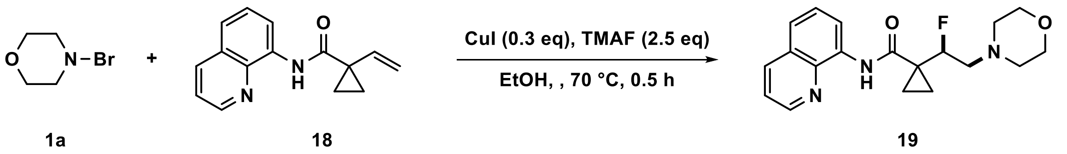

To a dry Schlenk flask were added CuI (11.4 mg, 0.06 mmol, 0.30 equiv), **18** (47.6 mg, 0.20 mmol, 1.0 equiv), tetramethylammonium fluoride (TMAF, 46.6 mg, 0.50 mmol, 2.5 equiv) and anhydrous EtOH (3.0 mL). The mixture was degassed three times with argon. Then pre-prepared *N*-bromodialkylamine **1a** (2.0 mL, 0.40 mmol, 0.20 M in EtOH, 2.0 equiv) was added by syringe pump dropwise into the reaction mixture for half an hour at 70 °C (oil bath). This process should avoid exposure to light. When the addition was completed, the mixture was cooled to ambient temperature. The reaction mixture was filtered by Celite, and the filtrate was concentrated *in vacuo*. Purification using column chromatography (PE/EA = 2:1) afforded **19** as yellow oil (37.1 mg, 0.11 mmol, 54%). **^{1}H NMR** (600 MHz, $CDCl_3$) δ 10.59 (d, J = 7.2 Hz, 1H), 8.84 (dd, J = 4.2, 1.8 Hz, 1H), 8.71 (dd, J = 7.2, 1.8 Hz, 1H), 8.15 (dd, J = 8.4, 1.8 Hz, 1H), 7.55 – 7.49 (m, 2H), 7.45 (dd, J = 8.4, 4.2 Hz, 1H), 4.53 – 4.43 (m, 1H), 3.70 – 3.62 (m, 4H), 3.11 (ddd, J = 18.0, 14.4, 7.8 Hz, 1H), 2.82 (ddd, J = 17.4, 14.4, 3.0 Hz, 1H), 2.64 – 2.59 (m, 2H), 2.58 – 2.54 (m, 2H), 1.65 – 1.59 (m, 1H), 1.32 (ddd, J = 9.6, 6.6, 4.8 Hz, 1H), 1.14 (ddd, J = 9.6, 7.2, 4.8 Hz, 1H), 0.88 – 0.84 (m, 1H). **^{13}C NMR** (150 MHz, $CDCl_3$) δ 169.4, 148.5, 138.8, 136.3, 134.9, 128.1, 127.4, 121.74, 121.70, 116.9, 96.9 (d, J = 173.4 Hz), 67.0, 61.2 (d, J = 22.4 Hz), 54.4, 28.8 (d, J = 23.4 Hz), 14.8, 10.7 (d, J = 6.8 Hz). **^{19}F NMR (**475 MHz, $CDCl_3$) δ -175.86 – -176.12 (m, 1F). **IR** ν_{max} (film): 2923, 2852, 2362, 2342, 1675, 1530, 1487, 1454, 1424, 1387, 1324, 1116, 1009, 951, 864, 826, 791, 757 cm^{-1}. **HRMS** (ESI) m/z calcd for $C_{19}H_{22}FN_3NaO_2$ $[M+Na]^+$: 366.1588; found: 366.1584.

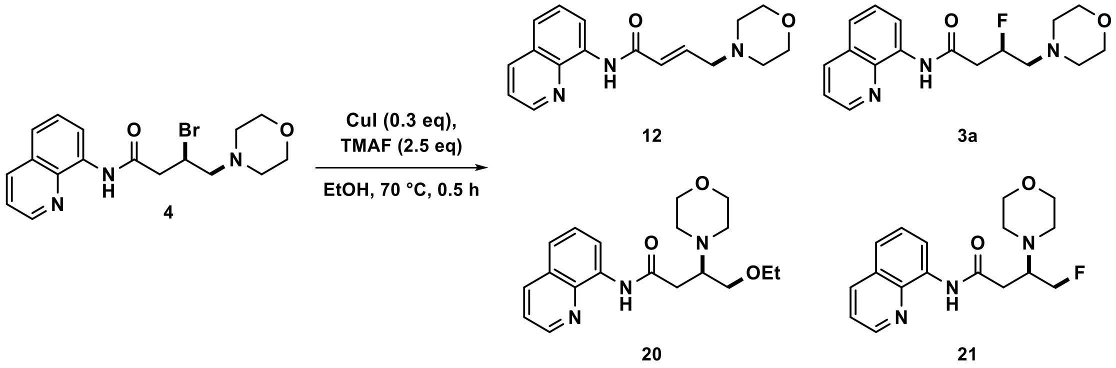

To a dry Schlenk flask were added CuI (11.4 mg, 0.06 mmol, 0.3 equiv), known compound **4**[2] (75.4 mg, 0.20 mmol, 1.0 equiv), tetramethylammonium fluoride (TMAF, 46.6 mg, 0.50 mmol, 2.5 equiv) and anhydrous EtOH (5.0 mL). The mixture was degassed three times with argon and then stirred for half an hour at 70 °C (oil bath). After cooled to ambient temperature, the reaction mixture was filtered by Celite, and the filtrate was concentrated *in vacuo*. Purification using column chromatography (PE/EA = 5:1, 1:1 to 1:2) afforded compounds **20** as yellow oil (8.9 mg,

0.03 mmol, 13%), **21** as yellow oil (8.2 mg, 0.03 mmol, 13%), **3a** as yellow oil (9.5 mg, 0.03 mmol, 15%), **12** as yellow oil (25.0 mg, 0.08 mmol, 42%).

Compound **20** : **^{1}H NMR** (600 MHz, $CDCl_3$) δ 11.28 (s, 1H), 8.85 – 8.82 (m, 2H), 8.16 (dd, *J* = 8.4, 1.8 Hz, 1H), 7.55 – 7.49 (m, 2H), 7.45 (dd, *J* = 8.4, 4.2 Hz, 1H), 4.01 – 3.94 (m, 2H), 3.92 – 3.85 (m, 2H), 3.66 (dd, *J* = 9.6, 5.4 Hz, 1H), 3.53 (dd, *J* = 10.2, 5.4 Hz, 1H), 3.49 (q, *J* = 7.2 Hz, 2H), 3.28 – 3.22 (m, 1H), 3.00 – 2.93 (m, 2H), 2.82 (dd, *J* = 16.2, 10.2 Hz, 1H), 2.77 – 2.71 (m, 2H), 2.61 (dd, *J* = 15.6, 3.6 Hz, 1H), 1.20 (t, *J* = 7.2 Hz, 3H). **^{13}C NMR** (150 MHz, $CDCl_3$) δ 171.1, 148.1, 139.1, 136.4, 135.5, 128.3, 127.6, 121.7, 121.6, 117.7, 69.5, 67.2, 66.8, 61.1, 49.8, 36.7, 15.3. **IR** ν_{max} (film): 2955, 2924, 2853, 1740, 1677, 1526, 1487, 1463, 1424, 1377, 1323, 1116, 1006, 971, 852, 825, 791, 755 cm^{-1}. **HRMS** (ESI) m/z calcd for $C_{19}H_{26}N_3O_3$ $[M+H]^+$: 344.1969; found: 344.1973.

Compound **21** : **^{1}H NMR** (600 MHz, $CDCl_3$) δ 11.10 (s, 1H), 8.84 (dd, *J* = 4.2, 1.8 Hz, 1H), 8.81 (dd, *J* = 7.2, 1.8 Hz, 1H), 8.17 (dd, *J* = 8.4, 1.8 Hz, 1H), 7.56 – 7.51 (m, 2H), 7.47 (dd, *J* = 8.4, 4.2 Hz, 1H), 4.77 – 4.57 (m, 2H), 4.00 – 3.93 (m, 2H), 3.91 – 3.85 (m, 2H), 3.39 – 3.30 (m, 1H), 2.98 – 2.92 (m, 2H), 2.90 (dd, *J* = 15.6, 9.6 Hz, 1H), 2.79 – 2.73 (m, 2H), 2.59 (dd, *J* = 16.2, 4.2 Hz, 1H). **^{13}C NMR** (150 MHz, $CDCl_3$) δ 170.1, 148.2, 139.0, 136.5, 135.2, 128.2, 127.5, 121.9, 121.7, 117.6, 82.7 (d, *J* = 173.0 Hz), 67.2, 61.2 (d, *J* = 18.0 Hz), 50.0, 35.3 (d, *J* = 6.9 Hz). **^{19}F NMR (**475 MHz, $CDCl_3$) δ -224.08 – -224.36 (m, 1F). **IR** ν_{max} (film): 2956, 2925, 2853, 1676, 1526, 1488, 1463, 1424, 1377, 1324, 1116, 825, 792, 739, 704 cm^{-1}. **HRMS** (ESI) m/z calcd $C_{17}H_{21}FN_3O_2$ $[M+H]^+$: 318.1612; found: 318.1614.

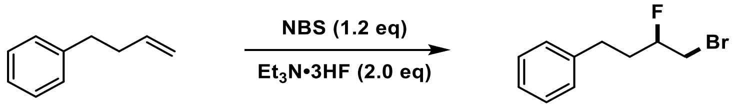

24

To a dry Schlenk flask were added 4-phenyl-1-butene (661 mg, 5.0 mmol, 1.0 equiv), NBS (1.07 g, 6.0 mmol, 1.2 equiv), $Et_3N{\cdot}3HF$ (1.61 g, 10.0 mmol, 2.0 equiv), and anhydrous DCM (2.5 mL) at 0 °C. The mixture was degassed three times with argon, and then stirred for 2 hours. The reaction mixture was filtered by Celite, and the filtrate was concentrated *in vacuo*. Purification using column chromatography (PE/EA = 8:1) afforded compound **24** as yellow oil (58.6 mg, 0.19 mmol, 48%). **^{1}H NMR** (600 MHz, $CDCl_3$) δ 7.34 – 7.24 (m, 2H), 7.23 – 7.17 (m, 3H), 4.70 – 4.55 (m, 1H), 3.46 (dd, *J* = 19.8, 5.4 Hz, 2H), 2.82 (ddd, *J* = 14.4, 9.6, 5.4 Hz, 1H), 2.71 (dt, *J* = 14.4, 8.4 Hz, 1H), 2.15 – 1.92 (m, 2H). **^{13}C NMR** (150 MHz, $CDCl_3$) δ 140.7, 128.7, 128.5, 126.4, 91.7, 90.5, 35.3, 35.1, 33.8, 33.6, 31.0, 31.0. **^{19}F NMR** (565 MHz, $CDCl_3$) δ -166.65 – -185.28 (m). **IR** ν_{max} (film): 2952, 2482, 2342, 1645, 1542, 1487, 1453, 1392, 1345, 1120, 1009, 947, 828, 794, 756 cm^{-1}. **HRMS** (ESI) m/z calcd for $C_{10}H_{13}FBr$ $[M+H]^+$: 231.0179; found: 231.0185.

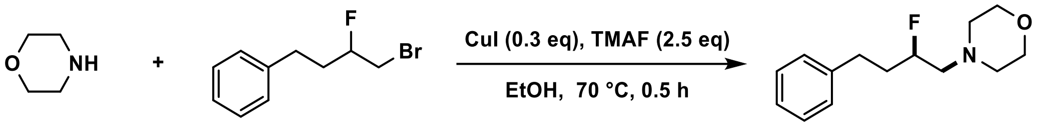

To a dry Schlenk flask were added CuI (11.4 mg, 0.06 mmol, 0.3 equiv), **24** (46.2 mg, 0.20 mmol, 1.0 equiv), tetramethylammonium fluoride (TMAF, 46.6 mg, 0.50 mmol, 2.5 equiv), and anhydrous EtOH (3.0 mL). The mixture was degassed three times with argon. Then morpholine (35 μL, 0.40 mmol, 2.0 equiv) was added into the reaction mixture. The mixture was stirred for 0.5 hours at 70 °C (oil bath). After that, the reaction mixture was filtered by Celite, and the filtrate was concentrated *in vacuo*. Further purification by a flash column chromatography using eluents (PE/EA = 20:1) just recovered the substrate **24** (39.1 mg, 0.17 mmol, 87%).

Part 7: References

[1]. C. L. Tang, R. Zhang, B. Zhu, J. K. Fu, Y. Deng, L. Tian, W. Guan, X. H. Bi. *J. Am. Chem. Soc.* **2018**, *140*, 16929.

[2]. Y. Li, Y. J. Liang, J. C. Dong, Y. Deng, C. Y. Zhao, Z. M. Su, W. Guan, X. H. Bi, Q. Liu, J. K. Fu. *J. Am. Chem. Soc.* **2019**, *141*, 18475.

[3]. D. D. Yang, H. Huang, M.-H. Li, X.-J. Si, H. Zhang, J.-L. Niu, and M.-P. Song. *Org. Lett.* **2020**, *22*, 4333.

[4]. L.-Y. Fu, J. Ying, X. X. Qi, J.-B. Peng, X.-F. Wu. *J. Org. Chem.* **2019**, *84*, 1421.

[5]. T. Y. Taha, S. M. Aboukhatwa, R. C. Knopp, N. Ikegaki, H. Abdelkarim, J. Neerasa, Y. L. Lu, R. Neelarapu, T. W. Hanigan, G. R. J. Thatcher, P. A. Petukhov. *ACS Med. Chem. Lett.* **2017**, *8*, 824.

[6]. S. G. Davies, A. L. A. Figuccia, A. M. Fletcher Paul, M. Roberts, and J. E. Thomson. *J. Org. Chem.* **2016**, *81*, 6481.

[7]. E. Sathiyaraj, S. Thirumaran, *Spectrochim. Acta A: Mol. Biomol. Spectrosc.* **2012**, *97*, 575.

[8]. M. G. Götz, K. E. James, E. Hansell, J. Dvořák, A. Seshaadri, D. Sojka, P. Kopáček, J. H. McKerrow, C. Caffrey, J. C. Powers. *J. Med. Chem.* **2008**, *51*, 2816.

[9]. R. Ebule, S. Mudshinge, M. H. Nantz, M. S. Mashuta, G. B. Hammond, B. Xu, *J. Org. Chem.* **2019**, *84*, 3249.

[10]. P. D. G. Greenwood, E. Grenet, J. Waser, *Chem. Eur. J.* **2019**, *25*, 3010.

[11]. C. Grohmann, H. Wang, F. Glorius, *Org. Lett.* **2012**, *14*, 656.

[12]. S. Mondal, S. Samanta, S. Jana, A. Hajra, *J. Org. Chem*. **2017**, *82*, 4504.

[13]. K. Hayashi, E. Kujime, H. Katayama, S. Sano, M. Shiro, Y. Nagao. *Chem. Pharm. Bull.* **2009**, *57*, 1142.

Part 8: NMR spectra

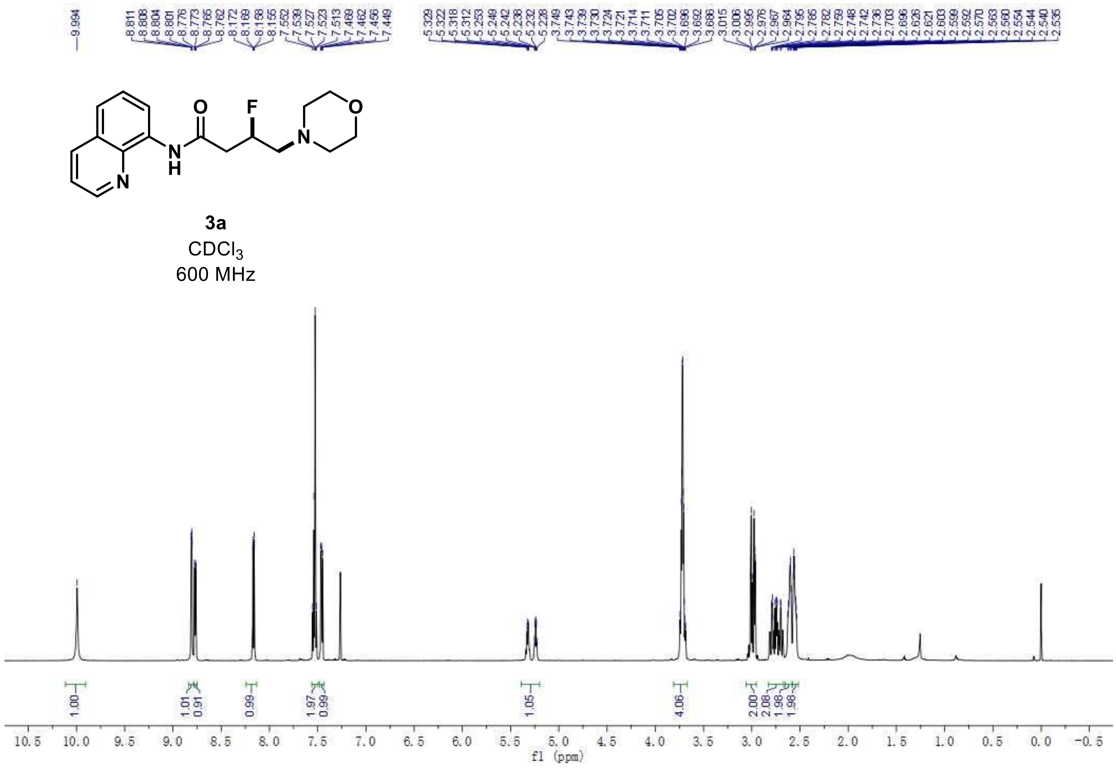

Figure S9. ^{1}H NMR Spectra of **3a**.

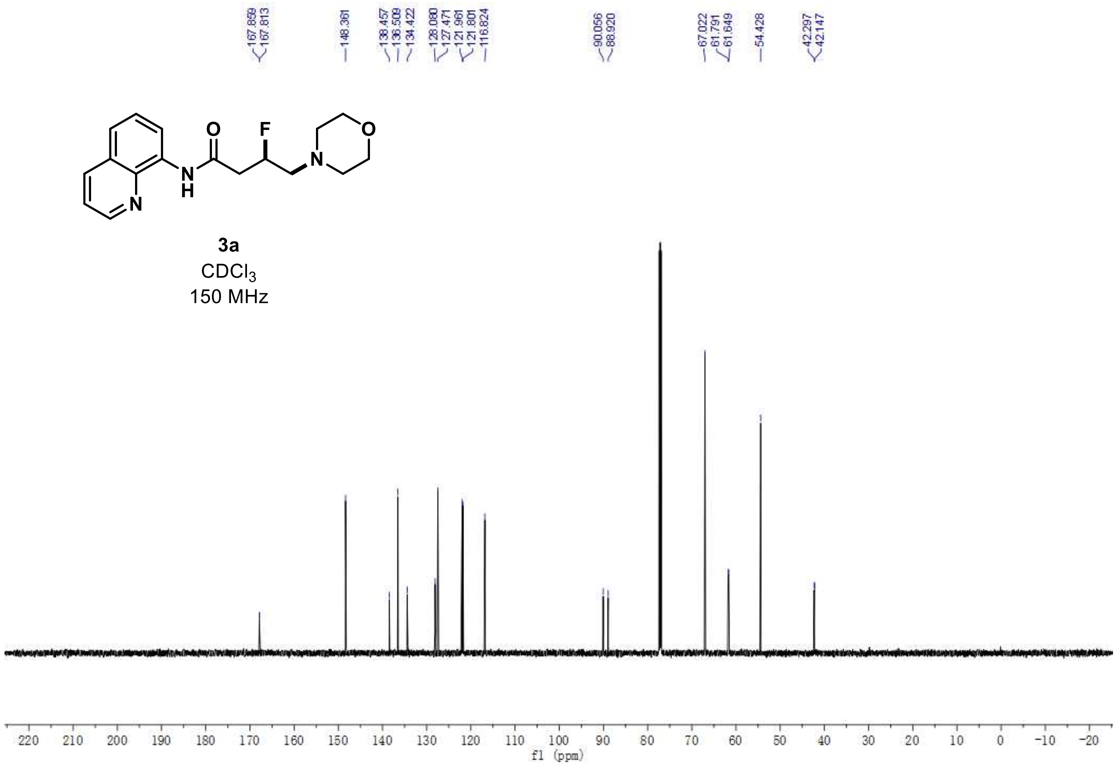

Figure S10. ^{13}C NMR Spectra of **3a**.

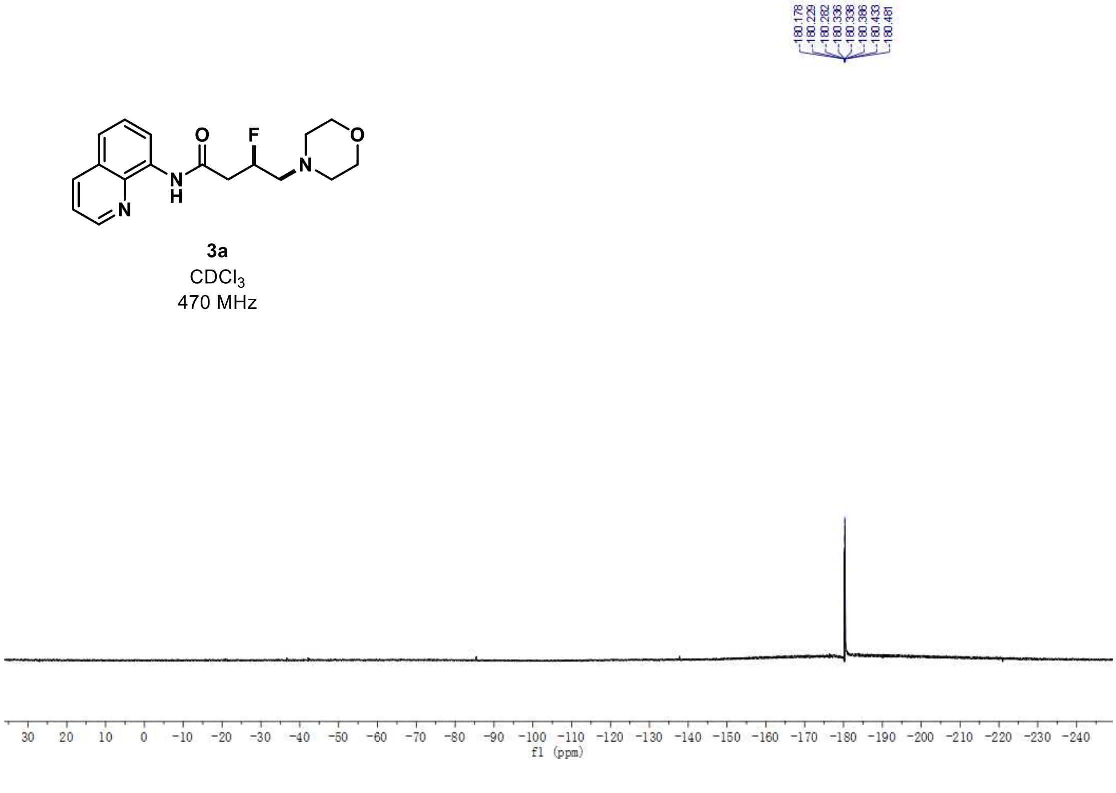

Figure S11. ^{19}F NMR Spectra of **3a**.

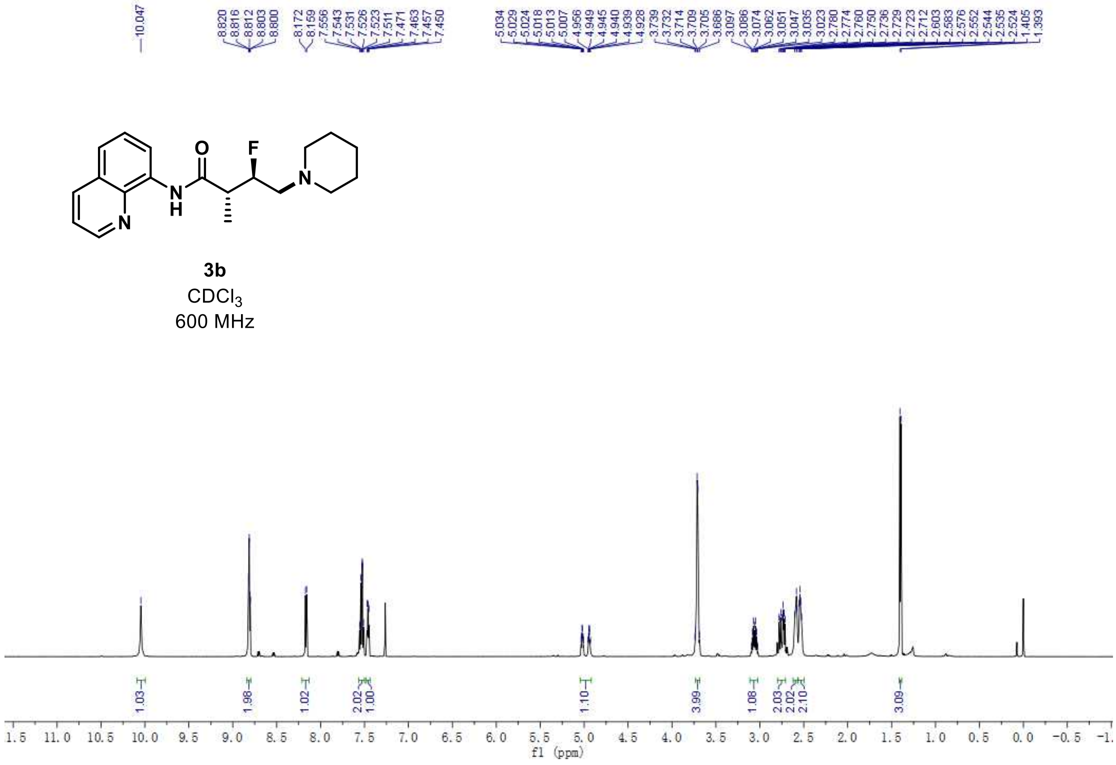

Figure S12. ^{1}H NMR Spectra of **3b**.

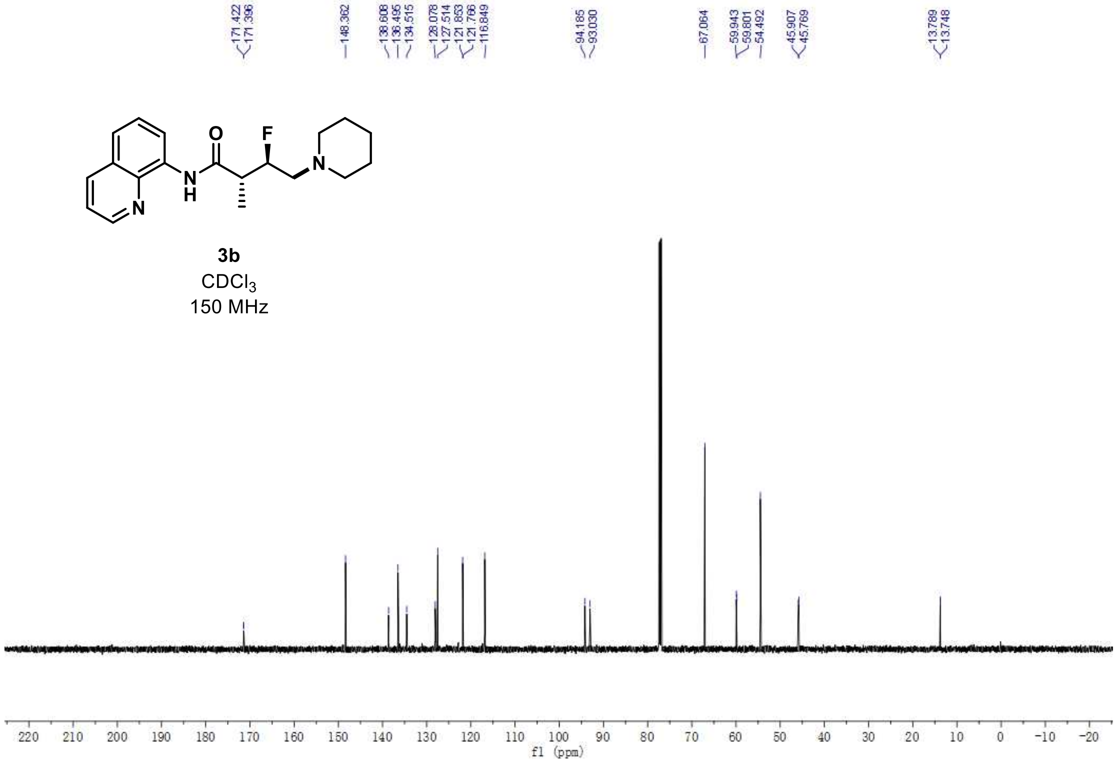

Figure S13. ^{13}C NMR Spectra of **3b**.

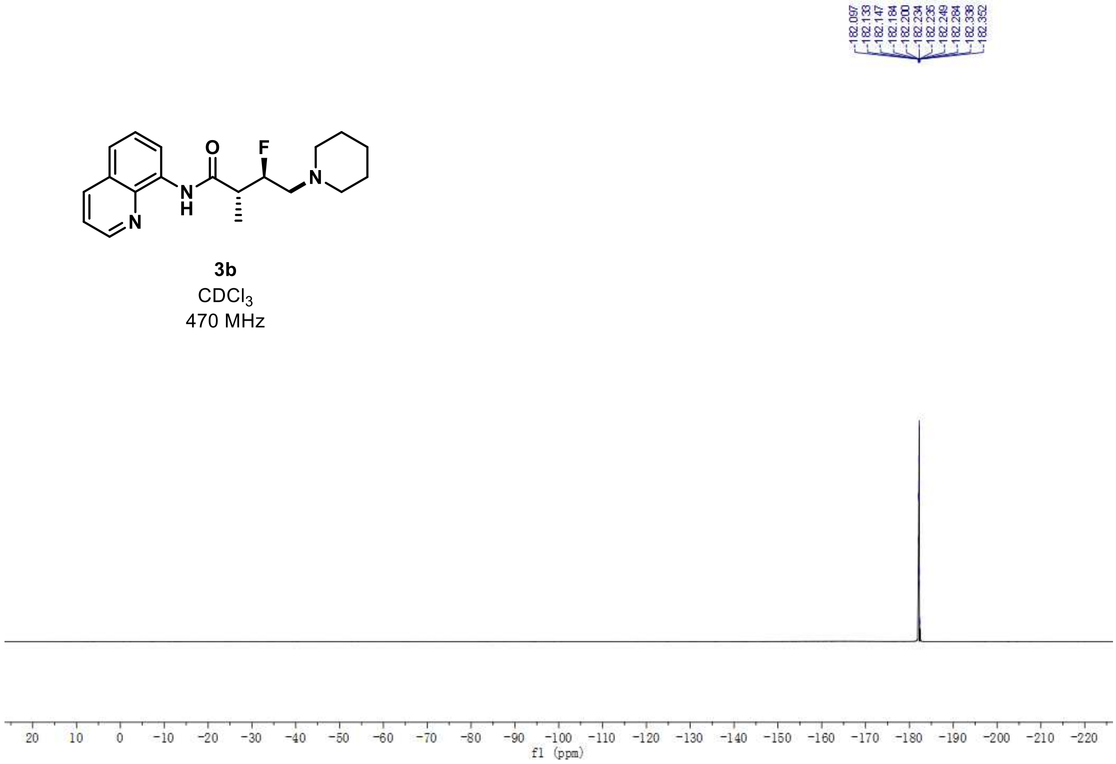

Figure S14. ^{19}F NMR Spectra of **3b**.

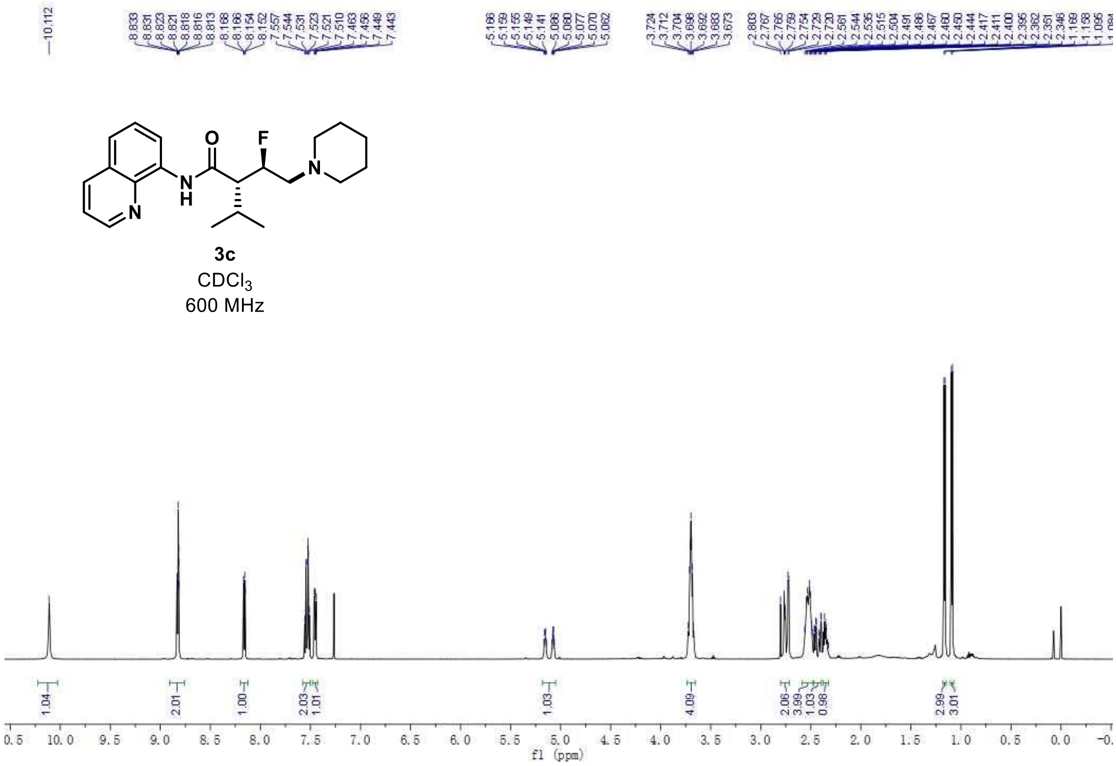

Figure S15. ^{1}H NMR Spectra of **3c**.

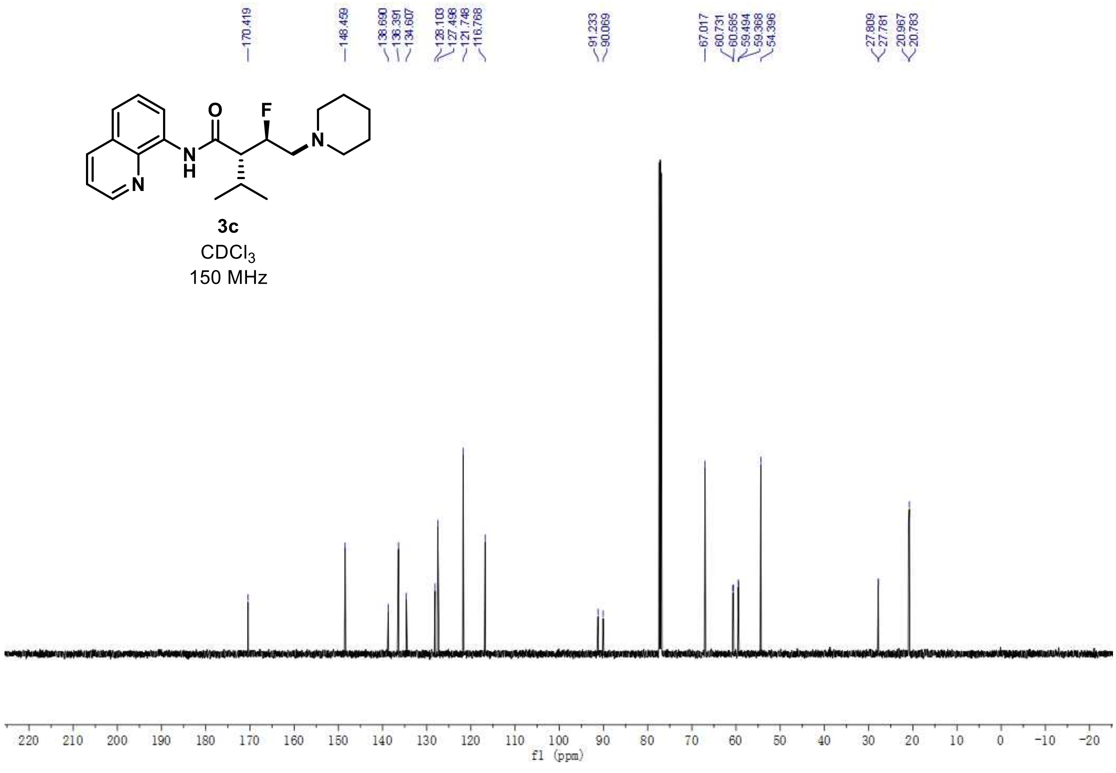

Figure S16. ^{13}C NMR Spectra of **3c**.

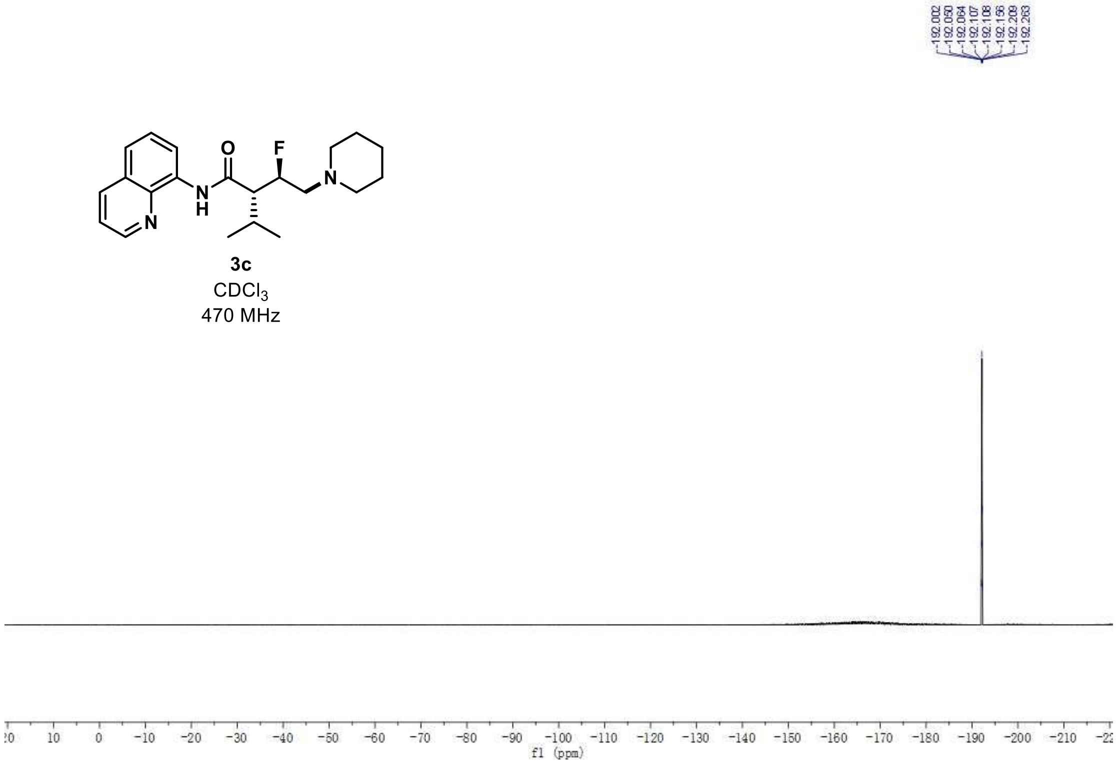

Figure S17. ^{19}F NMR Spectra of **3c**

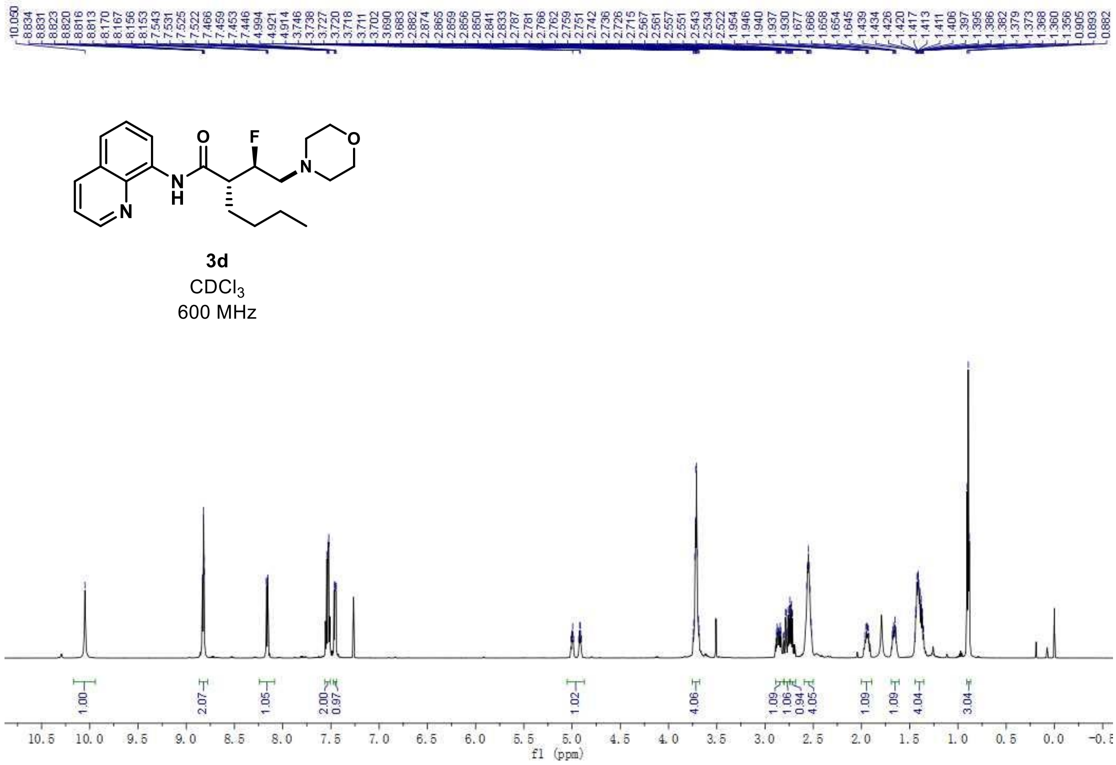

Figure S18. ^{1}H NMR Spectra of **3d**.

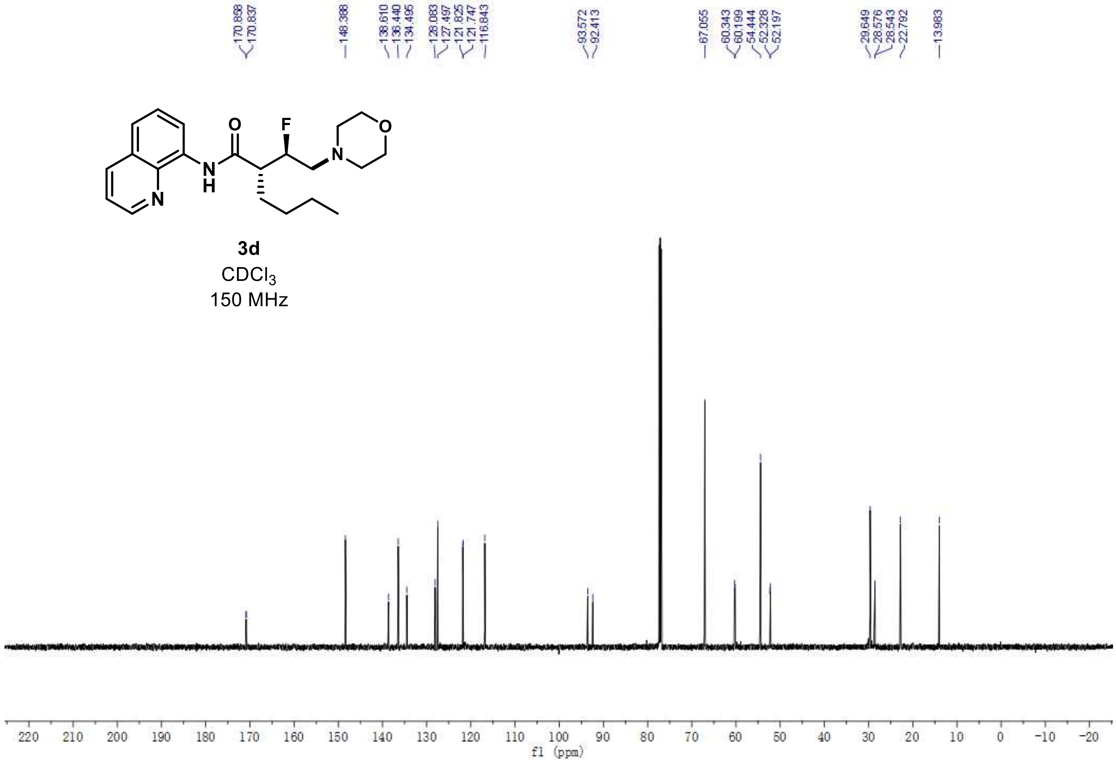

Figure S19. ^{13}C NMR Spectra of **3d**.

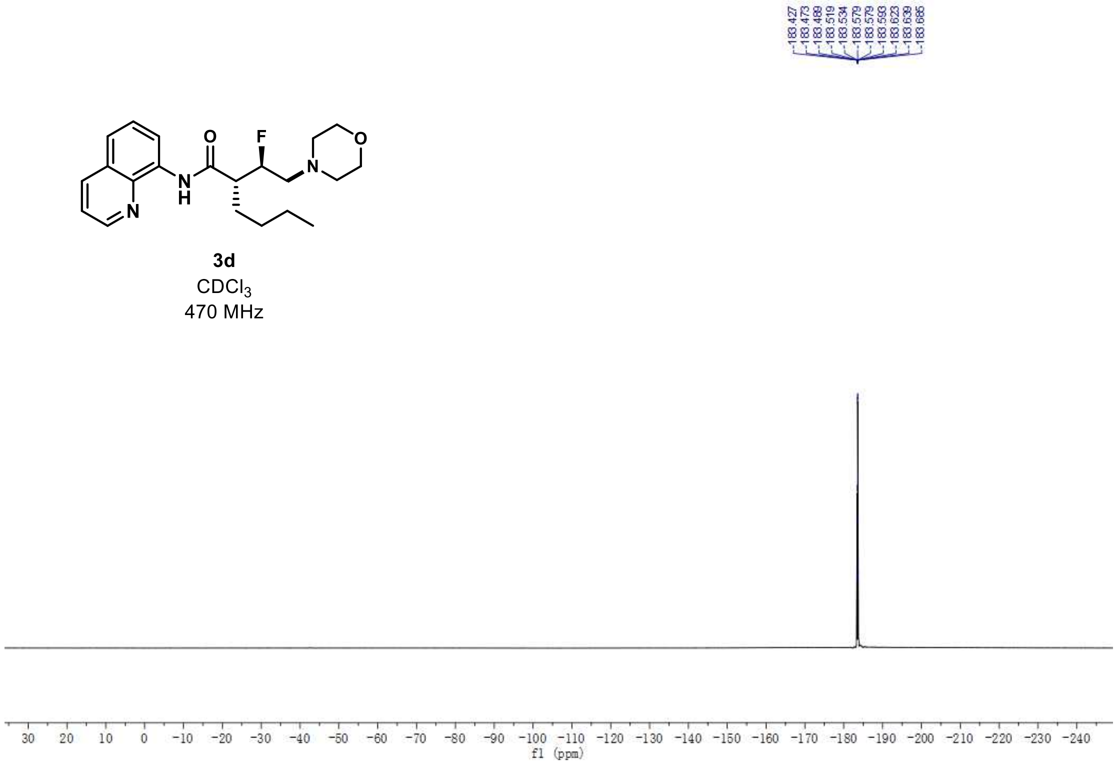

Figure S20. ^{19}F NMR Spectra of **3d**.

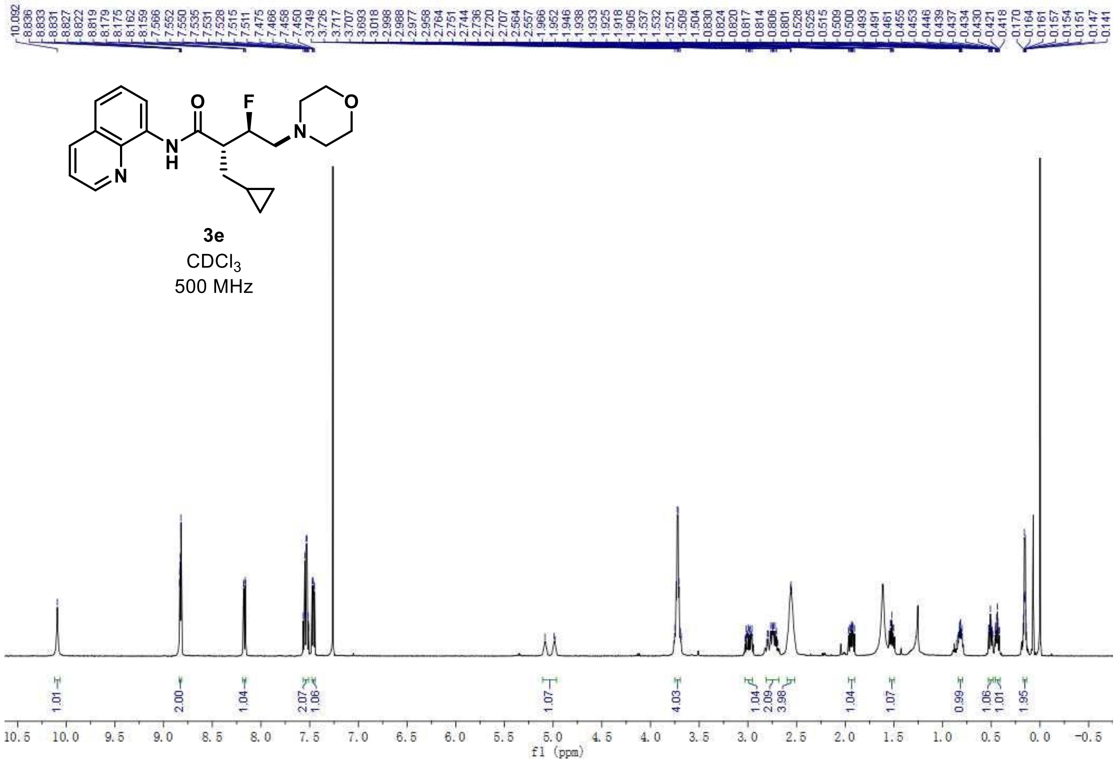

Figure S21. ^{1}H NMR Spectra of **3e**.

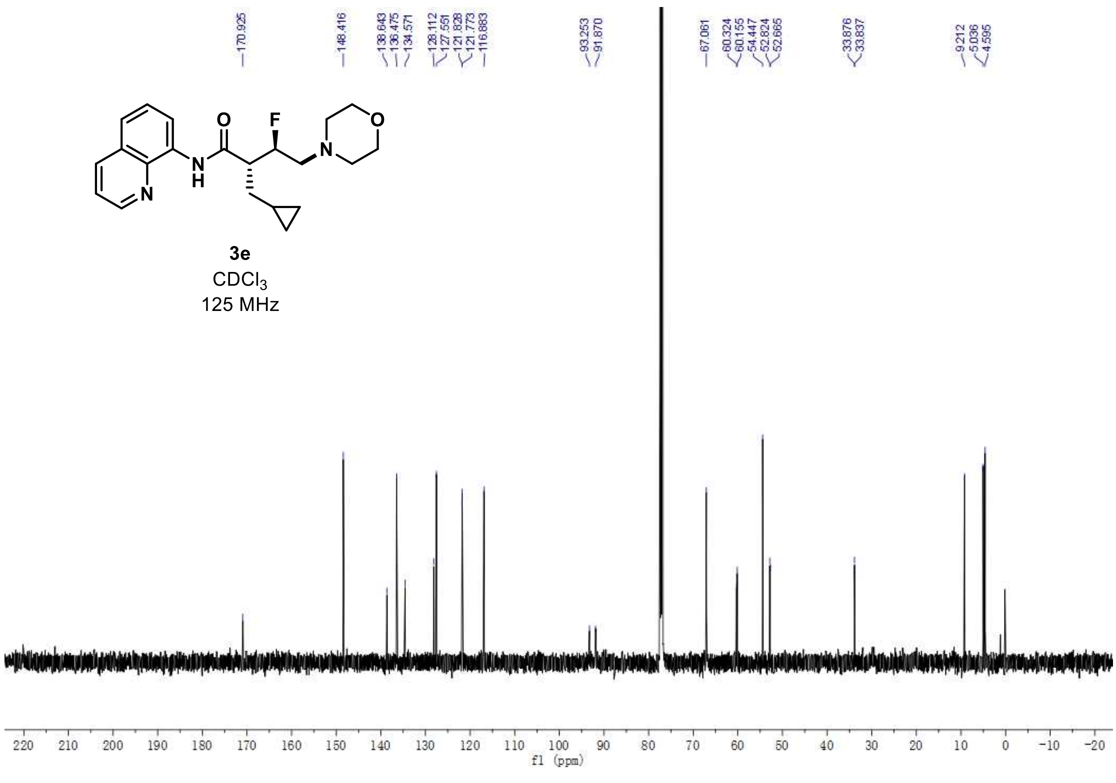

Figure S22. ^{13}C NMR Spectra of **3e**.

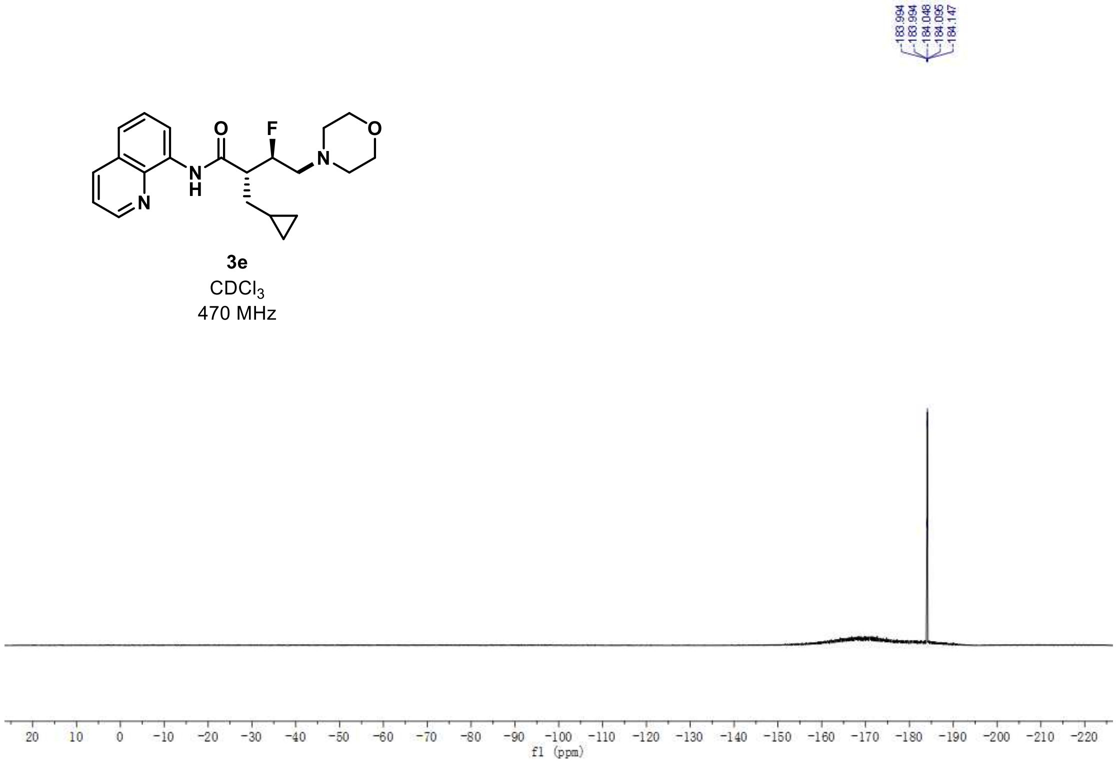

Figure S23. ^{19}F NMR Spectra of **3e**.

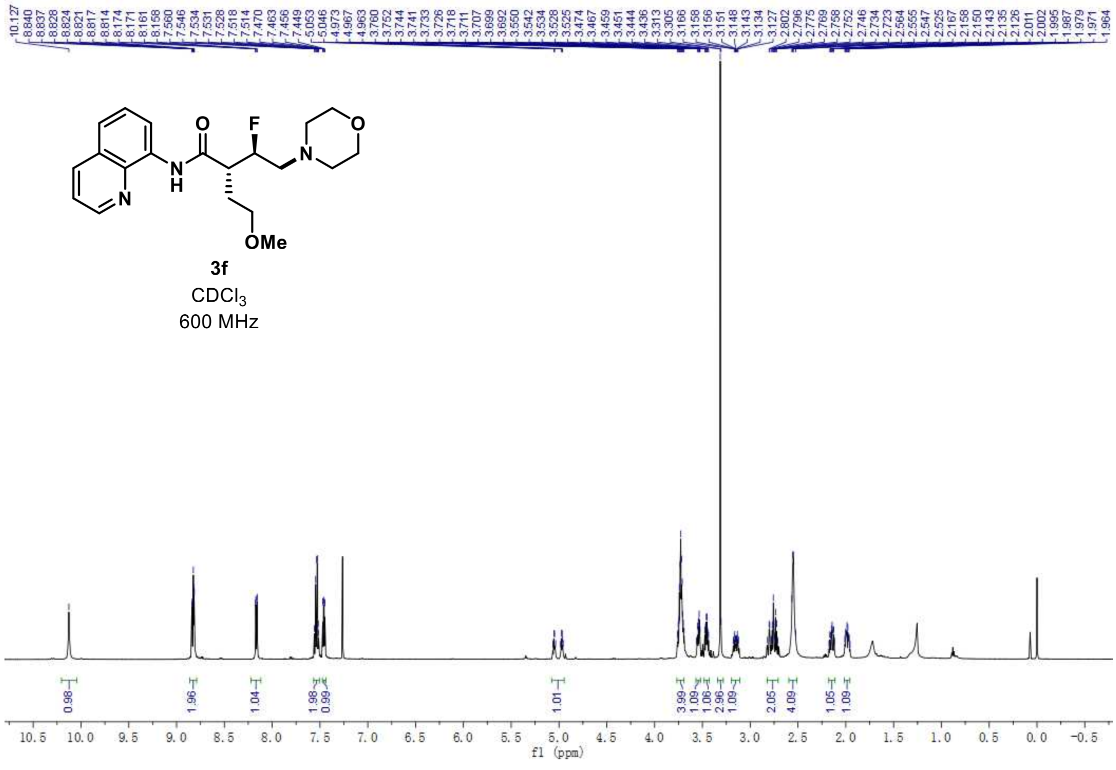

Figure S24. ^{1}H NMR Spectra of **3f**.

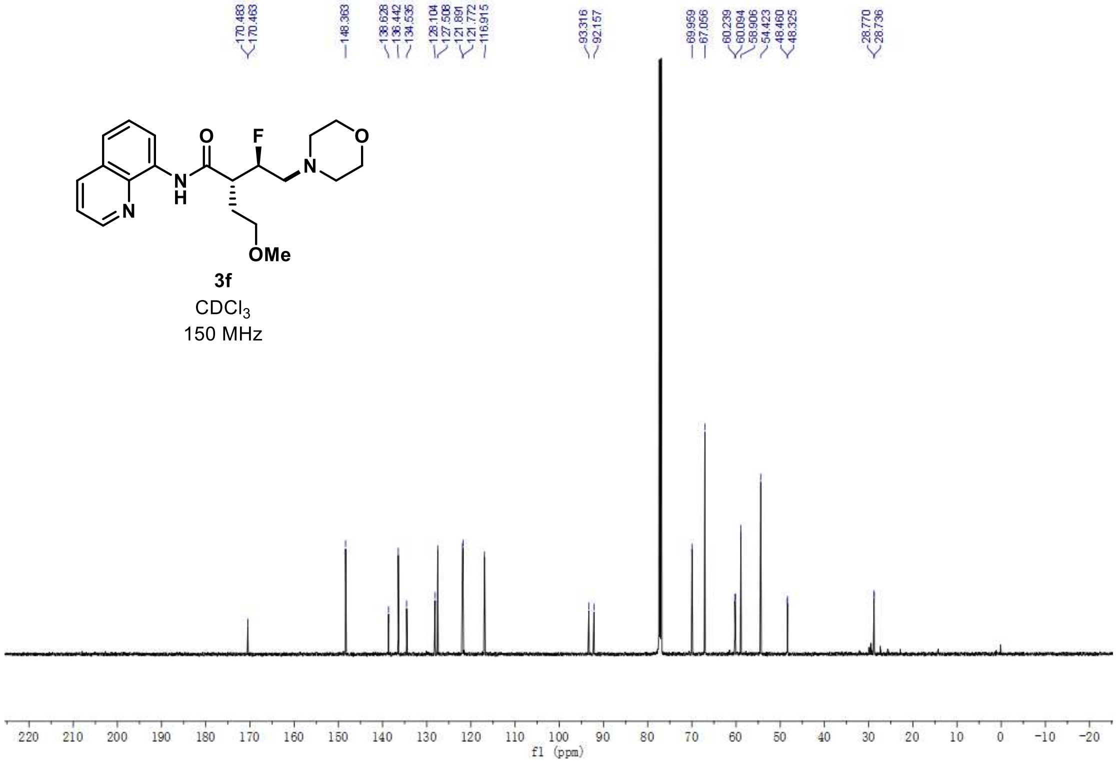

Figure S25. ^{13}C NMR Spectra of **3f**.

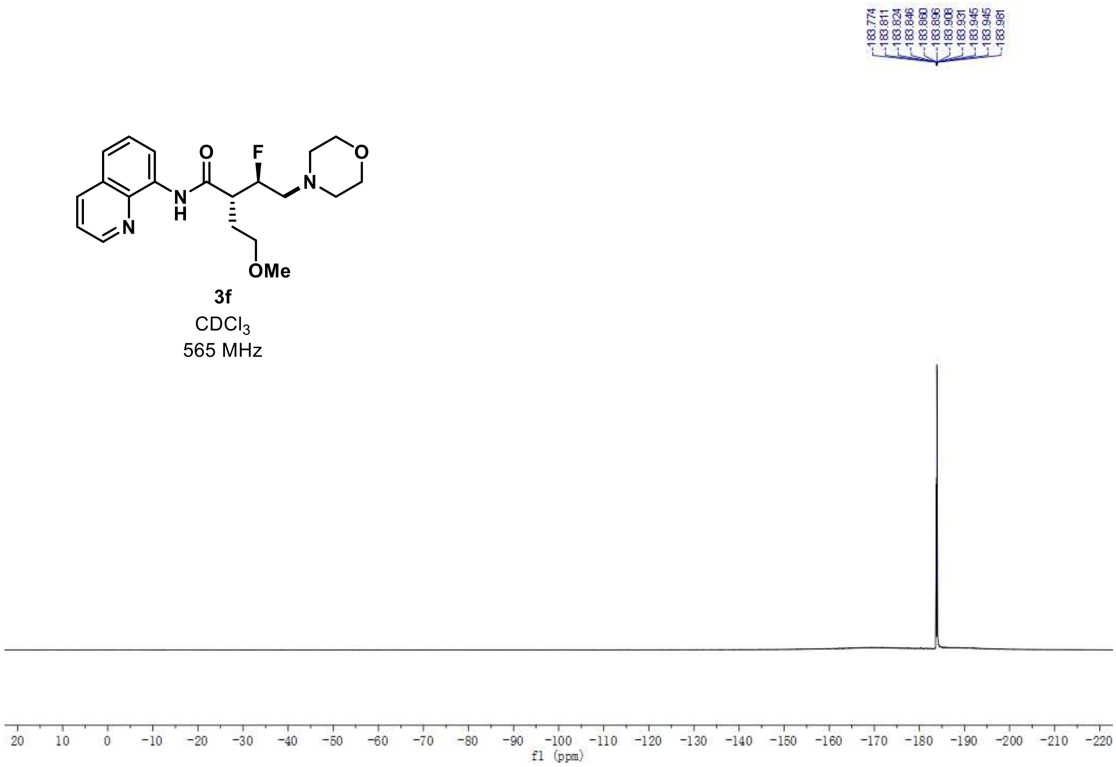

Figure S26. ^{19}F NMR Spectra of **3f**.

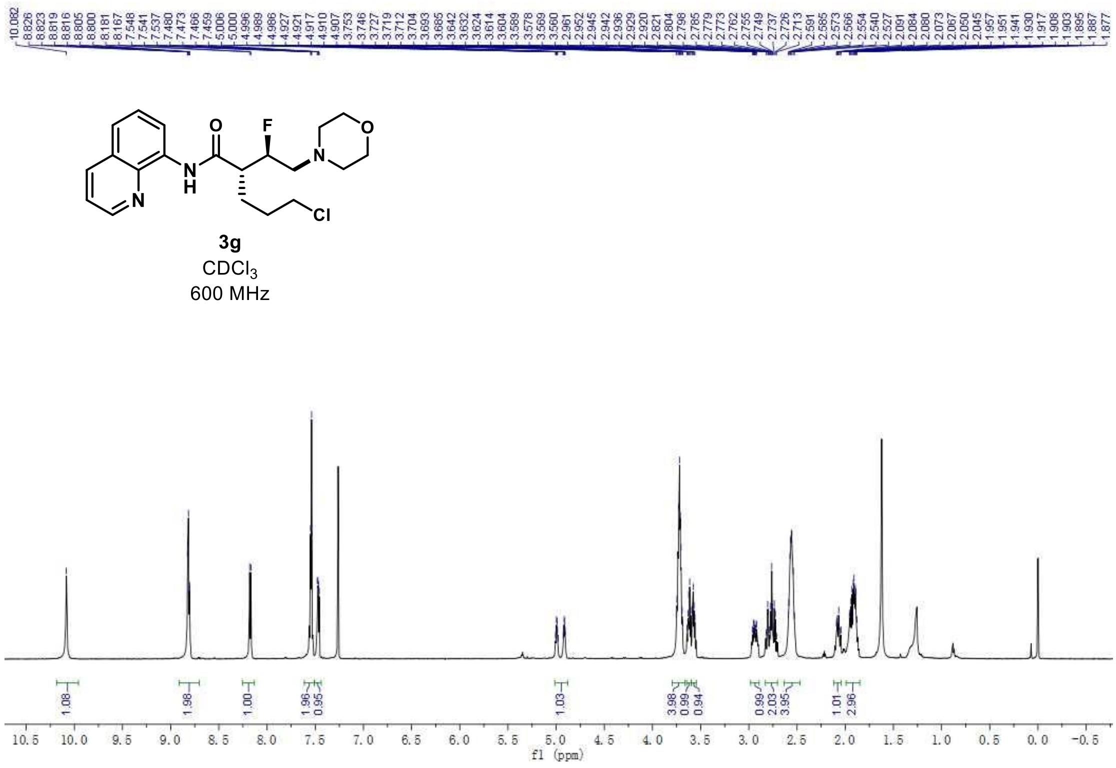

Figure S27. ^{1}H NMR Spectra of **3g**.

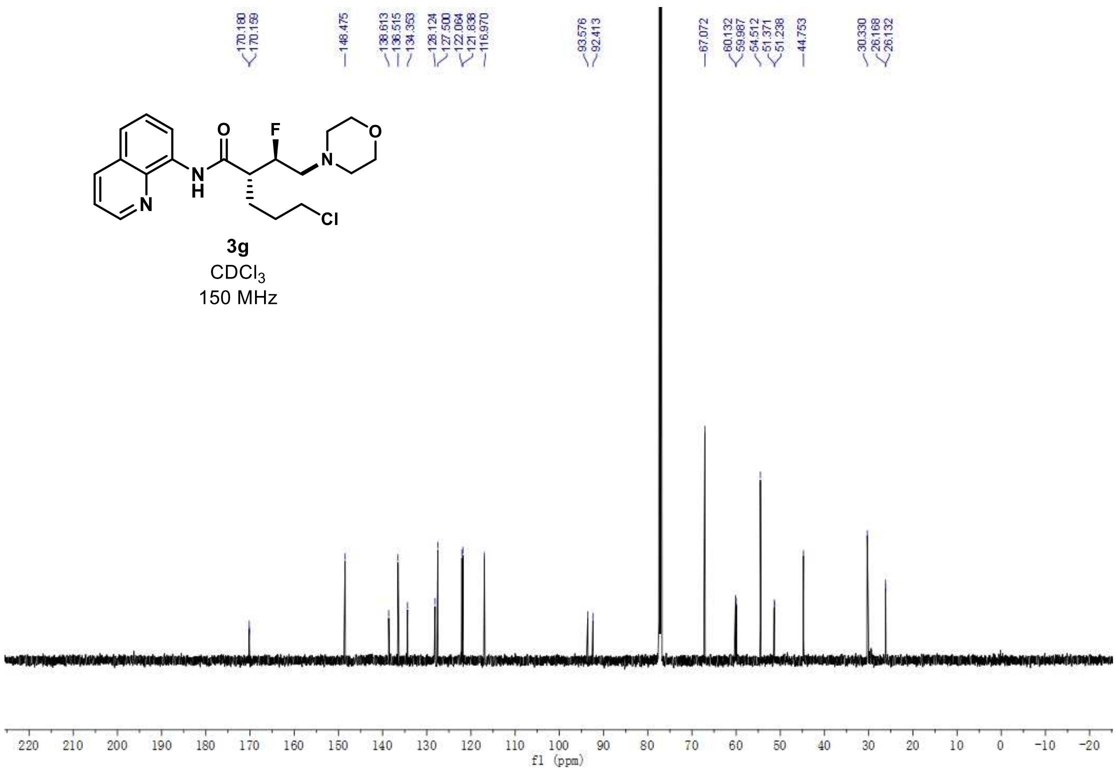

Figure S28. ^{13}C NMR Spectra of **3g**.

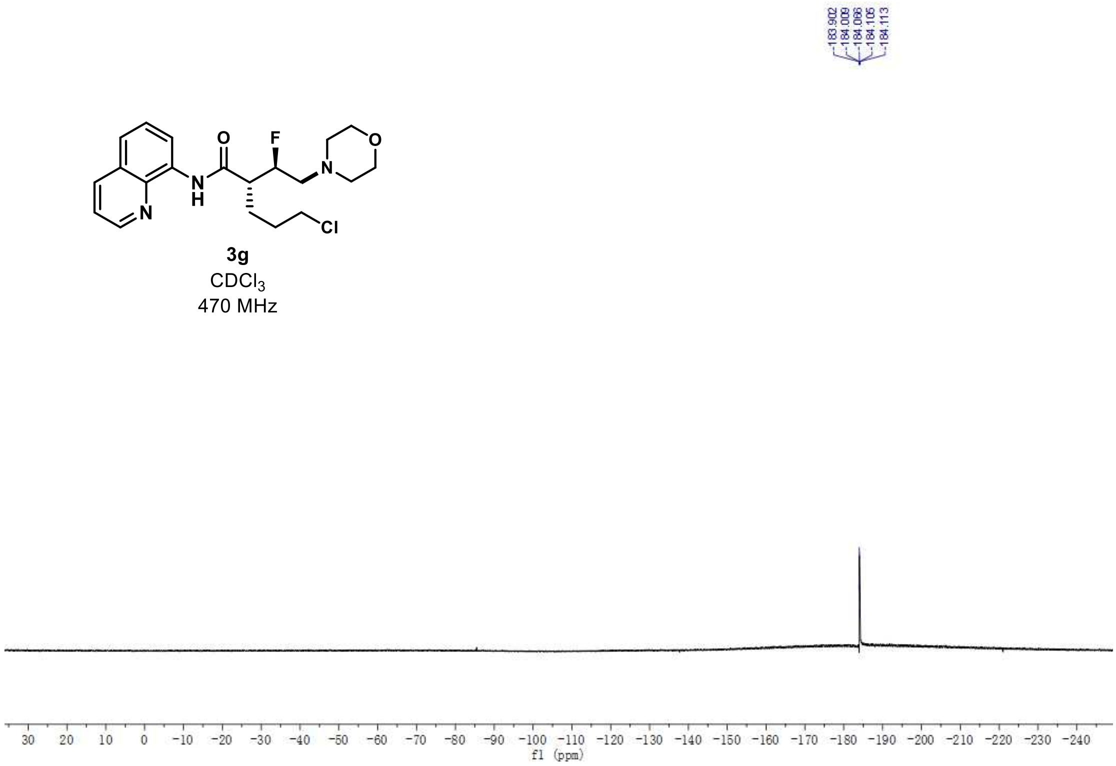

Figure S29. ^{19}F NMR Spectra of **3g**.

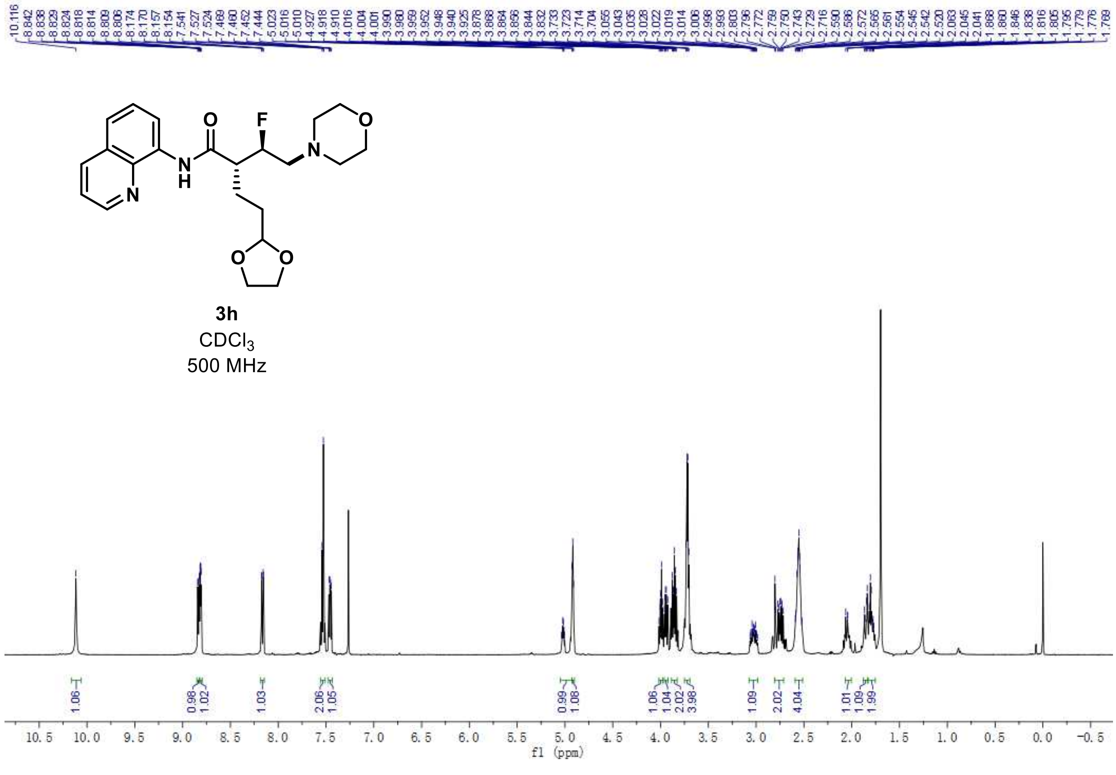

Figure S30. ^{1}H NMR Spectra of **3h**.

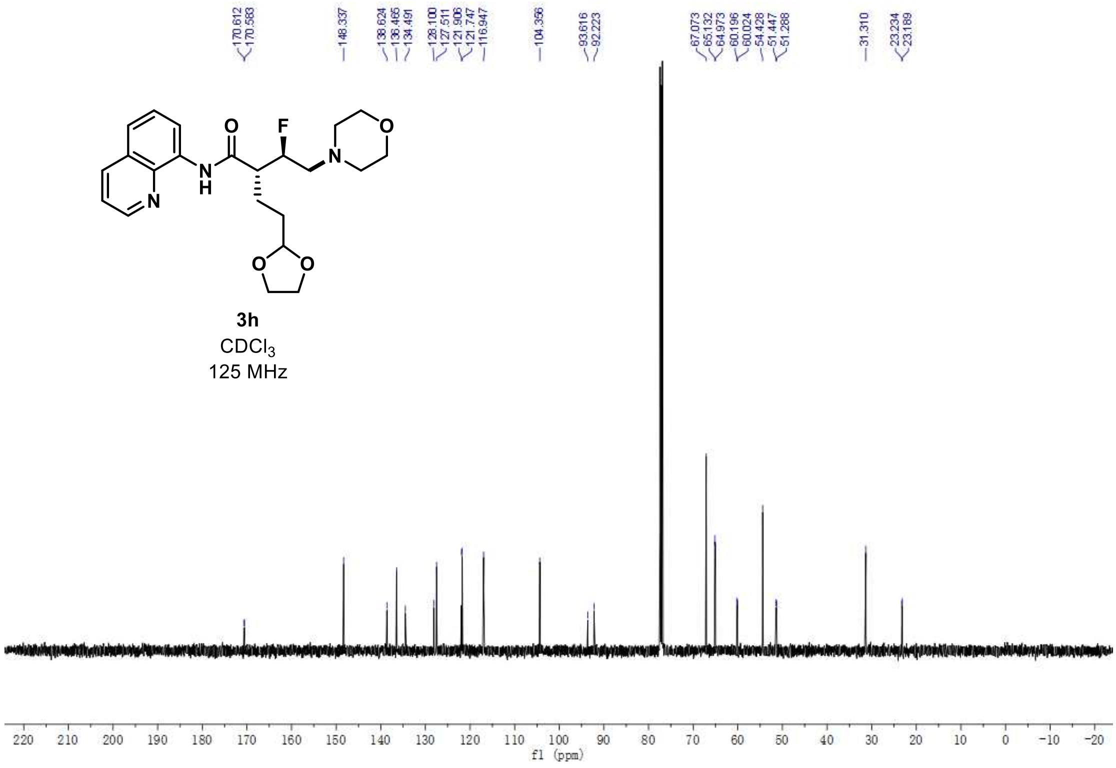

Figure S31. ^{13}C NMR Spectra of **3h**.

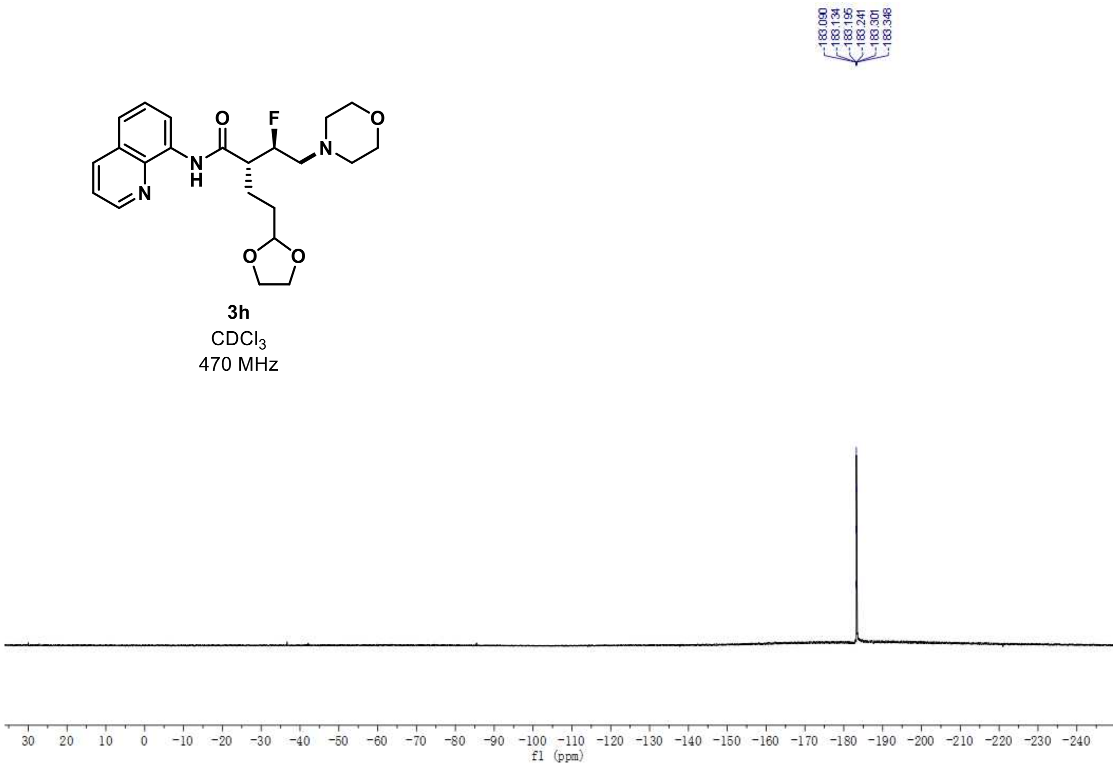

Figure S32. ^{19}F NMR Spectra of **3h**.

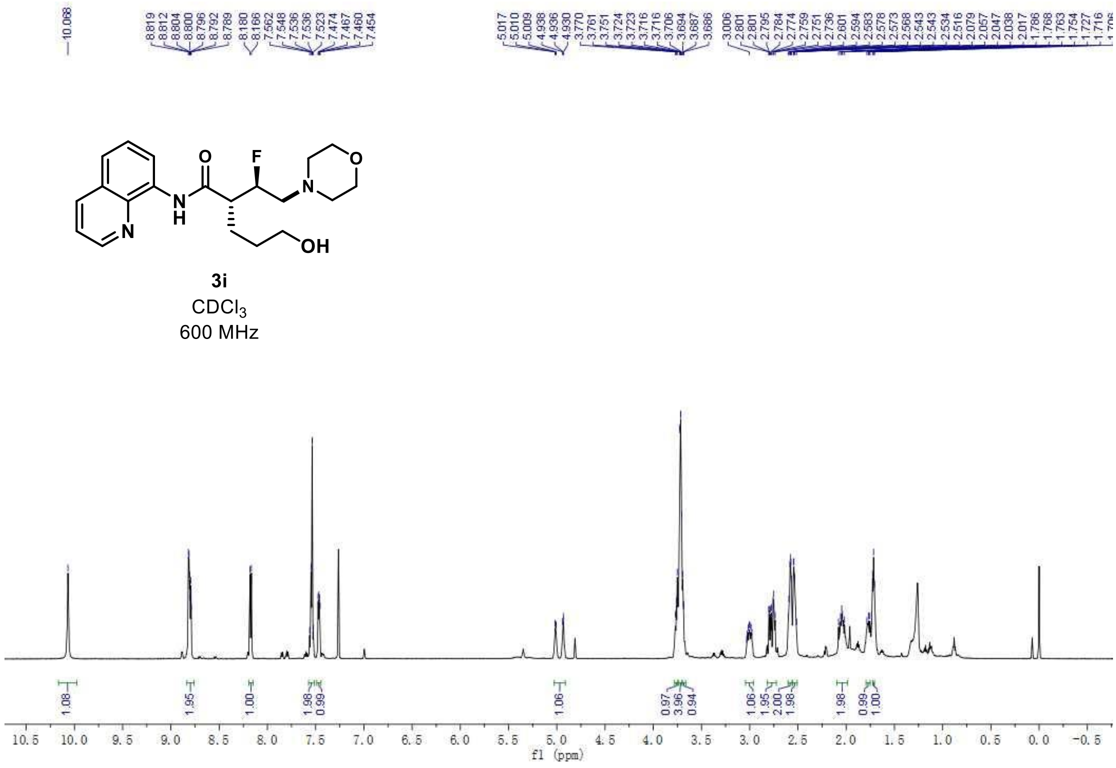

Figure S33. ^{1}H NMR Spectra of **3i**.

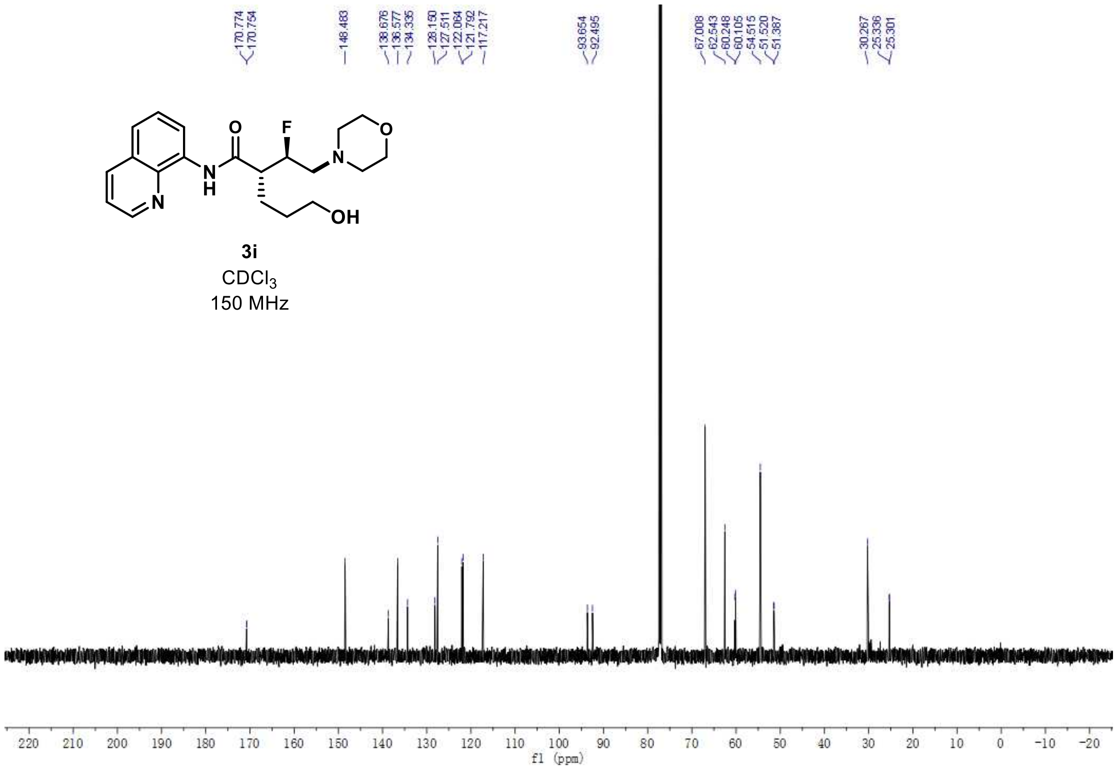

Figure S34. ^{13}C NMR Spectra of **3i**.

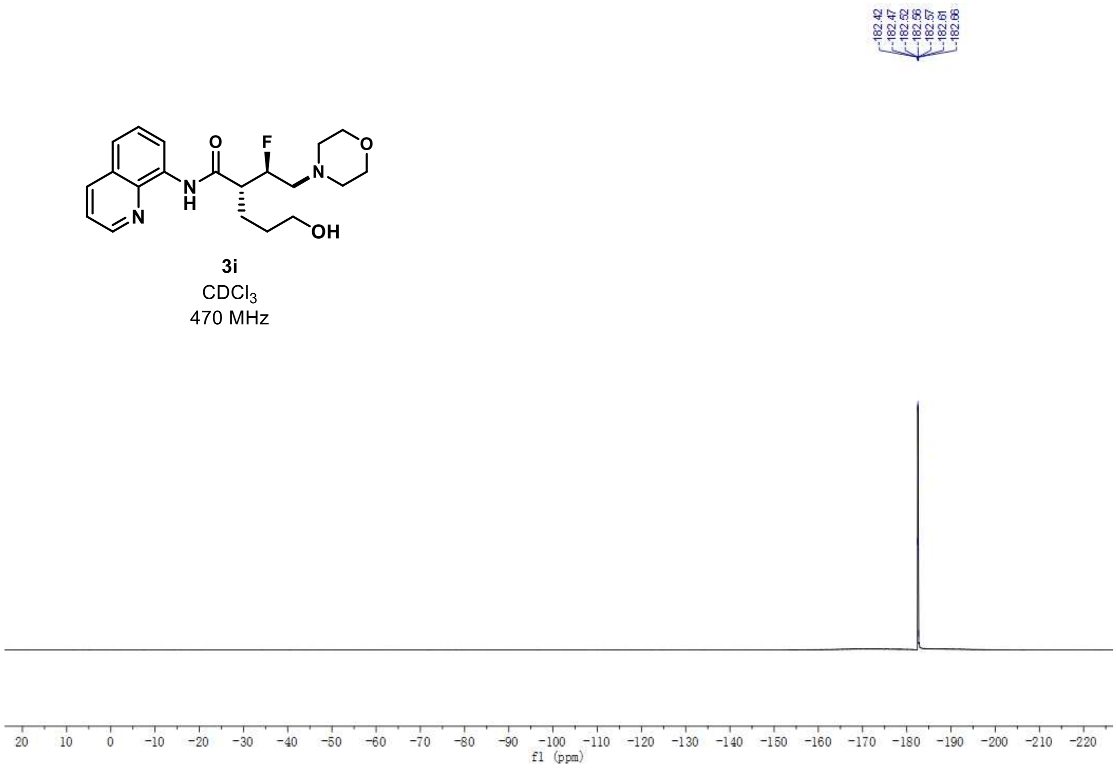

Figure S35. ^{19}F NMR Spectra of **3i**.

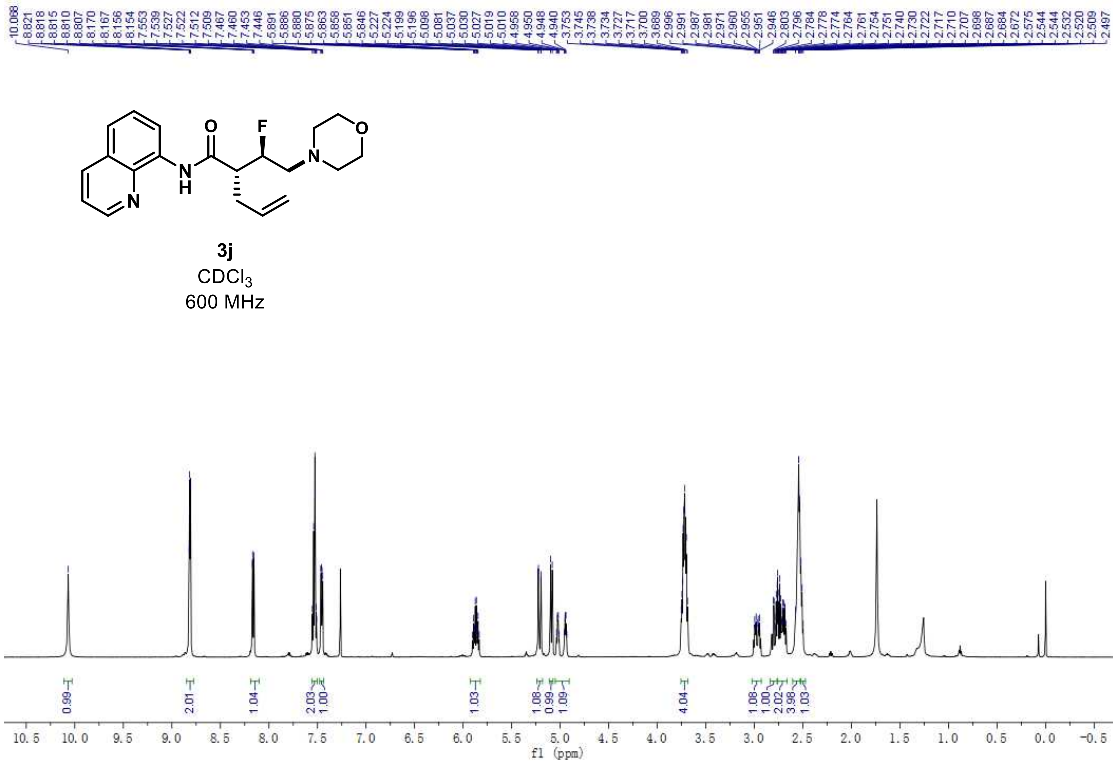

Figure S36. ^{1}H NMR Spectra of **3j**.

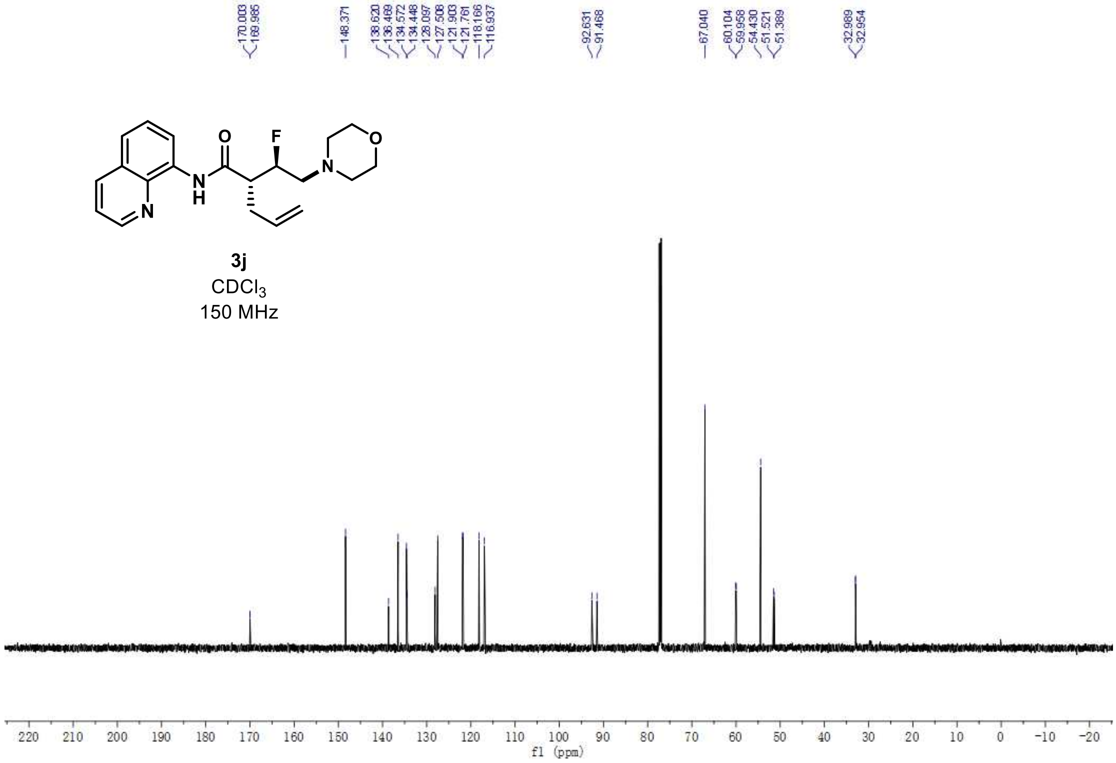

Figure S37. ^{13}C NMR Spectra of **3j**.

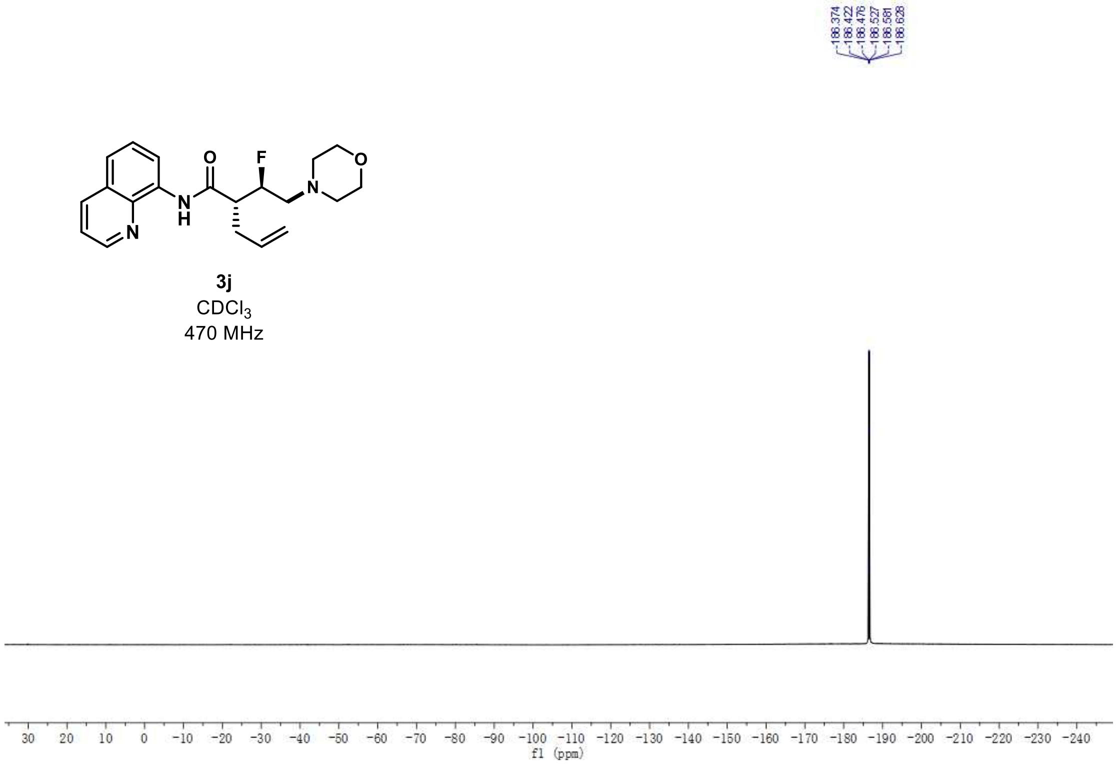

Figure S38. ^{19}F NMR Spectra of **3j**.

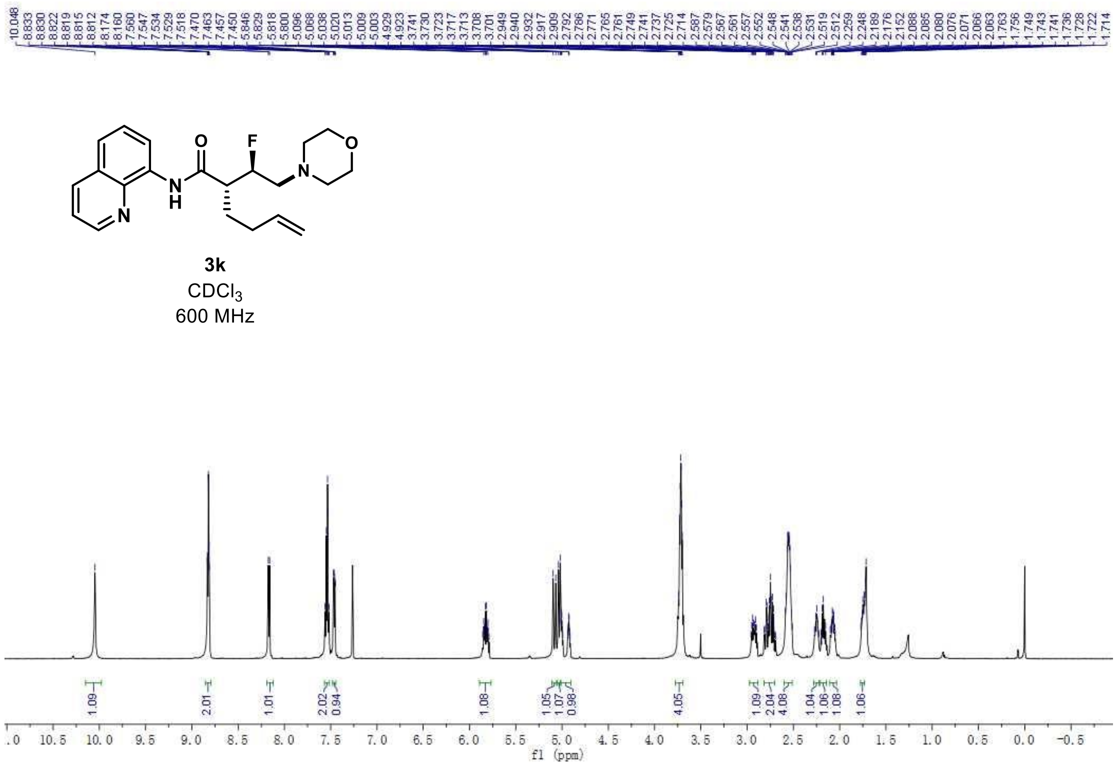

Figure S39. ^{1}H NMR Spectra of **3k**.

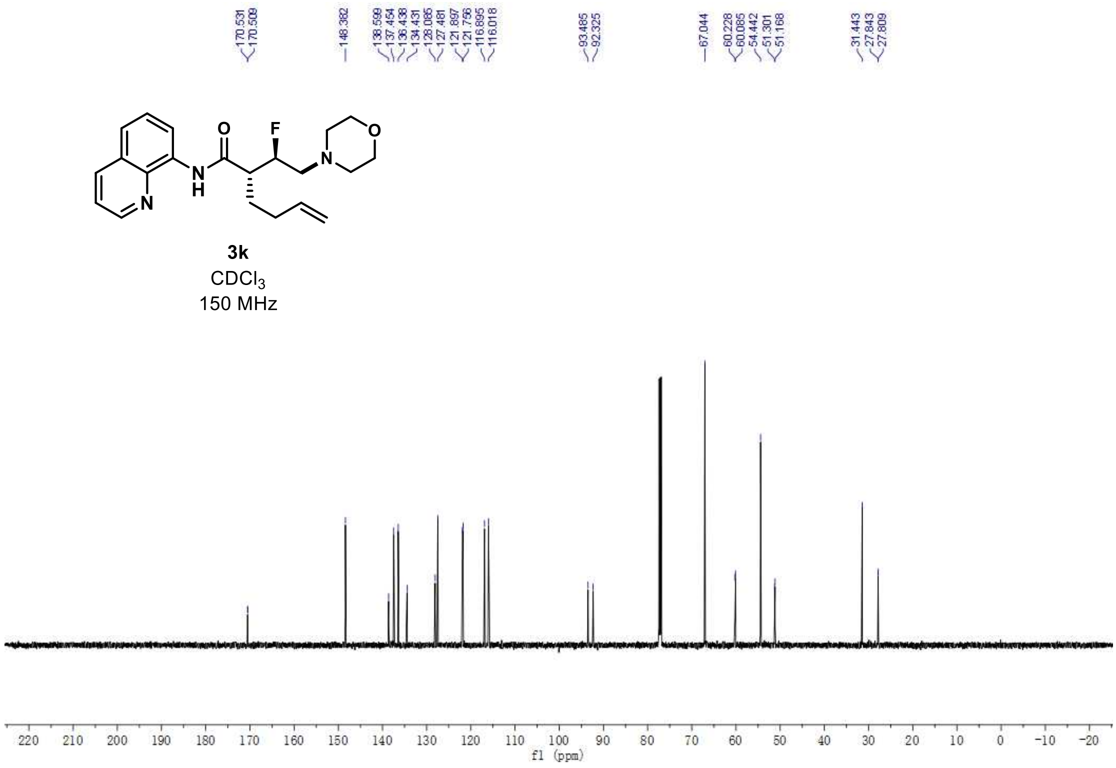

Figure S40. ^{13}C NMR Spectra of **3k**.

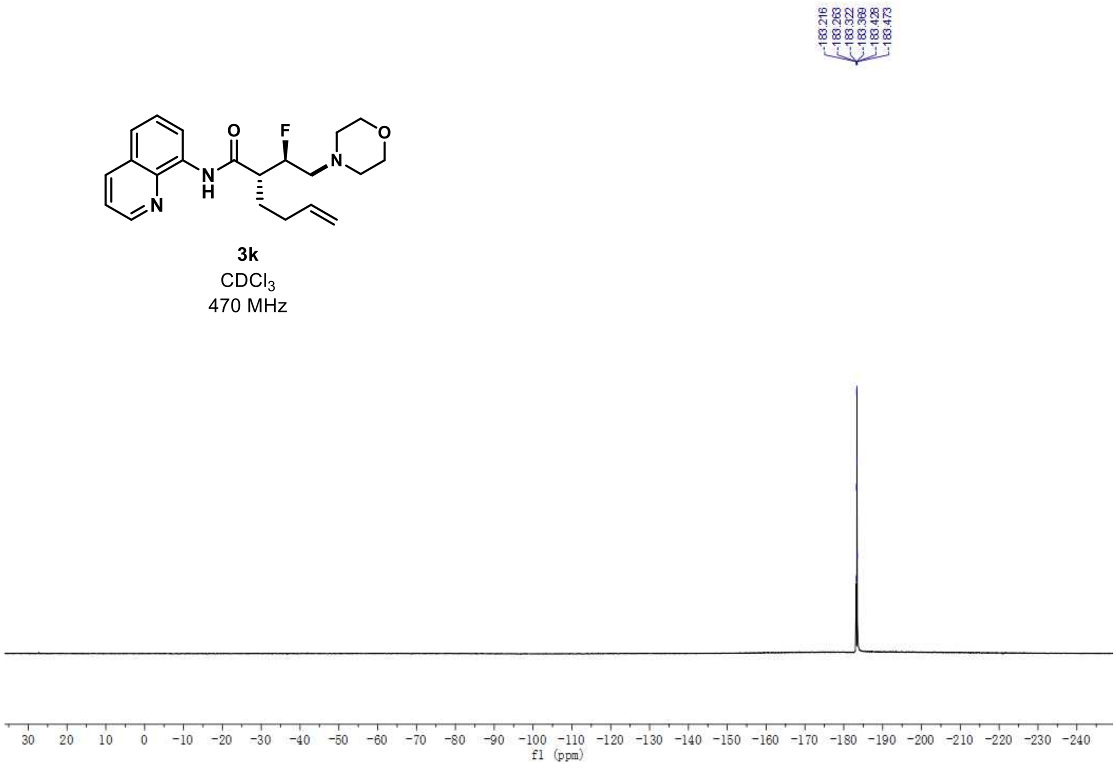

Figure S41. ^{19}F NMR Spectra of **3k**.

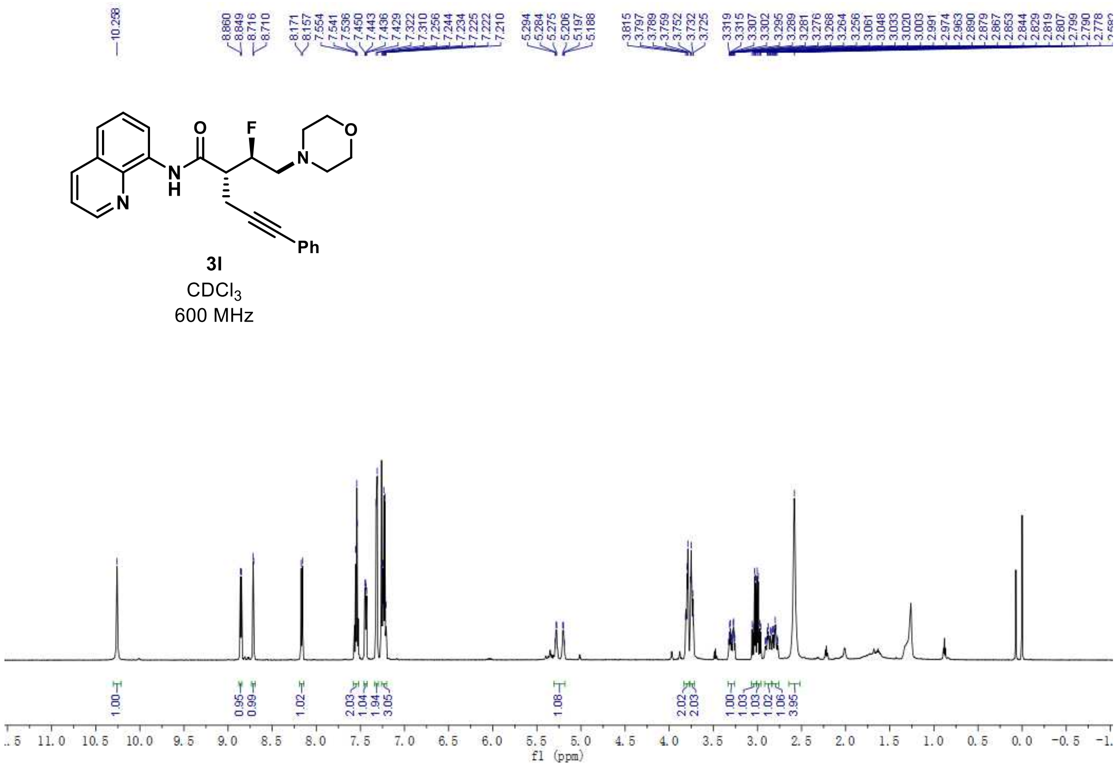

Figure S42. ^{1}H NMR Spectra of **3l**.

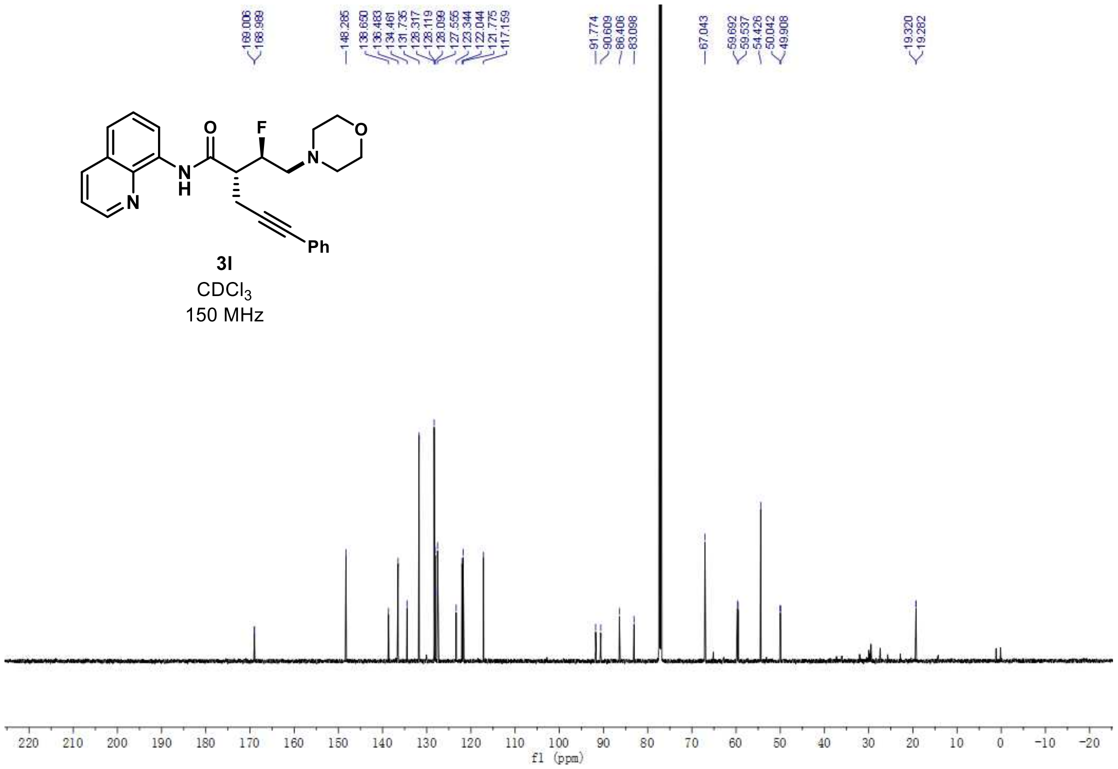

Figure S43. ^{13}C NMR Spectra of **3l**.

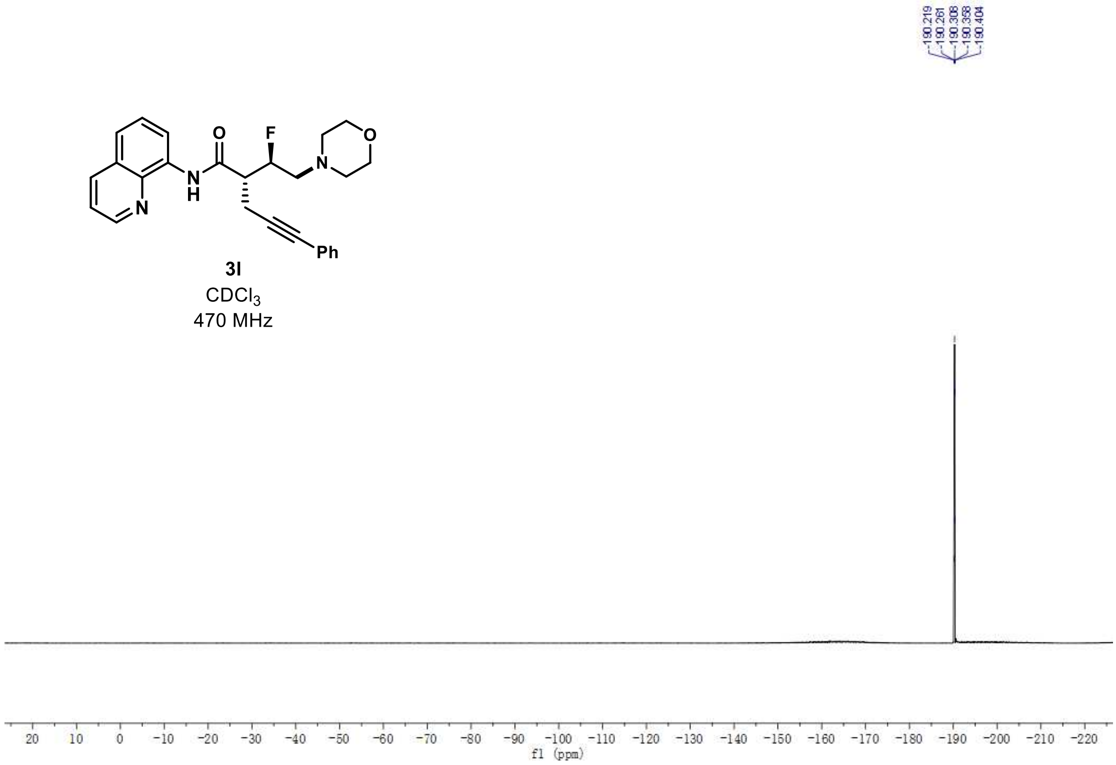

Figure S44. ^{19}F NMR Spectra of **3l**.

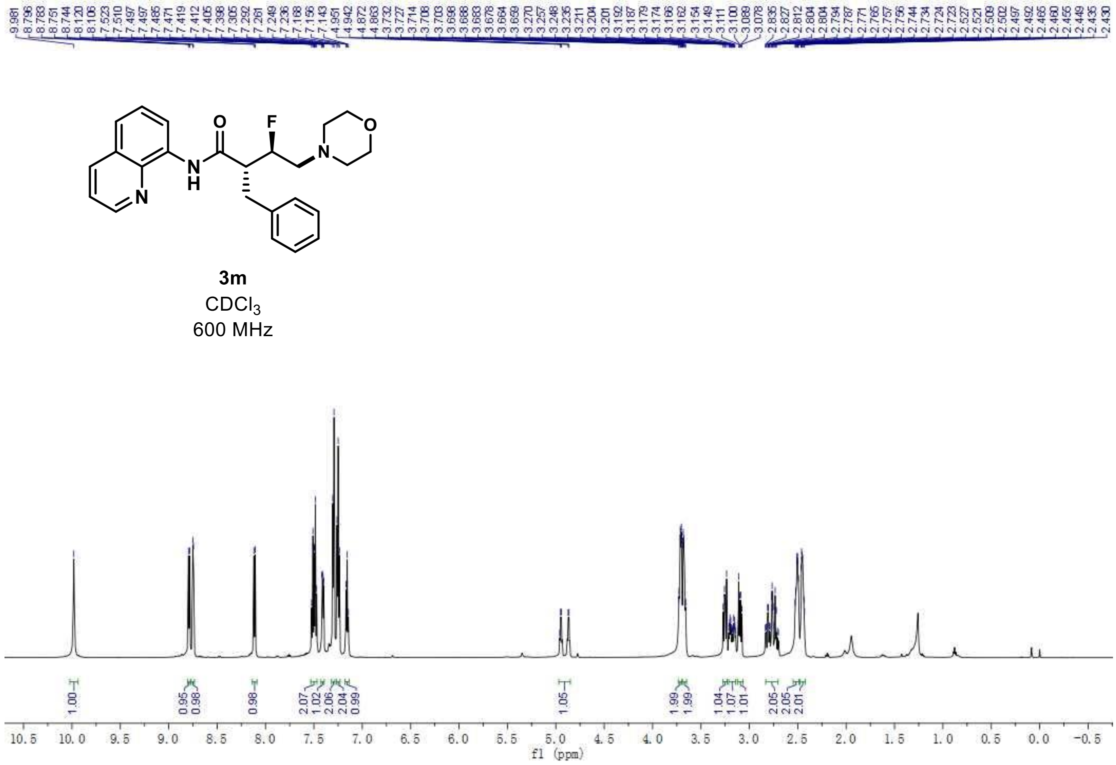

Figure S45. ^{1}H NMR Spectra of **3m**.

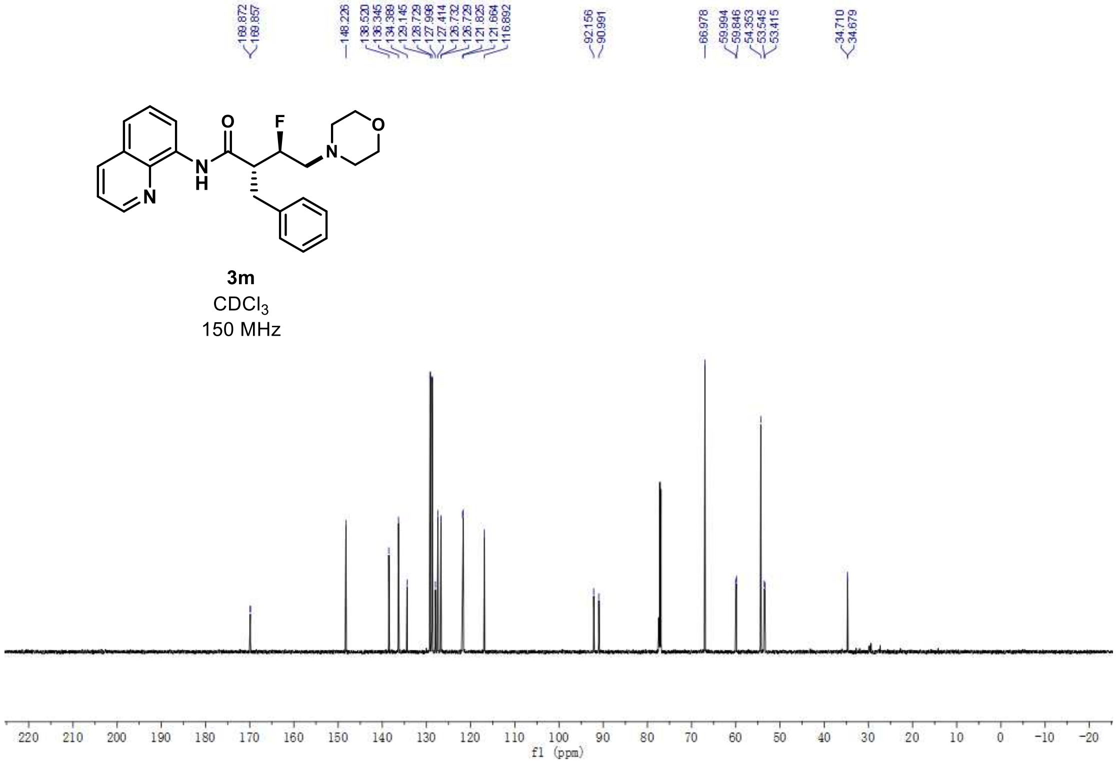

Figure S46. ^{13}C NMR Spectra of **3m**.

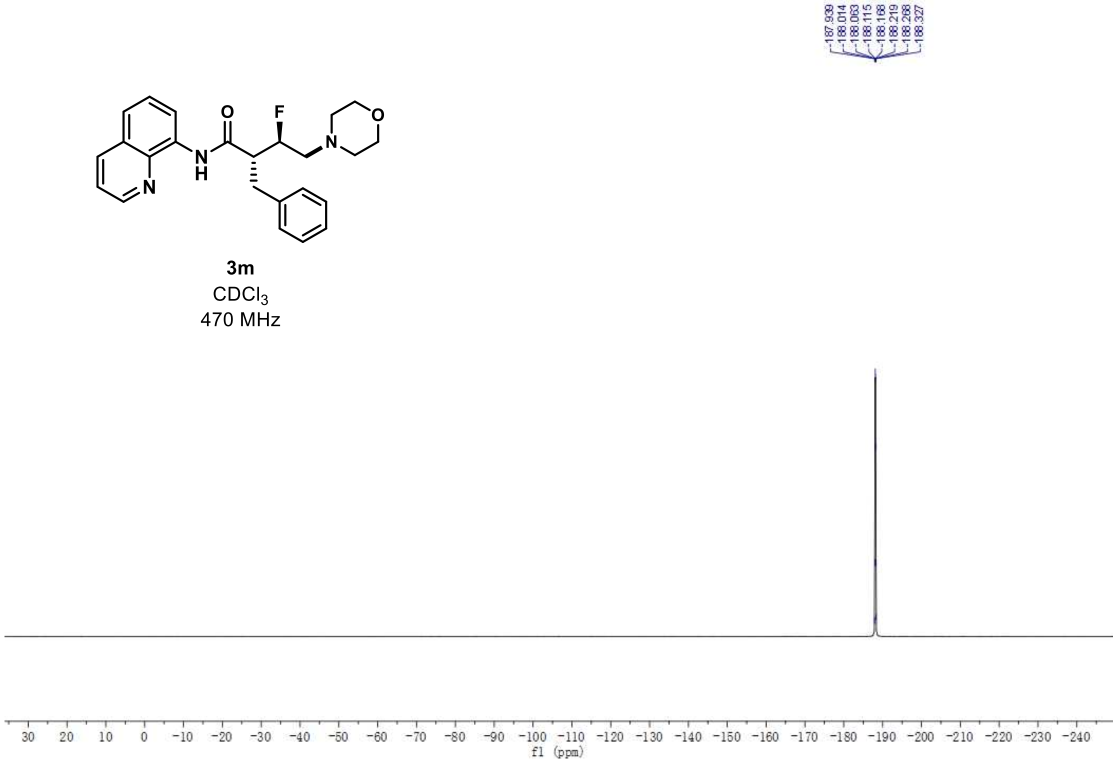

Figure S47. ^{19}F NMR Spectra of **3m**.

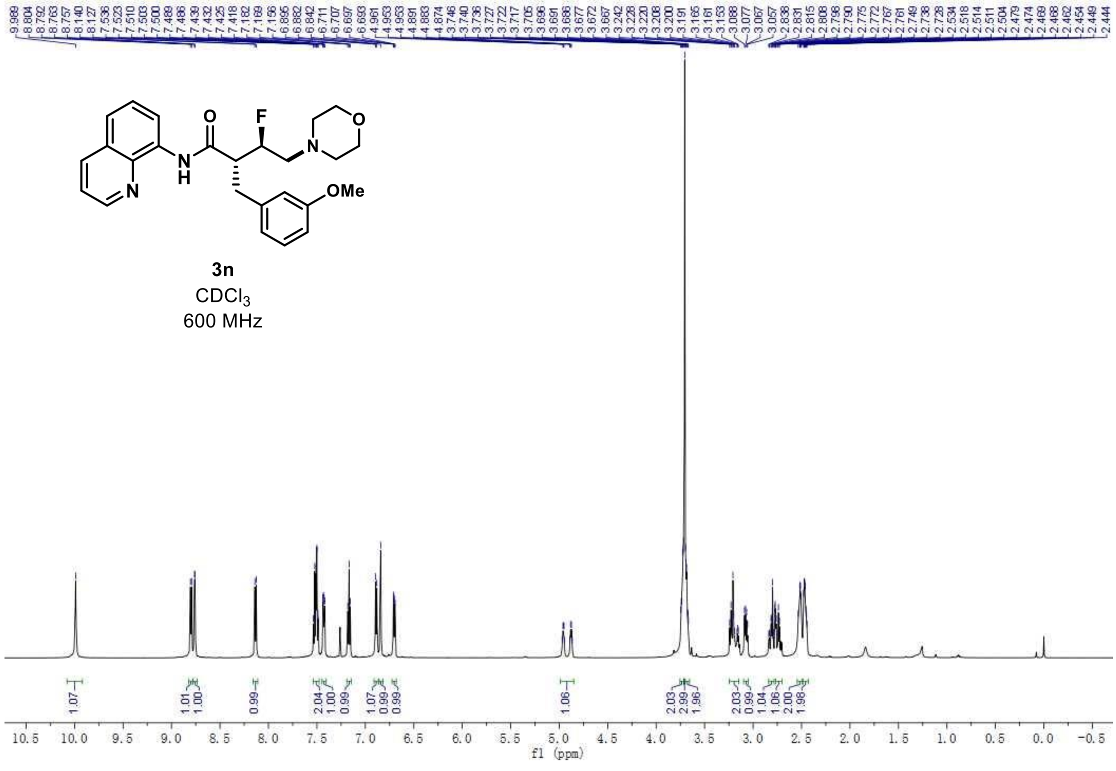

Figure S48. ^{1}H NMR Spectra of **3n**.

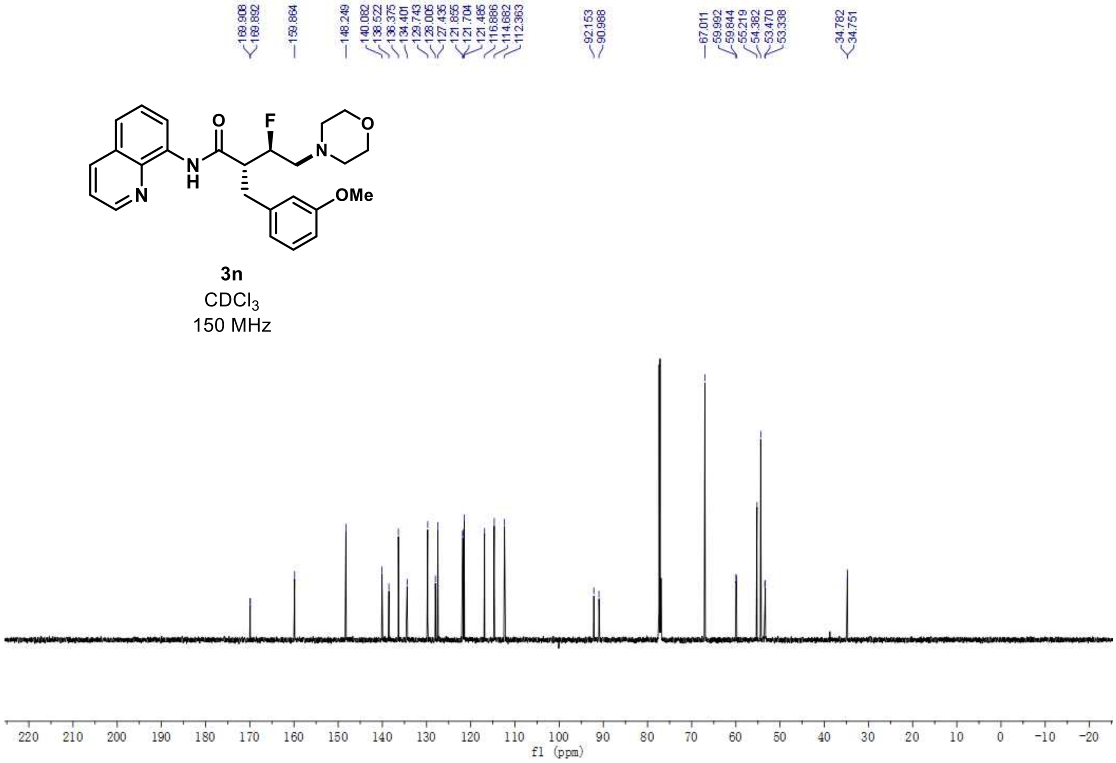

Figure S49. ^{13}C NMR Spectra of **3n**.

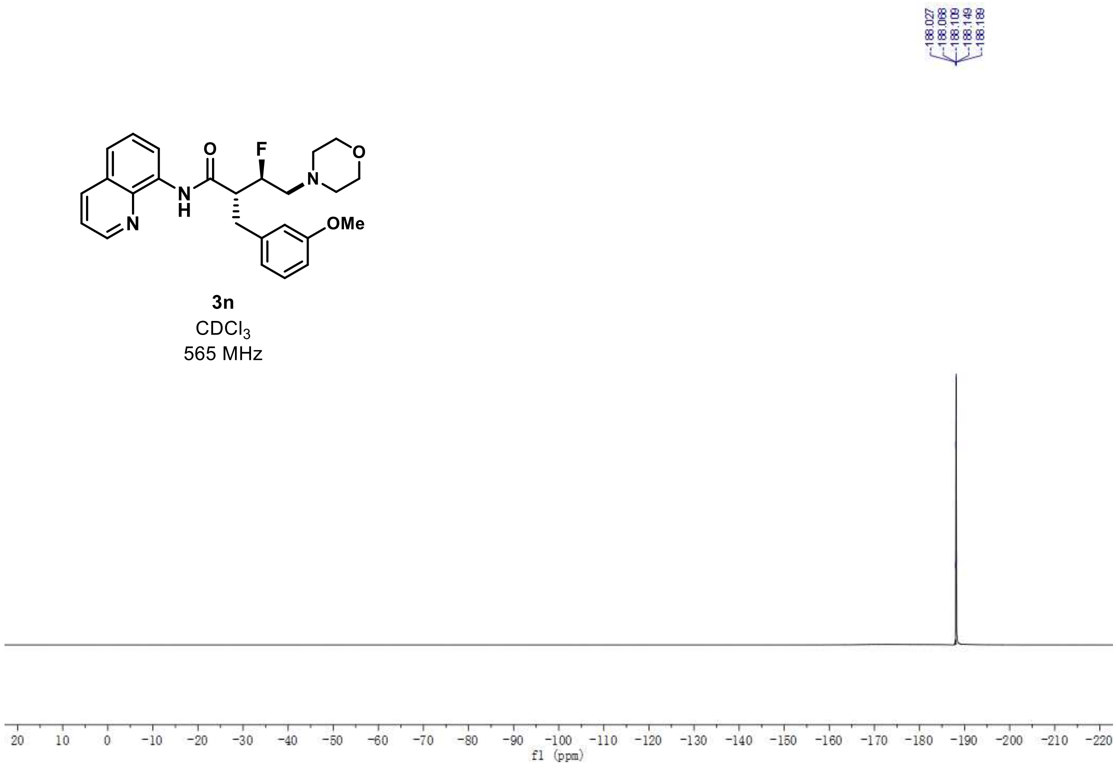

Figure S50. ^{19}F NMR Spectra of **3n**.

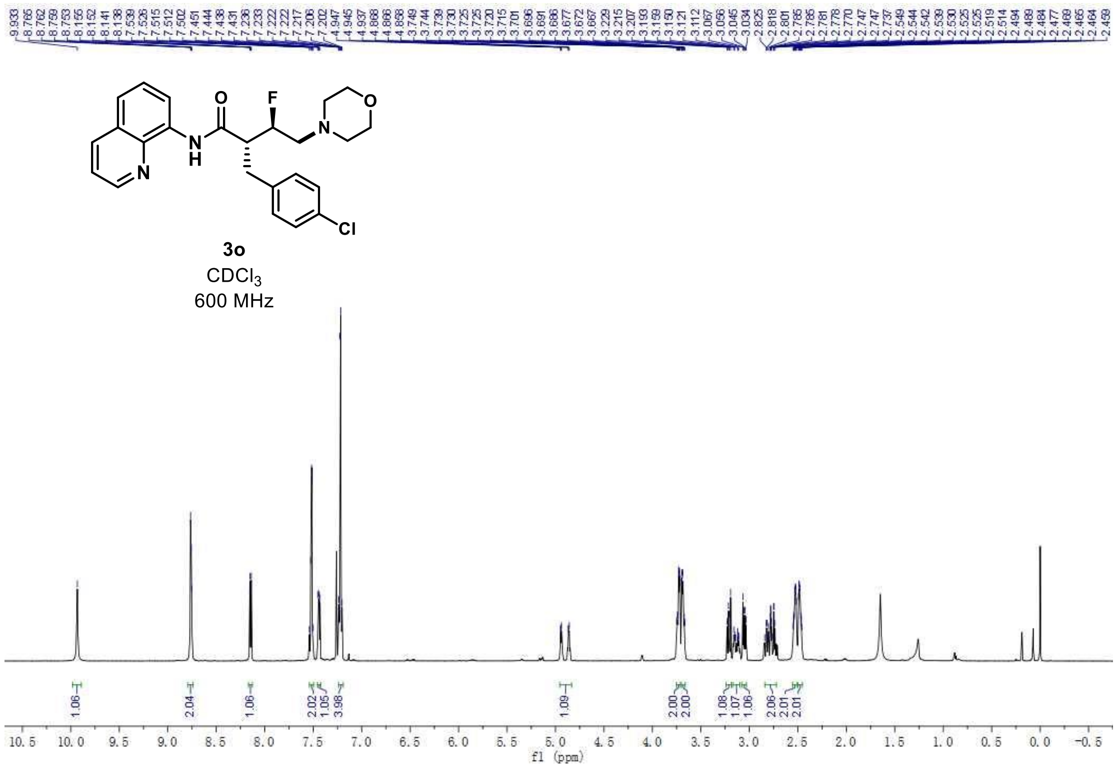

Figure S51. ^{1}H NMR Spectra of **3o**.

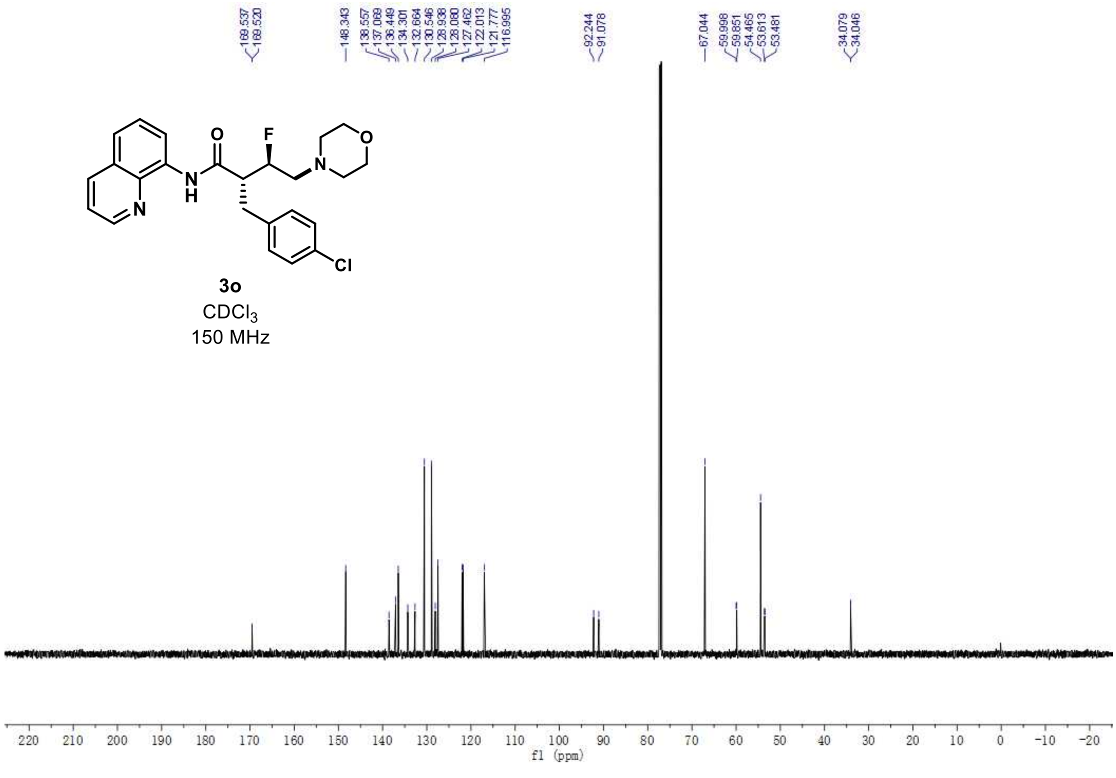

Figure S52. ^{13}C NMR Spectra of **3o**.

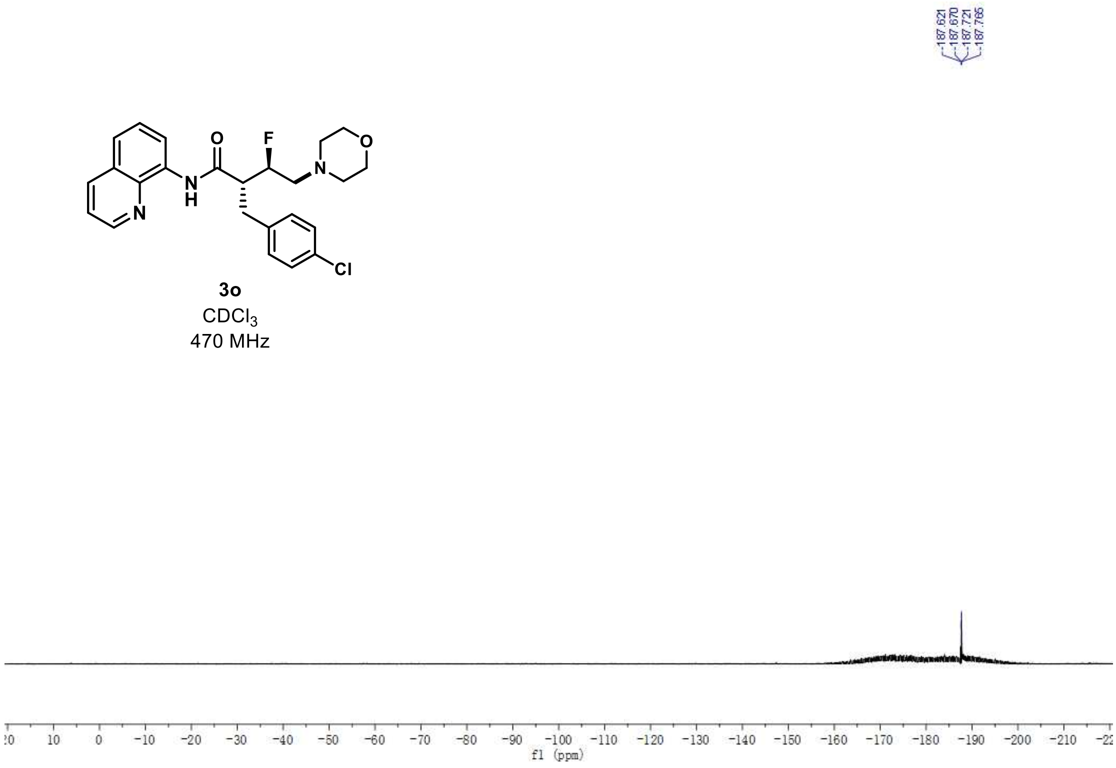

Figure S53. ^{19}F NMR Spectra of **3o**.

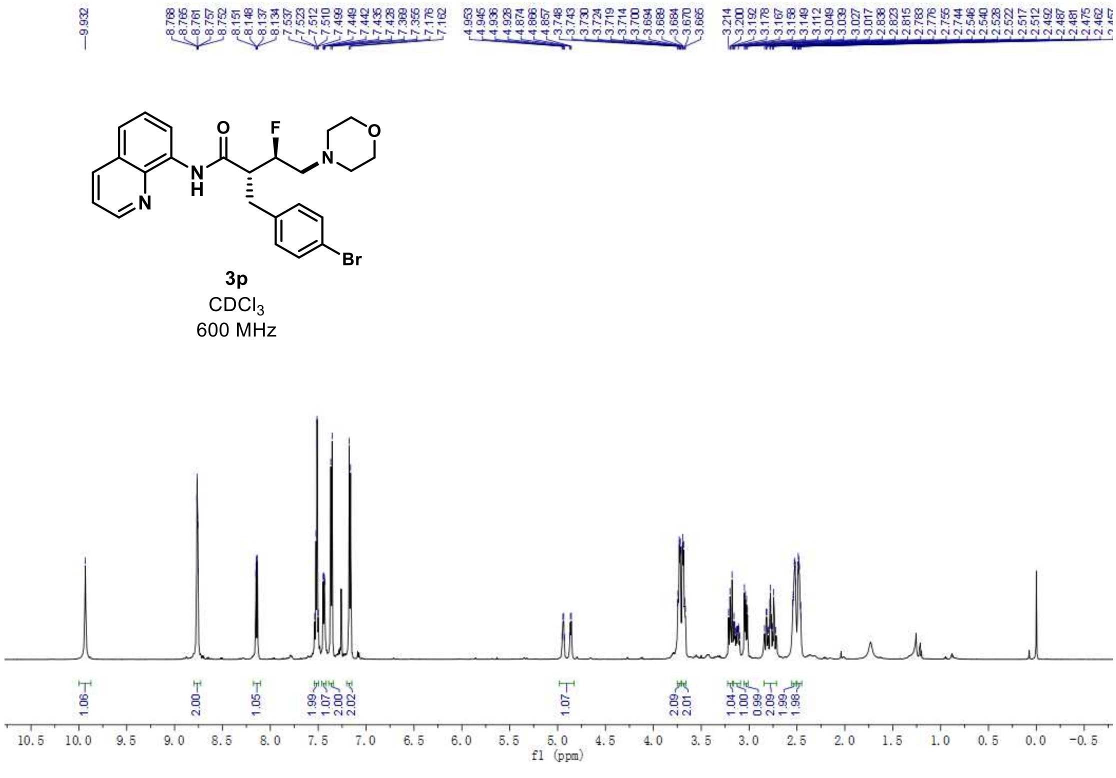

Figure S54. ^{1}H NMR Spectra of **3p**.

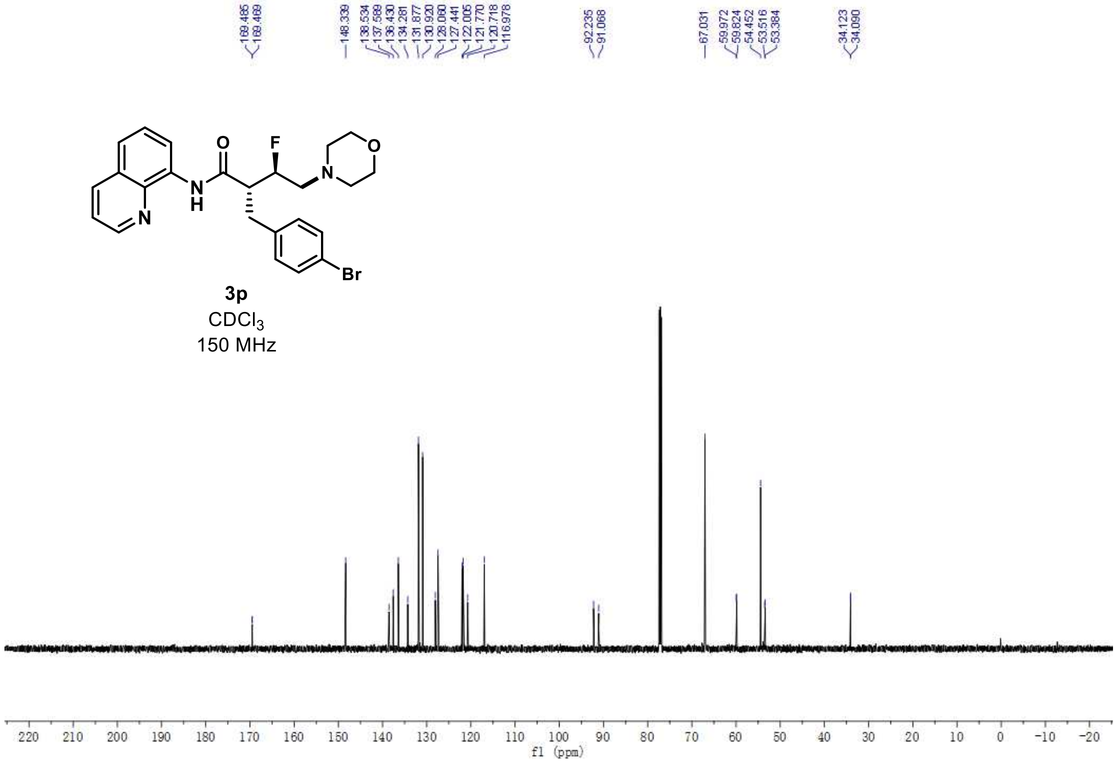

Figure S55. ^{13}C NMR Spectra of **3p**.

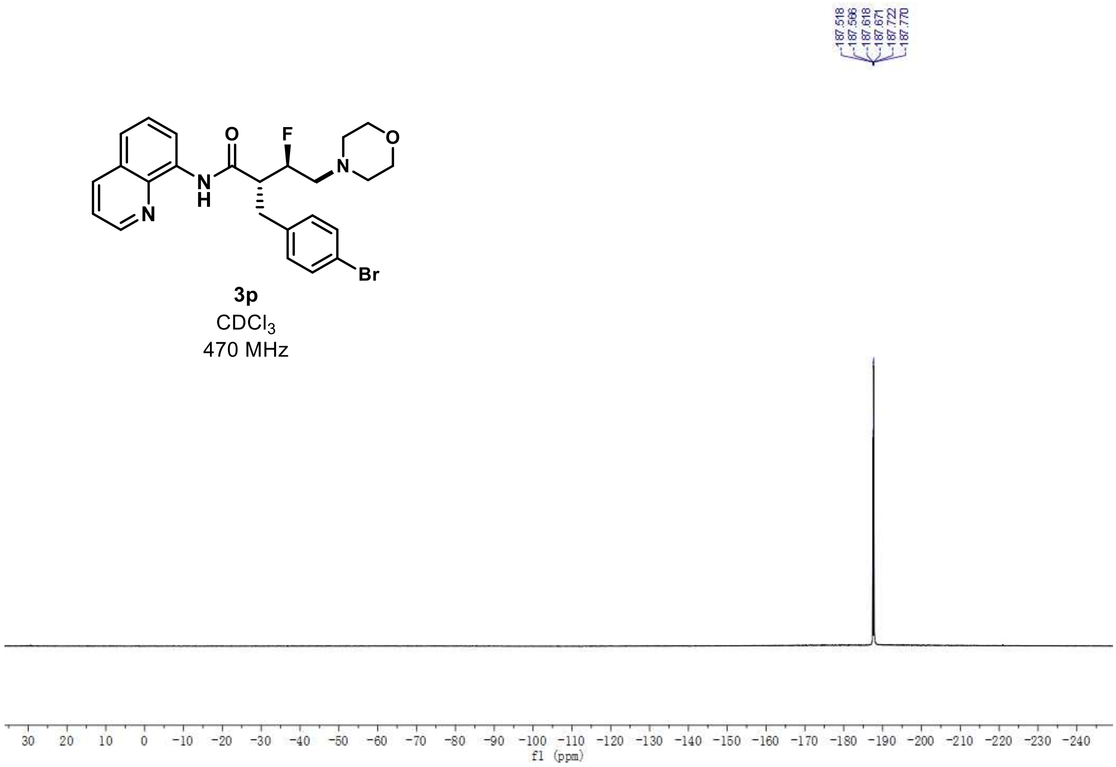

Figure S56. ^{19}F NMR Spectra of **3b**.

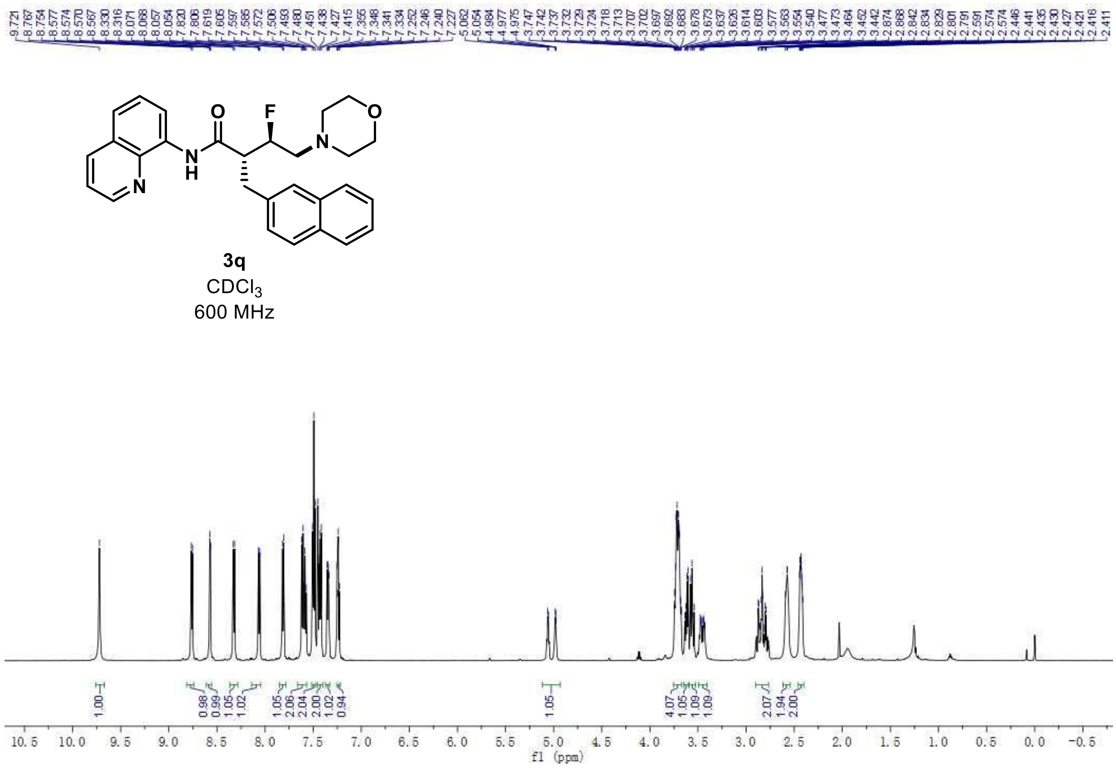

Figure S57. ^{1}H NMR Spectra of **3q**.

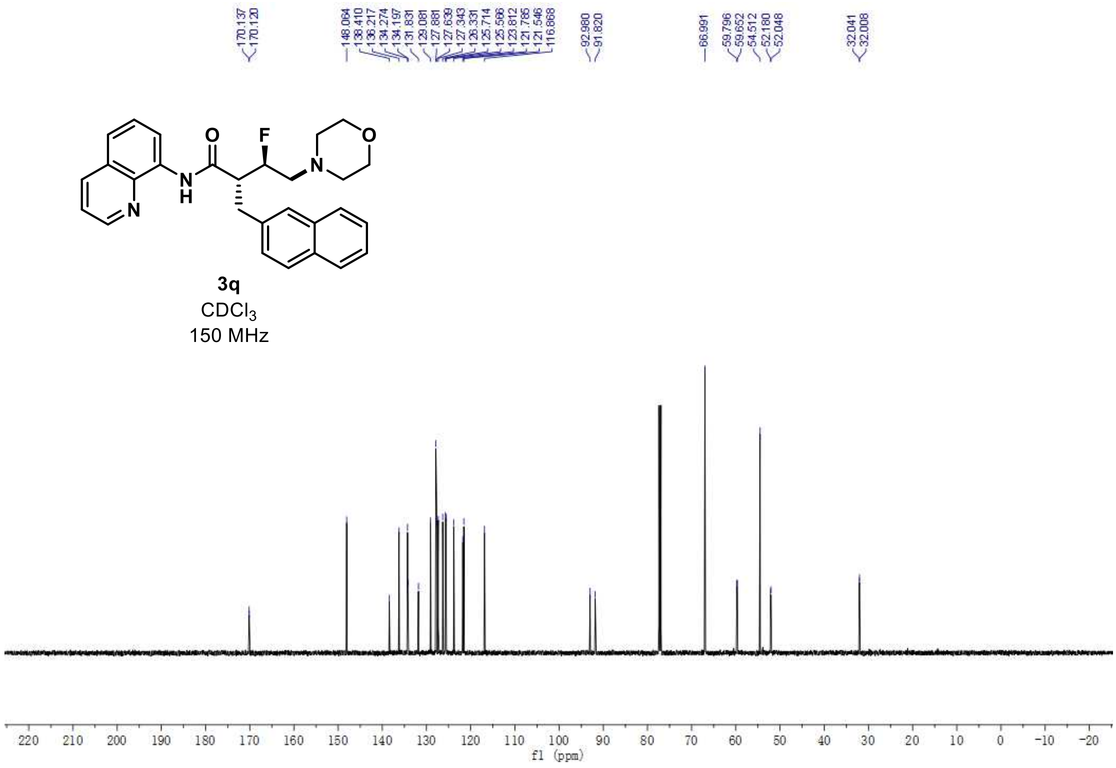

Figure S58. ^{13}C NMR Spectra of **3q**.

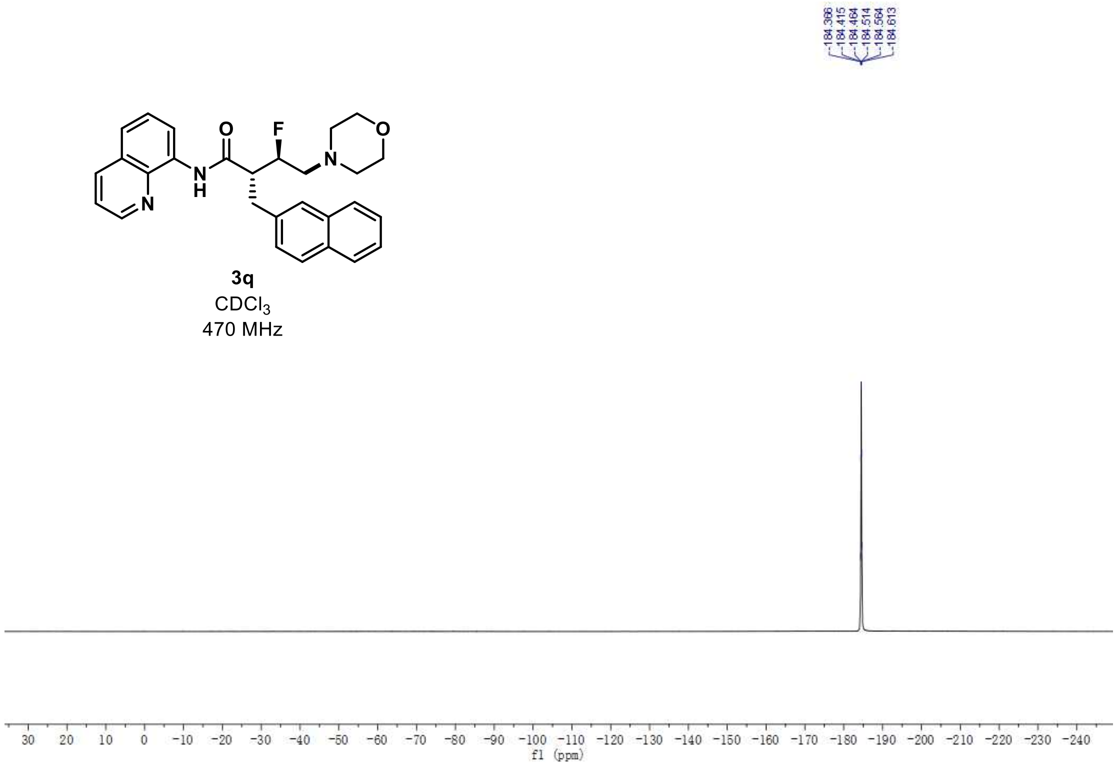

Figure S59. ^{19}F NMR Spectra of **3q**.

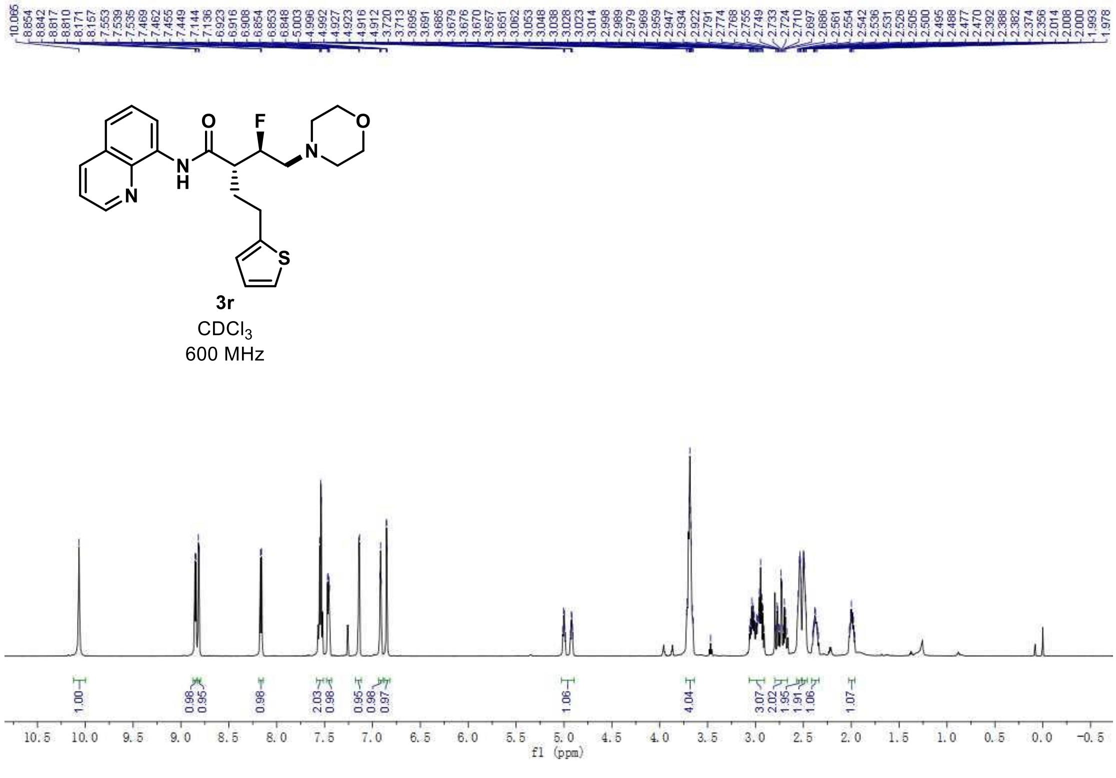

Figure S60. ^{1}H NMR Spectra of **3r**.

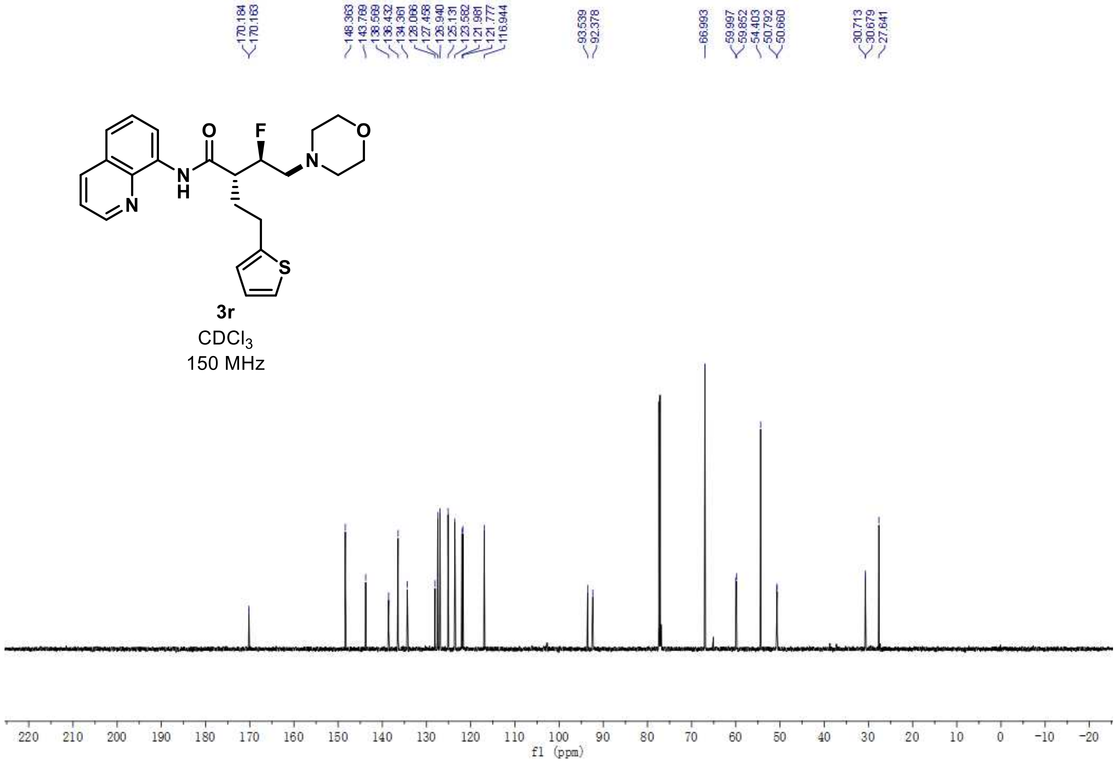

Figure S61. ^{13}C NMR Spectra of **3r**.

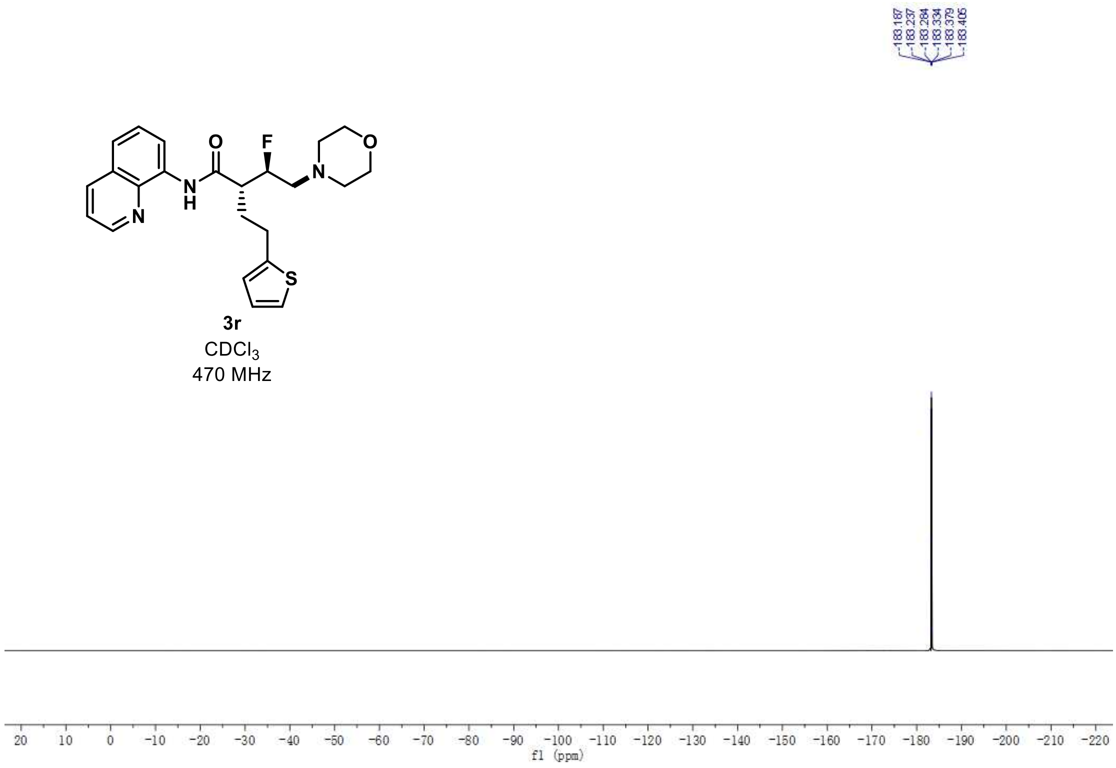

Figure S62. ^{19}F NMR Spectra of **3r**.

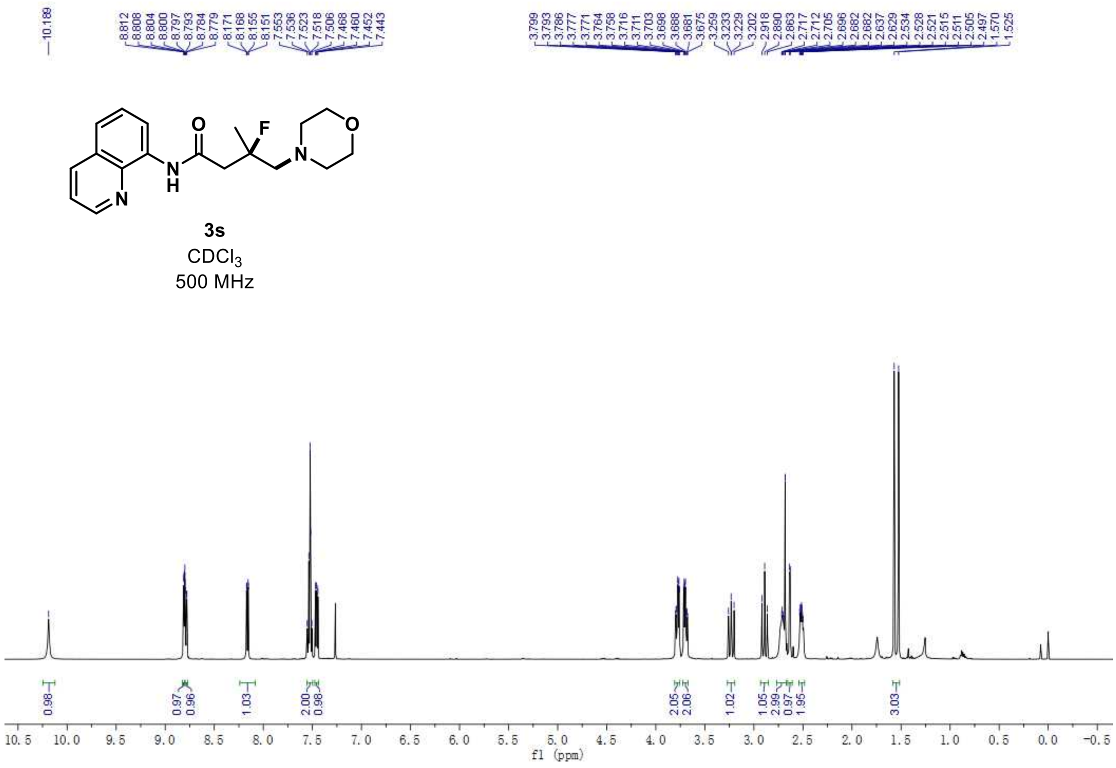

Figure S63. ^{1}H NMR Spectra of **3s**.

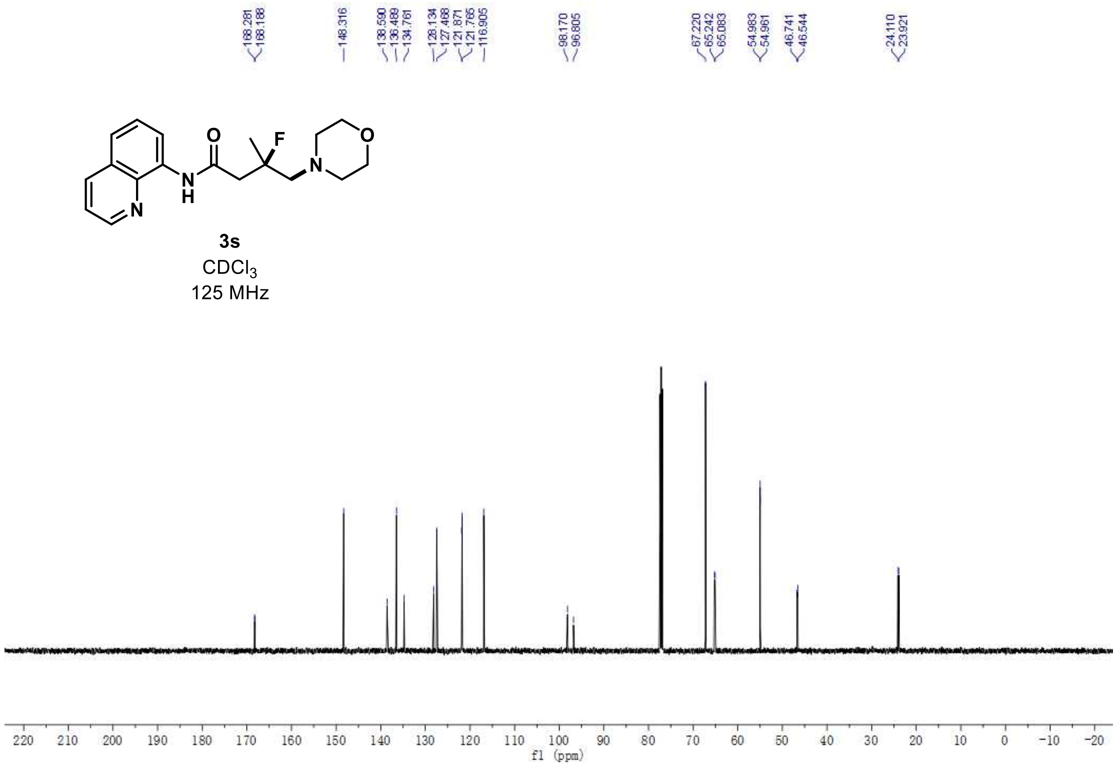

Figure S64. ^{13}C NMR Spectra of **3s**.

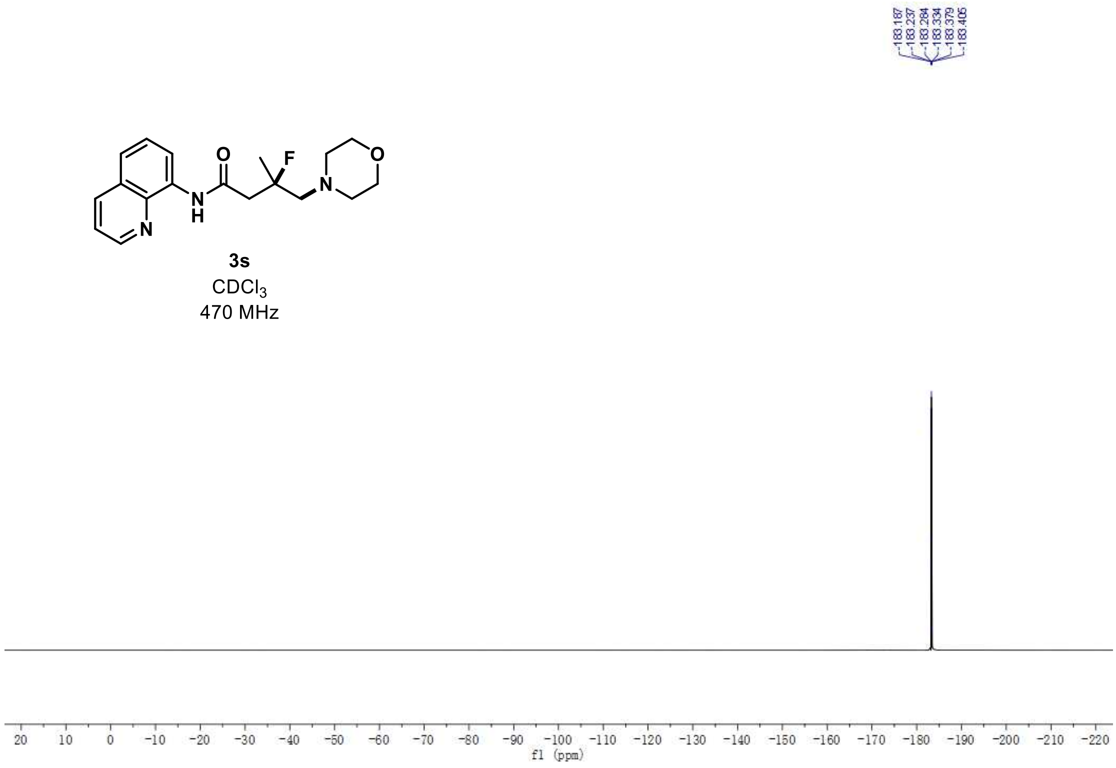

Figure S65. ^{19}F NMR Spectra of **3s**.

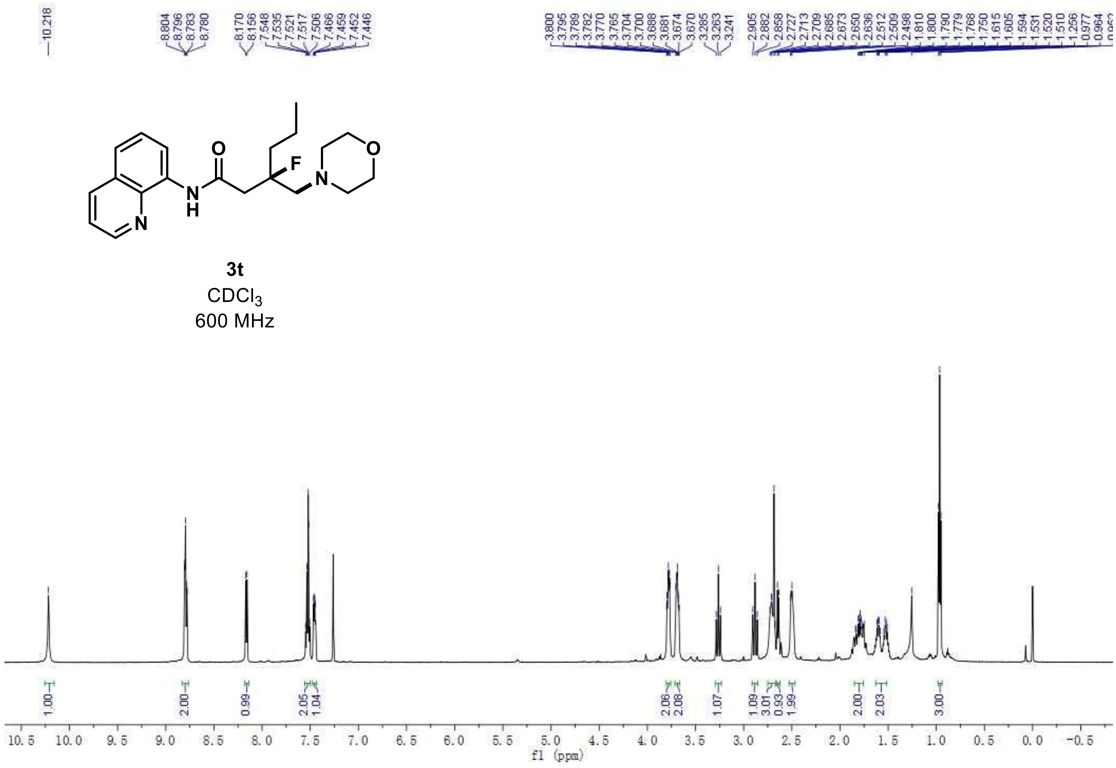

Figure S66. ^{1}H NMR Spectra of **3t**.

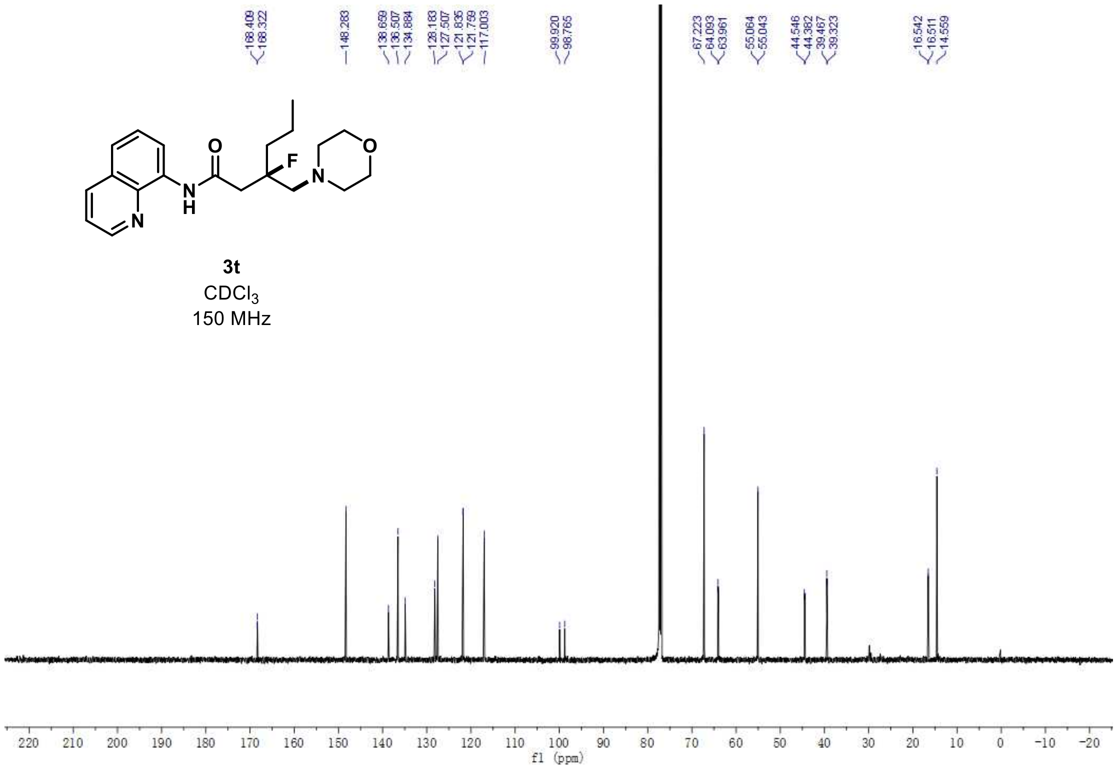

Figure S67. ^{13}C NMR Spectra of **3t**.

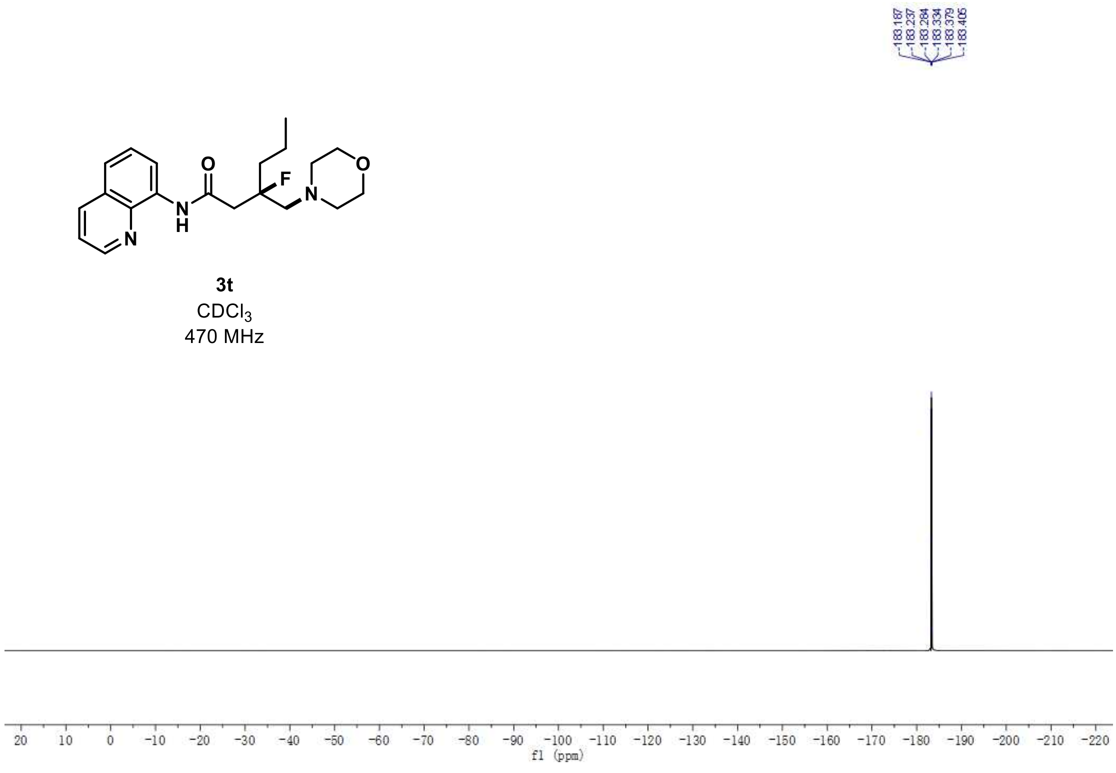

Figure S68. ^{19}F NMR Spectra of **3t**.

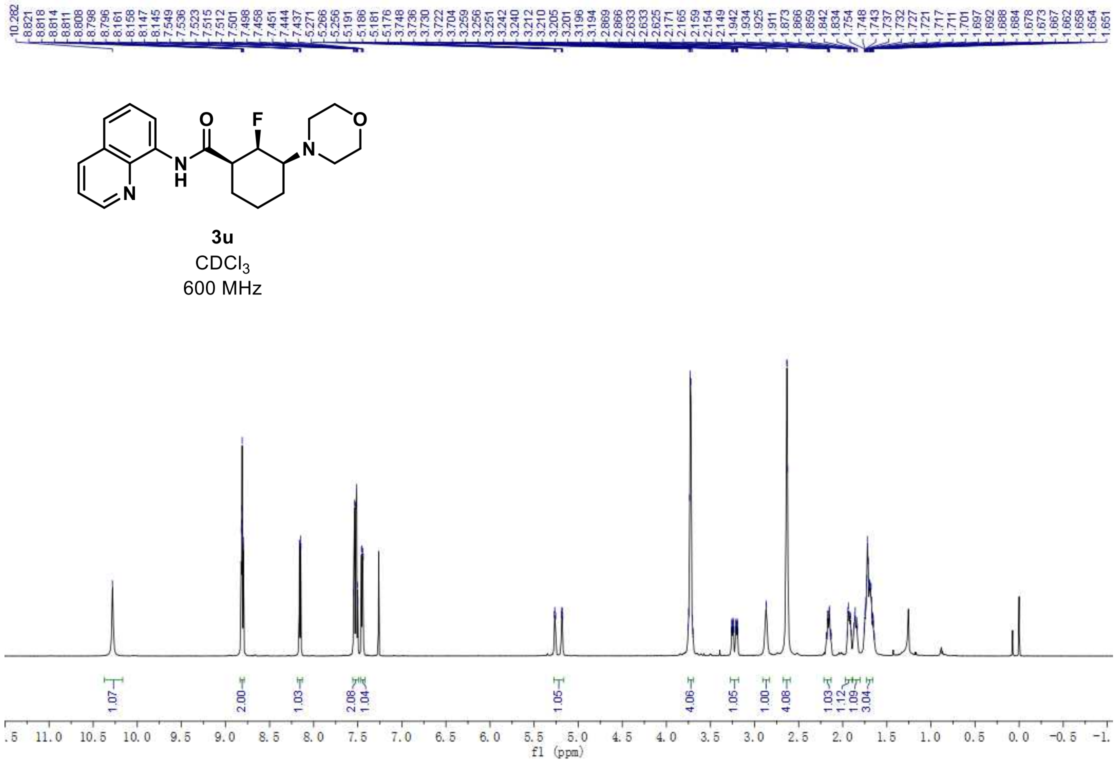

Figure S69. ^{1}H NMR Spectra of **3u**.

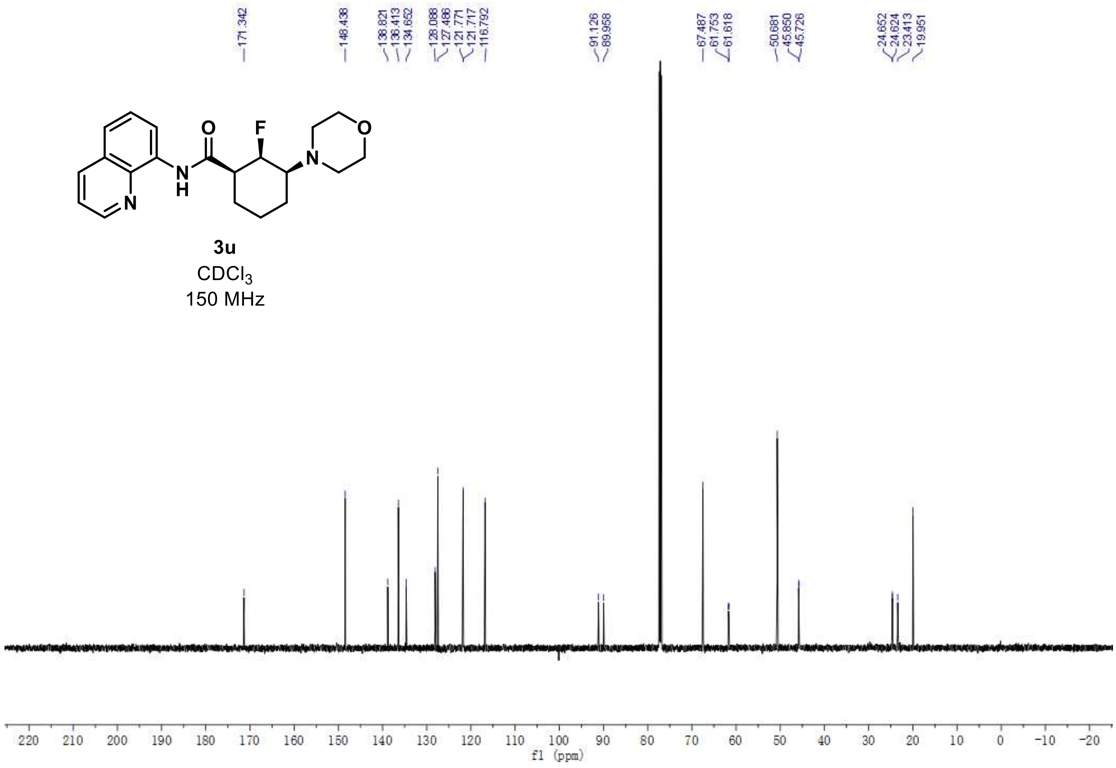

Figure S70. ^{13}C NMR Spectra of **3u**.

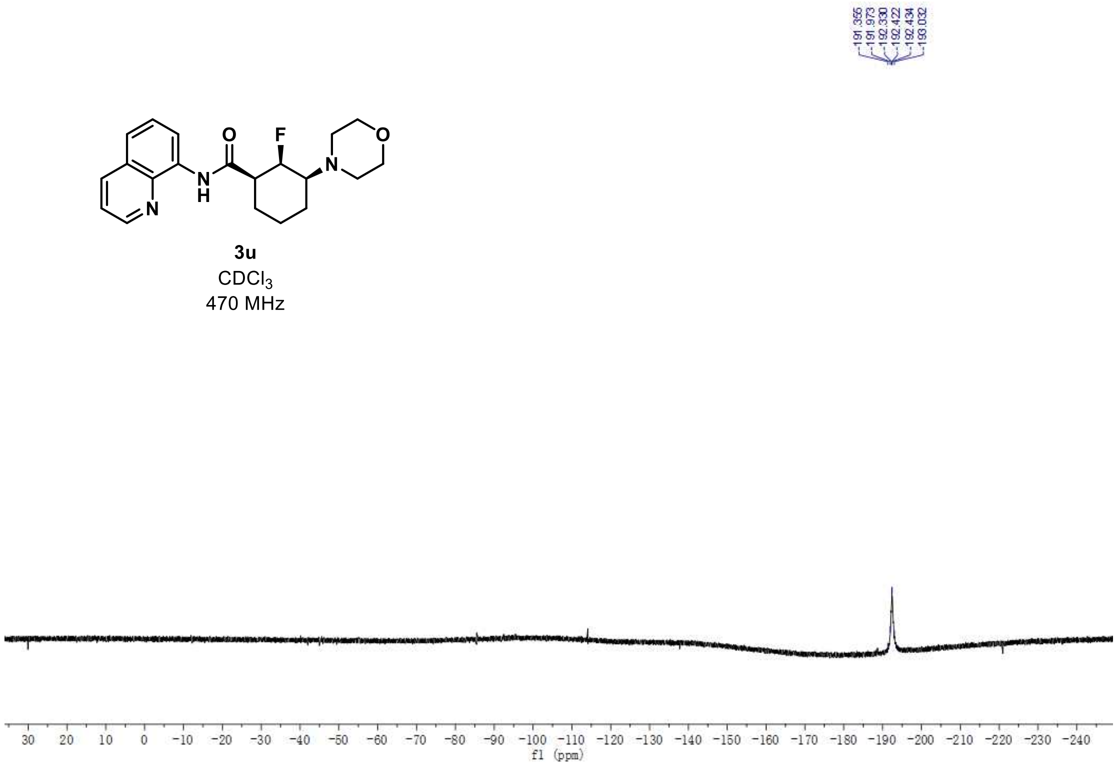

Figure S71. ^{19}F NMR Spectra of **3u**.

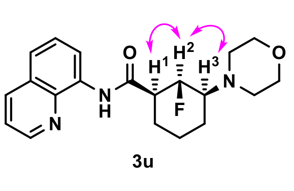

3u

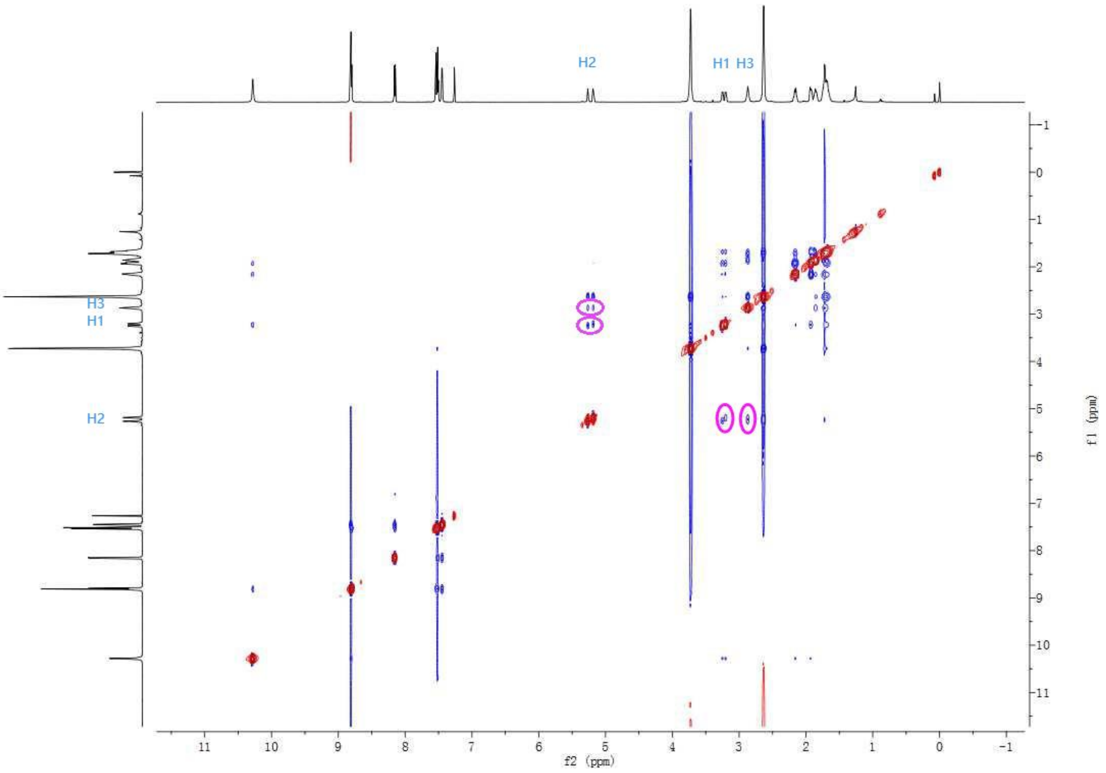

Figure S72. Noesy Spectra of **3u**.

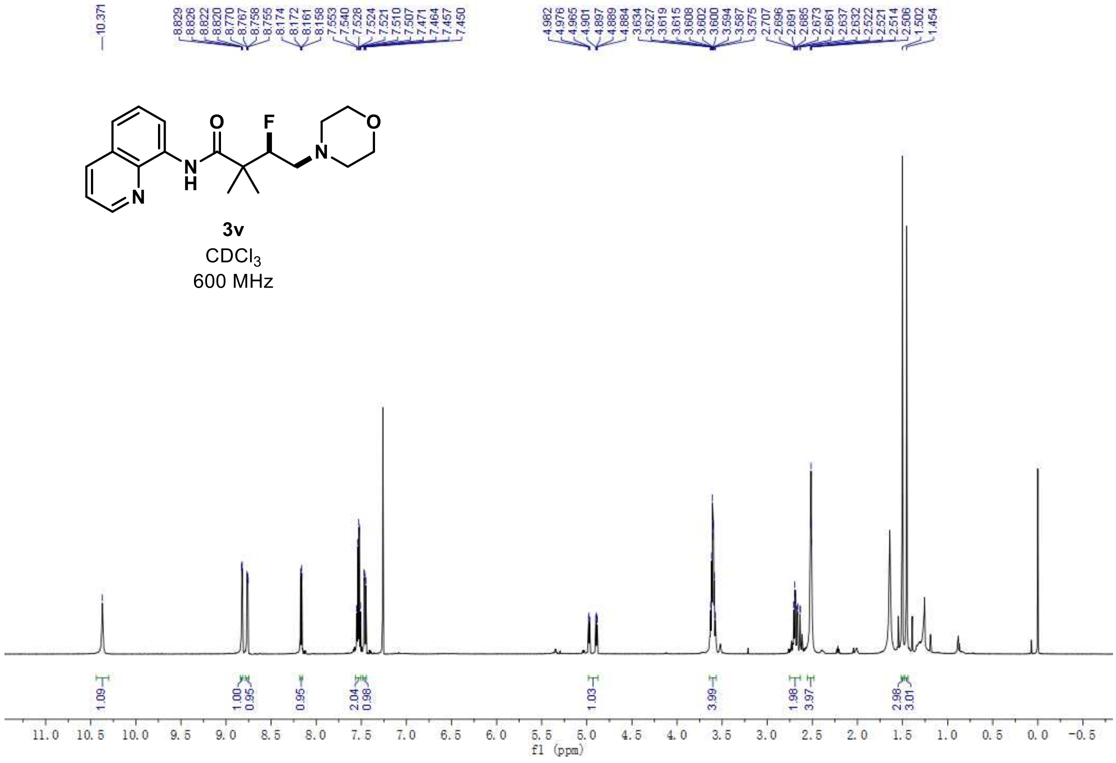

Figure S73. ^{1}H NMR Spectra of **3v**.

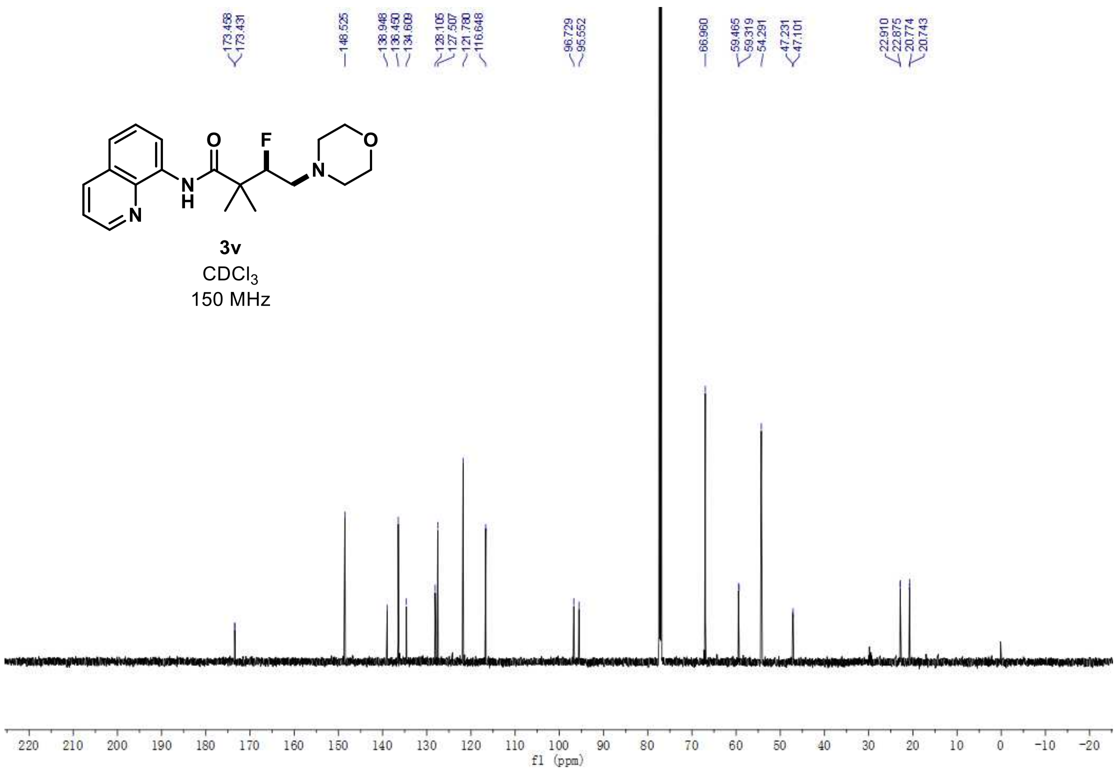

Figure S74. ^{13}C NMR Spectra of **3v**.

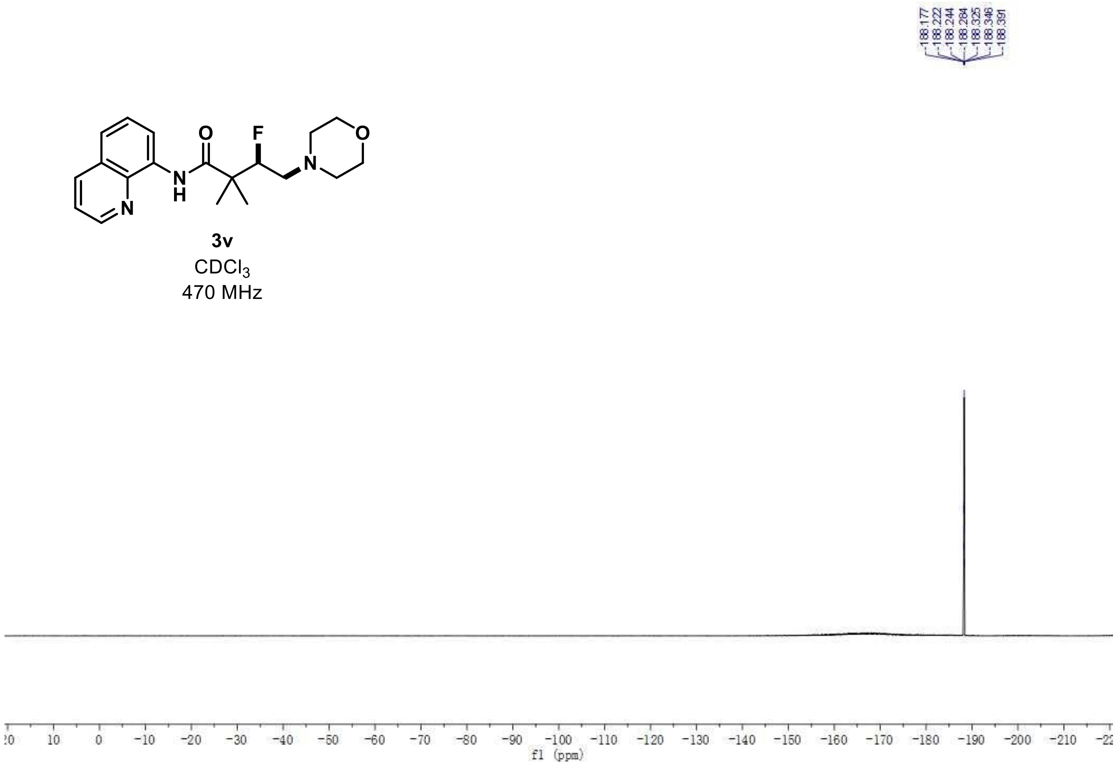

Figure S75. ^{19}F NMR Spectra of **3v**.

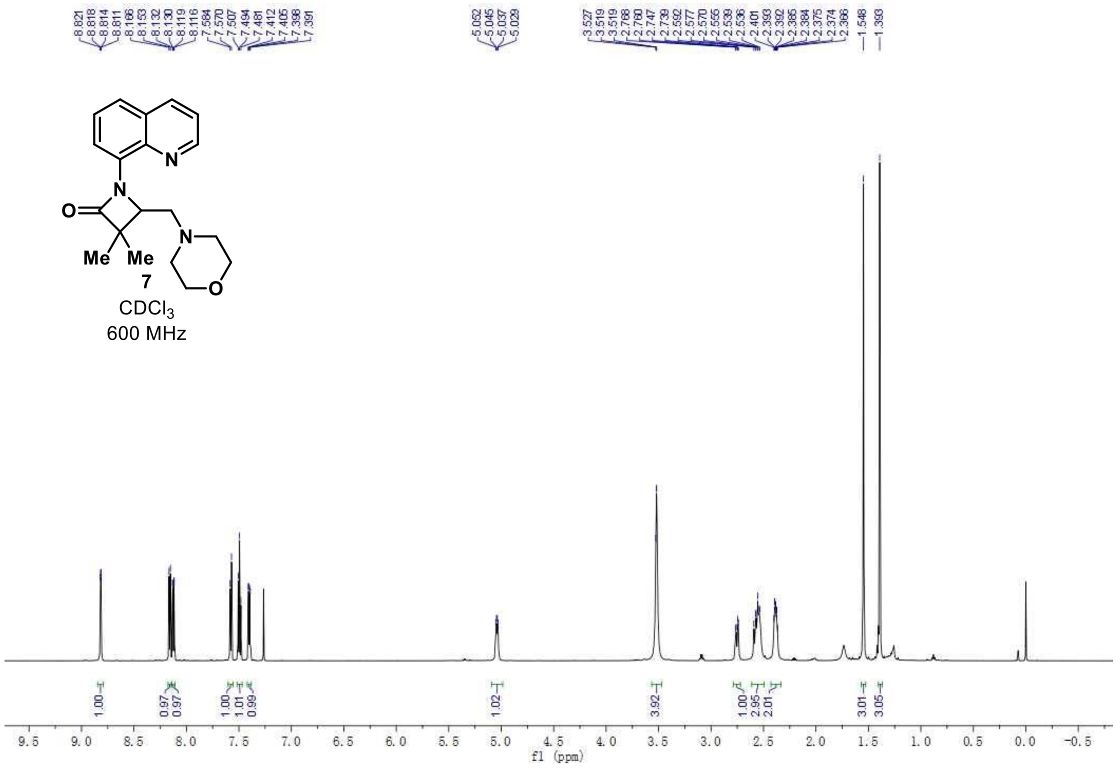

Figure S76. ^{1}H NMR Spectra of **7**.

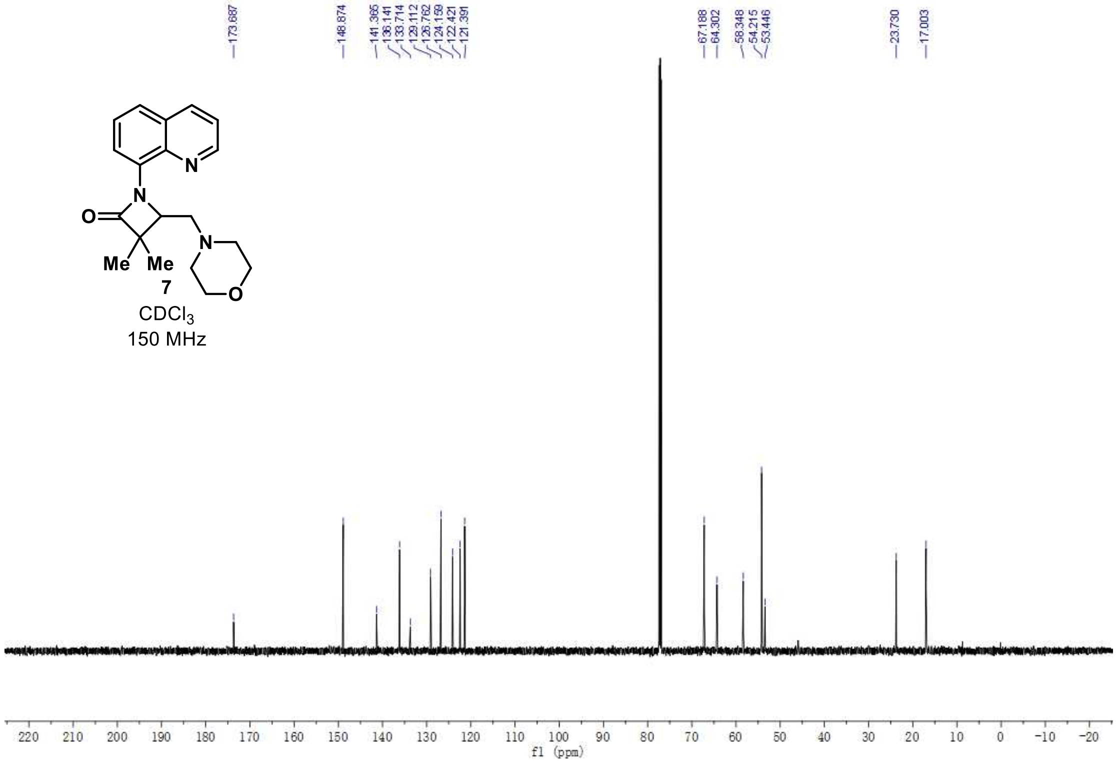

Figure S77. ^{13}C NMR Spectra of **7**.

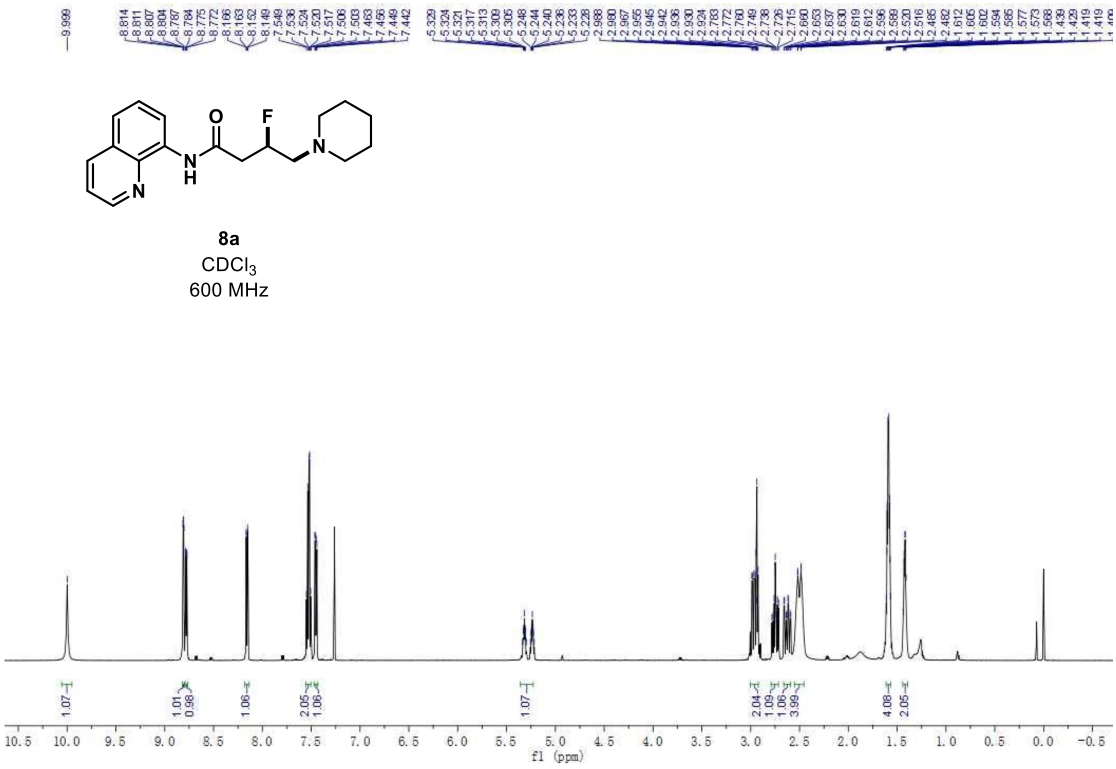

Figure S78. ^{1}H NMR Spectra of **8a**.

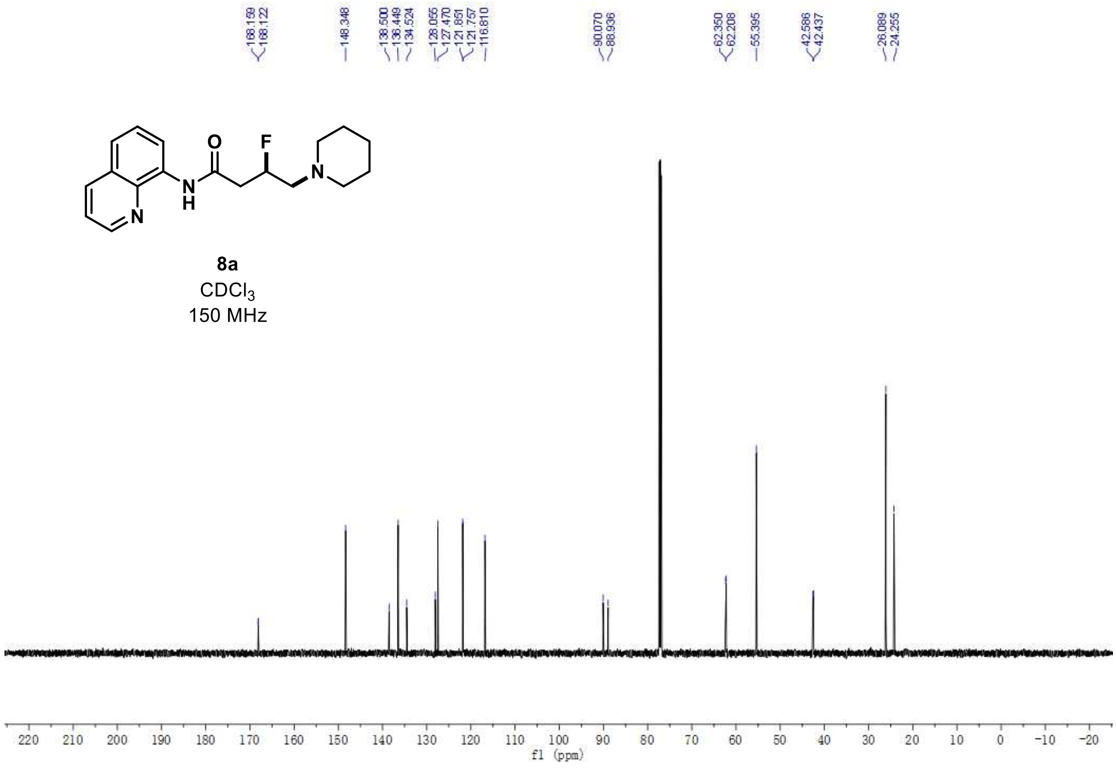

Figure S79. ^{13}C NMR Spectra of **8a**.

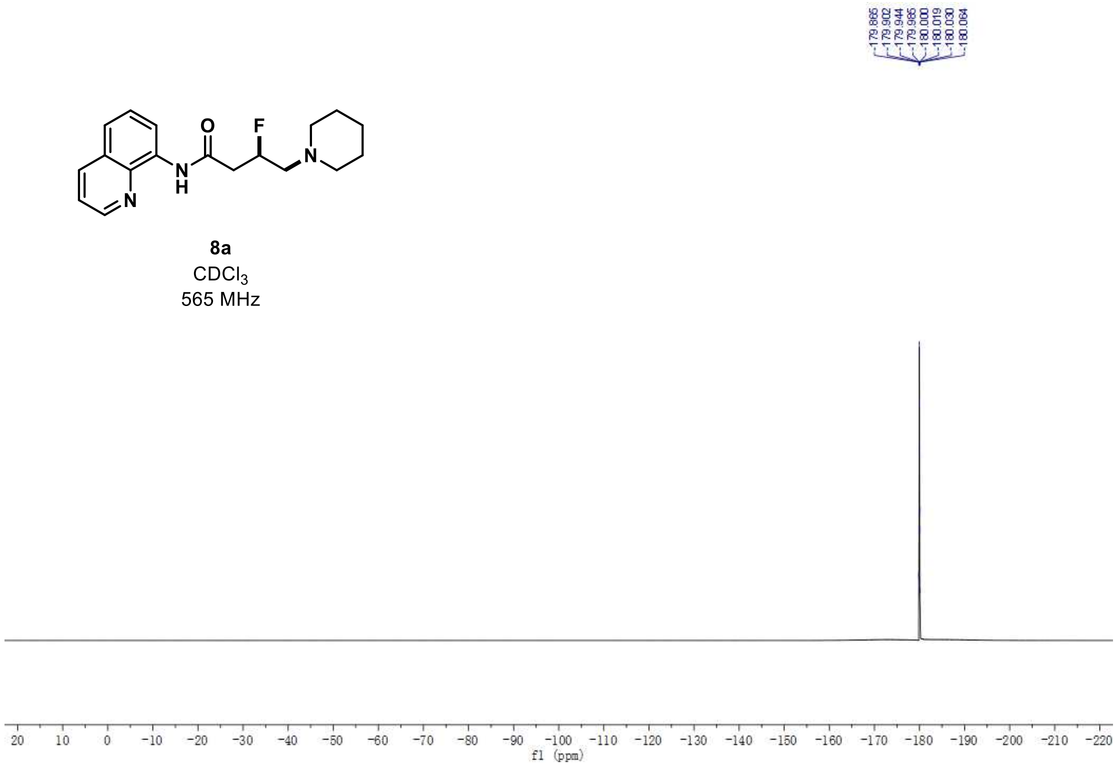

Figure S80. ^{19}F NMR Spectra of **8a**.

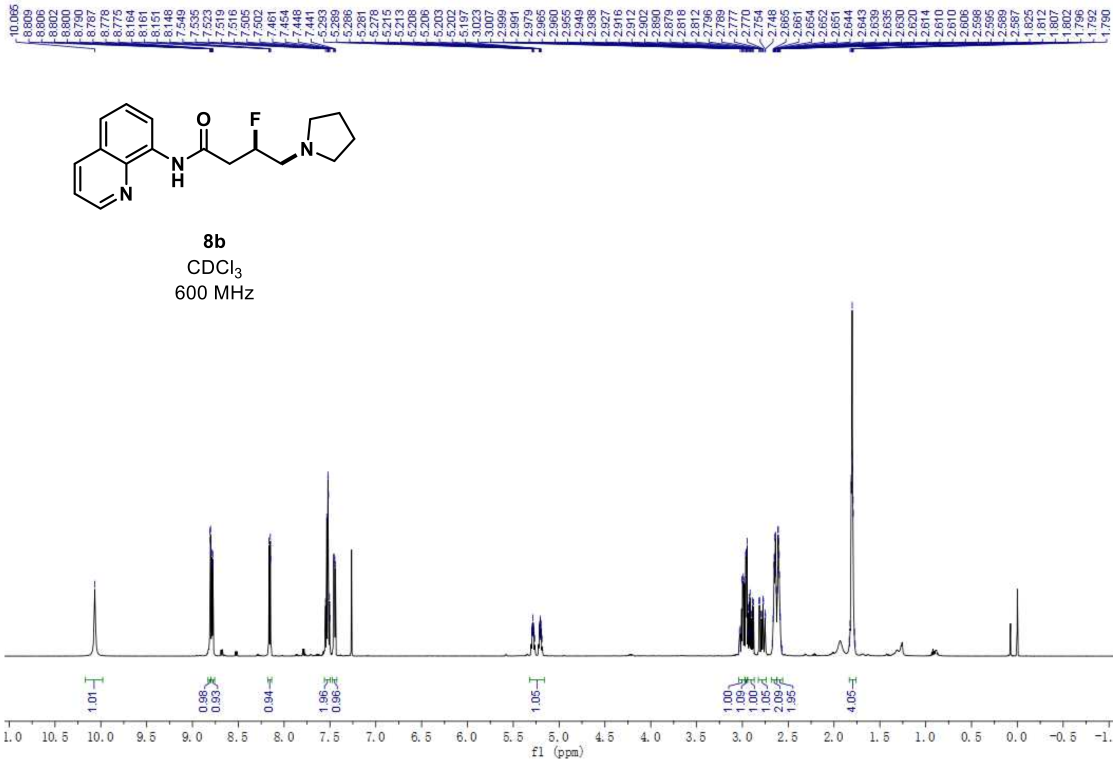

Figure S81. ^{1}H NMR Spectra of **8b**.

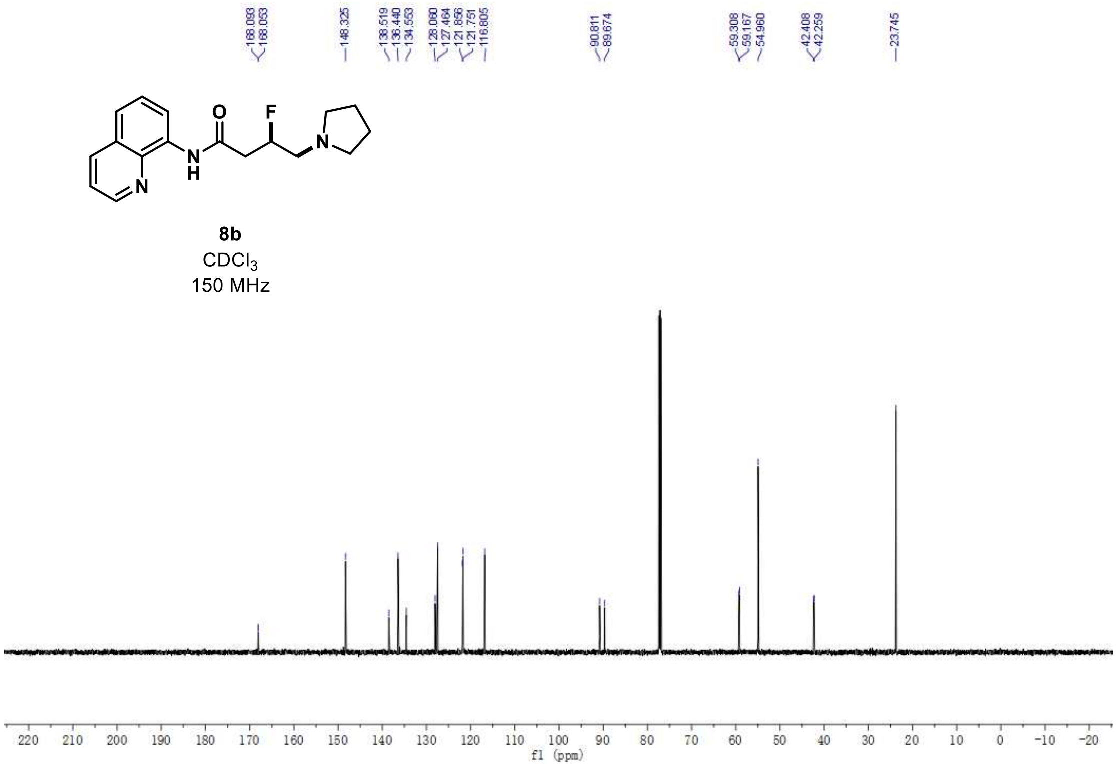

Figure S82. ^{13}C NMR Spectra of **8b**.

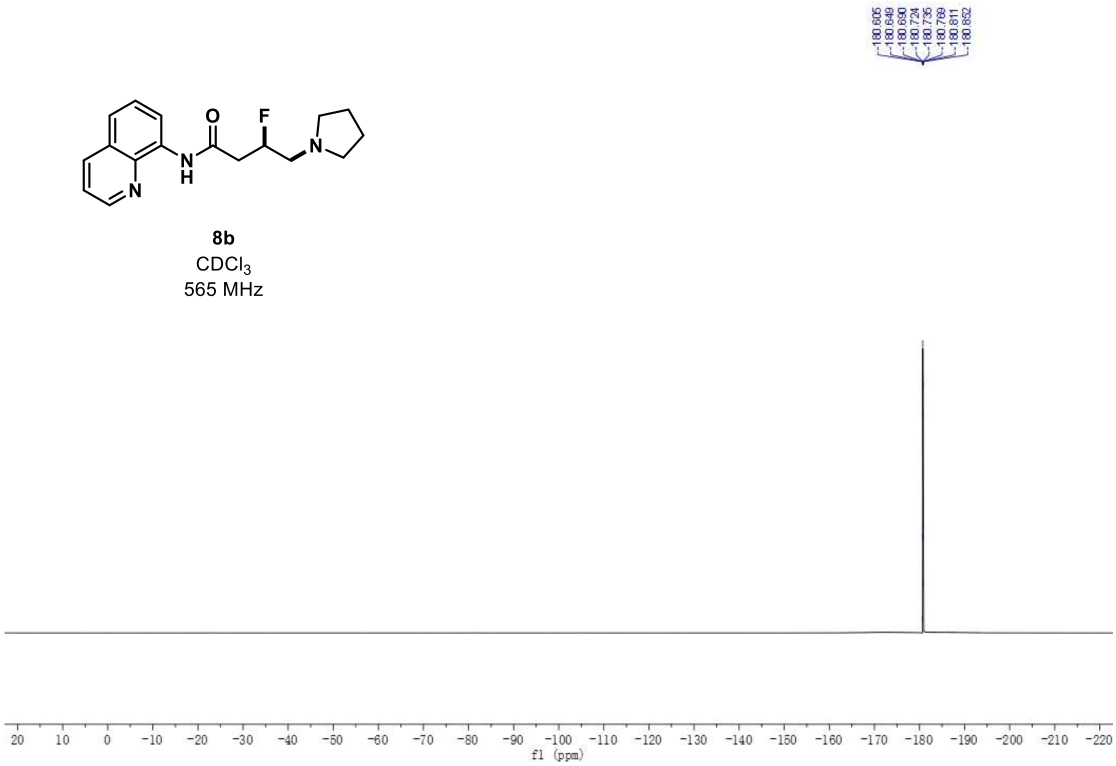

Figure S83. ^{19}F NMR Spectra of **8b**.

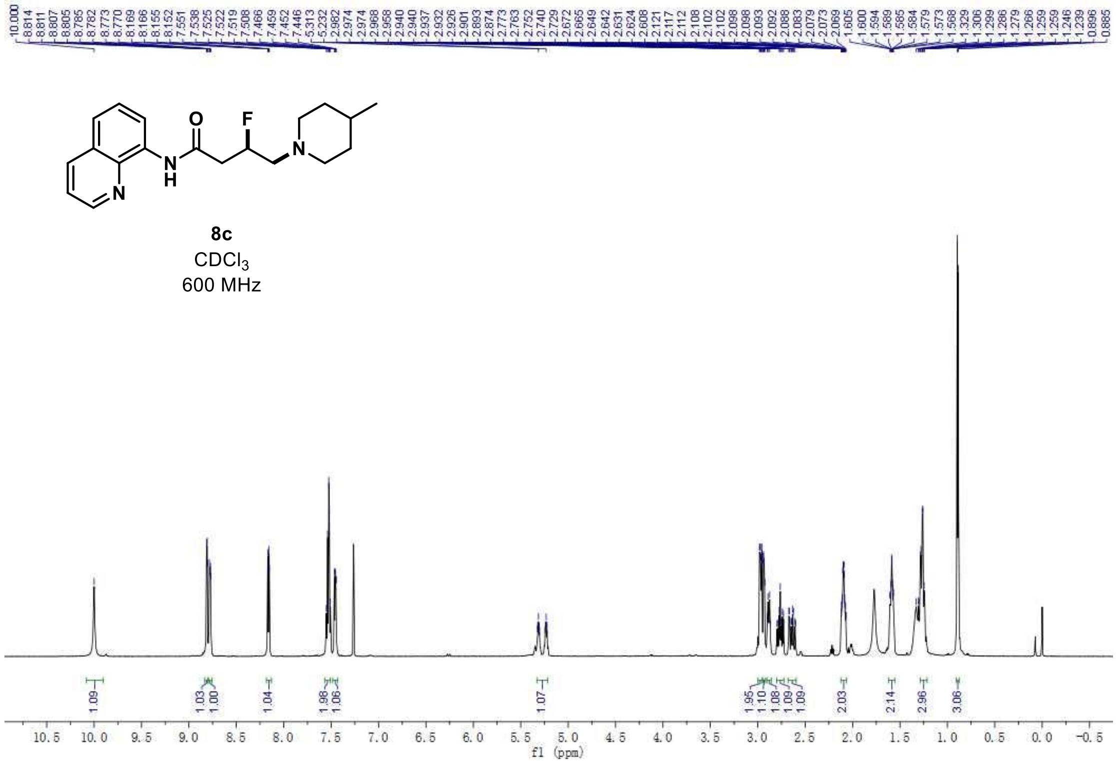

Figure S84. ^{1}H NMR Spectra of **8c**.

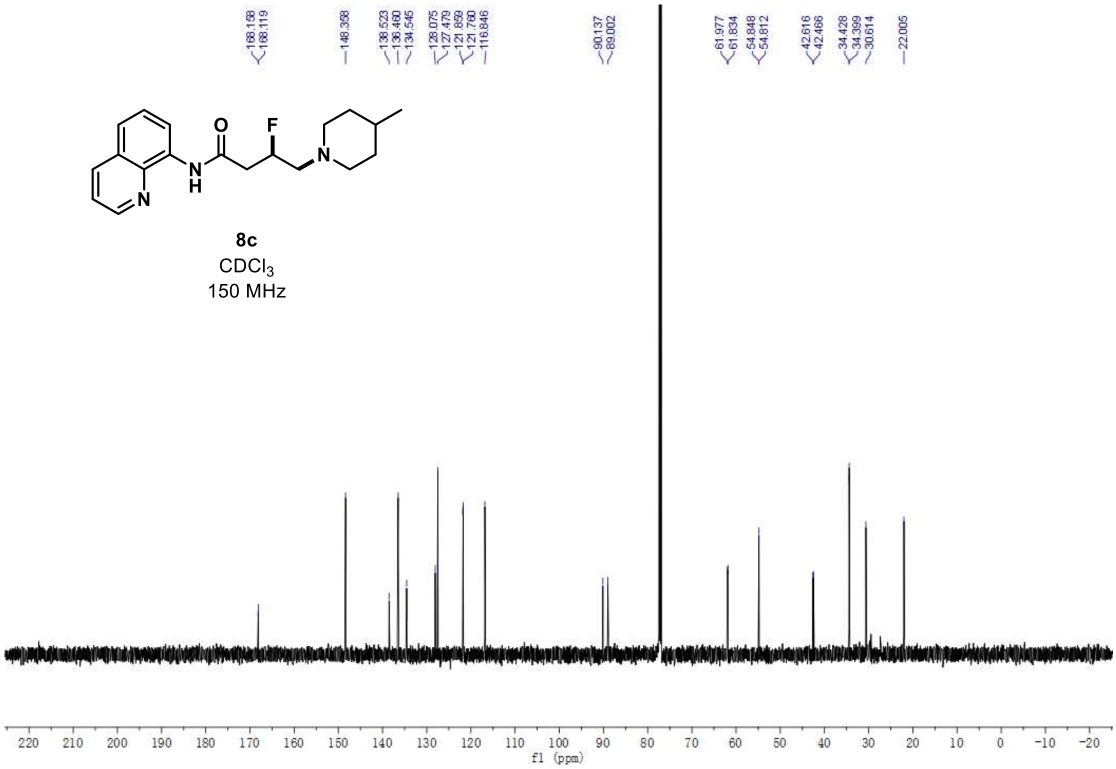

Figure S85. ^{13}C NMR Spectra of **8c**.

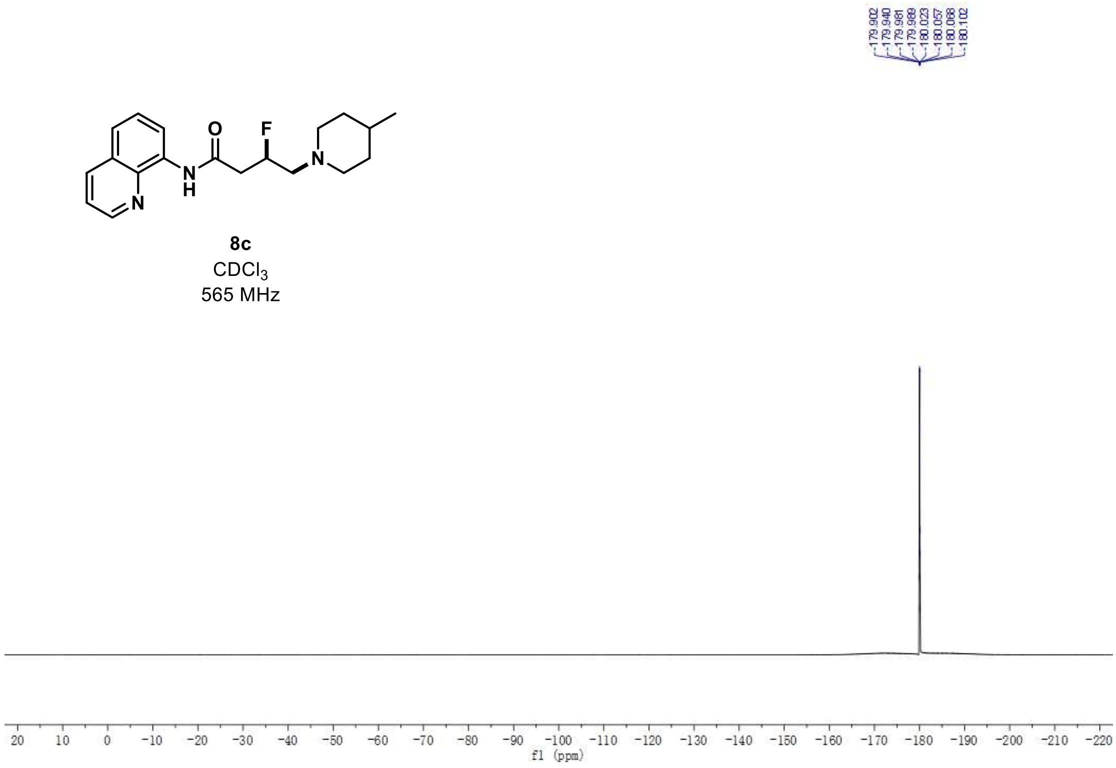

Figure S86. ^{19}F NMR Spectra of **8c**.

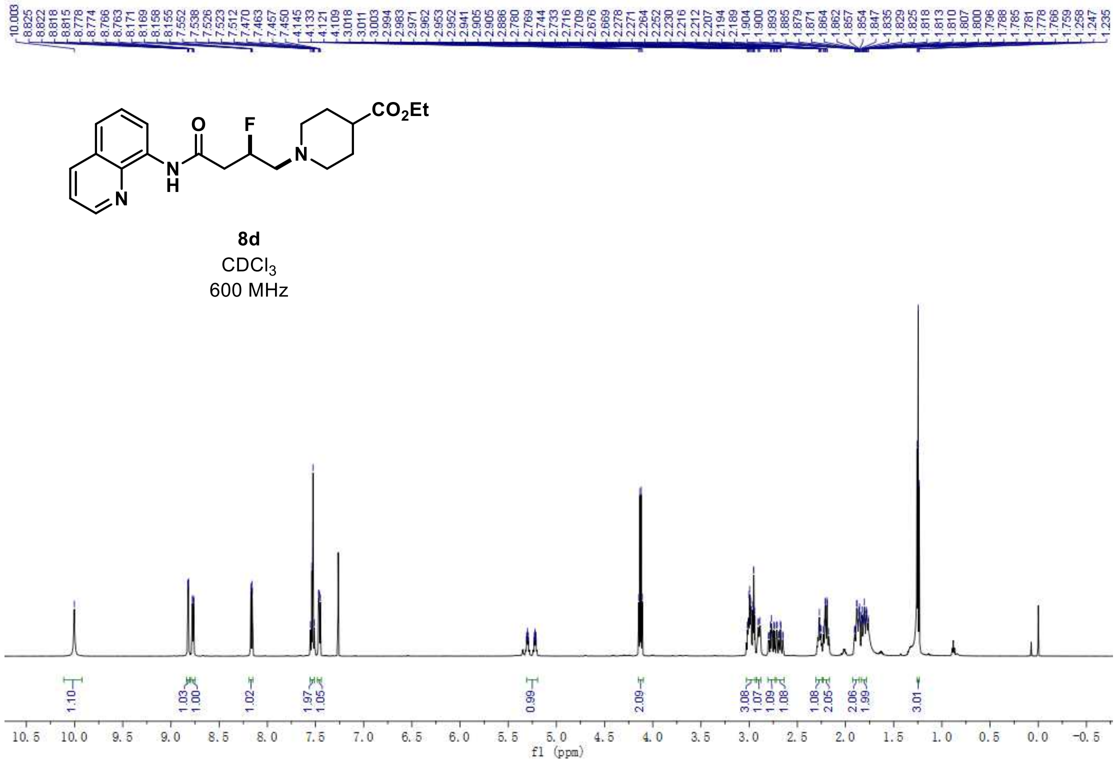

Figure S87. ^{1}H NMR Spectra of **8d**.

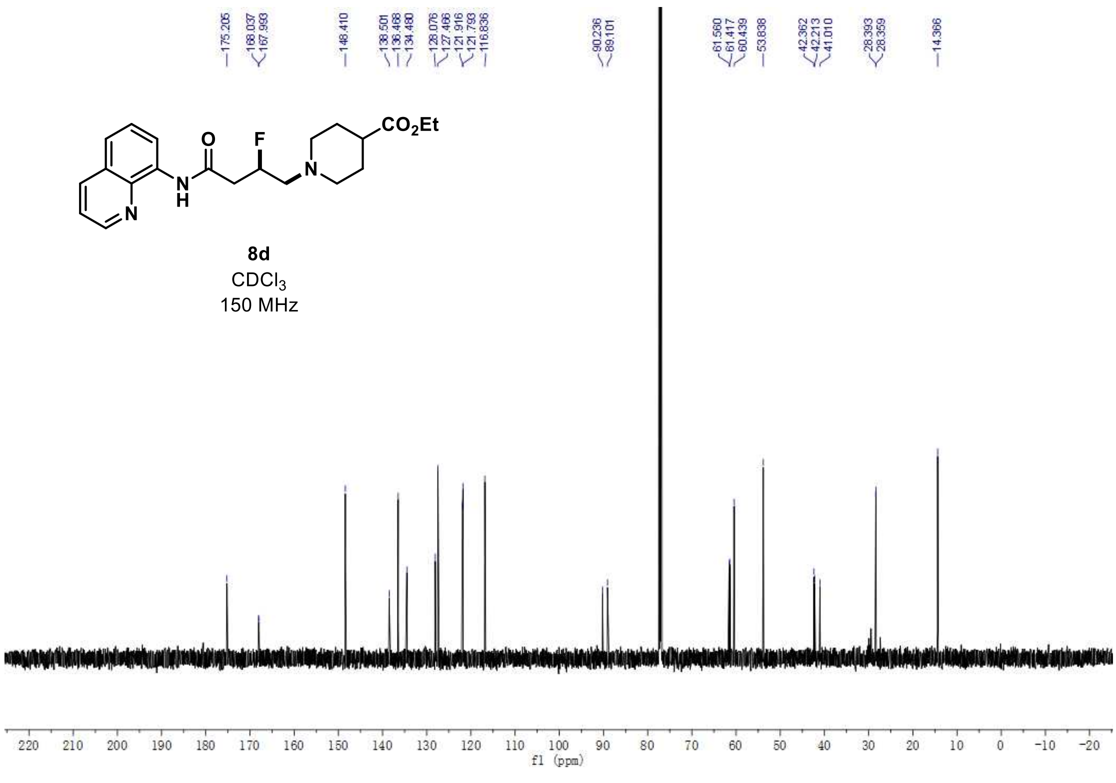

Figure S88. ^{13}C NMR Spectra of **8d**.

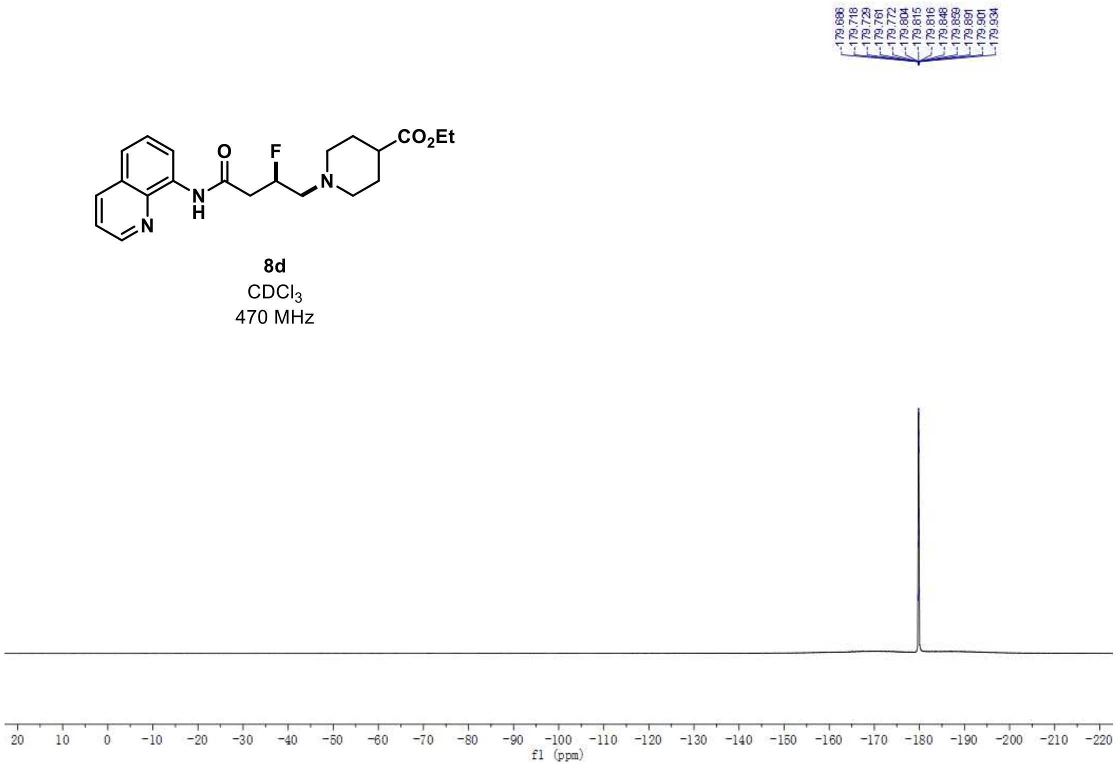

Figure S89. ^{19}F NMR Spectra of **8d**.

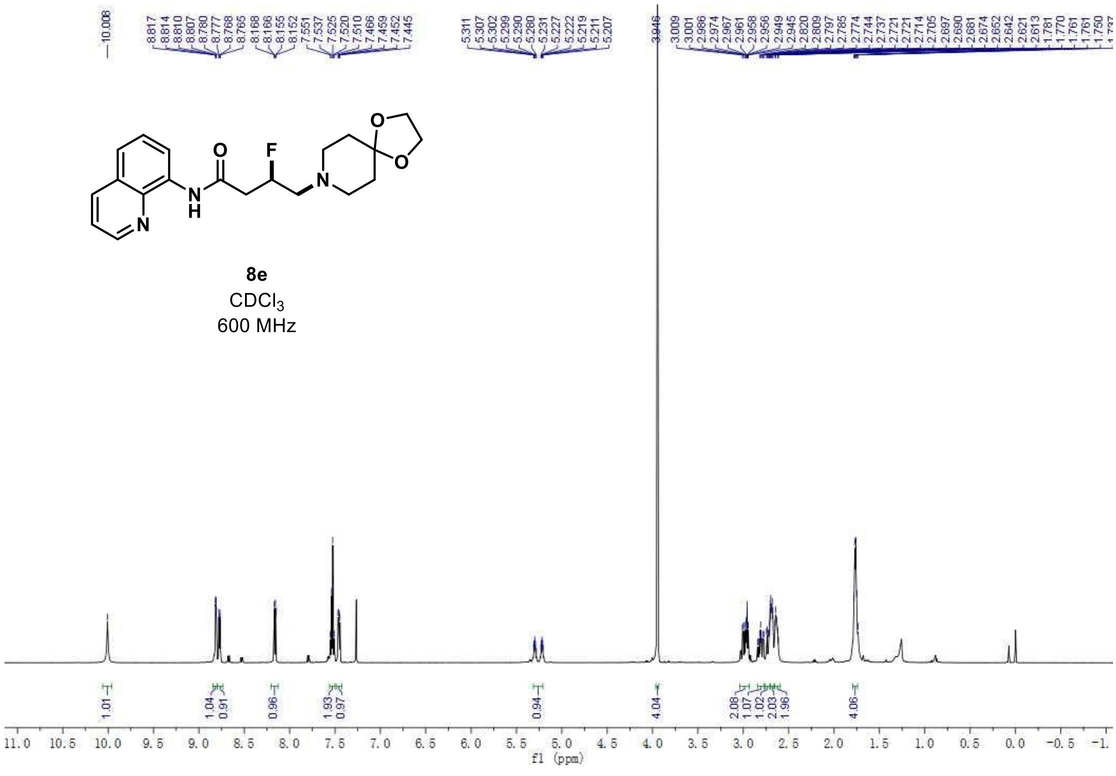

Figure S90. ^{1}H NMR Spectra of **8e**.

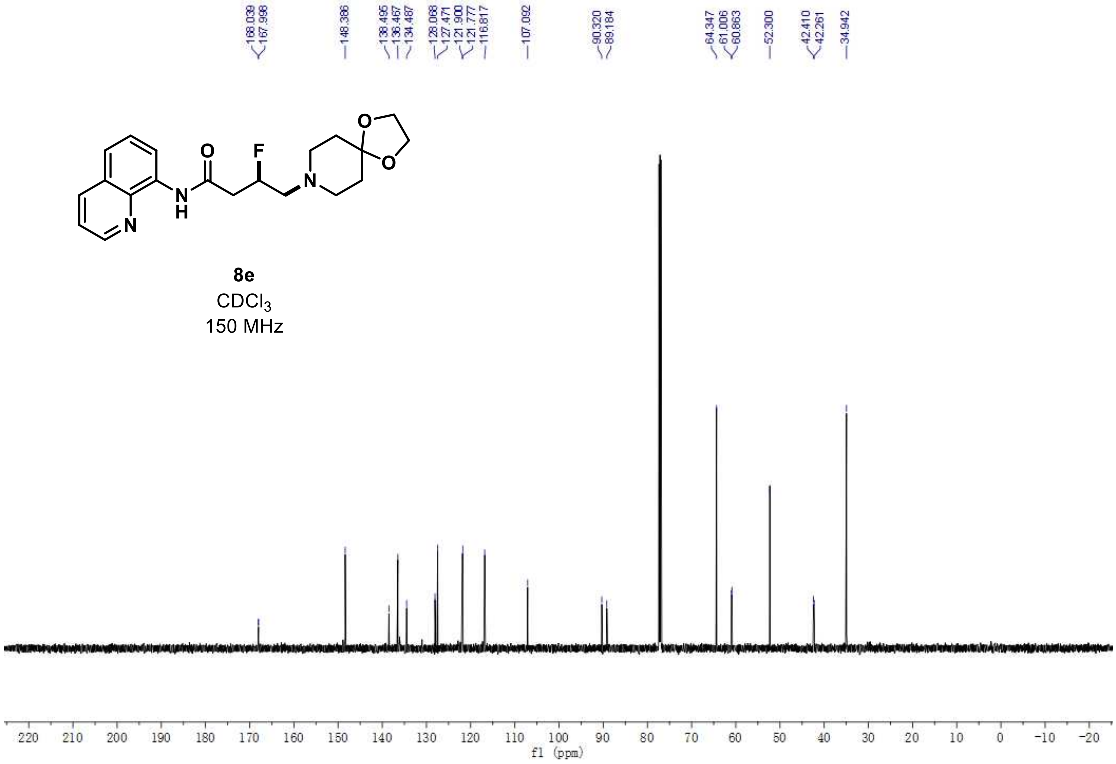

Figure S91. ^{13}C NMR Spectra of **8e**.

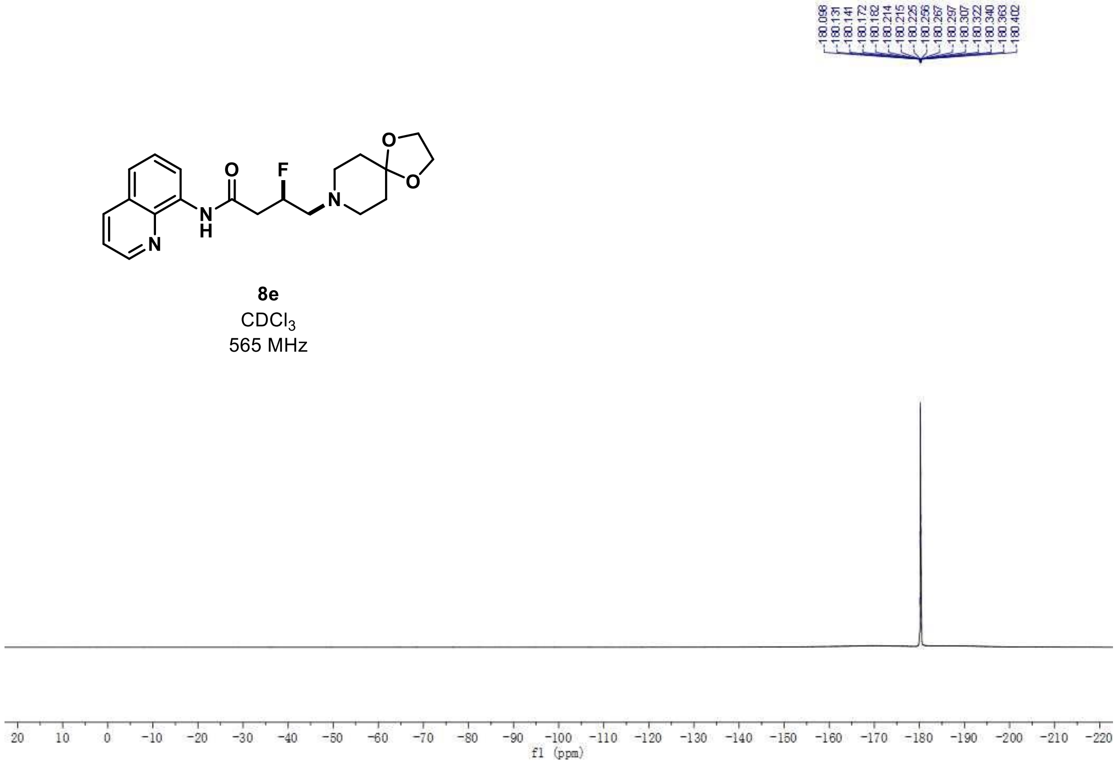

Figure S92. ^{19}F NMR Spectra of **8e**.

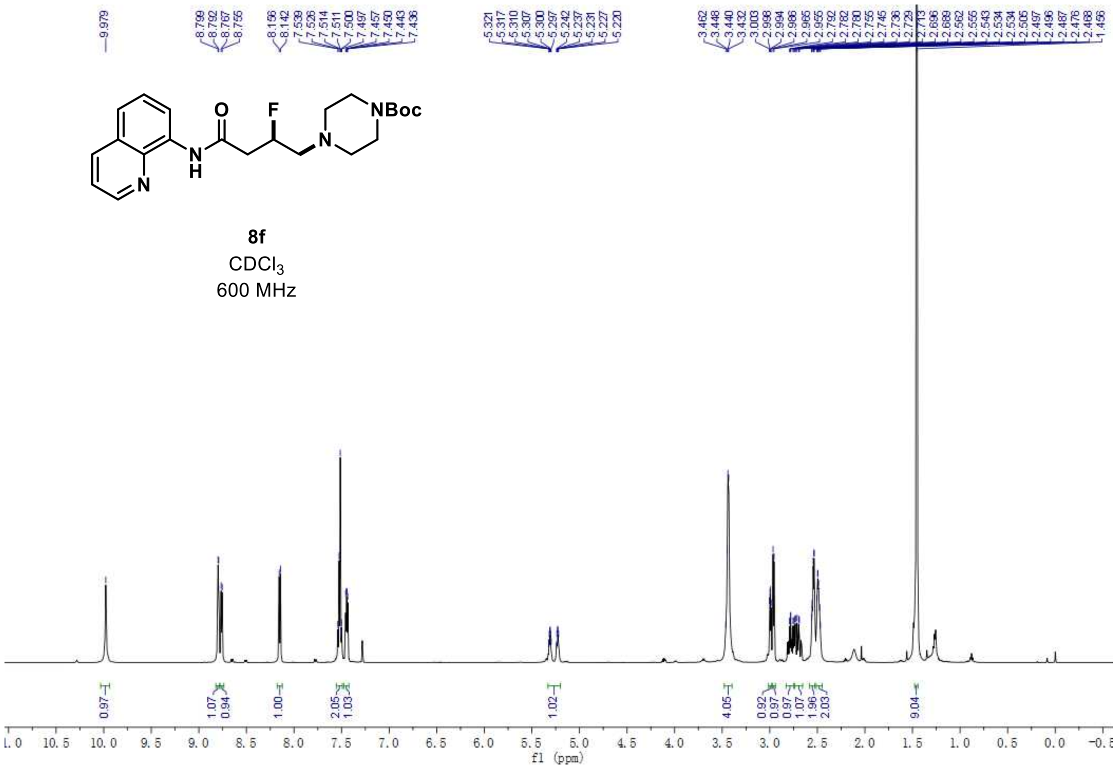

Figure S93. ^{1}H NMR Spectra of **8f**.

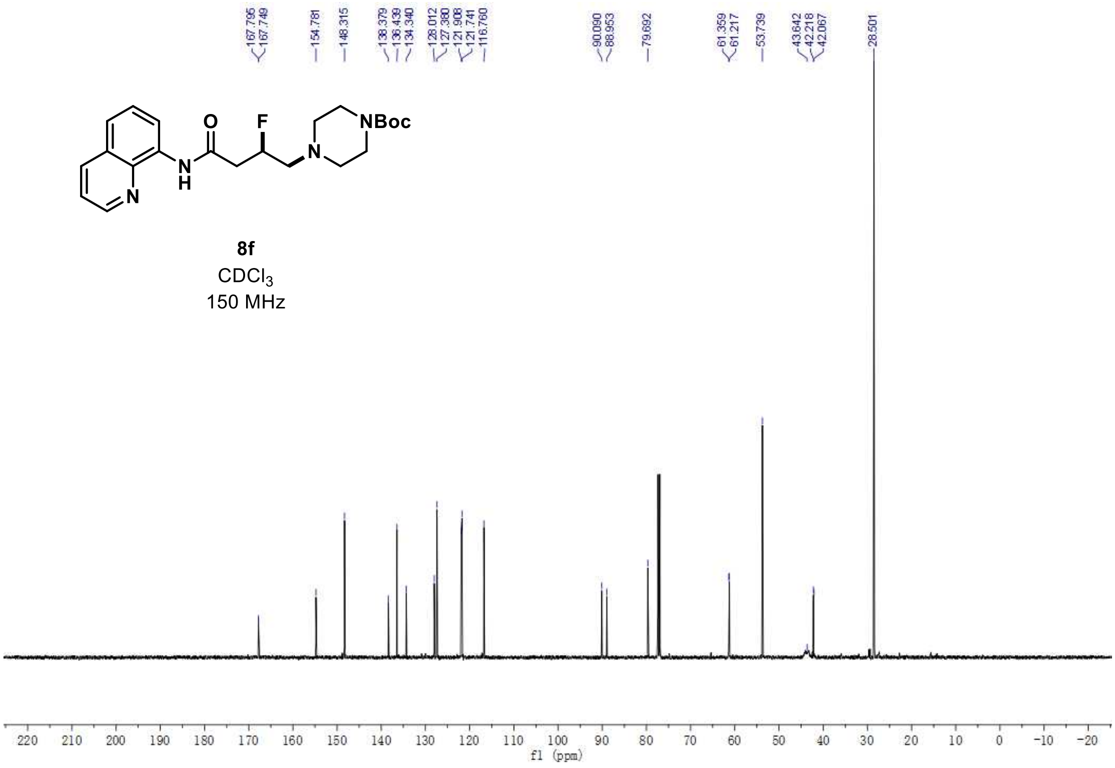

Figure S94. ^{13}C NMR Spectra of **8f**.

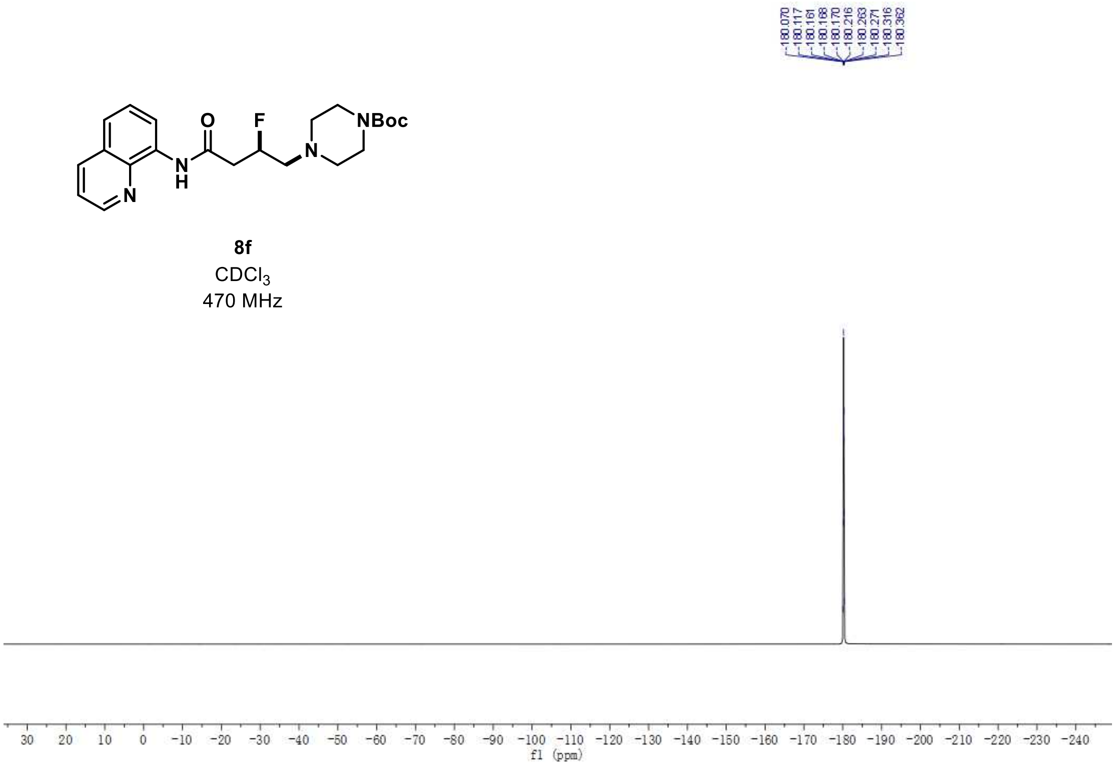

Figure S95. ^{19}F NMR Spectra of **8f**.

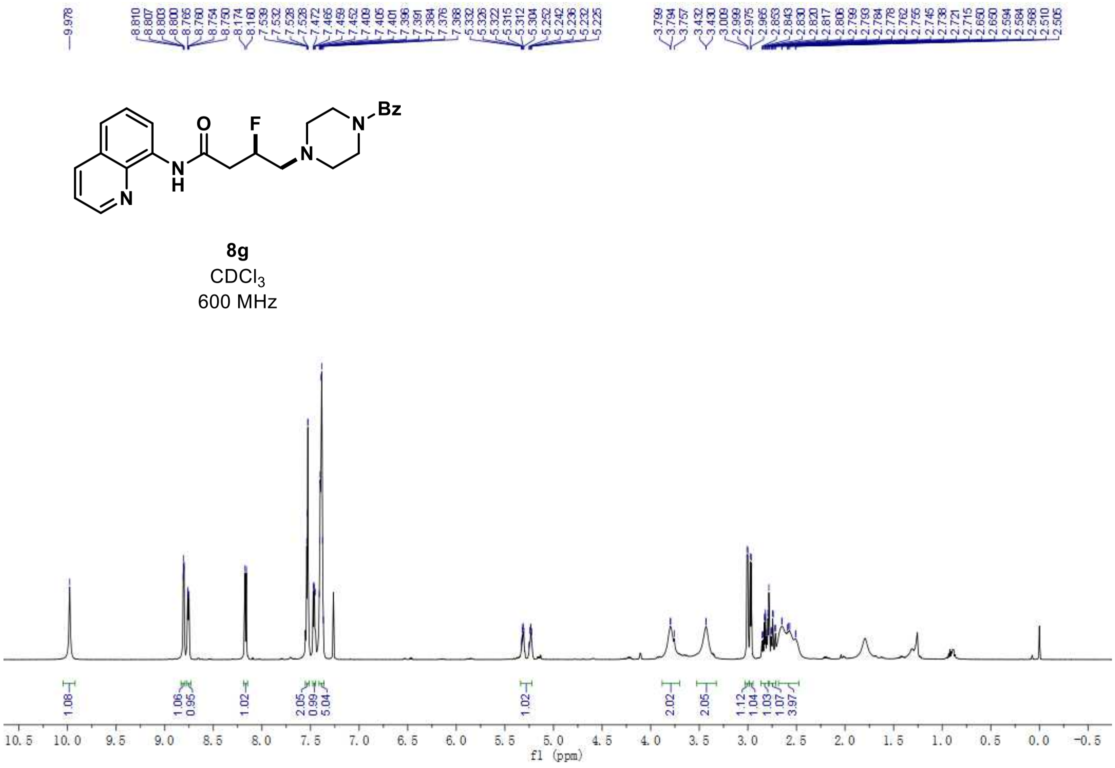

Figure S96. ^{1}H NMR Spectra of **8g**.

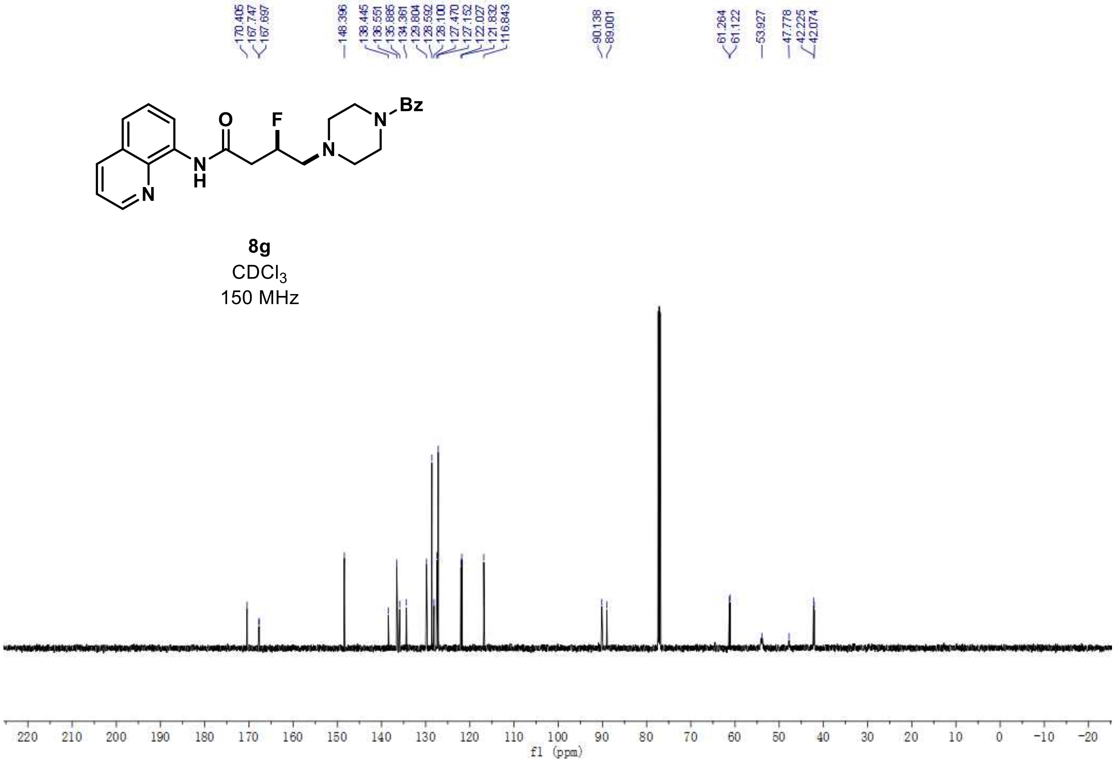

Figure S97. ^{13}C NMR Spectra of **8g**.

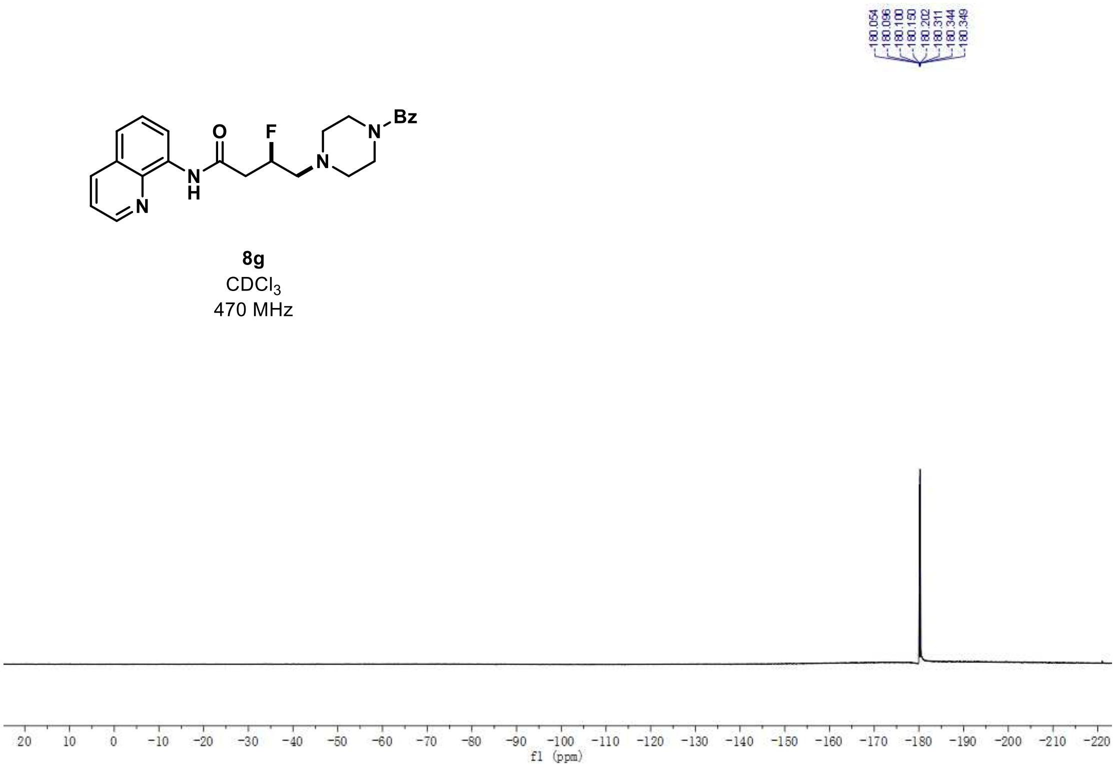

Figure S98. ^{19}F NMR Spectra of **8g**.

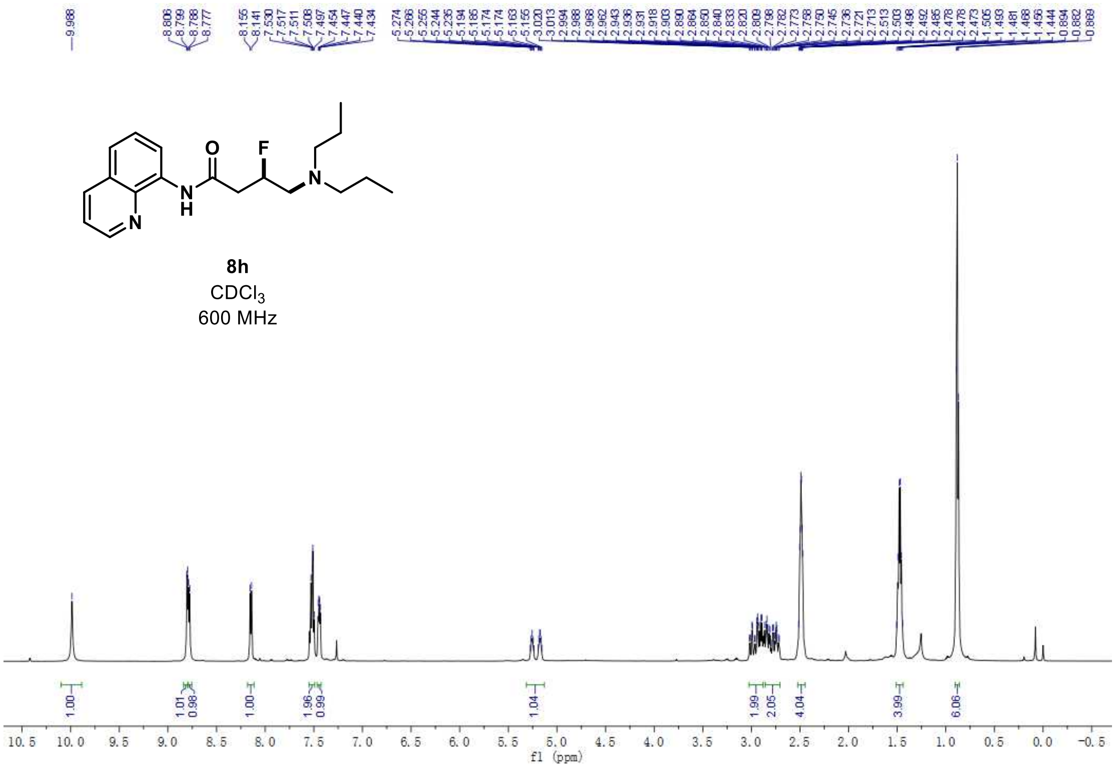

Figure S99. ^{1}H NMR Spectra of **8h**.

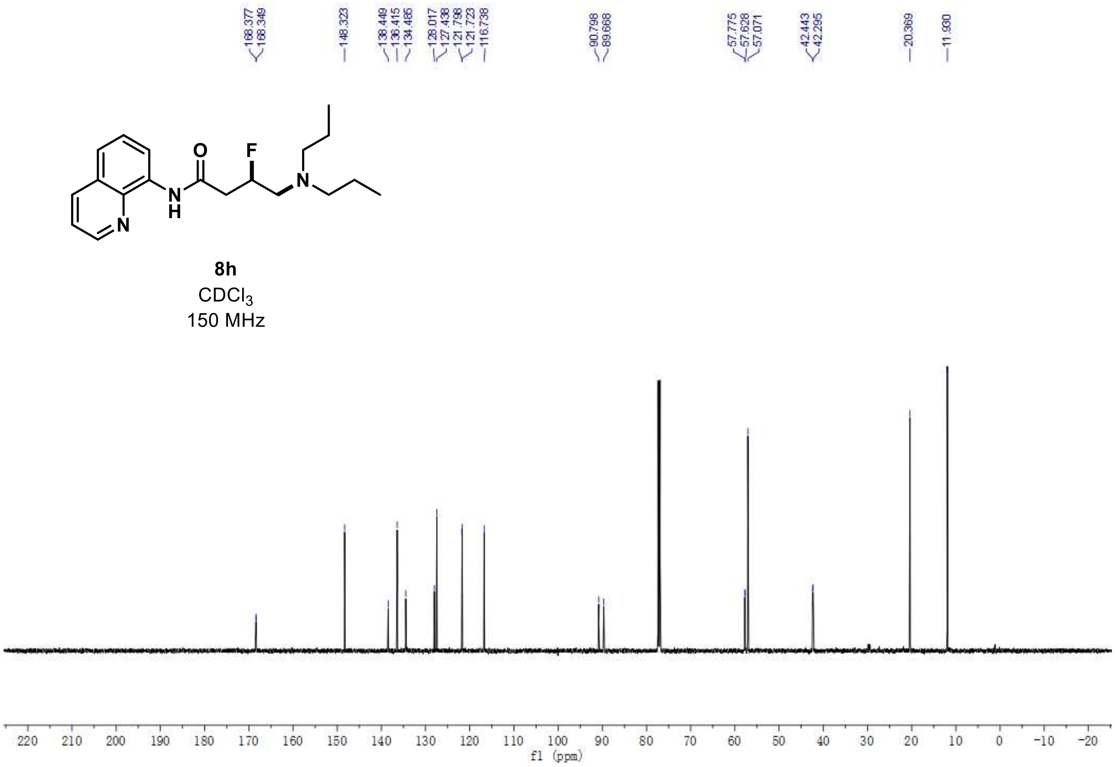

Figure S100. ^{13}C NMR Spectra of **8h**.

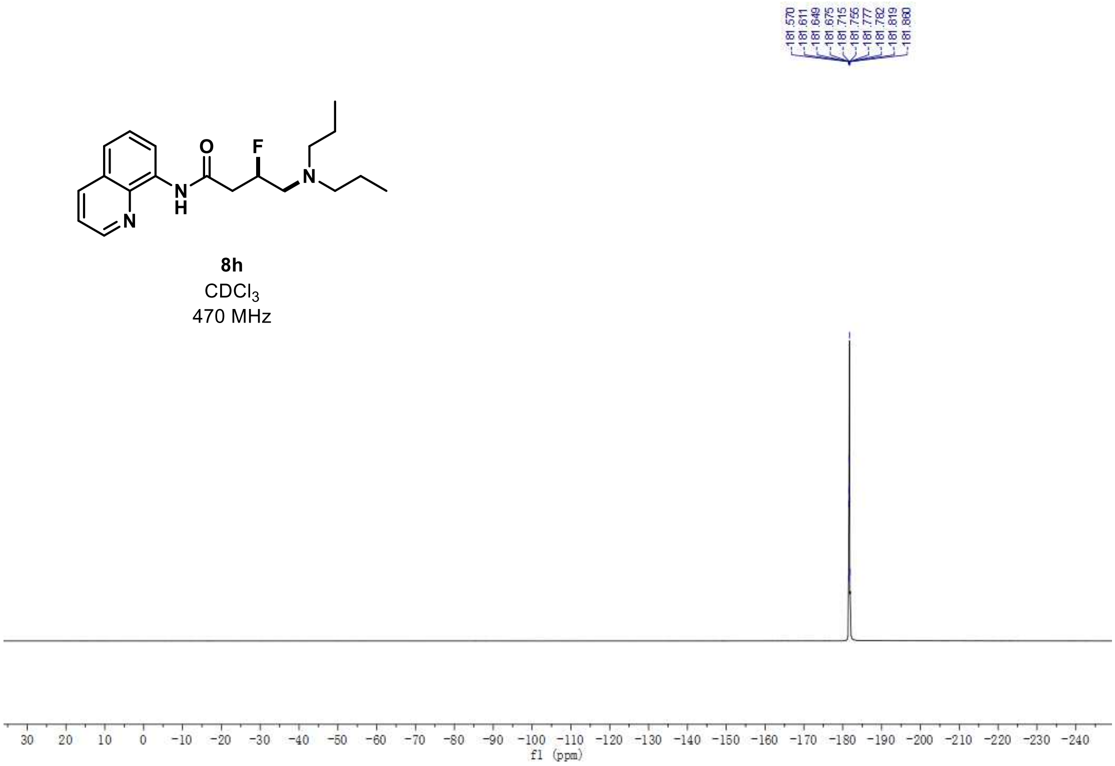

Figure S101. ^{19}F NMR Spectra of **8h**.

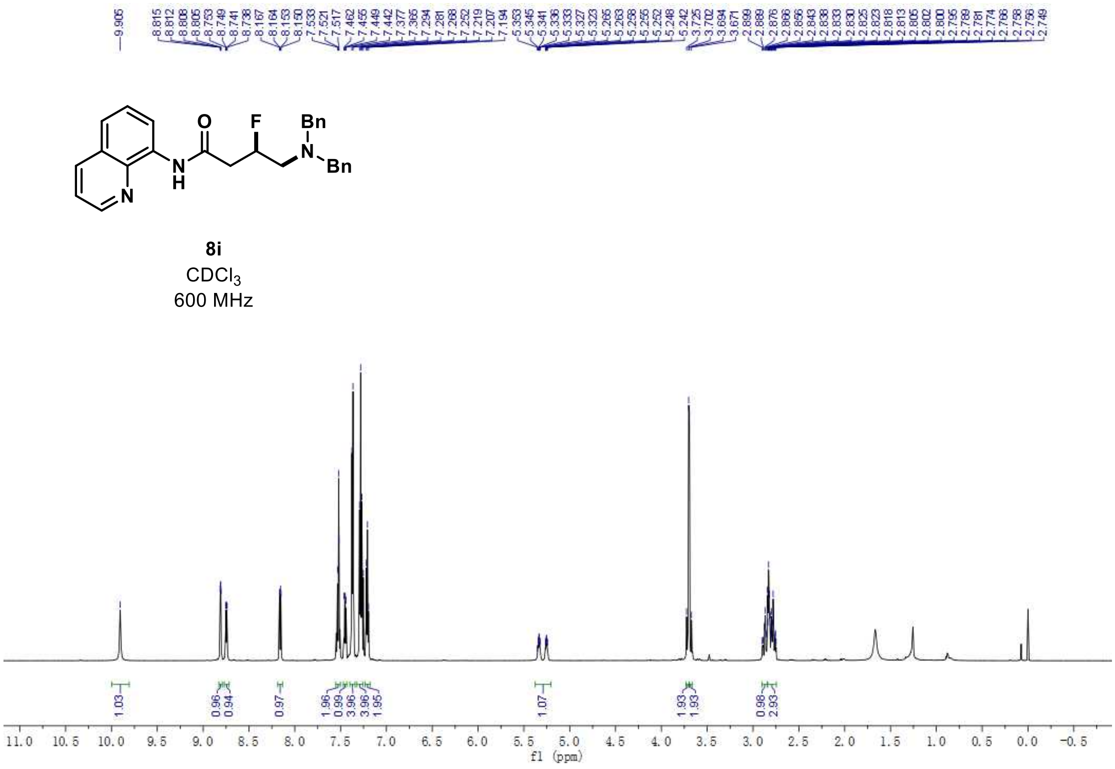

Figure S102. ^{1}H NMR Spectra of **8i**.

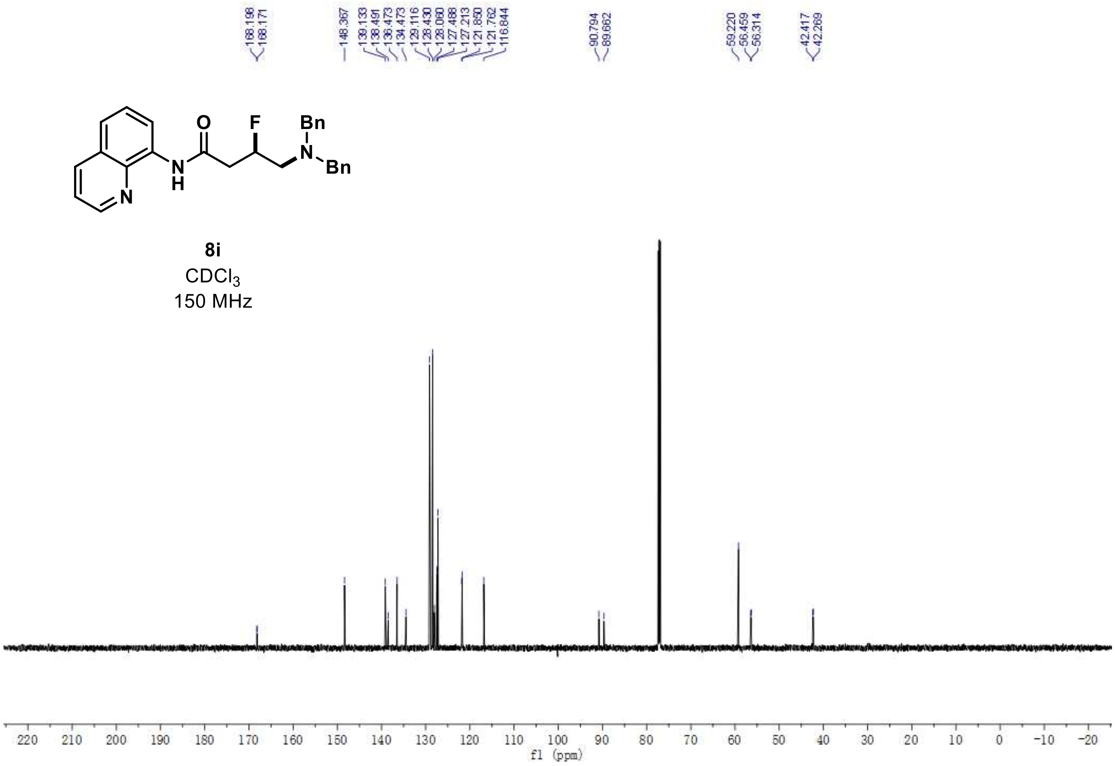

Figure S103. ^{13}C NMR Spectra of **8i**.

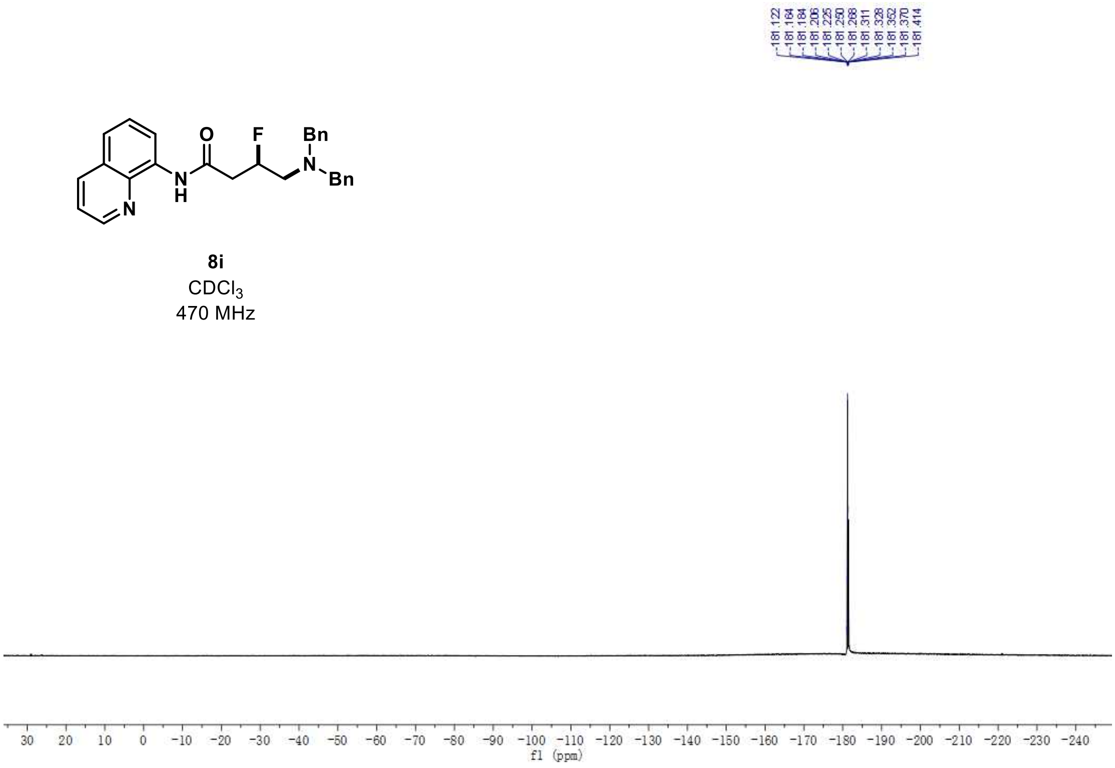

Figure S104. ^{19}F NMR Spectra of **8i**.

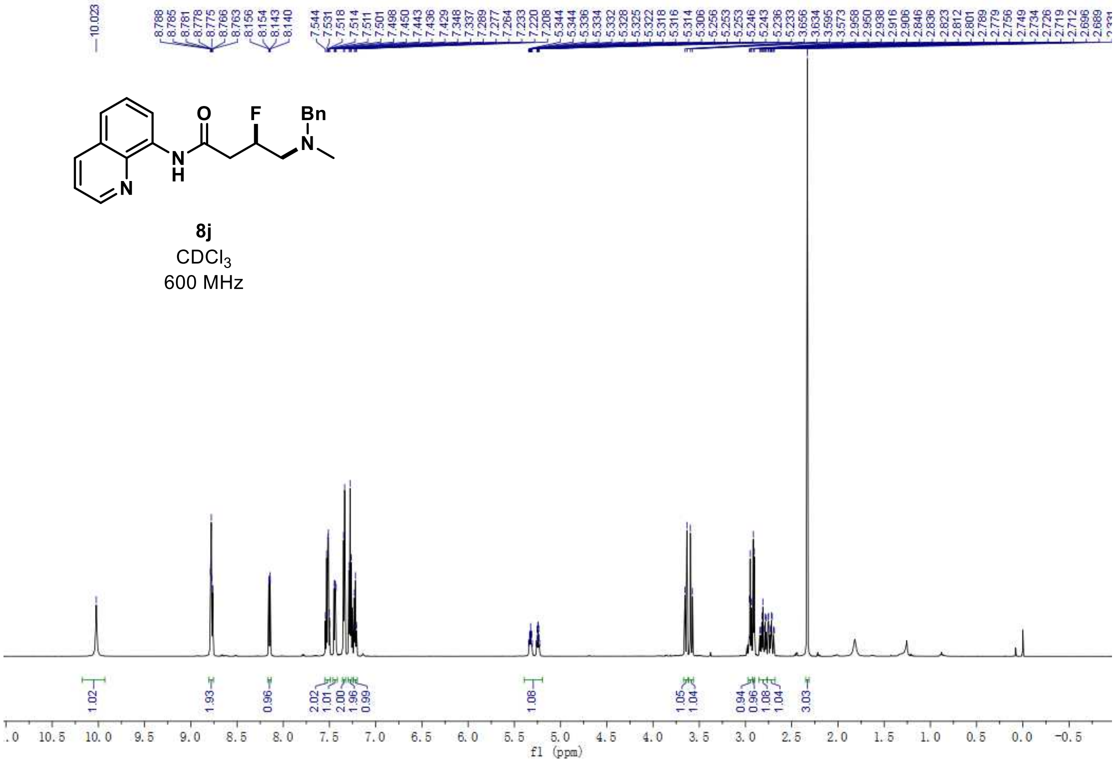

Figure S105. ^{1}H NMR Spectra of **8j**.

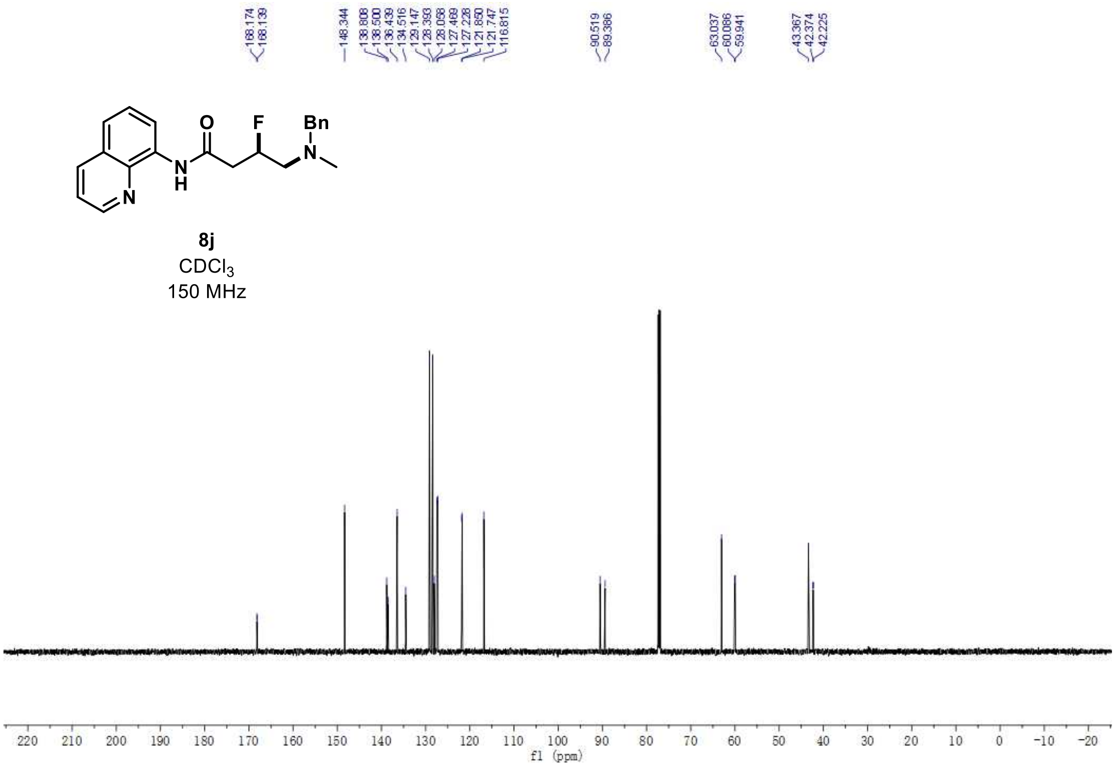

Figure S106. ^{13}C NMR Spectra of **8j**.

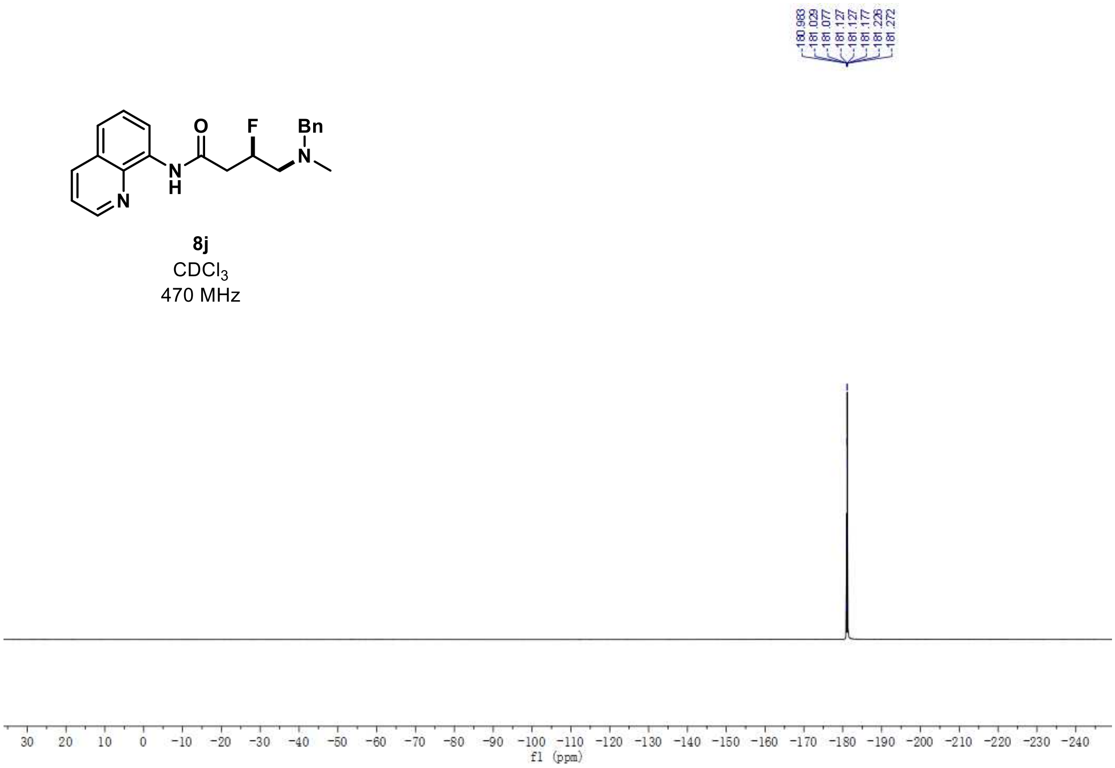

Figure S107. ^{19}F NMR Spectra of **8j**.

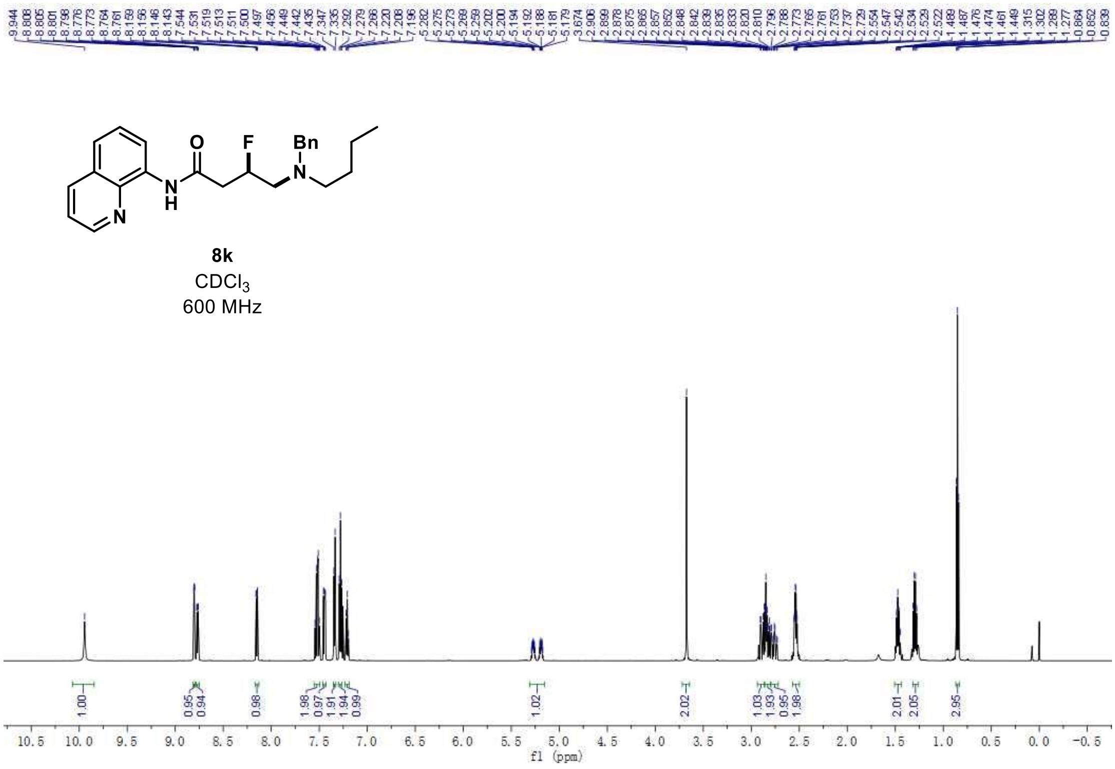

Figure S108. ^{1}H NMR Spectra of **8k**.

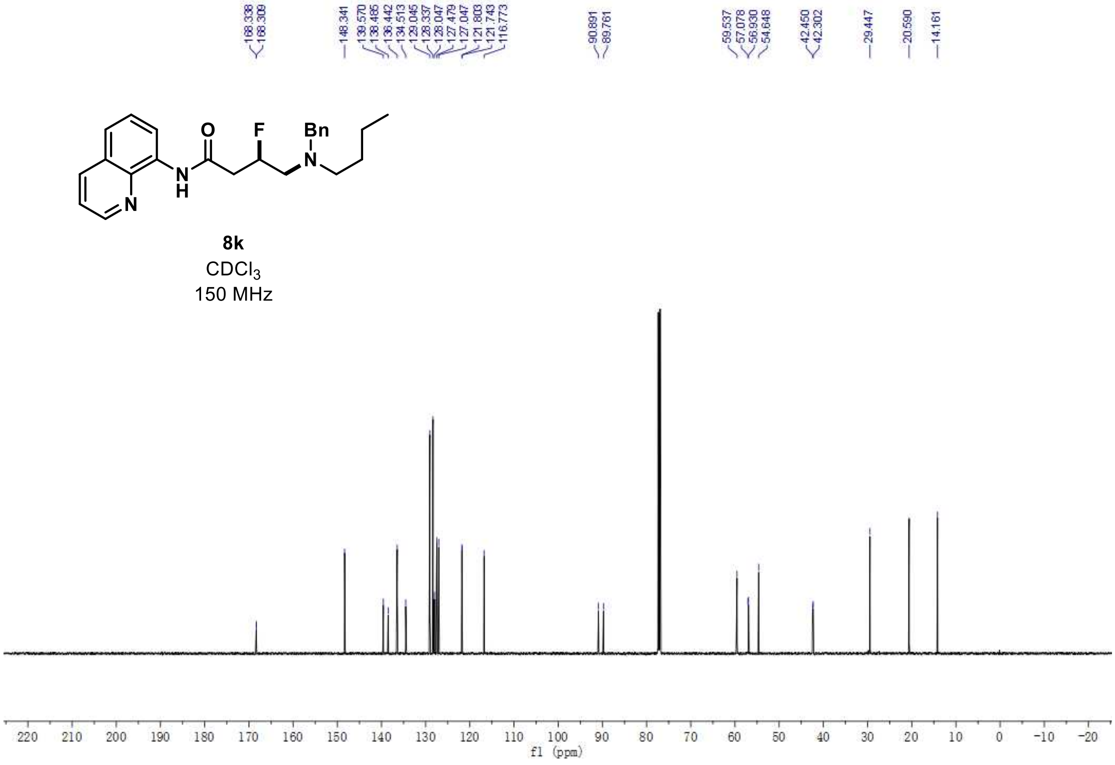

Figure S109. ^{13}C NMR Spectra of **8k**.

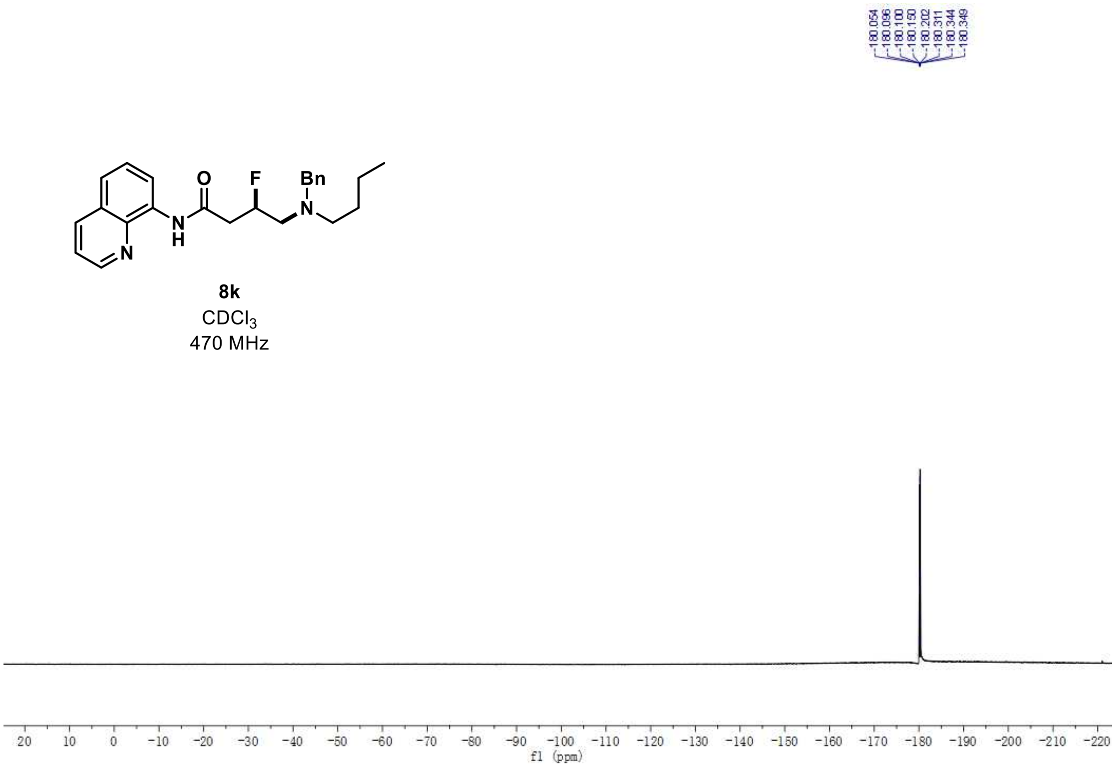

Figure S110. ^{19}F NMR Spectra of **8k**.

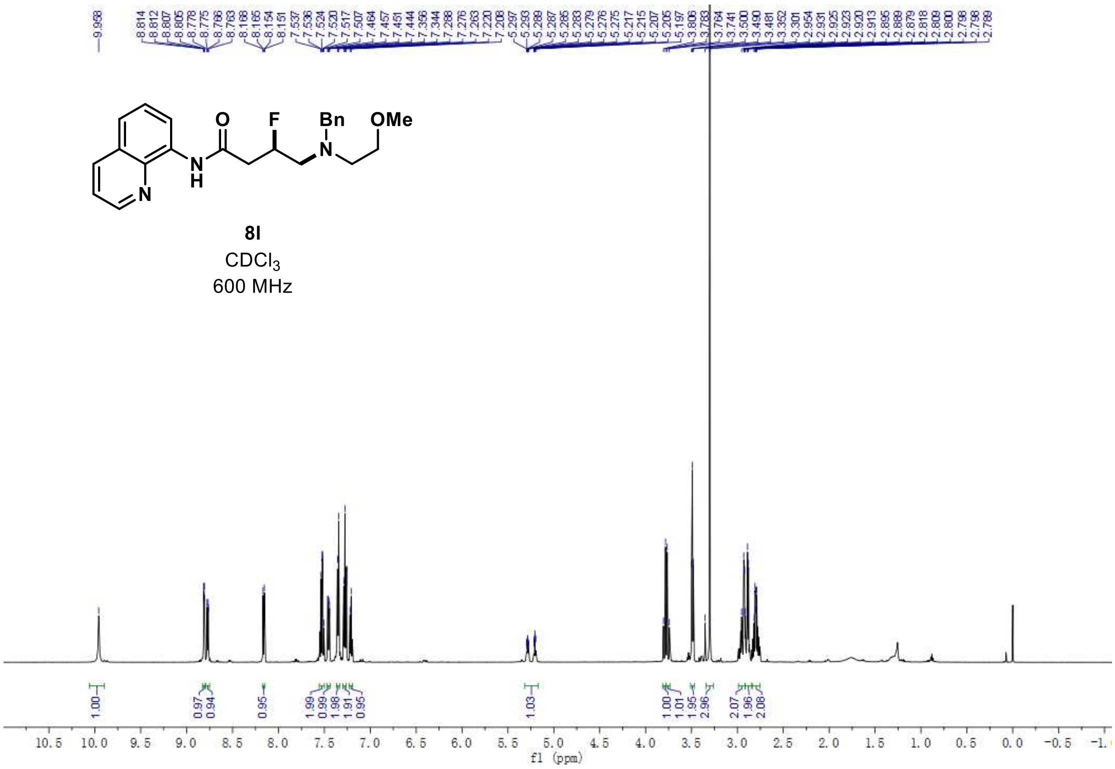

Figure S111. ^{1}H NMR Spectra of **8l**.

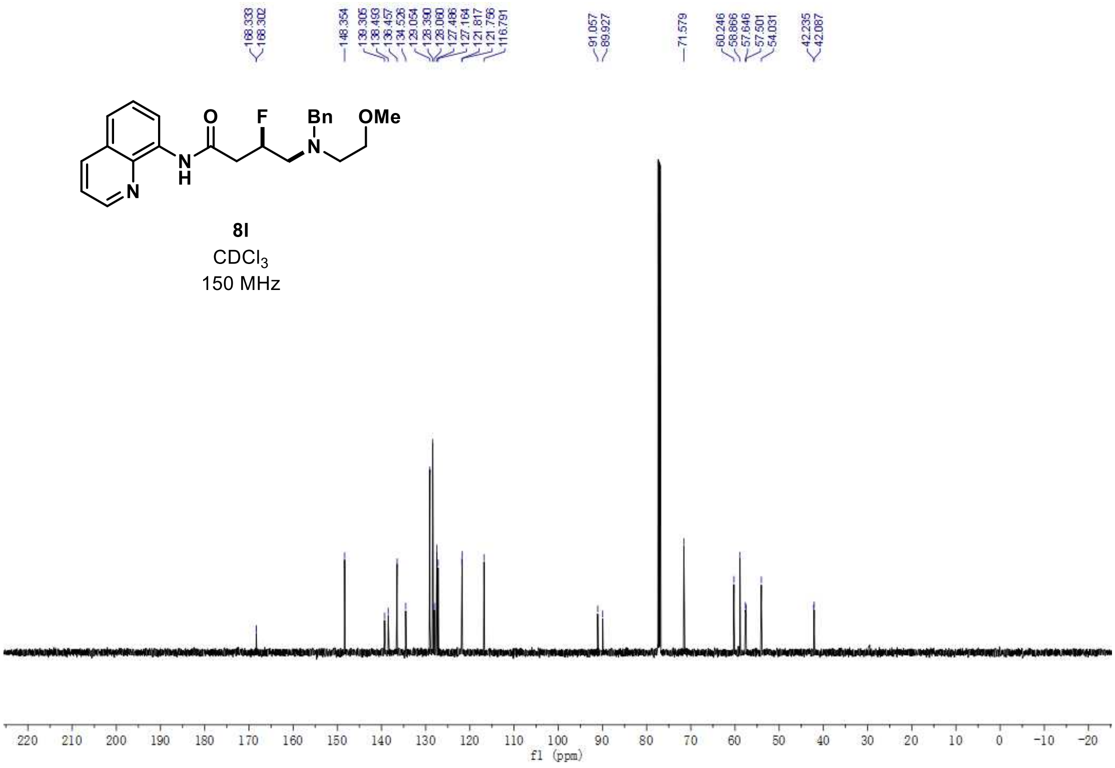

Figure S112. ^{13}C NMR Spectra of **8l**.

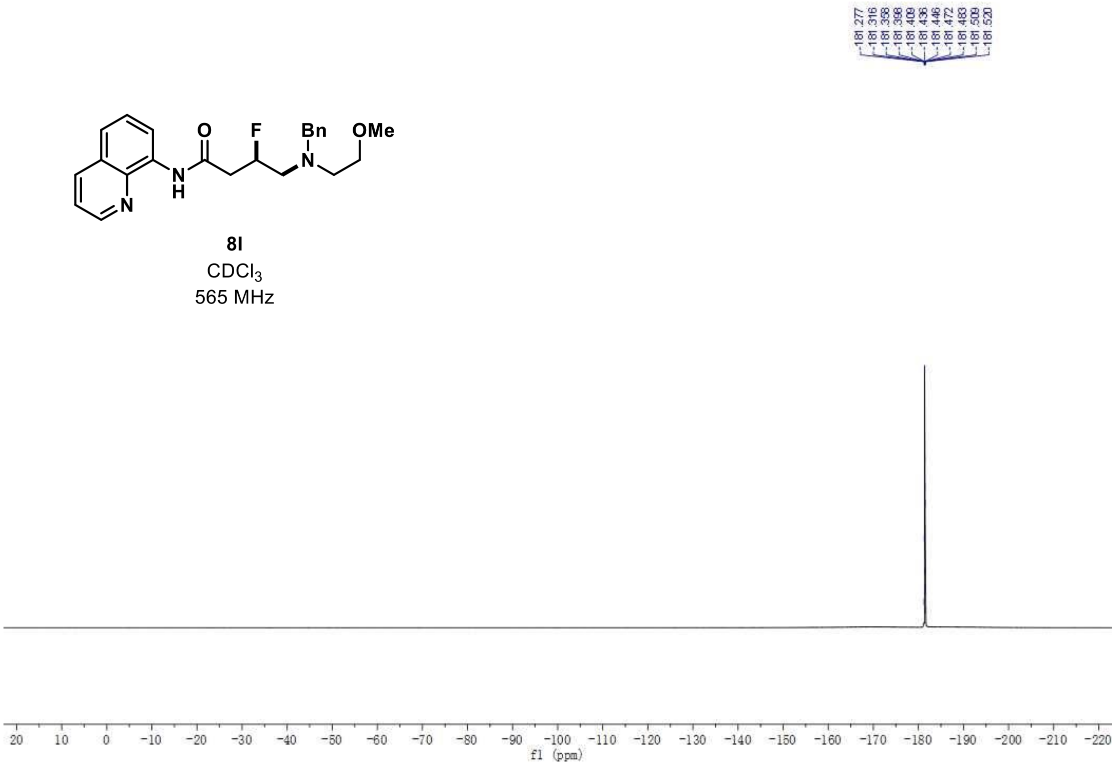

Figure S113. ^{19}F NMR Spectra of **8l**.

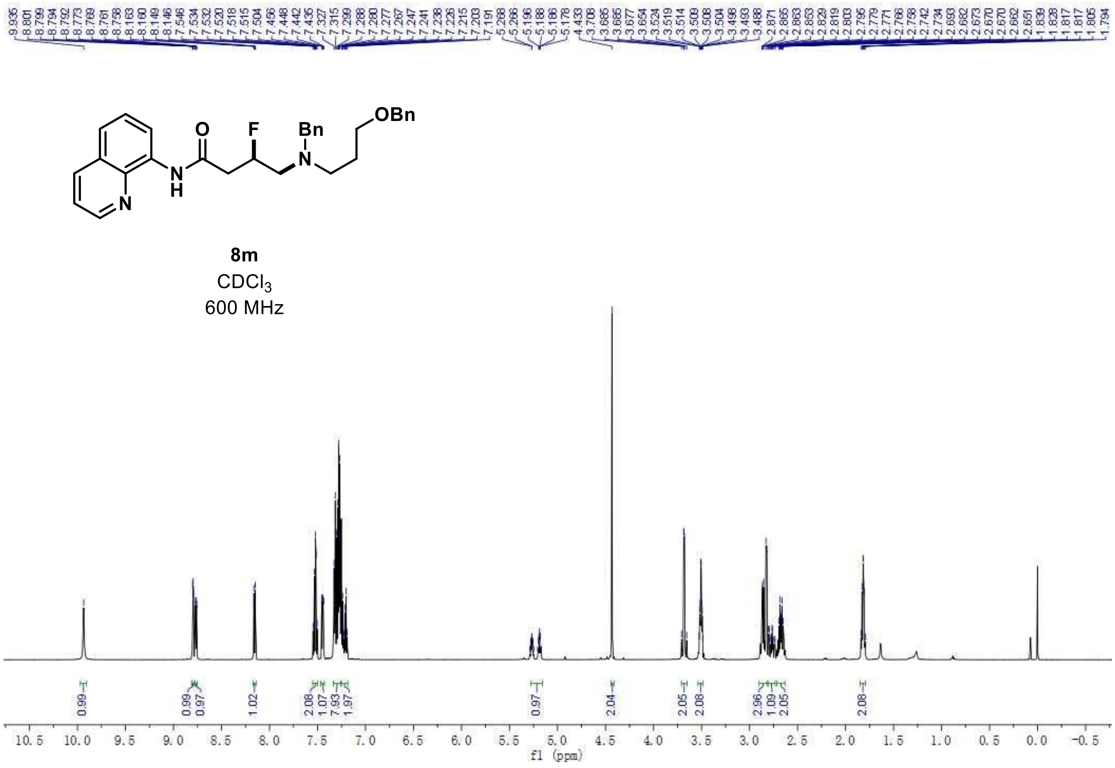

Figure S114. ^{1}H NMR Spectra of **8m**.

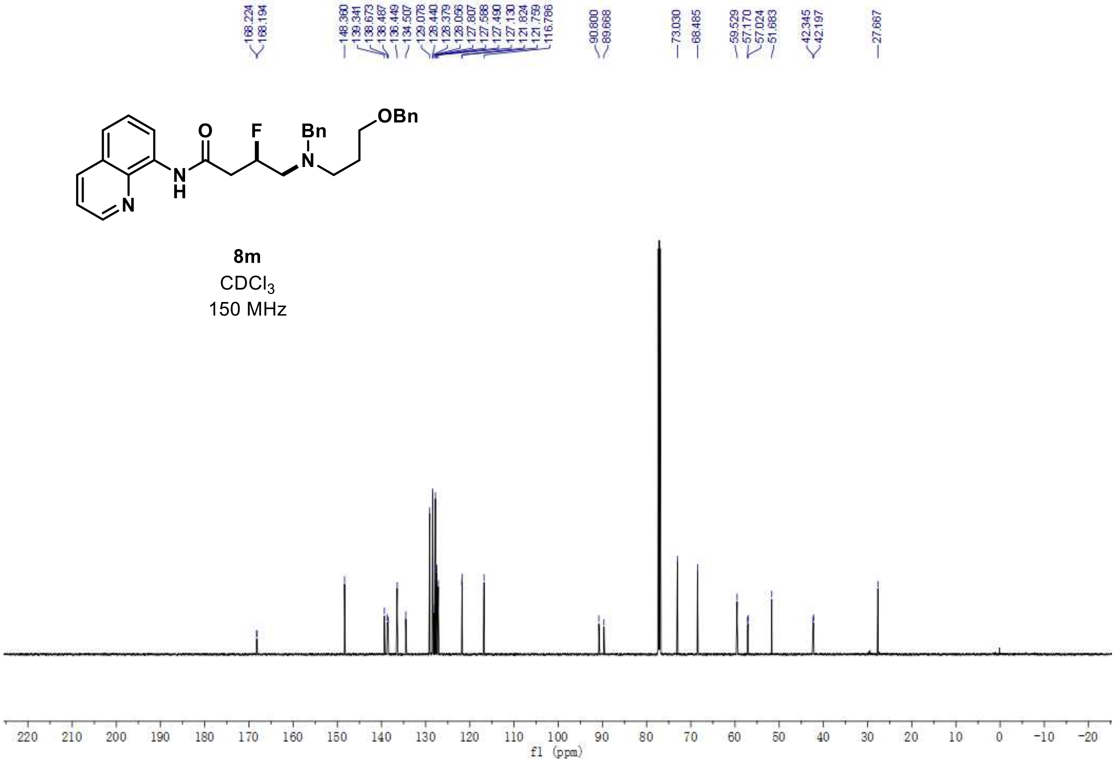

Figure S115. ^{13}C NMR Spectra of **8m**.

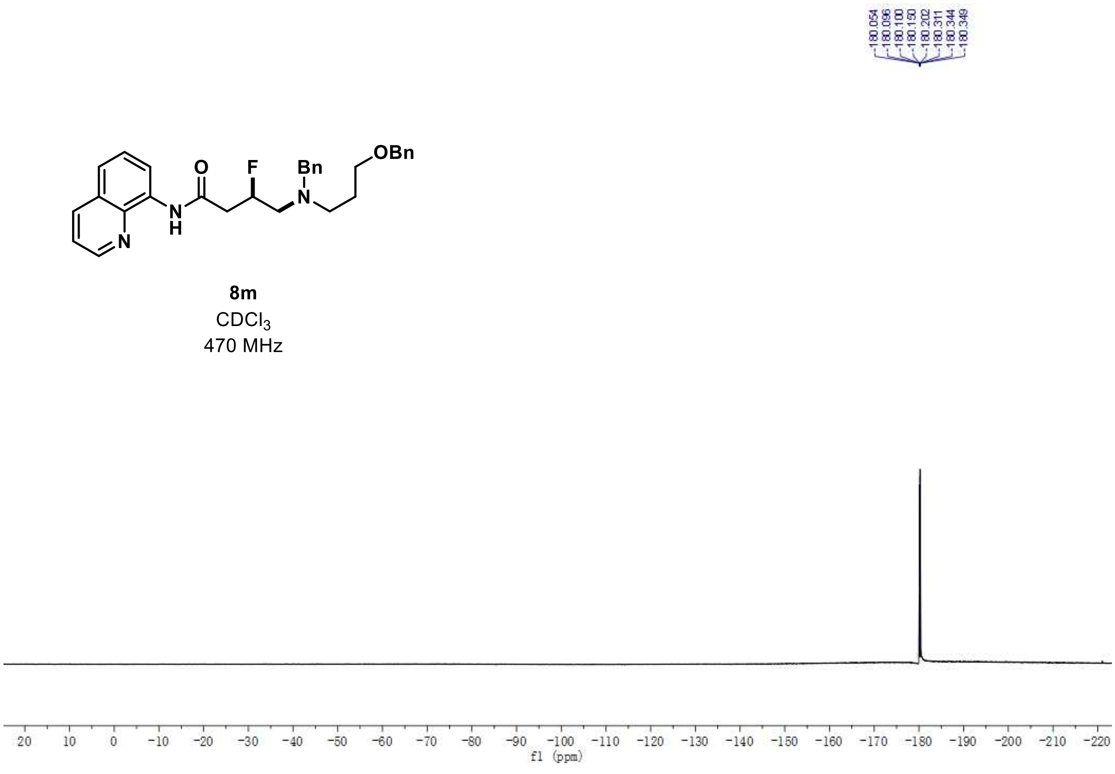

Figure S116. ^{19}F NMR Spectra of **8m**.

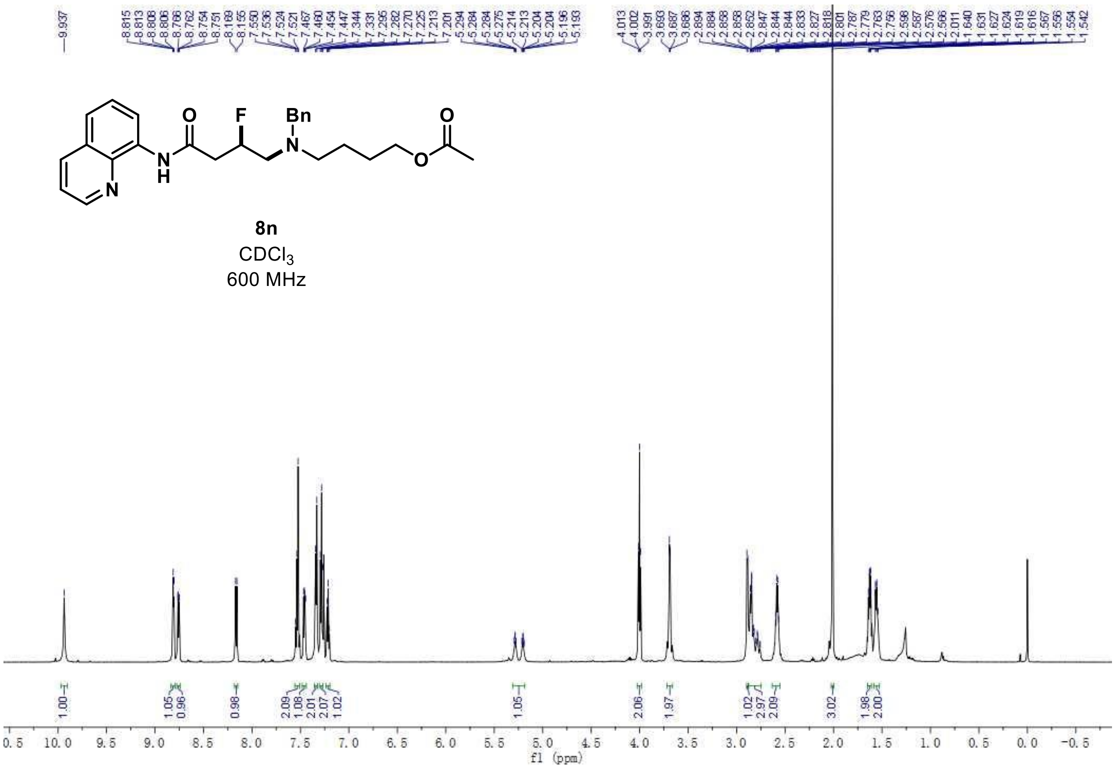

Figure S117. ^{1}H NMR Spectra of **8n**.

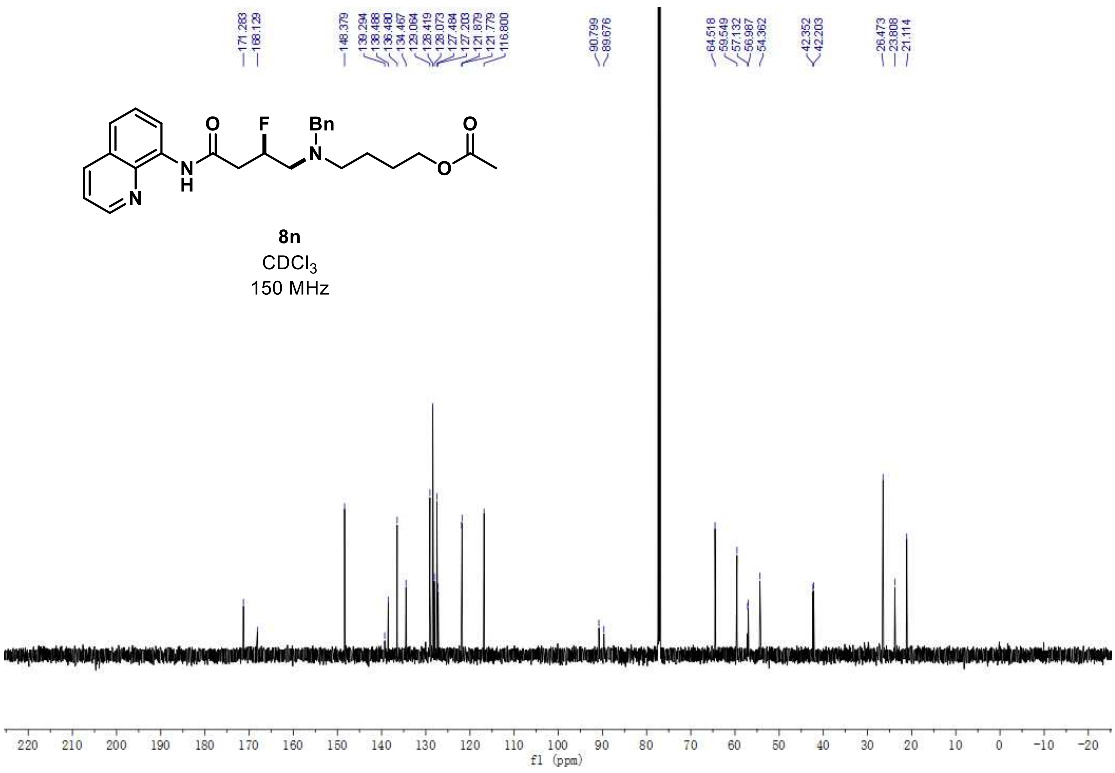

Figure S118. ^{13}C NMR Spectra of **8n**.

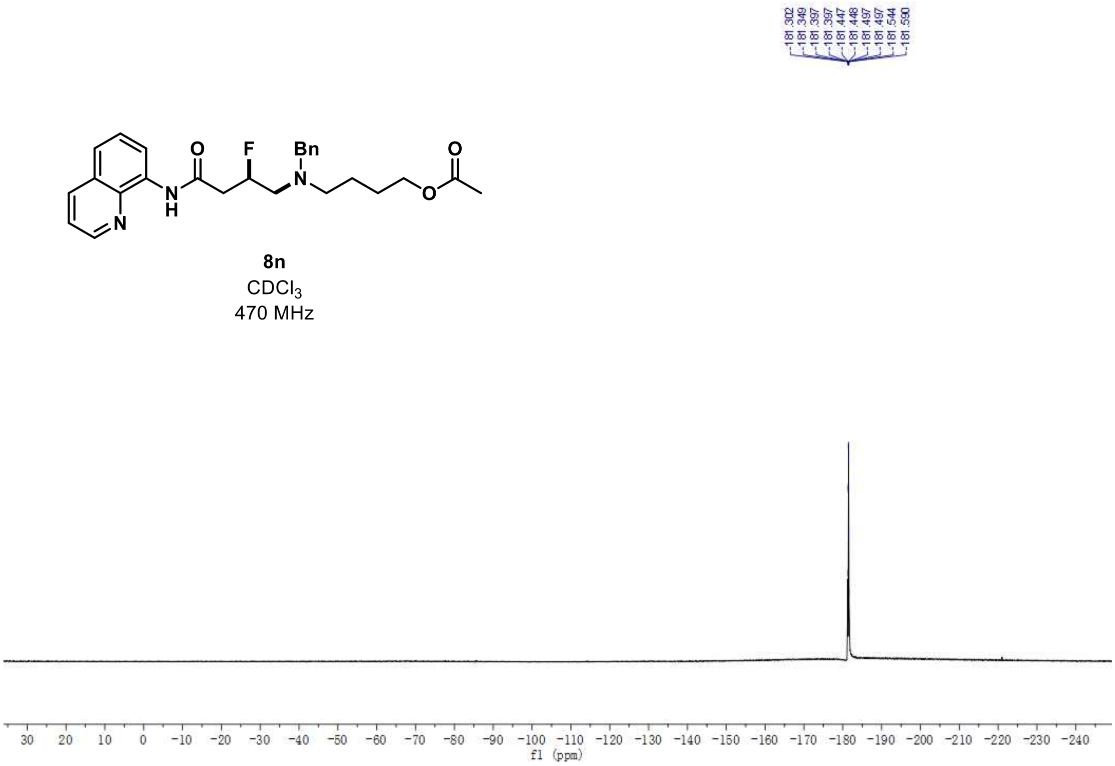

Figure S119. ^{19}F NMR Spectra of **8n**.

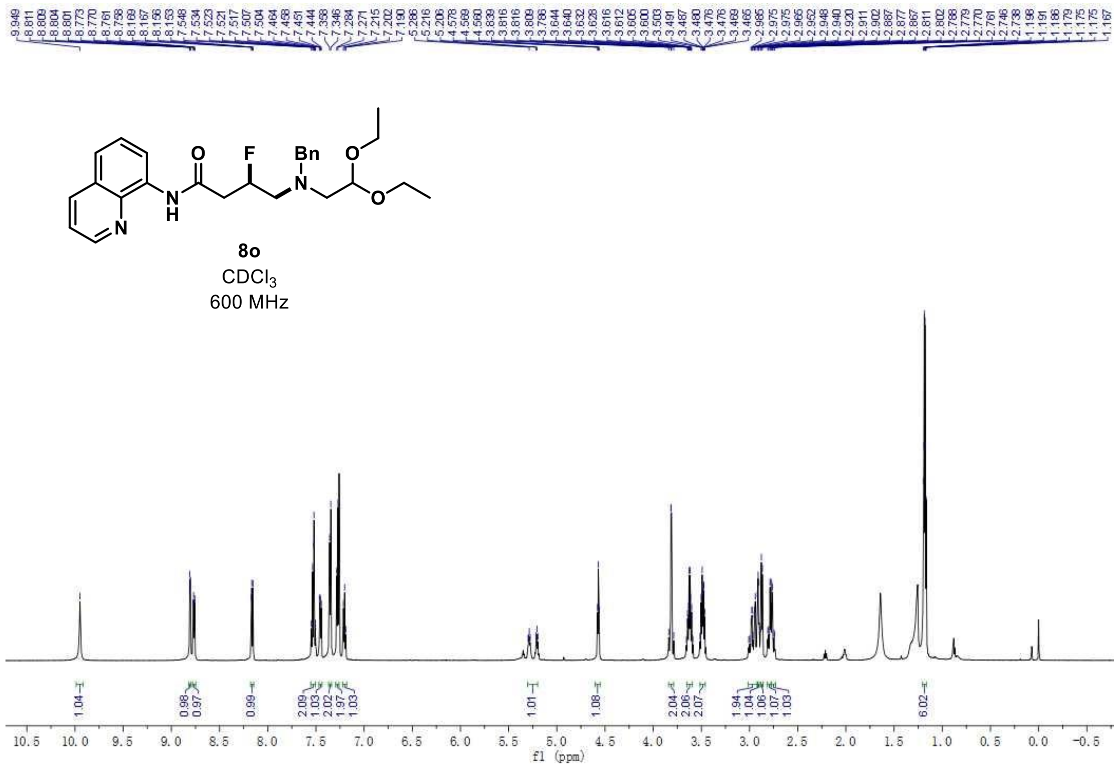

Figure S120. ^{1}H NMR Spectra of **8o**.

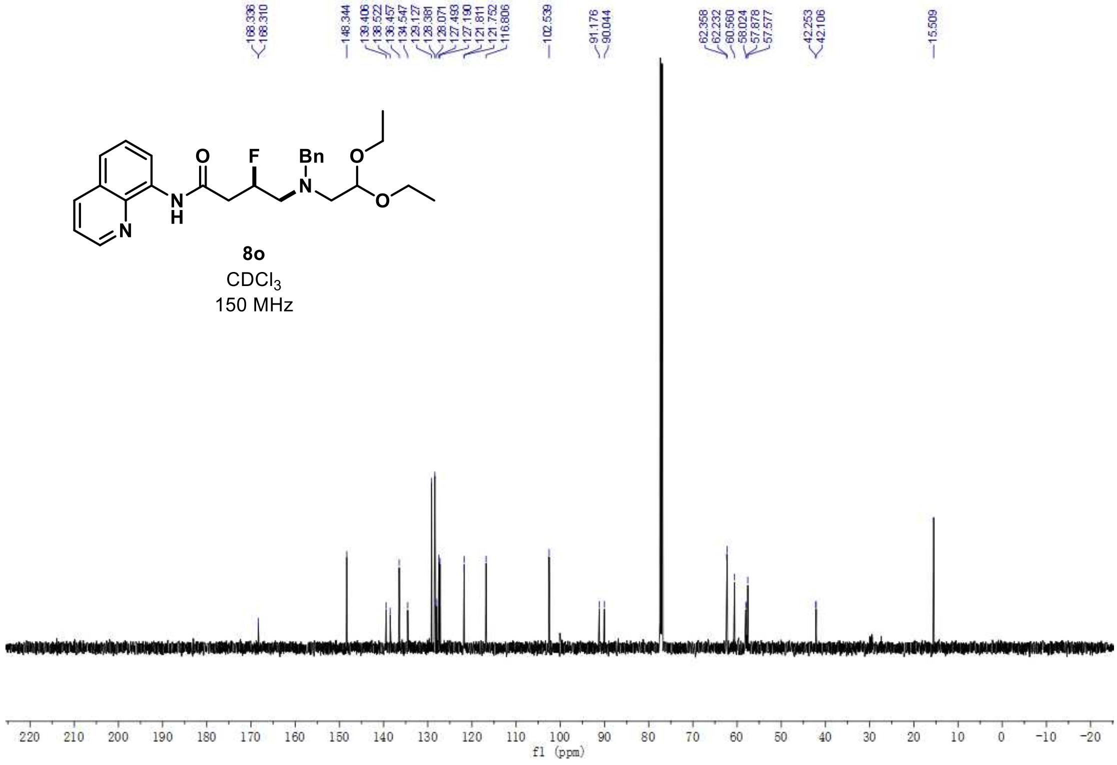

Figure S121. ^{13}C NMR Spectra of **8o**.

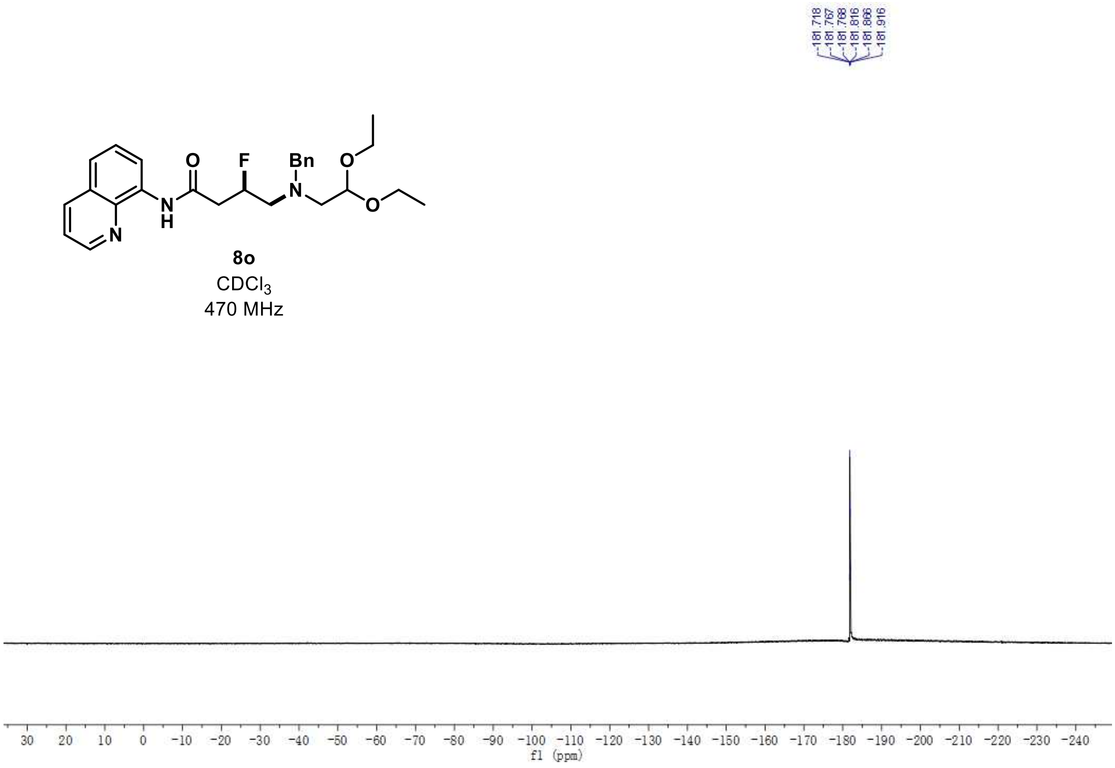

Figure S122. ^{19}F NMR Spectra of **8o**.

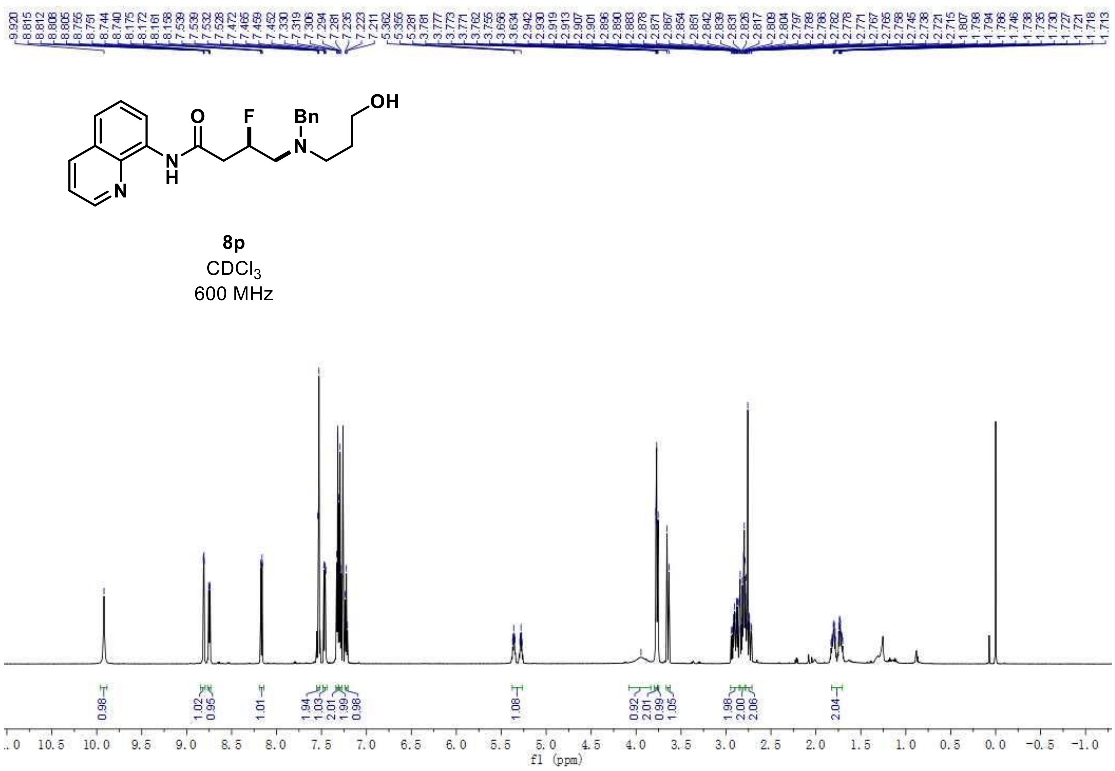

Figure S123. ^{1}H NMR Spectra of **8p**.

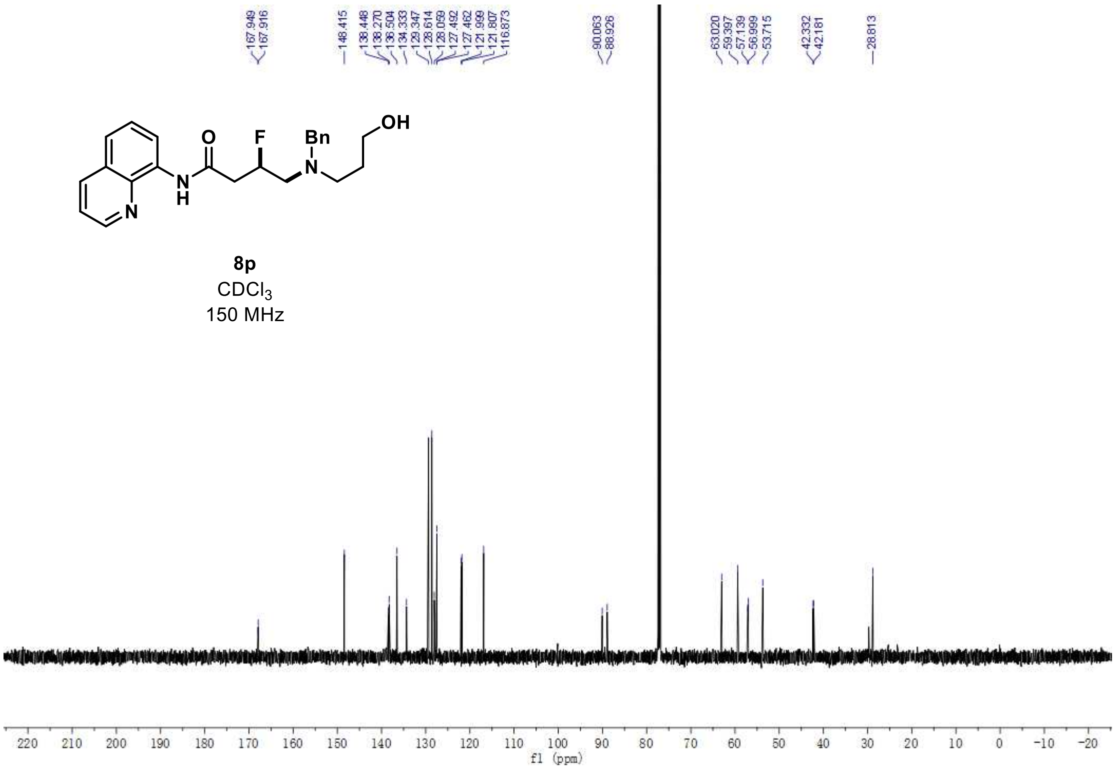

Figure S124. ^{13}C NMR Spectra of **8p**.

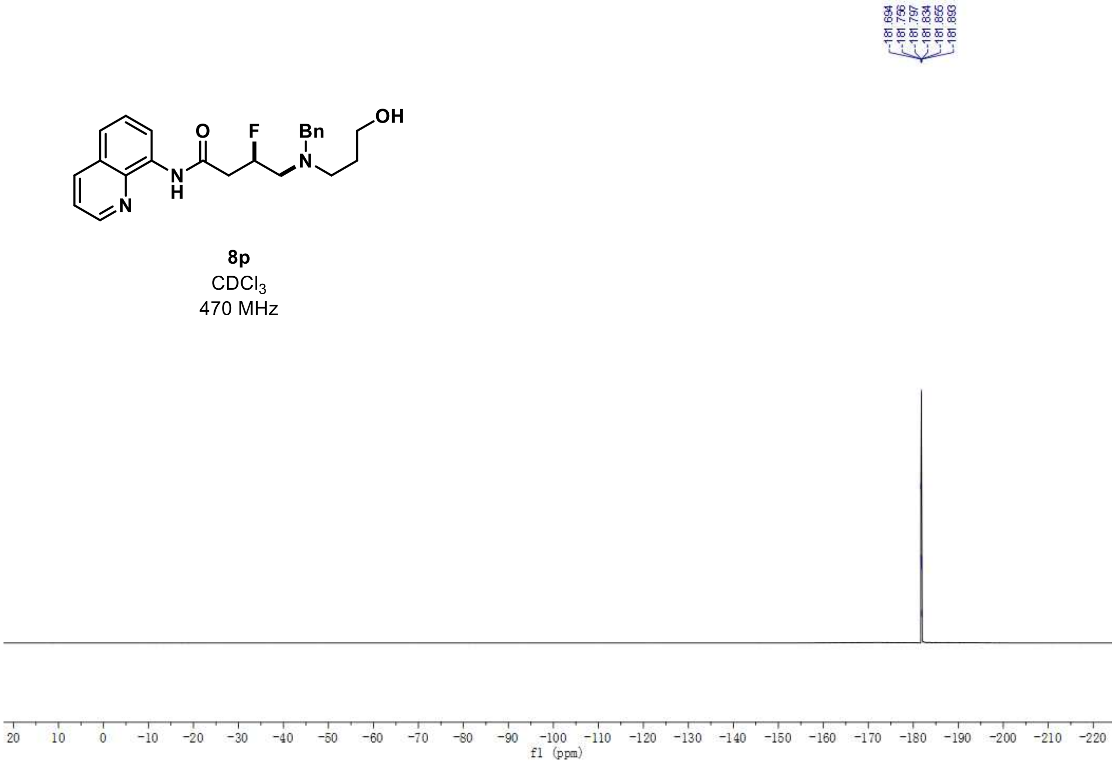

Figure S125. ^{19}F NMR Spectra of **8p**.

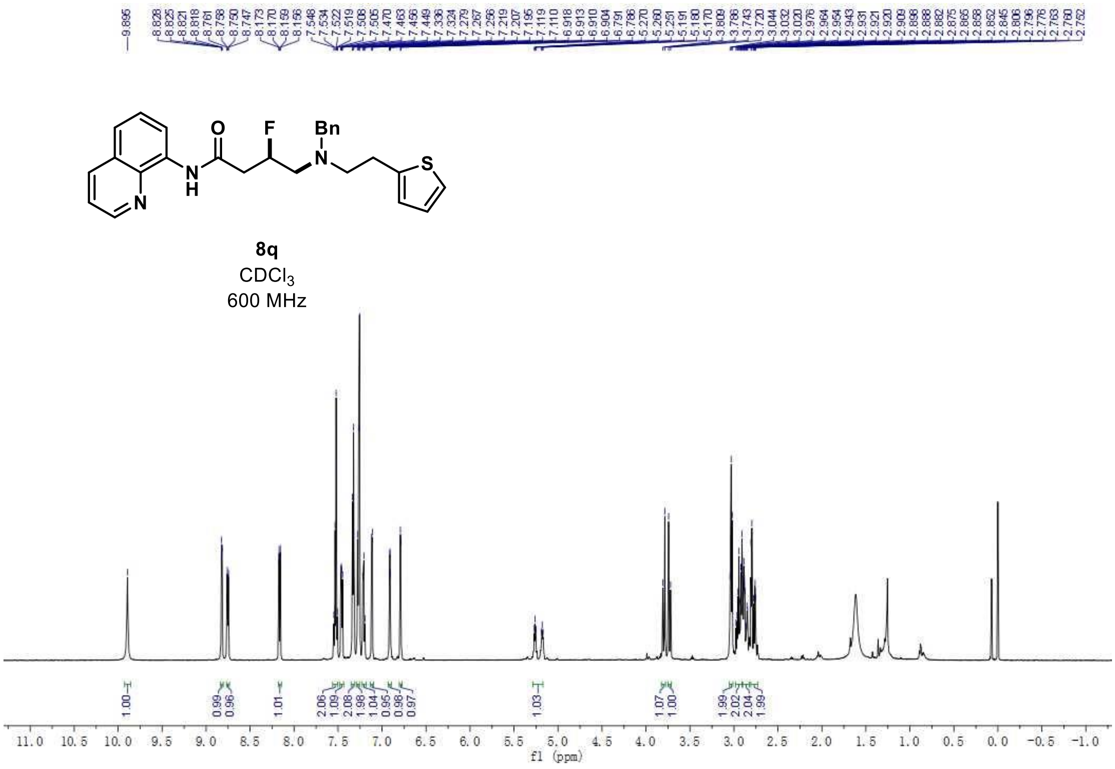

Figure S126. ^{1}H NMR Spectra of **8q**.

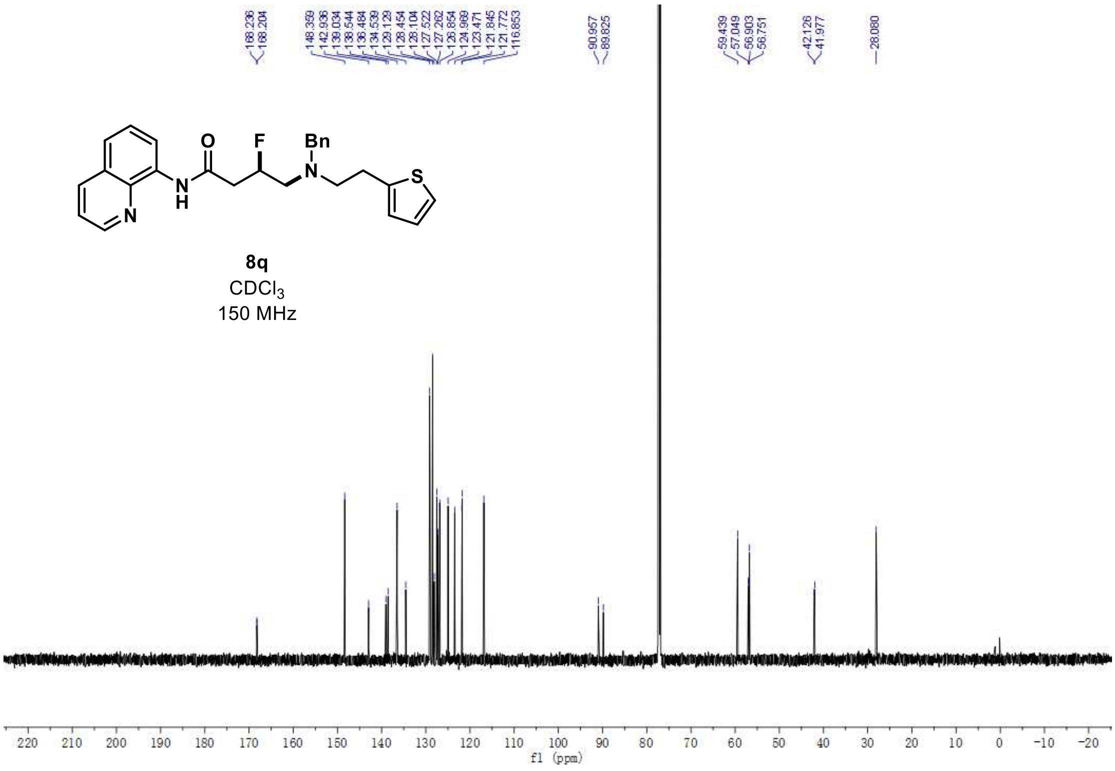

Figure S127. ^{13}C NMR Spectra of **8q**.

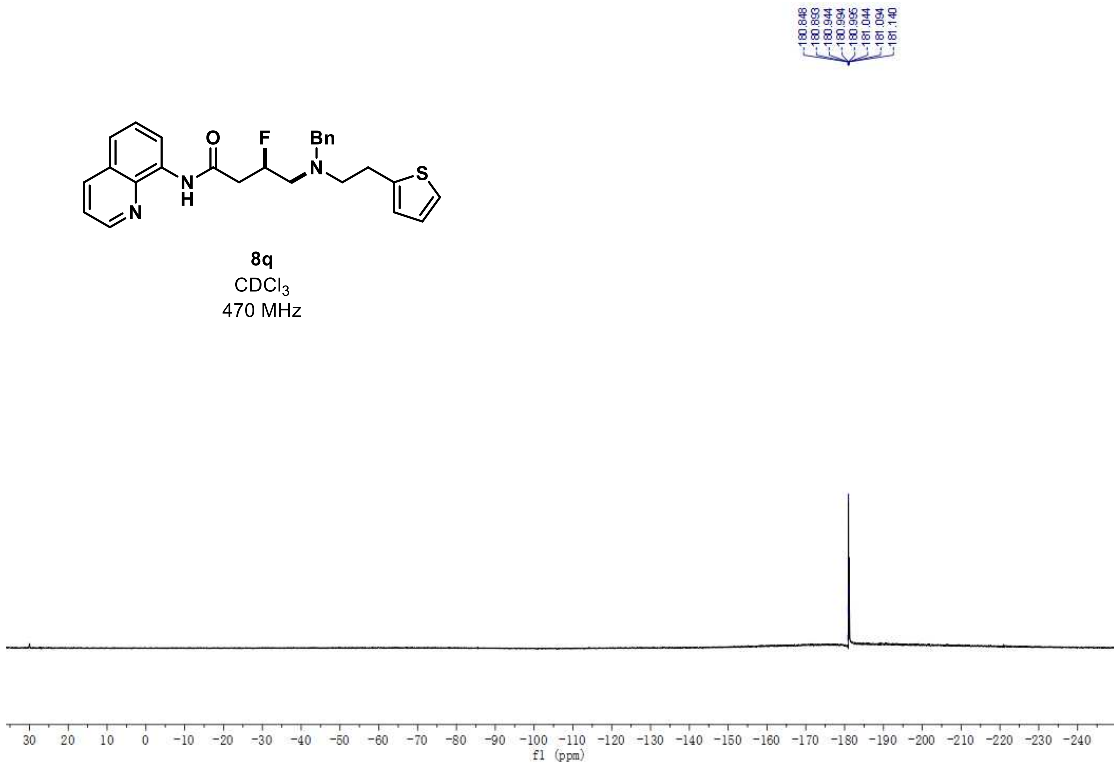

Figure S128. ^{19}F NMR Spectra of **8q**.

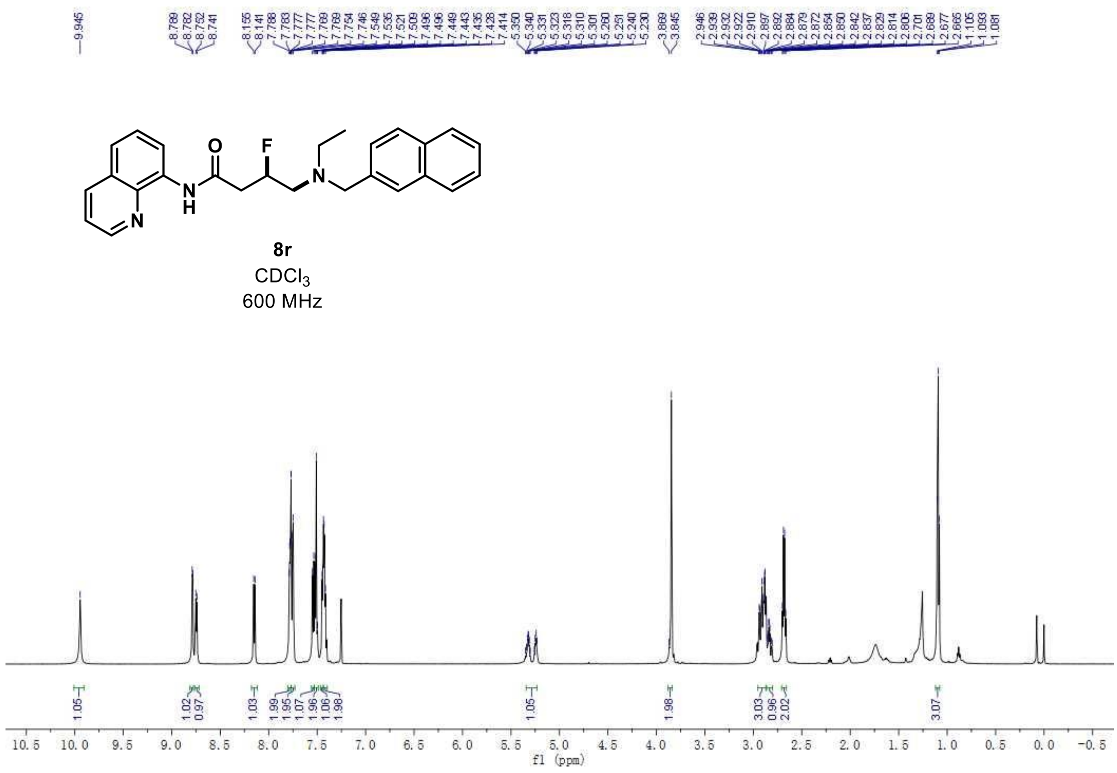

Figure S129. ^{1}H NMR Spectra of **8r**.

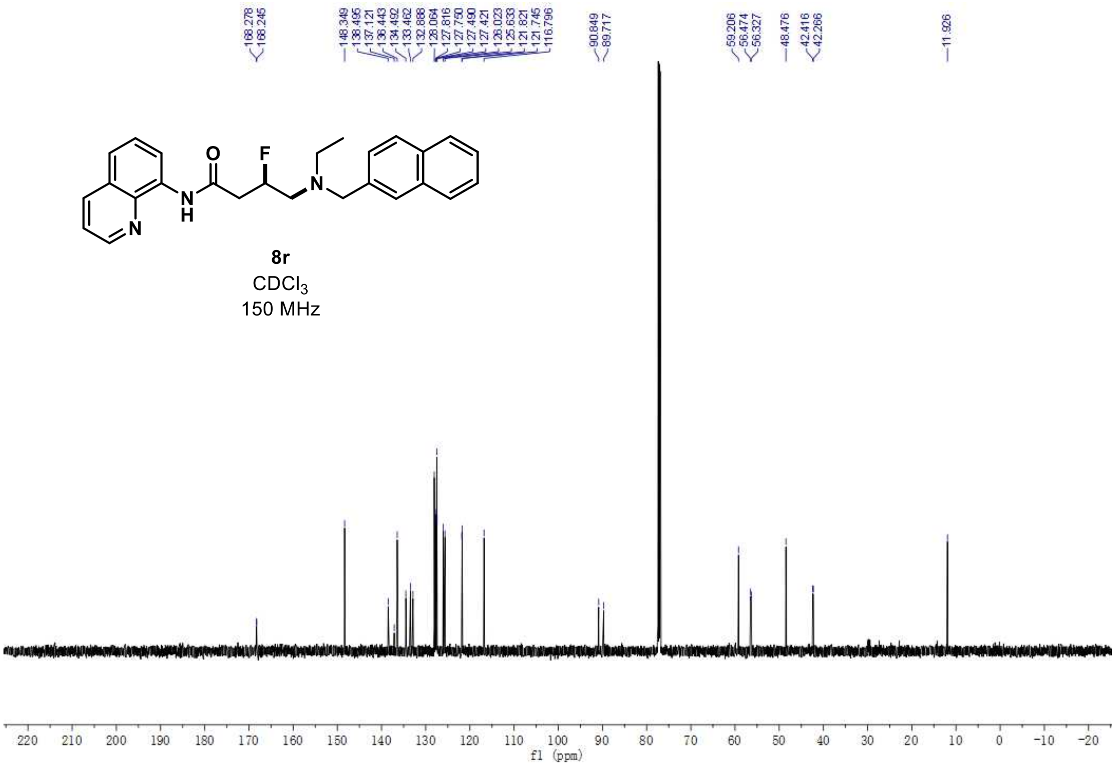

Figure S130. ^{13}C NMR Spectra of **8r**.

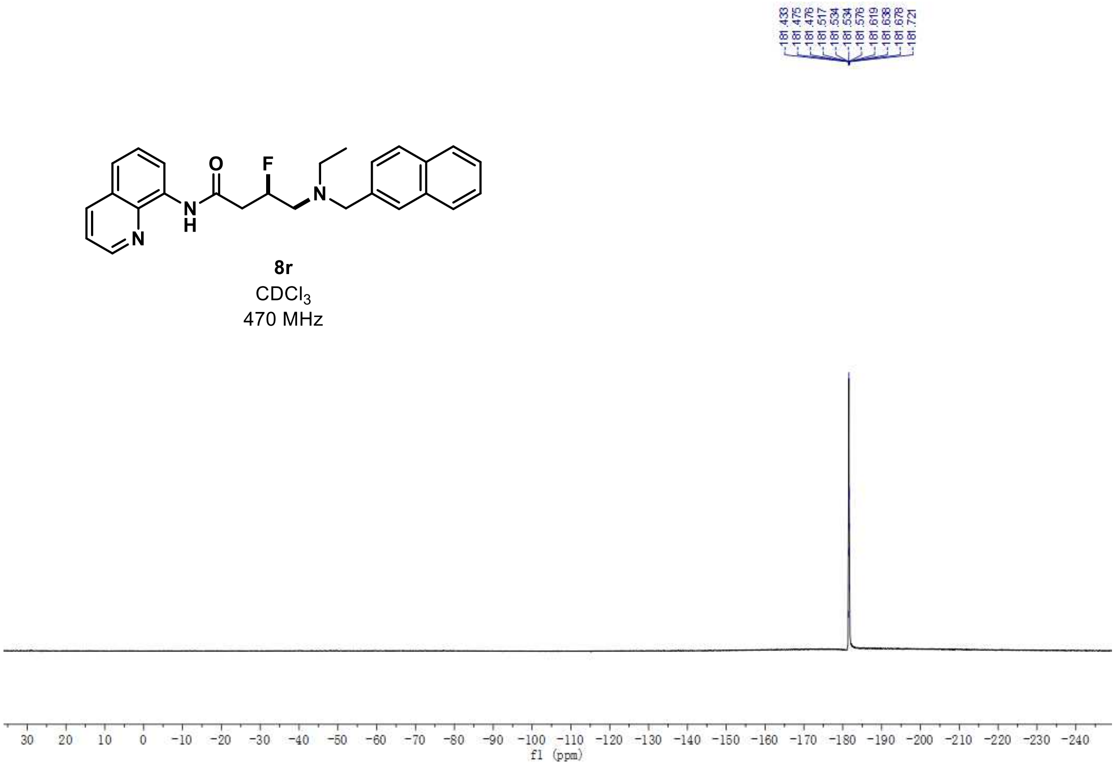

Figure S131. ^{19}F NMR Spectra of **8r**.

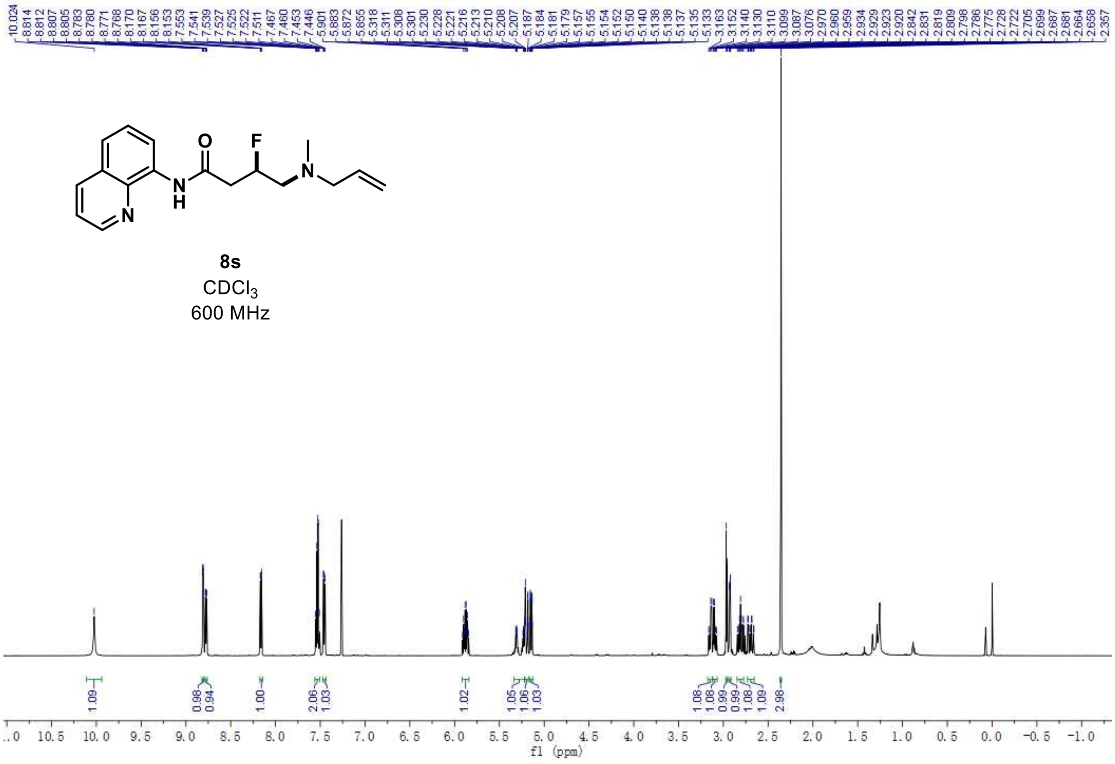

Figure S132. ^{1}H NMR Spectra of **8s**.

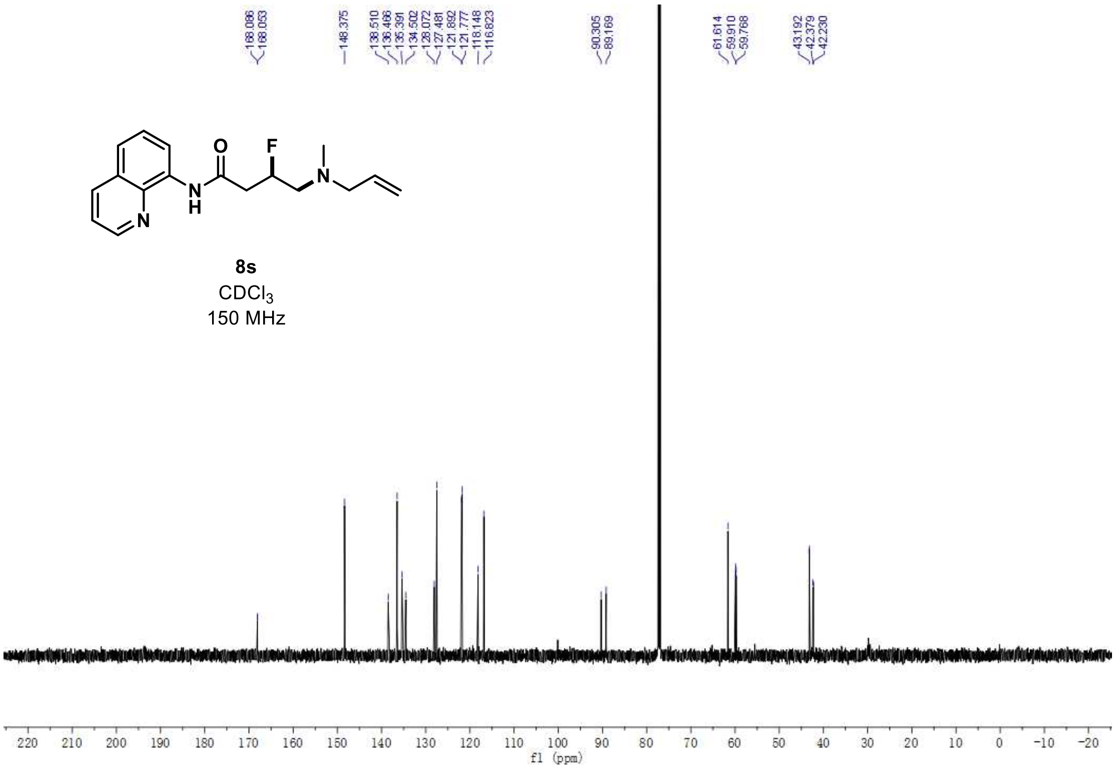

Figure S133. ^{13}C NMR Spectra of **8s**.

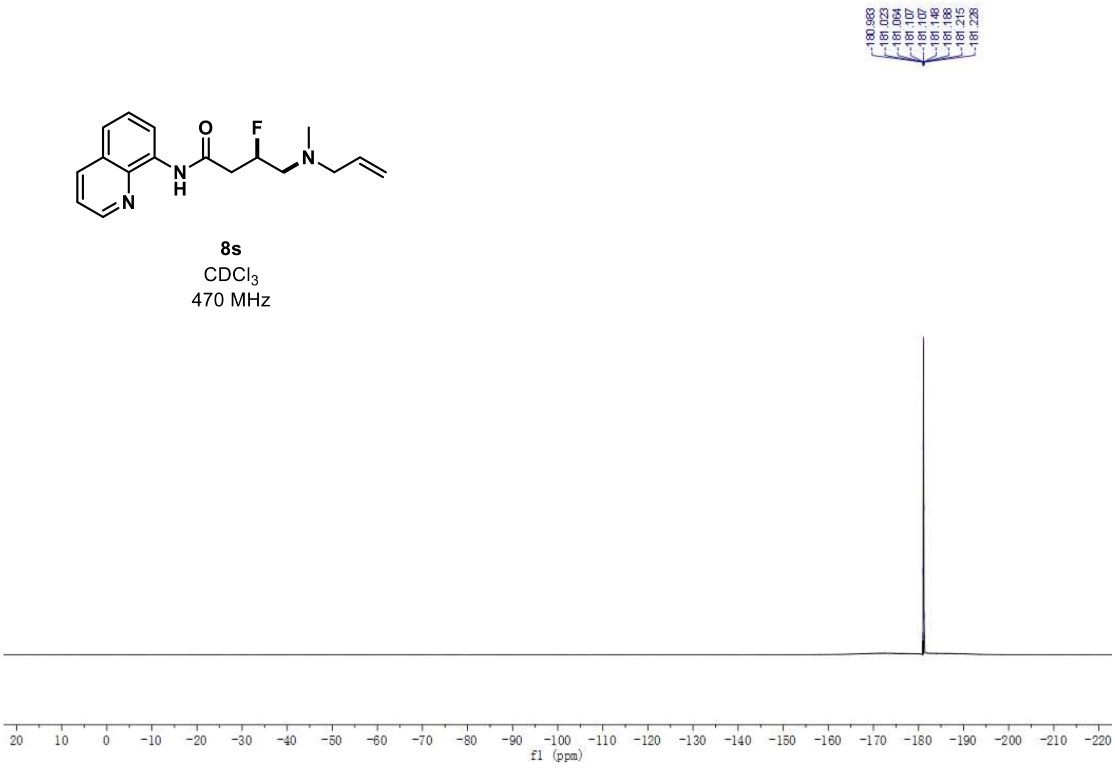

Figure S134. ^{19}F NMR Spectra of **8s**.

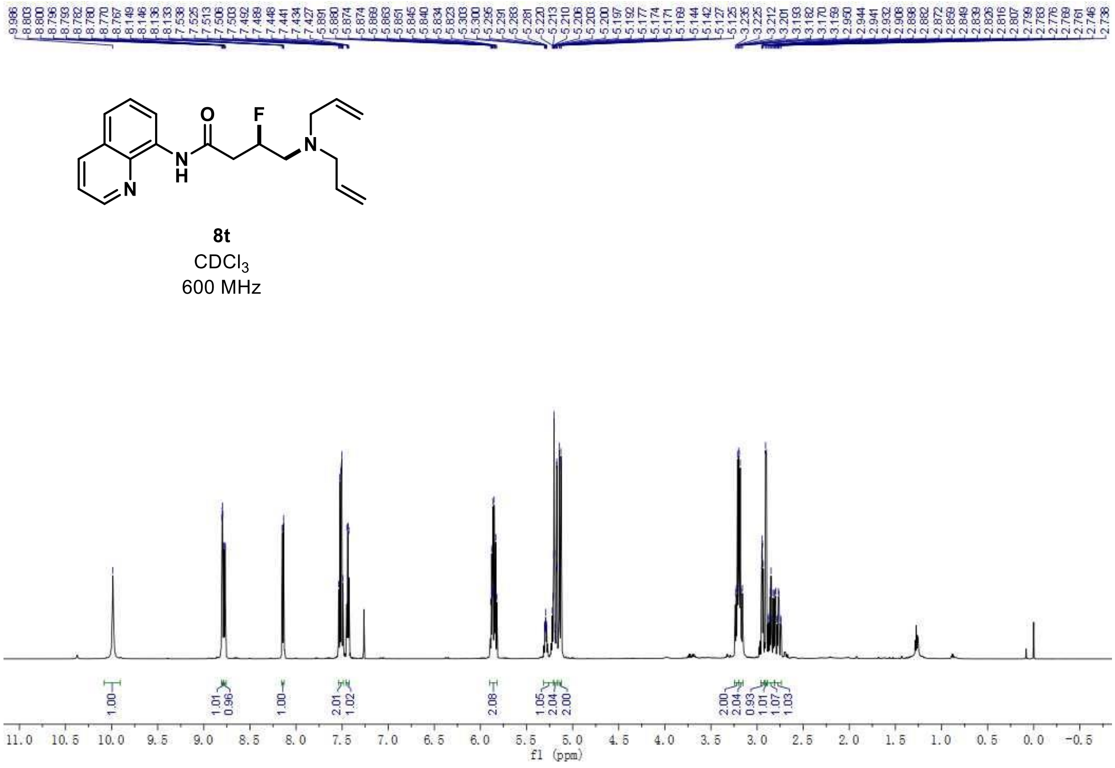

Figure S135. ^{1}H NMR Spectra of **8t**.

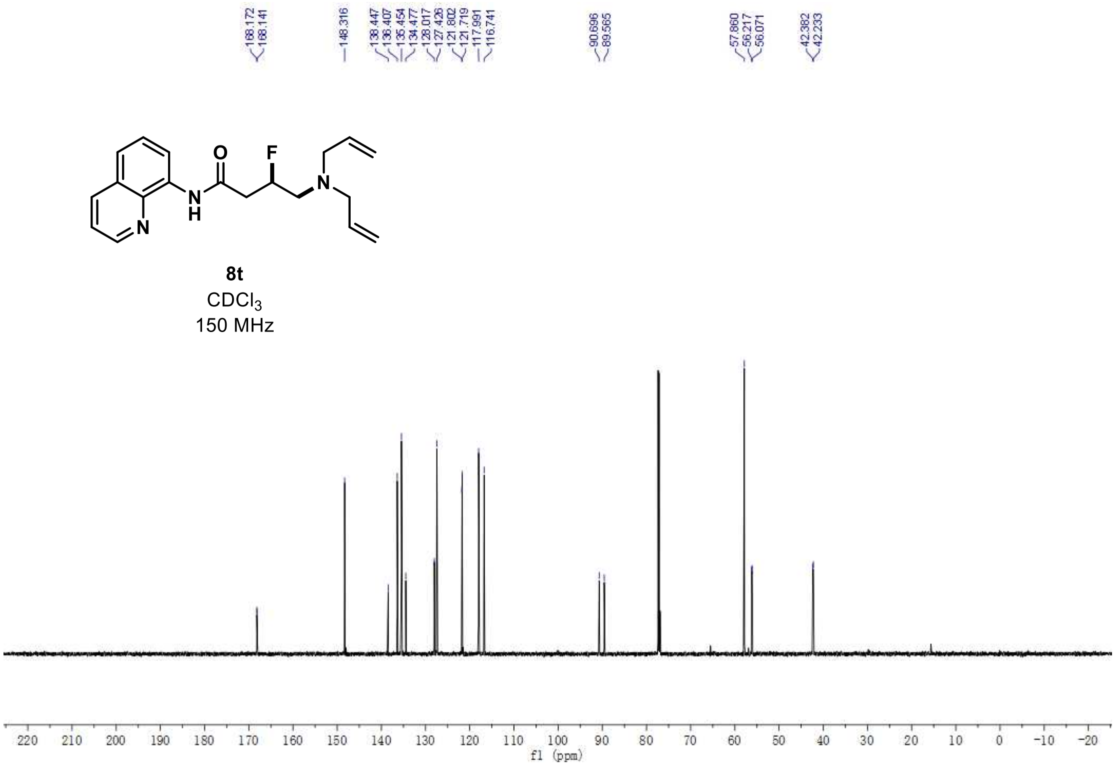

Figure S136. ^{13}C NMR Spectra of **8t**.

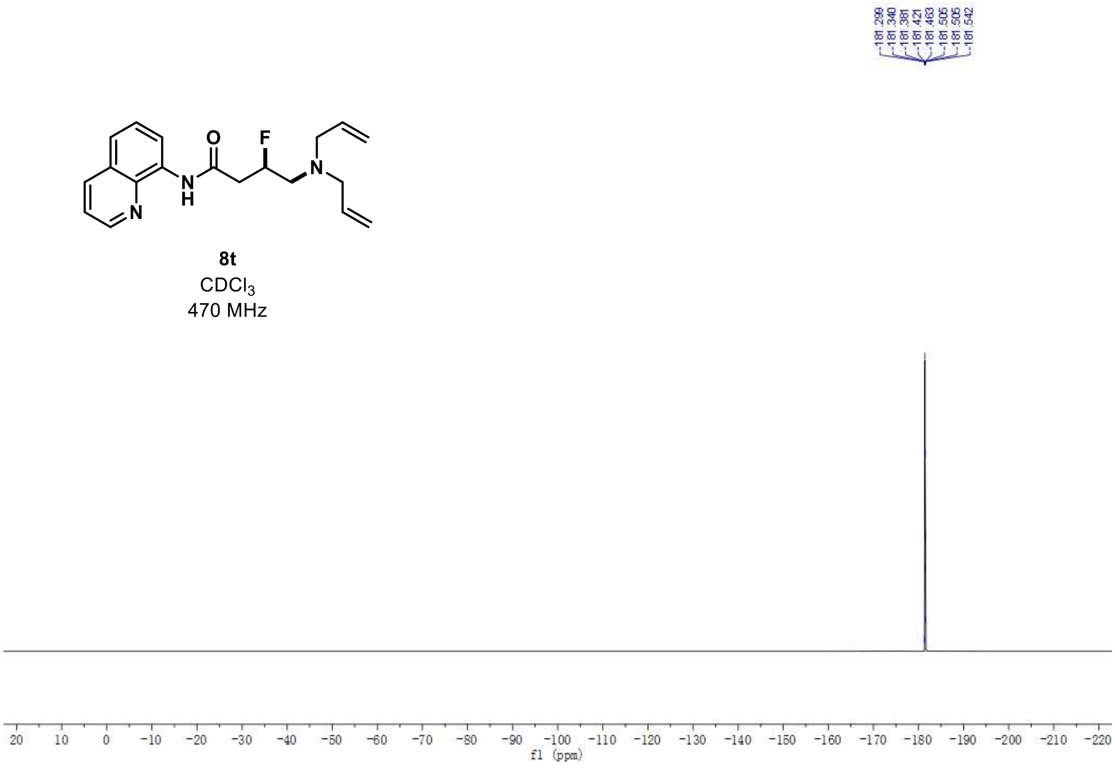

Figure S137. ^{19}F NMR Spectra of **8t**.

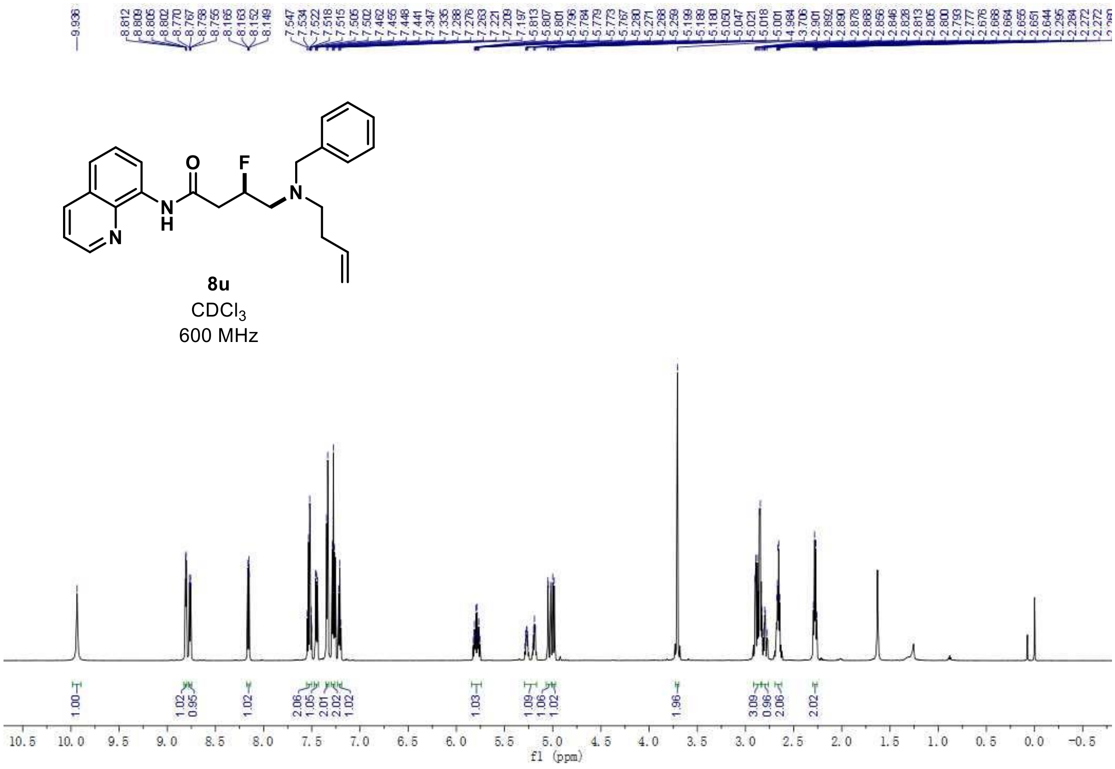

Figure S138. ^{1}H NMR Spectra of **8u**.

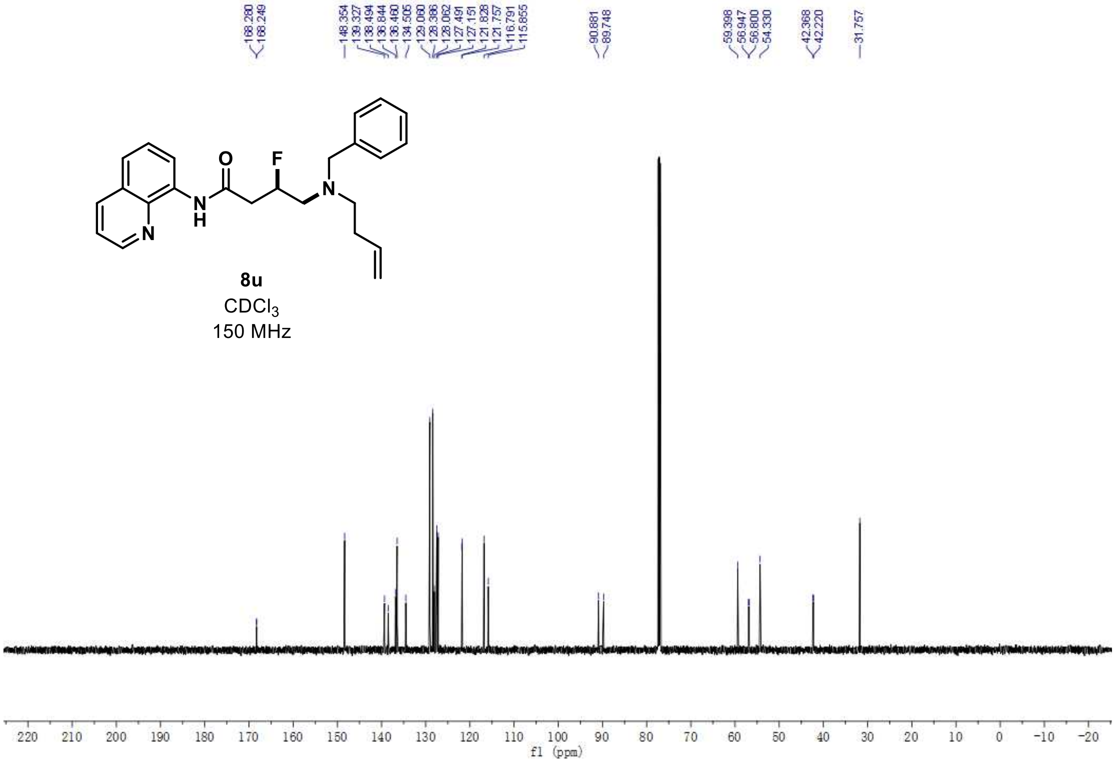

Figure S139. ^{13}C NMR Spectra of **8u**.

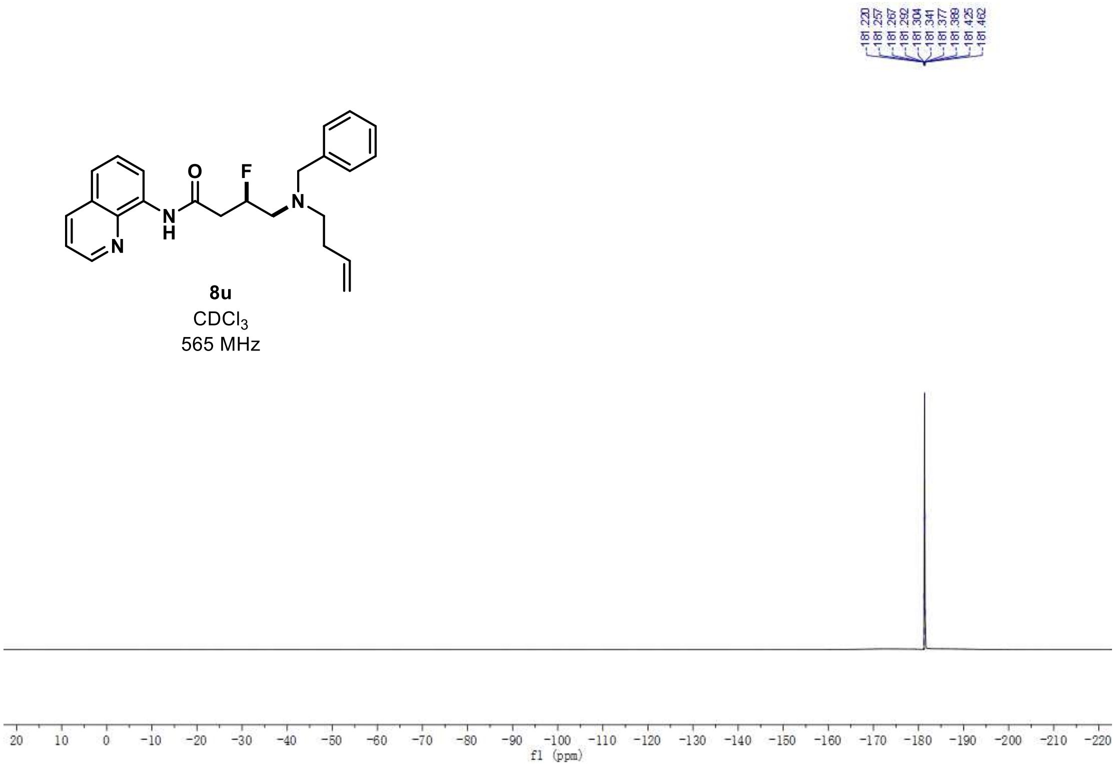

Figure S140. ^{19}F NMR Spectra of **8u**.

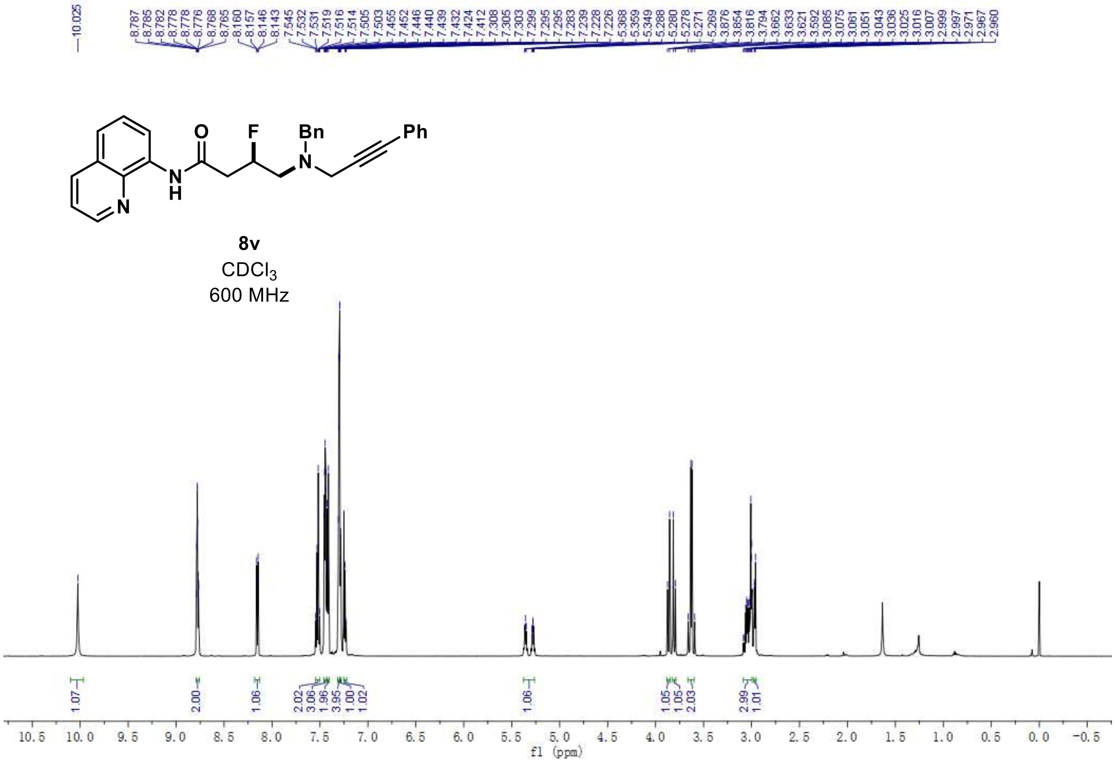

Figure S141. ^{1}H NMR Spectra of **8v**.

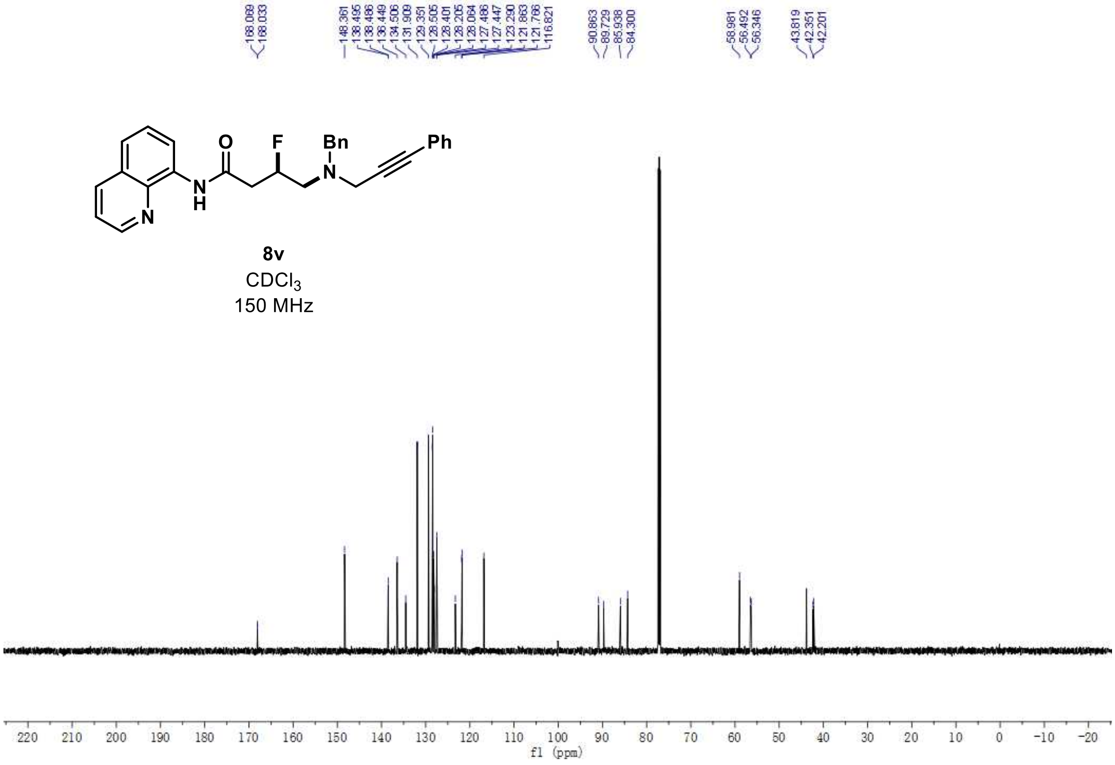

Figure S142. ^{13}C NMR Spectra of **8v**.

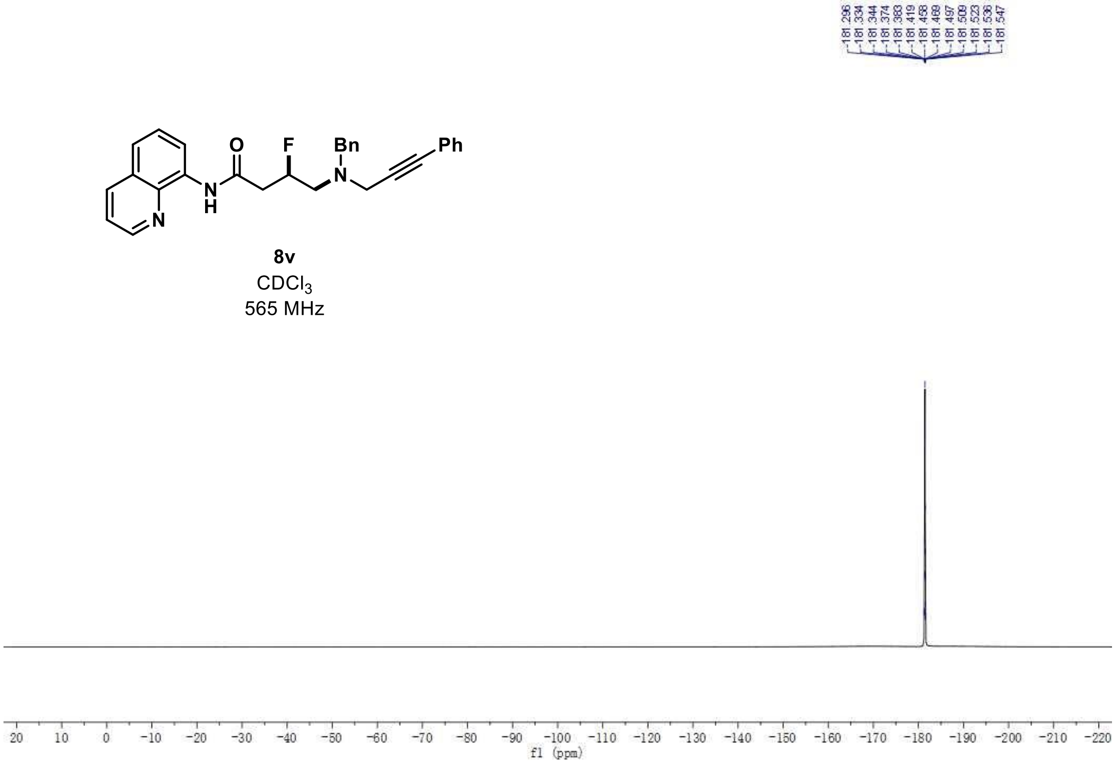

Figure S143. ^{19}F NMR Spectra of **8v**.

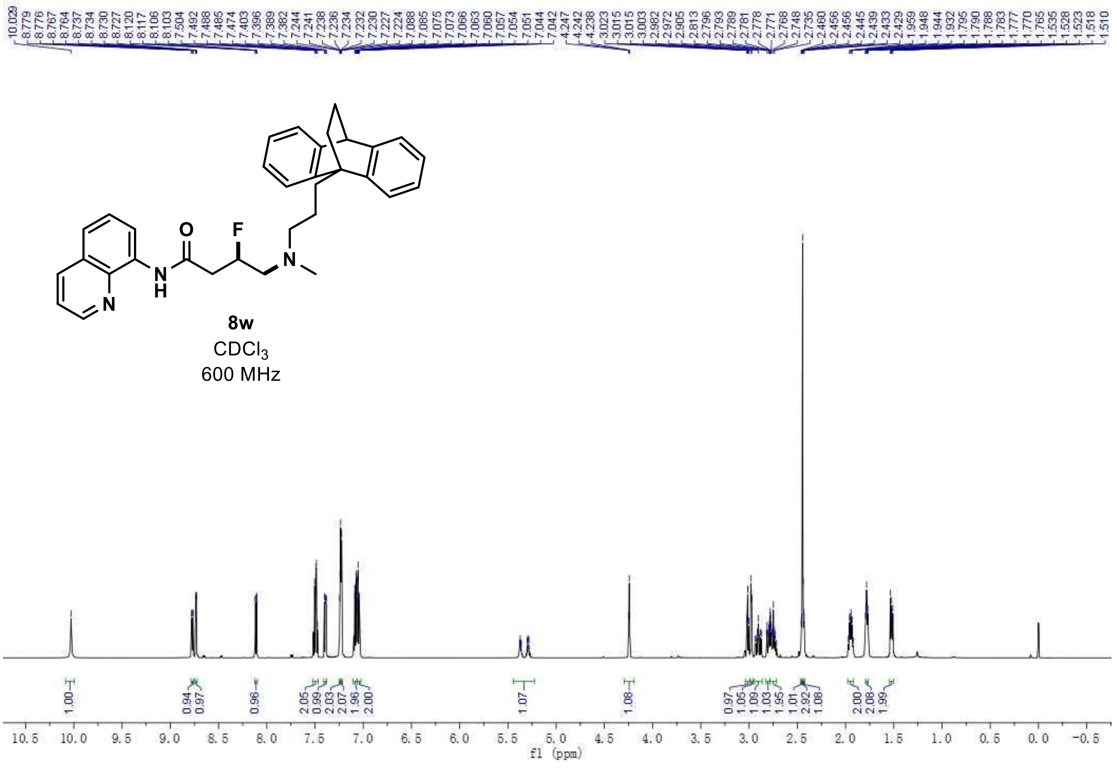

Figure S144. ^{1}H NMR Spectra of **8w**.

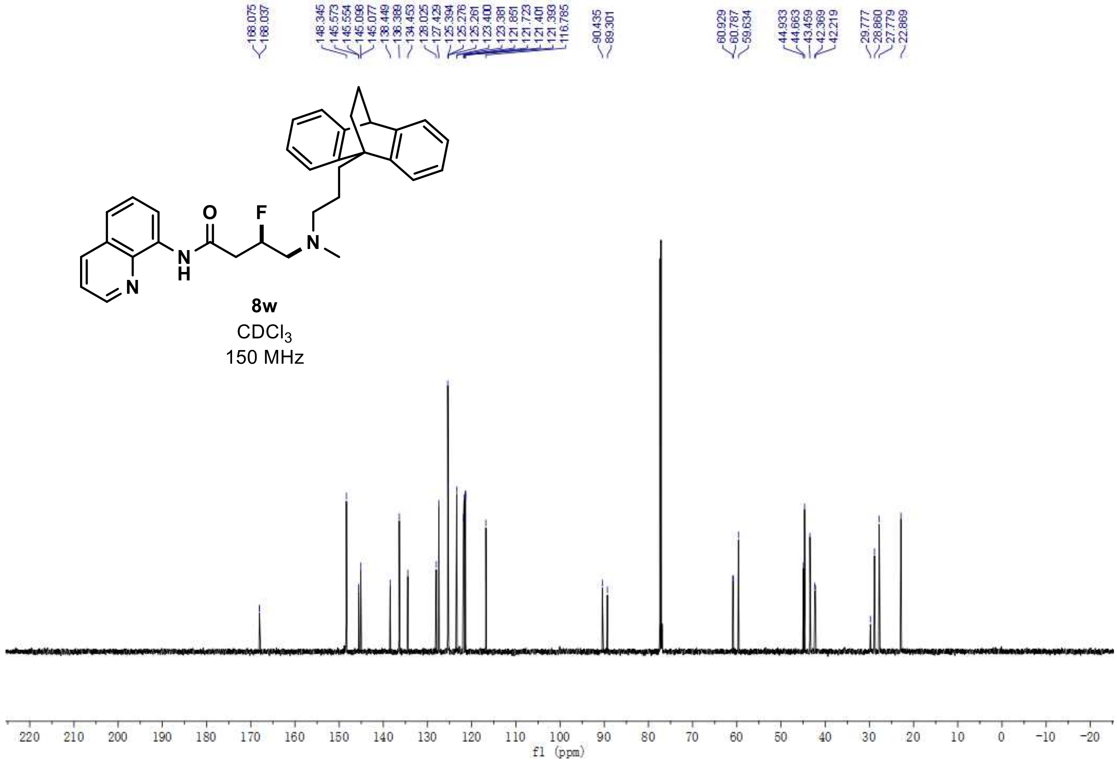

Figure S145. ^{13}C NMR Spectra of **8w**.

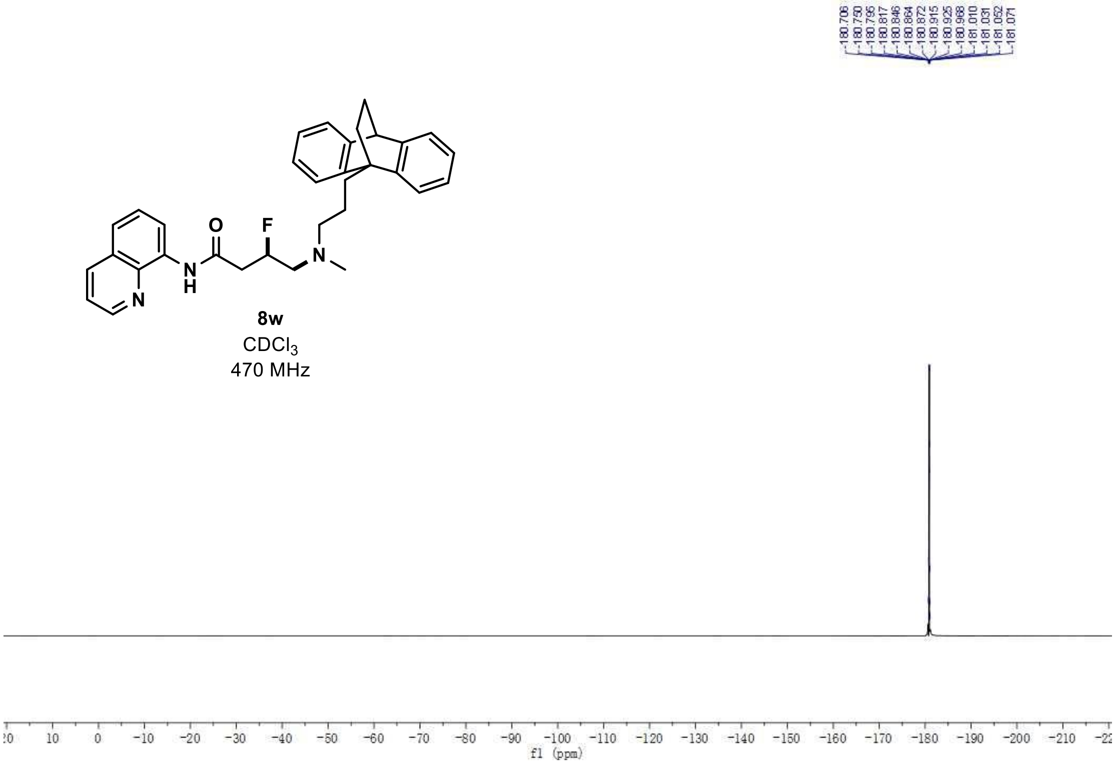

Figure S146. ^{19}F NMR Spectra of **8w**.

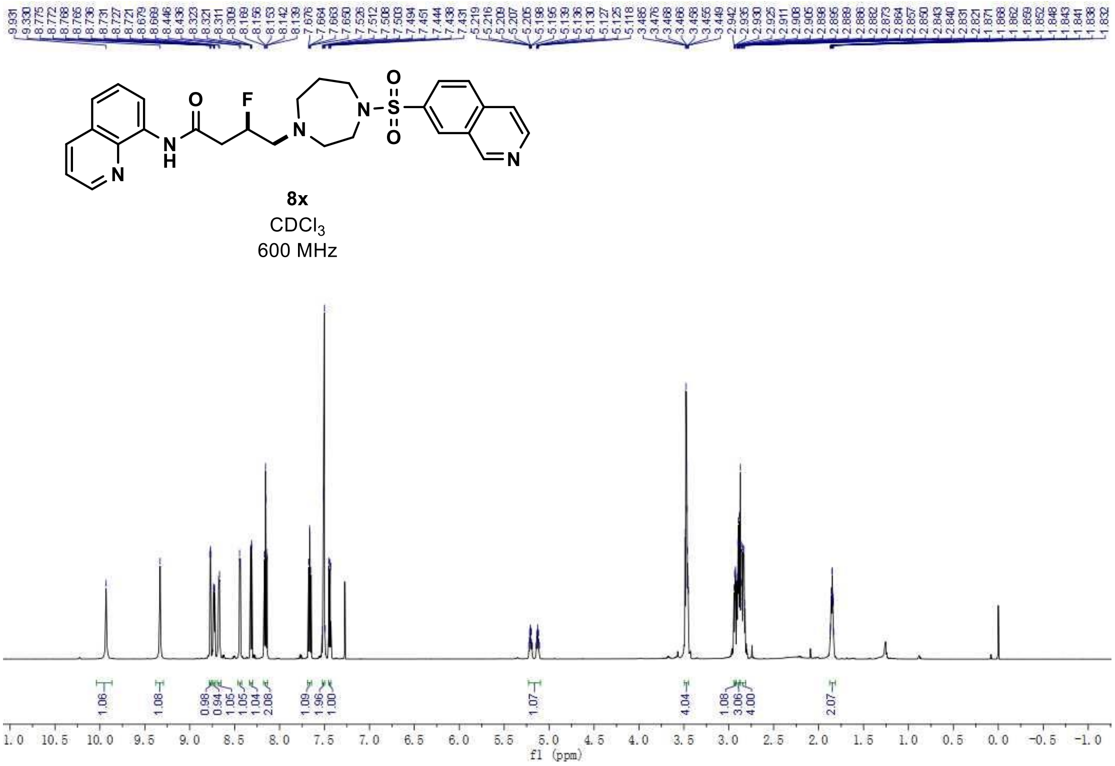

Figure S147. ^{1}H NMR Spectra of **8x**.

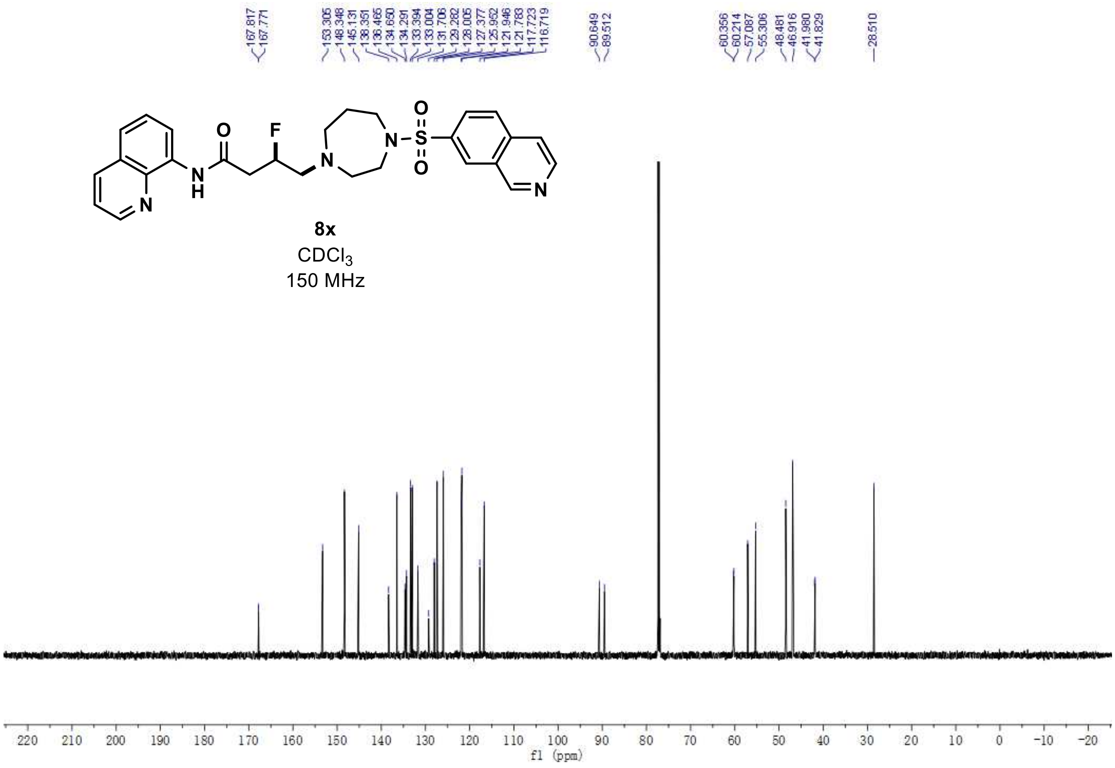

Figure S148. ^{13}C NMR Spectra of **8x**.

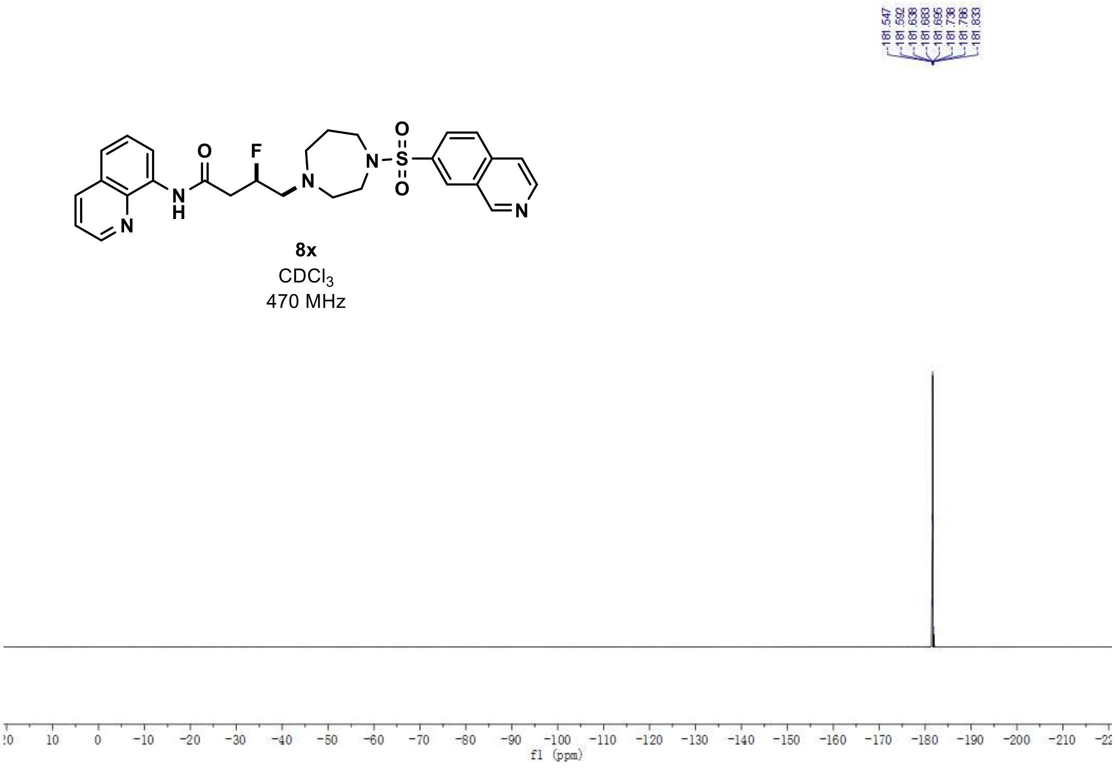

Figure S149. ^{19}F NMR Spectra of **8x**.

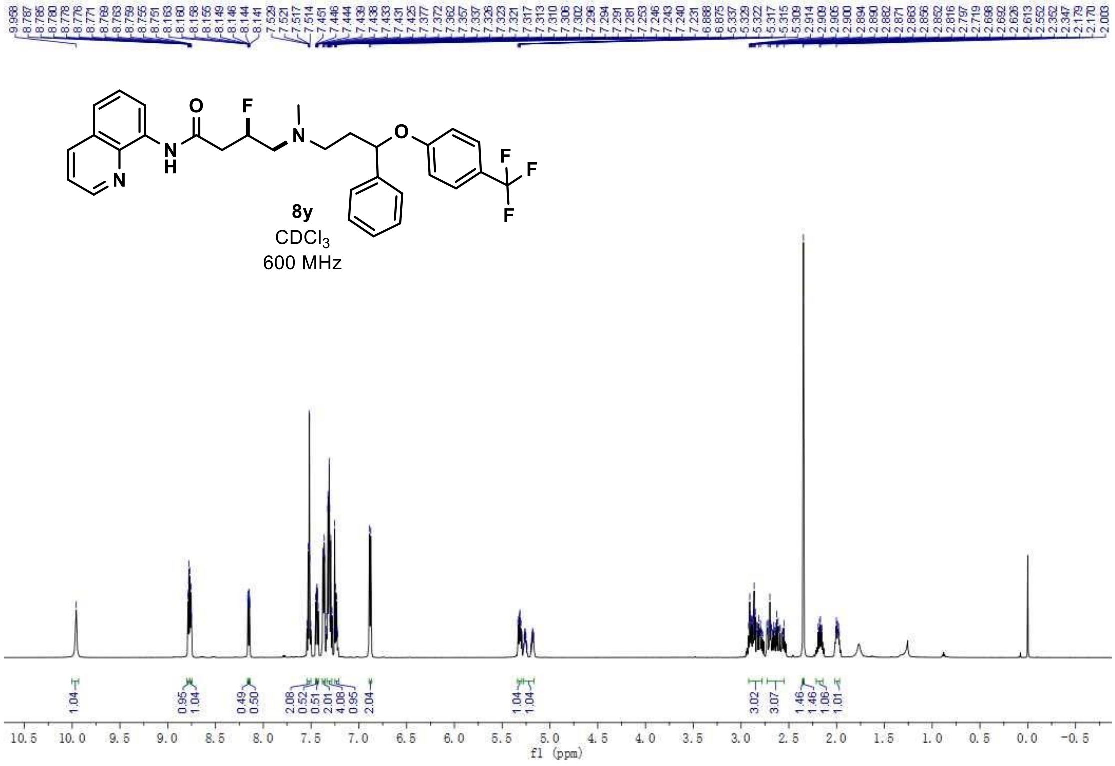

Figure S150. ^{1}H NMR Spectra of **8y**.

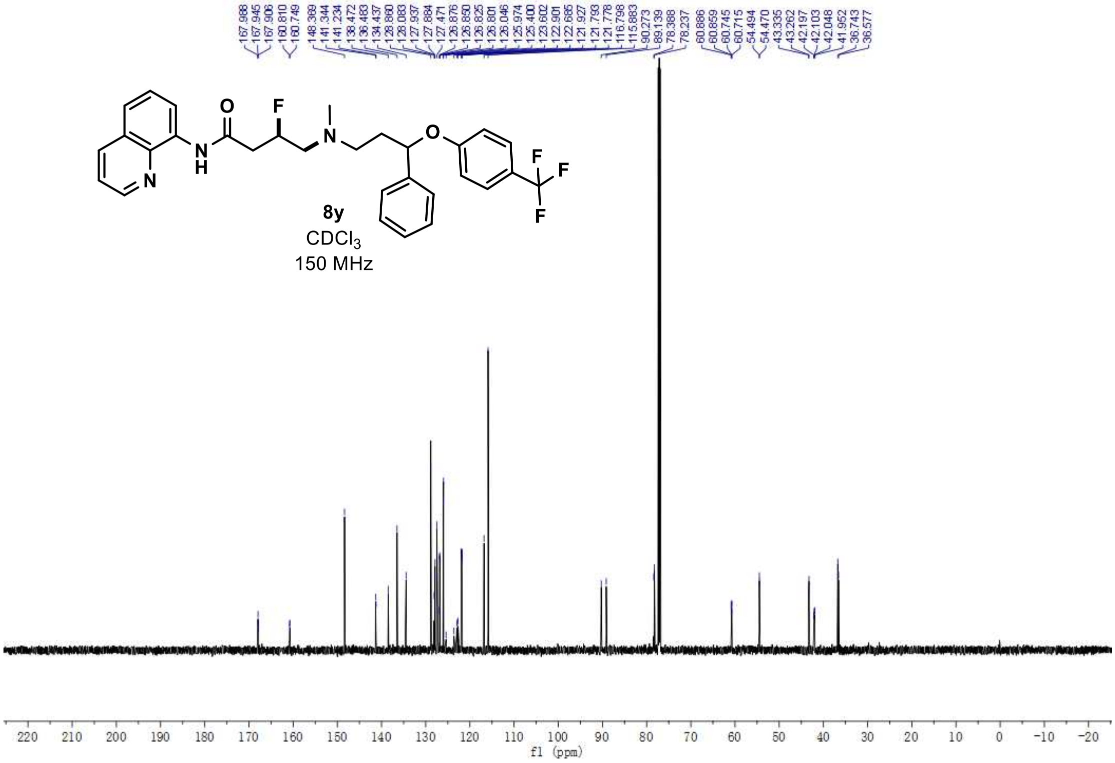

Figure S151. ^{13}C NMR Spectra of **8y**.

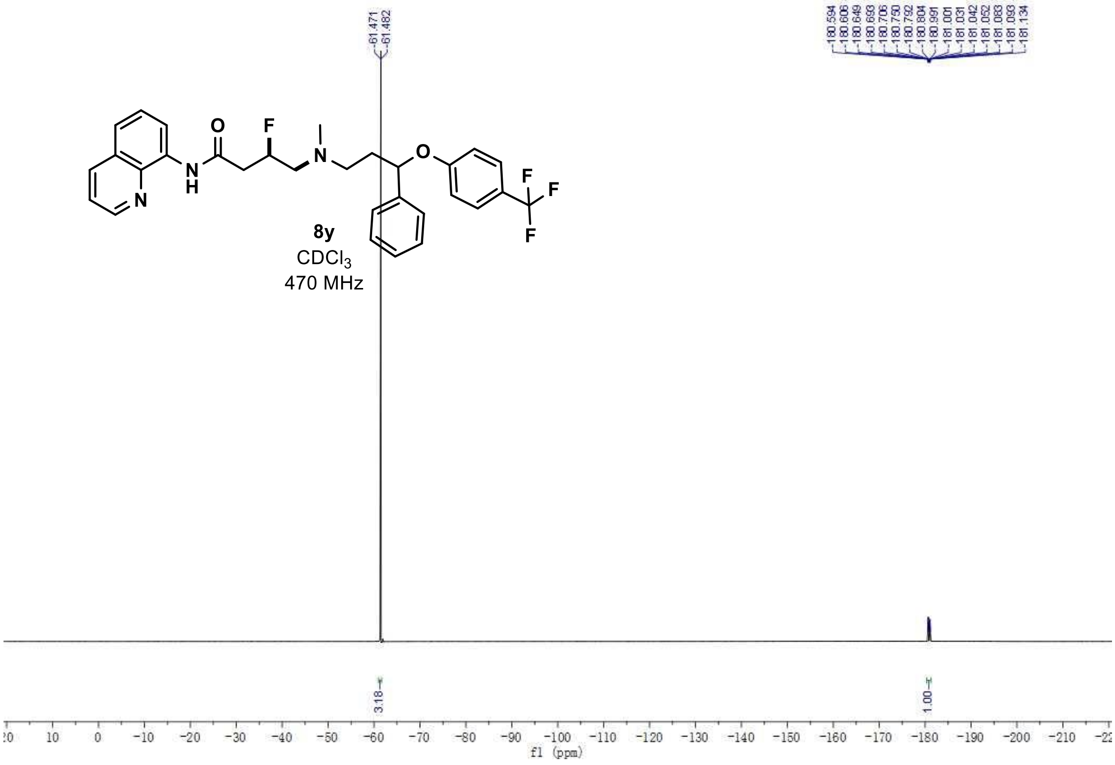

Figure S152. ^{19}F NMR Spectra of **8y**.

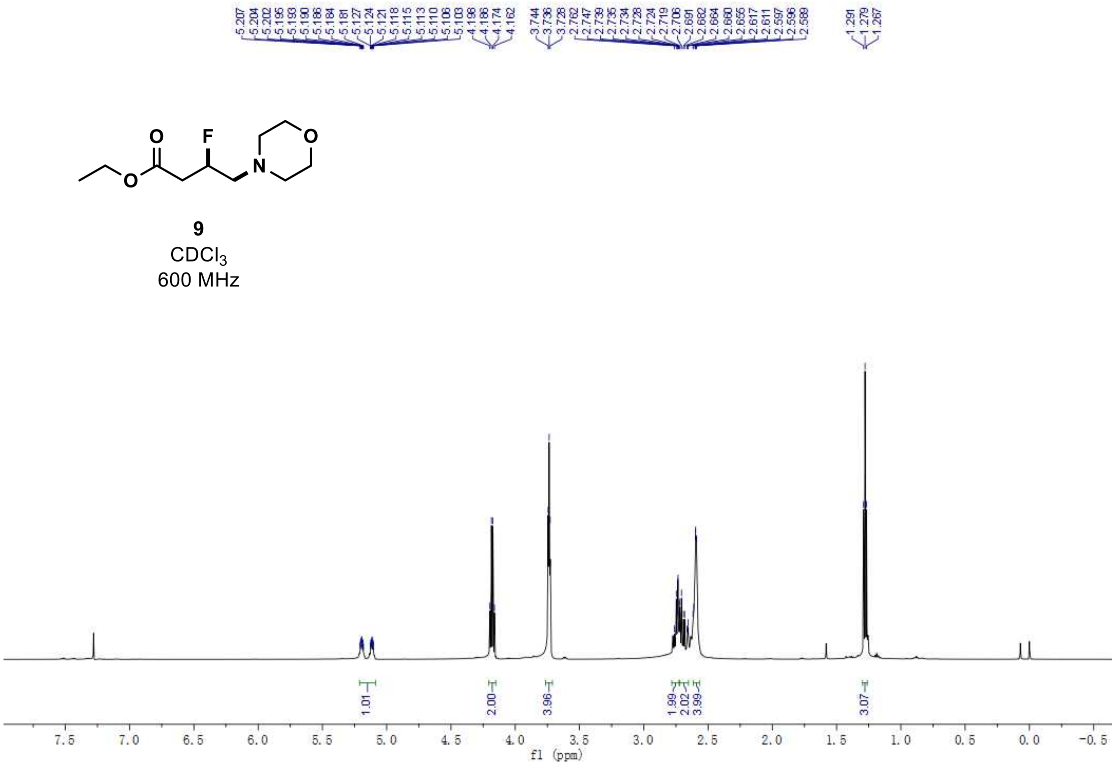

Figure S153. ^{1}H NMR Spectra of **9**.

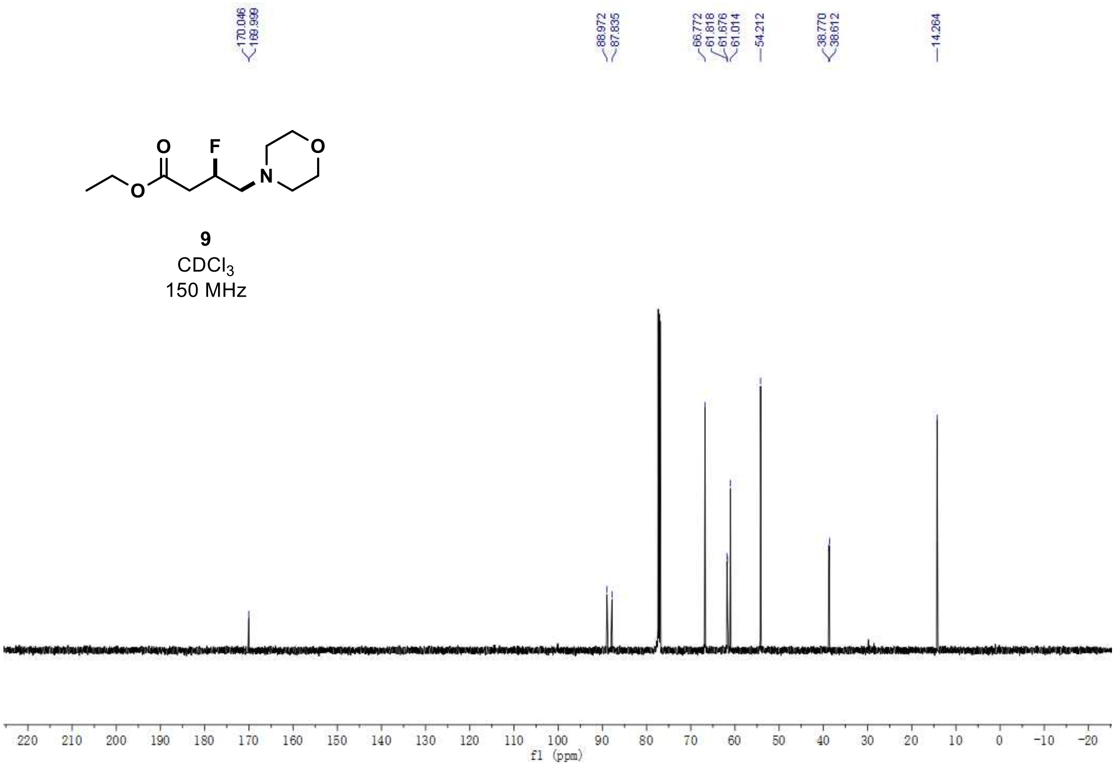

Figure S154. ^{13}C NMR Spectra of **9**.

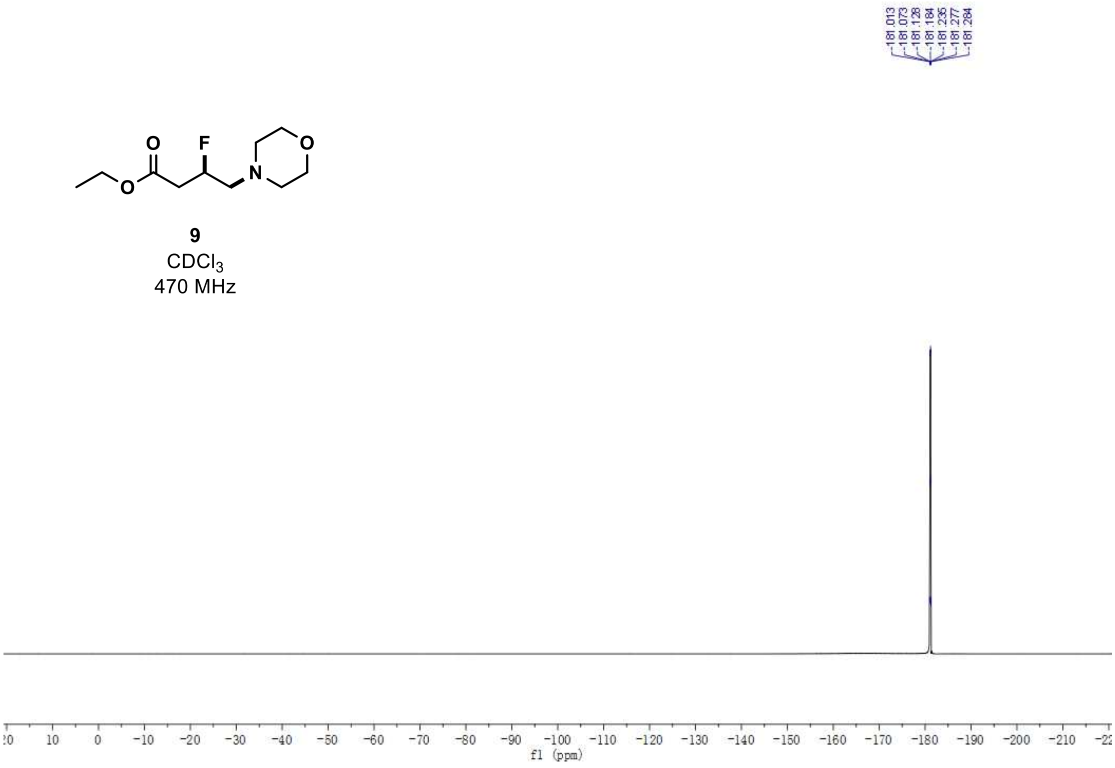

Figure S155. ^{19}F NMR Spectra of **9**.

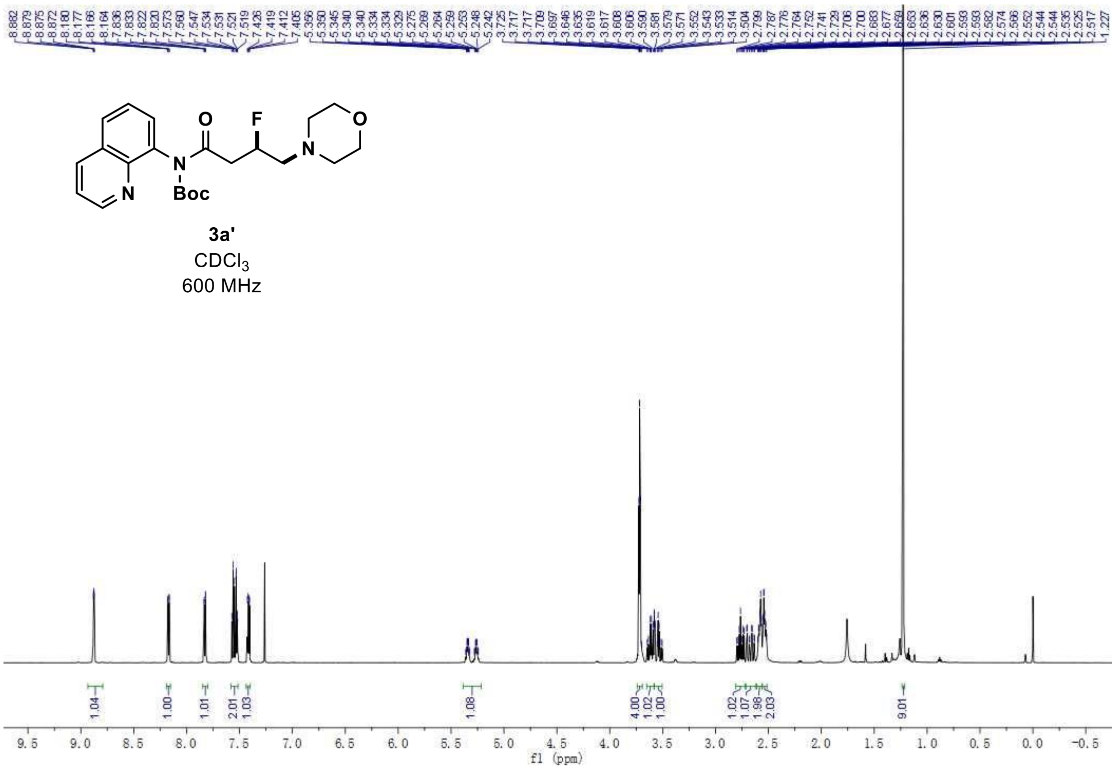

Figure S156. ^{1}H NMR Spectra of **3a’**.

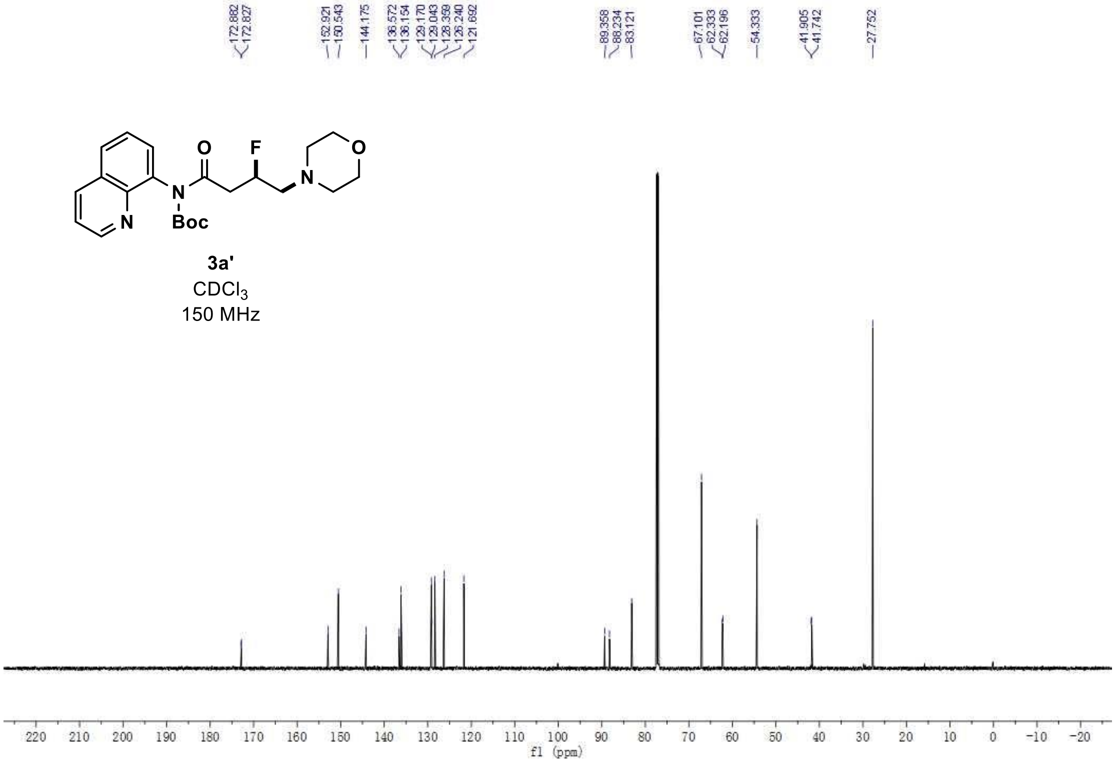

Figure S157. ^{13}C NMR Spectra of **3a’**.

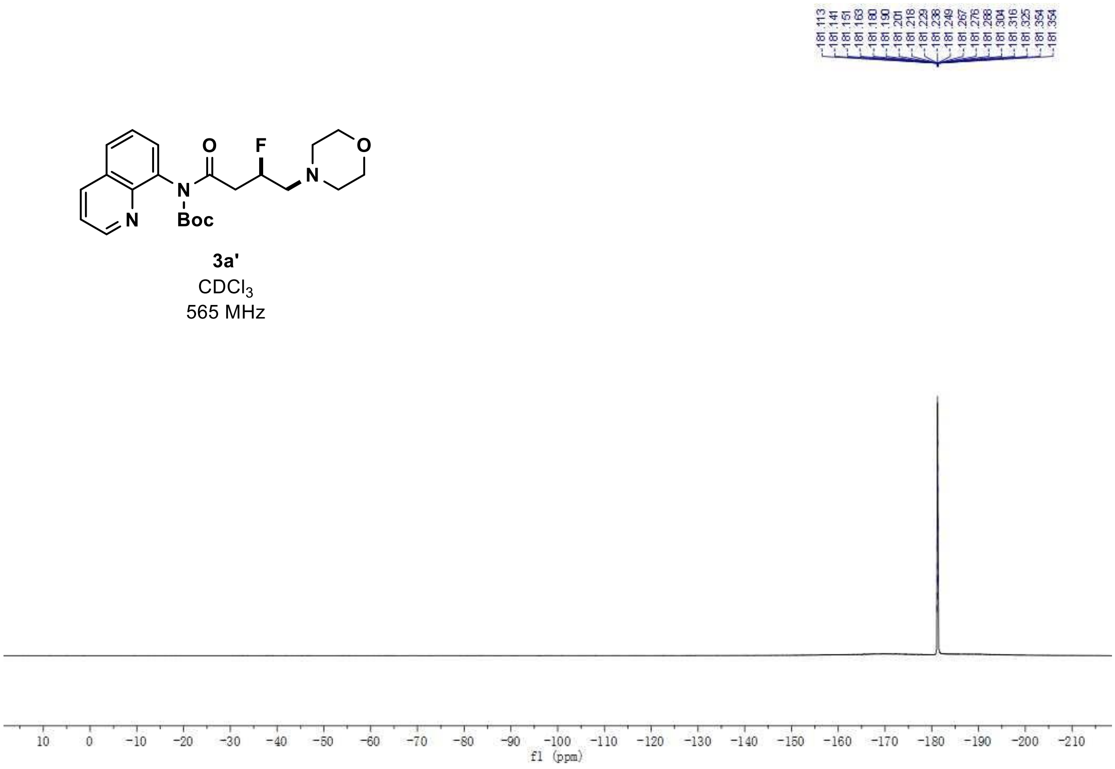

Figure S158. ^{19}F NMR Spectra of **3a'**.

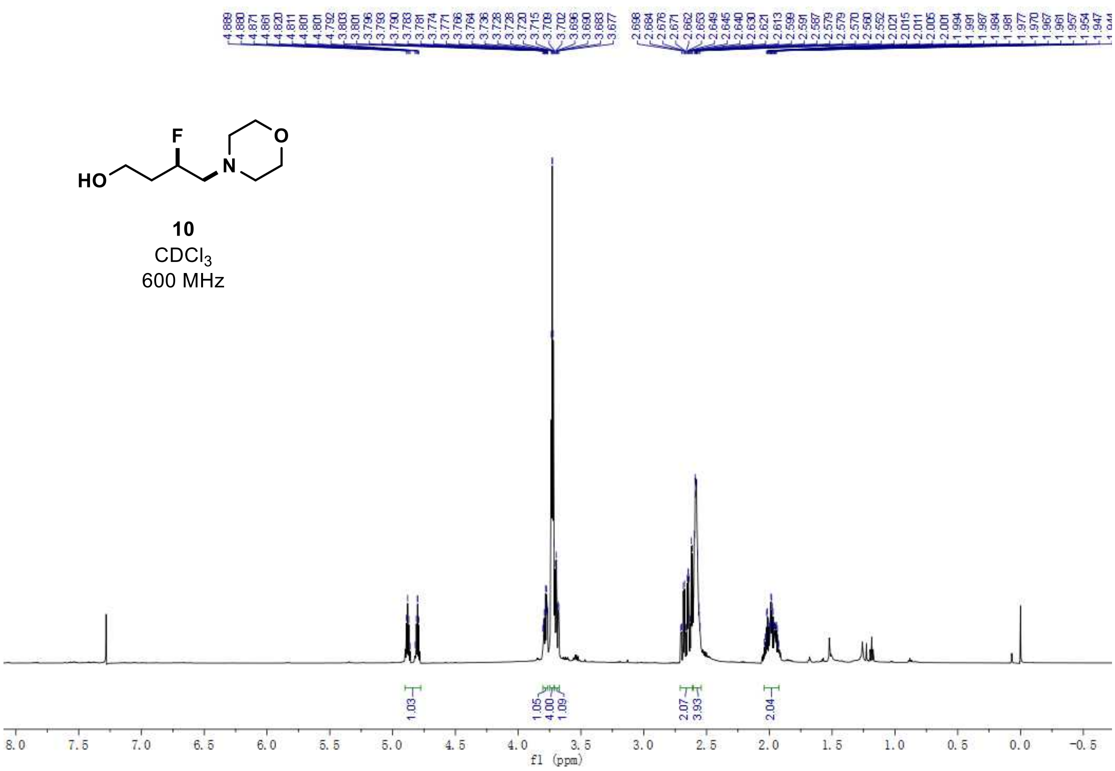

Figure S159. ^{1}H NMR Spectra of **10**.

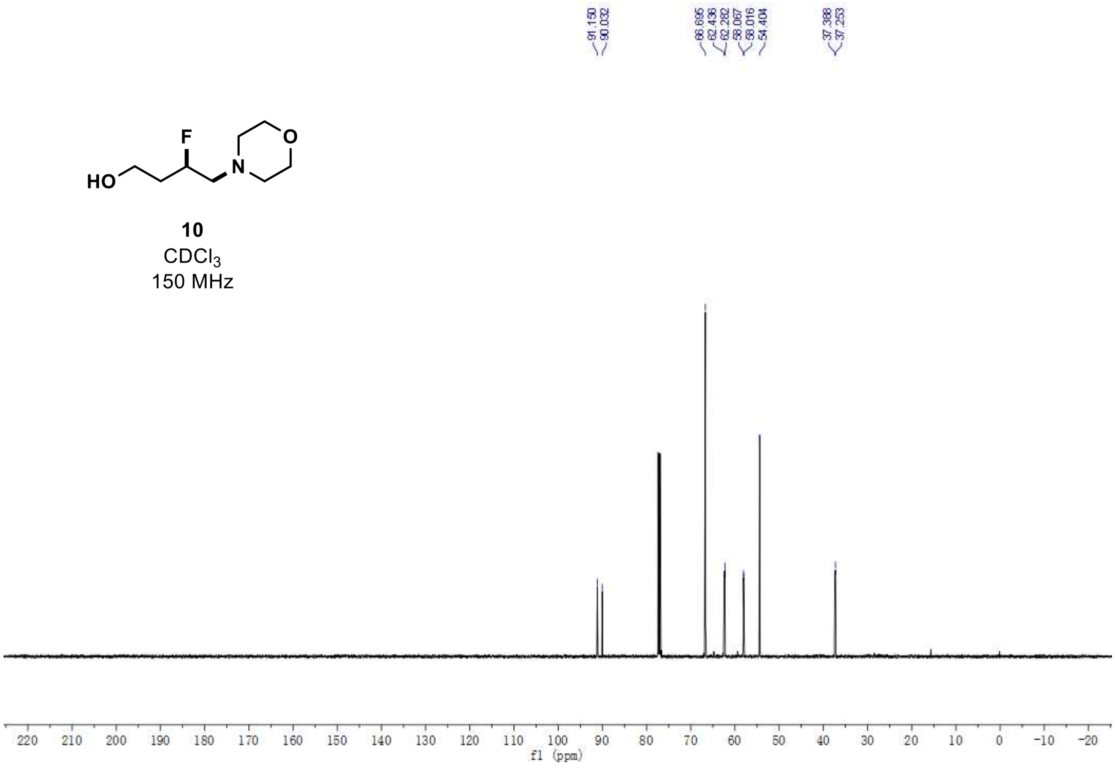

Figure S160. ^{13}C NMR Spectra of **10**.

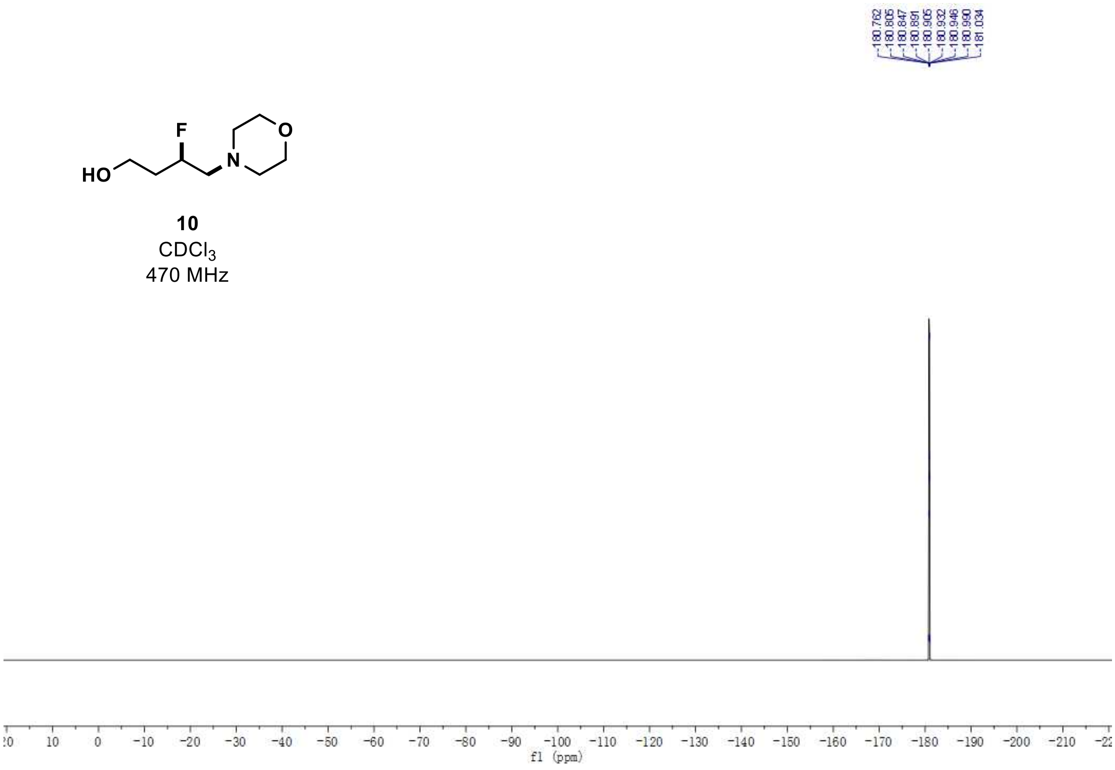

Figure S161. ^{19}F NMR Spectra of **10**.

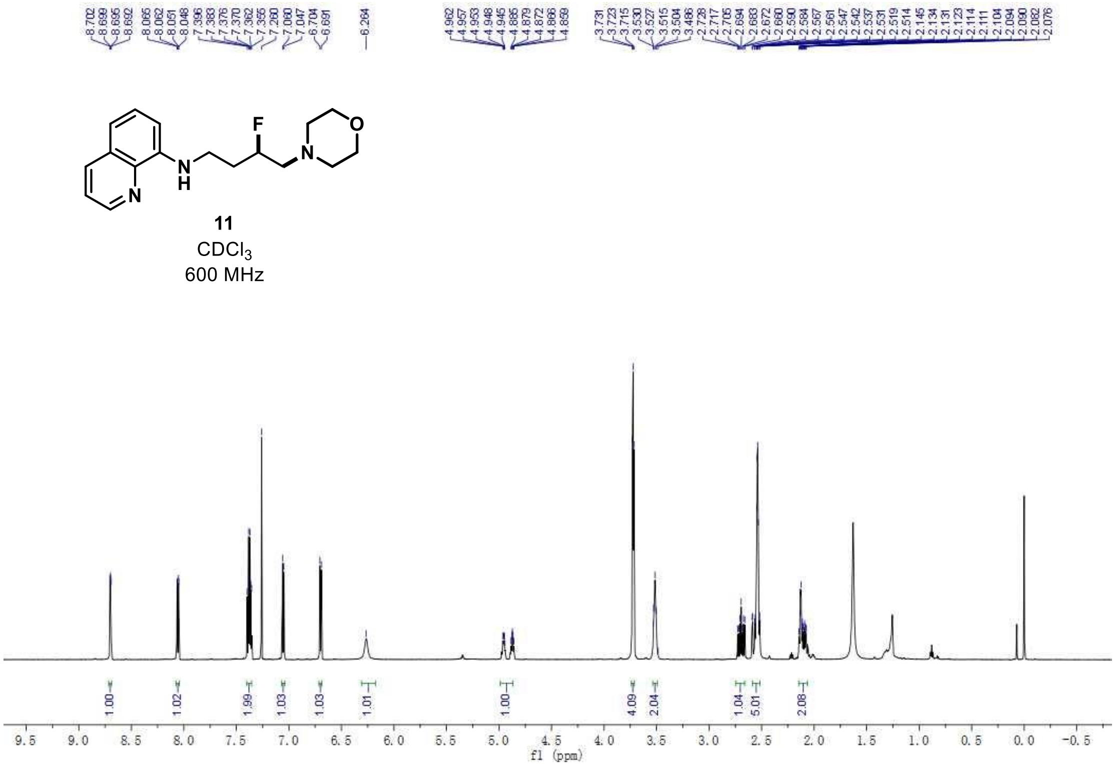

Figure S162. ^{1}H NMR Spectra of **11**.

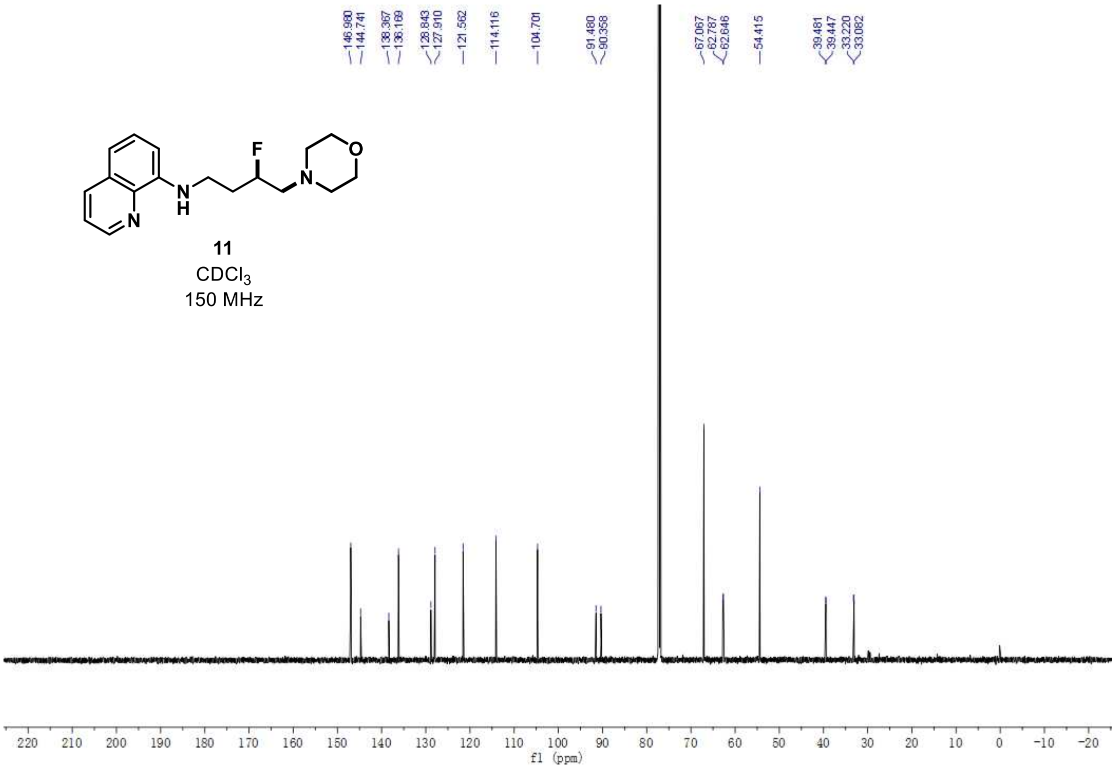

Figure S163. ^{13}C NMR Spectra of **11**.

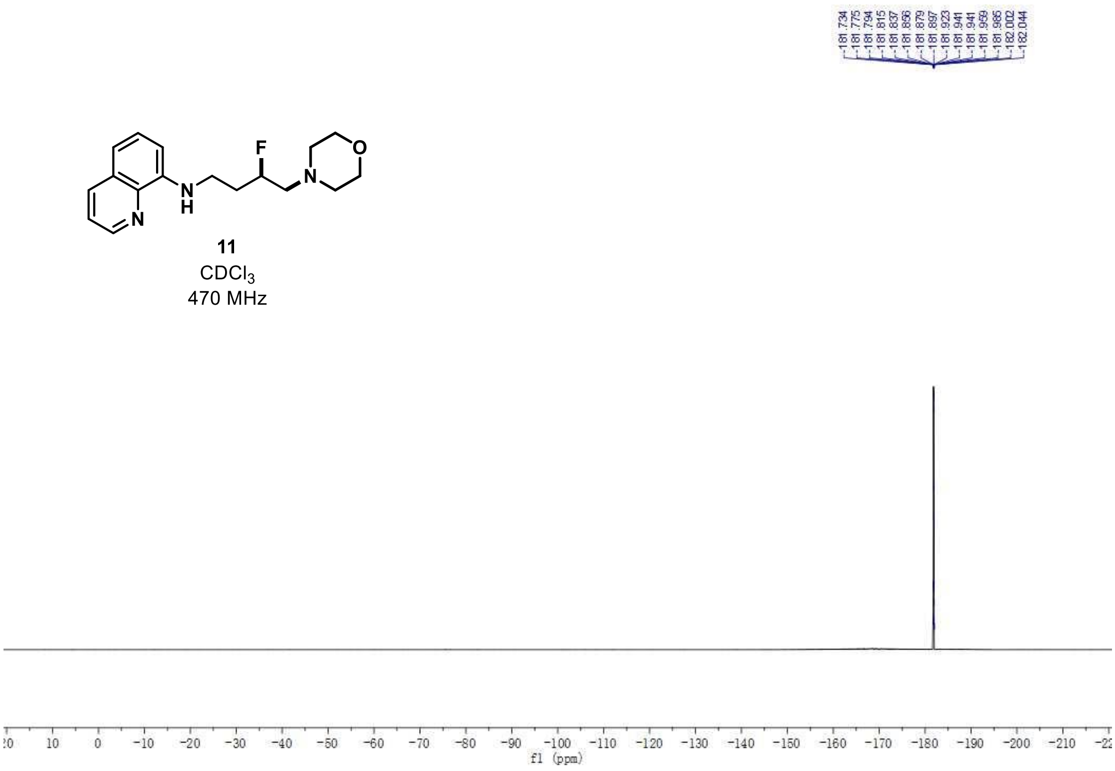

Figure S164. ^{19}F NMR Spectra of **11**.

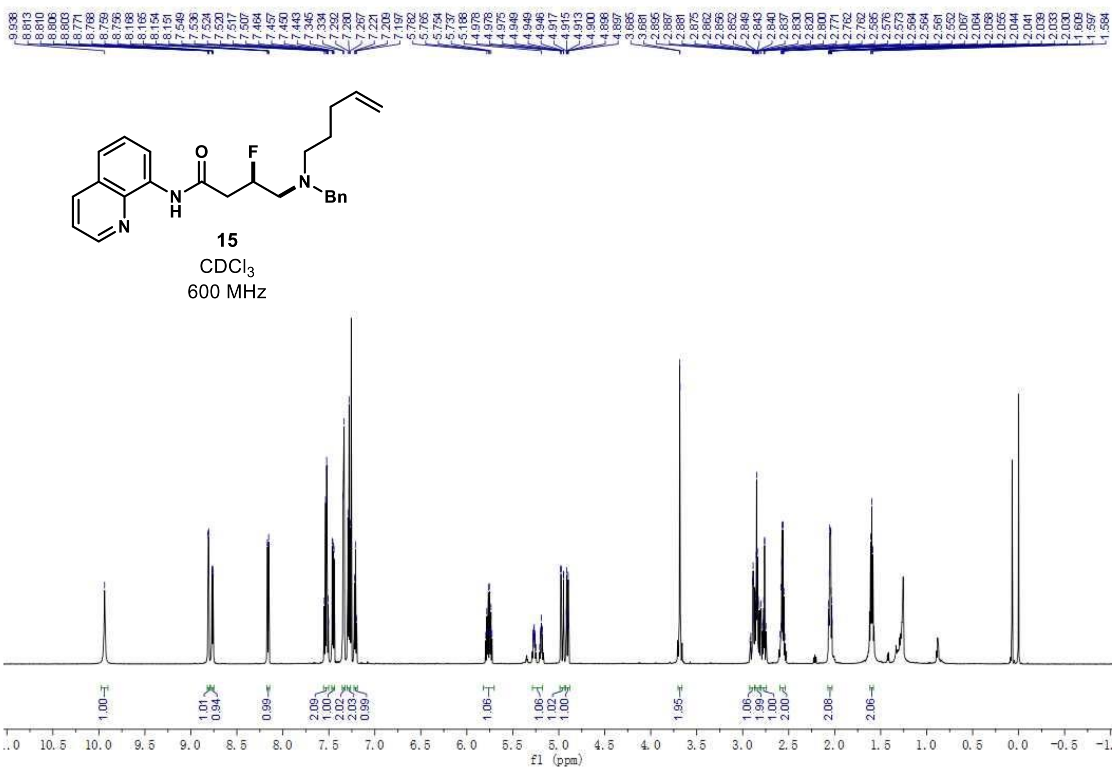

Figure S165. ^{1}H NMR Spectra of **15**.

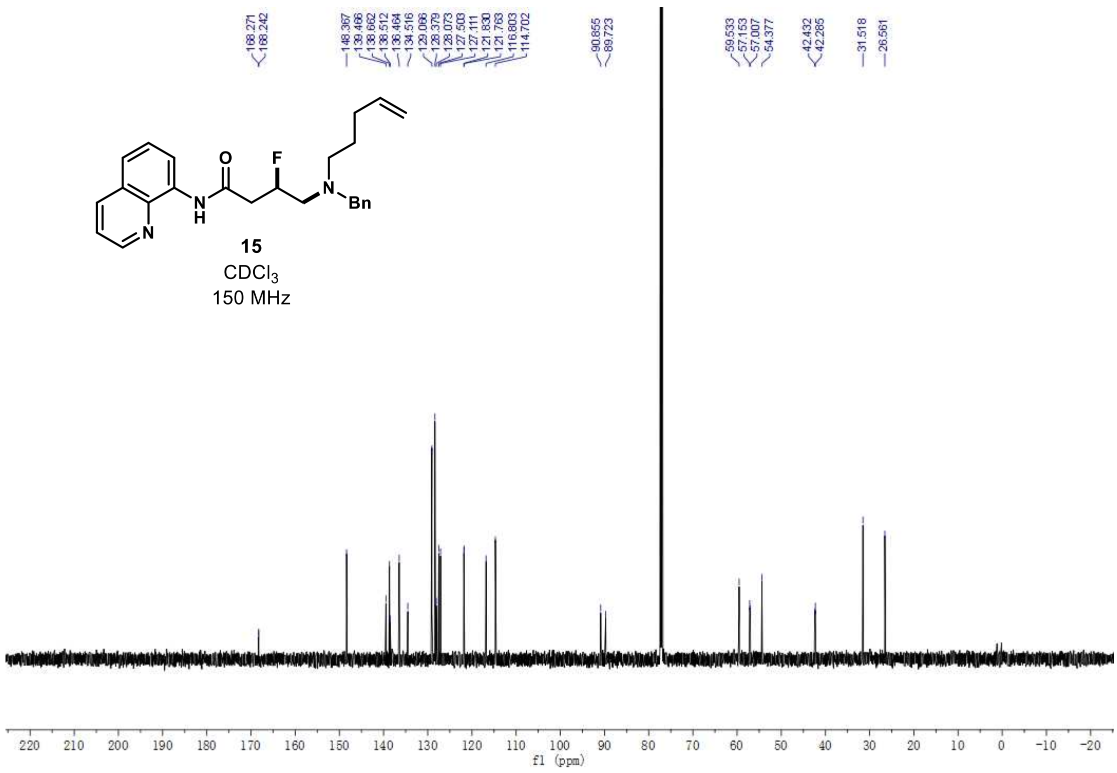

Figure S166. ^{13}C NMR Spectra of **15**.

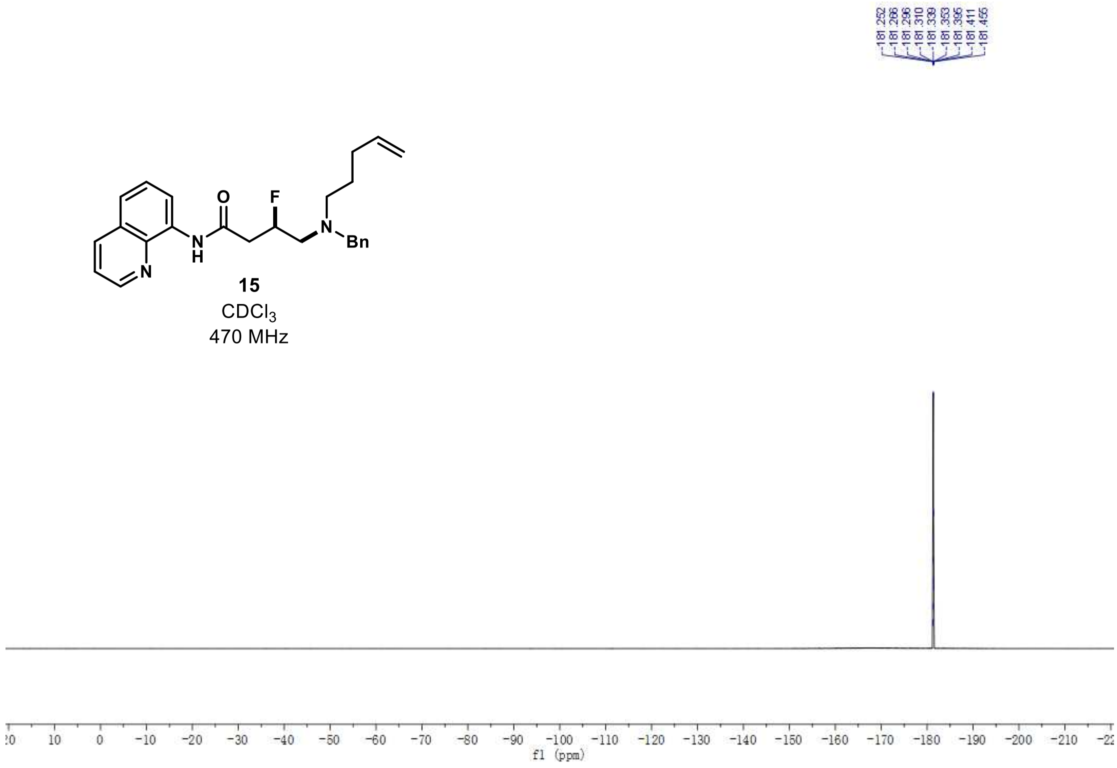

Figure S167. ^{19}F NMR Spectra of **15**.

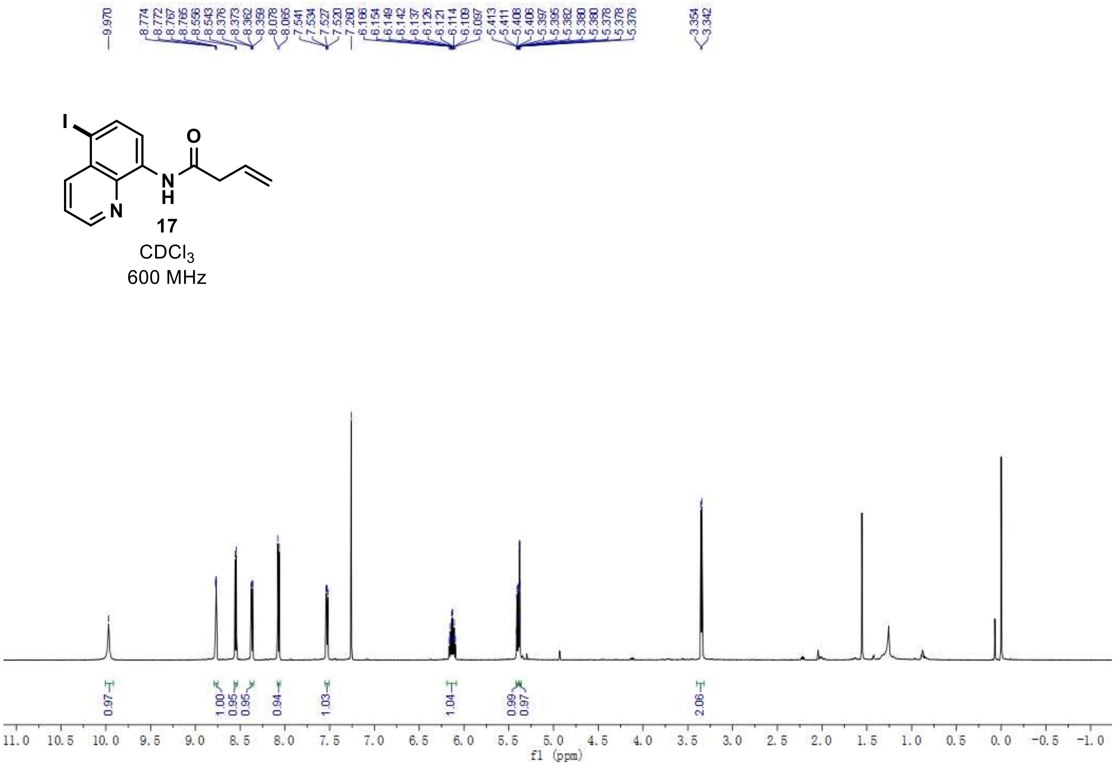

Figure S168. ^{1}H NMR Spectra of **17**.

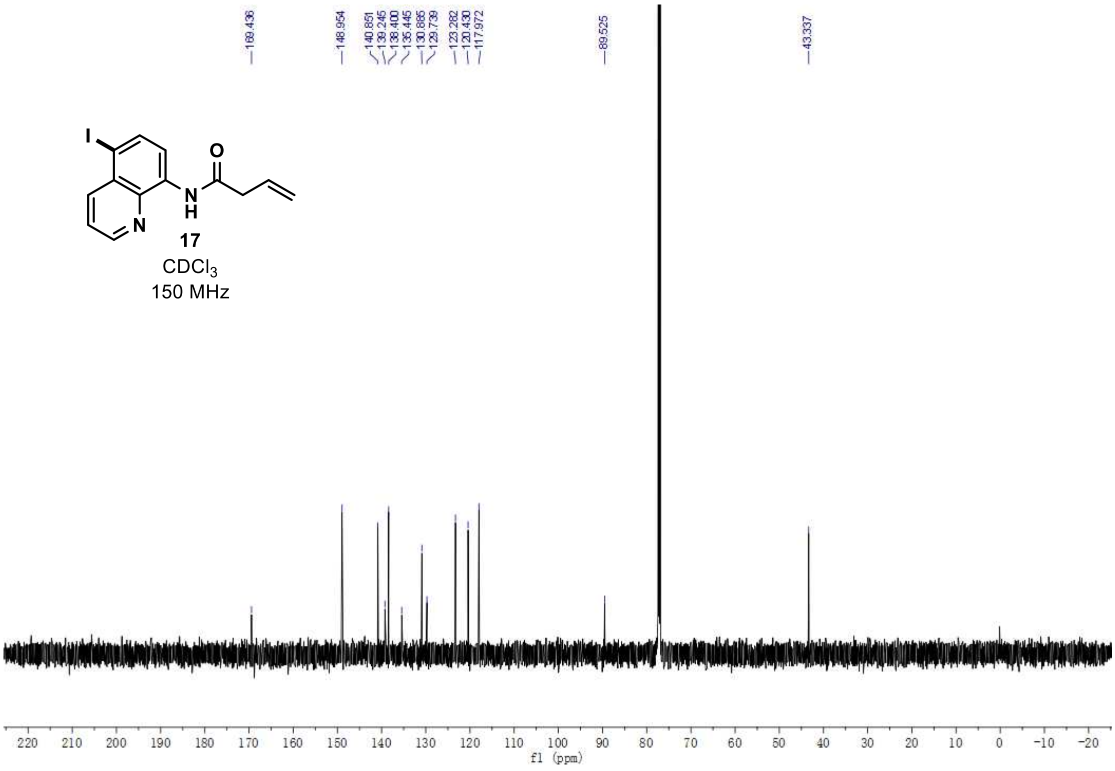

Figure S169. ^{13}C NMR Spectra of **17**.

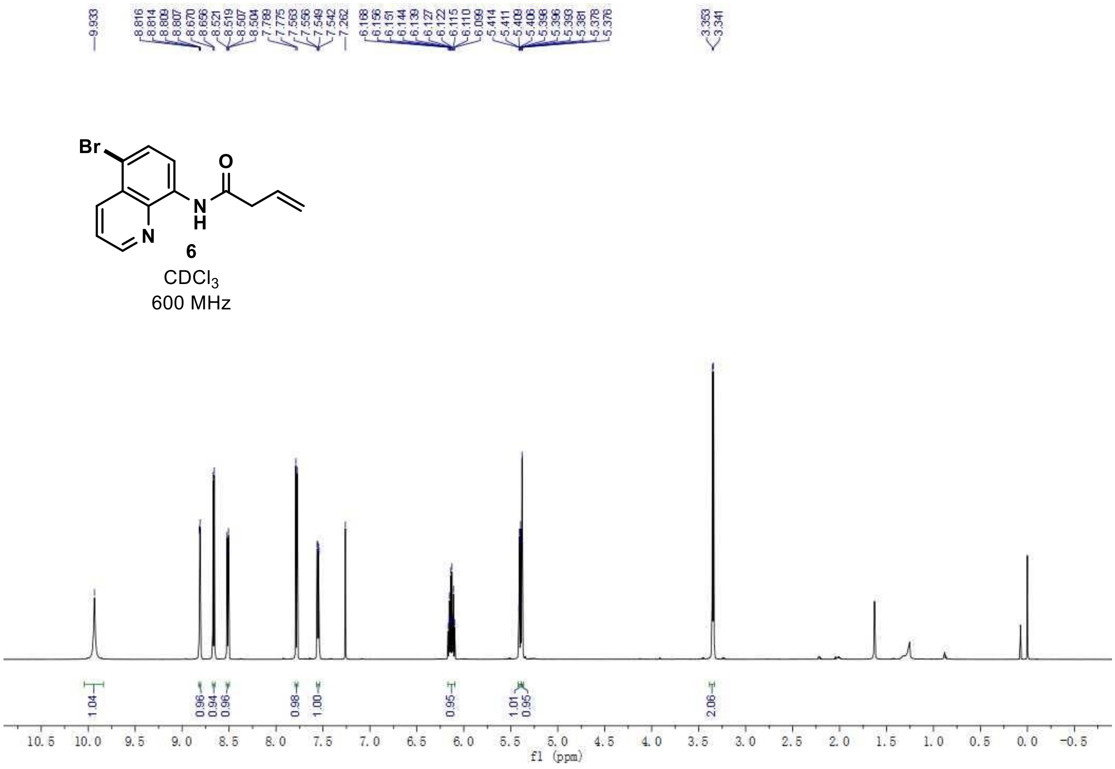

Figure S170. ^{1}H NMR Spectra of **6**.

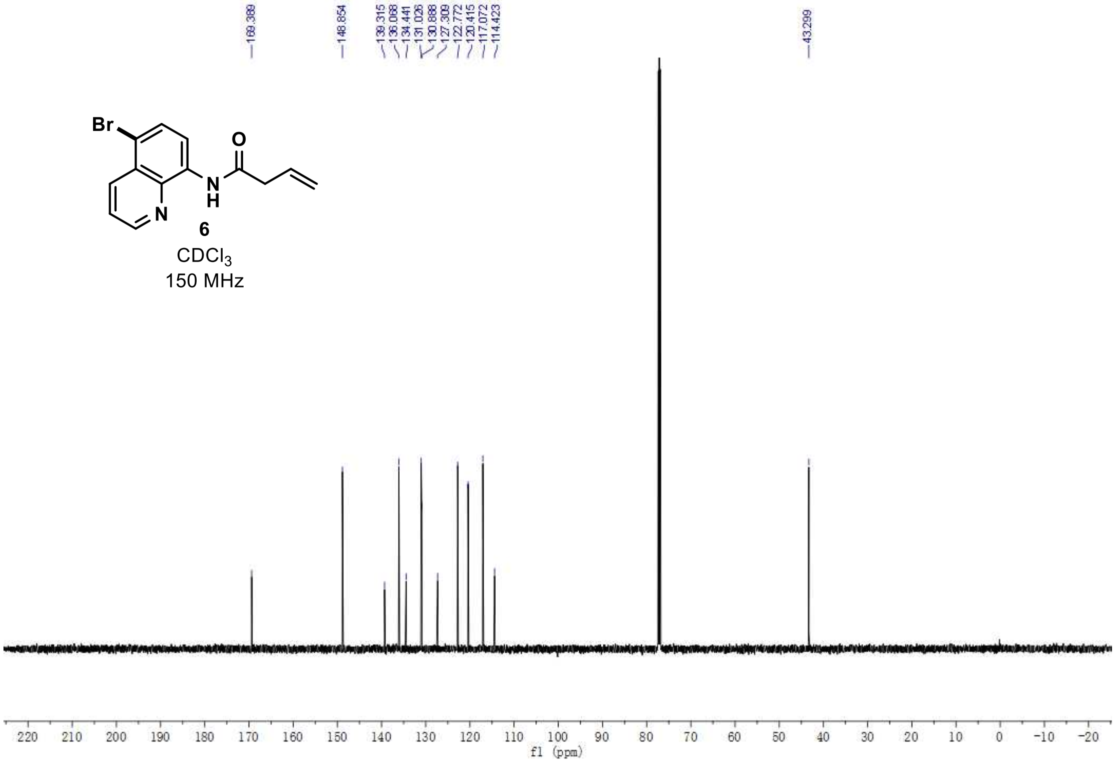

Figure S171. ^{13}C NMR Spectra of **6**.

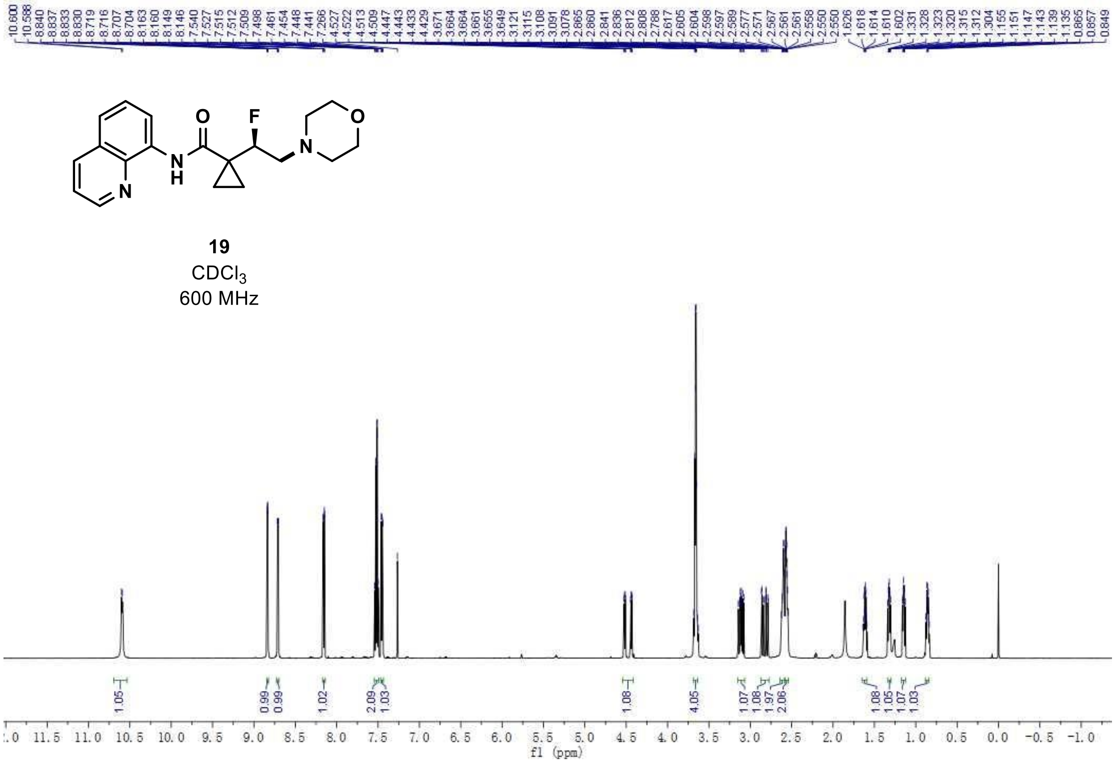

Figure S172. ^{1}H NMR Spectra of **19**.

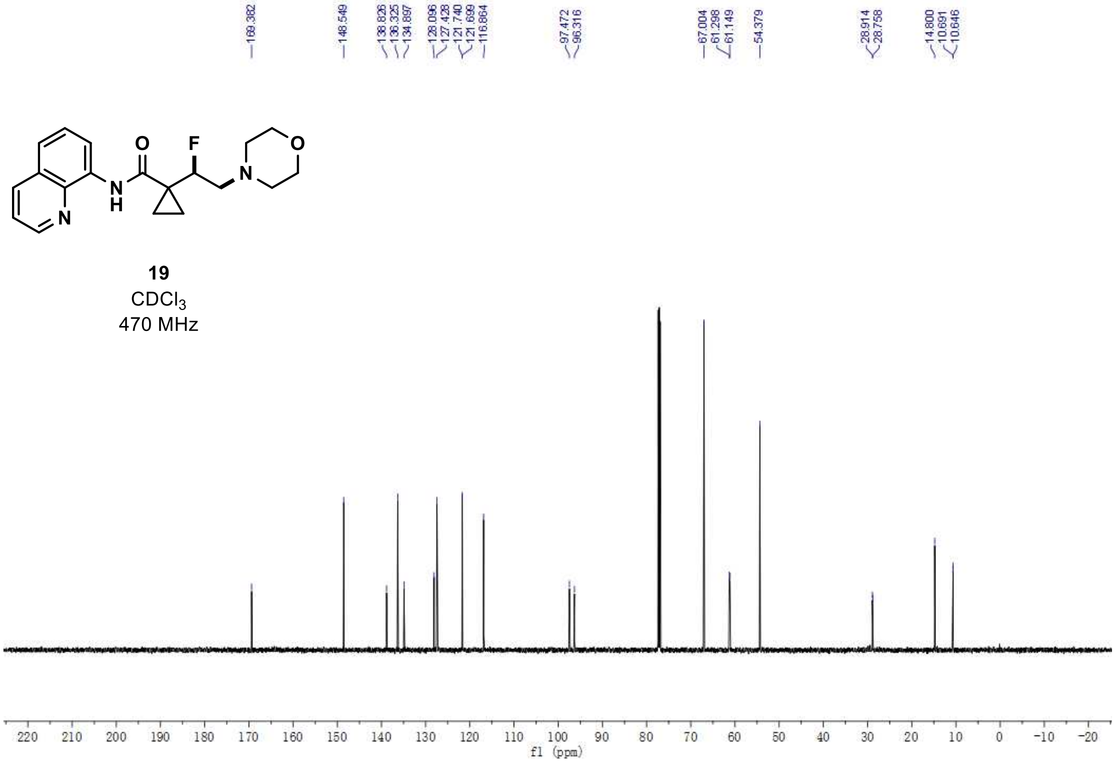

Figure S173. ^{13}C NMR Spectra of **19**.

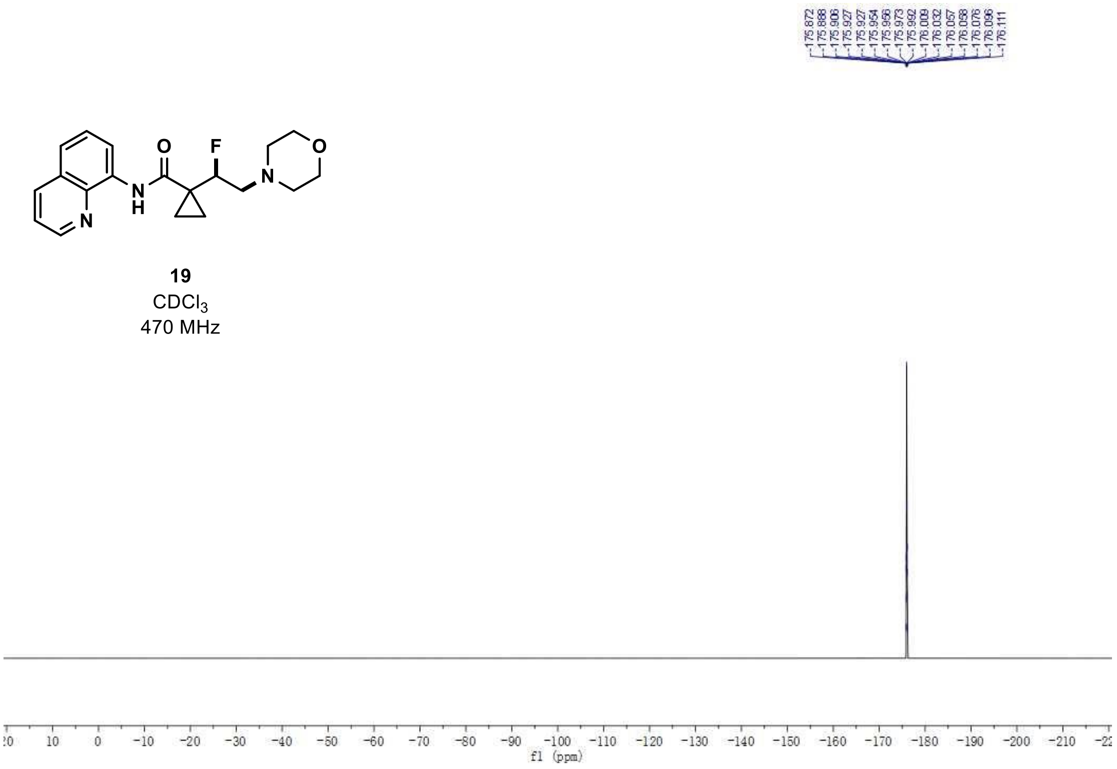

Figure S174. ^{19}F NMR Spectra of **19**.

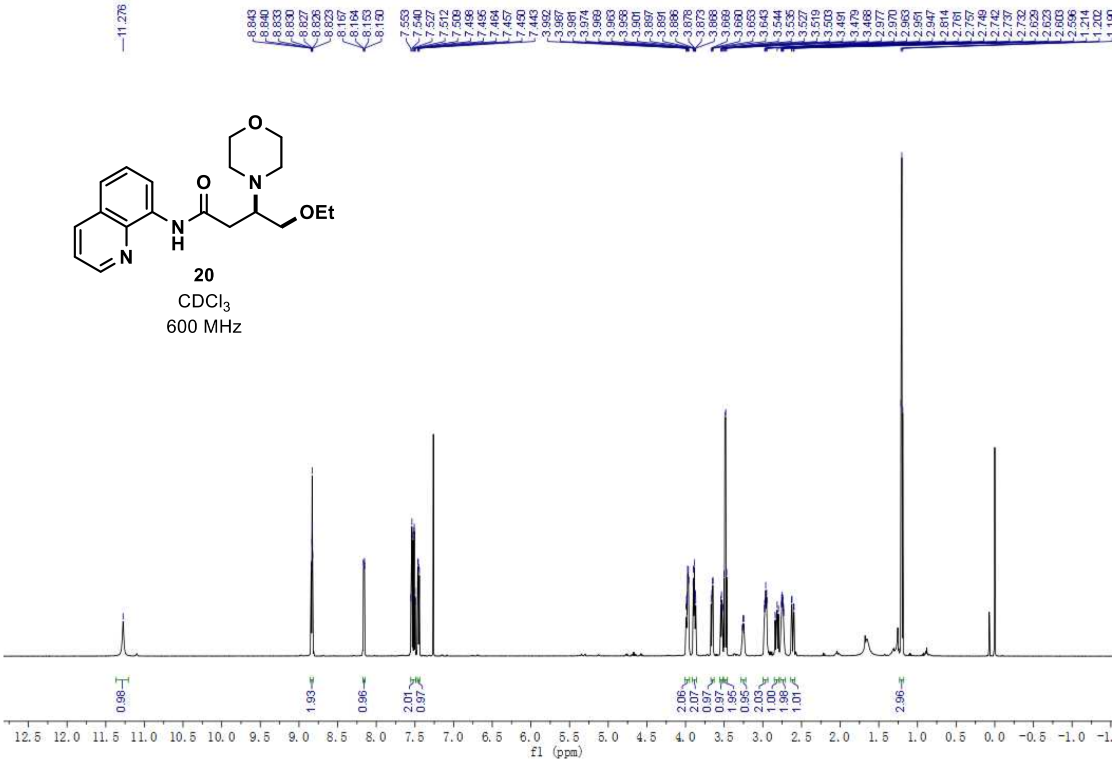

Figure S175. ^{1}H NMR Spectra of **20**.

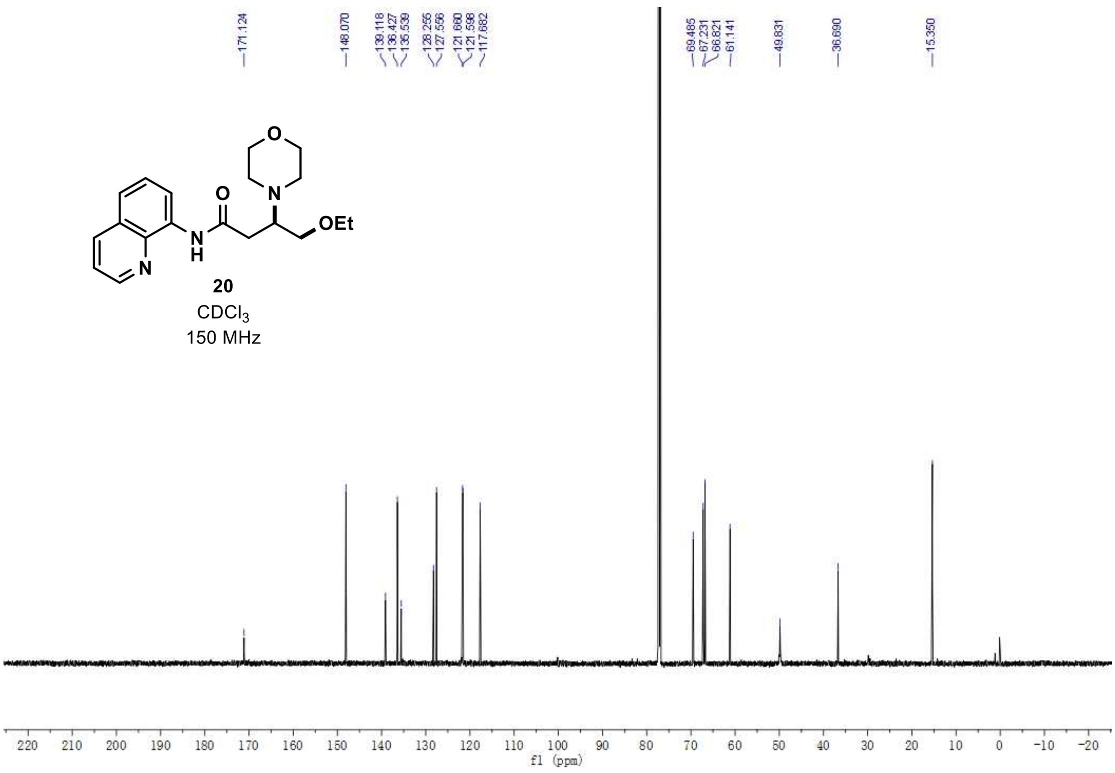

Figure S176. ^{13}C NMR Spectra of **20**.

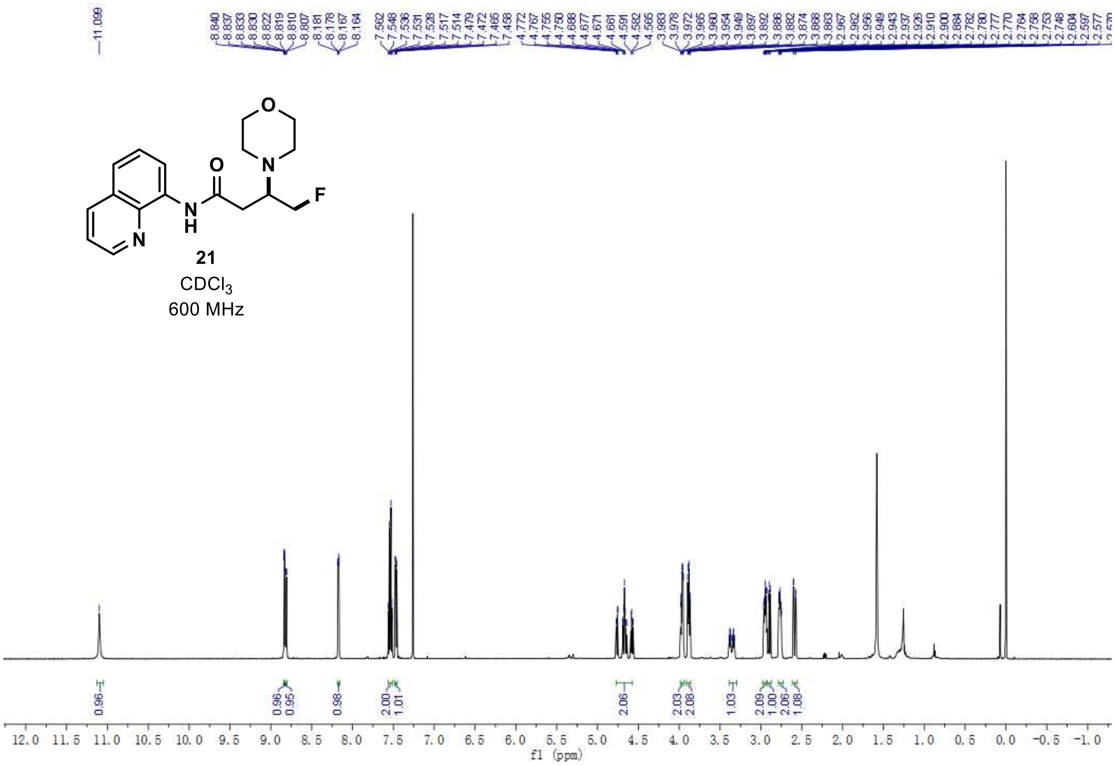

Figure S177. ^{1}H NMR Spectra of **21**.

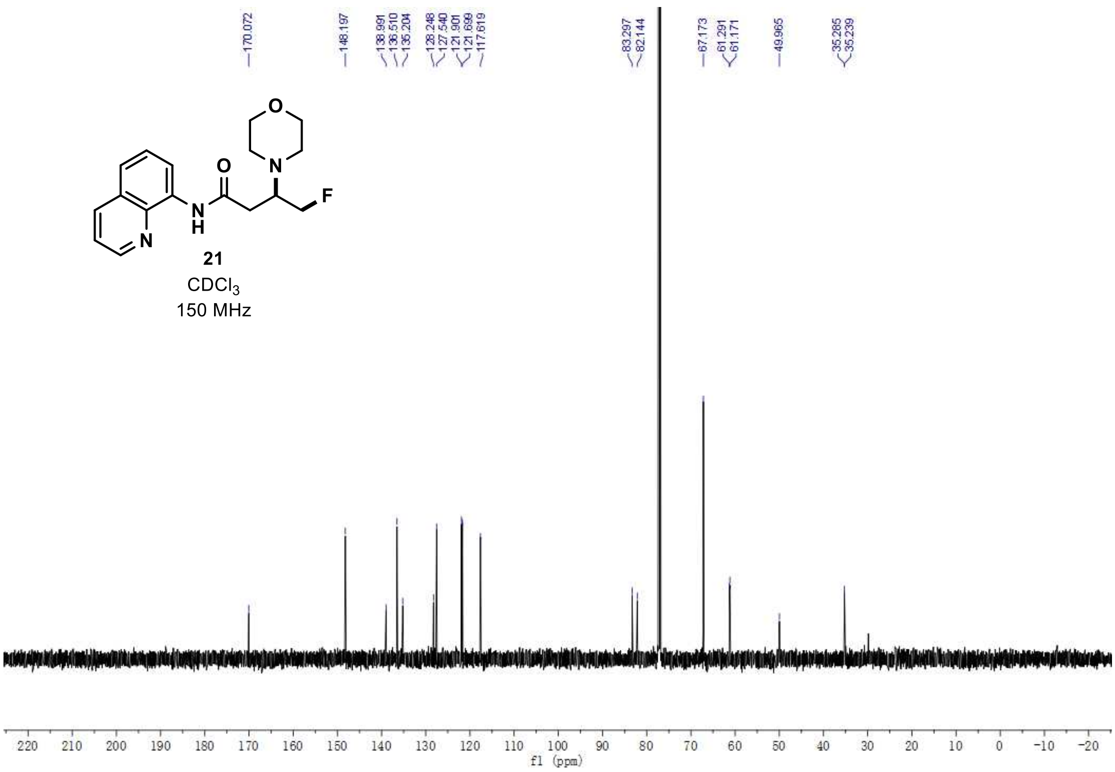

Figure S178. ^{13}C NMR Spectra of **21**.

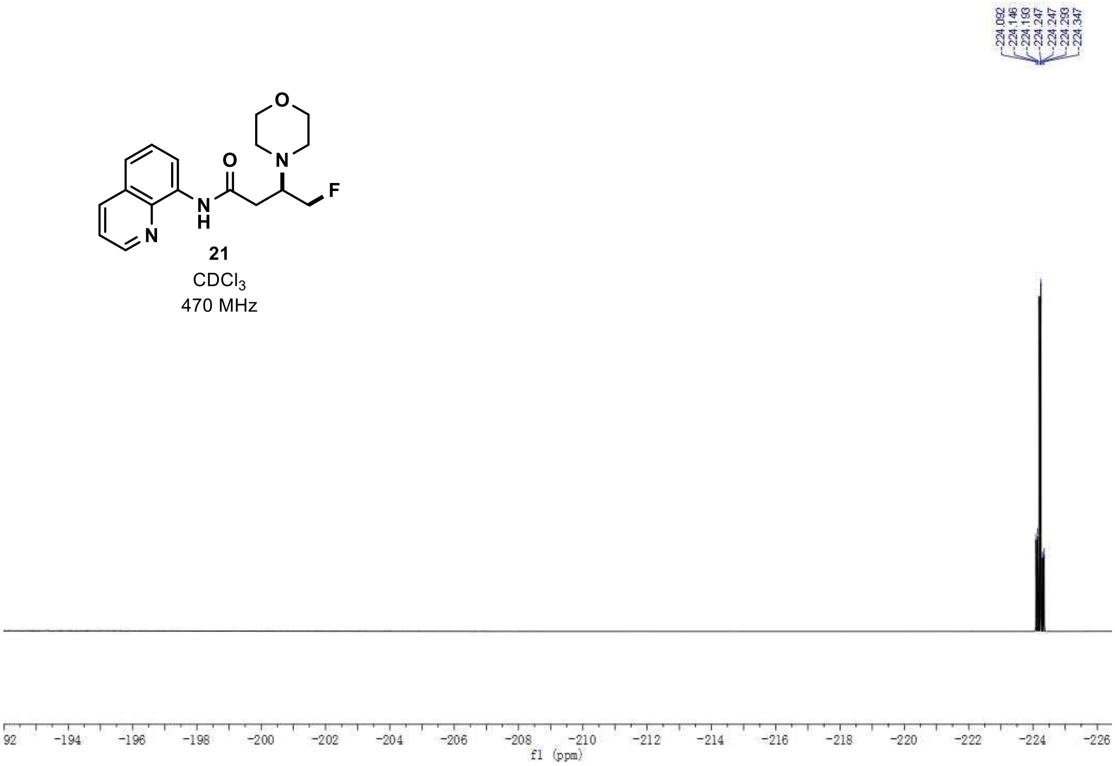

Figure S179. ^{19}F NMR Spectra of **21**.

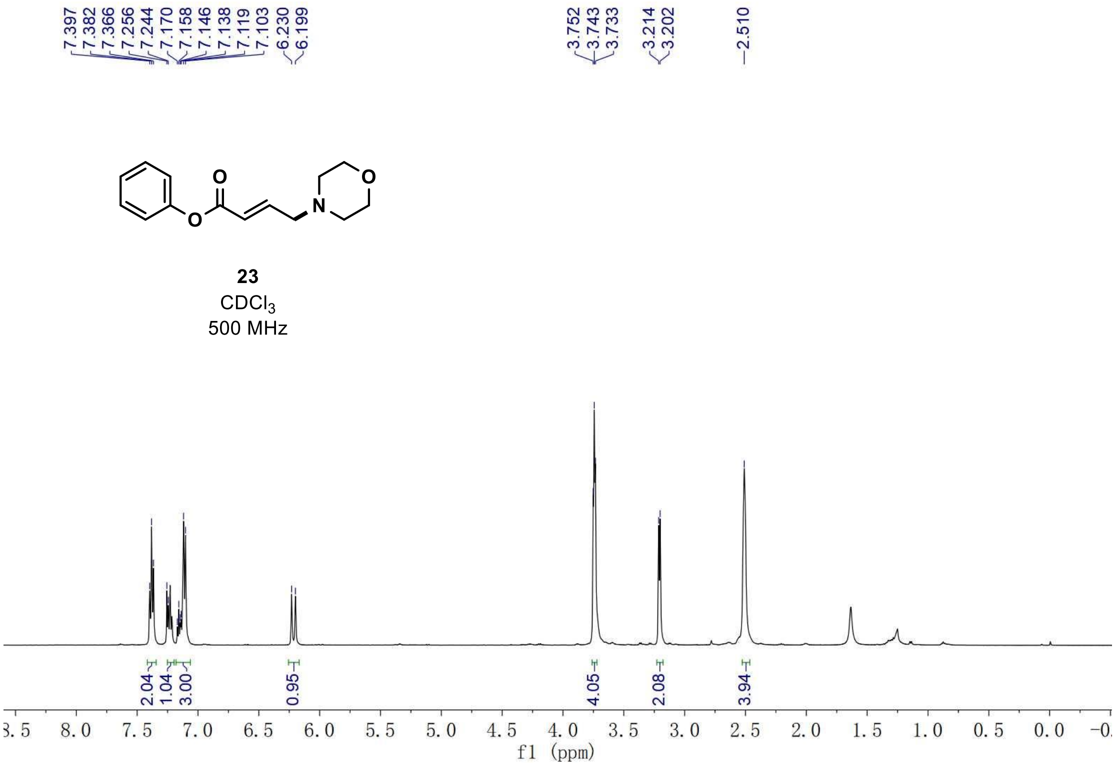

Figure S180. ^{1}H NMR Spectra of **23**.

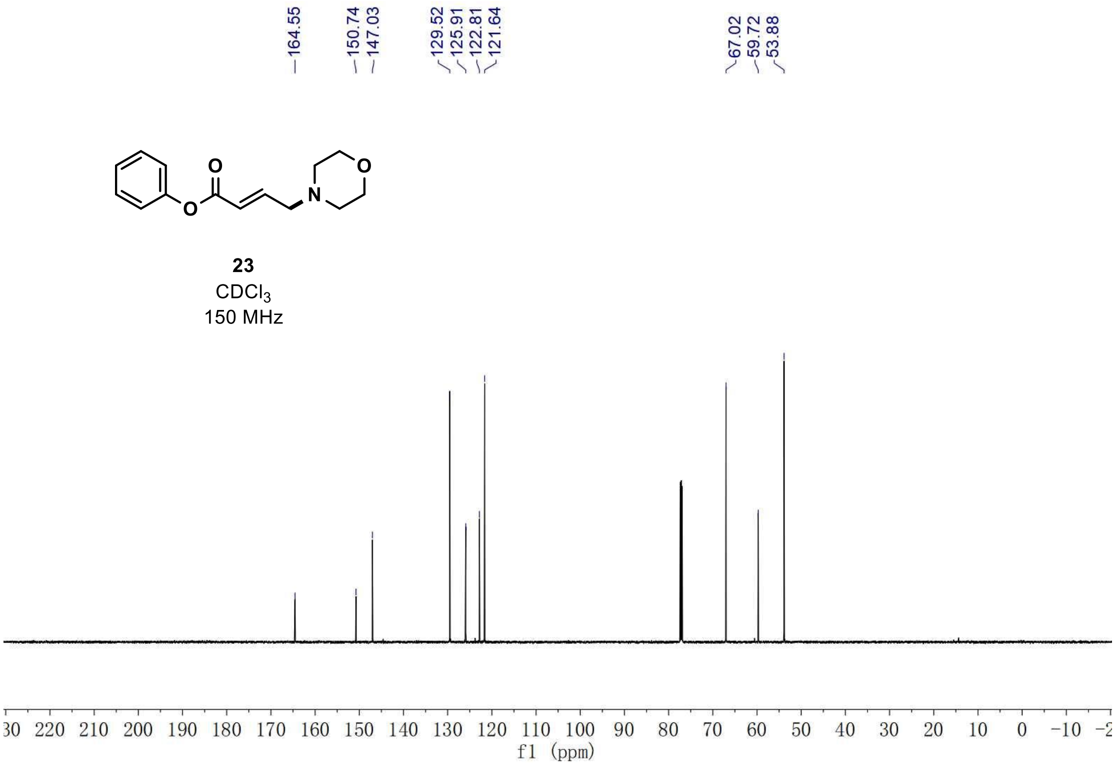

Figure S181. ^{13}C NMR Spectra of **23**.

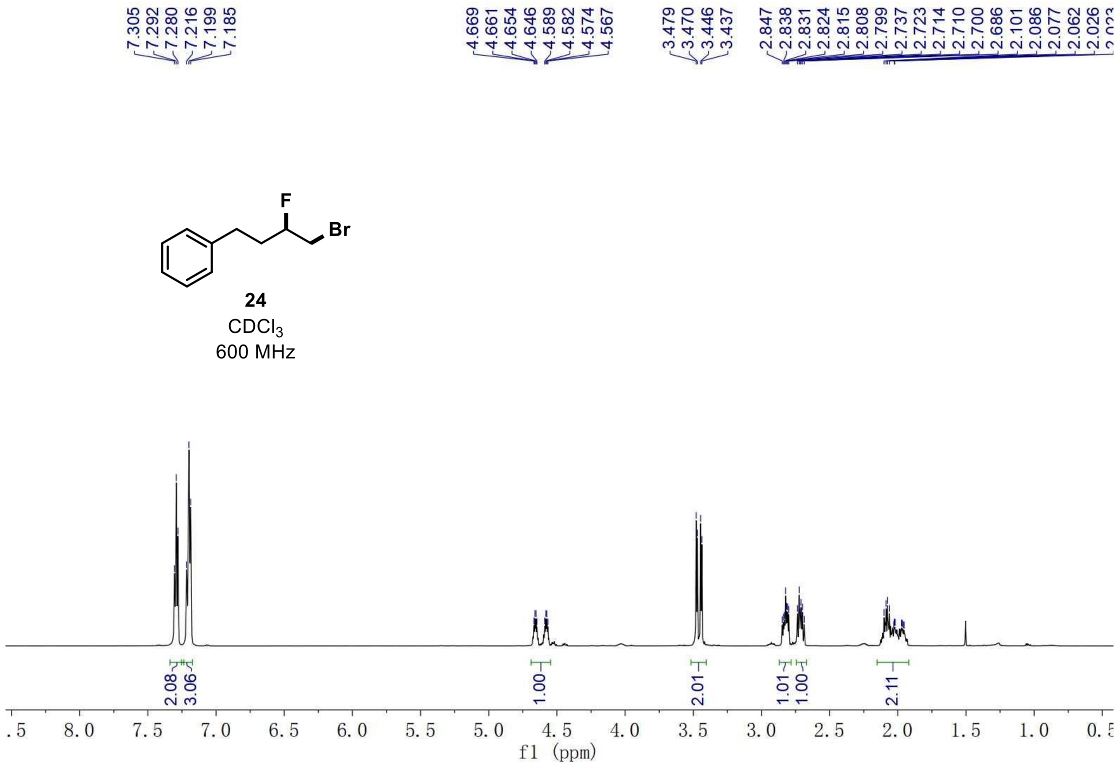

Figure S182. ^{1}H NMR Spectra of **24**.

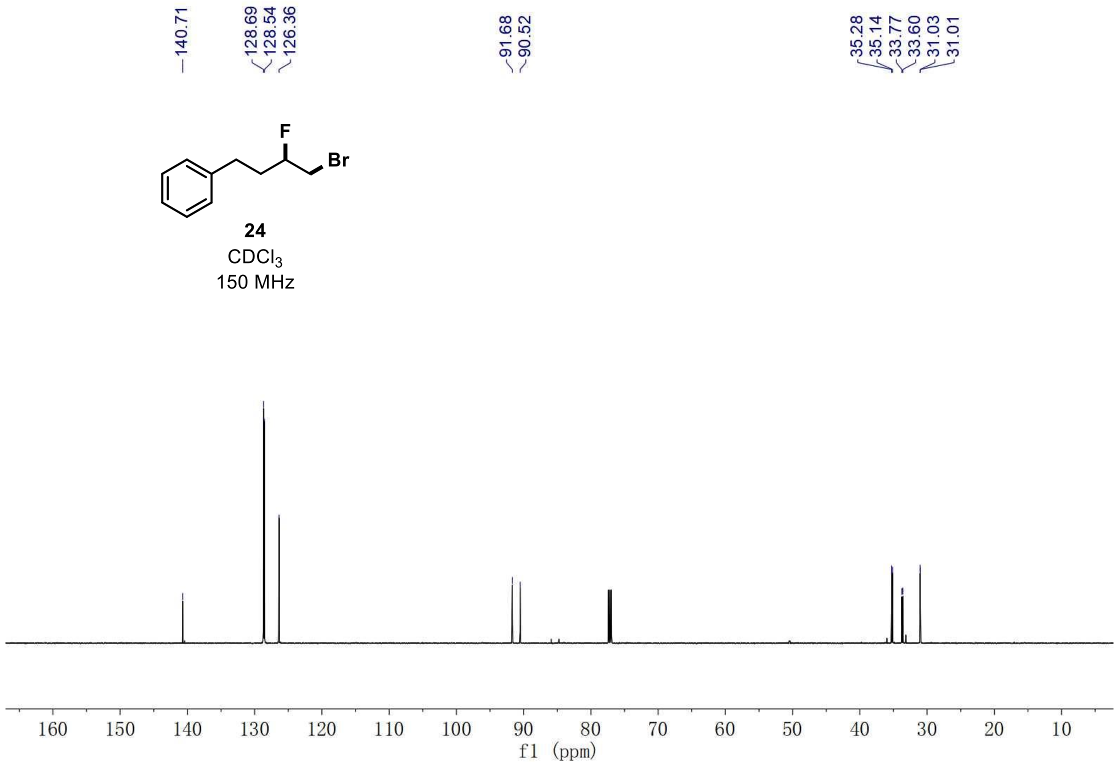

Figure S183. ^{13}C NMR Spectra of **24**.

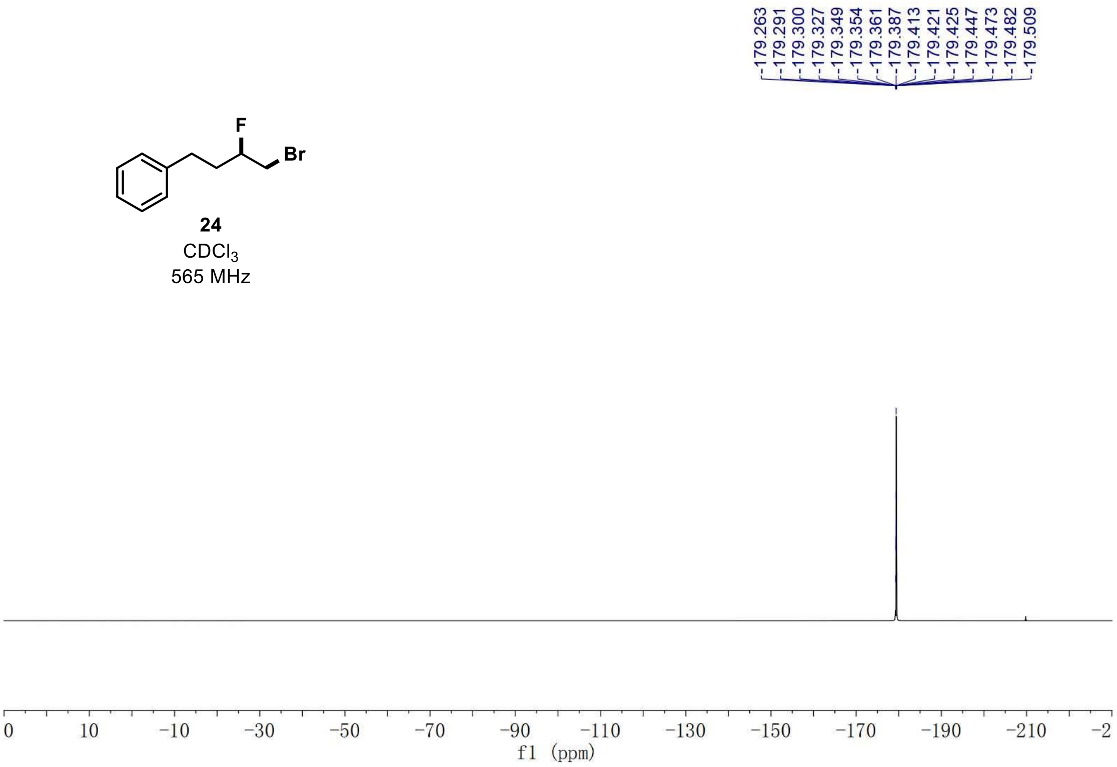

Figure S184. ^{19}F NMR Spectra of **24**.
